# Copper-Mediated Dehydrogenative C(sp^3^)–H Borylation of Alkanes

**DOI:** 10.1021/jacs.3c02185

**Published:** 2023-07-06

**Authors:** Ruocheng Sang, Wangyujing Han, Hanwen Zhang, Carla M. Saunders, Adam Noble, Varinder K. Aggarwal

**Affiliations:** School of Chemistry, University of Bristol, Cantock’s Close, Bristol BS8 1TS, U.K.

## Abstract

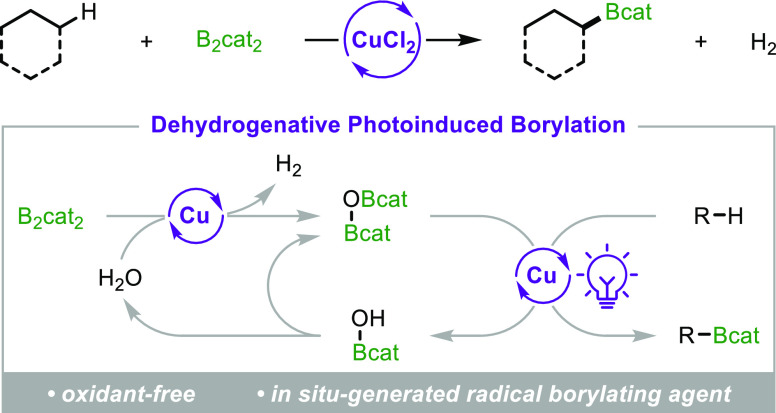

Borylations of inert carbon–hydrogen bonds are highly useful for transforming feedstock chemicals into versatile organoboron reagents. Catalysis of these reactions has historically relied on precious-metal complexes, which promote dehydrogenative borylations with diboron reagents under oxidant-free conditions. Recently, photoinduced radical-mediated borylations involving hydrogen atom transfer pathways have emerged as attractive alternatives because they provide complimentary regioselectivities and proceed under metal-free conditions. However, these net oxidative processes require stoichiometric oxidants and therefore cannot compete with the high atom economy of their precious-metal-catalyzed counterparts. Herein, we report that CuCl_2_ catalyzes radical-mediated, dehydrogenative C(sp^3^)–H borylations of alkanes with bis(catecholato)diboron under oxidant-free conditions. This is a result of an unexpected dual role of the copper catalyst, which promotes oxidation of the diboron reagent to generate an electrophilic bis-boryloxide that acts as an effective borylating agent in subsequent redox-neutral photocatalytic C–H borylations.

## Introduction

Boronic acids and esters are privileged functional groups in organic chemistry because they are air- and moisture-stable yet offer unparalleled diversity as reagents due to the versatile reactivity of the C–B bond.^1^ This has led to extensive research into the design of practical methodologies for their synthesis from readily available substrates. Arguably, the most direct approach for incorporating boron moieties into organic molecules is by substitution of C–H bonds, which provides the opportunity to convert unreactive feedstock chemicals into valuable reagents in a single step.^2^ Impressive developments in catalyst design have enabled borylations of C(sp^2^)–H bonds of aromatic rings to be achieved using readily available diboron reagents under mild conditions and with exquisite levels of regiocontrol. By contrast, borylations of C(sp^3^)–H bonds of non-activated alkanes remain synthetically challenging due to their lower reactivity toward commonly used precious-metal catalysts, which often necessitates the use of forcing conditions.^3^ Furthermore, the high selectivity of these catalysts for borylation of primary C–H bonds limits their application to reactions at more sterically hindered secondary sites unless a suitable directing group is installed.^4^ As a result, there is a need for alternative catalytic approaches for challenging C(sp^3^)–H borylations that proceed under mild conditions and provide regioselectivities complementary to those obtained with precious-metal catalysts.

**Figure 1:**
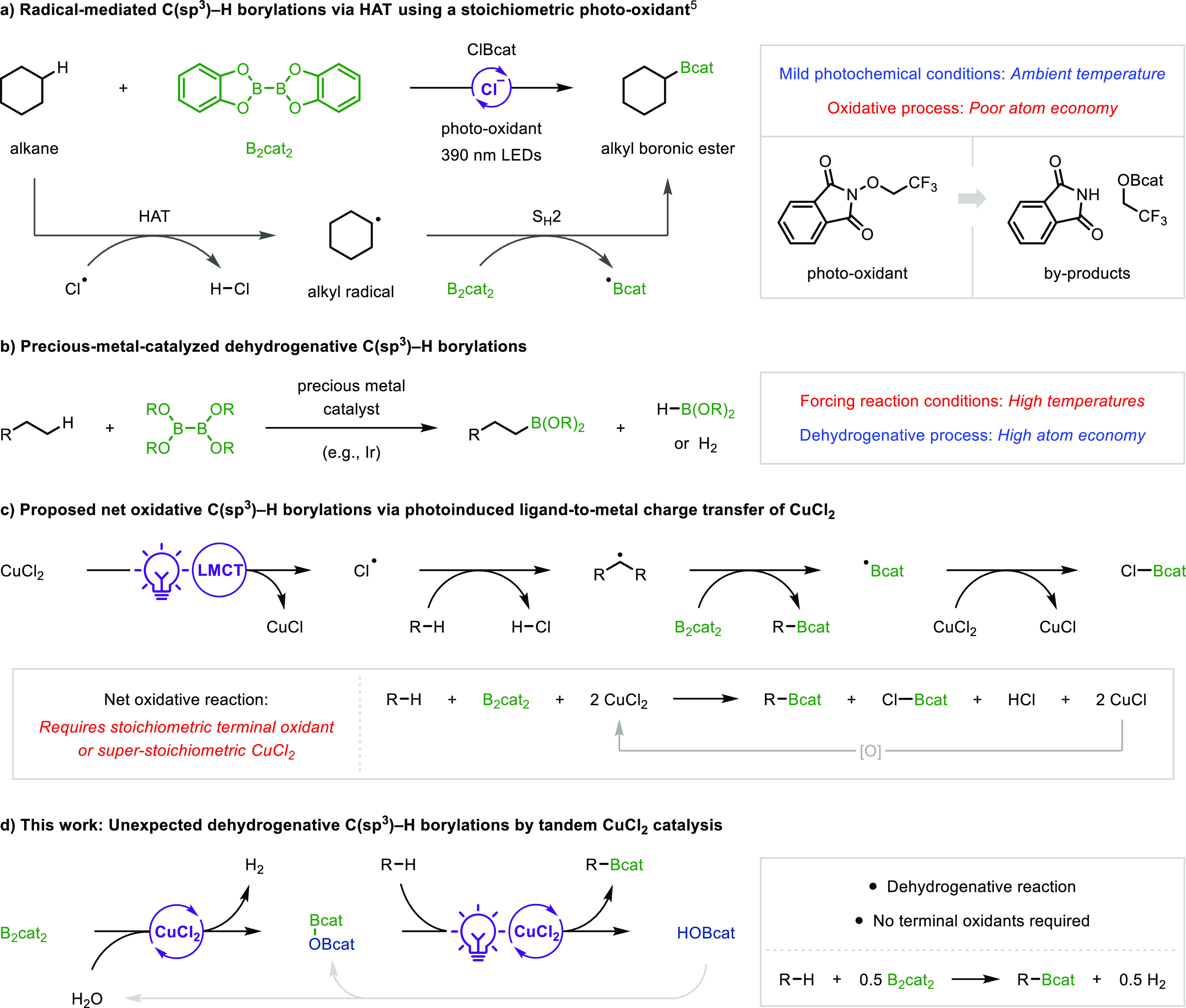
Borylations of non-activated alkanes with diboron reagents.

We recently reported the first example of a radical-mediated C(sp^3^)–H borylation of alkanes (Figure 1a).^5^ These reactions involve photoinduced generation of chlorine radicals from a chloride catalyst (*B*-chlorocatecholborane, ClBcat), which enables the cleavage of strong C(sp^3^)–H bonds by hydrogen atom transfer (HAT). The resulting alkyl radical is then intercepted by bis(catecholato)diboron (B_2_cat_2_) in a homolytic substitution reaction to generate alkyl boronic ester products. This methodology allows challenging C(sp^3^)–H borylations to be performed at ambient temperature and in the absence of metal catalysts;^6^ however, the overall oxidative process suffers from poor atom economy due to the necessary use of an *N*-alkoxyphthalimide as a stoichiometric photo-oxidant. By comparison, precious-metal-catalyzed borylations proceed under oxidant-free conditions because of the ability of the catalysts to undergo oxidative addition and reductive elimination reactions with both the diboron reagent and C–H bonds, which enables them to promote dehydrogenative reactions with the release of dihydrogen gas (H_2_) (Figure 1b).^3^ To provide more practical radical-mediated C(sp^3^)–H borylations, we sought to identify photocatalysts that could promote HAT-induced alkyl radical formation and borylation either with a commercially available and inexpensive terminal oxidant or, more preferably, whilst avoiding the need for oxidants altogether.

Considering potential photocatalysts, we were drawn to first-row transition-metal chlorides because these inexpensive salts can undergo photoinduced ligand-to-metal charge transfer (LMCT) to generate chlorine radicals,^7^ which we have shown to be suitable HAT species in borylation reactions.^5^ We identified cupric chloride (CuCl_2_) as a promising candidate since it was shown by Rovis to efficiently catalyze photoinduced alkylations of non-activated C(sp^3^)–H bonds with electron-deficient alkenes.^8^ In addition, copper(II) salts were successfully employed by MacMillan in photoinduced decarboxylative borylations of aryl carboxylic acids, thus demonstrating the compatibility of diboron reagents with LMCT-mediated radical formation.^9^ We reasoned that CuCl_2_ could catalyze related C(sp^3^)–H borylations; however, given that the overall process is net oxidative, a terminal oxidant would still be required (Figure 1c).^10^ Nonetheless, we were encouraged by recent examples of net oxidative photoinduced decarboxylative functionalization reactions wherein Cu(II) salts promote radical formation by photoinduced LMCT and also function as terminal oxidants.^11^ Therefore, a simpler and more economic radical-mediated C(sp^3^)–H borylation protocol could still be achieved by replacing ClBcat and a non-commercial *N*-alkoxyphthalimide photo-oxidant with stoichiometric quantities of inexpensive CuCl_2_. Herein, we report that CuCl_2_ can indeed promote C(sp^3^)–H borylations of alkanes with B_2_cat_2_ in the absence of additional stoichiometric reagents. However, contrary to our expectation, we found that the reaction could be rendered catalytic in CuCl_2_; thus, the use of stoichiometric oxidants could be eliminated altogether. This was found to result from a CuCl_2_-catalyzed dehydrogenative reaction between B_2_cat_2_ and water to generate the bis-boryloxide O(Bcat)_2_, which was found to be an efficient electrophilic borylating agent for subsequent redox-neutral LMCT-mediated C(sp^3^)–H borylations (Figure 1d). This mechanistically distinct, tandem catalytic process was applicable to the borylation of a wide range of alkanes, with the reaction efficiencies and functional group tolerance outperforming that of our previously reported metal-free protocol.^5^ Indeed, some simple substrates, such as tetrahydrofuran, which could not be borylated under our previous conditions, now undergo successful borylation. The reactions also display regioselectivities that contrast those expected for chlorine radical-mediated HAT, predominantly giving products of borylation of strong, non-activated C(sp^3^)–H bonds rather than weaker tertiary and α-heteroatom positions.

## Results and Discussion

### Reaction Development

We began by investigating the borylation of cyclohexane with B_2_cat_2_ using 2 equiv of CuCl_2_ in acetonitrile (MeCN) under irradiation with violet LEDs (390 nm), with the expectation that the excess CuCl_2_ should generate chlorine radicals via photoinduced LMCT and function as a terminal oxidant (Table 1). We were pleased to find that these conditions allowed efficient C(sp^3^)–H borylation to occur, with boronic ester **1** formed in 76% yield after ligand exchange of the hydrolytically labile catechol boronic ester to the stable pinacol boronic ester (entry 1). We subsequently studied the influence of the copper loading and were surprised to observe almost identical reaction efficiency when the amount of CuCl_2_ was reduced to 20 mol % (entries 2–3). Control experiments revealed the essential roles of metal chlorides, B_2_cat_2_, and light in this transformation (entries 4–6, 0% yield). Additional screening of catalysts, solvents, diboron reagents, and the use of LiCl to improve CuCl_2_ solubility^8,12^ failed to provide any improvements (entries 7–8, see Section 2.3 of the Supporting Information for additional optimization studies). However, we observed an interesting enhancement in the yield of **1** when CuCl_2_ and B_2_cat_2_ were prestirred in MeCN in the dark for 10 h before adding cyclohexane and irradiating (entries 9–10), which suggested that a thermally promoted background reaction between CuCl_2_ and B_2_cat_2_ was beneficial for the subsequent photoinduced borylation reaction.

**Table 1:**
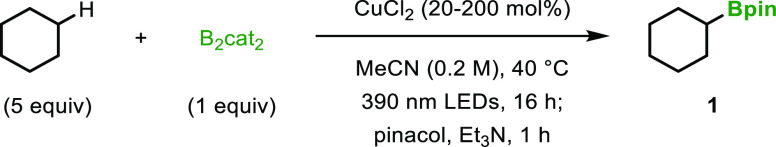
Optimization of Reaction Conditions

entry	conditions	yield of **1** (%)^a^
1	200 mol % CuCl_2_	76%
2	100 mol % CuCl_2_	74%
3	20 mol % CuCl_2_	72%
4	20 mol % CuBr_2,_ no CuCl_2_	0%
5	20 mol % CuCl_2_, B_2_pin_2_, no B_2_cat_2_	0%
6	20 mol % CuCl_2_, no light	0%
7	20 mol % FeCl_3,_ no CuCl_2_	51%
8	20 mol % CuCl_2,_ 50% LiCl	64%
with pre-stirring of CuCl_2_ and B_2_cat_2_ for 10 h		
9	100 mol % CuCl_2_	83%
10	20 mol % CuCl_2_	77%

a Yields were determined by GC-FID analysis.

### Preliminary Mechanistic Investigation

**Figure 2:**
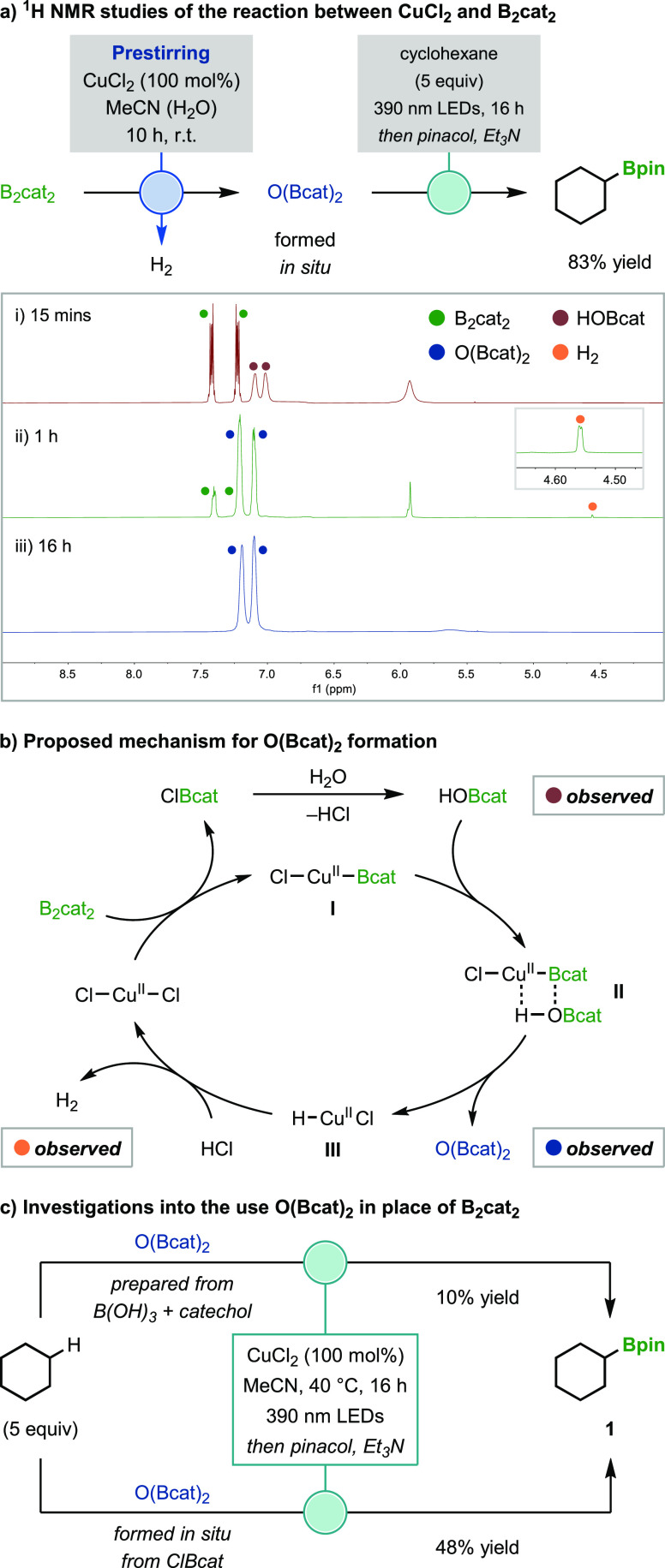
Preliminary mechanistic investigations. Yields were determined by GC-FID analysis.

These intriguing results prompted us to investigate the mechanism to rationalize the unexpected catalytic activity of CuCl_2_ and the beneficial effect of pre-stirring with B_2_cat_2_. We studied the reaction between CuCl_2_ and B_2_cat_2_ at room temperature in CD_3_CN by ^1^H and ^11^B NMR (Figures 2a and S6, S7), which revealed that B_2_cat_2_ was rapidly consumed by hydrolytic B–B bond cleavage by trace water impurities in the solvent to generate two new boron species: the hydrogen borate HOBcat and the bis-boryloxide O(Bcat)_2_. Complete conversion of B_2_cat_2_ to O(Bcat)_2_ occurred within 10 h, with HOBcat being a relatively short-lived intermediate only observed in the first 15 min of the reaction, whereas no B–B bond cleavage was observed in the absence of CuCl_2_. A proposed mechanism for this Cu-catalyzed hydrolytic cleavage of B_2_cat_2_ involves initial transmetalation with CuCl_2_ to generate Cu(II)-boryl complex **I** and ClBcat (Figure 2b), which has previously been reported for Cu(II) chlorides^13^ and other Cu(II) complexes.^14^ The electrophilic ClBcat should then undergo rapid hydrolysis by trace water in the solvent to form HOBcat and hydrogen chloride (HCl). Subsequent σ-bond metathesis between HOBcat and **I** via transition state **II** leads to O(Bcat)_2_ and Cu(II)-hydride **III**.^15^ Finally, ligand exchange of **III** with HCl regenerates CuCl_2_ with the liberation of H_2_, which was confirmed by ^1^H NMR (Figure 2a, spectrum ii). Interestingly, while dehydrogenative reactions between diboron reagents and H_2_O have previously been reported with palladium catalysts,^16^ they are unknown using copper catalysis.

The discovery of the complete conversion of B_2_cat_2_ to O(Bcat)_2_ prior to irradiation clearly indicates that B_2_cat_2_ is not responsible for C–B bond formation in the subsequent C(sp^3^)–H borylation. Therefore, we evaluated the effectiveness of independently synthesized O(Bcat)_2_ as a borylating agent in the reaction of cyclohexane (Figure 2c). Using O(Bcat)_2_ prepared by condensation of B(OH)_3_ with catechol provided 10% of **1**, thus demonstrating a productive reaction, albeit in poor yield due to difficulty in obtaining O(Bcat)_2_ in high purity owing to its air and moisture sensitivity. To overcome this issue, we prepared O(Bcat)_2_ in situ by hydrolysis of ClBcat, which gave boronic ester **1** in an improved 48% yield. Based on these results, we concluded that O(Bcat)_2_ was the borylating agent in the C(sp^3^)–H borylation reaction. Crucially, the CuCl_2_-catalyzed conversion of B_2_cat_2_ to O(Bcat)_2_ reverses the polarity of the borylating agent by transforming the nucleophilic diboron into an electrophilic bis-boryloxide. This means that the subsequent photoinduced LMCT-mediated borylation becomes a redox-neutral process and therefore catalytic in CuCl_2_. To our knowledge, this represents the first report of bis-boryloxides being used as borylating agents for alkyl radicals and could have useful implications in radical-mediated borylation reactions by providing a polarity reversal from commonly used, nucleophilic diboron reagents.^5,6,17^

### Substrate Scope

**Figure 3:**
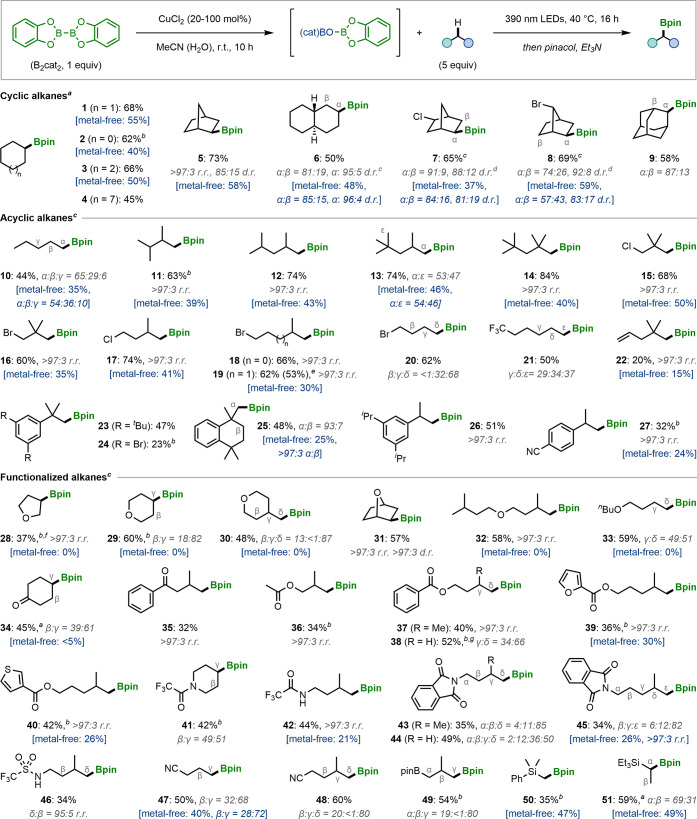
Scope of C–H borylation reactions. Conditions: B_2_cat_2_ (0.3 mmol) and CuCl_2_ (20–100 mol %) in MeCN (1.5 mL, 0.2 M) were pre-stirred for 10 h, then alkane (5 equiv) was added and irradiated (390 nm Kessil lamps) for 16 h. Yields are of isolated products. Regioisomeric ratios (r.r.) and diastereomeric ratios (d.r.) were determined by GC analysis. Only the d.r. of the major regioisomer is shown, and the d.r. of the minor is given in the Supporting Information. The metal-free yields refer to those obtained using the conditions reported in a previous study.^5^^*a*^Using 20 mol % CuCl_2_. ^*b*^Reactions performed without pre-stirring CuCl_2_ and B_2_cat_2_. ^*c*^Using 100 mol % CuCl_2_. ^*d*^The r.r. and d.r. were determined by ^1^H NMR analysis after oxidation to the corresponding alcohol. ^*e*^Reaction was performed on a 3 mmol scale. ^*f*^Using alkane (20 equiv), 200 mol % CuCl_2_, and 40 mol % pyridine. ^*g*^The r.r. was determined by ^1^H NMR analysis.

With optimized conditions in hand, the scope of this photoinduced C(sp^3^)–H borylation was explored (Figure 3). Using 20 mol % CuCl_2_, cyclic alkanes were converted to alkyl boronic esters in good yields and with high regio- and diastereoselectivities (**1–9**). Borylations were found to occur selectively at less sterically hindered secondary C–H bonds, with no products derived from the reaction of tertiary C–H bonds observed, except for adamantane (**9**) which gave the tertiary boronic ester as a minor product. A broad range of acyclic alkanes was also successfully borylated (**10**–**22**). Although the borylation of these substrates was successful with catalytic CuCl_2_, significantly higher yields were obtained with an increased CuCl_2_ loading of 100 mol % (see Section 3.1 of the Supporting Information). The reactions display high regioselectivities for primary over secondary and tertiary C–H bonds, which is the opposite trend to that expected for C–H bond cleavage via HAT to chlorine radicals,^18^ including those generated by LMCT.^8,19^ The high selectivity for borylation of sterically less hindered C–H bonds matches our previous metal-free protocol,^5^ where the presence of catecholborate species was proposed to override the inherent selectivity of chlorine radical-mediated HAT, which is generally determined by C–H bond dissociation energy (BDE).^20^ In addition to steric effects, electronics also influenced the regioselectivity, with no C–B bond formation observed proximal to electron-withdrawing groups (**15**–**21**). Alkyl halides (**15**–**19**) and terminal alkenes (**22**) were tolerated despite the potential for side-reactions via dehalogenation and diboration processes, respectively.^13,21^ Considering the apparent sensitivity of the reaction to sterics, we were pleased to find that *tert*-butyl groups were successfully borylated (**13**–**16**),^22^ which contrasts the selectivity of precious-metal-catalyzed protocols.^3a,22,23^ Alkyl substituted electron-rich, -poor and -neutral arenes (**23**–**27**) exclusively provided C(sp^3^)–H borylated products.^3c^ High selectivities for borylation of methyl groups over both aromatic and the much weaker benzylic C–H bonds were observed.^20^ Unfortunately, benzylic methyl groups could not be borylated, most likely because oxidation of the benzylic radical to a carbocation outcompetes borylation (see Section 3.7.5 of the Supporting Information for evidence of benzylic carbocation formation).

We subsequently extended the scope of the transformation beyond structurally simple alkanes by employing more functionalized substrates (Figure 3), particularly those with oxygen- and nitrogen-containing groups that were unsuccessful in our metal-free borylation protocol.^5^ A wide range of synthetically useful functional groups was tolerated, including ethers (**28–33**), ketones (**34**, **35**), esters (**36–38**), heteraromatics (**39**, **40**), amides (**41**, **42**), imides (**43–45**), sulfonamides (**46**), nitriles (**47**, **48**), pinacol boronic esters (**49**), and silanes (**50**, **51**). Previous reports of HAT-mediated functionalizations of ethers are dominated by reactions that display high regioselectivity for substitution of C–H bonds α to oxygen;^24^ as a result, we were surprised to observe that borylation occurred selectively at positions distal to the oxygen and no α-oxy boronic ester products were detected (**28**–**33**). Like our hypothesis for the failed borylation of benzylic methyl groups, we suspect that this is a result of competitive single-electron oxidation of the α-oxy radicals to oxocarbenium ions. Notably, ketones were tolerated in our C–H borylation (**34**, **35**), whereas these substrates represent a limitation of precious-metal-catalyzed reactions,^3c^ presumably due to competitive nucleophilic borylations of the carbonyl.^25^ A current limitation of this methodology is the low reactivity of substrates bearing easily oxidizable or strongly basic amines (Figure S3). However, we found that both secondary and primary amines could be borylated upon protection as trifluoroacetamides (**41**, **42**), imides (**43–45**), or sulfonamides (**46**). Judicious selection of the N-protecting group was found to be crucial since no boronic ester products were observed for *tert*-butoxycarbonyl-, benzoyl-, or acetyl-protected amines. Once again, C–B bond formation occurred selectively at positions distal to the nitrogen functionality, with no α-amino boronic esters formed. Interestingly, borylation of tetraethylsilane selectively gave the α-silyl boronic ester **51**, which suggests that favorable HAT α to silicon overrides the typically observed selectivity for borylation of sterically less hindered C–H bonds. We also investigated the impact of the pre-stirring protocol to fully transform B_2_cat_2_ to O(Bcat)_2_ prior to irradiation and found that it was beneficial in almost all cases (see Section 2.5 of the Supporting Information). Although improvements in reaction efficiency were often modest, dramatically enhanced yields were observed in some cases, including for alkanes (**8**, **13**–**15**, **19**–**20**), alkyl arenes (**26**), and functionalized substrates (**32**, **34**–**35**, **46**, **51**).

**Figure 4:**
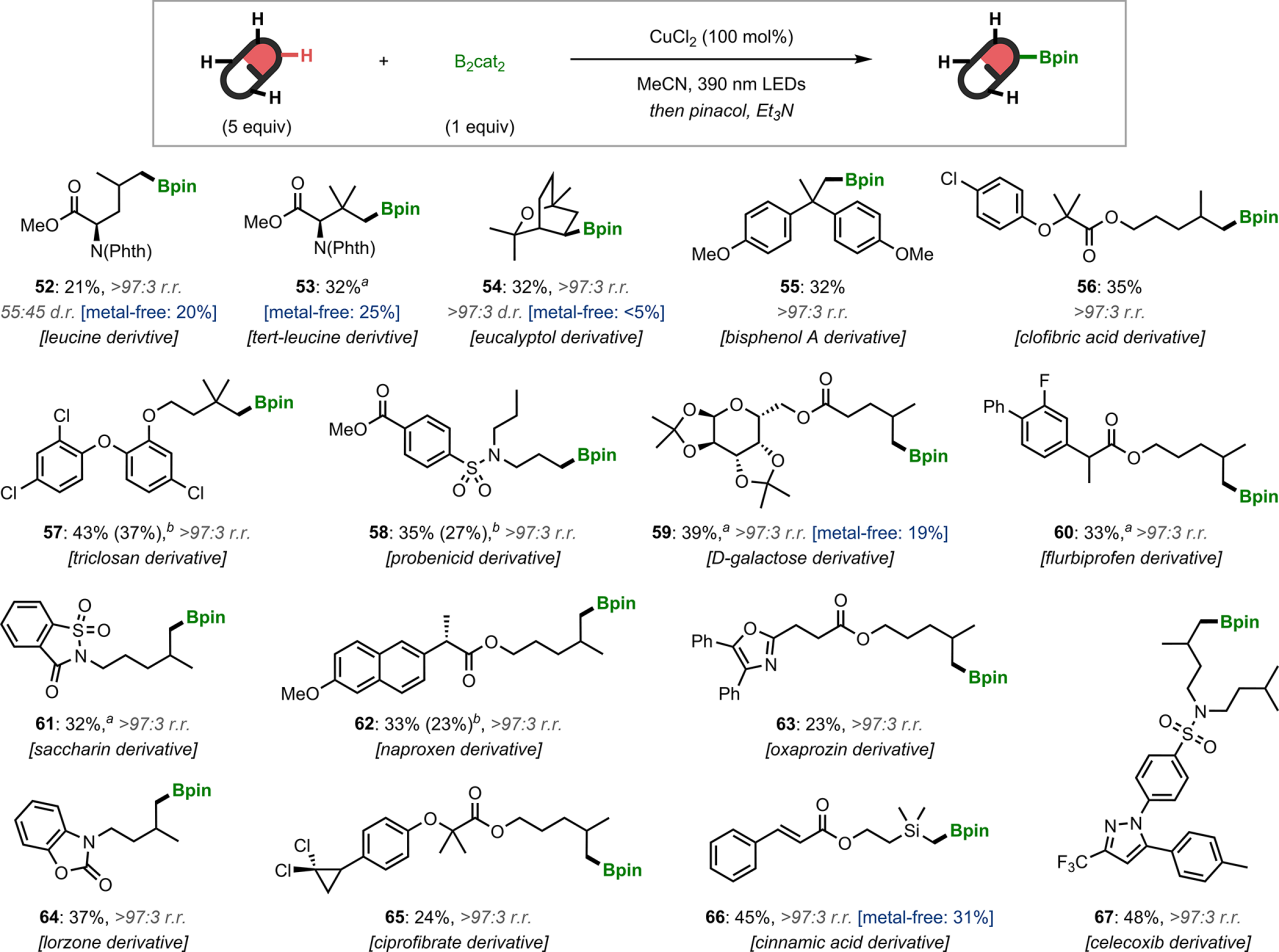
C–H borylations of natural products and drug derivatives. Conditions: B_2_cat_2_ (0.3 mmol), alkane (5 equiv), CuCl_2_ (100 mol %), MeCN (1.5 mL, 0.2 M), 390 nm Kessil lamps, 40 °C, 16 h. Yields are of isolated products. Regioisomeric ratios (r.r.) and diastereomeric ratios (d.r.) were determined by GC analysis. The metal-free yields refer to those obtained using the conditions reported in ref (5). ^*a*^Reactions performed by pre-stirring CuCl_2_ and B_2_cat_2_ at r.t. for 10 h before adding the alkane and irradiating. ^*b*^The yields in parentheses were obtained using the alkane as the limiting reagent under the following conditions: alkane (0.3 mmol, 1 equiv), B_2_cat_2_ (0.6 mmol, 2 equiv), CuCl_2_ (200 mol %), MeCN (1.5 mL, 0.2 M), 390 nm Kessil lamps, 40 °C, 24 h. N(phth): *N*-phthalimido.

To further demonstrate the regio- and chemoselectivity of this methodology, it was applied to a range of complex molecules (Figure 4). Derivatives of natural products (**52**–**54**, **59**, **66**) and drugs (**56**, **58**, **60**, **62**–**65**, **67**) were successfully borylated with excellent regioselectivities. As observed previously, borylation occurred selectively at methyl groups distal to heteroatoms. Notably, substrates possessing Lewis basic aza-heteroaromatic rings (**63**, **67**), activated alkenes (**66**), and benzylic methyl groups were tolerated (**67**). Interestingly, eucalyptol gave a 32% yield of secondary boronic ester **54** as a single regio- and diastereoisomer, thus demonstrating the excellent selectivity of this borylation reaction compared to other HAT-mediated C–H functionalizations of this substrate.^26^ Next, we addressed the stoichiometry of the alkane, which is used in a large excess under our standard conditions. Although we found that unreacted alkanes could be recovered in excellent yields (Tables S9, S11, and S13–S15), the use of 5 equiv of a complex substrate is undesirable; therefore we sought to demonstrate that fewer equivalents could be used without significant reduction in the reaction efficiency. The syntheses of boronic esters **57**, **58**, and **62** were performed using reduced alkane loading (3 equiv), and we were pleased to find that this modification did not lead to substantial reduction in yields (Tables S13–S15). Remarkably, similar yields could also be obtained when using the alkane as the limiting reagent with 2 equiv of B_2_cat_2_, and this stoichiometry was also found to provide synthetically useful yields in the borylation of simple alkanes (Tables S8, S9, and S11).

After the generality of our photoinduced LMCT-enabled borylation was explored, the efficiency of this protocol was benchmarked against our previously reported metal-free borylation.^5^ The current protocol represents a simpler and more economical option for C(sp^3^)–H borylation, given the low cost of CuCl_2_ compared to ClBcat and the *N*-alkoxyphthalimide photo-oxidant. In terms of the reaction mechanism, the generation of chlorine radicals by LMCT of CuCl_2_ is more efficient than the intermolecular SET process in our metal-free borylation,^7^ which led to improvements in yields (see comparisons in Figures 3, 4, and S2) and allowed the reactions to be performed with decreased alkane stoichiometry (5 vs 10 equiv). Importantly, substrates that were found to be incompatible under our metal-free conditions, such as ketones, ethers, and cyclic amides, could be successfully borylated using CuCl_2_. Additionally, enhanced regioselectivities were observed for several substrates (**7**, **8**, **10**), although this was not always the case, since some products were formed with comparable (**13**, **47**) or lower (**6**, **25**, **45**) regioselectivities.

### Mechanistic Study

**Figure 5:**
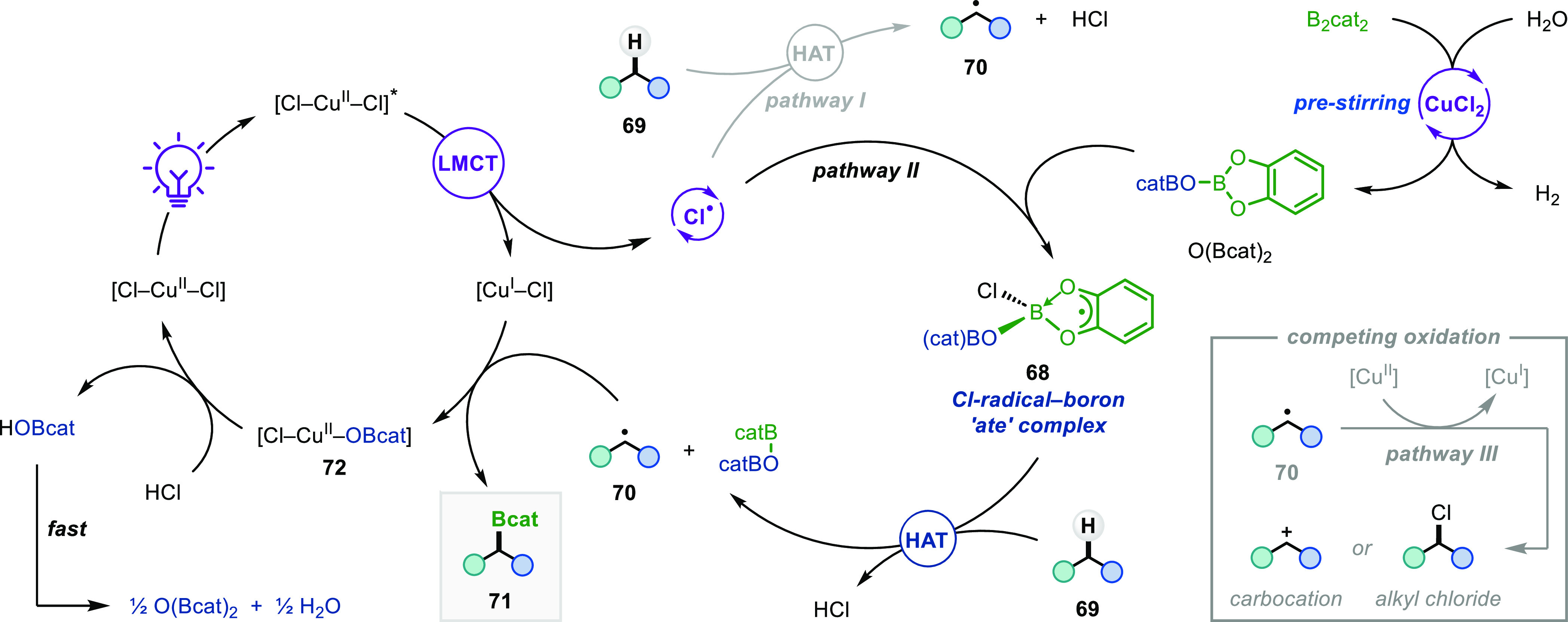
Proposed mechanism.

Our proposed mechanism for this photoinduced C–H borylation is shown in Figure 5. The reaction begins with CuCl_2_-catalyzed oxidative cleavage of B_2_cat_2_ with water to produce O(Bcat)_2_ as a novel electrophilic borylating agent. Irradiation then enables photoexcitation of CuCl_2_ and LMCT to generate chlorine radicals.^7^ The regioselectivity of alkyl radical formation by HAT from alkanes to chlorine radicals is generally dictated by the C–H BDE, so follows the reactivity trend tertiary > secondary > primary.^27^ This predictable site-selectivity has been observed in C–H functionalizations mediated by chlorine radicals generated by both single-electron oxidation of chloride^18^ and LMCT from transition-metal chlorides.^8,19^ The high selectivity for borylation of primary C–H bonds in our protocol indicates that HAT to “free” chlorine radicals (pathway I) is unlikely to be the dominant pathway.^28^ Therefore, we propose that chlorine radicals formed via LMCT first react with O(Bcat)_2_ to afford chlorine radical–boron “ate” complex **68**,^5^ where the unpaired electron is delocalized onto the catecholate ligand.^29^ Alkyl radical formation then occurs through the selective cleavage of sterically unhindered C–H bonds of alkanes **69** by **68**, where the steric constraints of this radical “ate” complex override the inherent thermodynamic preference for HAT from weaker C–H bonds.^30^ Comparison of the regioselectivities obtained in this work with those of our previously reported metal-free protocol (see Figure 3) indicate that related but structurally distinct radical–boron “ate” complexes are involved in alkyl radical generation.^5^ Subsequent C–B bond formation occurs by the reaction of alkyl radical **70** with O(Bcat)_2_, giving boronic ester **71** and copper(II) borate **72**. We investigated the C–B bond forming step using density functional theory (DFT) calculations and found that direct homolytic substitution of O(Bcat)_2_ by alkyl radicals is kinetically and thermodynamically disfavored; therefore, copper plays an important role in this step (see Section 4 of the Supporting Information). Turnover of the copper catalyst is then achieved by protodemetalation of **72** with HCl to give HOBcat.

**Figure 6:**
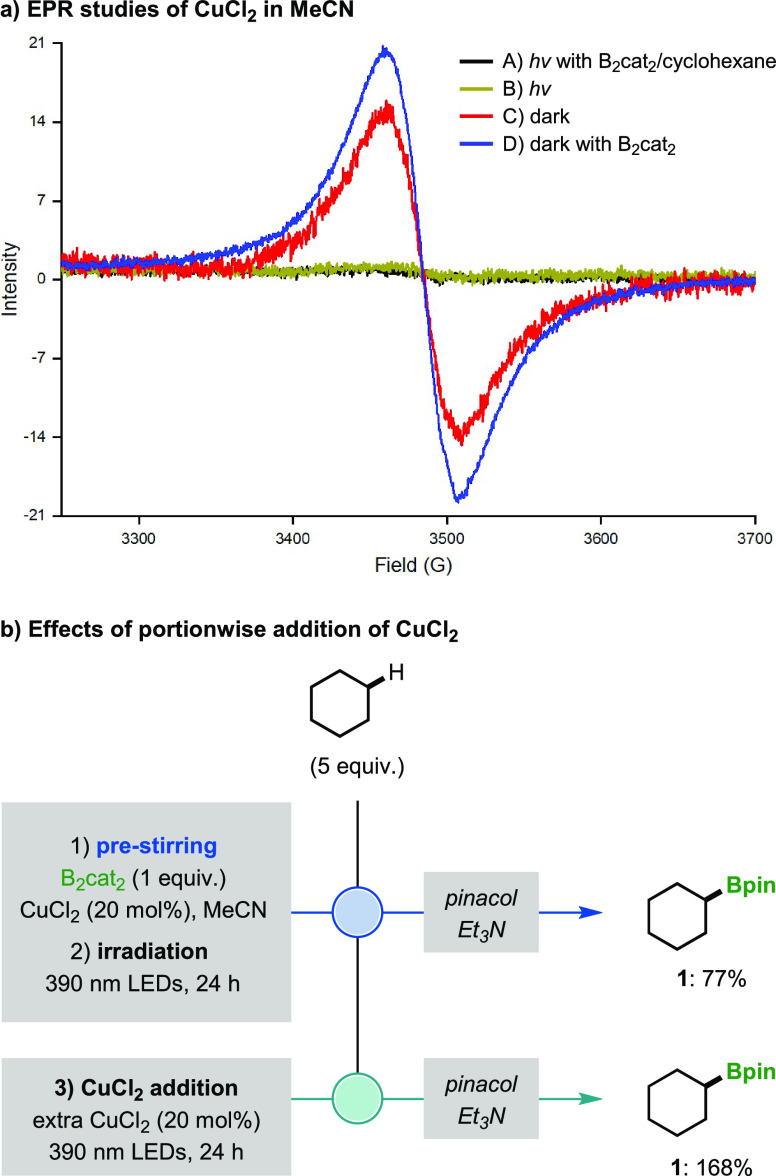
Mechanistic studies. Yields were determined by GC-FID analysis. The yield is based on the moles of product per mole of B_2_cat_2_; a yield above 100% reflects the conversion of the HOBcat by-product to the boronic ester product.

Based on this mechanism, we expected to form HOBcat as the by-product of the photoinduced borylation but were surprised to observe only O(Bcat)_2_ upon NMR analysis of the crude reaction mixture (Figures S16–S18). As a result, we propose that condensation of two molecules of HOBcat occurs rapidly in the presence of HCl and CuCl_2_ as dehydrating agents, thus leading to the formation of more of the active borylating agent O(Bcat)_2_ (Figure 5). Interestingly, regeneration of H_2_O during this process means that it is acting as a co-catalyst in the tandem copper catalytic process, which provides an explanation for the success of the reaction even when anhydrous solvents were used (see Table S5). This result also poses the question: if O(Bcat)_2_ is formed as a product, why does the borylation reaction stop proceeding? Since we found that extension of irradiation time did not facilitate complete conversion of O(Bcat)_2_, and the unreacted alkane could be recovered in high yield, termination of the reaction must be due to the full consumption of CuCl_2_. This was confirmed by electron paramagnetic resonance (EPR) spectroscopy (Figure 6a), which showed that complete consumption of CuCl_2_ occurred during the borylation of cyclohexane (Spectrum A). Reduction of CuCl_2_ also occurred upon irradiation in the absence of B_2_cat_2_ and cyclohexane (Spectrum B),^12^ whereas no reduction occurred in the dark (Spectra C and D). During the borylation reaction, consumption of CuCl_2_ likely occurs due to deleterious single-electron oxidation or chlorination of alkyl radicals **70** (pathway III, Figure 5),^12^ which competes with C–B bond formation and reduces the catalytically active CuCl_2_ to CuCl. Further evidence that the reaction termination is due to consumption of CuCl_2_ was obtained by successfully restarting the reaction upon adding more CuCl_2_ (Figure 6b). For the borylation of cyclohexane, 20 mol % CuCl_2_ provided 77% of boronic ester **1** after 24 h and this yield did not increase if irradiation was continued for an additional 48 h (see Section 3.6 of the Supporting Information). However, when a further 20 mol % CuCl_2_ was added after 24 h and irradiation continued for another 24 h, **1** was formed in a remarkable 168% yield. This result confirms that incomplete O(Bcat)_2_ conversion was a result of catalyst deactivation, whereas in the presence of sufficient amounts of CuCl_2_, it is possible to utilize both boron atoms of B_2_cat_2_ for the generation of alkyl boronic ester products, thus mirroring the high atom economy previously limited to precious-metal-catalyzed borylations.^3^

**Figure 7:**
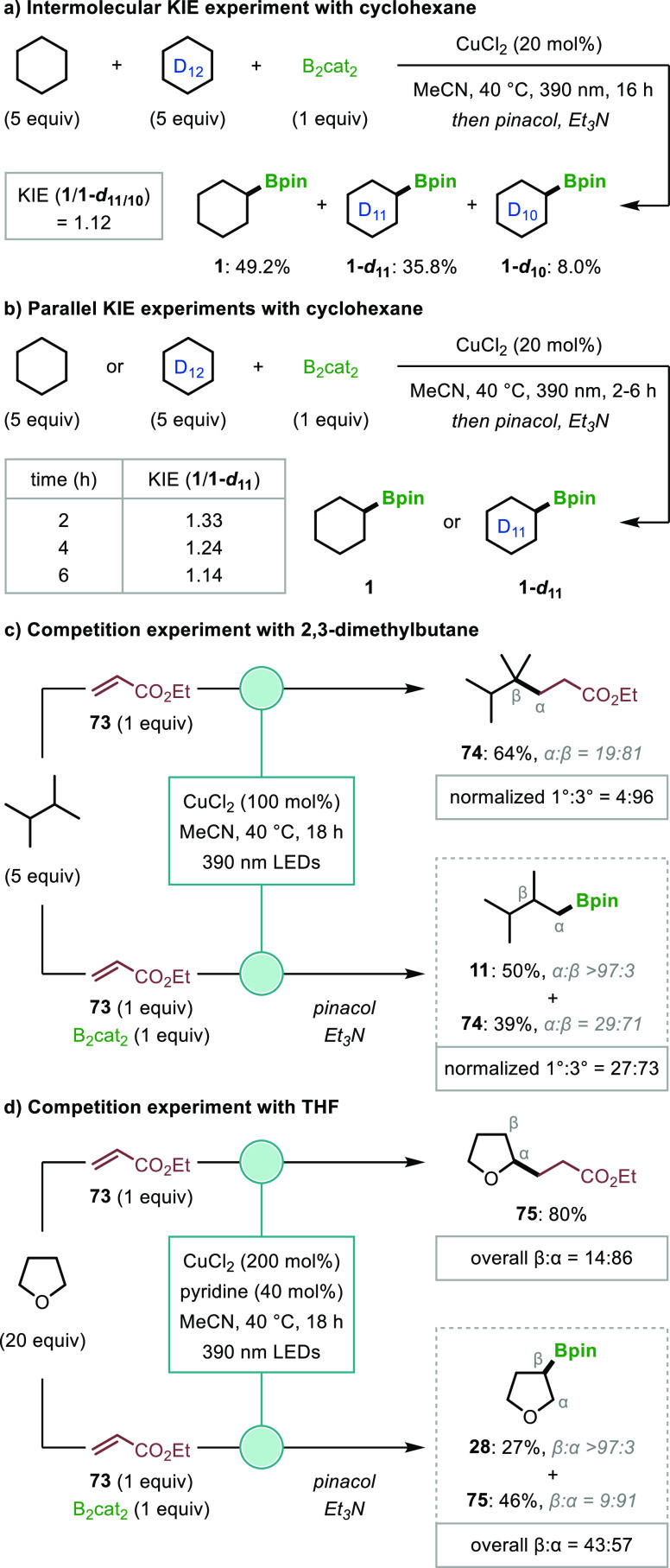
Mechanistic Investigation on regioselectivity. Yields were determined by GC-FID analysis.

To gain insight into the HAT process and the origin of the unusual regioselectivity, a series of competition experiments were performed. Kinetic isotope effect (KIE) studies with cyclohexane and cyclohexane-*d*_12_ revealed small KIE values in both intermolecular and parallel experiments (Figures 7a,b),^8,31^ which suggests that HAT occurs before the turnover-limiting step of the reaction.^32^ This was further supported by the formation of trace amounts of **1-*d*_10_** in the competition experiment, which confirms that HAT is reversible and occurs at a faster rate than borylation of the intermediate alkyl radicals. Similar results were also observed in an intermolecular KIE experiment with THF and THF-*d*_8_ (see Sections 3.9.1 and 3.9.2 of the Supporting Information).

Chlorine radical-mediated C–H functionalizations with electron-deficient alkenes have previously been shown to give high selectivity for reaction at tertiary and α-heteroatom positions;^8,18^ therefore, we performed borylations in the presence of ethyl acrylate (**73**) as a radical trap to intercept any tertiary or α-heteroatom radicals that do not undergo productive reactions with O(Bcat_2_). For 2,3-dimethylbutane, our borylation conditions provided boronic ester **11** in 77% yield with complete selectivity for reaction at primary C–H positions, whereas the alkylation with **73** gave carboxylate ester **74** in 81:19 r.r. in favor of the tertiary product (Figure 7c), which is consistent with the expected selectivity for HAT to “free” chloride radicals.^33^ The competition experiment with equimolar amounts of B_2_cat_2_ and **73** provided 50% yield of **11**, still with complete primary selectivity, and 39% of **74** with a slightly reduced tertiary selectivity of 71:29. The same competition experiment was performed with tetrahydrofuran (THF), generating boronic ester **28** as a single β-regioisomer and carboxylate ester **75** in 91:9 α-selectivity (Figure 7d). These results confirm that both tertiary and α-oxy radicals are generated; however, borylation of these more stabilized radicals with O(Bcat)_2_ is prevented by oxidative side-reactions (Pathway III, Figure 5). Competitive oxidation of alkyl radicals by CuCl_2_ is consistent with the expected thermodynamically favored SET between CuCl_2_ (*E*_1/2_[Cu^II^/Cu^I^] = +0.47 V vs SCE in MeCN)^34^ and tertiary alkyl or α-oxy radicals (*E*_1/2_[R^+^/R^·^] in V vs SCE in MeCN is +0.09 V for *t*-butyl and −0.35 for tetrahydrofuran-2-yl),^35^ whereas these radicals are known to undergo successful borylation in the absence of strong single-electron oxidants (see Section 3.7.4 of the Supporting Information for evidence of tertiary alkyl radical oxidation).^36^ Interestingly, the normalized tertiary:primary selectivity (based on the number of C–H bonds) in the reactions of 2,3-dimethylbutane decreased from 24:1 to 3:1 upon the addition of B_2_cat_2_, and this selectivity was found to be consistent throughout the course of the reaction (Table S26). A decrease in α:β selectivity from 6:1 to 1.3:1 was also observed when B_2_cat_2_ was added to reaction with THF. These changes in regioselectivity in the presence of catecholborate species provide support for the formation of radical “ate” complex **68**, which provides a sterically hindered chlorine radical that favors HAT from primary C–H bonds. In addition, the results of the reactions of THF indicate that **68** shows less preference for HAT of hydridic α-heteroatom C–H bonds relative to ‘free’ chlorine radicals. Therefore, the regioselectivities of HAT reactions to **68** appear to be strongly influenced by both the steric and electronic properties of C–H bonds. The above results highlight the important influence of both HAT and C–B bond formation on the observed regioselectivities.

## Conclusions

We have discovered a copper-catalyzed protocol that enables the C(sp^3^)–H borylation of non-activated alkanes under mild photochemical conditions and in the absence of terminal oxidants. This was possible due to an unexpected CuCl_2_-catalyzed dehydrogenative reaction between B_2_cat_2_ and H_2_O to generate O(Bcat)_2_, which was found to be an efficient borylating agent for alkyl radicals. Crucially, this initial oxidation converts the nucleophilic diboron reagent into an electrophilic bis-boryloxide, therefore making the subsequent photoinduced LMCT-mediated C(sp^3^)–H borylation a redox-neutral process. These conditions allowed the borylation of a broad range of substates, displayed excellent functional group tolerance, and provided alkyl boronic ester products in high regioselectivities. Although many substrates were found to require stoichiometric CuCl_2_ for optimal yields due to competing reduction of Cu(II) to Cu(I), efficient catalysis was possible with some cyclic alkanes. Notably, the high atom economy of this radical-mediated borylation has previously only been demonstrated with precious-metal catalysts, thus demonstrating the power of photoinduced tandem copper catalysis.^37^

## Supporting Information Available

Additional experimental details, including procedures, photographs of the experimental setup, characterization data, spectra, and computational details (PDF)
