# Supplementary material for: Copper-Mediated Dehydrogenative C(sp3)–H Borylation of Alkanes
Source: J Am Chem Soc. 2023 Jul 6;145(28):15207–17. doi: 10.1021/jacs.3c02185 (PMC10360158; doi:10.1021/jacs.3c02185)
Supplement: Supplementary file 1 — ja3c02185_si_001.pdf [file ja3c02185_si_001.pdf]

SUPPORTING INFORMATION

Copper-Mediated Dehydrogenative C(sp^3)–H borylation of alkanes

Ruocheng Sang, Wangyujing Han, Hanwen Zhang, Carla M. Saunders, Adam Noble* and Varinder K. Aggarwal*

School of Chemistry, University of Bristol, Cantock's Close, Bristol BS8 1TS, United Kingdom

*e-mail: a.noble@bristol.ac.uk; v.aggarwal@bristol.ac.uk

TABLE OF CONTENTS

LIST OF SUPPLEMENTARY SCHEMES, FIGURES AND TABLES 3

LIST OF CHARACTERISED PRODUCTS 4

1. MATERIALS AND GENERAL METHODS 7

1.1. Glassware, Solvents and Reagents 7

1.2. Chromatography and Instrumentation 7

1.3. Naming of Compounds 8

2. EXPERIMENTAL DATA 9

2.1. General Procedures 9

2.1.1. General Procedure A: Standard procedure with $CuCl_2$ (0.2 equiv.) 9

2.1.2. General Procedure A-Prestirring: Standard procedure with $CuCl_2$ (0.2 equiv.) 9

2.1.3. General Procedure B: Standard procedure with $CuCl_2$ (1.0 equiv.) 10

2.1.4. General Procedure B-Prestirring: Standard procedure with $CuCl_2$ (1.0 equiv.) 10

2.1.5. General Procedure C: Standard procedure with $CuCl_2$ and pyridine 11

2.2. Photochemical Equipment and Setup 12

2.2.1. Photochemical equipment and setup for General Procedures A and B 12

2.2.2. Photochemical equipment and setup for General Procedure C 12

2.3. Reaction Optimisation 13

2.3.1. Standard procedure for reaction optimisation 13

2.3.2. Catalyst screening 13

2.3.3. Solvent screening 14

2.3.4. Effect of alkane equivalents and reaction concentration 14

2.3.5. Effect of $CuCl_2$ loading 15

2.3.6. Effect of H_2O concentration 15

2.3.7. Control experiments 16

2.3.8. Reaction optimisation with 1 equivalent of cyclohexane 16

2.4. Synthesis of Alkane Substrates 17

2.5. Substrate Scope 30

2.6. Scale-up Reaction 81

2.7. Alkane Stoichiometry Studies 82

2.8. Selectivity Comparison 86

2.9. Comparison with Metal-free Method 87

2.10. Unsuccessful Substrates 88

2.11. Reaction Cost Comparison 89

2.12. Comparison between General Procedures B and C for Borylations of Cyclic Ethers 89

3. MECHANISTIC STUDIES 90

3.1. Studies into the $CuCl_2$ Loading 90

3.1.1. Effect of pre-stirring with different $CuCl_2$ loadings 90

3.1.2. Effect of $CuCl_2$ loading on regioselectivity of adamantane 91
3.1.3. Effect of $CuCl_2$ loading on regioselectivity of 2,5-dimethylhexane 92
3.1.4. Effect of $CuCl_2$ loading on regioselectivity of pentane 93
3.2. Investigations into the Reaction of $CuCl_2$ with B_2cat_2 94
3.2.1. NMR studies of $CuCl_2$ and B_2cat_2 in CD_3CN 94
3.2.2. H_2 gas detection 96
3.3. Investigations into the Use of ClBcat as the Borylating Agent 97
3.3.1. NMR spectra of ClBcat in CD_3CN 97
3.3.2. Investigation into the reactivity of ClBcat in the borylation of cyclohexane 99
3.3.3. NMR studies into the borylation reaction with ClBcat 100
3.4. Investigations into the Formation and Reactivity of $O(Bcat)_2$ 102
3.4.1. Independent synthesis of HOBcat 102
3.4.2. Independent synthesis of $O(Bcat)_2$ 102
3.4.3. NMR comparison of $O(Bcat)_2$ 103
3.4.4. Investigation into the reactivity of independently synthesised $O(Bcat)_2$ 104
3.4.5. Reaction monitoring of the $O(Bcat)_2$ formation 105
3.4.6. Investigation into the effects of water concentration on $O(Bcat)_2$ formation 106
3.5. NMR Studies of the Borylation Reaction 108
3.6. Effect of Portionwise Addition of $CuCl_2$ 110
3.7. Competition Experiments 113
3.7.1. Competition experiment with 2,3-dimethylbutane 113
3.7.2. Competition experiment with THF 115
3.7.3. Reaction monitoring of the competition reaction with 2,3-dimethylbutane. 117
3.7.4. Evidence for oxidation of tertiary alkyl radical intermediates 119
3.7.5. Evidence for oxidation of benzylic radical intermediates 121
3.8. EPR Studies 122
3.9. Kinetic Isotope Effect Experiments 125
3.9.1. Determination of the KIE for the borylation of THF 125
3.9.2. Determination of the KIE for the alkylation of THF with ethyl acrylate 127
3.9.3. Determination of the KIE for the borylation of cyclohexane 129
3.9.4. Parallel KIE study for the borylation of cyclohexane 131
3.10. Further Discussion of the Proposed Mechanism 132
3.10.1. Mechanistic proposals for $O(Bcat)_2$ formation 132
3.10.2. Mechanistic proposals for the observed regioselectivities 133
3.10.3. Mechanistic proposals for Cu(II) consumption 137
3.10.4. Mechanistic proposals for byproduct formation 138
4. DFT CALCULATIONS 139
4.1. General Notes 139
4.2. Borylation with B_2cat_2 139
4.3. Borylation with $OB(cat)_2$ 140
4.4. Borylation with $OB(cat)_2$ in the Presence of CuCl 141

4.5. Discussion of Computational Results 142
4.6. XYZ Coordinates 143
5. NMR SPECTROSCOPIC DATA 156
6. GC-FID DATA 295
7. REFERENCES 310

LIST OF SUPPLEMENTARY SCHEMES, FIGURES AND TABLES

Table S1. Catalyst screening 13
Table S2. Solvent screening 14
Table S3. Effects of alkane equivalents and reaction concentration 14
Table S4. Solvent screening 15
Table S5. Effect of H_2O concentration 15
Table S6. Control experiments 16
Table S7. Reaction optimisation with 1 equivalent of cyclohexane 16
Table S8. Effect of alkane stoichiometry: Boronic ester **1** 82
Table S9. Effect of alkane stoichiometry: Boronic ester **5** 82
Table S10. Effect of alkane stoichiometry: Boronic ester **19** 83
Table S11. Effect of alkane stoichiometry: Boronic ester **23** 83
Table S12. Effect of alkane stoichiometry: Boronic ester **31** 83
Table S13. Effect of alkane stoichiometry: Boronic ester **57** 84
Table S14. Effect of alkane stoichiometry: Boronic ester **58** 84
Table S15. Effect of alkane stoichiometry: Boronic ester 62 85
Figure S1. Selectivity comparison 86
Figure S2. Comparison with metal-free method 87
Figure S3. Unsuccessful substrates 88
Table S16. Reaction cost comparison 89
Figure S4. Comparison between General Produce B and C Using Cyclic Ether 89
Figure S5. Effects of pre-stirring using 0.2–1 equiv. $CuCl_2$ 90
Table S17. Effect of $CuCl_2$ on regioselectivity in the borylation of adamantane 91
Table S18. Effect of $CuCl_2$ on regioselectivity in the borylation of 2,5-dimethylhexane 92
Table S19. Effect of $CuCl_2$ on regioselectivity in the borylation of pentane 93
Figure S6. ^1H NMR spectra of B_2cat_2 and $CuCl_2$ in CD_3CN 94
Figure S7. ^{11}B NMR spectra of B_2cat_2 and $CuCl_2$ in CD_3CN 95
Figure S8. ^{13}C NMR spectra of B_2cat_2 and $CuCl_2$ in CD_3CN after 16 h 95
Figure S9. Detection of H_2 gas by ^1H NMR 96
Figure S10. ^1H NMR spectra of ClBcat in CD_3CN 97
Figure S11. ^{11}B NMR spectra of ClBcat in CD_3CN 98
Table S20. Reaction optimization using ClBcat as borylating reagent 99
Figure S12. ^{11}B NMR spectra of the reaction with ClBcat 100
Figure S13. ^1H NMR spectra of the reaction with ClBcat after 16 h irradiation 101
Figure S14. ^{13}C NMR spectra of the reaction with ClBcat after 16 h irradiation 101
Table S21. ^1H NMR comparison for synthetic and in situ formed $O(Bcat)_2$ 103

Table S22. ^{13}C NMR comparison synthetic and in situ formed $O(Bcat)_2$ ... 103
Figure S15. Reaction monitoring of the $O(Bcat)_2$ formation ... 105
Table S23. Effects of water (1 equiv.) on $O(Bcat)_2$ formation ... 106
Table S24. Effects of water (5 equiv.) on $O(Bcat)_2$ formation ... 106
Table S25. Control experiment on B–B bond cleavage ... 107
Figure S16. ^{11}B NMR spectra of the borylation reaction in CD_3CN ... 108
Figure S17. ^{1}H NMR spectra of the borylation reaction in CD_3CN after 16 h irradiation ... 109
Figure S18. ^{13}C NMR spectra of the borylation reaction in CD_3CN after 16 h irradiation ... 109
Figure S19. ^{11}B NMR spectra of the borylation reaction with additional $CuCl_2$ ... 111
Table S26. Time study for the competition reaction with 2,3-dimethylbutane ... 117
Figure S20. Observation of alkene side-products derived from tertiary alkyl radicals ... 119
Figure S21. X-band EPR measurements recorded at 25 °C ... 123
Figure S22. HR-GCMS trace for the KIE experiments with THF ... 125
Figure S23. HR-GCMS quantification for the KIE experiments with THF ... 126
Figure S24. HR-GCMS trace for the recovery of the starting material ... 126
Figure S25. HR-GCMS trace for the KIE experiments with THF and ethyl acrylate ... 128
Figure S26. HR-GCMS quantification for the KIE experiments with THF and ethyl acrylate ... 128
Figure S27. HR-GCMS data for the KIE experiments with cyclohexane ... 130
Figure S28. HR-GCMS quantification for the KIE experiments with cyclohexane ... 130
Table S27. Parallel KIE study for the borylation of cyclohexane ... 131
Figure S29. Possible HAT species ... 133
Figure S30. Proposed pathways for the borylation and alkylation of 2,3-dimethylbutane ... 134
Figure S31. Proposed pathway for formation of the HAT species ... 136
Figure S32. Competing pathways for the reaction of primary and tertiary radicals ... 136
Figure S33. Possible pathways for the consumption of $CuCl_2$ ... 137
Figure S34. Calculated free energies for the borylation of an ethyl radical with B_2cat_2 ... 139
Figure S35. Calculated free energies for the borylation of an ethyl radical with $O(Bcat)_2$ ... 140
Figure S36. Calculated free energies for the borylation of an ethyl radical with $O(Bcat)_2$ and CuCl ... 141

LIST OF CHARACTERISED PRODUCTS

4-Methylpentyl furan-2-carboxylate (**39-S**) ... 17
4-Methylpentyl thiophene-3-carboxylate (**40-S**) ... 17
2-Isopentylisoindoline-1,3-dione (**43-S**) ... 18
1,1,1-Trifluoro-N-isopentylmethanesulfonamide (**46-S**) ... 19
N-Phth *L*-leucine methyl ester (**52-S**) ... 19
N-Phth *L*-*tert*-leucine methyl ester (**53-S**) ... 20
Dimethoxy bisphenol A (**55-S**) ... 21
Clofibric ester derivative (**56-S**) ... 21
Triclosan derivative (**57-S**) ... 22
Probenecid methyl ester (**58-S**) ... 23
D-Galactopyranose ester derivative (**59-S**) ... 23
Flurbiprofen derivative (**60-S**) ... 24
Saccharin derivative (**61-S**) ... 25
Naproxen derivative (**62-S**) ... 25
Oxaprozin ester derivative (**63-S**) ... 26
Lorzone derivative (**64-S**) ... 27

Ciprofibrate ester derivative (**65-S**) 27
Celecoxib derivative (**67-S**) 28
2-Cyclohexyl-4,4,5,5-tetramethyl-1,3,2-dioxaborolane (**1**) 30
2-Cyclopentyl-4,4,5,5-tetramethyl-1,3,2-dioxaborolane (**2**) 30
2-Cycloheptyl-4,4,5,5-tetramethyl-1,3,2-dioxaborolane (**3**) 31
2-Cyclododecyl-4,4,5,5-tetramethyl-1,3,2-dioxaborolane (**4**) 31
2-((1*R**,2*R**,4*R**)-Bicyclo[2.2.1]heptan-2-yl)-4,4,5,5-tetramethyl-1,3,2-dioxaborolane (**5**) 32
(2*R**,4a*R**,8a*R**)-Decahydronaphthalen-2-ol (**6-ox**) 33
(1*R**,2*S**,4*R**)-5-Chlorobicyclo[2.2.1]heptan-2-ol (**7-ox**) 34
(1*R**,2*R**,4*S**,7*R**)-7-Bromo-bicyclo[2.2.1]heptan-2-ol (**8-ox-a**) and (1*S**,2*R**,4*R**,7*R**)-7-bromo-bicyclo[2.2.1]heptan-2-ol (**8-ox-b**) 35
2-((1*R*,3*S*,5*r*,7*r*)-Adamantan-2-yl)-4,4,5,5-tetramethyl-1,3,2-dioxaborolane (**9a**) and 2-((3*r*,5*r*,7*r*)-adaman-tan-1-yl)-4,4,5,5-tetramethyl-1,3,2-dioxaborolane (**9b**) 36
4,4,5,5-Tetramethyl-2-pentyl-1,3,2-dioxaborolane (**10a**), 4,4,5,5-tetramethyl-2-(pentan-2-yl)-1,3,2-dioxa-borolane (**10b**), and 4,4,5,5-tetramethyl-2-(pentan-3-yl)-1,3,2-dioxaborolane (**10c**) 37
2-(2,3-Dimethylbutyl)-4,4,5,5-tetramethyl-1,3,2-dioxaborolane (**11**) 38
2-(2,4-Dimethylpentyl)-4,4,5,5-tetramethyl-1,3,2-dioxaborolane (**12**) 38
4,4,5,5-Tetramethyl-2-(2,4,4-trimethylpentyl)-1,3,2-dioxaborolane (**13a**) and 4,4,5,5-tetramethyl-2-(2,2,4-trimethylpentyl)-1,3,2-dioxaborolane (**13b**) 39
4,4,5,5-Tetramethyl-2-(2,2,4,4-tetramethylpentyl)-1,3,2-dioxaborolane (**14**) 40
2-(3-Chloro-2,2-dimethylpropyl)-4,4,5,5-tetramethyl-1,3,2-dioxaborolane (**15**) 40
2-(3-Bromo-2,2-dimethylpropyl)-4,4,5,5-tetramethyl-1,3,2-dioxaborolane (**16**) 41
2-(4-Chloro-2-methylbutyl)-4,4,5,5-tetramethyl-1,3,2-dioxaborolane (**17**) 41
2-(4-Bromo-2-methylbutyl)-4,4,5,5-tetramethyl-1,3,2-dioxaborolane (**18**) 42
2-(5-Bromo-2-methylpentyl)-4,4,5,5-tetramethyl-1,3,2-dioxaborolane (**19**) 43
2-(4-Bromobutyl)-4,4,5,5-tetramethyl-1,3,2-dioxaborolane (**20a**) and 2-(4-bromobutan-2-yl)-4,4,5,5-tetra-methyl-1,3,2-dioxaborolane (**20b**) 43
4,4,5,5-Tetramethyl-2-(6,6,6-trifluorohexyl)-1,3,2-dioxaborolane (**21a**) and 4,4,5,5-tetramethyl-2-(6,6,6-trifluorohexan-2-yl)-1,3,2-dioxaborolane (**21b**) 44
2-(2,2-Dimethylpent-4-en-1-yl)-4,4,5,5-tetramethyl-1,3,2-dioxaborolane (**22**) 45
2-(2-(3,5-Di-*tert*-butylphenyl)-2-methylpropyl)-4,4,5,5-tetramethyl-1,3,2-dioxaborolane (**23**) 46
2-(2-(3,5-Dibromophenyl)-2-methylpropyl)-4,4,5,5-tetramethyl-1,3,2-dioxaborolane (**24**) 47
4,4,5,5-Tetramethyl-2-((1,4,4-trimethyl-1,2,3,4-tetrahydronaphthalen-1-yl)methyl)-1,3,2-dioxaborolane (**25**) 47
2-(2-(3,5-Diisopropylphenyl)propyl)-4,4,5,5-tetramethyl-1,3,2-dioxaborolane (**26**) 48
4-(1-(4,4,5,5-Tetramethyl-1,3,2-dioxaborolan-2-yl)propan-2-yl)benzonitrile (**27**) 49
4,4,5,5-Tetramethyl-2-(tetrahydrofuran-2-yl)-1,3,2-dioxaborolane (**28**) 49
4,4,5,5-Tetramethyl-2-(tetrahydro-2H-pyran-4-yl)-1,3,2-dioxaborolane (**29a**) and 4,4,5,5-tetramethyl-2-(tetra-hydro-2H-pyran-3-yl)-1,3,2-dioxaborolane (**29b**) 50
4,4,5,5-Tetramethyl-2-((tetrahydro-2H-pyran-4-yl)methyl)-1,3,2-dioxaborolane (**30**) 51
2-((1*S*,2*R*,4*R*)-7-Oxabicyclo[2.2.1]heptan-2-yl)-4,4,5,5-tetramethyl-1,3,2-dioxaborolane (**31**) 51
2-(4-(Isopentyloxy)-2-methylbutyl)-4,4,5,5-tetramethyl-1,3,2-dioxaborolane (**32**) 52
2-(4-Butoxybutan-2-yl)-4,4,5,5-tetramethyl-1,3,2-dioxaborolane (**33a**) and 2-(4-butoxybutyl)-4,4,5,5-tetra-methyl-1,3,2-dioxaborolane (**33b**) 53
3-(4,4,5,5-Tetramethyl-1,3,2-dioxaborolan-2-yl)cyclohexan-1-one (**34a**) and 4-(4,4,5,5-tetramethyl-1,3,2-dioxaborolan-2-yl)cyclohexan-1-one (**34b**) 54
3-Methyl-1-phenyl-4-(4,4,5,5-tetramethyl-1,3,2-dioxaborolan-2-yl)butan-1-one (**35**) 55
2-Methyl-3-(4,4,5,5-tetramethyl-1,3,2-dioxaborolan-2-yl)propyl acetate (**36**) 55
3-Methyl-4-(4,4,5,5-tetramethyl-1,3,2-dioxaborolan-2-yl)butyl benzoate (**37**) 56
4-(4,4,5,5-Tetramethyl-1,3,2-dioxaborolan-2-yl)butyl benzoate (**38a**) and 3-(4,4,5,5-tetramethyl-1,3,2-dioxaborolan-2-yl)butyl benzoate (**38b**) 57

4-Methyl-5-(4,4,5,5-tetramethyl-1,3,2-dioxaborolan-2-yl)pentyl furan-2-carboxylate (**39**) 58
4-Methyl-5-(4,4,5,5-tetramethyl-1,3,2-dioxaborolan-2-yl)pentyl thiophene-3-carboxylate (**40**)......................... 58
2,2,2-Trifluoro-1-(3-hydroxypiperidin-1-yl)ethan-1-one (**41a-ol**) and 2,2,2-trifluoro-1-(4-hydroxy-piperidin-1-yl)ethan-1-one (**41b-ol**).. 59
2,2,2-Trifluoro-N-(3-methyl-4-(4,4,5,5-tetramethyl-1,3,2-dioxaborolan-2-yl)butyl)acetamide (**42**).................... 60
2-(3-Methyl-4-(4,4,5,5-tetramethyl-1,3,2-dioxaborolan-2-yl)butyl)isoindoline-1,3-dione (**43**)........................... 61
2-(4-(4,4,5,5-Tetramethyl-1,3,2-dioxaborolan-2-yl)butyl)isoindoline-1,3-dione (**44**) 62
2-(4-Methyl-5-(4,4,5,5-tetramethyl-1,3,2-dioxaborolan-2-yl)pentyl)isoindoline-1,3-dione (**45**).......................... 62
1,1,1-Trifluoro-N-(3-methyl-4-(4,4,5,5-tetramethyl-1,3,2-dioxaborolan-2-yl)butyl)methanesulfonamide (**46**)... 63
4-(4,4,5,5-Tetramethyl-1,3,2-dioxaborolan-2-yl)butanenitrile (**47**) 64
4-Methyl-5-(4,4,5,5-tetramethyl-1,3,2-dioxaborolan-2-yl)pentanenitrile (**48**)... 64
2,2'-(2-Methylpropane-1,3-diyl)bis(4,4,5,5-tetramethyl-1,3,2-dioxaborolane) (**49**).. 65
Dimethyl(phenyl)((4,4,5,5-tetramethyl-1,3,2-dioxaborolan-2-yl)methyl)silane (**50**).. 66
Triethyl(2-(4,4,5,5-tetramethyl-1,3,2-dioxaborolan-2-yl)ethyl)silane (**51**) 66
N-Phth *L*-Leucine derivative pinacol boronic acid ester (**52**)... 67
N-Phth *L*-*tert*-Leucine derivative pinacol boronic acid ester (**53**) 68
Eucalyptol pinacol boronic acid ester (**54**)... 68
Dimethoxy bisphenol A boronic acid pinacol ester (**55**) 69
Clofibric derivative pinacol bronic ester derivative (**56**).. 70
Triclosan derivative pinacol boronic acid ester (**57**) 70
Probenecid methyl ester pinacol boronic acid ester (**58**) 71
D-Galactopyranose derivative pinacol boronic acid ester (**59**)... 72
Flurbiprofen derivative pinacol boronic acid ester (**60**).. 73
Saccharine derivative pinacol boronic acid ester (**61**)... 74
Naproxen derivative pinacol boronic acid ester (**62**) 74
Oxaprozin derivative pinacol bronic ester derivative (**63**).. 75
Lorzone derivative pinacol boronic acid ester (**64**).. 76
Ciprofibrate derivative pinacol bronic ester derivative (**65**) 77
Cinnamic acid derivative pinacol boronic acid ester (**66**) 77
Celecoxib derivative pinacol boronic acid ester (**67**)... 78
2-(2,5-dimethylhexyl)-4,4,5,5-tetramethyl-1,3,2-dioxaborolane (**77**) 79
2-(2-(4-(*tert*-Butyl)phenyl)-2-methylpropyl)-4,4,5,5-tetramethyl-1,3,2-dioxaborolane (**78**)................................ 79
2-(2-(3-(*tert*-Butyl)phenyl)-2-methylpropyl)-4,4,5,5-tetramethyl-1,3,2-dioxaborolane (**79**)................................ 80

1. MATERIALS AND GENERAL METHODS

1.1. Glassware, Solvents and Reagents

All anhydrous solvents were commercially supplied or dried using an Anhydrous Engineering alumina column drying system [THF, toluene, Et_2O, DCM (CH_2Cl_2)]. Reagents were purchased from commercial sources and used as received. Acetonitrile (99.9+%, 100 mL, Extra Dry, AcroSeal™, w/o molecular sieves) was purchased from ACROS and used as received inside a glovebox. The water content was 33 ppm based on the Karl-Fischer titration. Acetonitrile-d_3 (≥99.8 atom % D, 10 g) was purchased from Sigma-Aldrich and used as received inside a glovebox. The water content was 117 ppm based on the Karl-Fischer titration. Bis(catecholato)diboron (B_2cat_2) (CAS: 13826-27-2) was purchased from Fluorochem (95% purity, 100 g).

1.2. Chromatography and Instrumentation

Thin layer chromatography (TLC) was performed using Merck Kieselgel 60 F254 fluorescent treated silica, which was visualised under UV light, or by staining with aqueous basic potassium permanganate followed by heating, *p*-anisaldehyde solution followed by heating, Hanessian's stain (CAM stain) followed by heating, or an ethanolic solution of phosphomolybdic acid followed by heating, as stated.

Flash column chromatography (FCC) was carried out using Sigma-Aldrich silica gel (60 Å, 230–400 mesh, 40–63 µm) or a Biotage Isolera™ flash purification system. In cases where automated column chromatography was employed the solvent gradient and flow rate are indicated.

NMR spectra were recorded at various field strengths, as indicated, using Bruker 400 MHz, Varian VNMR 400 MHz, Varian VNMR 500 MHz, or Bruker Cryo 500 MHz for ^{1}H, ^{11}B, ^{13}C and ^{19}F acquisitions. All NMR spectra were recorded at 25°C unless otherwise stated. Chemical shifts (δ) are reported in parts per million (ppm) and referenced $CDCl_3$ (^{1}H: 7.26 ppm; ^{13}C: 77.0 ppm) or MeCN-d_3 (^{1}H: 1.94 ppm; ^{13}C: 1.32 ppm). Coupling constants (J) are given in Hertz (Hz) and refer to apparent multiplicities (s = singlet, d = doublet, t = triplet, q = quartet, quin = quintet, sex = hextet, h = heptet, m = multiplet, br = broad signal, dd = doublet of doublets, *etc*.). The ^{1}H NMR spectra are reported as follows: chemical shift (multiplicity, coupling constants, number of protons).

Gas chromatography spectra (GC) were recorded on an Agilent 7890 GC with a HP-5MS column (15 m × 0.25 mm, 0.25 µm film thickness) and a split/splitless injection port (ratio: 35:1, rate: 42 mL/min). Chromatographic conditions: initial temperature: 70 °C, hold time: 1 min; Temperature program: ramp to 300 °C at 40 °C/min, hold at 300 °C for 3 min, final time: 9.75 min.

High resolution mass spectra (HRMS) were recorded on a Bruker Daltonics MicrOTOF II by Electrospray Ionisation (ESI); a Thermo Scientific QExactive by Electron Ionisation (EI); a Thermo Scientific Orbitrap Elite by ESI or Atmospheric Pressure Chemical Ionisation (APCI); or a Bruker UltrafleXtreme by Matrix-assisted Laser Desorption/Ionisation (MALDI).

IR spectra were recorded neat as a thin film on a Perkin Elmer Spectrum One FT-IR. Selected absorption maxima (ν_{max}) are reported in wavenumbers (cm^{-1}).

1.3. Naming of Compounds

Compound names are those generated by ChemDraw Professional 20.0 software (PerkinElmer), following the IUPAC nomenclature.

2. EXPERIMENTAL DATA

2.1. General Procedures

2.1.1. General Procedure A: Standard procedure with $CuCl_2$ (0.2 equiv.)

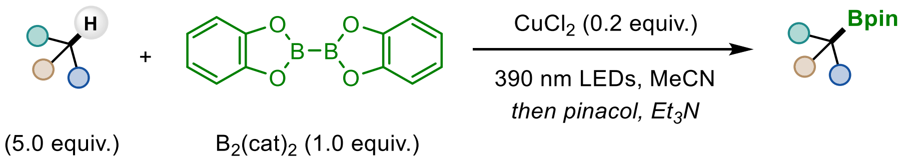

Under an ambient atmosphere, a flame dried 8 mL Biotage® microwave reaction vial equipped with a magnetic stir bar was charged with $CuCl_2$ (8 mg, 0.06 mmol, 0.2 equiv.) and B_2cat_2 (71 mg, 0.30 mmol, 1.0 equiv.). The vial was transferred into an anhydrous, argon-filled glovebox where anhydrous acetonitrile was added (1.5 mL, *c* = 0.20 M) followed by the alkane (1.5 mmol, 5.0 equiv.). The vial was sealed with a cap with septum, removed for the glovebox, and placed 4 cm away from two purple LEDs (Kessil PR160-390 nm LEDs). The reaction was stirred at a speed of 1200 rpm and irradiated for 16 h at 40 °C. After irradiation, while maintaining an argon atmosphere, a solution of pinacol (106 mg, 0.900 mmol, 3.00 equiv.) and Et_3N (0.84 mL, 6.0 mmol, 20 equiv.) in DCM (1 mL) was added and stirring was continued for 1 h. The reaction was concentrated *in vacuo* and purified by flash column chromatography or Biotage Isolera® flash purification on silica gel.

2.1.2. General Procedure A-Prestirring: Standard procedure with $CuCl_2$ (0.2 equiv.)

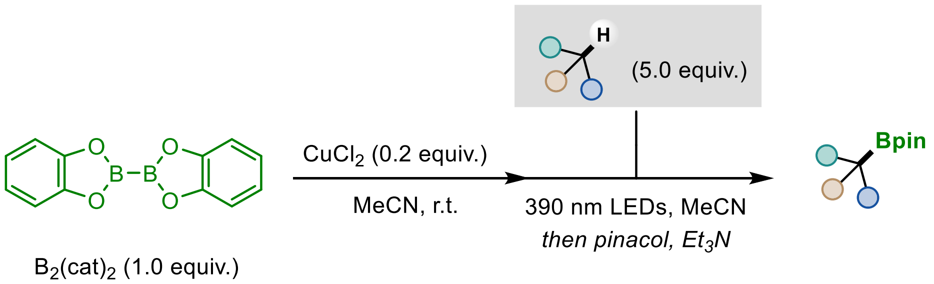

Under an ambient atmosphere, a flame dried 8 mL Biotage® microwave reaction vial equipped with a magnetic stir bar was charged with $CuCl_2$ (8 mg, 0.06 mmol, 0.2 equiv.) and B_2cat_2 (71 mg, 0.30 mmol, 1.0 equiv.). The vial was transferred into an anhydrous, argon-filled glovebox where anhydrous acetonitrile was added (1.5 mL, *c* = 0.20 M) and the vial was sealed by a Suba Seal septum. After stirring in the glovebox for 10 h, the alkane (1.5 mmol, 5.0 equiv.) was added. The vial was subsequently sealed with a cap with septum, removed from the glovebox, and placed 4 cm away from two purple LEDs (Kessil PR160-390 nm LEDs). The reaction was stirred at a speed of 1200 rpm and irradiated for 16 h at 40 °C. After irradiation, while maintaining an argon atmosphere, a solution of pinacol (106 mg, 0.900 mmol, 3.00 equiv.) and Et_3N (0.84 mL, 6.0 mmol, 20 equiv.) in DCM (1 mL) was added and stirring was continued for 1 h. The reaction was concentrated *in vacuo* and purified by flash column chromatography or Biotage Isolera® flash purification on silica gel.

2.1.3. General Procedure B: Standard procedure with $CuCl_2$ (1.0 equiv.)

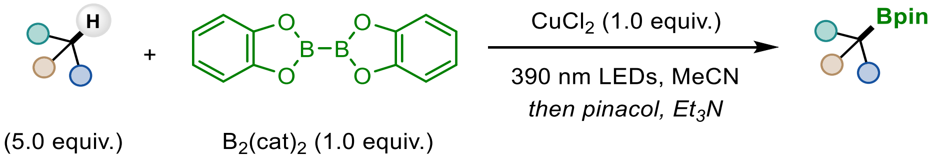

Under an ambient atmosphere, a flame dried 8 mL Biotage® microwave reaction vial equipped with a magnetic stir bar was charged with $CuCl_2$ (40 mg, 0.30 mmol, 1.0 equiv.) and B_2cat_2 (71 mg, 0.30 mmol, 1.0 equiv.). The vial was transferred into an anhydrous, argon-filled glovebox where anhydrous acetonitrile was added (1.5 mL, *c* = 0.20 M) followed by the alkane (1.5 mmol, 5.0 equiv.). The vial was sealed with a cap with septum, removed from the glovebox, and placed 4 cm away from two purple LEDs (Kessil PR160-390 nm LEDs). The reaction was stirred at a speed of 1200 rpm and irradiated for 16 h at 40 °C. After irradiation, while maintaining an argon atmosphere, a solution of pinacol (106 mg, 0.900 mmol, 3.00 equiv.) and Et_3N (0.84 mL, 6.0 mmol, 20 equiv.) in DCM (1 mL) was added and stirring was continued for 1 h. The reaction was concentrated *in vacuo* and purified by flash column chromatography or Biotage Isolera® flash purification on silica gel.

2.1.4. General Procedure B-Prestirring: Standard procedure with $CuCl_2$ (1.0 equiv.)

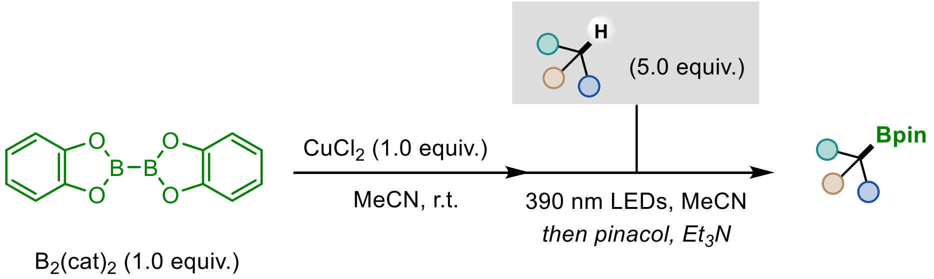

Under an ambient atmosphere, a flame dried 8 mL Biotage® microwave reaction vial equipped with a magnetic stir bar was charged with $CuCl_2$ (40 mg, 0.30 mmol, 1.0 equiv.) and B_2cat_2 (71 mg, 0.30 mmol, 1.0 equiv.). The vial was transferred into an anhydrous, argon-filled glovebox where anhydrous acetonitrile was added (1.5 mL, *c* = 0.20 M) and the vial was sealed by a Suba Seal septum. After stirring in the glovebox for 10 h, the alkane (1.5 mmol, 5.0 equiv.) was added. The vial was subsequently sealed with a cap with septum, removed from the glovebox, and placed 4 cm away from two purple LEDs (Kessil PR160-390 nm LEDs). The reaction was stirred at a speed of 1200 rpm and irradiated for 16 h at 40 °C. After irradiation, while maintaining an argon atmosphere, a solution of pinacol (106 mg, 0.900 mmol, 3.00 equiv.) and Et_3N (0.84 mL, 6.0 mmol, 20 equiv.) in DCM (1 mL) was added and stirring was continued for 1 h. The reaction was concentrated *in vacuo* and purified by flash column chromatography or Biotage Isolera® flash purification on silica gel.

2.1.5. General Procedure C: Standard procedure with $CuCl_2$ and pyridine

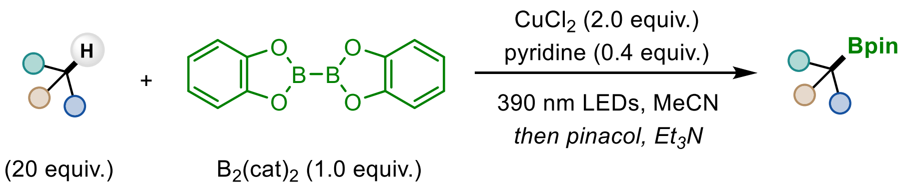

Under an ambient atmosphere, a flame dried 7 mL vial equipped with a magnetic stir bar was charged with $CuCl_2$ (80 mg, 0.60 mmol, 2.0 equiv.) and B_2cat_2 (71 mg, 0.30 mmol, 1.0 equiv.). The vial was transferred into an anhydrous, argon-filled glovebox where anhydrous acetonitrile was added (5.0 mL, c = 60 mM) followed by the alkane (6.0 mmol, 20 equiv.) and pyridine (9.5 mg, 9.7 µL, 0.12 mmol, 0.40 equiv.). The vial was subsequently sealed with a cap with septum, removed from the glovebox and placed in a PhotoCube® reactor where it was stirred at a speed of 1400 rpm and irradiated for 16 h at 35 °C. After irradiation while maintaining an argon atmosphere, a solution of pinacol (106 mg, 0.900 mmol, 3.00 equiv.) and Et_3N (0.84 mL, 6.0 mmol, 20 equiv.) in DCM (1 mL) was added and stirring was continued for 1 h. The reaction was concentrated *in vacuo* and purified by flash column chromatography or Biotage Isolera® flash purification on silica gel.

2.2. Photochemical Equipment and Setup

2.2.1. Photochemical equipment and setup for General Procedures A and B

The violet LED lamps were PR160L-390 nm Kessil LED lamps (λ_{max} = 390 nm) used with the intensity dial set to 100. The reaction vials were positioned approximately 4 cm from two LED lamps (see image below). During the photoinduced reactions, heat generated from the LED lamps resulted in warming of the reactions to approximately 40–45 ºC.

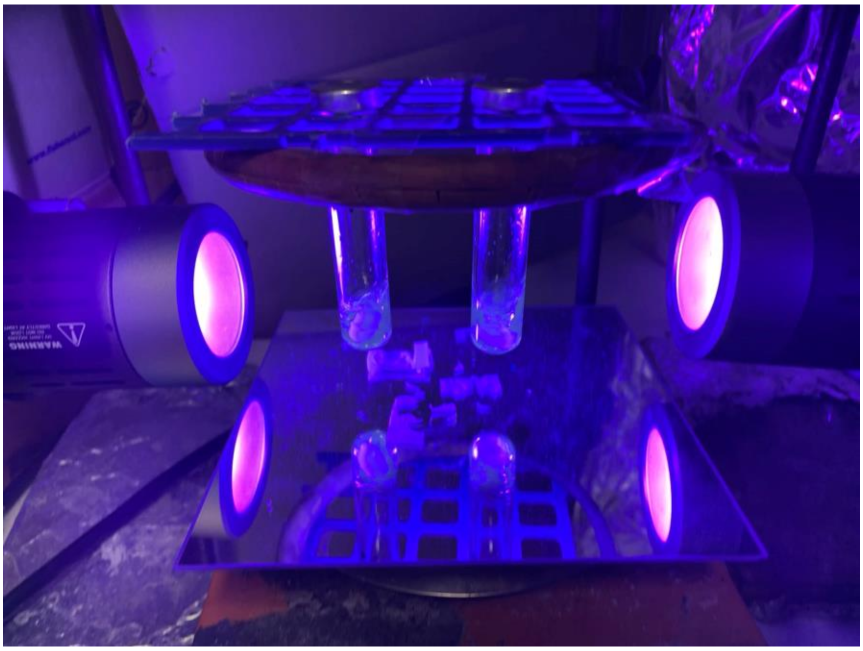

2.2.2. Photochemical equipment and setup for General Procedure C

The photochemical reactions were carried out in a PhotoCube® reactor manufactured by ThalesNano, using the violet LEDs (λ_{max} = 390 nm) set to 80% intensity, the LED panel selector set to 4, and the current selector set to Hi mode (see images below).

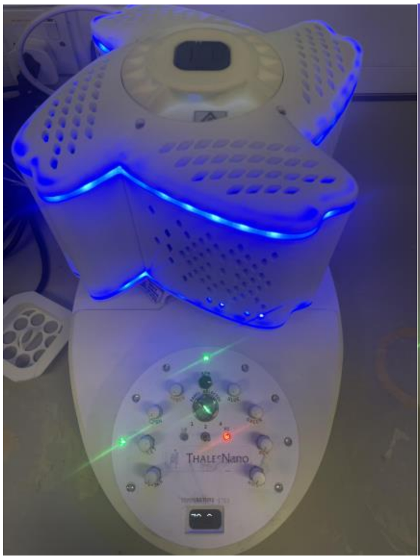

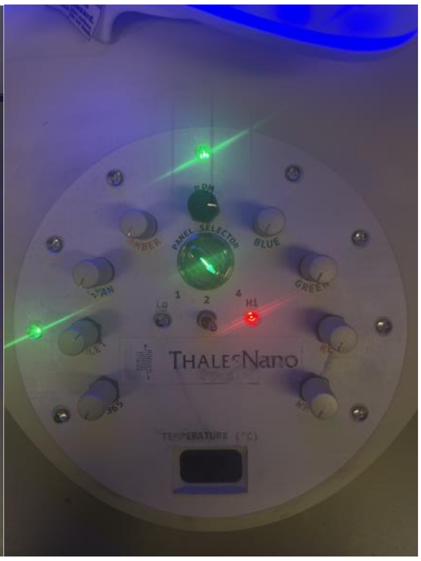

2.3. Reaction Optimisation

2.3.1. Standard procedure for reaction optimisation

Under an ambient atmosphere, a flame dried 8 mL Biotage® microwave reaction vial equipped with a magnetic stir bar was charged with catalyst and B_2cat_2 (71 mg, 0.30 mmol, 1.0 equiv.). The vial was transferred into an anhydrous, argon-filled glovebox where anhydrous solvent was added followed by cyclohexane (126 mg, 162 µL, 1.50 mmol, 5.00 equiv.). The vial was sealed with a cap with septum, removed from the glovebox, and placed 4 cm away from two purple LEDs (Kessil PR160-390 nm LEDs). The reaction was stirred at a speed of 1200 rpm and irradiated for 16 h at 40 °C. After irradiation, while maintaining an argon atmosphere, a solution of pinacol (106 mg, 0.900 mmol, 3.00 equiv.) and Et_3N (0.84 mL, 6.0 mmol, 20 equiv.) in DCM (1 mL) was added and stirring was continued for 1 h. 1,3,5-Trimethoxybenzene (50 mg, 0.30 mmol, 1.0 equiv.) as the internal standard was added. After vigorously shaking for 3 min, the crude reaction mixture was filtered through a short plug of silica and the yield was determined by GC-FID analysis.

2.3.2. Catalyst screening

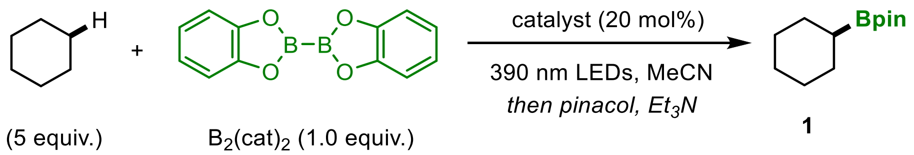

(5 equiv.) $B_2(cat)_2$ (1.0 equiv.) **1**

Entry	catalyst (20 mol%)	additive	GC-FID yield[a]
**1**	**$CuCl_2$**	**-**	**72% (64%)** [b]
2	$FeCl_3$	-	51%
3	$CoCl_2$	-	0
4	$NiCl_2$	-	0
5	$MgCl_2$	-	0
6	NaCl	-	0
7	$CuCl_2 \cdot 2H_2O$	-	68%
8	$CuBr_2$	-	0%
9	$Cu(OH)_2$	-	0%
10	$CuCl_2$	LiCl (0.5 equiv.)	64%
11	$Cu(OAc)_2$	LiCl (0.5 equiv.)	0
12	$Cu(BF_4)_2 \cdot xH_2O$	LiCl (0.5 equiv.)	28%

Table S1. Catalyst screening

[a] Yield determined by GC-FID analysis using 1,3,5-trimethoxybenzene as an internal standard. [b] Isolated yield.

2.3.3. Solvent screening

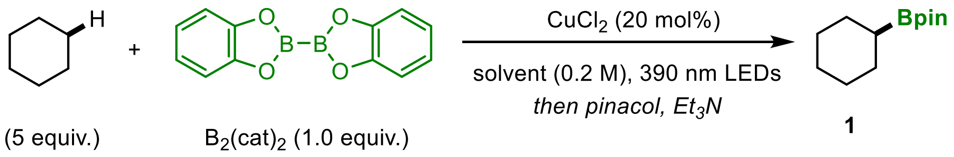

Entry	Solvent (0.2 M)	GC-FID yield[a]
**1**	**MeCN**	**72% (64%)[b]**
2	DMF	0
3	toluene	0
4	DCE	0
5	DCM	0
6	DMA	0
7	MeCN/DCE (10:1)	54%

Table S2. Solvent screening

[a] Yield determined by GC-FID analysis using 1,3,5-trimethoxybenzene as an internal standard. [b] Isolated yield.

2.3.4. Effect of alkane equivalents and reaction concentration

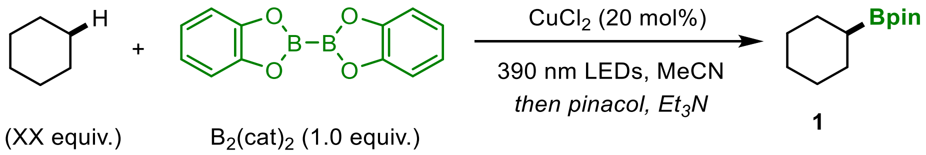

Entry	Alkane equivalents	Conc. of B_2cat_2	GC-FID yield[a]
**1**	**10 eq.**	**0.2 M**	**73%**
**2**	**5 eq.**	**0.2 M**	**72%**
3	3 eq.	0.2 M	52%
4	1 eq.	0.2 M	22%
5	5 eq.	0.5 M	70%
6	5 eq.	0.3 M	68%
7	5 eq.	0.1 M	61%

Table S3. Effects of alkane equivalents and reaction concentration

[a] Yield determined by GC-FID analysis using 1,3,5-trimethoxybenzene as an internal standard.

2.3.5. Effect of $CuCl_2$ loading

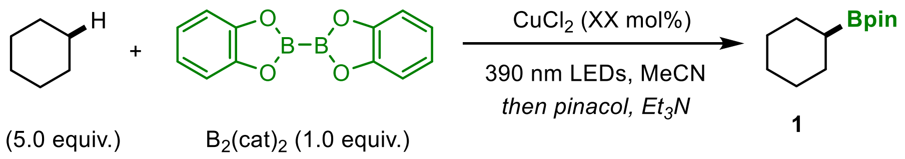

Entry	$CuCl_2$ loading	GC-FID yield[a]
1	10 mol%	66%
**2**	**20 mol%**	**72%**
3	40 mol%	70%
4	50 mol%	72%
5	100 mol%	74%
6	200 mol%	76%

Table S4. Solvent screening

[a] Yield determined by GC-FID analysis using 1,3,5-trimethoxybenzene as an internal standard.

2.3.6. Effect of H_2O concentration

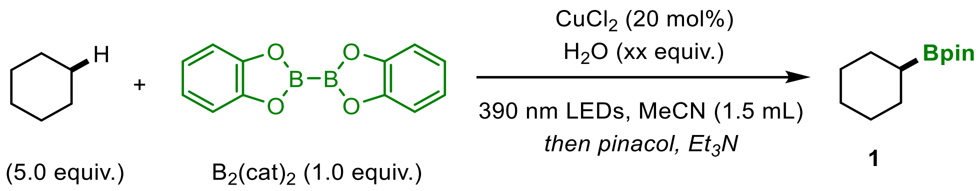

Entry[a]	added H_2O (mol%)	GC-FID yield (%)[b]	
		non-prestirring[d]	pre-stirring[e]
1	0	72	79
2[c]	0	74	*n.d.*
3	20	52	47
4	50	58	49
5	100	59	49
6	200	30	28
7	500	11	11

Table S5. Effect of H_2O concentration

[a] The water present in the solvents (1.5 mL) based on Karl-Fischer titrations amounts to 0.7 mol% for MeCN and 2.7 mol% for MeCN-d_3. [b] The yields were determined by GC-FID analysis using 1,3,5-trimethoxybenzene as an internal standard. The reported yields are the average of two duplicate reactions. [c] MeCN-d_3 (1.5 mL) as the solvent instead of MeCN. [d] Using General Procedure A. [e] Using General Procedure A-prestirring.

2.3.7. Control experiments

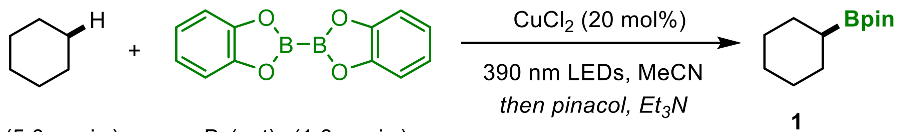

(5.0 equiv.) $B_2(cat)_2$ (1.0 equiv.) **1**

Entry	Changes from above	GC-FID yield[a]
**1**	**none**	**72%**
2	1 eq. B_2pin_2, no B_2cat_2	0
3	dark	0
4	no $CuCl_2$	0
5	white LEDs	<5%
6	blue LEDs	<5%
7	with prestirring (see Procedure A-prestirring)[b]	77%
**8**	**with prestirring (see *Procedure B-prestirring*)[c]**	**83%**
9	in PhotoCube (30 °C)	63%

Table S6. Control experiments

[a] Yield determined by GC-FID analysis using 1,3,5-trimethoxybenzene as an internal standard. [b] Following Procedure A-prestirring. [c] Following Procedure B-prestirring. The reported yields are the average from triplicate reactions.

2.3.8. Reaction optimisation with 1 equivalent of cyclohexane

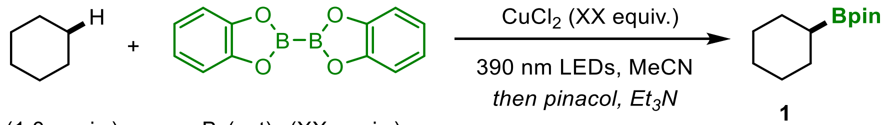

(1.0 equiv.) $B_2(cat)_2$ (XX equiv.) **1**

Entry	B_2cat_2 equivalents	$CuCl_2$ equivalents	GC-FID yield[a]
1	1	1	24%
2	1.2	1	27%
3	1.5	1	33%
4	2	1	42%
6	2	2	50%
7[b]	2	2	59% (47%)[c]
8	2.5	2.5	47%

Table S7. Reaction optimisation with 1 equivalent of cyclohexane

[a] Yield determined by GC-FID analysis using 1,3,5-trimethoxybenzene as an internal standard. [b] Cyclohexane was added after prestirring of $CuCl_2$ and B_2cat_2. [c] Isolated yield.

2.4. Synthesis of Alkane Substrates

4-Methylpentyl furan-2-carboxylate (39-S)

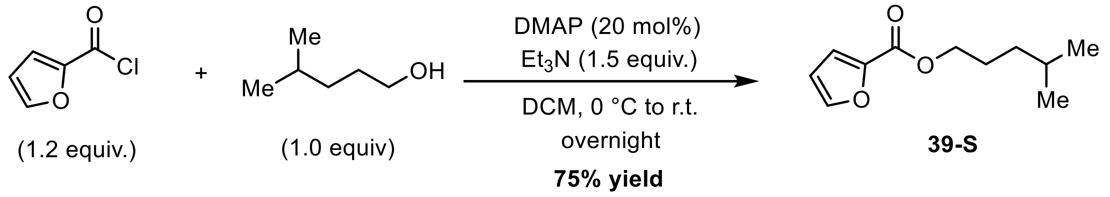

4-(Dimethylamino)pyridine (DMAP, 0.24 g, 2.0 mmol, 20 mol%) and Et_3N (1.5 g, 2.1 mL, 15 mmol, 1.5 equiv.) were added to a solution of 4-methylpentan-1-ol (0.88 g, 1.1 mL, 10 mmol, 1.0 equiv.) in dry DCM (40 mL, c = 0.25 M) at room temperature. Furan-2-carbonyl chloride (1.6 g, 1.2 mL, 12 mmol, 1.2 equiv.) was added dropwise at 0 °C. The resulting suspension was stirred at 0 °C for 1 h and then stirred at room temperature for 10 h. Subsequently, water was added slowly to quench the reaction at 0 °C. After allowing to warm to room temperature, the mixture was extracted with DCM twice. The combined organic layers were washed with brine, dried over $MgSO_4$, filtered, and concentrated *in vacuo.* The residue was purified by flash column chromatography, eluting with EtOAc/pentane (0 – 2%, v/v) to give **39-S** (1.5 g, 75%) as a colorless oil.

$\mathbf{R}_f$ = 0.80 (1:20 EtOAc/pentane, UV, CAM)

All recorded spectroscopic data matched those previously reported in the literature.[3]

NMR Spectroscopy (*see spectra*):

^{1}H NMR (400 MHz, $CDCl_3$) δ_H 7.56 (dd, J = 1.6, 0.8 Hz, 1H), 7.16 (dd, J = 3.6, 0.8 Hz, 1H), 6.49 (dd, J = 3.6, 1.6 Hz, 1H), 4.27 (t, J = 6.8 Hz, 2H), 1.80 – 1.68 (m, 2H), 1.58 (dq, J = 13.2, 6.8 Hz, 1H), 1.33 – 1.22 (m, 2H), 0.90 (d, J = 6.8 Hz, 6H) ppm;

^{13}C NMR (101 MHz, $CDCl_3$) δ_C 159.0, 146.3, 145.0, 117.8, 111.9, 65.5, 35.1, 27.8, 26.7, 22.6 ppm.

4-Methylpentyl thiophene-3-carboxylate (40-S)

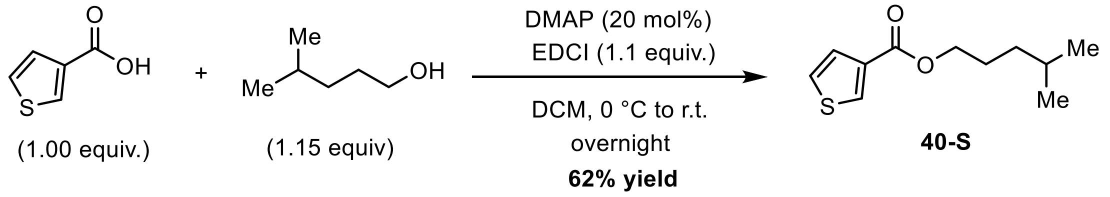

Thiophene-3-carboxylic acid (1.28 g, 10.0 mmol, 1.00 equiv.) and 4-(dimethylamino)pyridine (DMAP, 0.24 g, 2.0 mmol, 20 mol%) were added to a solution of 4-methylpentan-1-ol (1.17 g, 10.0 mmol, 1.15 equiv.) in dry DCM (25 mL, c = 0.4 M) at room temperature. 1-Ethyl-3-(3'-dimethylaminopropyl)carbodiimide hydrochloride (EDCI, 1.7 g, 11 mmol, 1.1 equiv.) was added in portions at 0 °C. The resulting suspension was stirred at 0 °C for 2 h and then stirred at room temperature for 10 h. Subsequently, the solvent was removed *in vacuo* and a mixture of EtOAc/H_2O (v/v = 3:1) was added. The layers were separated, and the aqueous layer was extracted with EtOAc twice. The combined organic layers were washed with saturated aqueous $NaHCO_3$ solution, dried

over $MgSO_4$, filtered, and concentrated *in vacuo*. The residue was purified by flash column chromatography, eluting with EtOAc/pentane (0 – 2%, v/v) to give **40-S** (1.3 g, 62%) as a colorless oil.

R*f* = 0.90 (1:20 EtOAc/pentane, UV, CAM)

NMR Spectroscopy (*see spectra*):

^{1}**H NMR** (400 MHz, $CDCl_3$) δ_H 8.10 (dd, *J* = 3.2, 1.2 Hz, 1H), 7.53 (dd, *J* = 5.2, 1.2 Hz, 1H), 7.30 (dd, *J* = 5.2, 3.2 Hz, 1H), 4.25 (t, *J* = 6.8 Hz, 2H), 1.79 – 1.68 (m, 2H), 1.66 – 1.54 (m, 1H), 1.35 – 1.26 (m, 2H), 0.91 (d, *J* = 6.8 Hz, 6H) ppm;

^{13}**C NMR** (101 MHz, $CDCl_3$) δ_C 163.0, 134.2, 132.6, 128.1, 126.0, 65.3, 35.2, 27.9, 26.8, 22.7 ppm.

All recorded spectroscopic data matched those previously reported in the literature.[3]

2-Isopentylisoindoline-1,3-dione (43-S)

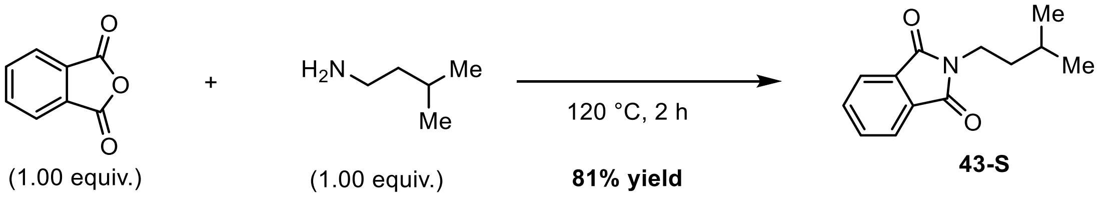

(1.00 equiv.) (1.00 equiv.) **81% yield** **43-S**

Prepared following a literature procedure[1]. 3-Methylbutan-1-amine (593 mg, 6.80 mmol, 1.00 equiv.), and phthalic anhydride (1.0 g, 6.8 mmol, 1.0 equiv.) were added in a sealed microwave vial and stirred at 120 °C for 2 h. After that, the reaction was cooled to room temperature, and diluted with EtOAc (20 mL) and then washed with water (2 × 10 mL). The layers were separated, and the aqueous layer was extracted with EtOAc (2 × 20 mL). The combined organic layers were dried over $MgSO_4$, filtered, and concentrated *in vacuo*. The residue was purified by flash column chromatography, eluting with EtOAc/petroleum ether (0 – 14%, v/v) to give **43-S** (1.2 g, 81% yield) as a colorless oil.

R*f* = 0.73 (1:6 EtOAc/petroleum ether, UV, $KMnO_4$)

NMR Spectroscopy (*see spectra*):

^{1}**H NMR** (400 MHz, $CDCl_3$) δ_H 7.82 (dd, *J* = 5.6, 3.2 Hz, 2H), 7.69 (dd, *J* = 5.6, 3.2 Hz, 2H), 3.72 – 3.65 (m, 2H), 1.65 – 1.58 (m, 1H), 1.58 – 1.52 (m, 2H), 0.95 (d, *J* = 6.4 Hz, 6H) ppm;

^{13}**C NMR** (101 MHz, $CDCl_3$) δ_C 168.6, 133.9, 132.4, 123.2, 37.5, 36.6, 26.0, 22.5 ppm.

All recorded spectroscopic data matched those previously reported in the literature.[1]

1,1,1-Trifluoro-N-isopentylmethanesulfonamide (46-S)

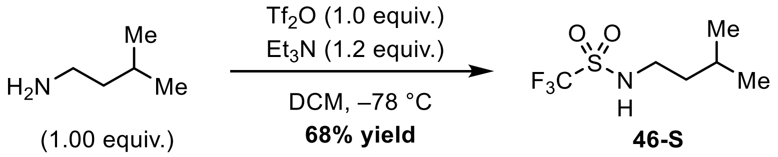

To a solution of isopentylamine (1.74 g, 2.31 mL, 20.0 mmol, 1.00 equiv.) in DCM (40 mL, *c* = 0.50 M) was added Et_3N (2.4 g, 3.3 mL, 24 mmol, 1.2 equiv.) at –78 °C. After stirring for 5 min at –78 °C, trifluoromethanesulfonic anhydride (3.3 mL, 20 mmol, 1.0 equiv.) was added dropwise and then the mixture was stirred for 1 h at –78 °C. After that, the reaction was quenched with saturated aqueous $NaHCO_3$ solution (40 mL) at 0 °C. The layers were separated, and the aqueous layer was extracted with DCM twice. The combined organic layers were dried over $MgSO_4$, filtered, and concentrated *in vacuo*. Biotage Isolera^{TM} flash purification on silica gel (SNAP 25 g silica cartridge), eluting with Et_2O/pentane (0 – 20%, v/v) to give **46-S** (3.0 g, 68%) as a colorless oil.

R*f* = 0.24 (1:20 EtOAc/pentane, CAM)

NMR Spectroscopy (*see spectra*):

^{1}**H NMR** (400 MHz, $CDCl_3$): δ_H 4.97 (brs, 1H), 3.34 – 3.27 (m, 2H), 1.66 (dt, *J* = 13.2, 6.8 Hz, 1H), 1.54 – 1.44 (m, 2H), 0.93 (d, *J* = 6.8 Hz, 6H) ppm;

^{13}**C NMR** (101 MHz, $CDCl_3$): δ_C 119.8 (q, *J* = 322.1 Hz), 43.0, 39.1, 25.4, 22.3 ppm;

^{19}**F NMR** (377 MHz, $CDCl_3$): δ_F –77.4 (s, 3F) ppm.

All recorded spectroscopic data matched those previously reported in the literature.[2]

***N*-Phth *L*-leucine methyl ester (52-S)**

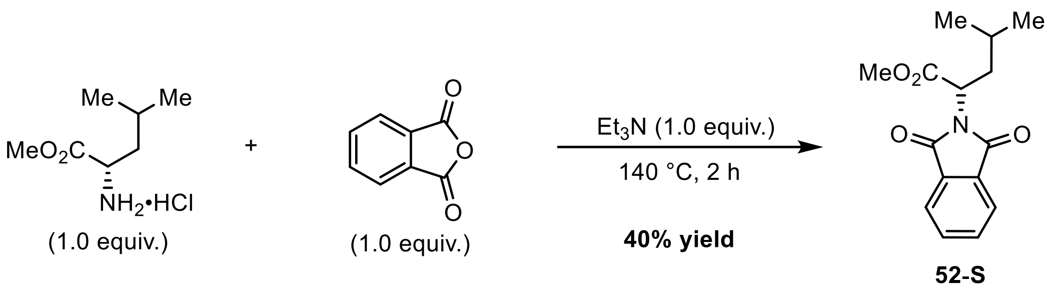

L-Leucine methyl ester hydrochloride (1.81 g, 10.0 mmol, 1.00 equiv.), phthalic anhydride (1.48 g, 10.0 mmol, 1.00 equiv.) and Et_3N (1.02 g, 10.0 mmol, 1.00 equiv.) were added in a sealed microwave vial and stirred at 140 °C for 2 h. After that, the reaction was cooled to room temperature, and diluted with EtOAc (40 mL) and water (20 mL). The layers were separated, and the aqueous layer was extracted with EtOAc (2 × 20 mL). The combined organic layers were dried over $MgSO_4$, filtered, and concentrated *in vacuo*. The residue was purified by flash column chromatography, eluting with EtOAc/petroleum ether (0 – 10%, v/v) to give **52-S** (1.1 g, 40%) as a colorless oil.

R*f* = 0.34 (1:9 EtOAc/petroleum ether, UV, $KMnO_4$)

NMR Spectroscopy (*see spectra*):

^{1}H NMR (400 MHz, $CDCl_3$) δ_H 7.79 (dd, *J* = 5.6, 3.2 Hz, 2H), 7.67 (dd, *J* = 5.6, 3.2 Hz, 2H), 4.88 (dd, *J* = 11.6, 4.4 Hz, 1H), 3.65 (s, 3H), 2.31 – 2.21 (m,1H), 1.94 – 1.84 (m, 1H), 1.49 – 1.35 (m, 1H), 0.87 (d, *J* = 6.8 Hz, 3H), 0.84 (d, *J* = 8.6 Hz, 3H) ppm;

^{13}C NMR (101 MHz, $CDCl_3$) δ_C 170.2, 167.6, 134.2, 131.8, 123.4, 52.6, 50.5, 37.2, 25.0, 23.1, 21.0 ppm.

All recorded spectroscopic data matched those previously reported in the literature.[3]

N-Phth *L*-*tert*-leucine methyl ester (53-S)

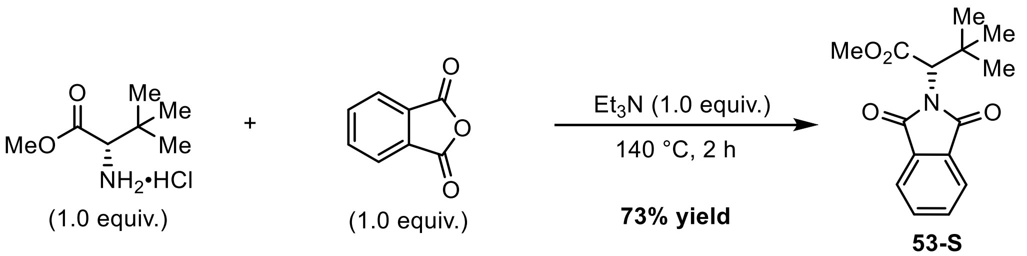

L-*tert*-Leucine methyl ester hydrochloride (1.09 g, 6.00 mmol, 1.00 equiv.), phthalic anhydride (0.89 g, 6.0 mmol, 1.0 equiv.), and Et_3N (0.61 g, 6.0 mmol, 1.0 equiv.) were added in a sealed microwave vial and stirred at 140 °C for 2 h. After that, the reaction was cooled to room temperature, and diluted with EtOAc (40 mL) and water (20 mL). The layers were separated, and the aqueous layer was extracted with EtOAc (2 × 20 mL). The combined organic layers were dried over $MgSO_4$, filtered, and concentrated *in vacuo*. The residue was purified by flash column chromatography, eluting with EtOAc/hexane (0 – 20%, v/v) to give **53-S** (1.2 g, 73%) as a white solid.

R*f* = 0.34 (1:4 EtOAc/hexane, UV, $KMnO_4$)

NMR Spectroscopy (*see spectra*):

^{1}H NMR (400 MHz, $CDCl_3$) δ_H 7.87 (dd, *J* = 5.6, 3.2 Hz, 2H), 7.75 (dd, *J* = 5.6, 3.2 Hz, 2H), 4.63 (s, 1H), 3.66 (s, 3H), 1.17 (s, 9H) ppm;

^{13}C NMR (101 MHz, $CDCl_3$) δ_C 168.4, 168.2, 134.3, 131.9, 123.7, 59.8, 52.2, 36.0, 28.0 ppm.

All recorded spectroscopic data matched those previously reported in the literature.[3]

Dimethoxy bisphenol A (55-S)

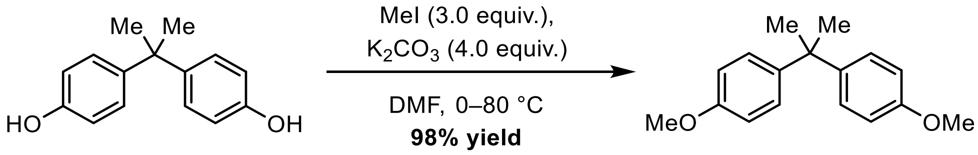

55-S

To a solution of bisphenol A (2.28 g, 10.0 mmol, 1.00 equiv.) in DMF (20 mL, *c* = 0.50 M) was added K_2CO_3 (5.5 g, 40 mmol, 4.0 equiv.). Methyl iodide (4.26 g, 1.87 mL, 30.0 mmol, 3.00 equiv.) was added dropwise at 0 °C and then the mixture was stirred for 15 h at 80 °C. After cooling to room temperature, the reaction was diluted with Et_2O (40 mL) and quenched with aqueous 1 M HCl solution (50 mL). The layers were separated, and the aqueous layer was extracted with Et_2O (2 × 40 mL). The combined organic layers were washed with brine (50 mL), dried over $MgSO_4$, filtered, and concentrated *in vacuo*. Biotage Isolera™ flash purification on silica gel (SNAP 25 g silica cartridge), eluting with Et_2O/pentane (0 – 20%, v/v), gave **55-S** (2.5 g, 98%) as a colorless oil.

R*f* = 0.5 (1:20 EtOAc/hexane, UV, $KMnO_4$)

NMR Spectroscopy (*see spectra*):

^{1}H NMR (400 MHz, $CDCl_3$): δ_H 7.25 – 7.16 (m, 4H), 7.04 – 6.94 (m, 4H), 2.28 (s, 6H), 1.67 (s, 6H) ppm;

^{13}C NMR (101 MHz, $CDCl_3$): δ_C 169.7, 148.7, 148.0, 128.0, 121.1, 42.6, 31.1, 21.3 ppm.

All recorded spectroscopic data matched those previously reported in the literature.[4]

Clofibric ester derivative (56-S)

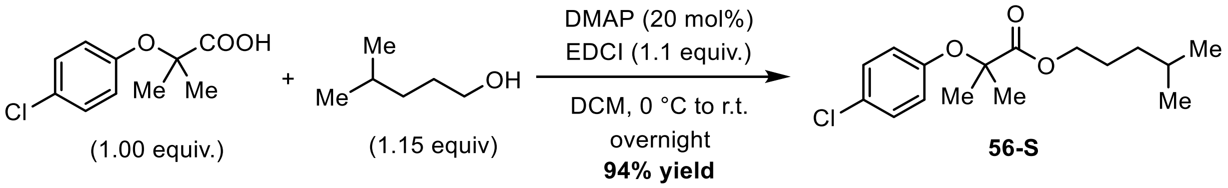

56-S

Clofibric acid (2.2 g, 10 mmol, 1.0 equiv.) and 4-(dimethylamino)pyridine (DMAP, 0.24 g, 2.0 mmol, 20 mol%) were added to a solution of 4-methylpentan-1-ol (1.17 g, 11.5 mmol, 1.15 equiv.) in dry DCM (25 mL, *c* = 0.40 M) at room temperature. 1-Ethyl-3-(3'-dimethylaminopropyl)carbodiimide hydrochloride (EDCI, 1.7 g, 11 mmol, 1.1 equiv.) was added in portions at 0 °C. The resulting suspension was stirred at 0 °C for 2 h and then stirred at room temperature for 10 h. Subsequently, the solvent was removed *in vacuo* and a mixture of EtOAc/H_2O (v/v = 3:1) was added. The layers were separated, and the aqueous layer was extracted with EtOAc twice. The combined organic layers were washed with saturated aqueous $NaHCO_3$ solution, dried over $MgSO_4$, filtered, and concentrated *in vacuo*. The residue was purified by flash column chromatography, eluting with EtOAc/pentane (0 – 2%, v/v) to give **56-S** (2.8 g, 94%) as a colorless oil.

R*f* = 0.70 (1:20 EtOAc/pentane, UV, CAM)

NMR Spectroscopy (*see spectra*):

^{1}**H NMR** (400 MHz, $CDCl_3$) δ_H 7.20 – 7.15 (m, 2H), 6.80 – 6.75 (m, 2H), 4.13 (t, *J* = 6.8 Hz, 2H), 1.64 – 1.54 (m, 2H), 1.58 (s, 6H), 1.48 (dq, *J* = 13.4, 6.8 Hz, 1H), 1.15 – 1.07 (m, 2H), 0.84 (d, *J* = 6.8 Hz, 6H) ppm;

^{13}**C NMR** (101 MHz, $CDCl_3$) δ_C 174.1, 154.3, 129.2, 127.2, 120.4, 79.6, 66.0, 35.0, 27.7, 26.5, 25.5, 22.5 ppm.

HRMS (ESI^+) calcd. for $C_{16}H_{23}O_3Cl$ $[M]^+$, 298.1330, found 298.1328.

Triclosan derivative (57-S)

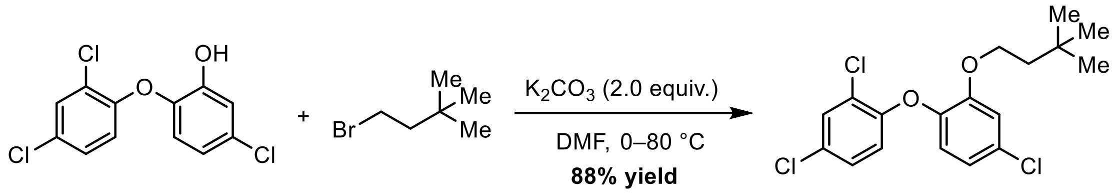

57-S

To a solution of triclosan (1.46 g, 5.00 mmol, 1.00 equiv.) in DMF (10 mL, c = 0.50 M) was added K_2CO_3 (1.4 g, 10 mmol, 1.0 equiv.). 1-Bromo-3,3-dimethylbutane (1.65 g, 1.44 mL, 10.0 mmol, 1.00 equiv.) was added dropwise at 0 °C and then the mixture was stirred for 15 h at 80 °C. After cooling to room temperature, the reaction was diluted with Et_2O (20 mL) and quenched with aqueous 1 M HCl solution (30 mL). The layers were separated, and the aqueous layer was extracted with Et_2O (2 × 20 mL). The combined organic layers were washed with brine (20 mL), dried over $MgSO_4$, filtered, and concentrated *in vacuo*. Biotage Isolera™ flash purification on silica gel (SNAP 25 g silica cartridge), eluting with EtOAc/pentane (0 – 10%, v/v), gave **57-S** (1.7 g, 88%) as a colorless oil.

NMR Spectroscopy (*see spectra*):

^{1}**H NMR** (400 MHz, $CDCl_3$): δ_H 7.42 (d, *J* = 2.4 Hz, 1H), 7.08 (dd, *J* = 8.8, 2.4 Hz, 1H), 6.97 (dd, *J* = 2.0, 0.7 Hz, 1H), 6.96 – 6.87 (m, 2H), 6.62 (d, *J* = 8.8 Hz, 1H), 3.98 (t, *J* = 7.2 Hz, 2H), 1.57 (t, *J* = 7.2 Hz, 2H), 0.88 (s, 9H) ppm;

^{13}**C NMR** (101 MHz, $CDCl_3$): δ_C 152.6, 151.3, 143.1, 130.7, 130.3, 127.9, 127.7, 124.6, 122.2, 121.0, 118.0, 114.9, 66.9, 42.1, 29.8, 29.7 ppm.

IR (film): ν_{max} 2943, 2921, 1598, 1495, 1359, 1263, 1191, 1142, 1100, 770, 746 cm^{-1}.

HRMS (ESI^+): m/z calc'd for $C_{18}H_{19}O_2Cl_3$ $[M]^+$, 372.0445; found, 372.0444.

Probenecid methyl ester (58-S)

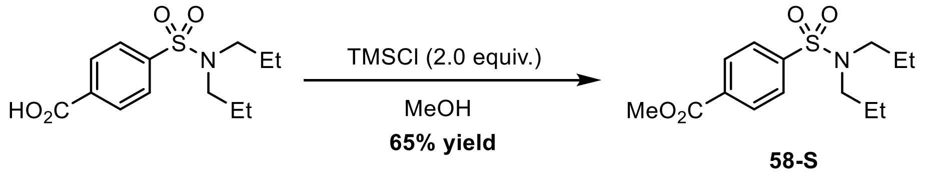

To a solution of probenecid (2.85 g, 10.0 mmol, 1.00 equiv.) in methanol (30 mL, *c* = 0.33 M) was added trimethylsilyl chloride (2.16 g, 2.56 mL, 20.0 mmol, 2.00 equiv). The reaction was stirred at room temperature for 14 h and then filtered through a 20 g silica plug, eluting with 50% EtOAc in hexanes. The filtrate was concentrated *in vacuo*. Biotage Isolera™ flash purification on silica gel (SNAP 25 g silica cartridge), eluting with Et_2O/pentane (0 – 10%, v/v), gave **58-S** (1.9 g, 65%) as a colorless solid.

R*f* = 0.55 (1:20 EtOAc/pentane, UV, CAM)

NMR Spectroscopy (*see spectra*):

^{1}**H NMR** (400 MHz, $CDCl_3$): δ_H 8.14 (d, *J* = 8.4 Hz, 2H), 7.86 (d, *J* = 8.4 Hz, 2H), 3.95 (s, 3H), 3.14 – 3.06 (m, 4H), 1.60 – 1.51 (m, 4H), 0.86 (t, *J* = 7.2 Hz, 6H) ppm;

^{13}**C NMR** (101 MHz, $CDCl_3$): δ_C 165.9, 144.5, 133.6, 130.4, 127.2, 52.7, 50.0, 22.1, 11.3 ppm.

All recorded spectroscopic data matched those previously reported in the literature.[5]

***D*-Galactopyranose ester derivative (59-S)**

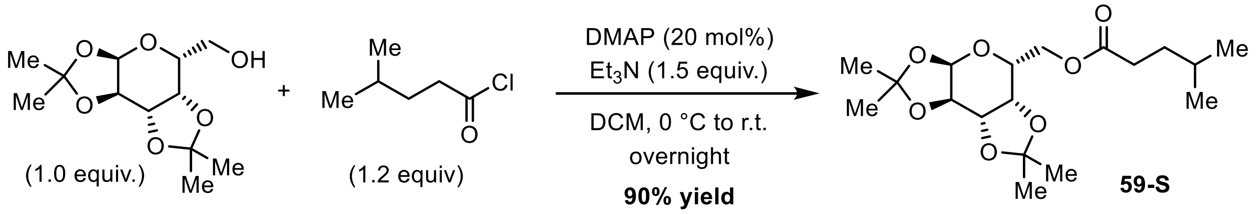

4-(Dimethylamino)pyridine (DMAP, 0.24 g, 2.0 mmol, 20 mol%) and Et_3N (1.5 g, 2.1 mL, 15 mmol, 1.5 equiv.) were added to a solution of 1,2:3,4-di-*O*-isopropylidene-*α*-*D*-galactopyranose (2.60 g, 10.0 mmol, 1.00 equiv.) in dry DCM (40 mL *c* = 0.25 M) at room temperature. The isocaproyl chloride (1.6 g, 12 mmol, 1.2 equiv.), was added dropwise at 0 °C. The resulting suspension was stirred at 0 °C for 1 h and then stirred at room temperature for 10 h. Subsequently, water was added slowly to quench the reaction at 0 °C. After allowing to warm to room temperature, the mixture was extracted with DCM twice. The combined organic layers were washed with brine, dried over $MgSO_4$, filtered, and concentrated *in vacuo.* The residue was purified by flash column chromatography, eluting with Et_2O/pentane (0 – 10%, v/v) to give **59-S** (3.2 g, 90%) as a colorless oil.

R*f* = 0.30 (1:20 EtOAc/pentane, UV, CAM)

NMR Spectroscopy (*see spectra*):

^{1}**H NMR** (400 MHz, $CDCl_3$) δ_H 5.53 (d, *J* = 5.2 Hz, 1H), 4.61 (dd, *J* = 8.0, 2.4 Hz, 1H), 4.34 – 4.28 (m, 2H),

4.23 (dd, J = 8.0, 2.0 Hz, 1H), 4.16 (dd, J = 12.0, 8.0 Hz, 1H), 4.01 – 3.97 (m, 1H), 2.39 – 2.30 (m, 2H), 1.59 – 1.48 (m, 3H), 1.50 (s, 3H), 1.45 (s, 3H), 1.33 (d, J = 3.2 Hz, 6H), 0.89 (s, 3H), 0.87 (s, 3H) ppm;

^{13}C NMR (101 MHz, $CDCl_3$) δ_C 174.1, 109.8, 108.9, 96.5, 71.2, 70.9, 70.6, 66.2, 63.4, 33.9, 32.4, 27.8, 26.2, 26.1, 25.1, 24.6, 22.4, 22.3 ppm.

All recorded spectroscopic data matched those previously reported in the literature.[3]

Flurbiprofen derivative (60-S)

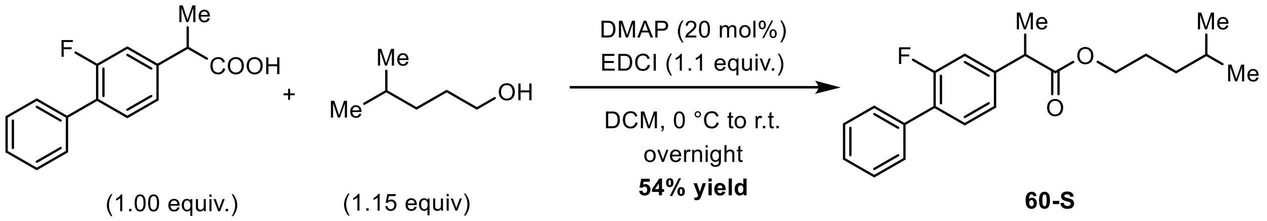

Flurbiprofen (2.44 g, 10.0 mmol, 1.00 equiv.) and 4-(dimethylamino)pyridine (DMAP, 0.24 g, 2.0 mmol, 20 mol%) were added to a solution of 4-methylpentan-1-ol (1.17 g, 11.5 mmol, 1.15 equiv.) in dry DCM (25 mL, c = 0.40 M) at room temperature. 1-Ethyl-3-(3'-dimethylaminopropyl)carbodiimide hydrochloride (EDCI, 1.7 g, 11 mmol, 1.1 equiv.) was added in portions at 0 °C The resulting suspension was stirred at 0 °C for 2 h and then stirred at room temperature for 10 h. Subsequently, the solvent was removed *in vacuo* and a mixture of EtOAc/H_2O (v/v = 3:1) was added. The layers were separated, and the aqueous layer was extracted with EtOAc twice. The combined organic layers were washed with saturated aqueous $NaHCO_3$ solution, dried over $MgSO_4$, filtered, and concentrated *in vacuo*. The residue was purified by flash column chromatography, eluting with EtOAc /pentane (0 – 2%, v/v) to give **60-S** (1.8 g, 54%) as a colorless oil.

R_f = 0.90 (1:30 EtOAc/pentane, UV, CAM)

NMR Spectroscopy (*see spectra*):

^{1}H NMR (400 MHz, $CDCl_3$): δ_H 8.05 – 7.98 (m, 1H), 7.92 – 7.85 (m, 1H), 7.85 – 7.76 (m, 2H), 3.77 – 3.68 (m, 2H), 1.89 – 1.74 (m, 2H), 1.66 – 1.51 (m, 1H), 1.32 – 1.18 (m, 2H), 0.87 (d, J = 6.8 Hz, 6H) ppm;

^{13}C NMR (101 MHz, $CDCl_3$) δ_C 174.2, 159.8 (d, J = 249.5 Hz), 142.2 (d, J = 7.1 Hz), 135.7, 130.9 (d, J = 4.0 Hz), 129.1 (d, J = 3.0 Hz), 128.6, 127.9 (d, J = 3.0 Hz), 127.8, 123.7 (d, J = 4.0 Hz), 115.4 (d, J = 24.2 Hz), 65.5, 45.3 (d, J = 2.0 Hz), 35.0, 27.8, 26.6, 22.6, 18.4 ppm;

^{19}F NMR (377 MHz, $CDCl_3$) δ_F –117.6 ppm.

IR (film) ν_{max}: 2956, 2871, 1732, 1484, 1418, 765, 697 cm^{-1}.

HRMS (ESI^+) calcd. for $C_{21}H_{25}O_2F$ $[M]^+$, 328.1833, found 328.1833.

Saccharin derivative (61-S)

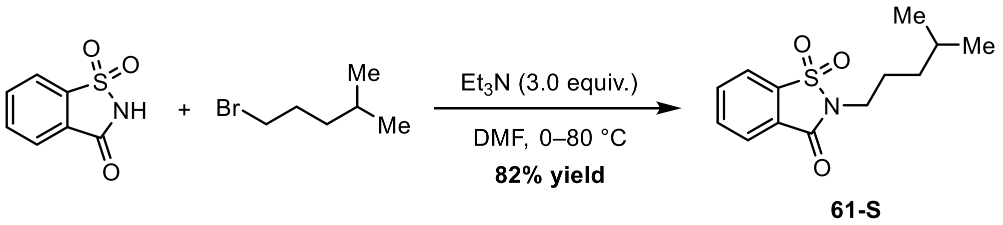

To a solution of saccharin (915 mg, 5.00 mmol, 1.00 equiv.) in DMF (15 mL, *c* = 0.33 M) was added Et_3N (1.5 g, 2.0 mL, 15 mmol, 3.0 equiv.). 1-Bromo-4-methylpentane (1.23 g, 1.15 mL, 7.50 mmol, 1.50 equiv.) was added dropwise at 0 °C and then the mixture was stirred for 15 h at 80 °C. After cooling to room temperature, the reaction was diluted with Et_2O (20 mL) and quenched with aqueous 1 M HCl solution (30 mL). The layers were separated, and the aqueous layer was extracted with Et_2O (2 × 20 mL). The combined organic layers were washed with brine (30 mL), dried over $MgSO_4$, filtered, and concentrated *in vacuo*. Biotage Isolera™ flash purification on silica gel (SNAP 25 g silica cartridge), eluting with EtOAc/pentane (0 – 10%, v/v), gave **61-S** (1.1 g, 82%) as a colorless oil.

R*f* = 0.25 (1:20 EtOAc/pentane, UV, CAM)

NMR Spectroscopy (*see spectra*):

^{1}H NMR (400 MHz, $CDCl_3$): δ_H 8.05 – 7.98 (m, 1H), 7.92 – 7.85 (m, 1H), 7.85 – 7.76 (m, 2H), 3.77 – 3.68 (m, 2H), 1.89 – 1.74 (m, 2H), 1.66 – 1.51 (m, 1H), 1.32 – 1.18 (m, 2H), 0.87 (d, *J* = 6.8 Hz, 6H) ppm;

^{13}C NMR (101 MHz, $CDCl_3$): δ_C 159.0, 137.8, 134.7, 134.3, 127.5, 125.1, 120.9, 39.7, 35.8, 27.6, 26.4, 22.5 ppm.

IR (film): ν_{max} 2955, 2853, 1730, 1463, 1334, 1295, 1183, 1061, 751, 676, 587, 509 cm^{-1}.

HRMS (EI$^+$): m/z calc'd for $C_{13}H_{17}NO_3S$ [M–Me]$^+$, 252.0689; found, 252.0688.

Naproxen derivative (62-S)

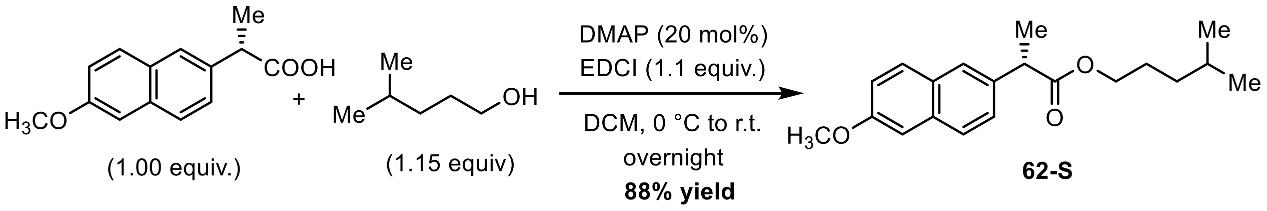

Naproxen (2.3 g, 10 mmol, 1.0 equiv.) and 4-(dimethylamino)pyridine (DMAP, 0.24 g, 2.0 mmol, 20 mol%) were added to a solution of 4-methylpentan-1-ol (1.17 g, 11.5 mmol, 1.15 equiv.) in dry DCM (25 mL, *c* = 0.40 M) at room temperature. 1-Ethyl-3-(3'-dimethylaminopropyl)carbodiimide hydrochloride (EDCI, 1.7 g, 11 mmol, 1.1 equiv.) was added in portions at 0 °C. The resulting suspension was stirred at 0 °C for 2 h and then stirred at room temperature for 10 h. Subsequently, the solvent was removed *in vacuo* and a mixture of EtOAc/H_2O (v/v = 3:1) was added. The layers were separated, and the aqueous layer was extracted with EtOAc twice. The

combined organic layers were washed with saturated aqueous $NaHCO_3$ solution, dried over $MgSO_4$, filtered, and concentrated *in vacuo*. The residue was purified by flash column chromatography, eluting with EtOAc/pentane (0 – 2%, v/v) to give **62-S** (2.8 g, 88%) as a colorless oil.

R*f* = 0.90 (1:30 EtOAc/pentane, UV, CAM)

NMR Spectroscopy (*see spectra*):

¹H NMR (400 MHz, $CDCl_3$) δ_H 7.73 – 7.66 (m, 3H), 7.42 (dd, *J* = 8.4, 2.0 Hz, 1H), 7.18 – 7.09 (m, 2H), 4.06 (t, *J* = 6.8 Hz, 2H), 3.91 (s, 3H), 3.85 (q, *J* = 7.2 Hz, 1H), 1.64 – 1.41 (m, 3H), 1.59 (d, *J* = 8.0 Hz, 3H), 1.15 – 1.07 (m, 2H), 0.81 (d, *J* = 2.4 Hz, 3H), 0.80 (d, *J* = 2.4 Hz, 3H) ppm;

¹³C NMR (101 MHz, $CDCl_3$) δ_C 174.8, 157.7, 136.0, 133.8, 129.4, 129.1, 127.2, 126.4, 126.0, 119.0, 105.7, 65.3, 55.4, 45.7, 35.0, 27.7, 26.6, 22.5, 18.6 ppm.

IR (film) ν_{max}: 2955, 2870, 1729, 1606, 1263, 1175,1157, 1032, 851, 810, 474 cm^{-1}.

HRMS (ESI^+) calcd. for $C_{20}H_{26}O_3$ $[M+H]^+$, 315.1955, found 315.1974.

Oxaprozin ester derivative (63-S)

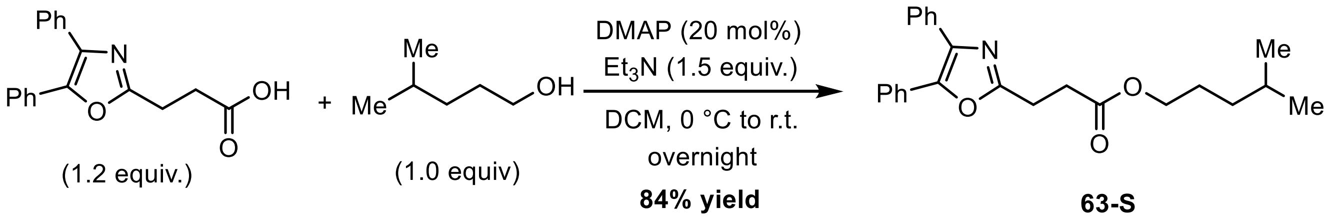

Oxaprozin (2.93 g, 10.0 mmol, 1.00 equiv.) and 4-(dimethylamino)pyridine (DMAP, 0.24 g, 2.0 mmol, 20 mol%) were added to a solution of 4-methylpentan-1-ol (1.17 g, 11.5 mmol, 1.15 equiv.) in dry DCM (20 mL, *c* = 0.5 M) at room temperature. 1-Ethyl-3-(3'-dimethylaminopropyl)carbodiimide hydrochloride (EDCI, 1.7 g, 5.5 mmol, 1.1 equiv.) was added in portions at 0 °C. The resulting suspension was stirred at 0 °C for 2 h and then stirred at room temperature for 10 h. Subsequently, the solvent was removed *in vacuo* and a mixture of EtOAc/H_2O (v/v = 3:1) was added. The layers were separated, and the aqueous layer was extracted with EtOAc twice. The combined organic layers were washed with saturated aqueous $NaHCO_3$ solution, dried over $MgSO_4$, filtered, and concentrated *in vacuo*. The residue was purified by flash column chromatography, eluting with EtOAc/pentane (0 – 10%, v/v) to give **63-S** (3.2 g, 84%) as a colorless oil.

R*f* = 0.20 (1:10 EtOAc/pentane, UV, CAM)

NMR Spectroscopy (*see spectra*):

¹H NMR (400 MHz, $CDCl_3$) δ_H 7.60 – 7.52 (m, 2H), 7.53 – 7.45 (m, 2H), 7.31 – 7.15 (m, 6H), 4.03 (t, *J* = 6.8 Hz, 2H), 3.14 – 3.06 (m, 2H), 2.86 – 2.79 (m, 2H), 1.62 – 1.48 (m, 2H), 1.45 (dq, *J* = 13.2, 6.8 Hz, 1H), 1.19 – 1.09 (m, 2H), 0.79 (d, *J* = 6.8 Hz, 6H) ppm;

¹³C NMR (101 MHz, $CDCl_3$) δ_C 172.2, 162.0, 145.5, 135.3, 132.6, 129.2, 128.8, 128.7, 128.6, 128.2, 128.0, 126.6, 65.4, 35.1, 31.4, 27.8, 26.6, 23.7, 22.6 ppm.

IR (film) v_{max}: 2954, 2870, 1734, 1571, 1444, 1220, 1167, 1057, 962, 762 cm^{-1}.

HRMS (ESI^+) calcd. for $C_{24}H_{27}NO_3$ $[M+H]^+$, 378.2069, found 378.2056.

Lorzone derivative (64-S)

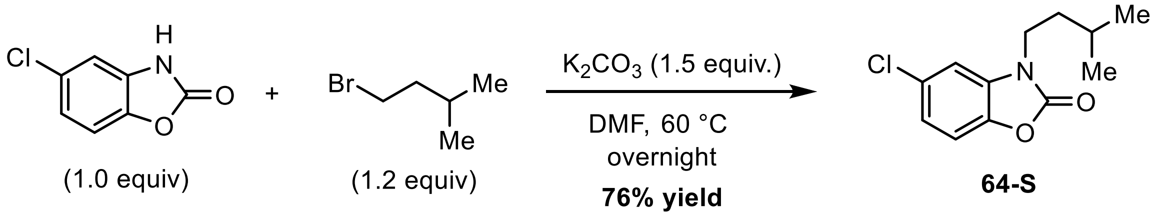

To a solution of lorzone (1.36 g, 8.00 mmol, 1.00 equiv.) in DMF (15 mL, *c* = 0.53 M) was added K_2CO_3 (1.7 g, 12 mmol, 1.5 equiv.). 1-bromo-3-methylbutane (1.45 g, 9.60 mmol, 1.20 equiv.) was added dropwise and then the mixture was stirred for 10 h at 60 °C. After cooling to room temperature, the reaction was diluted with EtOAc (20 mL) and quenched with aqueous 1 M HCl solution (30 mL). The layers were separated, and the aqueous layer was extracted with EtOAc (2 × 20 mL). The combined organic layers were washed with brine (30 mL), dried over $MgSO_4$, filtered, and concentrated *in vacuo*. Biotage Isolera™ flash purification on silica gel (SNAP 25 g silica cartridge), eluting with EtOAc/pentane (0 – 10%, v/v), gave **64-S** (1.6 g, 76%) as a colorless oil.

R_f = 0.40 (1:10 EtOAc/pentane, UV, CAM)

NMR Spectroscopy (*see spectra*):

^{1}H NMR (400 MHz, $CDCl_3$) δ_H 7.11 (d, *J* = 8.4 Hz, 2H), 7.07 (dd, *J* = 8.4, 2.0 Hz, 2H), 6.95 (d, *J* = 2.0 Hz, 1H), 3.85 – 3.74 (m, 2H), 1.72 – 1.57 (m, 3H), 0.99 (d, *J* = 6.4 Hz, 6H) ppm;

^{13}C NMR (101 MHz, $CDCl_3$) δ_C 154.4, 141.3, 132.3, 129.4, 122.3, 111.0, 108.9, 41.1, 36.4, 25.9, 22.5 ppm.

IR (film) v_{max}: 2958, 2871, 1769, 1610, 1484, 1367, 1250, 1060, 997, 948, 801, 749 cm^{-1}.

HRMS (EI^+): m/z calc'd for $C_{12}H_{14}NO_2Cl$ $[M]^+$, 239.0713; found, 239.0703.

Ciprofibrate ester derivative (65-S)

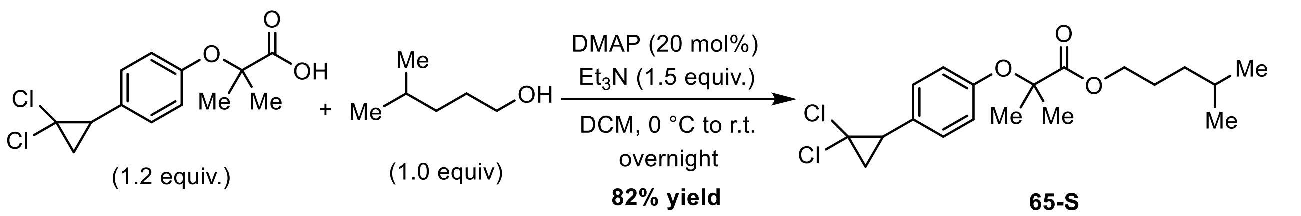

Ciprofibrate (1.45 g, 5.00 mmol, 1.00 equiv.) and 4-(dimethylamino)pyridine (DMAP, 0.12 g, 1.0 mmol, 20 mol%) were added to a solution of 4-methylpentan-1-ol (0.58 g, 5.75 mmol, 1.15 equiv.) in dry DCM (12 mL, *c* = 0.40 M) at room temperature. 1-Ethyl-3-(3'-dimethylaminopropyl)carbodiimide hydrochloride (EDCI, 0.85 g, 5.5 mmol, 1.1 equiv.) was added in portions at 0 °C. The resulting suspension was stirred at 0 °C for 2 h and then stirred

at room temperature for 10 h. Subsequently, the solvent was removed *in vacuo* and a mixture of EtOAc/H_2O (v/v = 3:1) was added. The layers were separated, and the aqueous layer was extracted with EtOAc twice. The combined organic layers were washed with saturated aqueous $NaHCO_3$ solution, dried over $MgSO_4$, filtered, and concentrated *in vacuo*. The residue was purified by flash column chromatography, eluting with EtOAc/pentane (0 – 5%, v/v) to give **65-S** (1.5 g, 82%) as a colorless oil.

R*f* = 0.90 (1:10 EtOAc/pentane, UV, CAM)

NMR Spectroscopy (*see spectra*):

> **^{1}H NMR** (400 MHz, $CDCl_3$) δ_H 7.13 – 7.07 (m, 2H), 6.84 – 6.77 (m, 2H), 4.13 (t, *J* = 6.8 Hz, 2H), 2.82 (dd, *J* = 10.8, 8.4 Hz, 1H), 1.93 (dd, *J* = 10.8, 7.2 Hz, 1H), 1.77 (dd, *J* = 8.4, 7.2 Hz, 1H), 1.60 (s, 6H), 1.63 – 1.54 (m, 3H), 1.53 – 1.43 (m, 1H), 1.15 – 1.08 (m, 2H), 0.83 (d, *J* = 6.4 Hz, 6H) ppm;
>
> **^{13}C NMR** (101 MHz, $CDCl_3$) δ_C 174.4, 155.2, 129.8, 128.2, 118.7, 79.33, 66.0, 61.0, 53.6, 35.0, 27.8, 26.5, 26.0, 25.6, 22.6 ppm.

IR (film) ν_{max}: 2954, 2871, 1731, 1611, 1511, 1467, 1242, 1136, 964, 831, 760 cm^{-1}.

HRMS (ESI$^+$) calcd. for $C_{19}H_{26}O_3Cl_2$ $[M+Na]^+$, 395.1157, found 395.1144.

Celecoxib derivative (67-S)

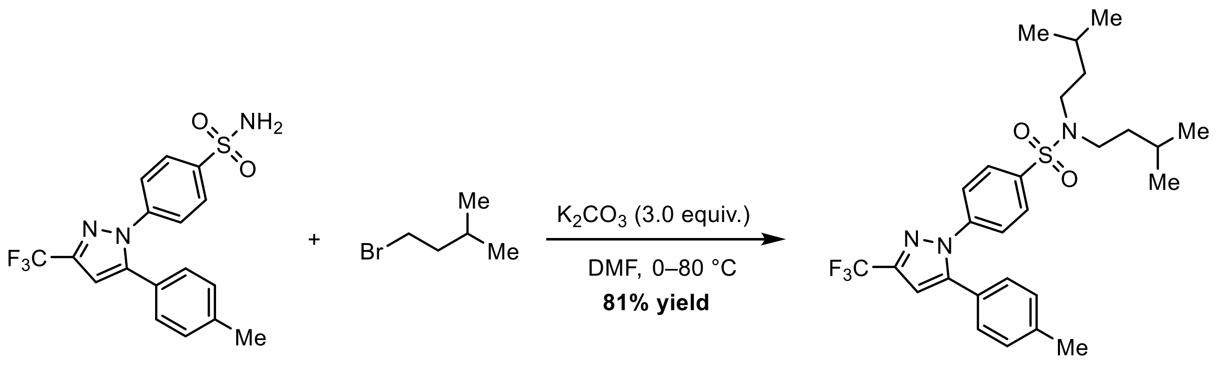

67-S

To a solution of celecoxib (762 mg, 2.00 mmol, 1.00 equiv.) in DMF (6 mL, c = 0.33 M) was added K_2CO_3 (0.82 g, 6.0 mmol, 3.0 equiv.). 1-Bromo-3,3-dimethylbutane (0.75 g, 0.60 mL, 5.0 mmol, 2.5 equiv.) was added dropwise at 0 °C and then the mixture was stirred for 15 h at 80 °C. After cooling to room temperature, the reaction was diluted with Et_2O (20 mL) and quenched with aqueous 1 M HCl solution (30 mL). The layers were separated, and the aqueous layer was extracted with Et_2O (2 × 20 mL). The combined organic layers were washed with brine (20 mL), dried over $MgSO_4$, filtered, and concentrated *in vacuo*. Biotage Isolera™ flash purification on silica gel (SNAP 25 g silica cartridge), eluting with EtOAc/pentane (5 – 20%, v/v), gave **67-S** (892 mg, 81%) as a colorless solid.

R*f* = 0.35 (1:10 EtOAc/pentane, CAM)

NMR Spectroscopy (*see spectra*):

^{1}H NMR (400 MHz, $CDCl_3$): δ_H 7.79 (d, *J* = 8.7 Hz, 2H), 7.45 (d, *J* = 8.7 Hz, 2H), 7.16 (d, *J* = 7.9 Hz, 2H), 7.09 (d, *J* = 8.1 Hz, 2H), 6.74 (s, 1H), 3.16 – 3.08 (m, 4H), 2.37 (s, 3H), 1.56 (dt, *J* = 13.2, 6.6 Hz, 2H), 1.44 – 1.34 (m, 4H), 0.88 (d, *J* = 6.6 Hz, 12H) ppm;

^{13}C NMR (101 MHz, $CDCl_3$): δ_C 145.3, 144.1 (q, *J* = 38.5 Hz), 142.3, 139.8 (d, J = 4.8 Hz), 129.8, 128.8, 128.2, 125.8, 125.6, 121.2 (q, J = 269.0 Hz), 106.3, 46.5, 37.4, 25.9, 22.5, 21.4 ppm;

^{19}F NMR (377 MHz, $CDCl_3$): δ_F –62.42 (s, CF_3);

All recorded spectroscopic data matched those previously reported in the literature.[6]

2.5. Substrate Scope

2-Cyclohexyl-4,4,5,5-tetramethyl-1,3,2-dioxaborolane (1)

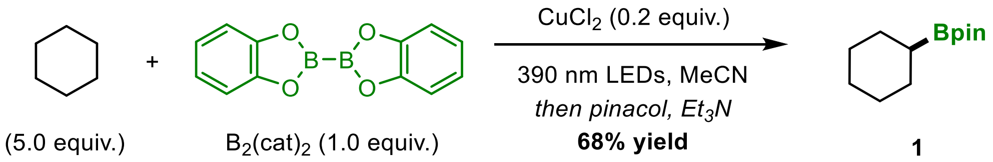

Prepared following **General Procedure A**, using cyclohexane (126 mg, 162 μL, 1.50 mmol, 5.00 equiv.), $CuCl_2$ (8 mg, 0.06 mmol, 0.2 equiv.), and B_2cat_2 (71 mg, 0.30 mmol, 1.0 equiv.) in MeCN (1.5 mL, *c* = 0.20 M). Biotage Isolera™ flash purification on silica gel (SNAP 5 g silica cartridge), eluting with Et_2O/pentane (0 – 5%, v/v), gave **1** (40.2 mg, 64%) as a colorless oil.

Prepared following **General Procedure A-prestirring**, boronic ester **1** was isolated in **68%** yield (42.7 mg).

When the reaction was performed using **1.0 equivalent** of cyclohexane (26 mg, 33 μL, 0.30 mmol, 1.0 equiv.), $CuCl_2$ (80 mg, 0.60 mmol, 2.0 equiv.), and B_2cat_2 (142 mg, 0.600 mmol, 2.00 equiv.) in MeCN (1.5 mL, *c* = 0.20 M), boronic ester was isolated in 45% yield (28.6 mg).

R*f* = 0.35 (1:9 EtOAc/pentane, CAM)

NMR Spectroscopy (*see spectra*):

- ^{1}**H NMR** (400 MHz, $CDCl_3$): δ_H 1.70 – 1.53 (m, 5H), 1.39 – 1.25 (m, 5H), 1.23 (s, 12H), 1.02 – 0.93 (m, 1H) ppm;
- ^{13}**C NMR** (101 MHz, $CDCl_3$): δ_C 82.9, 28.1, 27.3, 26.9, 24.9, 24.7 ppm. The carbon attached to boron was not observed due to quadrupolar relaxation.

All recorded spectroscopic data matched those previously reported in the literature.[7]

2-Cyclopentyl-4,4,5,5-tetramethyl-1,3,2-dioxaborolane (2)

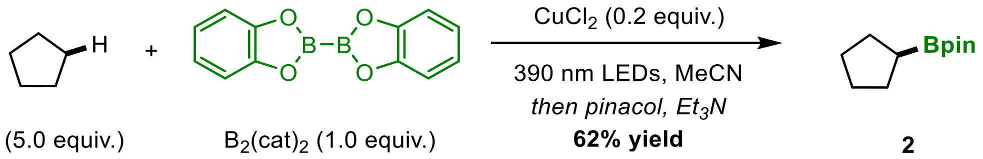

Prepared following **General Procedure A**, using cyclopentane (105 mg, 140 μL, 1.50 mmol, 5.00 equiv.), $CuCl_2$ (8 mg, 0.06 mmol, 0.2 equiv.), and B_2cat_2 (71 mg, 0.30 mmol, 1.0 equiv.) in MeCN (1.5 mL, *c* = 0.20 M). Biotage Isolera™ flash purification on silica gel (SNAP 5 g silica cartridge), eluting with Et_2O/pentane (0 – 5%, v/v), gave **2** (36.3 mg, **62%**) as a colorless oil.

Prepared following **General Procedure A-prestirring**, boronic ester **2** was isolated in 60% yield (34.8 mg).

R*f* = 0.35 (1:9 EtOAc/pentane, CAM)

NMR Spectroscopy (see spectra):

^{1}**H NMR** (400 MHz, $CDCl_3$): δ_H 1.85 – 1.67 (m, 2H), 1.66 – 1.54 (m, 2H), 1.54 – 1.37 (m, 4H), 1.23 (s, 12H), 1.20 – 1.12 (m, 1H) ppm;

^{13}**C NMR** (101 MHz, $CDCl_3$): δ_C 82.9, 28.6, 27.0, 24.9 ppm. The carbon attached to boron was not observed due to quadrupolar relaxation.

All recorded spectroscopic data matched those previously reported in the literature.[7]

2-Cycloheptyl-4,4,5,5-tetramethyl-1,3,2-dioxaborolane (3)

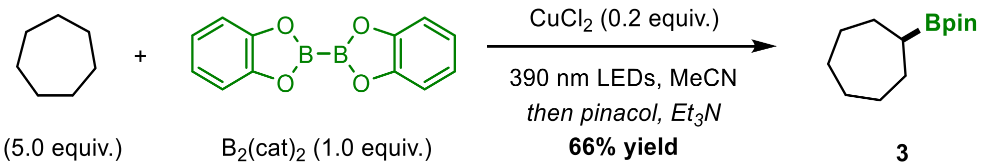

3

Prepared following **General Procedure A**, using cycloheptane (147 mg, 181 μL, 1.50 mmol, 5.00 equiv.), $CuCl_2$ (8 mg, 0.06 mmol, 0.2 equiv.), and B_2cat_2 (71 mg, 0.30 mmol, 1.0 equiv.) in MeCN (1.5 mL, c = 0.20 M). Biotage Isolera™ flash purification on silica gel (SNAP 5 g silica cartridge), eluting with Et_2O/pentane (0 – 5%, v/v), gave **3** (40.3 mg, 60%) as a colorless oil.

Prepared following **General Procedure A-prestirring**, boronic ester **3** was isolated in **66%** yield (44.2 mg).

R*f* = 0.35 (1:9 EtOAc/pentane, CAM)

NMR Spectroscopy (see spectra):

^{1}**H NMR** (400 MHz, $CDCl_3$): δ_H 1.79 – 1.60 (m, 4H), 1.60 – 1.39 (m, 8H), 1.22 (s, 12H), 1.09 – 1.03 (m, 1H) ppm;

^{13}**C NMR** (101 MHz, $CDCl_3$): δ_C 82.7, 29.6, 28.9, 28.3, 24.7 ppm. The carbon attached to boron was not observed due to quadrupolar relaxation.

All recorded spectroscopic data matched those previously reported in the literature.[8]

2-Cyclododecyl-4,4,5,5-tetramethyl-1,3,2-dioxaborolane (4)

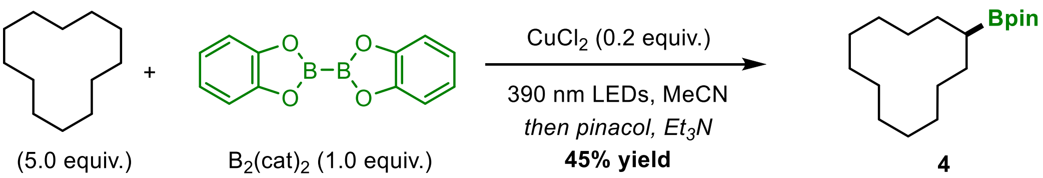

4

Prepared following **General Procedure A**, using cyclododecane (252 mg, 1.50 mmol, 5.00 equiv.), $CuCl_2$ (8 mg, 0.06 mmol, 0.2 equiv.) and B_2cat_2 (71 mg, 0.30 mmol, 1.0 equiv.) in MeCN (6.0 mL, c = 50 mM). The irradiation time is 40 h. Biotage Isolera™ flash purification on silica gel (SNAP 5 g silica cartridge), eluting with

Et_2O/pentane (0 – 5%, v/v), gave **4** (36.6 mg, 41%) as a colorless solid.

Prepared following **General Procedure A-prestirring**, boronic ester **4** was isolated in **45%** yield (39.5 mg).

R*f* = 0.50 (1:9 EtOAc/pentane, CAM)

NMR Spectroscopy (*see spectra*):

> **^{1}H NMR** (400 MHz, $CDCl_3$): δ_H 1.46 – 1.25 (m, 22H), 1.23 (s, 12H), 1.10 – 1.03 (m, 1H). ppm;
>
> **^{13}C NMR** (101 MHz, $CDCl_3$): δ_C 82.9, 25.0, 24.9, 24.4, 24.2, 23.8, 23.6, 23.5, 23.4 ppm. The carbon attached to boron was not observed due to quadrupolar relaxation.

All recorded spectroscopic data matched those previously reported in the literature.[9]

2-((1*R**,2*R**,4*R**)-Bicyclo[2.2.1]heptan-2-yl)-4,4,5,5-tetramethyl-1,3,2-dioxaborolane (5)

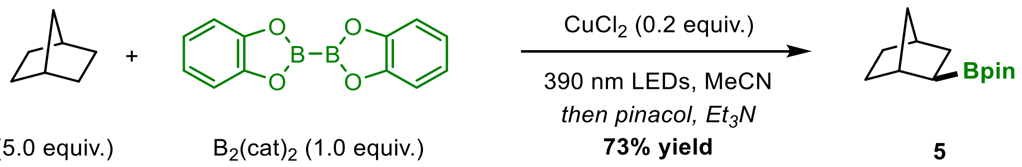

Prepared following **General Procedure A**, using norbornane (144 mg, 1.50 mmol, 5.00 equiv.), $CuCl_2$ (8 mg, 0.06 mmol, 0.2 equiv.), and B_2cat_2 (71 mg, 0.30 mmol, 1.0 equiv.) in MeCN (1.5 mL, *c* = 0.20 M). Biotage Isolera™ flash purification on silica gel (SNAP 5 g silica cartridge), eluting with Et_2O/pentane (0 – 5%, v/v), gave **5** (44.9 mg, 67%) as a colorless oil. The r.r. (>98:2) was determined by GC-FID analysis of the crude reaction mixture. The *exo*:*endo* (86:14) was determined by averaging the ratio of carbon resonances in the ^{13}C NMR of the purified product. Unreacted norbornane was recovered in 88% yield (109 mg).

Prepared following **General Procedure A-prestirring**, boronic ester **5** was isolated in **73%** yield (48.6 mg).

When the reaction was performed using **3.0 equivalents** of norbornane (86 mg, 0.90 mmol, 3.0 equiv.), boronic ester **5** was isolated in 52% yield (34.6 mg). Unreacted norbornane was recovered in 87% yield (62 mg).

When the reaction was performed using **1.0 equivalent** of norbornane (29 mg, 0.30 mmol, 1.0 equiv.), $CuCl_2$ (80 mg, 0.60 mmol, 2.0 equiv.), and B_2cat_2 (142 mg, 0.600 mmol, 2.00 equiv.) in MeCN (1.5 mL, *c* = 0.20 M), boronic ester **5** was isolated in 44% yield (29.5 mg). The irradiation time is 48 h.

R*f* = 0.30 (1:9 EtOAc/pentane, CAM)

NMR Spectroscopy (*see spectra*):

> **^{1}H NMR** (400 MHz, $CDCl_3$): δ_H (*exo*:*endo* = 86:14) 2.39 – 2.18 (m, 2H), 1.66 – 1.41 (m, 3H), 1.38 – 1.28 (m, 1H), 1.26 – 1.10 (m, 4H), 1.21 (s, 12H), 0.92 – 0.82 (m, 1H) ppm;
>
> **^{13}C NMR** (101 MHz, $CDCl_3$): δ_C (*exo*) 82.7, 38.7, 38.1, 36.6, 32.2, 32.2, 29.3, 24.7 ppm. The carbon attached to boron was not observed due to quadrupolar relaxation.
>
> **^{13}C NMR** (101 MHz, $CDCl_3$): δ_C (*endo*) 82.8, 41.1, 39.0, 37.3, 31.9, 30.0, 28.0, 24.9, 24.9 ppm. The carbon

attached to boron was not observed due to quadrupolar relaxation.

All recorded spectroscopic data matched those previously reported in the literature.[10]

(2*R,4a*R**,8a*R**)-Decahydronaphthalen-2-ol (6-ox)**

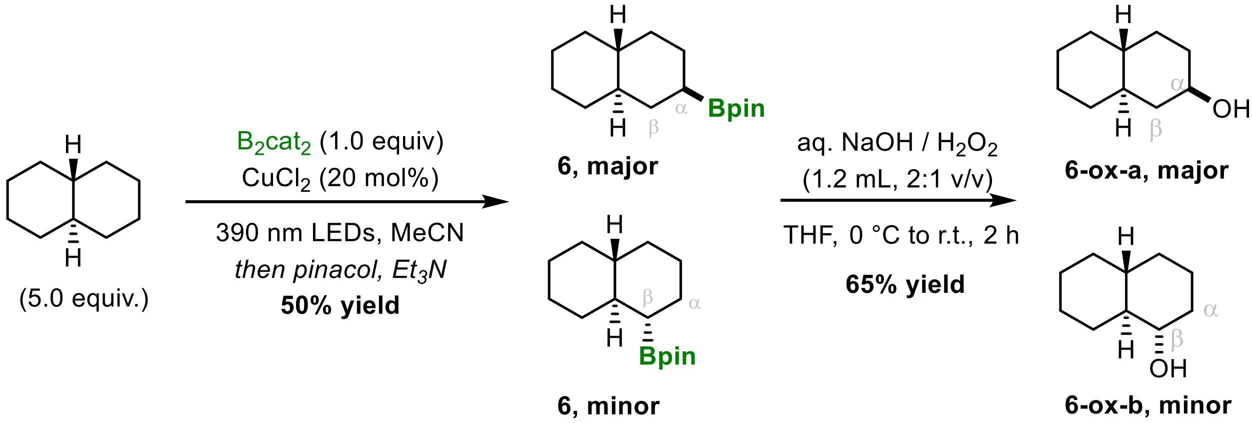

Prepared following **General Procedure A**, using *trans*-decahydronaphthalene (207 mg, 238 μL, 1.50 mmol, 5.00 equiv.), $CuCl_2$ (8.0 mg, 0.060 mmol, 0.20 equiv.) and B_2cat_2 (71 mg, 0.30 mmol, 1.0 equiv.) in MeCN (1.5 mL, *c* = 0.20 M). Flash column chromatography, eluting with Et_2O/pentane (0 – 2%, v/v) on silica gel gave **6** (32 mg, 40%) as a colorless oil. The d.r. and r.r. could not be determined by ^1H NMR or GC-FID analysis, therefore, boronic ester **6** was oxidized to the alcohol **6-ox**.

Prepared following **General Procedure A-prestirring**, boronic ester **6** was isolated in **50%** yield (40 mg).

R*f* = 0.70 (1:20 EtOAc/pentane, CAM)

To a solution of boronic ester **6** (32 mg, 0.12 mmol, 1.0 equiv.) in THF (2.0 mL) at 0 °C was added dropwise a solution of 2 M aqueous NaOH/30% aqueous H_2O_2 (1.2 mL, 2:1 v/v). The mixture was stirred at 0 °C for 1 h and then at room temperature for 1 h. Water (5 mL) and EtOAc (20 mL) were added and the layers were separated. The aqueous layer was extracted with EtOAc (3 × 15 mL) and the combined organic layers were washed with brine, dried over $MgSO_4$, filtered, and concentrated *in vacuo*. The r.r. (81:19 α:β) and d.r. (α = 95:5; β = >97:3) were determined by ^1H NMR analysis of the crude reaction mixture (see spectrum). The residue was purified by flash column chromatography, eluting with Et_2O/pentane (0 – 10%, v/v) to give **6-ox** (12 mg, 65%) as a colorless oil.

R*f* (**6-ox-a**, major) = 0.1 (1:10 EtOAc/pentane, CAM)

NMR Spectroscopy of 6-ox-a (*see spectra*):

^{1}H NMR (400 MHz, $CDCl_3$) δ_H 3.65 – 3.55 (m, 1H), 2.06 – 1.91 (m, 1H), 1.89 – 1.83 (m, 1H), 1.77 – 1.55 (m, 5H), 1.36 (s, 1H), 1.31 – 1.12 (m, 3H), 1.08 – 0.75 (m, 6H) ppm;

^{13}C NMR (101 MHz, $CDCl_3$) δ_C 71.0, 43.4, 42.5, 41.4, 36.0, 34.0, 33.4, 32.1, 26.7, 26.4 ppm.

R*f* (**6-ox-b**, minor) = 0.2 (1:10 EtOAc/pentane, CAM)

NMR Spectroscopy of 6-ox-b (*see spectra*):

^{1}H NMR (400 MHz, $CDCl_3$) δ_H 3.25 – 3.13 (m, 1H), 2.16 – 2.06 (m, 1H), 2.00 – 1.91 (m, 1H), 1.83 – 1.59 (m, 5H), 1.53 – 1.47 (m, 1H), 1.32 – 1.15 (m, 3H), 1.07 – 0.77 (m, 6H).

^{13}C NMR (101 MHz, $CDCl_3$) δ_C 75.2, 50.6, 41.3, 36.0, 33.7, 33.6, 29.2, 26.5, 26.3, 24.2 ppm.

All recorded spectroscopic data matched those previously reported in the literature.[3]

(1*R,2*S**,4*R**)-5-Chlorobicyclo[2.2.1]heptan-2-ol (7-ox)**

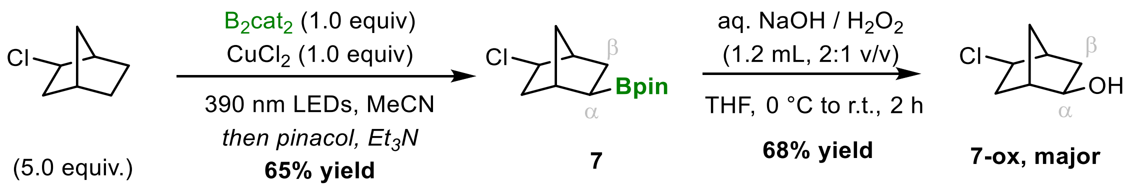

Prepared following **General Procedure B**, using *exo*-2-chloronorbornane (196 mg, 185 µL, 1.50 mmol, 5.00 equiv.), $CuCl_2$ (40 mg, 0.30 mmol, 1.0 equiv.) and B_2cat_2 (71 mg, 0.30 mmol, 1.0 equiv.) in MeCN (1.5 mL, c = 0.20 M). Flash column chromatography (1:50 Et_2O/pentane) on silica gel gave **7** (50 mg, **65%**) as a colorless oil. The d.r. and r.r. could not be determined by ^{1}H NMR or GC-FID analysis, therefore, the boronic ester **7** was oxidized to alcohol **7-ox**.

R*f* = 0.60 (1:20 EtOAc/pentane, CAM)

Prepared following **General Procedure B-prestirring**, boronic ester was isolated in **65%** yield (50 mg).

To a solution of boronic ester **7** (50 mg, 0.19 mmol, 1.0 equiv.) in THF (4.0 mL) at 0 °C was added dropwise a solution of 2 M aqueous NaOH/30% aqueous H_2O_2 (2.4 mL, 2:1 v/v). The mixture was stirred at 0 °C for 1 h and then at room temperature for 1 h. Water (5 mL) and EtOAc (20 mL) were added, and the layers were separated. The aqueous layer was extracted with EtOAc (3 × 15 mL) and the combined organic layers were washed with brine, dried over $MgSO_4$, filtered, and concentrated *in vacuo*. The r.r. (91:9 α:β) and d.r. (α = 88:12; β = 75:25) were determined by ^{1}H NMR analysis of the crude reaction mixture (*see spectrum*). The residue was purified by flash column chromatography (33% EtOAc/pentane) to give **7-ox** (19 mg, 68%) as a colorless oil.

R*f* (**7-ox**, major) = 0.1 (1:5 EtOAc/pentane, CAM)

NMR Spectroscopy of 7-ox (*see spectra*):

^{1}H NMR (400 MHz, $CDCl_3$) δ_H 3.81 – 3.73 (m, 1H), 3.72 (d, J = 6.8 Hz, 1H), 2.43 (dd, J = 5.2, 1.6 Hz, 1H), 2.24 (m, 1H), 1.81 – 1.76 (m, 3H), 1.73 – 1.67 (m, 1H), 1.66 – 1.57 (m, 2H), 1.48 – 1.34 (m, 1H) ppm;

^{13}C NMR (101 MHz, $CDCl_3$) δ_C 73.4, 61.0, 45.2, 44.4, 39.2, 38.3, 31.3 ppm.

All recorded spectroscopic data matched those previously reported in the literature.[3]

(1*R,2*R**,4*S**,7*R**)-7-Bromo-bicyclo[2.2.1]heptan-2-ol (8-ox-a) and (1*S**,2*R**,4*R**,7*R**)-7-bromo-bicyclo[2.2.1]heptan-2-ol (8-ox-b)**

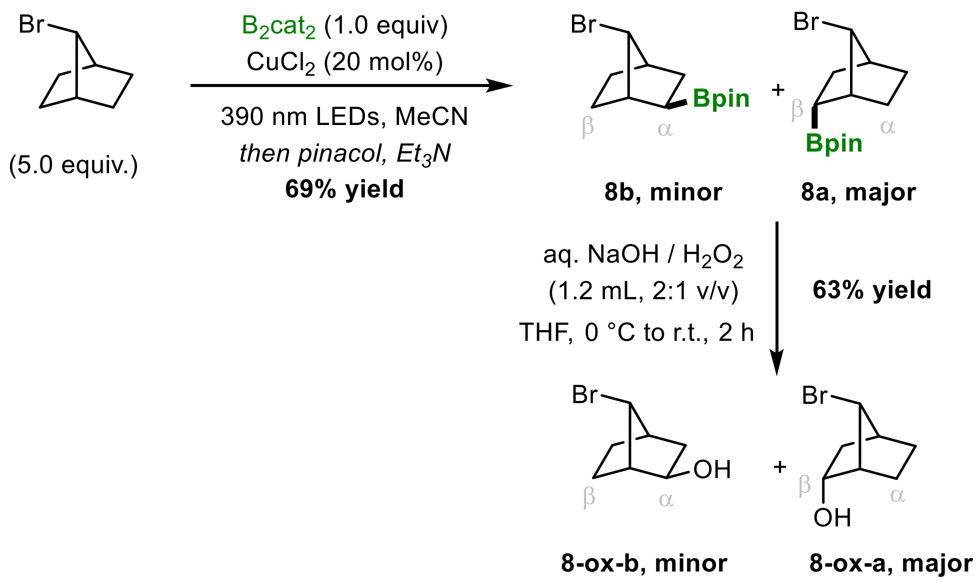

Prepared following **General Procedure A**, using 7-bromobicyclo[2.2.1]heptane (263 mg, 193 μL, 1.50 mmol, 5.00 equiv.), $CuCl_2$ (8.0 mg, 0.060 mmol, 0.20 equiv.) and B_2cat_2 (71 mg, 0.30 mmol, 1.0 equiv.) in MeCN (1.5 mL, *c* = 0.20 M). Flash column chromatography (1:50 Et_2O/pentane) on silica gel gave **8** (44 mg, 49%) as a colorless oil. The d.r. and r.r. could not be determined by ^{1}H NMR or GC-FID analysis, therefore, the boronic ester **8** was oxidized to the alcohol **8-ox**.

Prepared following **General Procedure B-prestirring**, boronic ester **8** was isolated in **69%** yield (62 mg).

R*f* = 0.60 (1:20 EtOAc/pentane, CAM)

To a solution of boronic ester **8** (44 mg, 0.15 mmol, 1.0 equiv.) in THF (2.0 mL) at 0 °C was added dropwise a solution of 2 M aqueous NaOH/30% aqueous H_2O_2 (1.5 mL, 2:1 v/v). The mixture was stirred at 0 °C for 1 h and then at room temperature for 1 h. Water (5 mL) and EtOAc (20 mL) were added, and the layers were separated. The aqueous layer was extracted with EtOAc (3 × 15 mL) and the combined organic layers were washed with brine, dried over $MgSO_4$, filtered, and concentrated *in vacuo*. The r.r. (74:26 α:β) and d.r. (α = 92:8; β = >97:3) were determined by ^{1}H NMR analysis of the crude reaction mixture (***see spectrum***). The residue was purified by flash column chromatography (10% EtOAc/pentane) to give **8-ox** (18 mg, 63%) as a colorless oil.

R*f* (**8-ox-a**, major) = 0.3 (1:10 EtOAc/pentane, CAM)

NMR Spectroscopy of 8-ox-a (*see spectra*):

^{1}H NMR (400 MHz, $CDCl_3$) δ_H 4.80 – 4.70 (m, 1H), 4.06 (dd, *J* = 2.0, 2.0 Hz, 1H), 2.50 – 2.45 (m, 1H), 2.39 (t, *J* = 6.0 Hz, 1H), 2.29 (t, *J* = 4.8 Hz, 1H), 2.12 – 2.00 (m, 1H), 1.77 – 1.62 (m, 1H), 1.55 – 1.39 (m, 3H), 1.07 (dt, *J* = 13.2, 2.8 Hz, 1H) ppm;

^{13}C NMR (101 MHz, $CDCl_3$) δ_C 71.9, 58.3, 48.1, 43.7, 37.4, 27.7, 18.7 ppm.

R*f* (**8-ox**, minor) = 0.2 (1:10 EtOAc/pentane, CAM)

NMR Spectroscopy of 8-ox-b (*see spectra*):

^{1}H NMR (400 MHz, $CDCl_3$) δ_H 4.30 (m, 1H), 3.86 (dt, *J* = 7.6, 2.4 Hz, 1H), 2.42 – 2.31 (m, 1H), 2.28 – 2.24 (m, 1H), 2.00 – 1.91 (m, 2H), 1.85 (dd, *J* = 13.2, 7.6 Hz, 1H), 1.55 – 1.40 (m, 2H), 1.22 – 1.02 (m, 2H) ppm;

^{13}C NMR (101 MHz, $CDCl_3$) δ_C 73.0, 56.0, 51.4, 42.2, 40.4, 26.2, 22.7 ppm.

All recorded spectroscopic data matched those previously reported in the literature.[3]

2-((1*R*,3*S*,5*r*,7*r*)-Adamantan-2-yl)-4,4,5,5-tetramethyl-1,3,2-dioxaborolane (9a) and 2-((3*r*,5*r*,7*r*)-adamantan-1-yl)-4,4,5,5-tetramethyl-1,3,2-dioxaborolane (9b)

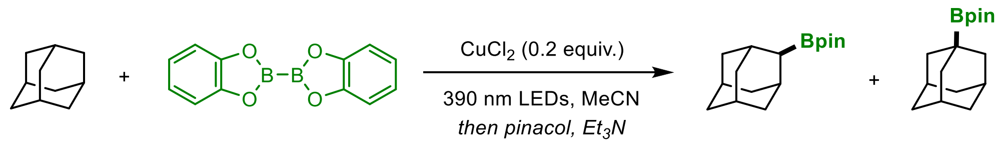

Prepared following **General Procedure A**, using adamantane (204 mg, 1.50 mmol, 5.00 equiv.), $CuCl_2$ (8 mg, 0.06 mmol, 0.2 equiv.), and B_2cat_2 (71 mg, 0.30 mmol, 1.0 equiv.) in MeCN (6.0 mL, c = 50 mM). The irradiation time is 40 h. Biotage Isolera™ flash purification on silica gel (SNAP 5 g silica cartridge), eluting with Et_2O/pentane (0 – 5%, v/v), gave a mixture of **9a** and **9b** (44.4 mg, 56%) as a colorless solid. The r.r. (87:13, **9a**:**9b**) was determined by GC-FID analysis of the crude reaction mixture (*see spectrum*).

Prepared following **General Procedure A-prestirring**, boronic ester **9** was isolated in **58%** yield (45.9 mg).

R*f* = 0.30 (1:9 EtOAc/pentane, CAM)

NMR Spectroscopy of 9a (*see spectra*):

^{1}H NMR (400 MHz, $CDCl_3$): δ_H 2.08 – 2.02 (m, 2H), 1.90 – 1.66 (m, 12H), 1.38 – 1.33 (m, 1H), 1.25 (s, 12H) ppm;

^{13}C NMR (101 MHz, $CDCl_3$): δ_C 82.9, 39.5, 37.9, 36.4, 29.5, 28.4, 28.2, 25.0 ppm. The carbon attached to boron was not observed due to quadrupolar relaxation.

All recorded spectroscopic data matched those previously reported in the literature.[6]

NMR Spectroscopy of 9b (*see spectra*):

^{1}H NMR (400 MHz, $CDCl_3$): δ_H 1.90 – 1.66 (overlapped m, 15H), 1.21 (s, 12H) ppm;

^{13}C NMR (101 MHz, $CDCl_3$): δ_C 82.3, 38.1, 37.7, 27.7, 24.8 ppm. The carbon attached to boron was not observed due to quadrupolar relaxation.

All recorded spectroscopic data matched those previously reported in the literature.[7]

4,4,5,5-Tetramethyl-2-pentyl-1,3,2-dioxaborolane (10a), 4,4,5,5-tetramethyl-2-(pentan-2-yl)-1,3,2-dioxaborolane (10b), and 4,4,5,5-tetramethyl-2-(pentan-3-yl)-1,3,2-dioxaborolane (10c)

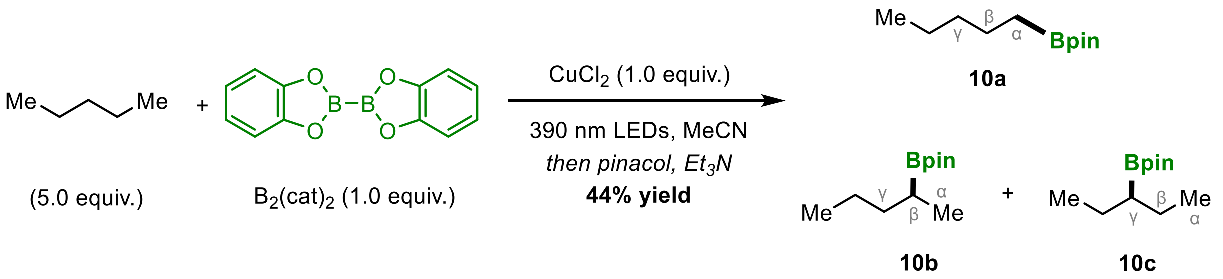

Prepared following **General Procedure B**, using pentane (108 mg, 172 μL, 1.50 mmol, 5.00 equiv.), $CuCl_2$ (40 mg, 0.30 mmol, 1.0 equiv.), and B_2cat_2 (71 mg, 0.30 mmol, 1.0 equiv.) in MeCN (1.5 mL, c = 0.20 M). Biotage Isolera™ flash purification on silica gel (SNAP 5 g silica cartridge), eluting with Et_2O/pentane (0 – 5%, v/v), gave a mixture of **10a**, **10b** and **10c** (20.8 mg, 35%) as a colorless oil. The r.r. (65:29:6, **10a:10b:10c**) was determined by GC-FID analysis of the crude reaction mixture (***see spectrum***).

Prepared following **General Procedure B-prestirring**, boronic ester **10** was isolated in **44%** yield (26 mg).

R*f* = 0.40 (1:10 EtOAc/pentane, CAM)

NMR Spectroscopy of 10a (*see spectra*):

^{1}H NMR (400 MHz, $CDCl_3$): δ_H 1.44 – 1.24 (overlapped m, 9H), 1.23 (s, 12H), 0.75 (t, *J* = 7.8 Hz, 3H) ppm;

^{13}C NMR (101 MHz, $CDCl_3$): δ_C 83.0, 34.8, 25.0, 23.8, 22.6, 14.2 ppm. The carbon attached to boron was not observed due to quadrupolar relaxation.

NMR Spectroscopy of 10b (*see spectra*):

^{1}H NMR (400 MHz, $CDCl_3$): δ_H 1.44 – 1.24 (overlapped m, 5H), 1.22 (s, 12H), 0.89 – 0.84 (overlapped m, 7H) ppm;

^{13}C NMR (101 MHz, $CDCl_3$): δ_C 82.9, 35.6, 24.9, 24.9, 22.2, 15.6, 14.5 ppm. The carbon attached to boron was not observed due to quadrupolar relaxation.

NMR Spectroscopy of 10c (*see spectra*):

^{1}H NMR (400 MHz, $CDCl_3$): δ_H 1.44 – 1.24 (overlapped m, 5H), 1.24 (s, 12H), 0.96 – 0.93 (overlapped m, 1H), 0.89 – 0.84 (overlapped m, 6H) ppm;

^{13}C NMR (101 MHz, $CDCl_3$): δ_C 82.9, 24.9, 24.1, 13.8 ppm. The carbon attached to boron was not observed due to quadrupolar relaxation.

All recorded spectroscopic data matched those previously reported in the literature.[7]

2-(2,3-Dimethylbutyl)-4,4,5,5-tetramethyl-1,3,2-dioxaborolane (11)

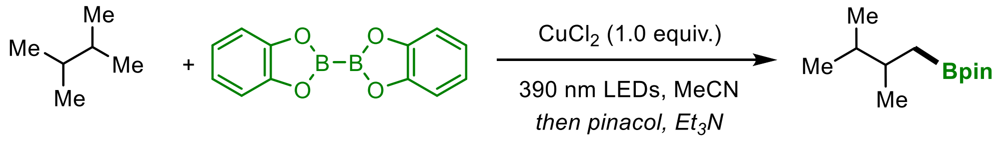

Prepared following **General Procedure B**, using 2,3-dimethylbutyl (129 mg, 195 μL, 1.50 mmol, 5.00 equiv.), $CuCl_2$ (40 mg, 0.30 mmol, 1.0 equiv.), and B_2cat_2 (71 mg, 0.30 mmol, 1.0 equiv.) in MeCN (1.5 mL, c = 0.20 M). Biotage Isolera™ flash purification on silica gel (SNAP 5 g silica cartridge), eluting with Et_2O/pentane (0 – 5%, v/v), gave **11** (40.2 mg, **63%**) as a colorless oil. The r.r. (>97:3) was determined by GC-FID analysis of the crude reaction mixture (***see spectrum***).

Prepared following **General Procedure B-prestirring**, boronic ester **11** was isolated in 56% yield (36 mg).

R*f* = 0.35 (1:10 EtOAc/pentane, CAM)

NMR Spectroscopy (*see spectra*):

^{1}H NMR (400 MHz, $CDCl_3$): δ_H 1.64 – 1.54 (m, 1H), 1.47 (m, 1H), 1.24 (s, 12H), 0.88 – 0.76 (m, 10H), 0.60 (dd, J = 15.2, 9.8 Hz, 1H) ppm;

^{13}C NMR (101 MHz, $CDCl_3$): δ_C 83.0, 35.3, 34.4, 25.1, 24.9, 19.9, 18.8, 18.7 ppm. The carbon attached to boron was not observed due to quadrupolar relaxation.

All recorded spectroscopic data matched those previously reported in the literature.[11]

2-(2,4-Dimethylpentyl)-4,4,5,5-tetramethyl-1,3,2-dioxaborolane (12)

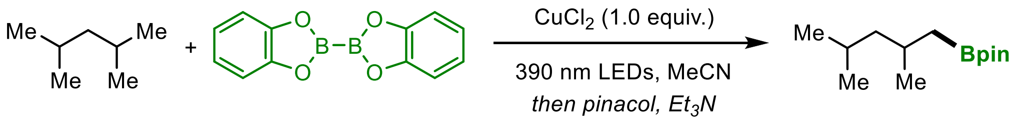

Prepared following **General Procedure B**, using 2,4-dimethylpentane (150 mg, 215 μL, 1.50 mmol, 5.00 equiv.), $CuCl_2$ (40 mg, 0.30 mmol, 1.0 equiv.) and B_2cat_2 (71 mg, 0.30 mmol, 1.0 equiv.) in MeCN (1.5 mL, c = 0.20 M). Biotage Isolera™ flash purification on silica gel (SNAP 5 g silica cartridge), eluting with Et_2O/pentane (0 – 5%, v/v), gave **12** (43.2 mg, 64%) as a colorless oil. The r.r.(>97:3) was determined by GC-FID analysis of the crude reaction mixture (***see spectrum***).

Prepared following **General Procedure B-prestirring**, boronic ester **12** was isolated in **74%** yield (50.1 mg).

R*f* = 0.35 (1:10 EtOAc/pentane, CAM)

NMR Spectroscopy (*see spectra*):

^{1}H NMR (400 MHz, $CDCl_3$): δ_H 1.76 (m, 1H), 1.61 (m, 1H), 1.24 (s, 12H), 1.14 – 0.97 (m, 2H), 0.88 (d, J = 6.6 Hz, 3H), 0.85 (d, J = 2.6 Hz, 3H), 0.84 (d, J = 2.6 Hz, 3H)., 0.79 (dd, J = 15.2, 6.0 Hz, 1H), 0.62 (dd, J

= 15.2, 8.3 Hz, 1H) ppm;

^{13}C NMR (101 MHz, $CDCl_3$): δ_C 82.9, 49.5, 27.2, 25.6, 25.0, 25.950 23.4, 22.6 ppm. The carbon attached to boron was not observed due to quadrupolar relaxation.

All recorded spectroscopic data matched those previously reported in the literature.[3]

4,4,5,5-Tetramethyl-2-(2,4,4-trimethylpentyl)-1,3,2-dioxaborolane (13a) and 4,4,5,5-tetramethyl-2-(2,2,4-trimethylpentyl)-1,3,2-dioxaborolane (13b)

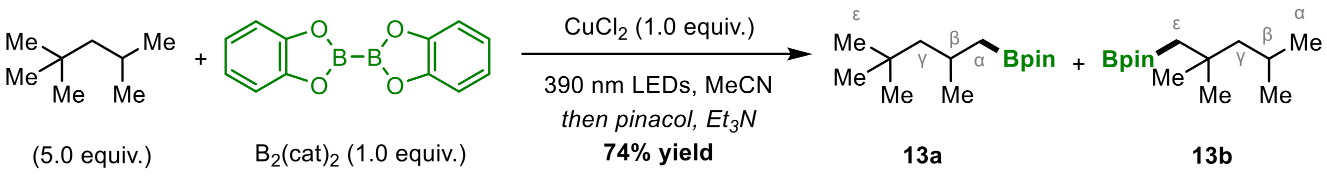

Prepared following **General Procedure B**, using 2,2,4-trimethylpentane (171 mg, 247 μL, 1.50 mmol, 5.00 equiv.), $CuCl_2$ (40 mg, 0.30 mmol, 1.0 equiv.) and B_2cat_2 (71 mg, 0.30 mmol, 1.0 equiv.) in MeCN (1.5 mL, *c* = 0.20 M). Biotage Isolera™ flash purification on silica gel (SNAP 5 g silica cartridge), eluting with Et_2O/pentane (0 – 5%, v/v), gave a mixture of **13a** and **13b** (29.2 mg, 41%) as a colorless oil. The r.r. (α:β:γ:ε = 53:<1:<1:48) was determined by ^{1}H NMR analysis of the purified product.

Prepared following **General Procedure B-prestirring**, boronic ester **13** was isolated in **74%** yield (53.0 mg).

R_f = 0.35 (1:10 EtOAc/pentane, CAM)

NMR Spectroscopy of 13a (*see spectra*):

^{1}H NMR (400 MHz, $CDCl_3$): δ_H 1.83 – 1.78 (m, 1H), 1.23 (s, 12H), 1.20 – 1.17 (m, 1H), 1.11 (dd, *J* = 13.8, 6.8 Hz, 1H), 0.95 (d, *J* = 7.0 Hz, 3H), 0.89 (s, 9H), 0.82 (dd, *J* = 15.2, 6.0 Hz, 1H), 0.69 (dd, *J* = 15.2, 8.2 Hz, 1H) ppm;

^{13}C NMR (101 MHz, $CDCl_3$): δ_C 82.9, 53.9, 31.3, 30.4, 26.2, 25.3, 25.0, 24.9 ppm. The carbon attached to boron was not observed due to quadrupolar relaxation.

All recorded spectroscopic data matched those previously reported in the literature[3].

NMR Spectroscopy of 13b (*see spectra*):

^{1}H NMR (400 MHz, $CDCl_3$): δ_H 1.70 – 1.60 (m, 1H), 1.23 (s, 12H), 1.20 – 1.17 (m, 2H), 1.11 (dd, *J* = 13.8, 6.8 Hz, 1H), 0.94 (s, 6H), 0.89 (d, *J* = 6.2 Hz, 6H), 0.80 (s, 2H) ppm;

^{13}C NMR (101 MHz, $CDCl_3$): δ_C 82.8, 53.8, 33.1, 30.1, 25.6, 24.9, 24.6 ppm. The carbon attached to boron was not observed due to quadrupolar relaxation.

All recorded spectroscopic data matched those previously reported in the literature.[3]

4,4,5,5-Tetramethyl-2-(2,2,4,4-tetramethylpentyl)-1,3,2-dioxaborolane (14)

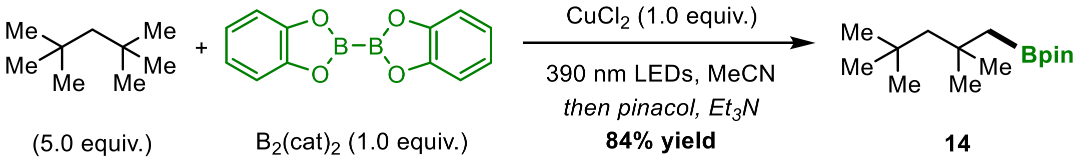

Prepared following **General Procedure B**, using 2,2,4,4-tetramethylpentane (193 mg, 268 μL, 1.50 mmol, 5.00 equiv.), $CuCl_2$ (40 mg, 0.30 mmol, 1.0 equiv.) and B_2cat_2 (71 mg, 0.30 mmol, 1.0 equiv.) in MeCN (1.5 mL, *c* = 0.20 M). Biotage Isolera™ flash purification on silica gel (SNAP 5 g silica cartridge), eluting with Et_2O/pentane (0 – 5%, v/v), gave **13** (40.3 mg, 52%) as a colorless oil. The r.r. (>97:3) was determined by GC-FID analysis of the crude reaction mixture (***see spectrum***).

Prepared following **General Procedure B-prestirring**, boronic ester **14** was isolated in **84%** yield (64.3 mg).

R*f* = 0.30 (1:10 EtOAc/pentane, CAM)

NMR Spectroscopy (*see spectra*):

^{1}**H NMR** (400 MHz, $CDCl_3$): δ_H 1.33 (s, 2H), 1.24 (s, 12H), 1.06 (s, 6H), 0.97 (s, 9H), 0.89 (s, 2H) ppm;

^{13}**C NMR** (101 MHz, $CDCl_3$): δ_C 82.8, 56.8, 34.4, 32.5, 32.1, 31.7, 25.0 ppm. The carbon attached to boron was not observed due to quadrupolar relaxation.

All recorded spectroscopic data matched those previously reported in the literature.[3]

2-(3-Chloro-2,2-dimethylpropyl)-4,4,5,5-tetramethyl-1,3,2-dioxaborolane (15)

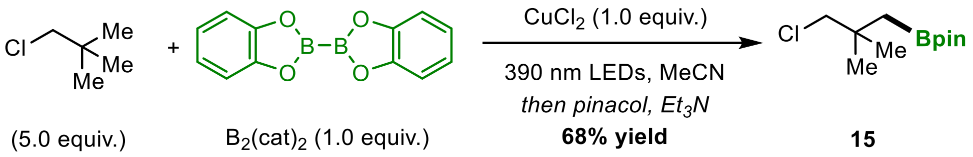

Prepared following **General Procedure B**, using 1-chloro-2,2-dimethylpropane (160 mg, 185 μL, 1.50 mmol, 5.00 equiv.), $CuCl_2$ (40 mg, 0.30 mmol, 1.0 equiv.) and B_2cat_2 (71 mg, 0.30 mmol, 1.0 equiv.) in MeCN (1.5 mL, *c* = 0.20 M). Biotage Isolera™ flash purification on silica gel (SNAP 5 g silica cartridge), eluting with Et_2O/pentane (0 – 5%, v/v), gave **15** (31.9 mg, 46%) as a colorless oil. The r.r. (>97:3) was determined by GC-FID analysis of the crude reaction mixture (***see spectrum***).

Prepared following **General Procedure B-prestirring**, boronic ester **15** was isolated in **68%** yield (47.1 mg).

R*f* = 0.30 (1:10 EtOAc/pentane, CAM)

NMR Spectroscopy (*see spectra*):

^{1}**H NMR** (400 MHz, $CDCl_3$): δ_H 3.44 (s, 2H), 1.24 (s, 12H), 1.05 (s, 6H), 0.91 (s, 2H) ppm;

^{13}**C NMR** (101 MHz, $CDCl_3$): δ_C 83.1, 57.8, 34.6, 27.2, 25.0 ppm. The carbon attached to boron was not observed due to quadrupolar relaxation.

All recorded spectroscopic data matched those previously reported in the literature.[3]

2-(3-Bromo-2,2-dimethylpropyl)-4,4,5,5-tetramethyl-1,3,2-dioxaborolane (16)

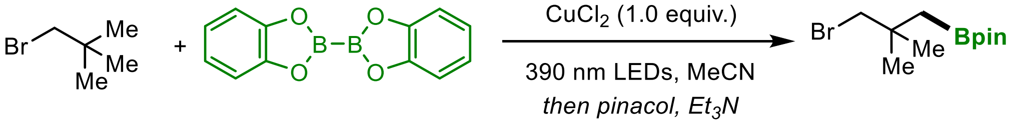

(5.0 equiv.) B$_2$(cat)$_2$ (1.0 equiv.) **16**

Prepared following **General Procedure B**, using 1-bromo-2,2-dimethylpropane (226 mg, 188 μL, 1.50 mmol, 5.00 equiv.), $CuCl_2$ (40 mg, 0.30 mmol, 1.0 equiv.) and B_2cat_2 (71 mg, 0.30 mmol, 1.0 equiv.) in MeCN (1.5 mL, *c* = 0.20 M). Biotage Isolera™ flash purification on silica gel (SNAP 5 g silica cartridge), eluting with Et_2O/pentane (0 – 5%, v/v), gave **15** (45.3 mg, 55%) as a colorless oil. The r.r. (>97:3) was determined by GC-FID analysis of the crude reaction mixture (***see spectrum***).

Prepared following **General Procedure B-prestirring**, boronic ester **16** was isolated in **60%** yield (49.1 mg).

$\mathbf{R}_f$ = 0.30 (1:10 EtOAc/pentane, CAM)

NMR Spectroscopy (*see spectra*):

^{1}**H NMR** (400 MHz, $CDCl_3$): δ_H 3.41 (s, 2H), 1.24 (s, 12H), 1.09 (s, 6H), 0.96 (s, 2H) ppm;

^{13}**C NMR** (101 MHz, $CDCl_3$): δ_C 83.2, 49.2, 33.9, 27.9, 25.0 ppm. The carbon attached to boron was not observed due to quadrupolar relaxation.

All recorded spectroscopic data matched those previously reported in the literature.[3]

2-(4-Chloro-2-methylbutyl)-4,4,5,5-tetramethyl-1,3,2-dioxaborolane (17)

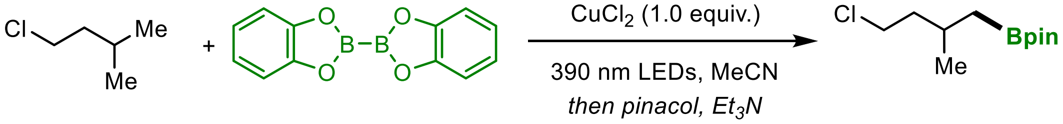

(5.0 equiv.) B$_2$(cat)$_2$ (1.0 equiv.) **17**

Prepared following **General Procedure B**, using 1-chloro-3-methylbutane (159 mg, 180 μL, 1.50 mmol, 5.00 equiv.), $CuCl_2$ (40 mg, 0.30 mmol, 1.0 equiv.) and B_2cat_2 (71 mg, 0.30 mmol, 1.0 equiv.) in MeCN (1.5 mL, *c* = 0.20 M). Biotage Isolera™ flash purification on silica gel (SNAP 5 g silica cartridge), eluting with Et_2O/pentane (0 – 5%, v/v), gave **17** (48.1 mg, 69%) as a colorless oil. The r.r. (>97:3) was determined by GC-FID analysis of the crude reaction mixture (***see spectrum***).

Prepared following **General Procedure B-prestirring**, boronic ester **17** was isolated in **74%** yield (51.7 mg).

$\mathbf{R}_f$ = 0.30 (1:10 EtOAc/pentane, CAM)

NMR Spectroscopy (*see spectra*):

^{1}H NMR (400 MHz, $CDCl_3$): δ_H 3.54 (ddt, J = 10.8, 7.2, 3.4 Hz, 2H), 1.98 – 1.85 (m, 1H), 1.81 – 1.71 (m, 1H), 1.66 (m, 1H), 1.24 (s, 12H), 0.94 (d, J = 6.6 Hz, 3H), 0.85 (dd, J = 15.4, 5.7 Hz, 1H), 0.69 (dd, J = 15.4, 8.3 Hz, 1H) ppm;

^{13}C NMR (101 MHz, $CDCl_3$): δ_C 83.2, 43.5, 42.2, 27.3, 25.0, 24.9, 21.1 ppm. The carbon attached to boron was not observed due to quadrupolar relaxation.

^{11}B NMR (128 MHz, $CDCl_3$): δ_B 33.8 (brs, 1B) ppm.

All recorded spectroscopic data matched those previously reported in the literature.[3]

2-(4-Bromo-2-methylbutyl)-4,4,5,5-tetramethyl-1,3,2-dioxaborolane (18)

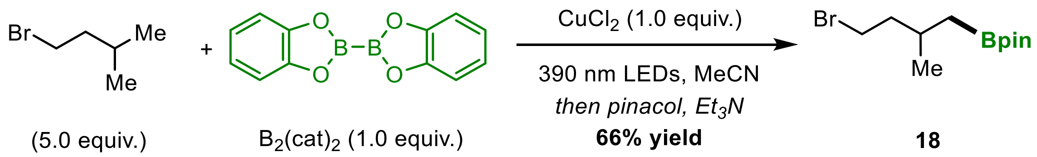

Prepared following **General Procedure B**, using 1-bromo-3-methylbutane (225 mg, 179 μL, 1.50 mmol, 5.00 equiv.), $CuCl_2$ (40 mg, 0.30 mmol, 1.0 equiv.) and B_2cat_2 (71 mg, 0.30 mmol, 1.0 equiv.) in MeCN (1.5 mL, c = 0.20 M). Biotage Isolera™ flash purification on silica gel (SNAP 5 g silica cartridge), eluting with Et_2O/pentane (0 – 5%, v/v), gave **18** (48.2 mg, 58%) as a colorless oil. The r.r. (>97:3) was determined by GC-FID analysis of the crude reaction mixture (***see spectrum***).

Prepared following **General Procedure B-prestirring**, boronic ester **18** was isolated in **66%** yield (55.4 mg).

R_f = 0.25 (1:10 EtOAc/pentane, CAM)

NMR Spectroscopy (*see spectra*):

^{1}H NMR (400 MHz, $CDCl_3$): δ_H 3.42 (ddt, J = 10.8, 7.2, 3.4 Hz, 2H), 1.96 – 1.78 (m, 2H), 1.80 – 1.68 (m, 1H), 1.24 (s, 12H), 0.94 (d, J = 6.6 Hz, 3H), 0.85 (dd, J = 15.4, 5.8 Hz, 1H), 0.69 (dd, J = 15.4, 8.0 Hz, 1H) ppm;

^{13}C NMR (101 MHz, $CDCl_3$): δ_C 83.2, 42.4, 32.3, 28.6, 25.0, 24.9, 21.8 ppm. The carbon attached to boron was not observed due to quadrupolar relaxation.

^{11}B NMR (128 MHz, $CDCl_3$): δ_B 33.9 (brs, 1B) ppm.

IR (film): ν_{max} 2925, 2855, 1375, 1325, 1280, 1261, 1151, 767, 757 cm^{-1}.

HRMS (APCI$^+$): m/z calc'd for $C_{11}H_{22}O_2BBr$ $[M+H]^+$, 277.0969; found, 277.0988.

2-(5-Bromo-2-methylpentyl)-4,4,5,5-tetramethyl-1,3,2-dioxaborolane (19)

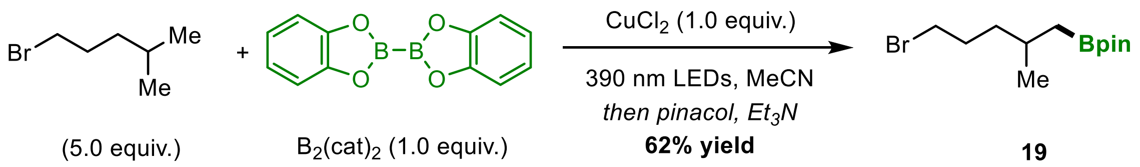

Prepared following **General Procedure B**, using 1-bromo-4-methylpentane (248 mg, 219 µL, 1.50 mmol, 5.00 equiv.), $CuCl_2$ (40 mg, 0.30 mmol, 1.0 equiv.) and B_2cat_2 (71 mg, 0.30 mmol, 1.0 equiv.) in MeCN (1.5 mL, *c* = 0.20 M). Biotage Isolera™ flash purification on silica gel (SNAP 5 g silica cartridge), eluting with Et_2O/pentane (0 – 5%, v/v), gave **19** (38.5 mg, 44%) as a colorless oil. The r.r. (>97:3) was determined by GC-FID analysis of the crude reaction mixture (***see spectrum***).

Prepared following **General Procedure B-prestirring**, boronic ester **19** was isolated in **62%** yield (54.6 mg, 0.17 mmol).

When the reaction was performed using **3.0 equivalents** of 1-bromo-4-methylpentane (149 mg, 131 µL, 0.900 mmol, 3.00 equiv.), boronic ester **19** was isolated in 34% yield (29.6 mg).

$\mathbf{R_f}$ = 0.30 (1:10 EtOAc/pentane, CAM)

NMR Spectroscopy (*see spectra*):

^{1}**H NMR** (400 MHz, $CDCl_3$): δ_H 3.38 (t, *J* = 7.0 Hz, 2H), 1.94 – 1.78 (m, 2H), 1.78 – 1.66 (m, 1H), 1.44 – 1.30 (m, 2H), 1.24 (s, 12H), 0.92 (d, *J* = 6.8 Hz, 3H), 0.83 (dd, *J* = 15.4, 6.0, 5.8 Hz, 1H), 0.67 (dd, *J* = 15.4, 8.2 Hz, 1H) ppm;

^{13}**C NMR** (101 MHz, $CDCl_3$): δ_C 83.1, 38.1, 34.4, 31.0, 29.1, 25.0, 24.9, 22.4 ppm. The carbon attached to boron was not observed due to quadrupolar relaxation.

All recorded spectroscopic data matched those previously reported in the literature.[3]

2-(4-Bromobutyl)-4,4,5,5-tetramethyl-1,3,2-dioxaborolane (20a) and 2-(4-bromobutan-2-yl)-4,4,5,5-tetra-methyl-1,3,2-dioxaborolane (20b)

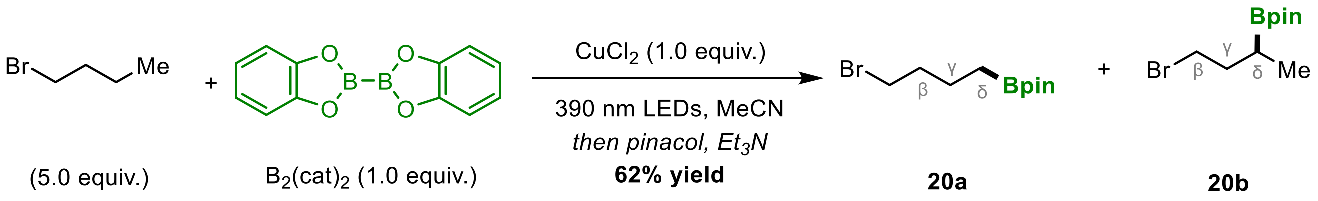

Prepared following **General Procedure B**, using 1-bromobutane (206 mg, 162 µL, 1.50 mmol, 5.00 equiv.), $CuCl_2$ (40 mg, 0.30 mmol, 1.0 equiv.) and B_2cat_2 (71 mg, 0.30 mmol, 1.0 equiv.) in MeCN (1.5 mL, *c* = 0.20 M). Biotage Isolera™ flash purification on silica gel (SNAP 5 g silica cartridge), eluting with Et_2O/pentane (0 – 5%, v/v), gave a mixture of **20a** and **20b** (34 mg, 44%) as a colorless oil. The r.r. (γ:δ = 32:68, **20b:20a**) was determined by GC-FID analysis of the crude reaction mixture (***see spectrum***).

Prepared following **General Procedure B-prestirring**, boronic ester **20** was isolated in **62%** yield (48.1 mg,

0.17 mmol).

R*f* = 0.20 (1:10 EtOAc/pentane, CAM)

NMR Spectroscopy of 20a (*see spectra*):

[1]**H NMR** (400 MHz, $CDCl_3$): δ_H 3.40 (t, *J* = 7.0 Hz, 2H), 1.87 (m, 2H), 1.60 – 1.49 (m, 2H), 1.24 (s, 12H), 0.80 (t, *J* = 8.0 Hz, 2H) ppm;

[13]**C NMR** (101 MHz, $CDCl_3$): δ_C 83.2, 35.5, 33.8, 25.0, 22.9 ppm. The carbon attached to boron was not observed due to quadrupolar relaxation.

All recorded spectroscopic data matched those previously reported in the literature.[12]

NMR Spectroscopy of 20b (*see spectra*):

[1]**H NMR** (400 MHz, $CDCl_3$): δ_H 3.46 (t, *J* = 7.2 Hz, 2H), 2.04 (dt, *J* = 14.2, 7.6 Hz, 1H), 1.82 (dt, *J* = 14.2, 7.2 Hz, 1H), 1.24 (s, 13H), 0.99 (d, *J* = 7.4 Hz, 3H) ppm;

[13]**C NMR** (101 MHz, $CDCl_3$): δ_C 83.3, 36.3, 33.6, 24.9, 24.9, 15.0 ppm;

[11]**B NMR** (128 MHz, $CDCl_3$): δ_B 33.6 (brs, 1B) ppm.

IR (film): ν_{max} 2920, 2855, 1280, 1261, 767 cm^{-1}.

HRMS (EI^+): m/z calc'd for $C_{10}H_{20}O_2BrB$ $[M–Me]^+$, 247.0499; found, 247.0500.

4,4,5,5-Tetramethyl-2-(6,6,6-trifluorohexyl)-1,3,2-dioxaborolane (21a) and 4,4,5,5-tetramethyl-2-(6,6,6-trifluorohexan-2-yl)-1,3,2-dioxaborolane (21b)

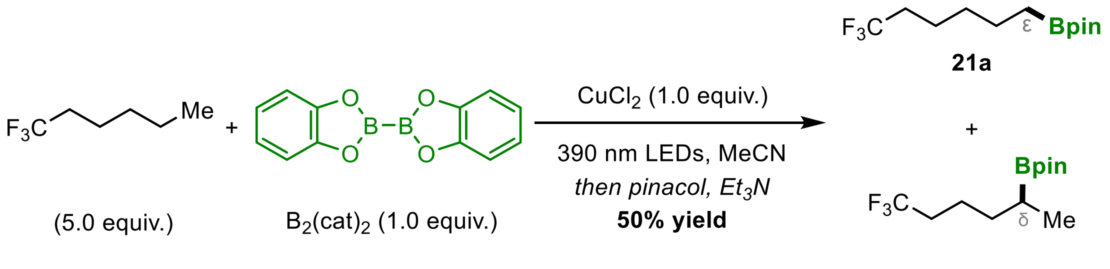

Prepared following **General Procedure B**, using 1,1,1-trifluorohexane (210 mg, 1.50 mmol, 5.00 equiv.), $CuCl_2$ (40 mg, 0.30 mmol, 1.0 equiv.) and B_2cat_2 (71 mg, 0.30 mmol, 1.0 equiv.) in MeCN (1.5 mL, *c* = 0.20 M). Biotage Isolera™ flash purification on silica gel (SNAP 5 g silica cartridge), eluting with Et_2O/pentane (0 – 5%, v/v), gave a mixture of **21a**, **21b** and other isomer (34.4 mg, 43%) as a colorless oil. The r.r. (γ:δ:ε = 29:34:37) was determined by GC-FID analysis of the crude reaction mixture (*see spectrum*).

Prepared following **General Procedure B-prestirring**, boronic ester **21** was isolated in **50%** yield (39.9 mg).

R*f* = 0.40 (1:10 EtOAc/pentane, CAM)

NMR Spectroscopy (*see spectra*):

^{1}**H NMR** (400 MHz, $CDCl_3$) (isomer **21a**+**21b**): δ_H 2.13 – 1.96 (m, 2H), 1.65 – 1.30 (m, 5H), 1.23 + 1.24 (2s, 12H), 1.01 – 0.96 (m, 1H), 0.78 (t, J = 7.5 Hz, 1H) ppm;

^{13}**C NMR** (101 MHz, $CDCl_3$) (isomer **21a**): δ_C 127.5 (q, J = 276.4 Hz), 83.1, 33.8 (p, J = 28.4 Hz), 31.5, 25.0, 23.7, 21.8 (q, J = 2.9 Hz) ppm; The carbon attached to boron was not observed due to quadrupolar relaxation.

^{13}**C NMR** (101 MHz, $CDCl_3$) (isomer **21b**): δ_C 127.5 (q, J = 276.4 Hz), 83.2, 33.8 (p, J = 28.4 Hz), 24.9, 24.8, 21.3 (q, J = 2.9 Hz), 15.4 ppm. The carbon attached to boron was not observed due to quadrupolar relaxation.

^{19}**F NMR** (377 MHz, $CDCl_3$) (isomer **21a**): δ_F – 66.43 (t, J = 11.0 Hz, CF_3);

^{19}**F NMR** (377 MHz, $CDCl_3$) (isomer **21b**): δ_F– 66.48 (t, J = 11.0 Hz, CF_3);

^{11}**B NMR** (128 MHz, $CDCl_3$) (isomer **21a**+**21b**): δ_B 33.7 (brs) ppm.

IR (film): ν_{max} 2950, 2924, 1773, 1731, 1276, 1261, 763, 750 cm^{-1}.

HRMS (EI^+): m/z calc'd for $C_{12}H_{22}O_2BF_3$ $[M–Me]^+$, 251.1425; found, 251.1425.

2-(2,2-Dimethylpent-4-en-1-yl)-4,4,5,5-tetramethyl-1,3,2-dioxaborolane (22)

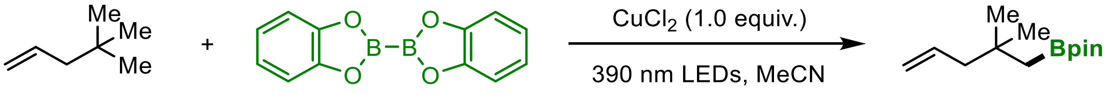

(5.0 equiv.) $B_2(cat)_2$ (1.0 equiv.) **22**

Prepared following **General Procedure B**, using 4,4-dimethylpent-1-ene (147 mg, 216 µL, 1.50 mmol, 5.00 equiv.), $CuCl_2$ (40 mg, 0.30 mmol, 1.0 equiv.), and B_2cat_2 (71 mg, 0.30 mmol, 1.0 equiv.) in MeCN (3.0 mL, c = 0.20 M). Flash column chromatography, eluting with Et_2O/pentane (0 – 14%, v/v) gave **22** (7.0 mg, 10%) as a colorless oil. The r.r. (>97:3) was determined by GC-FID analysis of the crude reaction mixture (***see spectrum***).

Prepared following **General Procedure B-prestirring**, boronic ester **22** was isolated in **20%** yield (14 mg).

R*f* =0.40 (1:19 Et_2O/pentane, CAM)

NMR Spectroscopy (*see spectra*):

^{1}**H NMR** (400 MHz, $CDCl_3$) δ_H 5.84 (ddt, J = 16.8, 10.4, 7.6 Hz, 1H), 5.03 – 4.95 (m, 2H), 2.02 (dt, J = 7.6, 1.2 Hz, 2H), 1.24 (s, 12H), 0.96 (s, 6H), 0.79 (s, 2H) ppm;

^{13}**C NMR** (101 MHz, $CDCl_3$) δ_C 136.29, 116.56, 82.73, 49.00, 32.47, 29.21, 24.87 ppm. The carbon attached to boron was not observed due to quadrupolar relaxation.

All recorded spectroscopic data matched those previously reported in the literature.[3]

2-(2-(3,5-Di-*tert*-butylphenyl)-2-methylpropyl)-4,4,5,5-tetramethyl-1,3,2-dioxaborolane (23)

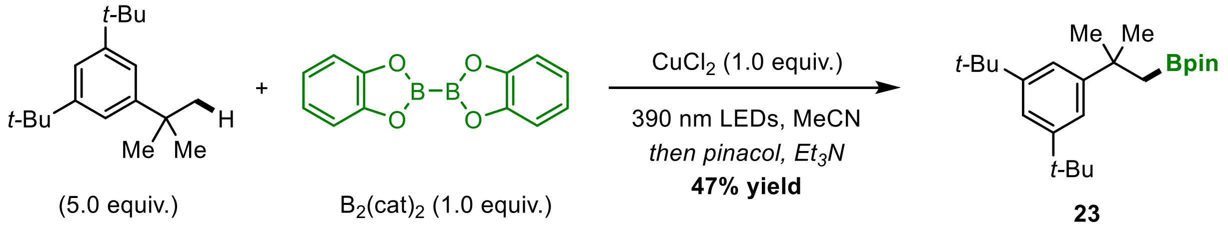

Prepared following **General Procedure A**, using 1,3,5-tri-*tert*-butylbenzene (370 mg, 1.50 mmol, 5.00 equiv.), $CuCl_2$•$2H_2O$ (10 mg, 0.060 mmol, 0.20 equiv.) and B_2cat_2 (71 mg, 0.30 mmol, 1.0 equiv.) in MeCN (3.0 mL, *c* = 0.10 M). Flash column chromatography, eluting with Et_2O/pentane (0 – 5%, v/v) gave **23** (48 mg, 43%) as a colorless oil. Unreacted 1,3,5-tri-*tert*-butylbenzene was recovered in 98% yield (331 mg).

Prepared following **General Procedure B-prestirring**, boronic ester **23** was isolated in **47%** yield (52 mg).

When the reaction was performed using **3.0 equivalents** of 1,3,5-tri-*tert*-butylbenzene (222 mg, 0.900 mmol), boronic ester **23** was isolated in 41% yield (46 mg). Unreacted 1,3,5-tri-tert-butylbenzene was recovered in 99% yield (189 mg).

When the reaction was performed using **1.0 equivalent** of 1,3,5-tri-*tert*-butylbenzene (74 mg, 0.30 mmol, 1.0 equiv.), $CuCl_2$ (80 mg, 0.60 mmol, 2.0 equiv.), and B_2cat_2 (143 mg, 0.600 mmol, 2.00 equiv.) in MeCN (1.5 mL, *c* = 0.2 M), boronic ester **23** was isolated in **39%** yield (44 mg). Unreacted alkane was recovered in 43% yield (20 mg).

R_f = 0.38 (5:95 Et_2O/pentane, UV, CAM)

NMR Spectroscopy (*see spectra*):

^{1}**H NMR** (400 MHz, $CDCl_3$) δ_H 7.27 (d, *J* = 1.8 Hz, 2H), 7.22 (t, *J* = 1.8 Hz, 1H), 1.44 (s, 6H), 1.34 (s, 18H), 1.29 (s, 2H), 1.09 (s, 12H) ppm;

^{13}**C NMR** (101 MHz, $CDCl_3$) δ_C 150.5, 149.7, 119.9, 119.3, 82.8, 36.7, 35.1, 31.8, 31.6, 24.8 ppm. The carbon attached to boron was not observed due to quadrupolar relaxation.

^{11}**B NMR** (128 MHz, $CDCl_3$) δ_B 33.1 ppm.

IR (film): 2958, 2867, 1596, 1469, 1379, 1355, 1322, 1249, 1227, 1144, 971, 898, 873, 847, 714 cm^{-1}.

HRMS (EI^+): m/z calculated for $C_{24}H_{41}BO_2$ $[M]^+$, 372.3194; found, 372.3190.

2-(2-(3,5-Dibromophenyl)-2-methylpropyl)-4,4,5,5-tetramethyl-1,3,2-dioxaborolane (24)

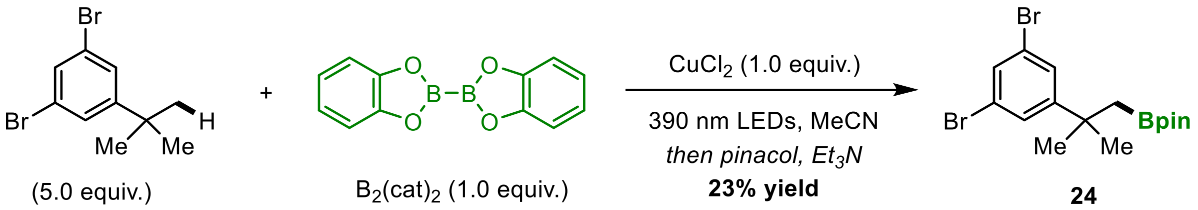

Prepared following **General Procedure B**, 1,3-dibromo-5-(tert-butyl)benzene (435 mg, 1.50 mmol, 5.00 equiv.), $CuCl_2$ (40 mg, 0.30 mmol, 1.0 equiv.), and B_2cat_2 (71 mg, 0.30 mmol, 1.0 equiv.) in MeCN (1.5 mL, *c* = 0.20 M). Flash column chromatography, eluting with EtOAc/hexane (0 – 5%, v/v) gave **24** (28 mg, **23%**) as a colorless oil.

Prepared following **General Procedure B-prestirring**, boronic ester **24** was isolated in 22% yield (27.6 mg).

R_f = 0.43 (1:10 EtOAc/hexane, CAM)

NMR Spectroscopy (*see spectra*):

^{1}H NMR (400 MHz, $CDCl_3$) δ_H 7.45 (s, 3H), 1.37 (s, 6H), 1.19 (s, 2H), 1.13 (s, 12H) ppm;

^{13}C NMR (101 MHz, $CDCl_3$) δ_C 155.8, 131.0, 128.1, 122.7, 83.1, 36.8, 31.2, 24.9 ppm. The carbon attached to boron was not observed due to quadrupolar relaxation.

^{11}B NMR (128 MHz, $CDCl_3$) δ_B 32.9 ppm.

IR (film): 2973, 2929, 1582, 1550, 1468, 1425, 1407, 1379, 1356, 1325, 1271, 1226, 1165, 1143, 971, 872, 849, 739, 687 cm^{-1}.

HRMS (EI$^+$): m/z calculated for $C_{16}H_{23}O_2BBr_2$ [M]$^+$, 416.0152; found, 416.0153.

4,4,5,5-Tetramethyl-2-((1,4,4-trimethyl-1,2,3,4-tetrahydronaphthalen-1-yl)methyl)-1,3,2-dioxaborolane (25)

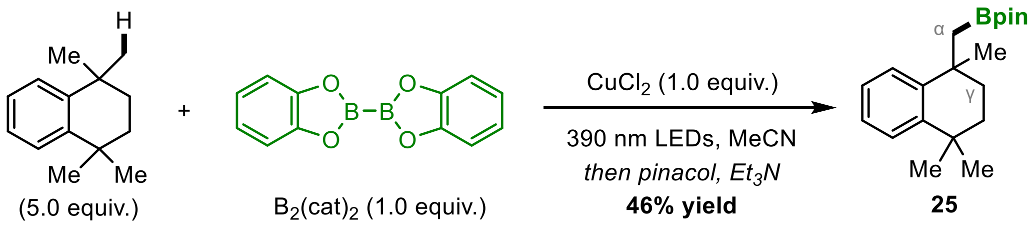

Prepared following **General Procedure B**, 1,1,4,4-tetramethyl-1,2,3,4-tetrahydronaphthalene (282 mg, 314μL 1.50 mmol, 5.00 equiv.), $CuCl_2$ (40 mg, 0.30 mmol, 1.0 equiv.), and B_2cat_2 (71 mg, 0.30 mmol, 1.0 equiv.) in MeCN (1.5 mL, *c* = 0.20 M). Flash column chromatography, eluting with Et_2O/pentane (0 – 5%, v/v) gave **25** (39 mg, 41%) as a colorless oil. The r.r. (α:γ = 93:7) was determined by GC-FID analysis of the crude reaction mixture (*see spectrum*). Only the major product (α isomer) is listed for characterisation.

Prepared following **General Procedure B-prestirring**, boronic ester **25** was isolated in **48%** yield (45 mg)

R*f* = 0.40 (1:19 Et_2O/pentane, CAM)

NMR Spectroscopy (*see spectra*):

^{1}H NMR (400 MHz, $CDCl_3$) δ_H 7.32 – 7.23 (m, 2H), 7.11 – 7.04 (m, 2H), 2.23 – 2.14 (m, 1H), 1.80 – 1.71 (td, *J* = 3.0, 10.2 Hz 1H), 1.65 – 1.52 (m, 2H), 1.41 (d, *J* = 15.0 Hz, 1H), 1.34 (s, 3H), 1.29 (s, 6H), 1.15 (d, *J* = 15.0 Hz, 1H), 1.08 (s, 6H), 1.02 (s, 6H) ppm ;

^{13}C NMR (101 MHz, $CDCl_3$) δ_C 145.6, 144.9, 127.0, 126.4, 125.5, 125.5, 82.8, 36.1, 35.4, 34.3, 33.9, 33.16, 32.1, 31.7, 24.8, 24.7 ppm. The carbon attached to boron was not observed due to quadrupolar relaxation.

All recorded spectroscopic data matched those previously reported in the literature.[3]

2-(2-(3,5-Diisopropylphenyl)propyl)-4,4,5,5-tetramethyl-1,3,2-dioxaborolane (26)

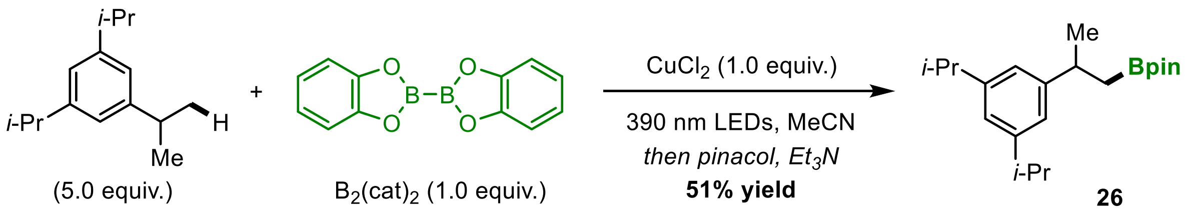

Prepared following **General Procedure B**, using 1,3,5-triisopropylbenzene (307 mg, 361 µL, 1.50 mmol, 5.00 equiv.), $CuCl_2$ (40 mg, 0.30 mmol, 1.0 equiv.), and B_2cat_2 (71 mg, 0.30 mmol, 1.0 equiv.) in MeCN (3.0 mL, *c* = 0.10 M). Flash column chromatography, eluting with Et_2O/pentane (0 – 3%, v/v) gave **26** (21 mg, 21%) as a colorless oil. The r.r. (>97:3) was determined by GC-FID analysis of the crude reaction mixture (***see spectrum***).

Prepared following **General Procedure B-prestirring**, boronic ester **26** was isolated in **51%** yield (50.5 mg).

R*f* = 0.27 (1:19 Et_2O/pentane, UV, CAM)

NMR Spectroscopy (*see spectra*):

^{1}H NMR (400 MHz, $CDCl_3$) δ_H 6.92 (d, *J* = 1.8 Hz, 2H), 6.87 (t, *J* = 1.8 Hz, 1H), 3.05 – 2.94 (hex, *J* = 7.0 Hz, 1H), 2.85 (hept, *J* = 6.8 Hz, 2H), 1.28 (m, 2H), 1.26 (d, *J* = 7.0 Hz, 3H), 1.24 (d, *J* = 6.8 Hz, 6H), 1.23 (d, *J* = 6.8 Hz, 6H), 1.16 (s, 6H), 1.15 (s, 6H) ppm;

^{13}C NMR (101 MHz, $CDCl_3$) δ_C 149.2, 148.6, 122.4, 122.1, 83.0, 36.1, 34.4, 25.2, 24.9, 24.8, 24.3, 24.3 ppm. The carbon attached to boron was not observed due to quadrupolar relaxation.

All recorded spectroscopic data matched those previously reported in the literature.[13]

4-(1-(4,4,5,5-Tetramethyl-1,3,2-dioxaborolan-2-yl)propan-2-yl)benzonitrile (27)

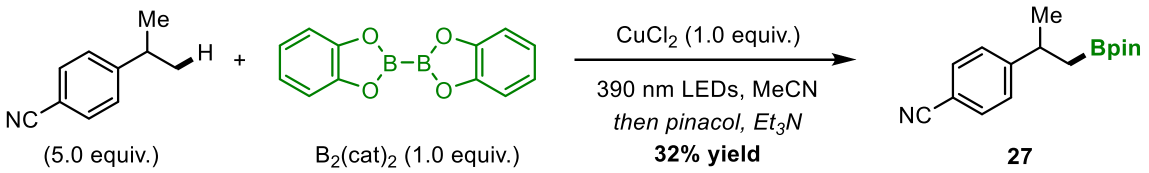

27

Prepared following **General Procedure B**, using 4-isopropylbenzonitrile (218 mg, 229 μL 1.50 mmol, 5.00 equiv.), $CuCl_2$ (40 mg, 0.30 mmol, 1.0 equiv.), and B_2cat_2 (71 mg, 0.30 mmol, 1.0 equiv.) in MeCN (3.0 mL, c = 0.10 M). Flash column chromatography, eluting with EtOAc/hexane (0 – 8%, v/v) gave **27** (26 mg, **32%**) as a colorless oil. The r.r. (>97:3) was determined by GC-FID analysis of the crude reaction mixture (***see spectrum***).

R*f* = 0.29 (1:6 EtOAc/hexane, CAM)

NMR Spectroscopy (*see spectra*):

^{1}**H NMR** (400 MHz, $CDCl_3$) δ_H 7.58 – 7.53 (m, 2H), 7.35 – 7.31 (m, 2H), 3.08 (m, 1H), 1.27 (d, J = 6.8 Hz, 3H), 1.18 – 1.12 (m, 2H), 1.16 (s, 6H), 1.14 (s, 6H), ppm;

^{13}**C NMR** (101 MHz, $CDCl_3$) δ_C 155.0, 132.3, 127.7, 119.4, 109.7, 83.4, 36.2, 24.9, 24.8, 24.6 ppm. The carbon attached to boron was not observed due to quadrupolar relaxation.

All recorded spectroscopic data matched those previously reported in the literature.[3]

4,4,5,5-Tetramethyl-2-(tetrahydrofuran-2-yl)-1,3,2-dioxaborolane (28)

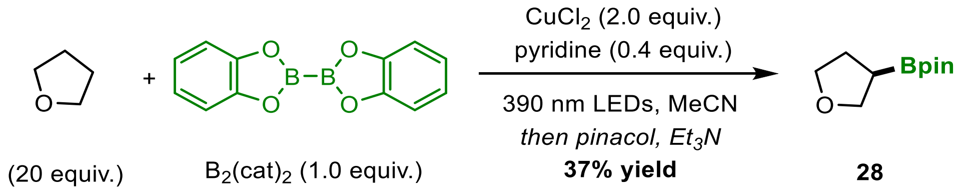

28

Prepared following **General Procedure C**, using tetrahydrofuran (432 mg, 486 μL, 6.00 mmol, 20.0 equiv.), $CuCl_2$ (80 mg, 0.60 mmol, 2.0 equiv.), pyridine (9.5 mg, 9.7 μL, 0.12 mmol, 0.40 equiv.), and B_2cat_2 (71 mg, 0.30 mmol, 1.0 equiv.) in MeCN (5.0 mL, c = 60 mM). Biotage Isolera™ flash purification on silica gel (SNAP 10 g silica cartridge), eluting with Et_2O/pentane (0 – 10%, v/v), gave **28** (22.0 mg, **37%**) as a colorless oil.

Prepared following **General Procedure B**, boronic ester **28** was isolated in **<5%** yield.

R*f* = 0.50 (1:4 EtOAc/pentane, CAM)

NMR Spectroscopy (*see spectra*):

^{1}**H NMR** (400 MHz, $CDCl_3$): δ_H 3.98 (t, J = 8.2 Hz, 1H), 3.80 (td, J = 8.0, 4.2 Hz, 1H), 3.70 (td, J = 8.0, 7.0 Hz, 1H), 3.61 (dd, J = 9.6, 8.0 Hz, 1H), 2.09 – 1.97 (m, 1H), 1.82 (ddt, J = 12.0, 10.0, 8.0 Hz, 1H), 1.66 – 1.55 (m, 1H), 1.24 (s, 12H) ppm;

^{13}**C NMR** (101 MHz, $CDCl_3$): δ_C 83.5, 70.4, 68.6, 28.9, 24.9 ppm. The carbon attached to boron was not

observed due to quadrupolar relaxation.

All recorded spectroscopic data matched those previously reported in the literature.[14]

4,4,5,5-Tetramethyl-2-(tetrahydro-2H-pyran-4-yl)-1,3,2-dioxaborolane (29a) and 4,4,5,5-tetramethyl-2-(tetra-hydro-2H-pyran-3-yl)-1,3,2-dioxaborolane (29b)

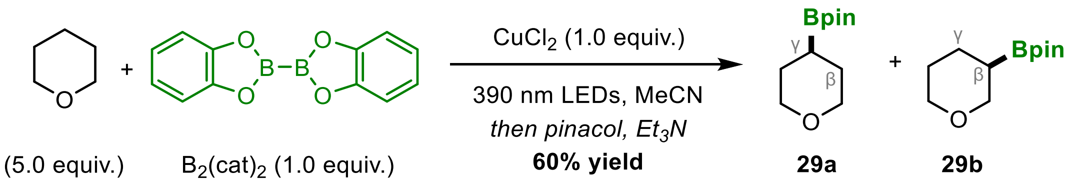

Prepared following **General Procedure B**, using tetrahydropyran (129 mg, 147 μL, 1.50 mmol, 5.00 equiv.), $CuCl_2$ (40 mg, 0.30 mmol, 1.0 equiv.) and B_2cat_2 (71 mg, 0.30 mmol, 1.0 equiv.) in MeCN (1.5 mL, *c* = 0.20 M). Biotage Isolera^{TM} flash purification on silica gel (SNAP 10 g silica cartridge), eluting with Et_2O/pentane (0 – 5%, v/v), gave **29a** and **29b** (38.3 mg, **60%**) as a colorless oil. The r.r. (β:γ = 18:82, **29b:29a**) was determined by GC-FID analysis of the crude reaction mixture (***see spectrum***).

Prepared following **General Procedure B-prestirring**, boronic ester **29** was isolated in 59% yield (36.5 mg).

Prepared following **General Procedure C**, boronic ester **29** was isolated in **32%** yield (19.6 mg).

R_f = 0.50 (1:4 EtOAc/pentane, CAM)

NMR Spectroscopy of 29a (*see spectra*):

^{1}H NMR (400 MHz, $CDCl_3$): δ_H 3.82 (dt, *J* = 11.2, 4.0 Hz, 2H), 3.60 – 3.39 (m, 2H), 1.72 – 1.46 (m, 4H), 1.27–1.17 (overlapped m, 1H), 1.24 (s, 6H), 1.22 (s, 6H) ppm;

^{13}C NMR (101 MHz, $CDCl_3$): δ_C 82.3, 70.0, 27.8, 24.9 ppm. The carbon attached to boron was not observed due to quadrupolar relaxation.

NMR Spectroscopy of 29b (*see spectra*):

^{1}H NMR (400 MHz, $CDCl_3$): δ_H 3.92 – 3.89 (m, 1H), 3.89 – 3.86 (m, 1H), 3.54 – 3.42 (m, 2H), 1.86 – 1.79 (m, 1H), 1.66 – 1.50 (m, 2H), 1.37–1.20 (m, 2H), 1.24 (overlapped s, 12H);

^{13}C NMR (101 MHz, $CDCl_3$): δ_C 82.3, 70.0, 69.8, 26.83, 25.1, 25.0, 24.9 ppm. The carbon attached to boron was not observed due to quadrupolar relaxation.

All recorded spectroscopic data matched those previously reported in the literature.[12,14]

4,4,5,5-Tetramethyl-2-((tetrahydro-2H-pyran-4-yl)methyl)-1,3,2-dioxaborolane (30)

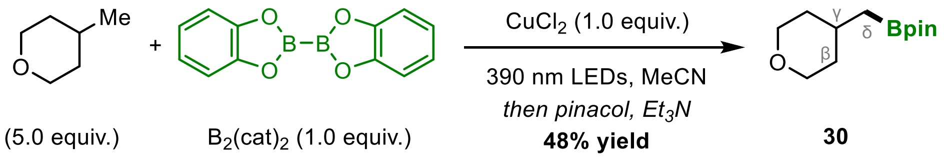

30

Prepared following **General Procedure B**, using 4-methyltetrahydropyran (150 mg, 176 µL, 1.50 mmol, 5.00 equiv.), $CuCl_2$ (40 mg, 0.30 mmol, 1.0 equiv.) and B_2cat_2 (71 mg, 0.30 mmol, 1.0 equiv.) in MeCN (1.5 mL, *c* = 0.20 M). Biotage Isolera™ flash purification on silica gel (SNAP 10 g silica cartridge), eluting with Et_2O/pentane (0 – 5%, v/v), gave **30** (27.8 mg, 41%) as a colorless oil. The r.r. (β:γ:δ = 13:<1:87) was determined by GC-FID of the purified product (***see spectrum***).

Prepared following **General Procedure B-prestirring**, boronic ester **30** was isolated in **48%** yield (32.5 mg).

R*f* = 0.45 (1:4 EtOAc/pentane, CAM)

NMR Spectroscopy (*see spectra*):

^{1}H NMR (400 MHz, $CDCl_3$): δ_H 3.91 (dtd, *J* = 11.6, 2.4, 1.2 Hz, 2H), 3.38 (td, *J* = 11.6, 2.0 Hz, 2H), 1.71 (td, *J* = 7.4, 3.6 Hz, 1H), 1.64 – 1.55 (m, 2H), 1.39 – 1.26 (m, 2H), 1.24 (s, 12H), 0.78 (d, *J* = 7.4 Hz, 2H) ppm;

^{13}C NMR (101 MHz, $CDCl_3$): δ_C 83.2, 68.4, 35.6, 31.6, 25.0, 24.9 ppm. The carbon attached to boron was not observed due to quadrupolar relaxation.

All recorded spectroscopic data matched those previously reported in the literature.[15]

2-((1*S*,2*R*,4*R*)-7-Oxabicyclo[2.2.1]heptan-2-yl)-4,4,5,5-tetramethyl-1,3,2-dioxaborolane (31)

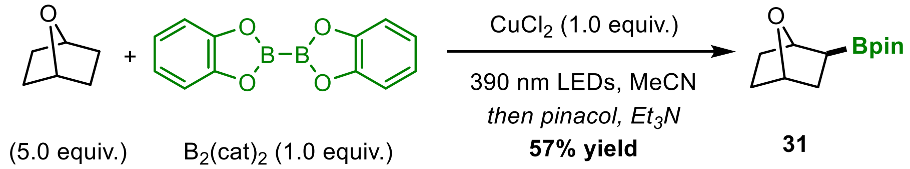

31

Prepared following **General Procedure B**, using 7-oxabicyclo[2.2.1]heptane (147 mg, 152 µL, 1.50 mmol, 5.00 equiv.), $CuCl_2$ (40 mg, 0.30 mmol, 1.0 equiv.) and B_2cat_2 (71 mg, 0.30 mmol, 1.0 equiv.) in MeCN (1.5 mL, *c* = 0.20 M). Biotage Isolera™ flash purification on silica gel (SNAP 5 g silica cartridge), eluting with Et_2O/pentane (0 – 5%, v/v), gave **31** (35.8 mg, 53%) as a colorless oil. The r.r. (>97:3) was determined by GC-FID analysis of the crude reaction mixture (***see spectrum***). The configuration was determined by key TOCSY correlation according to the similar compound in the literature.[16]

Prepared following **General Procedure B-prestirring**, boronic ester **31** was isolated in **57%** yield (38.6 mg).

Prepared following **General Procedure C**, boronic ester **31** was isolated in **57%** yield (38.4 mg).

When the reaction was performed using **3.0 equivalents** of 7-oxabicyclo[2.2.1]heptane (90 mg, 93 µL, 0.90

mmol, 3.0 equiv.), boronic ester **31** was isolated in 46% yield (30.9 mg).

R*f* = 0.50 (1:4 EtOAc/pentane, CAM)

NMR Spectroscopy (*see spectra*):

^{1}H NMR (400 MHz, $CDCl_3$): δ_H 4.62 (d, *J* = 4.2 Hz, 1H), 4.59 (t, *J* = 4.8 Hz, 1H), 1.81 – 1.66 (m, 3H), 1.66 – 1.57 (m, 1H), 1.54 – 1.39 (m, 2H), 1.24 (s, 12H), 1.16 (dd, *J* = 9.9, 6.1 Hz, 1H) ppm;

^{13}C NMR (101 MHz, $CDCl_3$): δ_C 83.4, 78.2, 76.9, 33.2, 32.7, 29.9, 24.9, 24.9 ppm. The carbon attached to boron was not observed due to quadrupolar relaxation.

^{11}B NMR (128 MHz, $CDCl_3$): δ_B 33.0 (brs, 1B) ppm.

IR (film): ν_{max} 2977, 1418, 1375, 1317, 1145, 866, 752 cm^{-1}.

HRMS (EI^+): m/z calc'd for $C_{12}H_{21}BO_3$ $[M]^+$, 224.1577; found, 224.1578.

2-(4-(Isopentyloxy)-2-methylbutyl)-4,4,5,5-tetramethyl-1,3,2-dioxaborolane (32)

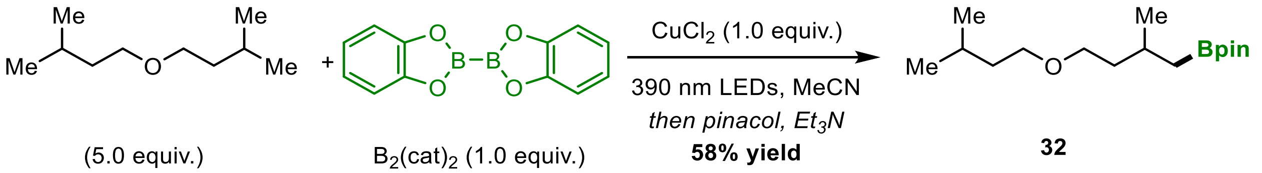

32

Prepared following **General Procedure B**, using distilled isoamyl ether (237 mg, 294 μL, 1.50 mmol, 5.00 equiv.), $CuCl_2$ (40 mg, 0.30 mmol, 1.0 equiv.) and B_2cat_2 (71 mg, 0.30 mmol, 1.0 equiv.) in MeCN (1.5 mL, *c* = 0.20 M). Biotage Isolera^{TM} flash purification on silica gel (SNAP 5 g silica cartridge), eluting with Et_2O/pentane (0 – 5%, v/v), gave **32** (33.8 mg, 40%) as a colorless oil. The r.r. (>97:3) was determined by GC-FID analysis of the crude reaction mixture (***see spectrum***).

Prepared following **General Procedure B-prestirring**, boronic ester **32** was isolated in **58%** yield (49 mg).

R*f* = 0.38 (1:4 EtOAc/pentane, $KMnO_4$)

NMR Spectroscopy (*see spectra*):

^{1}H NMR (400 MHz, $CDCl_3$): δ_H 3.47 – 3.36 (m, 4H), 1.88 – 1.75 (m, 1H), 1.72 – 1.60 (m, 1H), 1.61 – 1.54 (m, 2H), 1.46 (qd, *J* = 7.0, 2.9 Hz, 3H), 0.94 (d, *J* = 6.7 Hz, 3H), 0.89 (d, *J* = 6.6 Hz, 6H), 0.89 – 0.81 (overlapped m, 1H), 0.67 (dd, *J* = 15.4, 8.5 Hz, 1H) ppm;

^{13}C NMR (101 MHz, $CDCl_3$): δ_C 83.0, 69.6, 69.4, 39.2, 38.8, 26.9, 25.3, 25.0, 24.9, 22.9, 22.8, 22.5 ppm. The carbon attached to boron was not observed due to quadrupolar relaxation.

All recorded spectroscopic data matched those previously reported in the literature.[17]

2-(4-Butoxybutan-2-yl)-4,4,5,5-tetramethyl-1,3,2-dioxaborolane (33a) and 2-(4-butoxybutyl)-4,4,5,5-tetramethyl-1,3,2-dioxaborolane (33b)

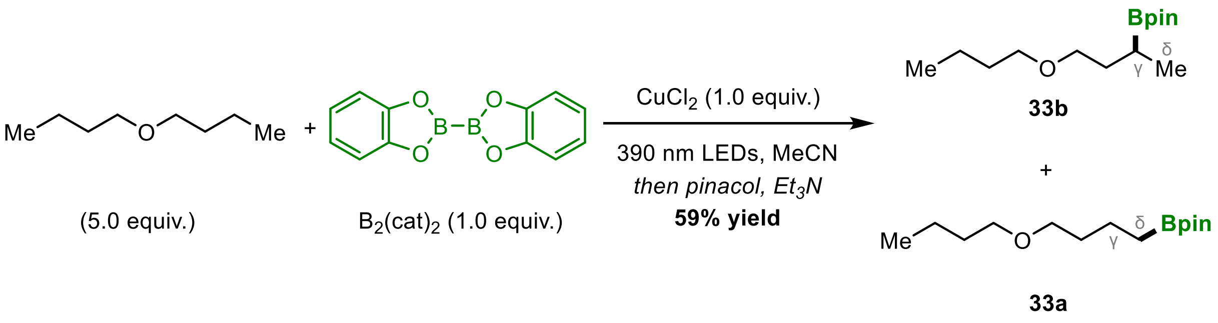

Prepared following **General Procedure B**, using dibutyl ether (195 mg, 255 µL, 1.50 mmol, 5.00 equiv.), $CuCl_2$ (40 mg, 0.30 mmol, 1.0 equiv.) and B_2cat_2 (71 mg, 0.30 mmol, 1.0 equiv.) in MeCN (1.5 mL, *c* = 0.20 M). Biotage Isolera™ flash purification on silica gel (SNAP 5 g silica cartridge), eluting with Et_2O/pentane (0 – 5%, v/v), gave **33a** and **33b** (20.6 mg + 19.9 mg, 53%) as a colorless oil. The r.r. (γ:δ = 49:51) was determined by GC-FID analysis of the crude reaction mixture (***see spectrum***).

Prepared following **General Procedure B-prestirring**, boronic ester **33** was isolated in **59%** yield (45.3 mg).

R*f* = 0.40 (1:4 EtOAc/pentane, CAM)

NMR Spectroscopy of 33a (*see spectra*):

^{1}H NMR (400 MHz, $CDCl_3$): δ_H 3.39 (td, *J* = 6.7, 1.3 Hz, 4H), 1.62 – 1.50 (m, 5H), 1.50 – 1.40 (m, 1H), 1.42 – 1.29 (m, 2H), 1.24 (s, 12H), 0.91 (t, *J* = 7.4 Hz, 3H), 0.79 (t, *J* = 7.8 Hz, 2H) ppm;

^{13}C NMR (101 MHz, $CDCl_3$): δ_C 83.2, 71.0, 70.9, 32.6, 32.1, 25.1, 21.9, 19.6, 14.2 ppm. The carbon attached to boron was not observed due to quadrupolar relaxation.

NMR Spectroscopy of 33b (*see spectra*):

^{1}H NMR (400 MHz, $CDCl_3$): δ_H 3.41 (dt, J = 7.9, 6.8 Hz, 4H), 1.92 – 1.68 (m, 1H), 1.64 – 1.48 (m, 5H), 1.42 – 1.30 (m, 2H), 1.23 (s, 12H), 0.98 (d, J = 7.2 Hz, 2H), 0.91 (t, J = 7.4 Hz, 3H) ppm;

^{13}C NMR (101 MHz, $CDCl_3$): δ_C 83.0, 70.8, 70.3, 33.2, 32.0, 25.0, 24.9, 19.5, 15.7, 14.1 ppm. The carbon attached to boron was not observed due to quadrupolar relaxation.

^{11}B NMR (128 MHz, $CDCl_3$): δ_B 34.0 (brs, 1B) ppm.

IR (film): ν_{max} 2961, 2924, 2851, 1470, 1378, 1261, 1055, 750 cm^{-1}.

HRMS (EI^+): m/z calc'd for $C_{14}H_{29}O_3B$ $[M–Me]^+$, 241.1969; found, 241.1970.

All recorded spectroscopic data matched those previously reported in the literature.[18]

3-(4,4,5,5-Tetramethyl-1,3,2-dioxaborolan-2-yl)cyclohexan-1-one (34a) and 4-(4,4,5,5-tetramethyl-1,3,2-dioxaborolan-2-yl)cyclohexan-1-one (34b)

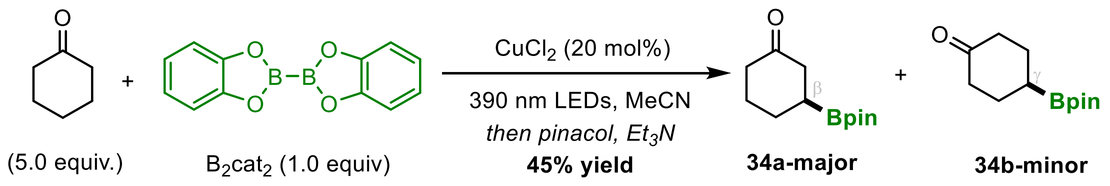

Prepared following **General Procedure A** using cyclohexanone (147 mg, 155 µL, 1.50 mmol, 5.00 equiv.), $CuCl_2$ (8.0 mg, 0.060 mmol, 0.20 equiv.) and B_2cat_2 (71 mg, 0.30 mmol, 1.0 equiv.) in MeCN (1.5 mL, *c* = 0.20 M). Flash column chromatography eluting with Et_2O/pentane (0 – 25%, v/v) on silica gel gave **34a** and **34b** (20 mg, 31%) as a colorless oil. The r.r. (β:γ = 61:39) was determined by GC-FID analysis of the crude reaction mixture (***see spectrum***).

Prepared following **General Procedure A-prestirring**, boronic ester **34** was isolated in **45%** yield (31 mg).

R_f (34a, major) = 0.50 (1:1 EtOAc/pentane, CAM)

NMR Spectroscopy of 34a (*see spectra*):

^{1}H NMR (400 MHz, $CDCl_3$) δ_H 2.43 – 2.23 (m, 4H), 2.10 – 2.00 (m, 2H), 1.92 – 1.71 (m, 2H), 1.69 – 1.55 (m, 1H), 1.50 – 1.39 (m, 1H), 1.23 (s, 12H) ppm;

^{13}C NMR (101 MHz, $CDCl_3$) δ_C 212.4, 83.6, 42.7, 42.0, 28.5, 26.6, 24.9, 24.8 ppm. *The carbon attached to boron could not be observed due to quadrupolar relaxation.*

All recorded spectroscopic data matched those previously reported in the literature.[19]

R_f (34b, minor) = 0.50 (1:1 EtOAc/pentane, CAM)

NMR Spectroscopy of 34b (*see spectra*):

^{1}H NMR (400 MHz, $CDCl_3$) δ_H 2.43 – 2.23 (m, 4H), 2.10 – 2.00 (m, 2H), 1.92 – 1.71 (m, 2H), 1.69 – 1.55 (m, 1H), 1.50 – 1.39 (m, 1H), 1.25 (s, 12H) ppm;

^{13}C NMR (101 MHz, $CDCl_3$) δ_C 212.4, 83.5, 42.4, 28.7, 25.2, 24.9 ppm; The carbon attached to boron could not be observed due to quadrupolar relaxation.

^{11}B NMR (128 MHz, $CDCl_3$) δ_B 33.89 ppm.

IR (film) ν_{max}: 2928, 2858, 1711, 1381, 1323, 1143, 980, 852, 673 cm^{-1}.

HRMS (ESI^+) calcd. for $C_{12}H_{21}O_3B$ $[M]^+$, 224.1578, found 224.1577.

3-Methyl-1-phenyl-4-(4,4,5,5-tetramethyl-1,3,2-dioxaborolan-2-yl)butan-1-one (35)

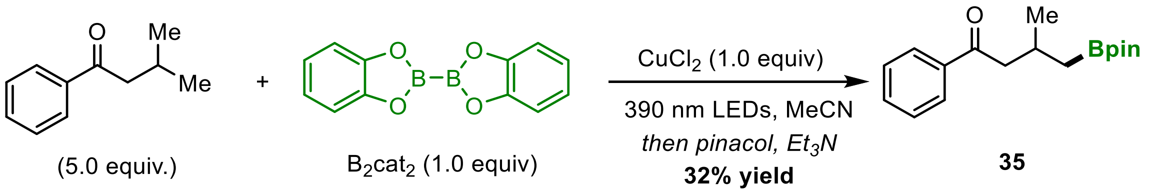

35

Prepared following **General Procedure B**, using isobutyl acetate (243 mg, 252 µL, 1.50 mmol, 5.00 equiv.), $CuCl_2$ (40 mg, 0.30 mmol, 1.0 equiv.) and B_2cat_2 (71 mg, 0.30 mmol, 1.0 equiv.) in MeCN (1.5 mL, *c* = 0.20 M). Flash column chromatography eluting with EtOAc/pentane (0 – 3%, v/v) on silica gel gave **35** (16 mg, 20%) as a colorless oil. The r.r. (>97:3) was determined by GC-FID analysis of the crude reaction mixture (***see spectrum***).

Prepared following **General Procedure B-prestirring**, boronic ester **35** was isolated in **32%** yield (28 mg).

R*f* = 0.50 (1:20 EtOAc/pentane, CAM)

NMR Spectroscopy (*see spectra*):

^{1}H NMR (400 MHz, $CDCl_3$) δ_H 8.01 – 7.96 (m, 2H), 7.57 – 7.52 (m, 1H), 7.47 – 7.42 (m, 2H), 3.06 (dd, *J* = 15.2, 5.6 Hz, 1H), 2.74 (dd, *J* = 15.2, 8.4 Hz, 1H), 2.41 (ddt, *J* = 14.3, 7.9, 6.5 Hz, 1H), 1.25 (s, 12H), 1.00 (d, *J* = 6.7 Hz, 3H), 0.93 (dd, *J* = 15.7, 6.5 Hz, 1H), 0.83 (dd, *J* = 15.7, 7.6 Hz, 1H) ppm;

^{13}C NMR (101 MHz, $CDCl_3$) δ_C 200.6, 137.6, 132.9, 128.6, 128.4, 83.2, 48.1, 26.9, 25.1, 25.0, 22.8, 21.2 ppm;

^{11}B NMR (128 MHz, $CDCl_3$) δ_B 34.1 ppm.

IR (film) ν_{max}: 2927, 1682, 1370, 1316, 1215, 1143, 970, 847, 691 cm^{-1}.

HRMS (ESI$^+$) calcd. For $C_{17}H_{25}O_3BNa$ [M+Na]$^+$,311.1794, found 311.1805.

2-Methyl-3-(4,4,5,5-tetramethyl-1,3,2-dioxaborolan-2-yl)propyl acetate (36)

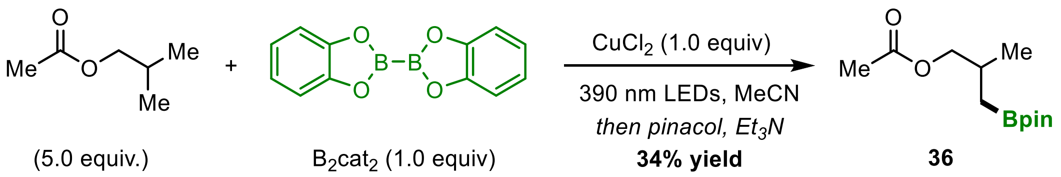

36

Prepared following **General Procedure B**, using isobutyl acetate (174 mg, 201 µL, 1.50 mmol, 5.00 equiv.), $CuCl_2$ (40 mg, 0.30 mmol, 1.0 equiv.) and B_2cat_2 (71 mg, 0.30 mmol, 1.0 equiv.) in MeCN (1.5 mL, *c* = 0.20 M). Flash column chromatography eluting with Et_2O/pentane (0 – 3%, v/v) on silica gel gave **36** (25 mg, 34%) as a colorless oil. The r.r. (>97:3) was determined by GC-FID analysis of the crude reaction mixture (***see spectrum***).

Prepared following **General Procedure B-prestirring**, boronic ester **36** was isolated in **34%** yield (25 mg).

R*f* = 0.30 (1:20 EtOAc/pentane, CAM)

NMR Spectroscopy (see spectra):

^{1}H NMR (400 MHz, $CDCl_3$) δ_H 3.93 – 3.81 (m, 2H), 2.03 (s, 3H), 1.23 (s, 12H), 0.95 (d, *J* = 6.8 Hz, 3H), 0.88 (dd, *J* = 15.6, 5.6 Hz, 1H), 0.66 (dd, *J* = 15.6, 8.8 Hz, 1H) ppm;

^{13}C NMR (101 MHz, $CDCl_3$) δ_C 171.4, 83.2, 71.0, 29.2, 25.0, 24.9, 21.1, 19.3 ppm; The carbon attached to boron could not be observed due to quadrupolar relaxation.

^{11}B NMR (128 MHz, $CDCl_3$) δ_B 33.6 ppm.

IR (film) ν_{max}: 2977, 1738, 1371, 1317, 1143, 1134, 970, 847 cm^{-1}.

HRMS (ESI^+) calcd. for $C_{12}H_{23}O_4B$ $[M-Me]^+$, 227.1449, found 227.1450.

3-Methyl-4-(4,4,5,5-tetramethyl-1,3,2-dioxaborolan-2-yl)butyl benzoate (37)

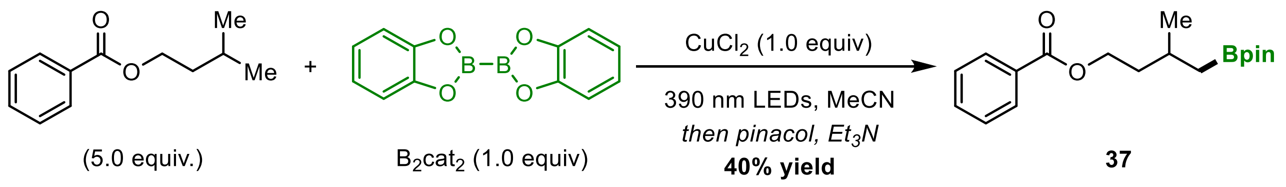

Prepared following **General Procedure B**, using isopentyl benzoate (288 mg, 1.50 mmol, 5.00 equiv.), $CuCl_2$ (40 mg, 0.30 mmol, 1.0 equiv.) and B_2cat_2 (71 mg, 0.30 mmol, 1.0 equiv.) in MeCN (1.5 mL, *c* = 0.20 M). Flash column chromatography eluting with Et_2O/pentane (0 – 10%, v/v) on silica gel gave **37** (29 mg, 31%) as a colorless oil. The r.r. (>97:3) was determined by GC-FID analysis of the crude reaction mixture (see spectrum).

Prepared following **General Procedure B-prestirring**, boronic ester **37** was isolated in **40%** yield (38 mg).

R_f = 0.30 (1:30 EtOAc/pentane, CAM)

NMR Spectroscopy (see spectra):

^{1}H NMR (400 MHz, $CDCl_3$) δ_H 8.08 – 8.01 (m, 2H), 7.58 – 7.50 (m, 1H), 7.47 – 7.38 (m, 2H), 4.35 (td, *J* = 6.8, 1.2 Hz, 2H), 2.00 – 1.89 (m, 1H), 1.84 – 1.74 (m, 1H), 1.70 – 1.59 (m, 1H), 1.24 (s, 12H), 1.01 (d, *J* = 6.8 Hz, 3H), 0.97 – 0.82 (m, 1H), 0.76 (dd, *J* = 15.6, 8.0 Hz, 1H) ppm;

^{13}C NMR (101 MHz, $CDCl_3$) δ_C 166.8, 132.9, 130.8, 129.7, 128.4, 83.1, 63.8, 38.0, 26.8, 25.0, 24.9, 22.4 ppm. The carbon attached to boron could not be observed due to quadrupolar relaxation.

^{11}B NMR (128 MHz, $CDCl_3$) δ_B 33.45 ppm.

IR (film) ν_{max}: 2976, 2926, 1718, 1370, 1314, 1142, 1111, 968, 846, 688 cm^{-1}.

HRMS (ESI^+) calcd. for $C_{18}H_{27}O_4B$ $[M+H]^+$, 319.2075, found 319.2077.

4-(4,4,5,5-Tetramethyl-1,3,2-dioxaborolan-2-yl)butyl benzoate (38a) and 3-(4,4,5,5-tetramethyl-1,3,2-dioxaborolan-2-yl)butyl benzoate (38b)

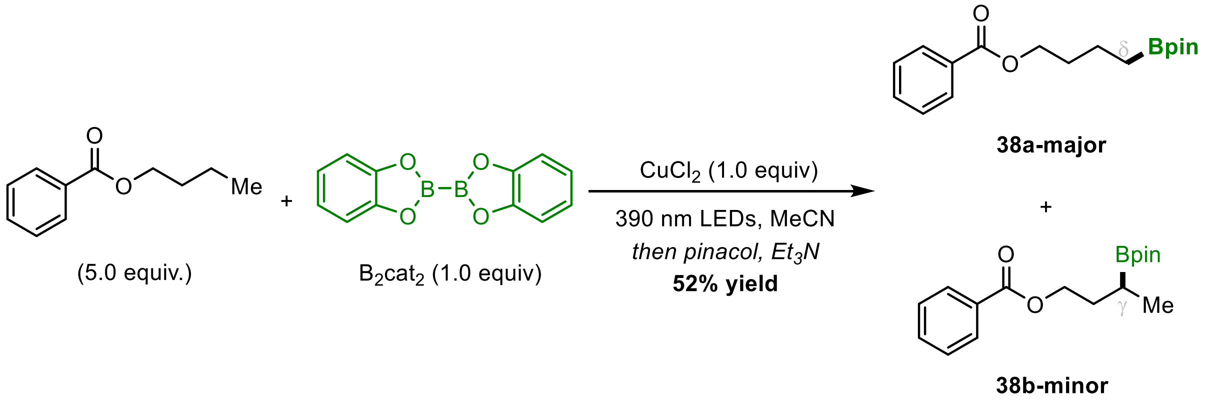

Prepared following **General Procedure B**, using butyl benzoate (267 mg, 1.50 mmol, 5.00 equiv.), $CuCl_2$ (40 mg, 0.30 mmol, 1.0 equiv.) and B_2cat_2 (71 mg, 0.30 mmol, 1.0 equiv.) in MeCN (1.5 mL, *c* = 0.20 M). Flash column chromatography eluting with Et_2O/pentane (0 – 2%, v/v) on silica gel gave **38** (47 mg, **52%**) as a colorless oil. The r.r. (δ:γ = 66:34) was determined by ^{13}C NMR analysis of the purified product **(*see spectra*)**.

Prepared following **General Procedure B-prestirring**, boronic ester **38** was isolated in 30% yield (27 mg).

R*f* (38a, major) = 0.40 (1:30 EtOAc/pentane, CAM)

NMR Spectroscopy (*see spectra*) of 38a:

^{1}H NMR (400 MHz, $CDCl_3$) δ_H 8.05 – 8.03 (m, 2H), 7.56 – 7.51 (m, 1H), 7.45 – 7.38 (m, 2H), 4.31 (t, *J* = 6.4 Hz, 2H), 1.81 – 1.68 (m, 2H), 1.62 – 1.53 (m, 2H), 1.24 (s, 12H), 0.86 (t, *J* = 7.6 Hz, 2H) ppm;

^{13}C NMR (101 MHz, $CDCl_3$) δ_C 166.82, 132.84, 130.73, 129.71, 128.41, 83.14, 65.12, 31.39, 24.96, 20.75 ppm. The carbon attached to boron could not be observed due to quadrupolar relaxation.

All recorded spectroscopic data matched those previously reported in the literature.[9]

R*f* (38b, minor) = 0.40 (1:30 EtOAc/pentane, cerium molybdate)

NMR Spectroscopy (*see spectra*) of 38b:

^{1}H NMR (400 MHz, $CDCl_3$) δ_H 8.05 – 8.03 (m, 2H), 7.56 – 7.51 (m, 1H), 7.45 – 7.38 (m, 2H), 4.36 (td, *J* = 7.2, 2.0 Hz, 2H), 2.00 – 1.91 (m, 1H), 1.83 – 1.68 (m, 2H), 1.23 (s, 12H), 1.05 (d, *J* = 7.6 Hz, 3H) ppm;

^{13}C NMR (101 MHz, $CDCl_3$) δ_C 166.82, 132.84, 130.76, 129.71, 128.39, 83.24, 64.63, 31.91, 24.90, 24.83, 15.47 ppm. The carbon attached to boron could not be observed due to quadrupolar relaxation.

^{11}B NMR (128 MHz, $CDCl_3$) δ_B 34.65 ppm.

IR (film) ν_{max}: 2977, 1719, 1314, 1279, 1143, 711 cm^{-1}.

HRMS (ESI^+) calcd. for $C_{17}H_{25}O_4B$ $[M-Me]^+$, 289.1606, found 289.1606.

4-Methyl-5-(4,4,5,5-tetramethyl-1,3,2-dioxaborolan-2-yl)pentyl furan-2-carboxylate (39)

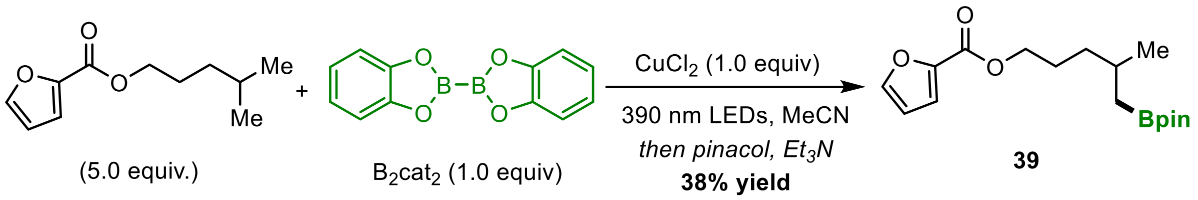

Prepared following **General Procedure B**, using 4-methylpentyl furan-2-carboxylate (294 mg, 1.50 mmol, 5.00 equiv.), $CuCl_2$ (40 mg, 0.30 mmol, 1.0 equiv.) and B_2cat_2 (71 mg, 0.30 mmol, 1.0 equiv.) in MeCN (1.5 mL, *c* = 0.20 M). Flash column chromatography eluting with Et_2O/pentane (0 – 3%, v/v) on silica gel gave **39** (35 mg, **36%**) as a colorless oil. The r.r. (>97:3) was determined by GC-FID analysis of the crude reaction mixture (***see spectrum***).

Prepared following **General Procedure B-prestirring**, boronic ester **39** was isolated in 30% yield (29 mg).

$\mathbf{R}_f$ = 0.40 (1:20 EtOAc/pentane, CAM)

NMR Spectroscopy (*see spectra*):

^{1}H NMR (400 MHz, $CDCl_3$) δ_H 7.55 (dd, *J* = 1.6, 0.8 Hz, 1H), 7.15 (dd, *J* = 3.6, 0.8 Hz, 1H), 6.49 (dd, *J* = 3.6, 1.6 Hz, 1H), 4.27 (t, *J* = 6.8 Hz, 2H), 1.84 – 1.65 (m, 3H), 1.45 – 1.15 (m, 2H), 1.22 (s, 12H), 0.93 (d, *J* = 6.8 Hz, 3H), 0.84 (dd, *J* = 15.6, 6.0 Hz, 1H), 0.67 (dd, *J* = 15.6, 8.4 Hz, 1H) ppm;

^{13}C NMR (101 MHz, $CDCl_3$) δ_C 159.0, 146.2, 145.1, 117.8, 111.9, 83.0, 65.5, 35.6, 29.3, 26.7, 25.0, 24.9, 22.4 ppm. The carbon attached to boron could not be observed due to quadrupolar relaxation.

All recorded spectroscopic data matched those previously reported in the literature.[3]

4-Methyl-5-(4,4,5,5-tetramethyl-1,3,2-dioxaborolan-2-yl)pentyl thiophene-3-carboxylate (40)

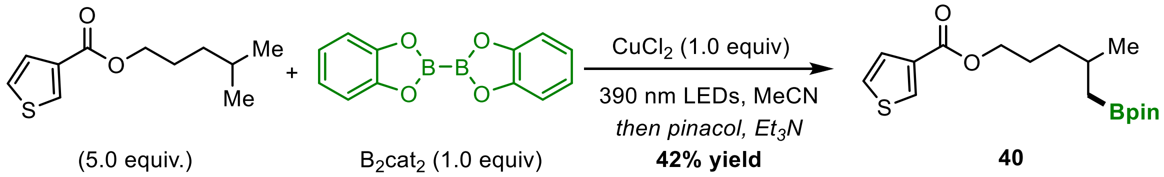

Prepared following **General Procedure B**, using 4-methylpentyl thiophene-3-carboxylate (318 mg, 1.50 mmol, 5.00 equiv.), $CuCl_2$ (40 mg, 0.30 mmol, 1.0 equiv.) and B_2cat_2 (71 mg, 0.30 mmol, 1.0 equiv.) in MeCN (1.5 mL, *c* = 0.20 M). Flash column chromatography eluting with EtOAc/pentane (0 – 2%, v/v) on silica gel gave **40** (42 mg, **42%**) as a colorless oil. The r.r. (>97:3) was determined by GC-FID analysis of the crude reaction mixture (***see spectrum***).

Prepared following **General Procedure B-prestirring**, boronic ester **40** was isolated in 19% yield (19 mg).

$\mathbf{R}_f$ = 0.40 (1:20 EtOAc/pentane, CAM)

NMR Spectroscopy (*see spectra*):

^{1}H NMR (400 MHz, $CDCl_3$) δ_H 8.11 – 8.04 (m, 1H), 7.53 – 7.48 (m, 1H), 7.30 – 7.24 (m, 1H), 4.28 – 4.20 (m, 2H), 1.81 – 1.65 (m, 3H), 1.46 – 1.35 (m, 1H), 1.34 – 1.26 (m, 1H), 1.23 (s, 12H), 0.94 (d, *J* = 6.8Hz, 3H), 0.85 (dd, *J* = 15.6, 6.0 Hz, 1H), 0.68 (dd, *J* = 15.6, 8.0 Hz, 1H) ppm;

^{13}C NMR (101 MHz, $CDCl_3$) δ_C 163.0, 134.2, 132.6, 128.1, 126.0, 83.0, 65.3, 35.8, 29.4, 26.7, 25.0, 24.9, 22.4 ppm; The carbon attached to boron could not be observed due to quadrupolar relaxation.

All recorded spectroscopic data matched those previously reported in the literature.[3]

2,2,2-Trifluoro-1-(3-hydroxypiperidin-1-yl)ethan-1-one (41a-ol) and 2,2,2-trifluoro-1-(4-hydroxy-piperidin-1-yl)ethan-1-one (41b-ol)

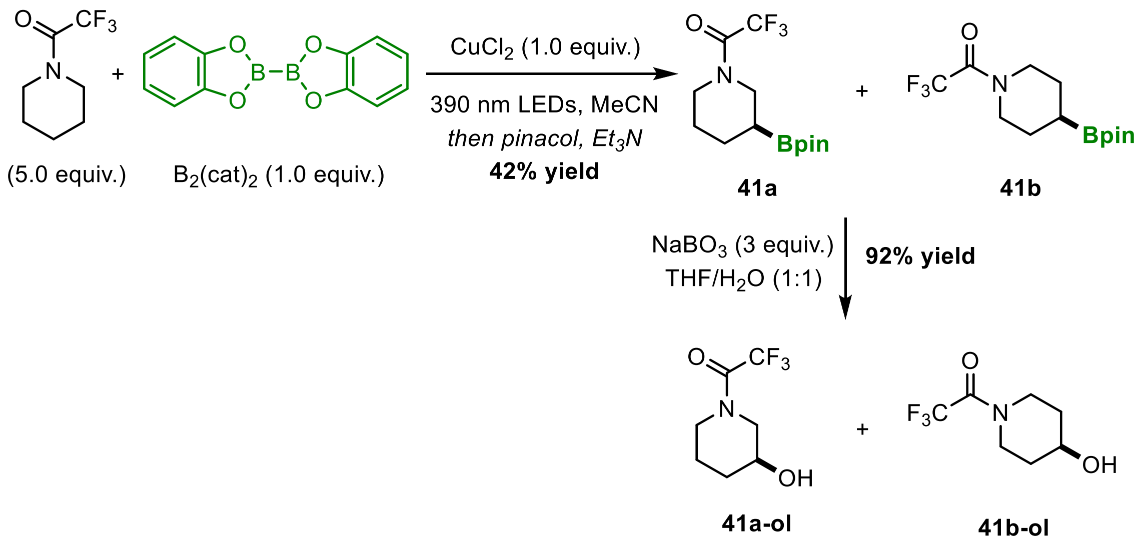

Prepared following **General Procedure B**, using 2,2,2-trifluoro-1-(piperidin-1-yl)ethan-1-one (271 mg, 1.50 mmol, 5.00 equiv.), $CuCl_2$ (40 mg, 0.30 mmol, 1.0 equiv.) and B_2cat_2 (71 mg, 0.30 mmol, 1.0 equiv.) in MeCN (1.5 mL, *c* = 0.20 M). Biotage Isolera™ flash purification on silica gel (SNAP 10 g silica cartridge), eluting with Et_2O/pentane (0 – 10%, v/v), gave a mixture of **41a** and **41b** (37.9 mg, 42%) as a colorless oil. The r.r. (α:β = 52:48) was determined by GC-FID analysis of the crude reaction mixture (*see spectrum*). Due to the cyclic amide rotation in ^{1}H NMR analysis, both isomers could not be clearly assigned, therefore, the boronic esters **41a** and **41b** was oxidized to alcohol **41a-ol** and **41b-ol**.

Prepared following **General Procedure B-prestirring**, boronic ester **41** was isolated in 37% yield (33.5 mg).

To a solution of the above mixture of **41a** and **41b** (37.7 mg, 0.122 mmol, 1.00 equiv.) in THF/H_2O (2 mL, *c* = 0.06 M, 1:1, v/v) was added $NaBO_3 \cdot 4H_2O$ (55 mg, 0.36 mmol, 3.0 equiv.). The resulting mixture was stirred at room temperature for 6 h. After that, the reaction was diluted with DCM (10 mL) and quenched with saturated aqueous NH_4Cl solution (10 mL). The layers were separated, and the aqueous layer was extracted with DCM (2 × 10 mL). The combined organic layers were dried over $MgSO_4$, filtered, and concentrated under vacuum. Biotage Isolera™ flash purification on silica gel (SNAP 10 g silica cartridge), eluting with Et_2O/pentane (5 – 20%, v/v), gave **41a-ol** and **41b-ol** (10.9 + 11.3 mg, 92%) as a colorless oil.

R_f = 0.28 (1:1 EtOAc/pentane, CAM)

NMR Spectroscopy of 41b-ol (*see spectra*):

^{1}H NMR (400 MHz, $CDCl_3$): δ_H 4.09 – 4.00 (m, 1H), 3.93 (ddd, *J* = 12.4, 7.6, 3.6 Hz, 1H), 3.85 – 3.76 (m, 1H), 3.51 (ddd, *J* = 12.4, 7.6, 3.6 Hz, 1H), 3.48 – 3.35 (m, 1H), 1.99 – 1.90 (m, 2H), 1.70 (overlapped brs, 1OH), 1.64 (m, 2H) ppm;

^{13}C NMR (101 MHz, $CDCl_3$): δ_C 156.6 (sq, *J* = 36.0 Hz), 116,7 (q, *J* = 288.9 Hz), 65.9, 42.7 + 42.6 (2 rotamers), 40.61, 34.3 + 33.4 (2 rotamers) ppm;

^{19}F NMR (377 MHz, $CDCl_3$): δ_F –69.0 ppm.

NMR Spectroscopy of 41a-ol (*see spectra*):

^{1}H NMR (400 MHz, $CDCl_3$): δ_H 3.90 – 3.60 (m, 3H), 3.48 – 3.19 (m, 2H), 2.63 (brs, 1OH), 2.02 – 1.81 (m, 2H), 1.70 – 1.48 (m, 2H) ppm;

^{13}C NMR (101 MHz, $CDCl_3$): (major rotamor): δ_C 156.3 (sq, *J* = 36.1 Hz), 116.7 (q, *J* = 288.9 Hz), 65.6, 50.8, 43.5, 30.5, 22.9 ppm;

^{13}C NMR (101 MHz, $CDCl_3$) (minor rotamor): δ_C 156.3 (sq, *J* = 36.1 Hz), 116.7 (q, *J* = 288.9 Hz), 66.1, 52.1, 46.4, 32.5, 21.9 ppm;

^{19}F NMR (377 MHz, $CDCl_3$) (major rotamor): δ_F –68.9 ppm;

^{19}F NMR (377 MHz, $CDCl_3$) (minor rotamor): δ_F –68.4 ppm.

All recorded spectroscopic data matched those previously reported in the literature.[20,21]

2,2,2-Trifluoro-N-(3-methyl-4-(4,4,5,5-tetramethyl-1,3,2-dioxaborolan-2-yl)butyl)acetamide (42)

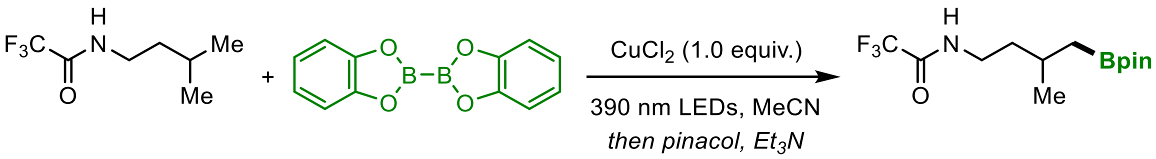

Prepared following **General Procedure B**, using 2,2,2-trifluoro-N-isopentylacetamide (273 mg, 1.50 mmol, 5.00 equiv.), $CuCl_2$ (40 mg, 0.30 mmol, 1.0 equiv.) and B_2cat_2 (71 mg, 0.30 mmol, 1.0 equiv.) in MeCN (1.5 mL, *c* = 0.20 M). Biotage Isolera™ flash purification on silica gel (SNAP 10 g silica cartridge), eluting with Et_2O/pentane (2 – 10%, v/v), gave **42** (37.5 mg, 41%) as a colorless oil. The r.r. (>97:3) was determined by GC-FID analysis of the crude reaction mixture (*see spectrum*). Unreacted alkane was recovered in 90% yield (238 mg).

Prepared following **General Procedure B-prestirring**, boronic ester **42** was isolated in **44%** yield (40.5 mg).

R*f* = 0.30 (1:4 EtOAc/pentane, CAM)

NMR Spectroscopy (*see spectra*):

^{1}H NMR (400 MHz, $CDCl_3$): δ_H 6.88 (br, 1H), 3.52 – 3.41 (m, 1H), 3.30 – 3.19 (m, 1H), 1.85 – 1.69 (m, 1H), 1.63 – 1.51 (m, 1H), 1.52 – 1.38 (m, 1H), 1.25 (s, 12H), 0.97 (d, *J* = 6.8 Hz, 3H), 0.80 (d, *J* = 6.8 Hz, 2H)

ppm;

^{13}C NMR (101 MHz, $CDCl_3$): δ_C 157.0 (q, *J* = 36.4 Hz), 116.1 (q, *J* = 288.0 Hz), 83.5, 38.4, 37.8, 26.8, 25.0, 24.8, 23.3 ppm; The carbon attached to boron was not observed due to quadrupolar relaxation.

^{19}F NMR (377 MHz, $CDCl_3$): δ_F –75.9 (s, 3F) ppm.

All recorded spectroscopic data matched those previously reported in the literature.[3]

2-(3-Methyl-4-(4,4,5,5-tetramethyl-1,3,2-dioxaborolan-2-yl)butyl)isoindoline-1,3-dione (43)

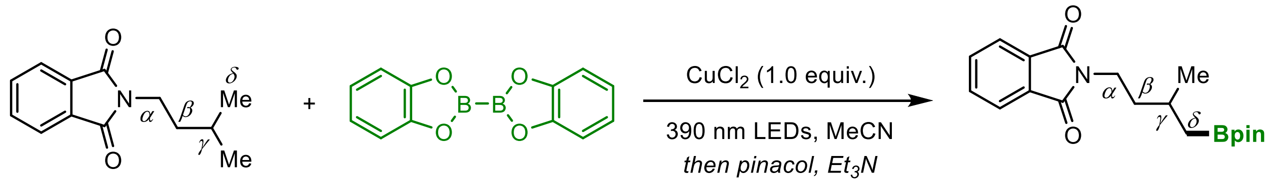

(5.0 equiv.) $B_2(cat)_2$ (1.0 equiv.) **35% yield** **43**

Prepared following **General Procedure B**, using 2-isopentylisoindoline-1,3-dione (326 mg, 1.50 mmol, 5.00 equiv.), $CuCl_2$ (40 mg, 0.30 mmol, 1.0 equiv.), and B_2cat_2 (71 mg, 0.30 mmol, 1.0 equiv.) in MeCN (1.5 mL, *c* = 0.20 M). Flash column chromatography, eluting with eluting with Et_2O/pentane (0 – 14%, v/v) gave **43** (30 mg, 29%) as a colorless oil. The r.r. (*α*:*β*:*δ* =4:11:85) was determined by GC-FID analysis of the crude reaction mixture (***see spectrum***). Only the major product (*δ* isomer) is listed for characterisation.

Prepared following **General Procedure B-prestirring**, boronic ester **43** was isolated in **35%** yield (36 mg).

R*f* =0.28 (1:3 Et_2O/pentane, UV, CAM)

NMR Spectroscopy (*see spectra*):

^{1}H NMR (400 MHz, $CDCl_3$) δ_H 7.82 (dd, *J* = 5.6, 3.2 Hz, 2H), 7.69 (dd, *J* = 5.6, 3.2 Hz, 2H), 3.74 – 3.65 (m, 2H), 1.80 – 1.67 (m, 2H), 1.57 – 1.47 (m, 1H), 1.22 (s, 12H), 1.02 (d, *J* = 6.4 Hz, 3H), 0.89 (dd, *J* = 15.6, 5.6 Hz, 1H), 0.73 (dd, *J* = 15.6, 8.0 Hz, 1H) ppm ;

^{13}C NMR (101 MHz, $CDCl_3$) δ_C 168.5, 133.9, 132.5, 123.2, 83.1, 37.9, 36.6, 27.5, 25.0, 24.9, 22.1 ppm. The carbon attached to boron was not observed due to quadrupolar relaxation.

^{11}B NMR (128 MHz, $CDCl_3$) δ_B 33.7 ppm.

IR (film): 2976, 2920, 2850, 1773, 1711, 1467, 1436, 1395, 1369, 1317, 1166, 1144, 969, 872, 846 cm^{-1}.

HRMS (ESI^+): m/z calculated for $C_{19}H_{26}NBO_4$ $[M+H]^+$, 344.2028; found, 344.2034.

2-(4-(4,4,5,5-Tetramethyl-1,3,2-dioxaborolan-2-yl)butyl)isoindoline-1,3-dione (44)

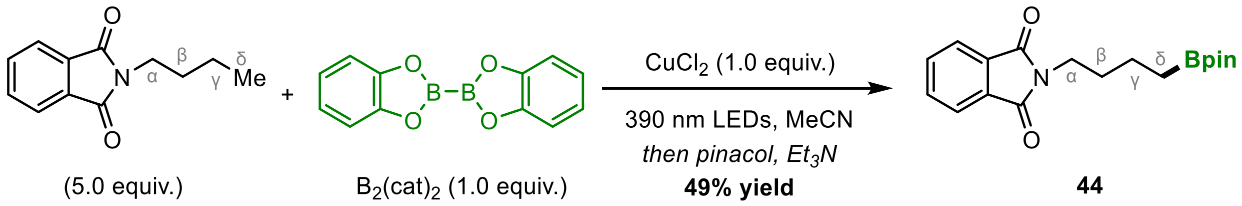

Prepared following **General Procedure B**, using 2-butylisoindoline-1,3-dione (305 mg, 1.50 mmol, 5.00 equiv.), $CuCl_2$ (40 mg, 0.30 mmol, 1.0 equiv.) and B_2cat_2 (71 mg, 0.30 mmol, 1.0 equiv.) in MeCN (3.0 mL, *c* = 0.10 M). Flash column chromatography, eluting with Et_2O/pentane (0 – 14%, v/v) gave **44** (27 mg, 27%) as a colorless oil. The r.r. (*α*:*β*:*γ*:*δ* = 2:12:36:50) was determined by GC-FID analysis of the crude reaction mixture (***see spectrum***). Only the major product (*δ* isomer) is listed for characterisation.

Prepared following **General Procedure B-prestirring**, boronic **44** was isolated in **49%** yield (48 mg). Unreacted 2-butylisoindoline-1,3-dione was recovered in 87% yield (252 mg, 1.24 mmol).

R_f = 0.23 (1:3 Et_2O/pentane, UV, CAM)

NMR Spectroscopy (*see spectra*):

^{1}H NMR (400 MHz, $CDCl_3$): δ_H 7.83 (dd, *J* = 5.6, 3.2 Hz, 2H), 7.69 (dd, *J* = 5.6, 3.2 Hz, 2H), 3.67 (t, *J* = 7.2 Hz, 2H), 1.73 – 1.64 (m, 2H), 1.51 – 1.44 (m, 2H), 1.22 (s, 12H), 0.82 (t, *J* = 7.6 Hz, 2H) ppm;

^{13}C NMR (101 MHz, $CDCl_3$): δ_C 168.6, 133.9, 132.4, 123.3, 83.1, 38.1, 31.2, 25.0, 21.5 ppm. The carbon attached to boron was not observed due to quadrupolar relaxation.

All recorded spectroscopic data matched those previously reported in the literature.[22]

2-(4-Methyl-5-(4,4,5,5-tetramethyl-1,3,2-dioxaborolan-2-yl)pentyl)isoindoline-1,3-dione (45)

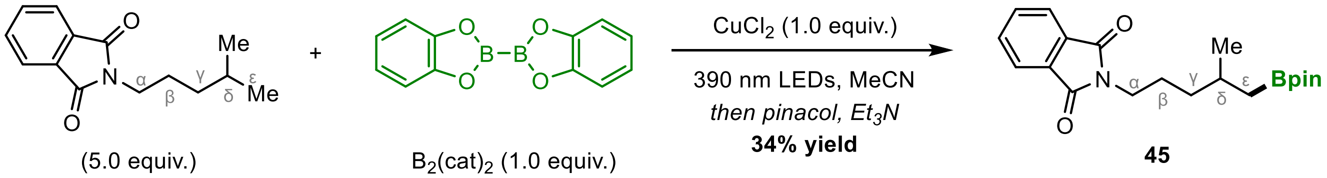

Prepared following **General Procedure B**, using 2-(4-methylpentyl)isoindoline-1,3-dione (173 mg, 0.75 mmol, 5.00 equiv.), $CuCl_2$ (20 mg, 0.15 mmol, 1.0 equiv.), and B_2cat_2 (36 mg, 0.15 mmol, 1.0 equiv.) in MeCN (0.75 mL, *c* = 0.20 M). Flash column chromatography, eluting with Et_2O/pentane (0 – 33%, v/v) gave **45** (10 mg, 19%) as a colorless oil. The r.r. (*β*:*γ*:*ε* = 6:12:82) was determined by GC-FID analysis of the crude reaction mixture (***see spectrum***). Only the major product (*ε* isomer) is listed for characterisation.

Prepared following **General Procedure B-prestirring**, boronic **45** was isolated in **34%** yield (36 mg).

R_f = 0.38 (1:2 Et_2O/pentane, $KMnO_4$)

NMR Spectroscopy (*see spectra*):

^{1}H NMR (400 MHz, $CDCl_3$) δ_H 7.83 (dd, J = 5.6, 3.2 Hz, 2H), 7.70 (dd, J = 5.6, 3.2 Hz, 2H), 3.66 (td, J = 7.8, 1.6 Hz, 2H), 1.75 – 1.62 (m, 3H), 1.38 – 1.27 (m, 2H), 1.21 (s, 12H), 0.91 (d, J = 6.8 Hz, 3H), 0.82 (dd, J = 15.6, 6.0 Hz, 1H), 0.65 (dd, J = 15.6, 8.4 Hz, 1H) ppm ;

^{13}C NMR (101 MHz, $CDCl_3$) δ_C 168.6, 133.9, 132.4, 123.3, 83.0, 38.5, 36.6, 29.4, 26.6, 25.0, 24.9, 22.3 ppm. The carbon attached to boron was not observed due to quadrupolar relaxation.

All recorded spectroscopic data matched those previously reported in the literature.[3]

1,1,1-Trifluoro-N-(3-methyl-4-(4,4,5,5-tetramethyl-1,3,2-dioxaborolan-2-yl)butyl)methanesulfonamide (46)

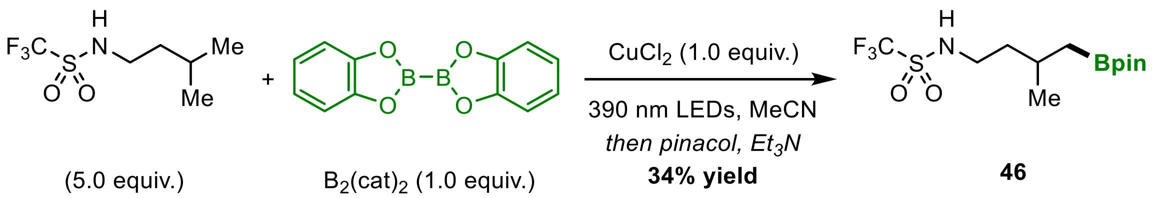

Prepared following **General Procedure B**, using 1,1,1-trifluoro-N-isopentylmethanesulfonamide **46-S** (330 mg, 1.50 mmol, 5.00 equiv.), $CuCl_2$ (40 mg, 0.30 mmol, 1.0 equiv.) and B_2cat_2 (71 mg, 0.30 mmol, 1.0 equiv.) in MeCN (1.5 mL, c = 0.20 M). Biotage Isolera™ flash purification on silica gel (SNAP 10 g silica cartridge), eluting with Et_2O/pentane (2 – 10%, v/v), gave **46** (28.4 mg, 27%) as a colorless oil. The r.r. (95:5) was determined by GC-FID analysis of the crude reaction mixture (***see spectrum***).

Prepared following **General Procedure B-prestirring**, boronic ester **46** was isolated in **34%** yield (35.1 mg).

R*f* = 0.65 (1:9 EtOAc/pentane, CAM)

NMR Spectroscopy (*see spectra*):

^{1}H NMR (400 MHz, $CDCl_3$): δ_H 5.78 (br, 1H), 3.38 – 3.22 (m, 2H), 1.87 – 1.74 (m, 1H), 1.64 – 1.53 (m, 1H), 1.51 – 1.40 (m, 1H), 1.26 (s, 6H), 1.26 (s, 6H), 0.97 (d, J = 6.8 Hz, 3H), 0.80 (dd, J = 6.8, 2.0 Hz, 2H) ppm;

^{13}C NMR (101 MHz, $CDCl_3$): δ_C 120.0 (q, J = 322.2 Hz), 83.8, 42.9, 38.9, 26.5, 25.0, 24.7, 23.5 ppm; The carbon attached to boron was not observed due to quadrupolar relaxation.

^{19}F NMR (377 MHz, $CDCl_3$): δ_F –77.4 ppm.

^{11}B NMR (128 MHz, $CDCl_3$): δ_B 33.9 (brs, 1B) ppm.

IR (film): ν_{max} 2974, 2867, 1373,1325, 1188, 1145, 851, 749, 611 cm^{-1}.

HRMS (APCI$^+$): m/z calc'd for $C_{12}H_{23}NO_4SBF_3$ $[M+H]^+$, 346.1466; found, 346.1483.

4-(4,4,5,5-Tetramethyl-1,3,2-dioxaborolan-2-yl)butanenitrile (47)

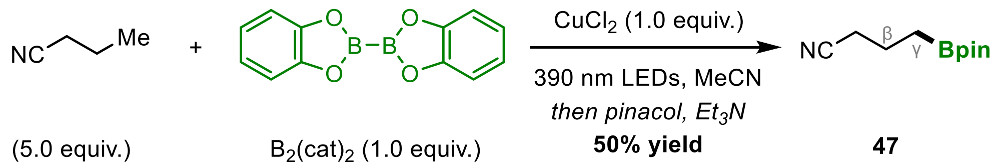

Prepared following **General Procedure B**, using butyronitrile (104 mg, 131 µL, 1.50 mmol, 5.00 equiv.), $CuCl_2$ (40 mg, 0.30 mmol, 1.0 equiv.) and B_2cat_2 (71 mg, 0.30 mmol, 1.0 equiv.) in MeCN (1.5 mL, *c* = 0.20 M). Biotage Isolera™ flash purification on silica gel (SNAP 5 g silica cartridge), eluting with Et_2O/pentane (0 – 5%, v/v), gave **47** and its isomers (26.3 mg, 45%) as a colorless oil. The r.r. (β:γ = 32:68) was determined by GC-FID analysis of the crude reaction mixture (***see spectrum***). Only the major product (γ isomer) is listed for characterisation.

Prepared following **General Procedure B-prestirring**, boronic ester **47** was isolated in **50%** yield (29.4 mg).

R*f* = 0.50 (1:6 EtOAc/pentane, $KMnO_4$)

NMR Spectroscopy (*see spectra*):

^{1}H NMR (400 MHz, $CDCl_3$): δ_H 2.36 (t, *J* = 7.2 Hz, 2H), 1.82 – 1.72 (m, 2H), 1.24 (s, 12H), 0.94 (t, *J* = 7.6 Hz, 1H) ppm;

^{13}C NMR (101 MHz, $CDCl_3$): δ_C 120.0, 83.5, 25.0, 20.5, 19.3 ppm. The carbon attached to boron was not observed due to quadrupolar relaxation.

All recorded spectroscopic data matched those previously reported in the literature.[3]

4-Methyl-5-(4,4,5,5-tetramethyl-1,3,2-dioxaborolan-2-yl)pentanenitrile (48)

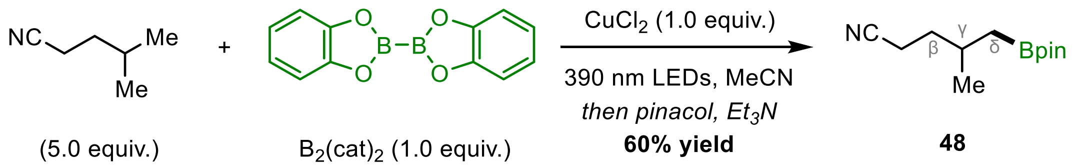

Prepared following **General Procedure B**, using 4-methylpentanenitrile (146 mg, 182 µL, 1.50 mmol, 5.00 equiv.), $CuCl_2$ (40 mg, 0.30 mmol, 1.0 equiv.) and B_2cat_2 (71 mg, 0.30 mmol, 1.0 equiv.) in MeCN (1.5 mL, *c* = 0.20 M). Biotage Isolera™ flash purification on silica gel (SNAP 5 g silica cartridge), eluting with Et_2O/pentane (0 – 5%, v/v), gave a mixture of **48** and other isomers (36.1 mg, 54%) as a colorless oil. The r.r. (β:γ:δ = 20:<1:80) was determined by GC-FID analysis of the crude reaction mixture (***see spectrum***). Only the major product (γ isomer) is listed for characterisation.

Prepared following **General Procedure B-prestirring**, boronic ester **48** was isolated in **60%** yield (40.6 mg).

R*f* = 0.45 (1:6 EtOAc/pentane, $KMnO_4$)

NMR Spectroscopy (*see spectra*):

^{1}H NMR (400 MHz, $CDCl_3$): δ_H 2.38 – 2.23 (m, 2H), 1.90 – 1.77 (m, 1H), 1.74 – 1.61 (m, 1H), 1.63 – 1.48

(m, 1H), 1.24 (s, 12H), 0.95 (d, *J* = 6.8 Hz, 3H), 0.83 (dd, *J* = 15.6, 5.6 Hz, 1H), 0.70 (dd, *J* = 15.6, 8.0 Hz, 1H) ppm;

^{13}C NMR (101 MHz, $CDCl_3$): δ_C 120.2, 83.3, 34.6, 28.9, 25.0, 24.9, 21.6, 15.2 ppm. The carbon attached to boron was not observed due to quadrupolar relaxation.

^{11}B NMR (128 MHz, $CDCl_3$): δ_B 33.7 (brs, 1B) ppm.

IR (film): ν_{max} 2978, 2927, 1372, 1321, 1144, 970, 846, 750 cm^{-1}.

HRMS (EI$^+$): m/z calc'd for $C_{13}H_{25}NO_2B$ [M–Me]$^+$, 223.1739; found, 223.1738.

2,2'-(2-Methylpropane-1,3-diyl)bis(4,4,5,5-tetramethyl-1,3,2-dioxaborolane) (49)

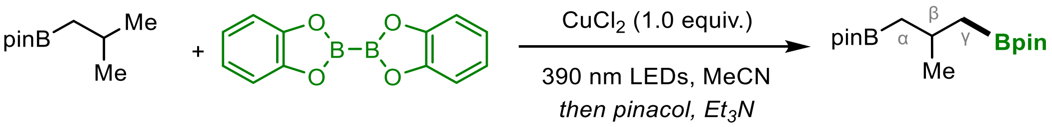

(5.0 equiv.) $B_2(cat)_2$ (1.0 equiv.) **49**

Prepared following **General Procedure B**, using 2-isobutyl-4,4,5,5-tetramethyl-1,3,2-dioxaborolane (276 mg, 1.50 mmol, 5.00 equiv.), $CuCl_2$ (40 mg, 0.30 mmol, 1.0 equiv.) and B_2cat_2 (71 mg, 0.30 mmol, 1.0 equiv.) in MeCN (1.5 mL, *c* = 0.20 M). Biotage Isolera™ flash purification on silica gel (SNAP 10 g silica cartridge), eluting with Et_2O/pentane (0 – 20%, v/v), gave a mixture of **49** and the other isomer (50.3 mg, **54%**) as a colorless oil. The r.r. (α:β:γ = 19:<1:80) was determined by GC-FID analysis of the crude reaction mixture (***see spectrum***). Only the major product (γ isomer) is listed for characterisation.

Prepared following **General Procedure B-prestirring**, boronic ester **49** was isolated in 50% yield (46.4 mg).

R*f* = 0.25 (1:4 EtOAc/pentane, CAM)

NMR Spectroscopy (*see spectra*):

^{1}H NMR (400 MHz, $CDCl_3$): δ_H 2.07 – 1.94 (m, 1H), 1.23 (s, 24H), 0.96 (d, *J* = 6.4 Hz, 3H), 0.87 (dd, *J* = 15.6, 5.6 Hz, 2H), 0.75 (dd, *J* = 15.6, 8.4 Hz, 2H) ppm;

^{13}C NMR (101 MHz, $CDCl_3$): δ_C 82.9, 26.3, 25.0, 24.9, 24.8 ppm. The carbon attached to boron was not observed due to quadrupolar relaxation.

^{11}B NMR (128 MHz, $CDCl_3$): δ_B 34.6 (brs, 2B) ppm.

IR (film): ν_{max} 2937, 2862, 1359, 1311, 1286, 1219, 1145, 844, 761, 750 cm^{-1}.

HRMS (EI$^+$): m/z calc'd for $C_{16}H_{32}O_4B_2$ [M–Me]$^+$, 295.2246; found, 295.2243.

Dimethyl(phenyl)((4,4,5,5-tetramethyl-1,3,2-dioxaborolan-2-yl)methyl)silane (50)

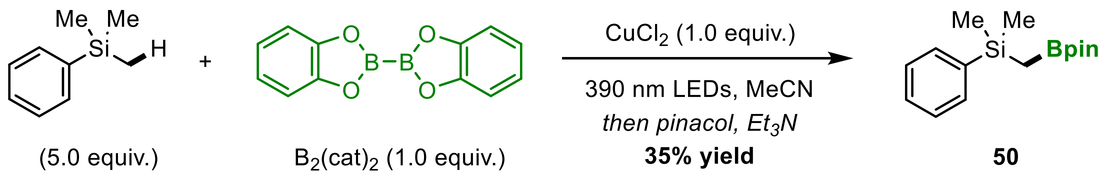

Prepared following **General Procedure B**, trimethyl(phenyl)silane (225 mg, 258 μL, 1.50 mmol, 5.00 equiv.), $CuCl_2$ (40 mg, 0.30 mmol, 1.0 equiv.) and B_2cat_2 (71 mg, 0.30 mmol, 1.0 equiv.) in MeCN (3.0 mL, *c* = 0.10 M). Flash column chromatography, eluting with Et_2O/pentane (0 – 2%, v/v) gave **50** (29 mg, **35%**) as a colorless oil.

Prepared following **General Procedure B-prestirring**, boronic ester **50** was isolated in 19% yield (15.7 mg).

R_f = 0.28 (1:49 Et_2O/pentane, CAM)

NMR Spectroscopy (*see spectra*):

^{1}**H NMR** (400 MHz, $CDCl_3$) δ_H 7.59 – 7.51 (m, 2H), 7.37 – 7.30 (m, 3H), 1.18 (s, 12H), 0.36 (s, 2H), 0.33 (s, 6H) ppm;

^{13}**C NMR** (101 MHz, $CDCl_3$) δ_C 140.3, 133.6, 128.9, 127.8, 83.0, 25.0, -0.8 ppm. The carbon attached to boron was not observed due to quadrupolar relaxation.

All recorded spectroscopic data matched those previously reported in the literature.[3]

Triethyl(2-(4,4,5,5-tetramethyl-1,3,2-dioxaborolan-2-yl)ethyl)silane (51)

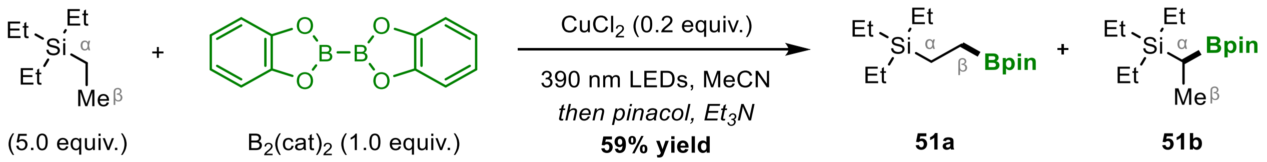

Prepared following **General Procedure A**, using tetraethylsilane (216 mg, 284 μL, 1.50 mmol, 5.00 equiv.), $CuCl_2{\cdot}2H_2O$ (10 mg, 0.060 mmol, 0.20 equiv.) and B_2cat_2 (71 mg, 0.30 mmol, 1.0 equiv.) in MeCN (3.0 mL, *c* = 0.10 M). Flash column chromatography, eluting with Et_2O/pentane (0 – 5%, v/v) gave **51a** and **51b** (27 mg, 33%) as a colorless oil. The r.r. (α:β = 69:31) was determined by GC-FID analysis of the crude product (***see spectrum***).

Prepared following **General Procedure A-prestirring**, boronic ester **51** was isolated in **59%** yield (48 mg).

R_f = 0.48 (1:19 Et_2O/pentane, CAM)

NMR Spectroscopy of 51a (*see spectra*):

^{1}**H NMR** (400 MHz, $CDCl_3$): δ_H 1.24 (s, 12H), 0.92 (t, *J* = 8.0 Hz, 9H), 0.75 – 0.68 (m, 2H), 0.61 – 0.53 (m, 2H), 0.49 (q, *J* = 8.0 Hz, 6H) ppm;

^{13}**C NMR** (101 MHz, $CDCl_3$) δ_C 83.0, 25.0, 7.6, 4.1, 3.1 ppm. The carbon attached to boron was not observed due to quadrupolar relaxation.

NMR Spectroscopy of 51b (see spectra):

^{1}H NMR (400 MHz, $CDCl_3$): δ_H 1.22 (s, 6H), 1.22 (s, 6H), 1.02 (d, J = 7.2 Hz, 3H), 0.95 (t, J = 8.0 Hz, 9H), 0.57 (q, J = 8.0 Hz, 6H), 0.42 (q, J = 7.2 Hz, 1H) ppm;

^{13}C NMR (101 MHz, $CDCl_3$) δ_C 82.6, 25.3, 24.9, 9.4, 7.7, 3.4 ppm. The carbon attached to boron was not observed due to quadrupolar relaxation.

All recorded spectroscopic data matched those previously reported in the literature.[3]

_N_-Phth _L_-Leucine derivative pinacol boronic acid ester (52)

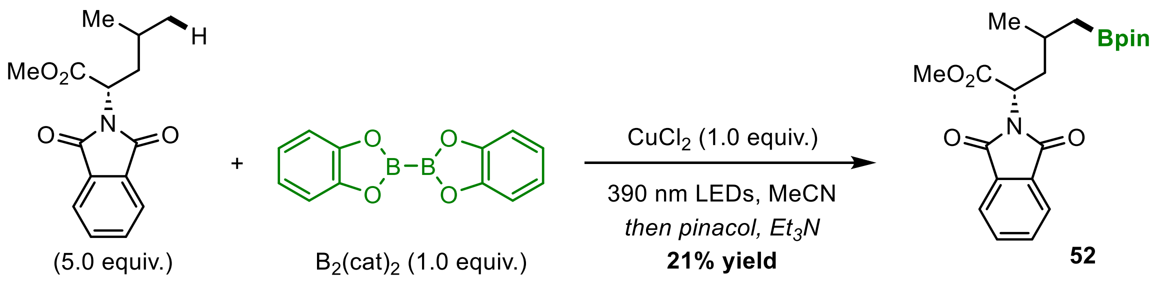

Prepared following **General Procedure B**, using methyl (*S*)-2-(1,3-dioxoisoindolin-2-yl)-4-methylpentanoate (413 mg, 1.50 mmol, 5.00 equiv.), $CuCl_2$ (40 mg, 0.30 mmol, 1.0 equiv.), and B_2cat_2 (71 mg, 0.30 mmol, 1.0 equiv.) in MeCN (3.0 mL, c = 0.10 M). Flash column chromatography, eluting with Et_2O/pentane (0 – 14%, v/v) gave **52** (25 mg, **21%**) as a colorless oil. The r.r. (>97:3) and d.r. (*d.r.* =55:45) were determined by GC-FID analysis of the crude reaction mixture (see spectrum).

Prepared following **General Procedure B-prestirring**, boronic ester **52** was isolated in 20% yield (24 mg).

R_f = 0.25 (1:3 Et_2O/pentane, CAM)

NMR Spectroscopy (see spectra):

^{1}H NMR (400 MHz, $CDCl_3$) δ_H (*55:45 ratio of diastereomers*)7.86 (dd, J = 5.6, 3.2 Hz, 2H), 7.75 – 7.71 (m, 2H), 4.98 (dd, J = 10.8, 4.4 Hz, 0.45H), 4.94 (dd, J = 11.6, 3.6 Hz, 0.55H), 3.72 (s, 3H), 2.39 (ddd, J = 14.4, 11.8, 3.4 Hz, 0.55H), 2.28 (ddd, J = 14.4, 10.8, 5.2 Hz, 0.45H), 2.14 (ddd, J = 14.4, 9.2, 4.8 Hz, 0.45H), 1.97 (ddd, J = 14.4, 10.8, 3.6 Hz, 0.55H), 1.76 – 1.59 (m, 1H), 1.21 + 1.18 +1.17 (3 × s, 12H), 1.00 + 0.96 (2 × d, J = 6.4 and 6.8 Hz, 3H), 1.00 – 0.95 (m, 0.45H), 0.83 (dd, J = 15.6, 6.8 Hz, 0.55H), 0.74 (dd, J = 15.6, 7.2 Hz, 0.55H), 0.68 (dd, J = 15.6, 9.2 Hz, 0.45H), ppm;

^{13}C NMR (101 MHz, $CDCl_3$) δ_C (*55:45 ratio of diastereomers*)170.5 + 170.4 (diastereomeric peaks), 167.9 + 167.8 (diastereomeric peaks), 134.2 + 134.2 (diastereomeric peaks), 132.2 + 132.1 (diastereomeric peaks), 123.6 + 123.6 (diastereomeric peaks), 83.2 + 83.1 (diastereomeric peaks), 52.8 + 52.8 (diastereomeric peaks), 50.8 + 50.7 (diastereomeric peaks), 38.0 + 37.8 (diastereomeric peaks), 26.9 + 26.5 (diastereomeric peaks), 25.0, 24.9 + 24.8 (diastereomeric peaks), 22.6 + 21.2 (diastereomeric peaks) ppm. The carbon attached to boron was not observed due to quadrupolar relaxation.

All recorded spectroscopic data matched those previously reported in the literature.[3]

N-Phth *L*-*tert*-Leucine derivative pinacol boronic acid ester (53)

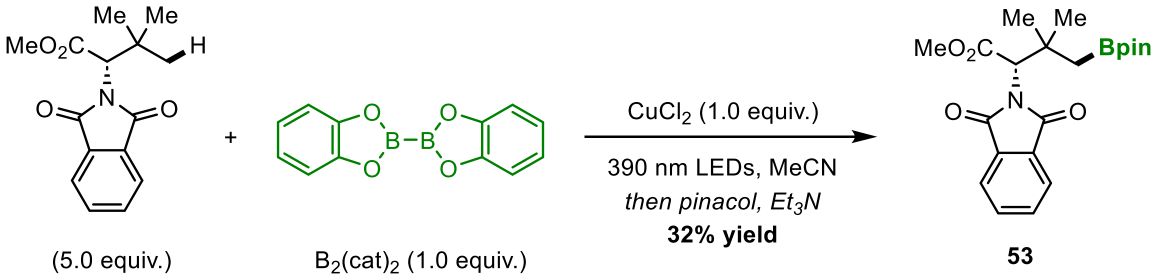

Prepared following **General Procedure B**, using methyl (*S*)-2-(1,3-dioxoisoindolin-2-yl)-3,3-dimethylbutanoate (413 mg, 1.50 mmol, 5.00 equiv.), $CuCl_2$ (40 mg, 0.30 mmol, 1.0 equiv.), and B_2cat_2 (71 mg, 0.30 mmol, 1.0 equiv.) in MeCN (3.0 mL, *c* = 0.10 M). Flash column chromatography, eluting with Et_2O/pentane (0 – 14%, v/v) gave **53** (25 mg, 21%) as a colorless oil. The r.r. (>97:3) was determined by GC-FID analysis of the crude reaction mixture (***see spectrum***).

Prepared following **General Procedure B-prestirring**, boronic ester **53** was isolated in **32%** yield (38 mg).

R_f =0.25 (1:3 Et_2O/pentane, CAM)

NMR Spectroscopy (*see spectra*):

^{1}H NMR (400 MHz, $CDCl_3$) δ_H 7.85 (dd, *J* = 5.6, 3.2 Hz, 2H), 7.73 (dd, *J* = 5.6, 3.2 Hz, 2H), 4.82 (s, 1H), 3.65 (s, 3H), 1.30 (d, *J* = 15.2 Hz, 1H),1.28 (s, 3H), 1.21 (s, 6H), 1.20 (s, 6H), 1.08 (d, *J* = 15.2 Hz, 1H) ppm;

^{13}C NMR (101 MHz, $CDCl_3$) δ_C 168.6, 168.3, 134.2, 132.1, 123.6, 83.1, 60.3, 52.1, 37.6, 27.7, 27.0, 25.0, 24.9 ppm. The carbon attached to boron was not observed due to quadrupolar relaxation.

All recorded spectroscopic data matched those previously reported in the literature.[3]

Eucalyptol pinacol boronic acid ester (54)

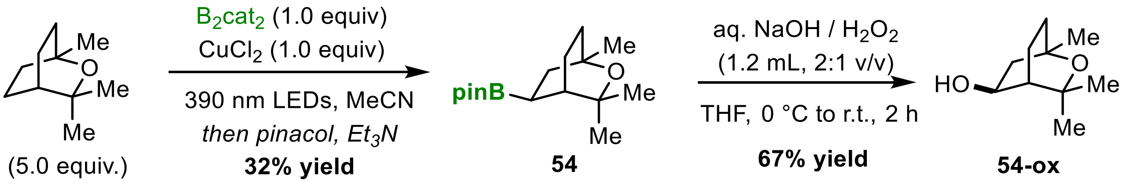

Prepared following **General Procedure B**, using eucalyptol (231 mg, 251 μL 1.50 mmol, 5.00 equiv.), $CuCl_2$ (40 mg, 0.30 mmol, 1.0 equiv.) and B_2cat_2 (71 mg, 0.30 mmol, 1.0 equiv.) in MeCN (1.5 mL, *c* = 0.20 M). Flash column chromatography (1:20 EtOAc/pentane) on silica gel gave **54** (27 mg, **32%**) as a colorless oil. The r.r. (>97:3) and d.r. (>97:3) were determined by GC-FID analysis of the crude reaction mixture (***see spectrum***). But the specific borylated product could not be confirmed by ^{1}H NMR or GC-FID analysis, therefore, the boronic ester **54** was oxidized to the alcohol **54-ox**.

Prepared following **General Procedure B-prestirring**, boronic ester **54** was isolated in 30% yield (25 mg).

To a solution of boronic ester **54** (27 mg, 0.096 mmol, 1.0 equiv.) in THF (3.0 mL) at 0 °C was added dropwise a solution of 2 M aqueous NaOH/30% aqueous H_2O_2 (1.5 mL, 2:1 v/v). The mixture was stirred at 0 °C for 1 h and then at room temperature for 1 h. Water (5 mL) and EtOAc (20 mL) were added and the layers separated. The aqueous layer was extracted with EtOAc (3 × 15 mL) and the combined organic layers were washed with brine, dried over $MgSO_4$, filtered, and concentrated *in vacuo*. The residue was purified by flash column chromatography (20% EtOAc/pentane) to give **54-ox** (11 mg, 67%) as a colorless oil. All recorded spectroscopic data matched those previously reported in the literature.

R*f* (**54-ox**) = 0.2 (1:10 EtOAc/pentane, CAM)

NMR Spectroscopy (*see spectra*):

^{1}H NMR (400 MHz, $CDCl_3$) δ_H 4.46 (d, *J* = 9.2 Hz, 1H), 2.17 – 2.09 (m, 1H), 2.08 – 2.00 (m, 1H), 1.83 – 1.73 (m, 1H), 1.69 – 1.59 (m, 1H), 1.56 – 1.52 (m, 1H), 1.51 – 1.45 (m, 1H), 1.35 – 1.30 (m, 1H), 1.29 (s, 3H), 1.21 (s, 3H), 1.07 (s, 3H) ppm;

^{13}C NMR (101 MHz, $CDCl_3$) δ_C 73.4, 71.1, 65.4, 43.0, 40.5, 31.2, 29.1, 28.5, 27.3, 14.0 ppm.

All recorded spectroscopic data matched those previously reported in the literature.[23]

Dimethoxy bisphenol A boronic acid pinacol ester (55)

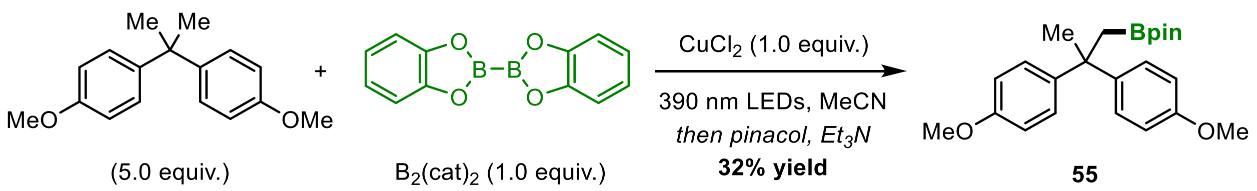

Prepared following **General Procedure B**, using dimethoxy bisphenol A, **55-S** (384 mg, 1.50 mmol, 5.00 equiv.), $CuCl_2$ (40 mg, 0.30 mmol, 1.0 equiv.) and B_2cat_2 (71 mg, 0.30 mmol, 1.0 equiv.) in MeCN (3.0 mL, *c* = 0.10 M). Biotage Isolera™ flash purification on silica gel (SNAP 10 g silica cartridge), eluting with Et_2O/pentane (0 – 5%, v/v), gave **55** (36.4 mg, **32%**) as a colorless oil. Unreacted alkane **55-S** was recovered in 90% yield (323 mg).

Prepared following **General Procedure B-prestirring**, boronic ester **55** was isolated in 17% yield (19 mg).

R*f* = 0.25 (1:20 EtOAc/pentane, CAM)

NMR Spectroscopy (*see spectra*):

^{1}H NMR (400 MHz, $CDCl_3$): δ_H 7.16 (d, *J* = 8.8 Hz, 4H), 6.79 (d, *J* = 8.8 Hz, 4H), 3.78 (s, 6H), 1.76 (s, 3H), 1.68 (s, 2H), 1.03 (s, 12H) ppm.

^{13}C NMR (101 MHz, $CDCl_3$): δ_C 157.4, 144.0, 128.1, 113.2, 82.9, 55.4, 43.3, 30.5, 24.8 ppm; The carbon attached to boron was not observed due to quadrupolar relaxation.

^{11}B NMR (128 MHz, $CDCl_3$): δ_B 33.7 (brs, 1B) ppm.

IR (film): ν_{max} 2923, 2965, 1280, 1257, 763, 749 cm^{-1}.

HRMS (EI^+): m/z calc'd for $C_{23}H_{31}BO_4$ $[M]^+$, 382.3210; found, 382.2307.

Clofibric derivative pinacol bronic ester derivative (56)

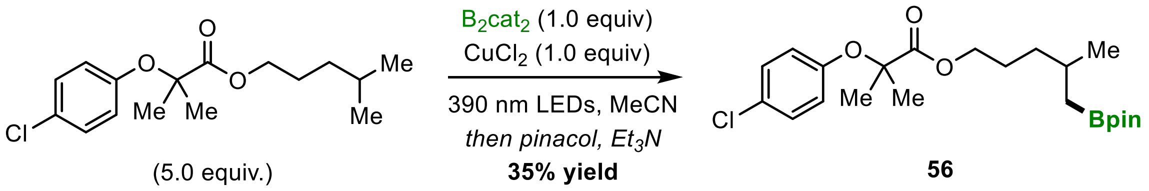

Prepared following **General Procedure B**, using clofibric ester derivative **56-S** (448 mg, 1.50 mmol, 5.00 equiv.), $CuCl_2$ (40 mg, 0.30 mmol, 1.0 equiv.) and B_2cat_2 (71 mg, 0.30 mmol, 1.0 equiv.) in MeCN (1.5 mL, *c* = 0.20 M). Flash column chromatography eluting with EtOAc /pentane (0 – 5%, v/v) on silica gel gave **56** (45 mg, **35%**) as a colorless oil. The r.r. (>97:3) was determined by ^1H NMR analysis of the purified product.

Prepared following **General Procedure B-prestirring**, boronic ester **56** was isolated in 17% yield (22 mg).

R_f = 0.20 (1:10 EtOAc/pentane, CAM)

NMR Spectroscopy (*see spectra*):

^{1}**H NMR** (400 MHz, $CDCl_3$) δ_H 7.20 – 7.15 (m, 2H), 6.80 – 6.74 (m, 2H), 4.12 (td, *J* = 6.8, 1.2 Hz, 2H), 1.70 – 1.58 (m, 3H), 1.57 (s, 6H), 1.23 (s, 12H), 1.21 – 1.10 (m, 2H), 0.88 (d, *J* = 6.8 Hz, 3H), 0.79 (dd, *J* = 15.6, 5.6 Hz, 1H), 0.61 (dd, *J* = 15.6, 8.4 Hz, 1H) ppm;

^{13}**C NMR** (101 MHz, $CDCl_3$) δ_C 174.1, 154.2, 129.2, 127.3, 120.6, 83.0, 79.6, 66.1, 35.6, 29.2, 26.4, 25.5, 25.5, 25.0, 24.9, 24.7, 22.3 ppm;

^{11}**B NMR** (128 MHz, $CDCl_3$) δ_B 33.7 ppm.

IR (film) ν_{max}: 2922, 1710, 1360, 1220, 1143, 529 cm^{-1}.

HRMS (ESI^+) calcd. for $C_{22}H_{34}O_5BCl$ $[M+Na]^+$, 447.2080, found 447.2069.

Triclosan derivative pinacol boronic acid ester (57)

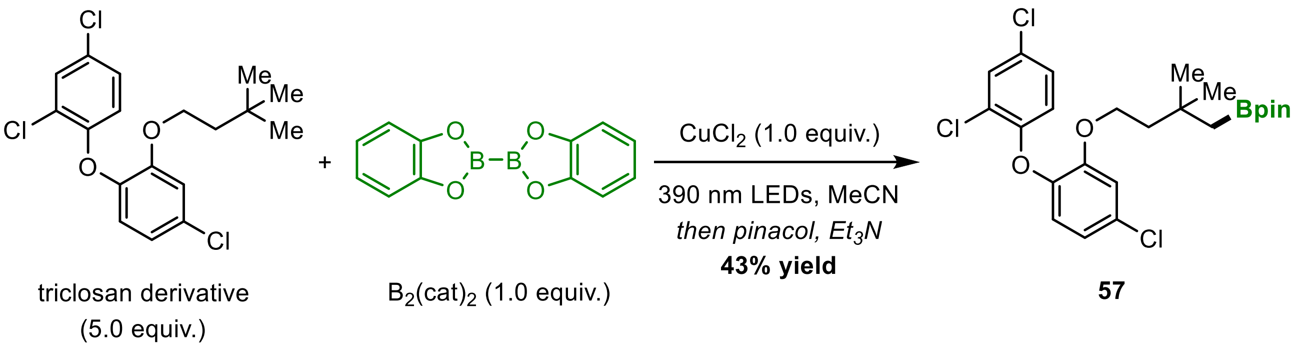

Prepared following **General Procedure B**, using triclosan derivative **57-S** (558 mg, 1.50 mmol, 5.00 equiv.),

$CuCl_2$ (40 mg, 0.30 mmol, 1.0 equiv.) and B_2cat_2 (71 mg, 0.30 mmol, 1.0 equiv.) in MeCN (3.0 mL, *c* = 0.10 M). Biotage Isolera™ flash purification on silica gel (SNAP 10 g silica cartridge), eluting with Et_2O/pentane (0 – 5%, v/v), gave **57** (64 mg, **43%**) as a colorless oil. The r.r. (>97:3) was determined by ^1H NMR analysis of the purified product. Unreacted alkane **57-S** was recovered in 93% yield (482 mg).

Prepared following **General Procedure B-prestirring**, boronic ester **57** was isolated in 35% yield (52 mg).

When the reaction was performed using **3.0 equivalents** of triclosan derivative, **57-S** (335 mg, 0.900 mmol, 3.00 equiv.), boronic ester **57** was isolated in 31% yield (46.9 mg, 0.170 mmol). Unreacted alkane **57-S** was recovered in 86% yield (202 mg).

When the reaction was performed using **1.0 equivalent** of triclosan derivative, **57-S** (102 mg, 0.300 mmol, 1.00 equiv.), $CuCl_2$ (80 mg, 0.60 mmol, 2.0 equiv.), and B_2cat_2 (143 mg, 0.600 mmol, 2.00 equiv.) in MeCN (1.5 mL, *c* = 0.2 M), boronic ester **57** was isolated in 37% yield (55.4 mg). The irradiation time is 60 h. Unreacted alkane **57-S** was recovered in 41% yield (42 mg).

R*f* = 0.45 (1:5 EtOAc/pentane, CAM)

NMR Spectroscopy (*see spectra*):

^{1}H NMR (400 MHz, $CDCl_3$): δ_H 7.41 (d, *J* = 2.4 Hz, 1H), 7.07 (dd, *J* = 8.8, 2.4 Hz, 1H), 6.98 (dd, *J* = 2.0, 0.8 Hz, 1H), 6.95 – 6.85 (m, 2H), 6.62 (d, *J* = 8.8 Hz, 1H), 4.01 (t, *J* = 7.2 Hz, 2H), 1.66 (t, *J* = 7.2 Hz, 2H), 1.22 (s, 12H), 0.95 (s, 6H), 0.78 (s, 2H) ppm;

^{13}C NMR (101 MHz, $CDCl_3$): δ_C 152.7, 151.4, 143.1, 130.7, 130.3, 127.8, 127.7, 124.6, 122.1, 120.8, 118.0, 114.9, 83.1, 67.0, 42.4, 31.6, 29.9, 25.0 ppm; The carbon attached to boron was not observed due to quadrupolar relaxation.

^{11}B NMR (128 MHz, $CDCl_3$): δ_B 33.0 (brs, 1B) ppm.

IR (film): ν_{max} 2955, 1598, 1495, 1473, 1409, 1390, 1191, 1258, 1231, 1114, 1056, 919, 855, 801 cm^{-1}.

HRMS (EI^+): m/z calc'd for $C_{24}H_{30}O_4Cl_3B$ $[M]^+$, 498.1297; found, 498.1296.

Probenecid methyl ester pinacol boronic acid ester (58)

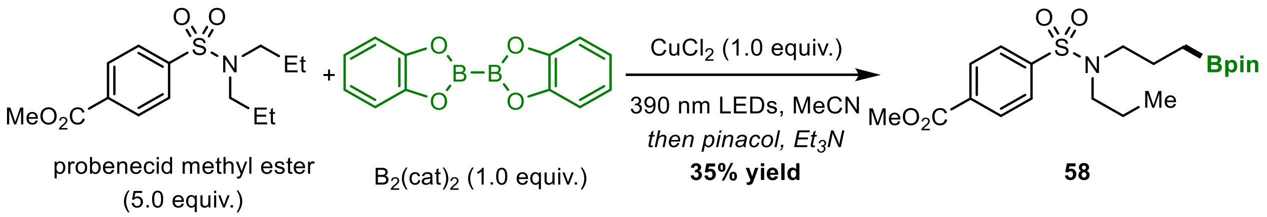

Prepared following **General Procedure B**, using probenecid methyl ester, **58-S** (450 mg, 1.50 mmol, 5.00 equiv.), $CuCl_2$ (40 mg, 0.30 mmol, 1.0 equiv.) and B_2cat_2 (71 mg, 0.30 mmol, 1.0 equiv.) in MeCN (3.0 mL, *c* = 0.10 M). Biotage Isolera™ flash purification on silica gel (SNAP 5 g silica cartridge), eluting with Et_2O/pentane (0 – 5%, v/v), gave **58** (44.4 mg, **35%**) as a colorless oil. The r.r. (>97:3) was determined by ^1H NMR analysis of the purified product. Unreacted alkane was recovered in 88% yield (387 mg).

Prepared following **General Procedure B-prestirring**, boronic ester **58** was isolated in 34% yield (43.0 mg).

When the reaction was performed using **3.0 equivalents** of probenecid methyl ester, **58-S** (0.28 g, 0.90 mmol, 3.0 equiv.), boronic ester was isolated in 31% yield (40.1 mg). Unreacted alkane was recovered in 90% yield (214 mg).

When the reaction was performed using **1.0 equivalent** of probenecid methyl ester, **58-S** (90 mg, 0.30 mmol, 1.0 equiv.), $CuCl_2$ (80 mg, 0.60 mmol, 2.0 equiv.), and B_2cat_2 (143 mg, 0.600 mmol, 2.00 equiv.) in MeCN (1.5 mL, *c* = 0.2 M), boronic ester was isolated in 27% yield (34.1 mg). The irradiation time is 48 h. Unreacted alkane was recovered in 43% yield (38.1 mg).

$\mathbf{R_f}$ = 0.33 (1:9 EtOAc/pentane, CAM)

NMR Spectroscopy (*see spectra*):

^{1}**H NMR** (400 MHz, $CDCl_3$): δ_H 8.14 (d, *J* = 8.8 Hz, 2H), 7.87 (d, *J* = 8.8 Hz, 2H), 3.86 (s, 3H), 3.17 – 3.02 (m, 4H), 1.64 – 1.49 (m, 4H), 1.23 (s, 12H), 0.85 (t, *J* = 7.6 Hz, 3H), 0.71 (t, *J* = 7.6 Hz, 2H) ppm.

^{13}**C NMR** (101 MHz, $CDCl_3$): δ_C 165.9, 144.6, 133.5, 130.3, 127.2, 83.3, 52.7, 50.0, 49.9, 25.0, 23.1, 21.9, 11.3 ppm. The carbon attached to boron was not observed due to quadrupolar relaxation.

^{11}**B NMR** (128 MHz, $CDCl_3$): δ_B 33.3 (brs, 1B) ppm.

IR (film): ν_{max} 2978, 1842, 1730, 1386, 1340, 1278, 1159, 976, 747, 603 cm^{-1}.

HRMS (ESI^+): m/z calc'd for $C_{20}H_{32}BNO_6S$ $[M+H]^+$, 426.2116; found, 426.2128.

D-Galactopyranose derivative pinacol boronic acid ester (59)

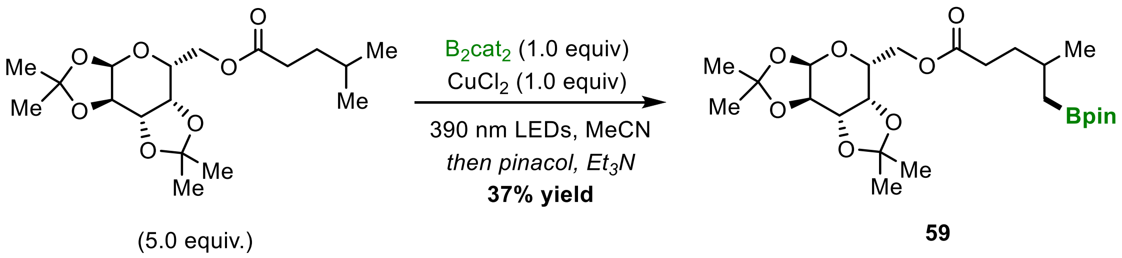

Prepared following **General Procedure B**, using 1,2:3,4-di-*O*-isopropylidene-*α*-*D*-galactopyranose ester derivative **59-S** (538 mg, 1.50 mmol, 5.00 equiv.), $CuCl_2$ (40 mg, 0.30 mmol, 1.0 equiv.) and B_2cat_2 (71 mg, 0.30 mmol, 1.0 equiv.) in MeCN (1.5 mL, *c* = 0.20 M). Flash column chromatography eluting with EtOAc/pentane (0 – 10%, v/v) on silica gel gave **59** (42 mg, 30%) as a colorless oil. The r.r. (>97:3) was determined by GC-FID analysis of the crude reaction mixture (***see spectrum***). The d.r. (60:40) was determined by ^{1}H NMR analysis of the purified product.

Prepared following **General Procedure B-prestirring**, boronic ester **59** was isolated in **37%** yield (54 mg).

$\mathbf{R_f}$ = 0.30 (1:10 EtOAc/pentane, CAM)

NMR Spectroscopy (*see spectra*):

^{1}H NMR (400 MHz, $CDCl_3$) δ_H 5.53 (d, *J* = 5.2 Hz, 1H), 4.61 (dd, *J* = 8.0, 2.4 Hz, 1H), 4.34 – 4.28 (m, 2H), 4.23 (dd, *J* = 8.0, 2.0 Hz, 1H), 4.16 (dd, *J* = 11.6, 8.0 Hz, 1H), 4.04 – 3.98 (m 1H), 2.39 – 2.30 (m, 2H), 1.79 – 1.50 (m, 3H), 1.50 (s, 3H), 1.45 (s, 3H), 1.34 (s, 3H), 1.33 (s, 3H), 1.23 (s, 12H), 0.92 (d, *J* = 6.4 Hz, 3H), 0.84 (dd, *J* = 15.6, 5.6 Hz, 1H), 0.65 (ddd, *J* = 15.6, 8.8, 1.2 Hz, 1H).

^{13}C NMR (101 MHz, $CDCl_3$) δ_C 174.1, 109.8, 108.9, 96.4, 83.1, 71.2, 70.8, 70.6, 66.17 + 66.15 (diastereomeric peaks), 63.3, 34.5, 32.37 + 32.35 (diastereomeric peaks), 29.32 + 29.27 (diastereomeric peaks), 26.2, 26.1, 25.1, 25.0, 24.9, 24.6, 21.98 + 21.96 (diastereomeric peaks) ppm; The carbon attached to boron was not observed due to quadrupolar relaxation.

All recorded spectroscopic data matched those previously reported in the literature.[3]

Flurbiprofen derivative pinacol boronic acid ester (60)

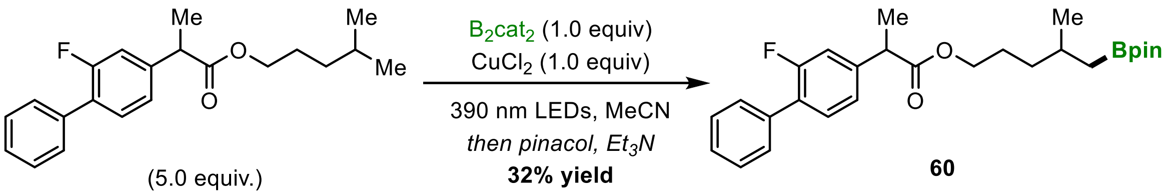

Prepared following **General Procedure B**, using flurbiprofen derivative **60-S** (493 mg, 1.50 mmol, 5.00 equiv.), $CuCl_2$ (40 mg, 0.30 mmol, 1.0 equiv.) and B_2cat_2 (71 mg, 0.30 mmol, 1.0 equiv.) in MeCN (1.5 mL, *c* = 0.20 M). Flash column chromatography eluting with Et_2O/pentane (0 – 3%, v/v) on silica gel gave **60** (40 mg, 30%) as a colorless oil. The r.r. (>97:3) was determined by GC-FID analysis of the crude reaction mixture (***see spectrum***).

Prepared following **General Procedure B-prestirring**, boronic ester **60** was isolated in **32%** yield (43 mg).

R_f = 0.30 (1:20 EtOAc/pentane, CAM)

NMR Spectroscopy (*see spectra*):

^{1}H NMR (400 MHz, $CDCl_3$) δ_H 7.56 – 7.50 (m, 2H), 7.46 – 7.33 (m, 4H), 7.18 – 7.10 (m, 2H), 4.08 (tt, *J* = 6.8, 2.4 Hz, 2H), 3.74 (q, *J* = 7.2 Hz, 1H), 1.73 – 1.58 (m, 2H), 1.53 (d, *J* = 7.2 Hz, 3H), 1.33 – 1.13 (m, 3H) 1.23 (s, 12H), 0.89 (dd, *J* = 6.8, 1.6 Hz, 3H), 0.86 – 0.77 (m, 1H), 0.63 (dd, *J* = 15.6, 8.4 Hz, 1H) ppm;

^{13}C NMR (101 MHz, $CDCl_3$) δ_C 174.2, 159.8 (d, *J* = 249.5 Hz), 142.2 (d, *J* = 7.1 Hz), 135.7, 130.9 (d, *J* = 4.0 Hz), 129.1 (d, *J* = 3.0 Hz), 128.6, 127.9 (d, *J* = 3.0 Hz), 127.7, 123.7 (d, *J* = 4.0 Hz), 115.4 (d, *J* = 24.2 Hz), 83.0, 65.6, 65.6, 45.3 (d, *J* = 2.0 Hz), 35.7, 29.3, 26.5, 25.0, 24.9, 22.4, 18.5 ppm; The carbon attached to boron was not observed due to quadrupolar relaxation.

^{19}F NMR (377 MHz, $CDCl_3$) δ_F –117.7 ppm;

^{11}B NMR (128 MHz, $CDCl_3$) δ_B 34.5 ppm.

IR (film) ν_{max}: 2977, 1733, 1370, 1316, 1144, 969, 766, 698 cm^{-1}.

HRMS (ESI^+) calcd. for $C_{27}H_{36}O_4BF$ $[M+H]^+$, 455.2763, found 455.2794.

Saccharine derivative pinacol boronic acid ester (61)

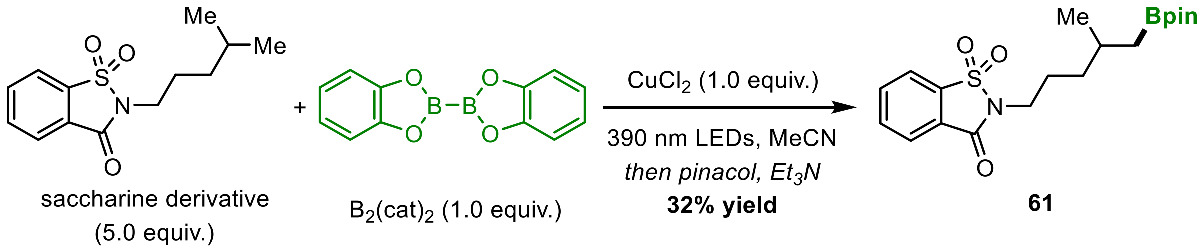

Prepared following **General Procedure B**, using saccharine derivative **61-S** (400 mg, 1.50 mmol, 5.00 equiv.), $CuCl_2$ (40 mg, 0.30 mmol, 1.0 equiv.) and B_2cat_2 (71 mg, 0.30 mmol, 1.0 equiv.) in MeCN (3.0 mL, *c* = 0.10 M). Biotage Isolera^{TM} flash purification on silica gel (SNAP 10 g silica cartridge), eluting with Et_2O/pentane (0 – 10%, v/v), gave **63** (36.4 mg, 31%) as a colorless oil. The r.r (>97:3). was determined by GC-FID analysis of the crude reaction mixture (***see spectrum***). Unreacted alkane **61-S** was recovered in 88% yield (330 mg).

Prepared following **General Procedure B-prestirring**, boronic ester **61** was isolated in **32%** yield (37.3 mg).

R*f* = 0.30 (1:5 EtOAc/pentane, CAM)

NMR Spectroscopy (*see spectra*):

^{1}**H NMR** (400 MHz, $CDCl_3$): δ_H 8.09 – 8.00 (m, 1H), 7.94 – 7.87 (m, 1H), 7.90 – 7.77 (m, 2H), 3.74 (td, *J* = 7.6, 1.2 Hz, 2H), 1.94 – 1.80 (m, 2H), 1.85 – 1.70 (m, 1H), 1.45 – 1.26 (m, 2H), 1.22 (s, 12H), 0.93 (d, *J* = 6.8 Hz, 3H), 0.85 (dd, *J* = 15.6, 5.6 Hz, 1H), 0.67 (dd, *J* = 15.6, 8.4 Hz, 1H) ppm;

^{13}**C NMR** (101 MHz, $CDCl_3$): δ_C 159.0, 137.9, 134.7, 134.4, 127.7, 125.2, 121.0, 83.0, 39.9, 36.5, 29.3, 26.4, 25.0, 24.9, 22.3 ppm. The carbon attached to boron was not observed due to quadrupolar relaxation.

^{11}**B NMR** (128 MHz, $CDCl_3$): δ_B 33.8 (brs, 1B) ppm.

IR (film): ν_{max} 2950, 1732, 1335, 1259, 1184, 751, 587 cm^{-1}.

HRMS (ESI^+): m/z calc'd for $C_{19}H_{28}BNO_5S$ $[M+H]^+$, 394.1854; found, 394.1857.

Naproxen derivative pinacol boronic acid ester (62)

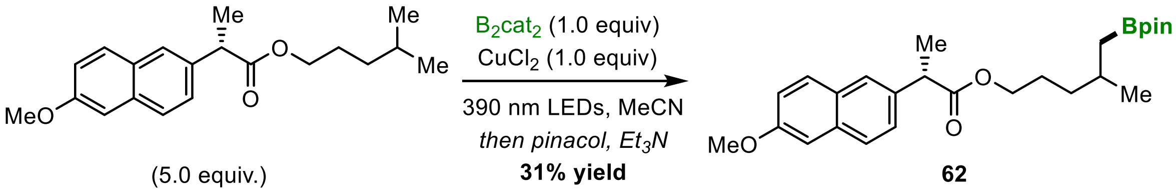

Prepared following **General Procedure B**, using naproxen ester derivative **62-S** (471 mg, 1.50 mmol, 5.00 equiv.), $CuCl_2$ (40 mg, 0.30 mmol, 1.0 equiv.) and B_2cat_2 (71 mg, 0.30 mmol, 1.0 equiv.) in MeCN (1.5 mL, *c* = 0.20 M). Flash column chromatography eluting with EtOAc/pentane (0 – 10%, v/v) on silica gel gave **62** (41 mg, **31%**) as a colorless oil. The r.r. (>97:3) was determined by GC-FID analysis of the crude reaction mixture (***see spectrum***). Unreacted alkane **62-S** was recovered in 85% yield (377 mg).

Prepared following **General Procedure B-prestirring**, boronic ester **62** was isolated in 27% yield (36 mg).

When the reaction was performed using 3.00 equivalents of naproxen ester derivative (283 mg, 0.900 mmol), boronic ester **62** was isolated in 20% yield (26 mg).

When the reaction was performed using 1.00 equivalents of naproxen ester derivative (283 mg, 0.90 mmol) and $CuCl_2$ (80 mg, 0.60 mmol, 2.0 equiv.) and B_2cat_2 (143 mg, 0.600 mmol, 2.00 equiv.) in MeCN (1.5 mL, *c* = 0.20 M), boronic ester **62** was isolated in 17% yield (22 mg).

$\mathbf{R_f}$ = 0.20 (1:10 EtOAc/pentane, CAM)

NMR Spectroscopy (*see spectra*):

^{1}H NMR (400 MHz, $CDCl_3$) δ_H 7.66 – 7.54 (m, 3H), 7.33 (dd, *J* = 8.4, 2.0 Hz, 1H), 7.09 – 7.00 (m, 2H), 4.03 – 3.91 (m, 2H), 3.83 (s, 3H), 3.76 (q, *J* = 7.1 Hz, 1H), 1.62 – 1.42 (m, 6H), 1.15 (s, 12H), 1.10 – 0.98 (m, 2H), 0.78 (dd, *J* = 6.8, 2.8 Hz, 3H), 0.73 – 0.63 (m, 1H), 0.52 (dd, *J* = 15.6, 8.4 Hz, 1H) ppm;

^{13}C NMR (101 MHz, $CDCl_3$) δ_C 174.8, 157.7, 136.0, 133.8, 129.4, 129.1, 127.2, 126.4, 126.0, 119.0, 105.7, 105.6, 83.0, 65.4, 65.4, 55.4, 45.7, 35.64, 35.63, 29.28, 29.27, 26.5, 25.0, 24.9, 22.3, 18.7 ppm; The carbon attached to boron was not observed due to quadrupolar relaxation.

^{11}B NMR (128 MHz, $CDCl_3$) δ_B 33.9 ppm.

IR (film) v_{max}: 2960, 1729, 1606, 1368, 1175, 1144, 1032, 848, 475 cm^{-1}.

HRMS (ESI^+) calcd. for $C_{26}H_{37}O_5B$ $[M+H]^+$, 441.2807, found 441.2806.

Oxaprozin derivative pinacol bronic ester derivative (63)

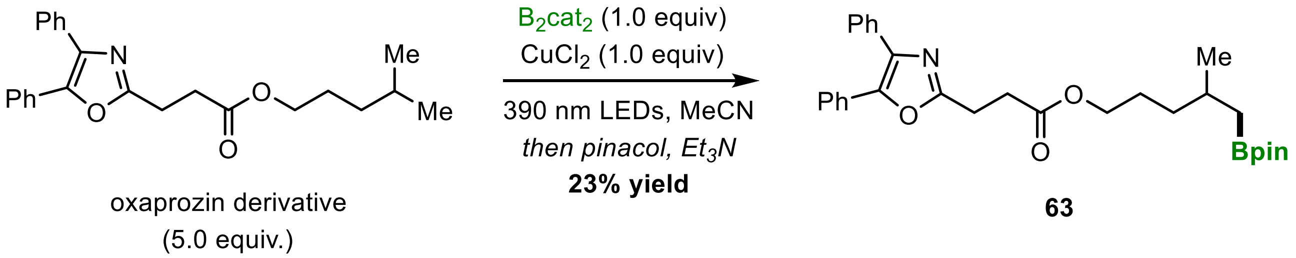

Prepared following **General Procedure B**, using oxaprozin ester derivative **63-S** (566 mg, 1.50 mmol, 5.00 equiv.), $CuCl_2$ (40 mg, 0.30 mmol, 1.0 equiv.) and B_2cat_2 (71 mg, 0.30 mmol, 1.0 equiv.) in MeCN (1.5 mL, *c* = 0.20 M). Flash column chromatography eluting with EtOAc /pentane (0 – 30%, v/v) on silica gel gave **63** (34 mg, **23%**) as a colorless oil. The r.r. (>97:3) was determined by ^{1}H NMR analysis of the purified product.

$\mathbf{R_f}$ = 0.30 (1:5 EtOAc/pentane, CAM)

NMR Spectroscopy (*see spectra*):

^{1}H NMR (400 MHz, $CDCl_3$) δ_H 7.67 – 7.59 (m, 2H), 7.61 – 7.53 (m, 2H), 7.40 – 7.26 (m, 6H), 4.10 (td, *J* = 6.8, 1.2 Hz, 2H), 3.18 (dd, *J* = 8.4, 6.8 Hz, 2H), 2.90 (dd, *J* = 8.4, 6.8 Hz, 2H), 1.75 – 1.57 (m, 3H), 1.39 – 1.16 (m, 2H), 1.24 (s, 12H), 0.91 (d, *J* = 6.4 Hz, 3H), 0.83 (dd, *J* = 15.2, 5.6 Hz, 1H), 0.65 (dd, *J* = 15.2, 8.4

Hz, 1H) ppm;

^{13}C NMR (101 MHz, $CDCl_3$) δ_C 172.2, 162.0, 145.5, 135.3, 132.6, 129.2, 128.8, 128.7, 128.6, 128.2, 128.0, 126.6, 83.0, 65.4, 35.7, 31.3, 29.3, 26.6, 25.0, 24.9, 23.7, 22.3 ppm; The carbon attached to boron was not observed due to quadrupolar relaxation.

^{11}B NMR (128 MHz, $CDCl_3$) δ_B 33.4 ppm.

IR (film) ν_{max}: 2919, 1712, 1450, 1371, 1241, 1146, 1015, 847 cm^{-1}.

HRMS (ESI$^+$) calcd. for $C_{30}H_{38}NO_5B$ [M+H]$^+$, 504.2843, found 504.2906.

Lorzone derivative pinacol boronic acid ester (64)

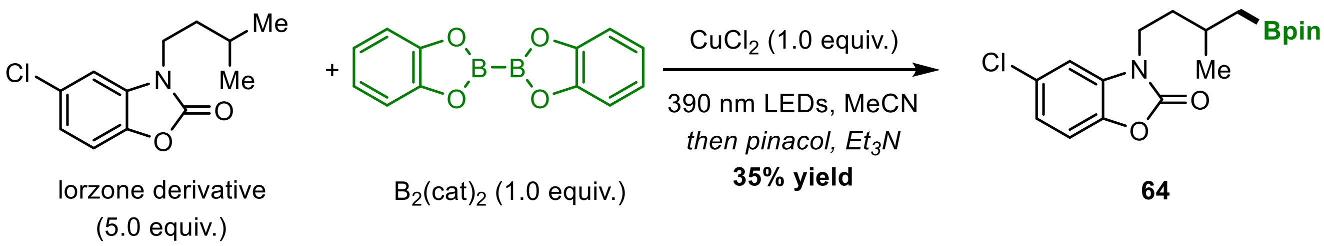

Prepared following **General Procedure B**, using lorzone derivative **64-S** (360 mg, 1.50 mmol, 5.00 equiv.), $CuCl_2$ (40 mg, 0.30 mmol, 1.0 equiv.) and B_2cat_2 (71 mg, 0.30 mmol, 1.0 equiv.) in MeCN (1.5 mL, *c* = 0.20 M). Biotage Isolera^{TM} flash purification on silica gel (SNAP 10 g silica cartridge), eluting with Et_2O/pentane (0 – 10%, v/v), gave **64** (38 mg, **34%**) as a colorless oil. The r.r. (>97:3) was determined by ^{1}H NMR analysis of the purified product. Unreacted alkane **64-S** was recovered in 86% yield (286 mg).

R*f* = 0.40 (1:10 EtOAc/pentane, CAM)

NMR Spectroscopy (*see spectra*):

^{1}H NMR (400 MHz, $CDCl_3$) δ_H 7.04 (dd, *J* = 8.4, 0.8 Hz, 2H), 6.99 (dd, *J* = 8.4, 2.0 Hz, 2H), 6.96 – 6.90 (m, 1H), 3.74 (dd, *J* = 8.0, 6.4 Hz, 2H), 1.81 – 1.67 (m, 2H), 1.58 – 1.48 (m, 1H), 1.16 (s, 12H), 0.97 (d, *J* = 6.4 Hz, 3H), 0.83 (dd, *J* = 15.6, 6.0 Hz, 1H), 0.73 (dd, *J* = 15.6, 6.8 Hz, 1H) ppm;

^{13}C NMR (101 MHz, $CDCl_3$) δ_C 154.4, 141.4, 132.4, 129.4, 122.2, 110.9, 109.0, 83.3, 41.1, 36.4, 27.3, 25.0, 24.9, 22.3 ppm; The carbon attached to boron was not observed due to quadrupolar relaxation.

^{11}B NMR (128 MHz, $CDCl_3$): δ_B 33.3 ppm.

IR (film) ν_{max}: 2978, 2872, 1772, 1487, 1369, 1254, 1144, 847, 807, 750 cm^{-1}.

HRMS (ESI$^+$): m/z calc'd for $C_{18}H_{25}BNO_4Cl$ [M+H]$^+$, 365.1565; found, 365.1549.

Ciprofibrate derivative pinacol bronic ester derivative (65)

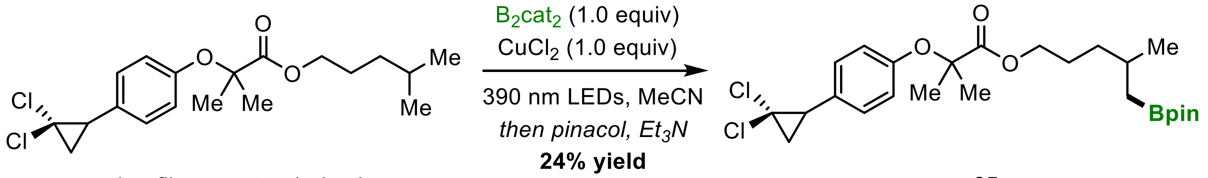

ciprofibrate ester derivative
(5.0 equiv.)

65

Prepared following **General Procedure B**, using ciprofibrate ester derivative **65-S** (560 mg, 1.50 mmol, 5.00 equiv.), $CuCl_2$ (40 mg, 0.30 mmol, 1.0 equiv.) and B_2cat_2 (71 mg, 0.30 mmol, 1.0 equiv.) in MeCN (1.5 mL, *c* = 0.20 M). Flash column chromatography eluting with EtOAc /pentane (0 – 10%, v/v) on silica gel gave **65** (36 mg, **24%**) as a colorless oil. The r.r. (>97:3) was determined by ^{1}H NMR analysis of the purified product. Unreacted alkane **65-S** was recovered in 76% yield (408 mg).

R_f = 0.20 (1:9 EtOAc/pentane, CAM)

NMR Spectroscopy (*see spectra*):

^{1}H NMR (400 MHz, $CDCl_3$) δ_H 7.13 – 7.07 (m, 2H), 6.83 – 6.78 (m, 2H), 4.13 (t, *J* = 6.8 Hz, 2H), 2.83 (dd, *J* = 10.8, 8.4 Hz, 1H), 1.96 – 1.89 (m, 1H), 1.80 – 1.73 (m, 1H), 1.69 – 1.54 (m, 3H), 1.59 (s, 6H), 1.23 (s, 12H), 1.21 – 1.09 (m, 2H), 0.87 (d, *J* = 6.8 Hz, 3H), 0.82 – 0.74 (m, 1H), 0.65 – 0.55 (m, 1H) ppm;

^{13}C NMR (101 MHz, $CDCl_3$) δ_C 174.4, 155.1, 129.8, 128.2, 118.7, 83.0, 79.3, 66.1, 61.0, 35.6, 35.0, 29.3, 26.4, 26.0, 25.6, 25.6, 25.0, 24.9, 22.3 ppm; The carbon attached to boron was not observed due to quadrupolar relaxation.

^{11}B NMR (128 MHz, $CDCl_3$) δ_B 33.4 ppm.

IR (film) ν_{max}: 2978, 1732, 1511, 1370, 1140, 968, 846, 760 cm^{-1}.

HRMS (ESI$^+$) calcd. for $C_{25}H_{37}O_5BCl_2$ $[M+H]^+$, 499.2111, found 499.2173.

Cinnamic acid derivative pinacol boronic acid ester (66)

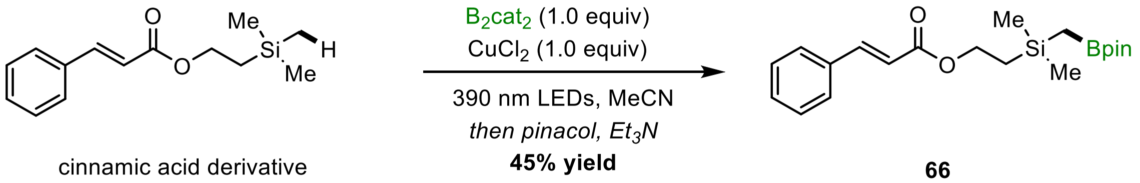

cinnamic acid derivative
(5 equiv.)

66

Prepared following **General Procedure B**, using cinnamic acid derivative (372 mg, 1.50 mmol, 5.00 equiv.), $CuCl_2$ (40 mg, 0.30 mmol, 1.0 equiv.) and B_2cat_2 (71 mg, 0.30 mmol, 1.0 equiv.) in MeCN (1.5 mL, *c* = 0.10 M). Biotage Isolera™ flash purification on silica gel (SNAP 10 g silica cartridge), eluting with Et_2O/pentane (0 – 10%, v/v), gave **66** (50.3 mg, **45%**) as a colorless oil. The r.r. (>97:3) was determined by ^{1}H NMR analysis of the purified product. Unreacted alkane was recovered in 98% yield (335 mg).

R_f = 0.45 (1:10 EtOAc/pentane, CAM)

NMR Spectroscopy (*see spectra*):

^{1}H NMR (400 MHz, $CDCl_3$): δ_H 7.67 (d, *J* = 16.0 Hz, 1H), 7.53– 7.49 (m, 1H), 7.41 – 7.29 (m, 3H), 6.43 (d, *J* = 16.0 Hz, 1H), 4.37 – 4.28 (m, 2H), 1.24 (s, 12H), 1.17 – 1.07 (m, 2H), 0.15 (s, 3H), 0.12 (s, 6H) ppm;

^{13}C NMR (101 MHz, $CDCl_3$): δ_C 167.3, 144.5, 134.7, 130.3, 128.2, 118.6, 83.1, 62.8, 25.1, 17.9, –1.00.ppm; The carbon attached to boron was not observed due to quadrupolar relaxation.

All recorded spectroscopic data matched those previously reported in the literature.[3]

Celecoxib derivative pinacol boronic acid ester (67)

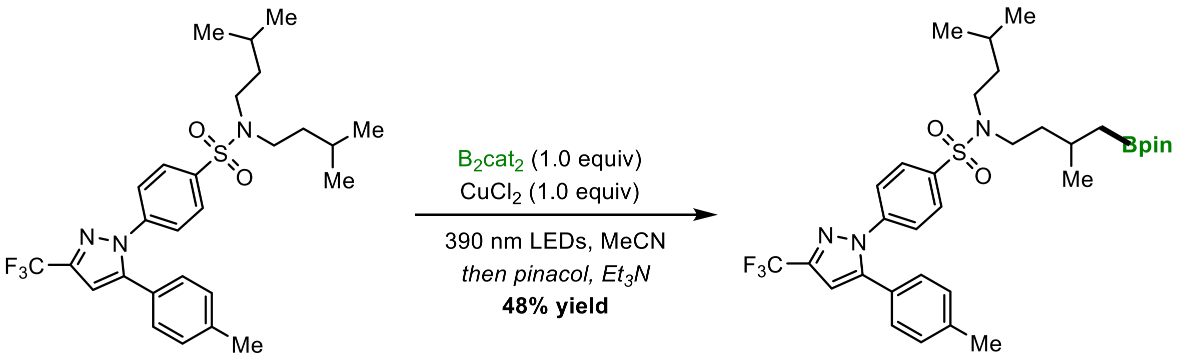

67

Prepared following **General Procedure B**, using celecoxib derivative **67-S** (783 mg, 1.50 mmol, 5.00 equiv.), $CuCl_2$ (40 mg, 0.30 mmol, 1.0 equiv.) and B_2cat_2 (71 mg, 0.30 mmol, 1.0 equiv.) in MeCN (3.0 mL, *c* = 0.10 M). Biotage Isolera™ flash purification on silica gel (SNAP 25 g silica cartridge), eluting with EtOAc/pentane (2 – 20%, v/v), gave **67** (92.4 mg, **48%**) as a colorless oil. The r.r. (>97:3) was determined by ^{1}H NMR analysis of the purified product. Unreacted alkane **67-S** was recovered in 86% yield (608 mg).

R*f* = 0.26 (1:10 EtOAc/pentane, CAM)

NMR Spectroscopy (*see spectra*):

^{1}H NMR (400 MHz, $CDCl_3$): δ_H 7.89 – 7.72 (m, 2H), 7.52 – 7.38 (m, 2H), 7.16 (d, *J* = 8.2 Hz, 2H), 7.09 (d, *J* = 8.2 Hz, 2H), 6.73 (s, 1H), 3.23 – 3.06 (m, 4H), 2.37 (s, 3H), 1.75 – 1.61 (m, 1H), 1.61 – 1.45 (m, 2H), 1.44 – 1.31 (m, 3H), 1.22 (s, 12H), 0.90 (d, *J* = 6.6 Hz, 3H), 0.88 (d, *J* = 6.6 Hz, 6H), 0.80 (dd, *J* = 15.5, 5.8 Hz, 1H), 0.65 (dd, *J* = 15.5, 8.2 Hz, 1H) ppm;

^{13}C NMR (101 MHz, $CDCl_3$): δ_C 145.3, 144.1 (q, *J* = 38.6 Hz), 142.2, 139.9 (d, *J* = 9.8 Hz), 129.8, 128.8, 128.2, 125.8, 125.6, 121.2 (q, *J* = 269.1 Hz), 106.3, 46.4, 83.1, 46.4, 37.7, 37.2, 27.5, 26.0, 25.0, 24.9, 22.5, 22.4, 22.2, 21.4 ppm; The carbon attached to boron was not observed due to quadrupolar relaxation.

^{19}F NMR (377 MHz, $CDCl_3$): δ_F – 62.43 (s, CF_3);

^{11}B NMR (128 MHz, $CDCl_3$) δ_B 33.2 ppm.

All recorded spectroscopic data matched those previously reported in the literature.[6]

2-(2,5-dimethylhexyl)-4,4,5,5-tetramethyl-1,3,2-dioxaborolane (77)

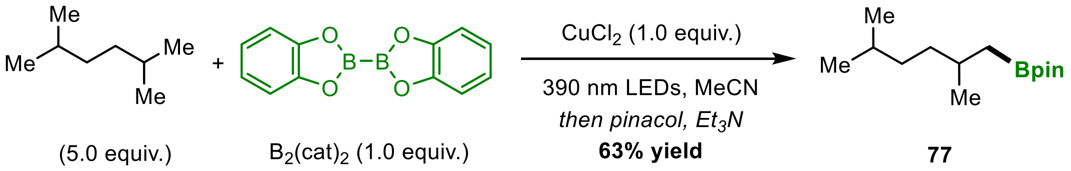

Prepared following **General Procedure B**, using using 2,5-dimethylhexane (117 mg, 1.50 mmol, 5.00 equiv.), $CuCl_2$ (40 mg, 0.30 mmol, 1.0 equiv.) and B_2cat_2 (71 mg, 0.30 mmol, 1.0 equiv.) in MeCN (1.5 mL, *c* = 0.20 M). Biotage Isolera™ flash purification on silica gel (SNAP 5 g silica cartridge), eluting with Et_2O/pentane (0 – 5%, v/v), gave **77** (35 mg, 49%) as a colorless oil. The r.r. (96:4) was determined by GC-FID analysis of the crude reaction mixture (***see spectrum***).

Prepared following **General Procedure B-prestirring**, boronic ester **77** was isolated in **63%** yield (45 mg).

R*f* = 0.35 (1:10 EtOAc/pentane, CAM)

NMR Spectroscopy (*see spectra*):

^{1}H NMR (400 MHz, $CDCl_3$): δ_H 1.73 – 1.57 (m, 1H), 1.53 – 1.42 (m, 1H), 1.24 (s, 12H), 1.20 – 1.11 (m, 3H), 0.90 (d, *J* = 6.6 Hz, 3H), 0.86 (d, *J* = 6.6 Hz, 6H), 0.82 (dd, *J* = 15.4, 5.9 Hz, 1H), 0.65 (dd, *J* = 15.3, 8.2 Hz, 1H) ppm;

^{13}C NMR (101 MHz, $CDCl_3$): δ_C 83.0, 37.4, 36.8, 29.9, 28.4, 25.0, 24.9, 22.9, 22.8, 22.6 ppm. The carbon attached to boron was not observed due to quadrupolar relaxation.

All recorded spectroscopic data matched those previously reported in the literature.[3]

2-(2-(4-(*tert*-Butyl)phenyl)-2-methylpropyl)-4,4,5,5-tetramethyl-1,3,2-dioxaborolane (78)

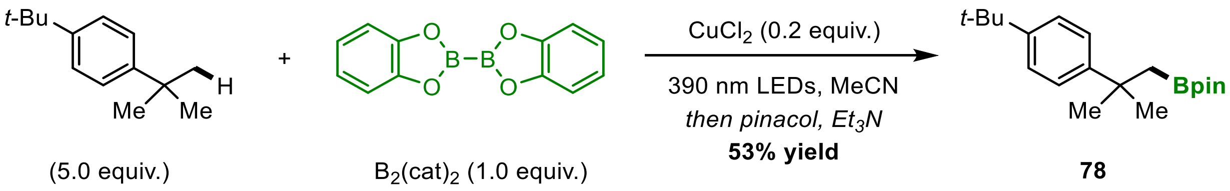

Prepared following **General Procedure A**, using 1,4-di-*tert*-butylbenzene (285 mg, 1.50 mmol, 5.00 equiv.), $CuCl_2 \cdot 2H_2O$ (10 mg, 0.060 mmol, 0.20 equiv.), and B_2cat_2 (71 mg, 0.30 mmol, 1.0 equiv.) in MeCN (1.5 mL, *c* = 0.20 M). Flash column chromatography, eluting with EtOAc/hexane (0 – 5%, v/v) gave **78** (27 mg, 29%) as a colorless oil.

Prepared following **General Procedure A-prestirring**, boronic ester **78** was isolated in **53%** yield (50 mg).

R*f* = 0.40 (1:19 EtOAc/hexane, CAM)

NMR Spectroscopy (*see spectra*):

^{1}H NMR (400 MHz, $CDCl_3$) δ_H 7.35 – 7.26 (m, 4H), 1.41 (s, 6H), 1.30 (s, 9H), 1.23 (s, 2H), 1.08 (s, 12H).

^{13}C NMR (101 MHz, $CDCl_3$) δ_C 148.4, 148.0, 125.3, 124.8, 82.8, 36.0, 34.3, 31.5, 31.4, 24.8 ppm. The carbon attached to boron was not observed due to quadrupolar relaxation.

All recorded spectroscopic data matched those previously reported in the literature.[3]

2-(2-(3-(*tert*-Butyl)phenyl)-2-methylpropyl)-4,4,5,5-tetramethyl-1,3,2-dioxaborolane (79)

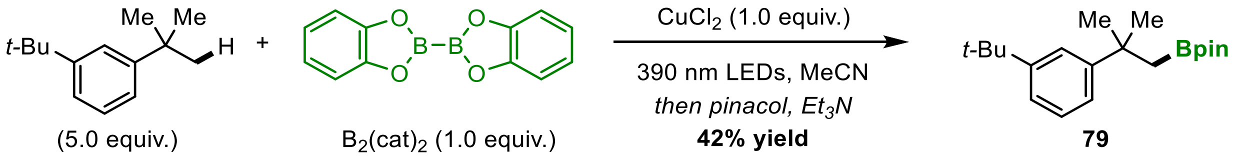

Prepared following **General Procedure B**, using 1,3-di-*tert*-butylbenzene (285 mg, 332 µL, 1.50 mmol, 5.00 equiv.), $CuCl_2$ (40 mg, 0.30 mmol, 1.0 equiv.), and B_2cat_2 (71 mg, 0.30 mmol, 1.0 equiv.) in MeCN (1.5 mL, *c* = 0.20 M). Flash column chromatography, eluting with EtOAc/hexane (0 – 5%, v/v) gave **79** (40 mg, **42%**) as a colorless oil.

Prepared following **General Procedure B-prestirring**, boronic ester **79** was isolated in 37% yield (35 mg).

R_f = 0.34 (1:19 EtOAc/hexane, CAM)

NMR Spectroscopy (*see spectra*):

^{1}H NMR (400 MHz, CDCl3) δ_H 7.45 – 7.42 (m, 1H), 7.23 – 7.19 (m, 2H), 7.19 – 7.15 (m, 1H), 1.42 (s, 6H), 1.32 (s, 9H), 1.26 (s, 2H), 1.09 (s, 12H) ppm ;

^{13}C NMR (101 MHz, CDCl3) δ_C 151.1, 150.4, 127.5, 122.8, 122.6, 122.3, 82.8, 36.6, 34.9, 31.6, 31.6, 24.9 ppm. The carbon attached to boron was not observed due to quadrupolar relaxation.

^{11}B NMR (128 MHz, $CDCl_3$) δ_B 33.1 ppm.

IR (film): 2961, 2869, 2245, 1511, 1465, 1420, 1389, 1379, 1354, 1322, 1272, 1227, 1214, 1143, 1119, 971, 910, 846, 831, 733, 579 cm^{-1}.

HRMS (EI^+): m/z calculated for $C_{20}H_{33}BO_2$ $[M]^+$, 316.2568; found, 316.2564.

2.6. Scale-up Reaction

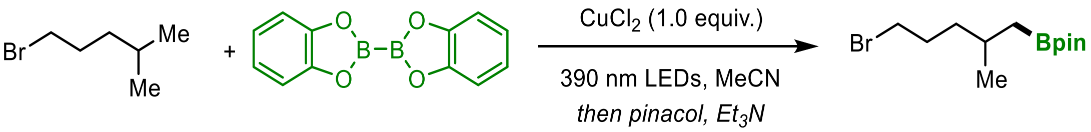

(5.0 equiv.) 3.0 mmol $B_2(cat)_2$ (1.0 equiv.) **19**

Under an ambient atmosphere, a flame dried 25 mL Biotage® microwave reaction vial equipped with a magnetic stir bar was charged with $CuCl_2$ (400 mg, 3.00 mmol, 1.00 equiv.) and B_2cat_2 (713 mg, 3.00 mmol, 1.00 equiv.). The vial was transferred into an anhydrous, argon-filled glovebox where anhydrous acetonitrile was added (15 mL, *c* = 0.20 M) and the vial was sealed by a Suba Seal septum. After stirring in the glovebox for 8 h, 1-bromo-4-methylpentane (2.48 g, 2.19 mL, 15.0 mmol, 5.00 equiv.) was added. The vial was subsequently sealed with a cap with septum and placed 4 cm away from two purple LEDs (Kessil PR160-390 nm LEDs, see image below). The reaction was stirred at a speed of 1200 rpm and irradiated for 16 h at 40 °C. After irradiation, the reaction solution was transferred into a 100 mL conical flask. A solution of pinacol (1.06 g, 9.00 mmol, 3.00 equiv.) and Et_3N (8.4 L, 60 mmol, 20 equiv.) in DCM (10 mL) was added and the mixture was stirred for 1 h. The reaction was concentrated *in vacuo* and purified by Biotage Isolera™ flash purification on silica gel (SNAP 25 g silica cartridge), eluting with Et_2O/pentane (0 – 5%, v/v) to give **19** (463 mg, 53%) as a colorless oil. The r.r. (>97:3) was determined by GC-FID analysis of the crude reaction mixture.

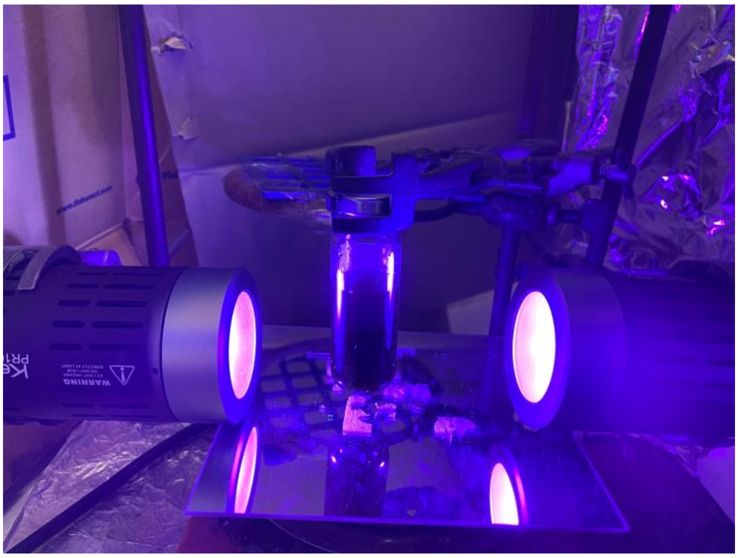

2.7. Alkane Stoichiometry Studies

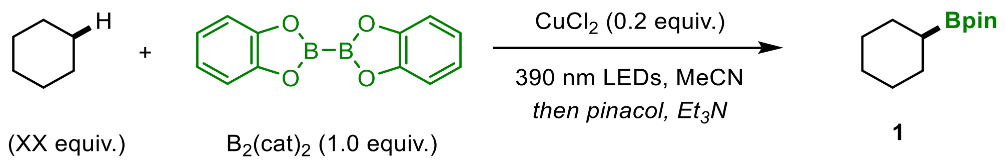

Entry	Alkane (XX equiv.)	GC-FID yield of **2**[a]
1	10	73%
2	5.0	72%
3	3.0	52%
4	1.0	22%
5[b]	1.0	50%
6[c]	1.0	60%

Table S8. Effect of alkane stoichiometry: Boronic ester 1

[a] Yield determined by GC-FID analysis using 1,3,5-trimethoxybenzene as an internal standard. [b] Reaction performed using B_2cat_2 (2.0 equiv.) and $CuCl_2$ (2.0 equiv.). [c] The reaction was irradiated for 24 h before an additional portion of $CuCl_2$ (20 mol%) was added and irradiation continued for a further 24 h.

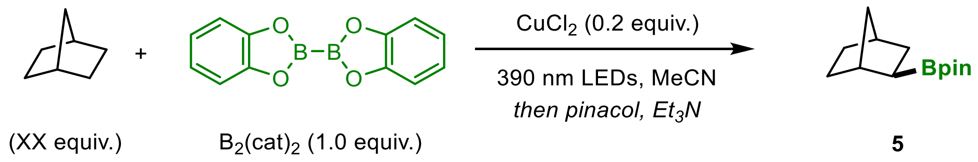

Entry	Alkane (XX equiv.)	Yield of **5**	Yield of recovered alkane
1	5.0	67%	88%
2	3.0	52%	87%
3[a]	1.0	44%	52%

Table S9. Effect of alkane stoichiometry: Boronic ester 5

[a] Reaction performed using B_2cat_2 (2.0 equiv.) and $CuCl_2$ (2.0 equiv.).

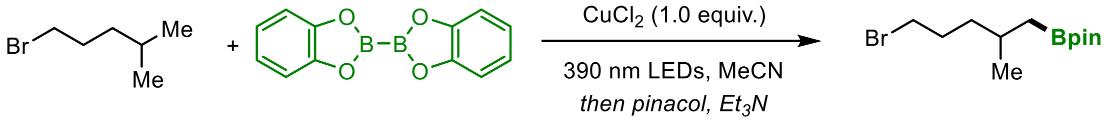

(XX equiv.) $B_2(cat)_2$ (1.0 equiv.) **19**

Entry	Alkane (XX equiv.)	Yield of **19**	Yield of recovered alkane
1	5.0	44%	*not determined*
2	3.0	36%	*not determined*

Table S10. Effect of alkane stoichiometry: Boronic ester 19

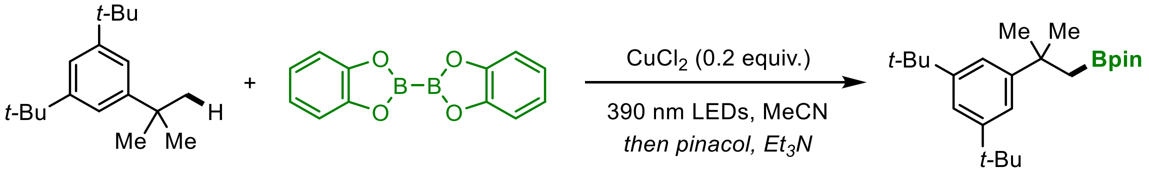

(XX equiv.) $B_2(cat)_2$ (1.0 equiv.) **23**

Entry	Alkane (XX equiv.)	Yield of **23**	Yield of recovered alkane
1	5.0	43%	99%
2	3.0	41%	98%
3[a]	1.0	39%	44%

Table S11. Effect of alkane stoichiometry: Boronic ester 23

[a] Reaction performed using B_2cat_2 (2.0 equiv.) and $CuCl_2$ (2.0 equiv.).

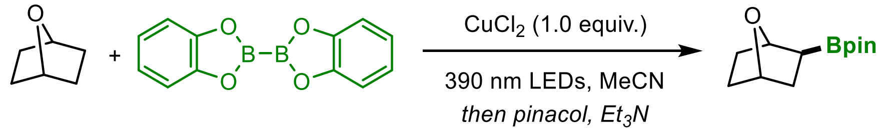

(XX equiv.) $B_2(cat)_2$ (1.0 equiv.) **31**

Entry	Alkane (XX equiv.)	Yield of **31**	Yield of recovered alkane
1	5.0	53%	*not determined*
2	3.0	43%	*not determined*

Table S12. Effect of alkane stoichiometry: Boronic ester 31

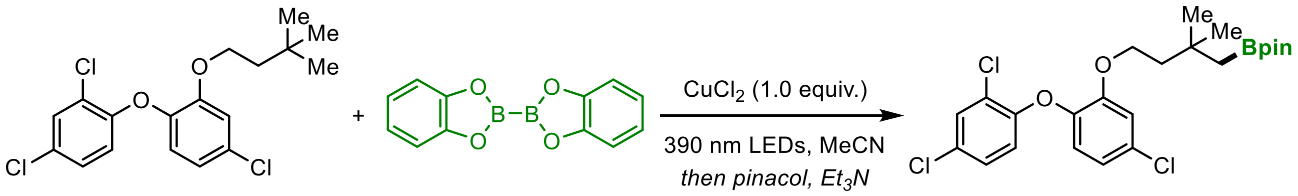

triclosan derivative (XX equiv.) B$_2$(cat)$_2$ (1.0 equiv.) **57**

Entry	Alkane (XX equiv.)	Yield of **57**	Yield of recovered **57-S**
1	5.0	43%	93%
2	3.0	31%	89%
3	1.0	28%	50%
4[a]	1.0	37%	41%

Table S13. Effect of alkane stoichiometry: Boronic ester 57

[a] Reaction performed using B$_2$cat$_2$ (2.0 equiv.) and CuCl$_2$ (2.0 equiv.).

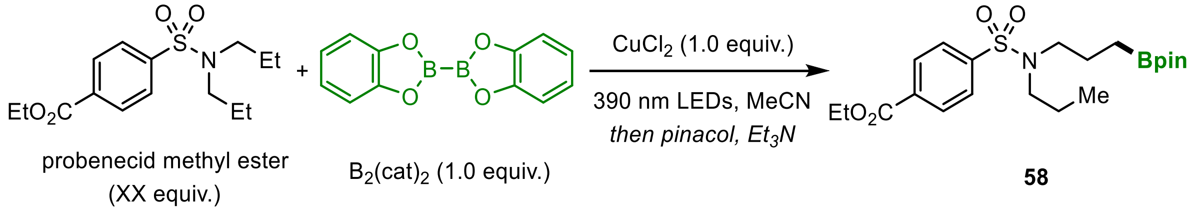

probenecid methyl ester (XX equiv.) B$_2$(cat)$_2$ (1.0 equiv.) **58**

Entry	Alkane (XX equiv.)	Yield of **58**	Yield of recovered **58-S**
1	5.0	35%	88%
2	3.0	31%	90%
3[a]	1.0	27%	43%

Table S14. Effect of alkane stoichiometry: Boronic ester 58

[a] Reaction performed using B$_2$cat$_2$ (2.0 equiv.) and CuCl$_2$ (2.0 equiv.).

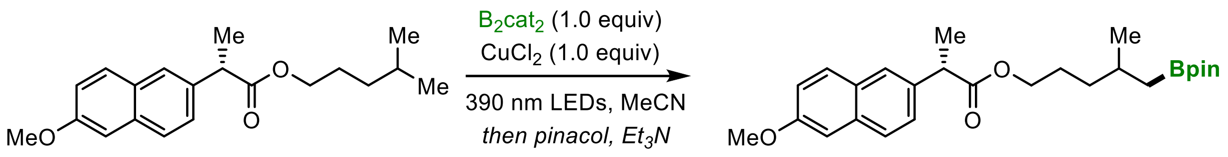

Naproxen derivative
(XX equiv.)

62

Entry	Alkane (x equiv.)	Yield of **62**	Yield of recovered **62-S**
1	5.0	33%	88%
2	3.0	21%	98%
3	1.0	15%	-
3[a]	1.0	23%	81%

Table S15. Effect of alkane stoichiometry: Boronic ester 62

[a] Reaction performed using B_2cat_2 (2 equiv.) and $CuCl_2$ (2 equiv.).

2.8. Selectivity Comparison

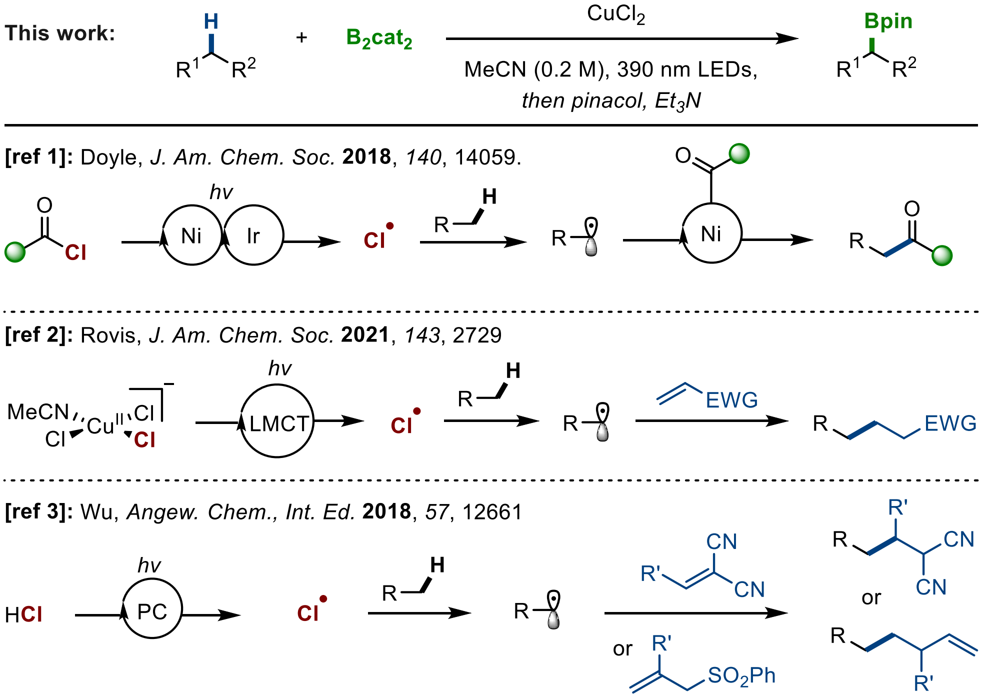

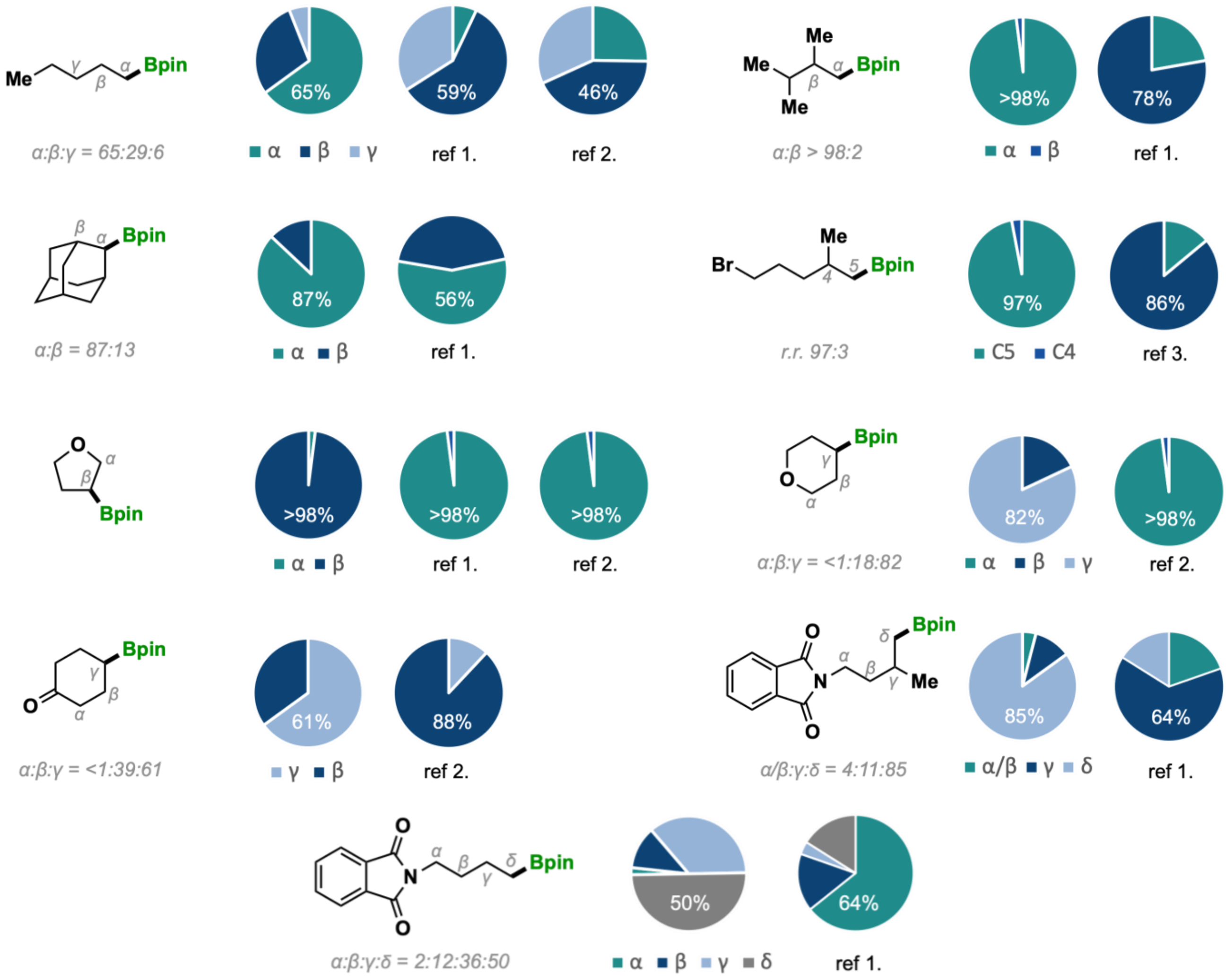

Figure S1. Selectivity comparison

2.9. Comparison with Metal-free Method

Metal-free photoinduced C(sp^3)-H borylation of alkanes: Aggarwal, *Nature*, **2020**, *586*, 714

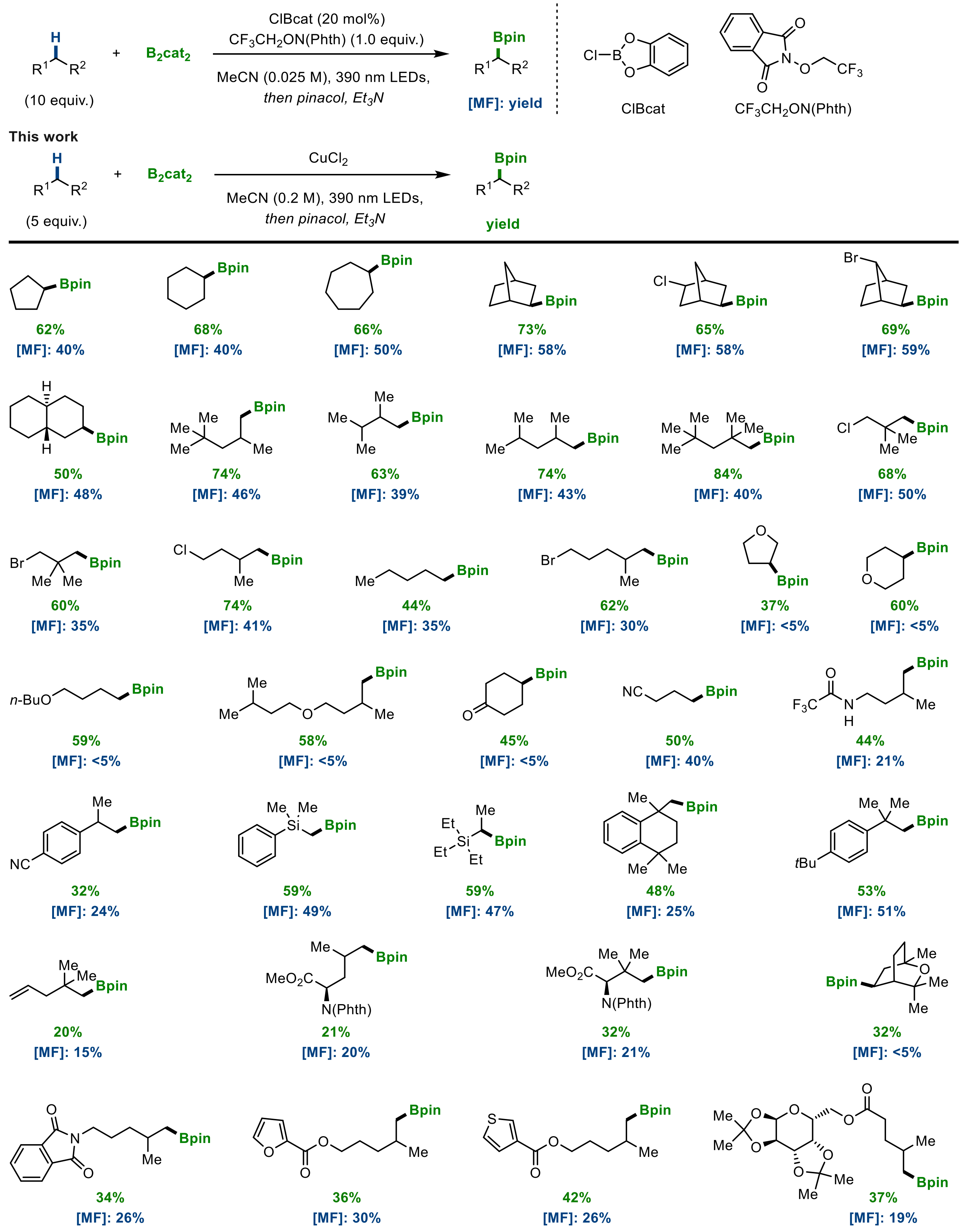

Figure S2. Comparison with metal-free method

2.10. Unsuccessful Substrates

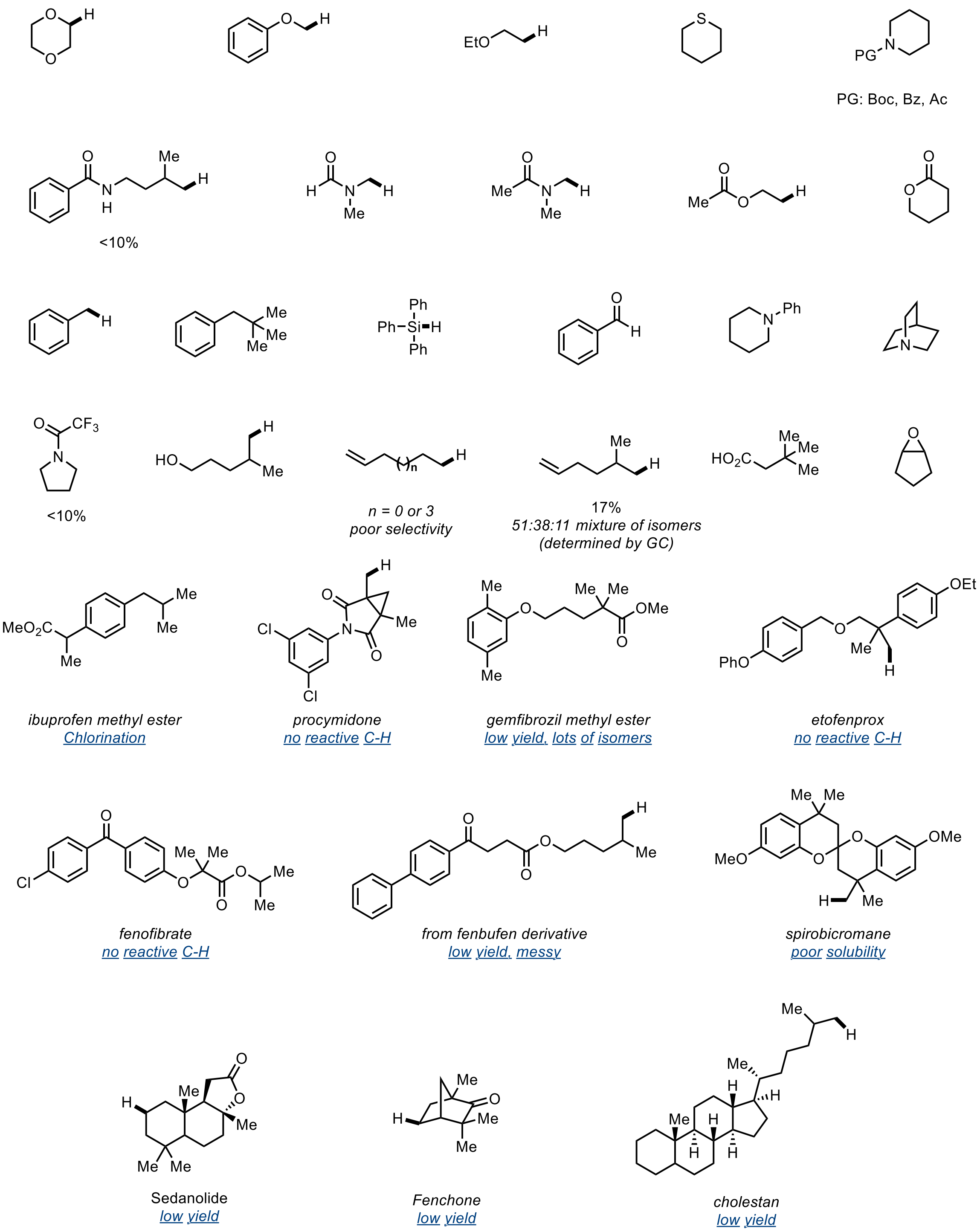

Figure S3. Unsuccessful substrates

2.11. Reaction Cost Comparison

Reagent	Price/mol (£)	Vender	This work	Metal-free[a]
B_2cat_2	862	Apollo	1.0 equiv.	1.0 equiv.
$CuCl_2$	16.7	Sigma-Aldrich	0.2 or 1.0 equiv.	0.2 equiv.
ClBcat	2994	Tokyo Chemical Industry (TCI)		1.0 equiv.
N-(2,2,2-trifluoro-ethoxy)phthalimide	1682	Self-made		1.0 equiv.
N-(2,2,2-trifluoro-ethoxy)phthalimide	77850	AbaChemScene		1.0 equiv.
pinacol	28.3	Fluorochem	3.0 equiv.	3.0 equiv.
cost/mol (£)			950[b]<br>964[c]	3232[d]<br>79400[e]

Table S16. Reaction cost comparison

[a] Reference 3. [b] $CuCl_2$ (0.2 equiv.). [c] $CuCl_2$ (1.0 equiv.). [d] Reaction cost with self-made reagents. [e] Reaction cost with commercially available reagents.

2.12. Comparison between General Procedures B and C for Borylations of Cyclic Ethers

The beneficial effect of pyridine in the borylation reaction of THF was not found to be general for cyclic ether substrates (Figure S4). As a result, we do not have an explanation for the role of pyridine in the reaction with THF.

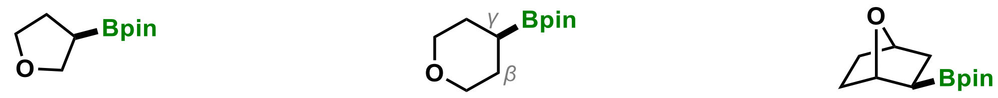

28 37% (with pyridine)[a] <5% (without pyridine)[b]

29 32% (with pyridine)[a] 60% (without pyridine)[b]

31 57% (with pyridine)[a] 53% (without pyridine)[b]

Figure S4. Comparison between General Produce B and C Using Cyclic Ether

[a] Prepared following **General Procedure C**; [b] Prepared following **General Procedure B**.

3. MECHANISTIC STUDIES

3.1. Studies into the $CuCl_2$ Loading

3.1.1. Effect of pre-stirring with different $CuCl_2$ loadings

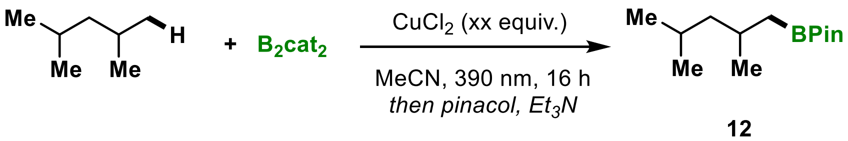

12
r>97:3 r.r.

Prepared following **General Procedure** and **General Procedure-prestirring**, using 2,4-dimethylpentane (150 mg, 215 µL, 1.50 mmol, 5.00 equiv.), $CuCl_2$ (0.060–3.0 mmol, 0.20–10 equiv.), and B_2cat_2 (71 mg, 0.30 mmol, 1.0 equiv.) in MeCN (1.5 mL, *c* = 0.20 M). Diethyl phthalate as an internal standard was subsequently added. After vigorously shaking for 1 min, 0.20 mL of the crude reaction mixture was filtered through a short plug of silica and the yields of **12** were determined by GC-FID analysis.

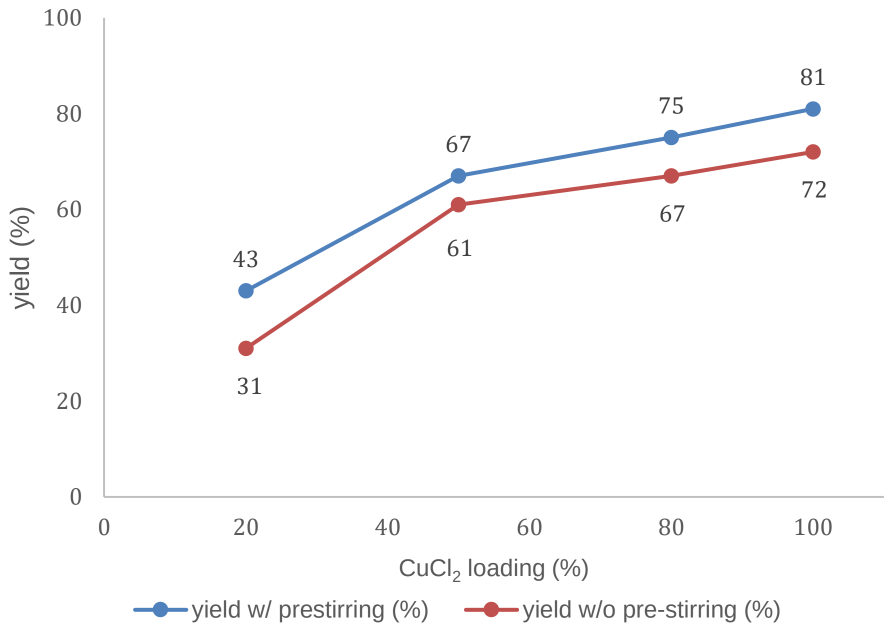

Figure S5. Effects of pre-stirring using 0.2–1 equiv. $CuCl_2$

3.1.2. Effect of $CuCl_2$ loading on regioselectivity of adamantane

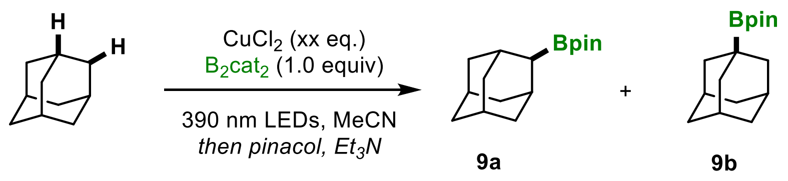

Prepared following **General Procedure** and **General Procedure-prestirring**, using adamantane (204 mg, 1.50 mmol, 5.00 equiv.), $CuCl_2$ (0.060–3.0 mmol, 0.20–10 equiv.), and B_2cat_2 (71 mg, 0.30 mmol, 1.0 equiv.) in MeCN (1.5 mL, *c* = 0.20 M). Diethyl phthalate as an internal standard was subsequently added. After vigorously shaking for 1 min, 0.20 mL of the crude reaction mixture was filtered through a short plug of silica and the yields of **9a** and **9b** were determined by GC-FID analysis.

Entry	$CuCl_2$ (xx equiv.)	2°/3° selectivity (**9a**:**9b**)	GC-FID yield	**9b** selectivity
1	0.2	87:13	60%	13%
2	0.5	77:23	55%	23%
3	1.0	47:53	58%	53%
4	2.0	23:77	51%	77%
5	3.0	11:89	34%	89%
6	5.0	<2:98	19%	>98%
7	10.0	chlorinated product	<5%	not determined

Table S17. Effect of $CuCl_2$ on regioselectivity in the borylation of adamantane

3.1.3. Effect of $CuCl_2$ loading on regioselectivity of 2,5-dimethylhexane

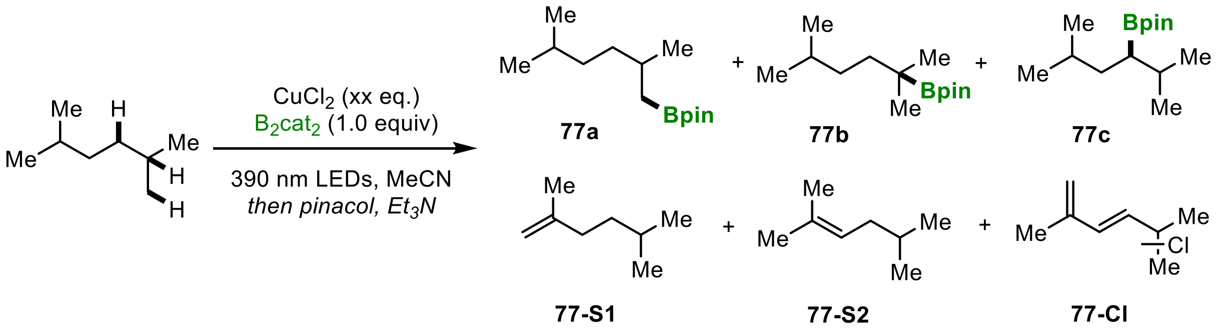

Prepared following **General Procedure** and **General Procedure-prestirring**, using 2,5-dimethylhexane (117 mg, 1.50 mmol, 5.00 equiv.), $CuCl_2$ (0.060–3.0 mmol, 0.20–10 equiv.), and B_2cat_2 (71 mg, 0.30 mmol, 1.0 equiv.) in MeCN (1.5 mL, c = 0.20 M). Diethyl phthalate as an internal standard was subsequently added. After vigorously shaking for 1 min, 0.20 mL of the crude reaction mixture was filtered through a short plug of silica and the yields of **77** and **side products 77-S1**, **77-S2**, **77-Cl** were determined by GC-FID analysis.

Entry	$CuCl_2$ (xx equiv.)	GC-FID yield	Regioselectivity (**77a**: **77b**: **77c**)	Side-product yields (**77-S1** / **77-S2** / **77-Cl**)[a]
1	0.2	20%	98:<1:2	3% / 4% / 4%
2	1.0	51%	96:1:3	8% / 4% / 16%
3	2.0	21%	93:4:3	8% / 4% / 22%
4	3.0	3%	77:15:8	14% / 9% / 31%

Table S18. Effect of $CuCl_2$ on regioselectivity in the borylation of 2,5-dimethylhexane

[a] The yields of the alkene and chlorinated side-products are uncalibrated, but are included to show the increase in side-product formation with increasing $CuCl_2$ loading.

3.1.4. Effect of $CuCl_2$ loading on regioselectivity of pentane

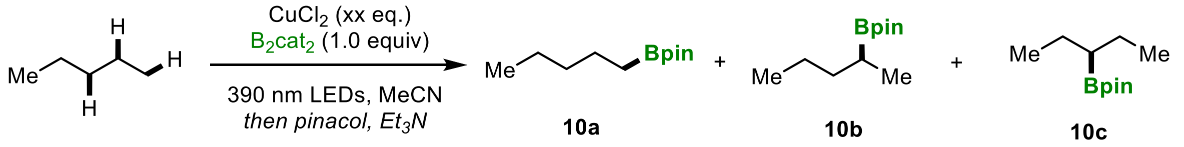

Prepared following **General Procedure** and **General Procedure-prestirring**, using pentane (108 mg, 172 µL, 1.50 mmol, 5.00 equiv.), $CuCl_2$ (0.060–3.0 mmol, 0.20–10 equiv.), and B_2cat_2 (71 mg, 0.30 mmol, 1.0 equiv.) in MeCN (1.5 mL, *c* = 0.20 M). Diethyl phthalate as an internal standard was subsequently added. After vigorously shaking for 1 min, 0.20 mL of the crude reaction mixture was filtered through a short plug of silica and the yields of **10a-c** were determined by GC-FID analysis.

Entry	$CuCl_2$ (xx equiv.)	GC-FID yield	regioselectivity (**10a**:**10b**:**10c**)	regioselectivity (1°:2°)
1	0.2	16%	57:32:11	57:43
2	1.0	47%	62:30:8	62:38
3	2.0	42%	43:48:9	43:57
4	3.0	13%	21:70:9	21:79

Table S19. Effect of $CuCl_2$ on regioselectivity in the borylation of pentane

3.2. Investigations into the Reaction of $CuCl_2$ with B_2cat_2

3.2.1. NMR studies of $CuCl_2$ and B_2cat_2 in CD_3CN

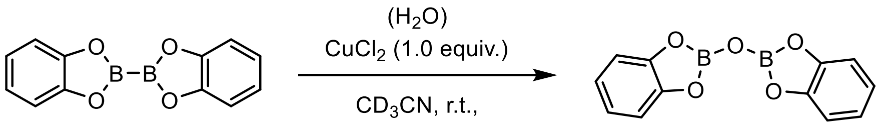

Under an ambient atmosphere, a J. Young NMR tube was charged with $CuCl_2$ (40 mg, 0.30 mmol, 1.0 equiv.), and B_2cat_2 (71 mg, 0.30 mmol, 1.0 equiv.). The NMR tube was transferred into an anhydrous, argon-filled glovebox where CD_3CN (1.5 mL, c = 0.20 M) was added. The NMR tube was capped before vigorously shaking for 1 min. The sample was removed from the glovebox and analysed by ^{1}H, ^{11}B and ^{13}C NMR.

Under an ambient atmosphere, a flame dried 8 mL Biotage® microwave reaction vial equipped with a magnetic stir bar was charged with $CuCl_2$ (40 mg, 0.30 mmol, 1.0 equiv.), and B_2cat_2 (71 mg, 0.30 mmol, 1.0 equiv.). The vial was transferred into an anhydrous, argon-filled glovebox where CD_3CN (1.5 mL, c = 0.20 M) was added. The reaction was stirred at a speed of 1200 rpm for 16 h before transferring into an NMR, removing from the glovebox, and analysing by ^{1}H, ^{11}B and ^{13}C NMR.

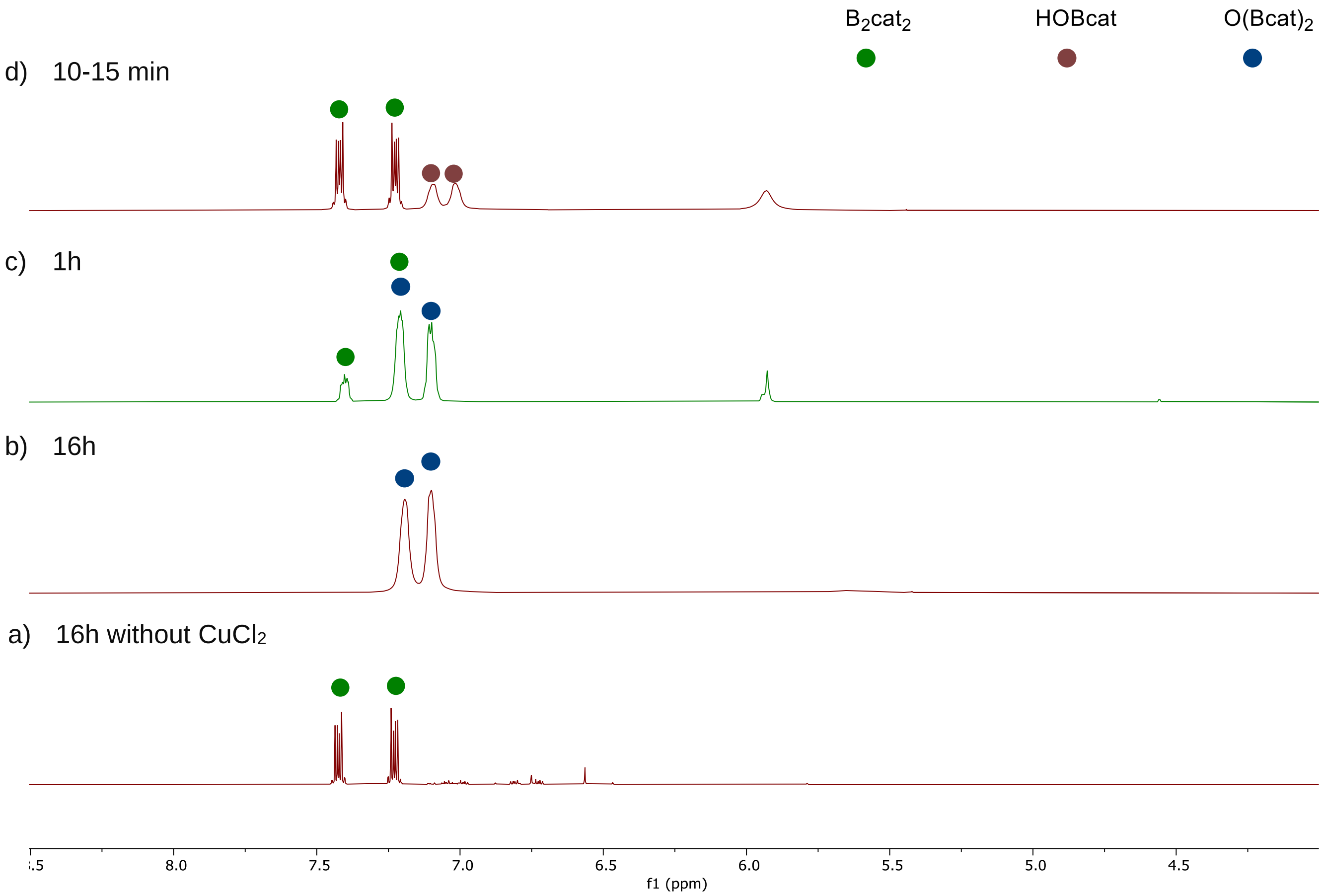

Figure S6. ^{1}H NMR spectra of B_2cat_2 and $CuCl_2$ in CD_3CN

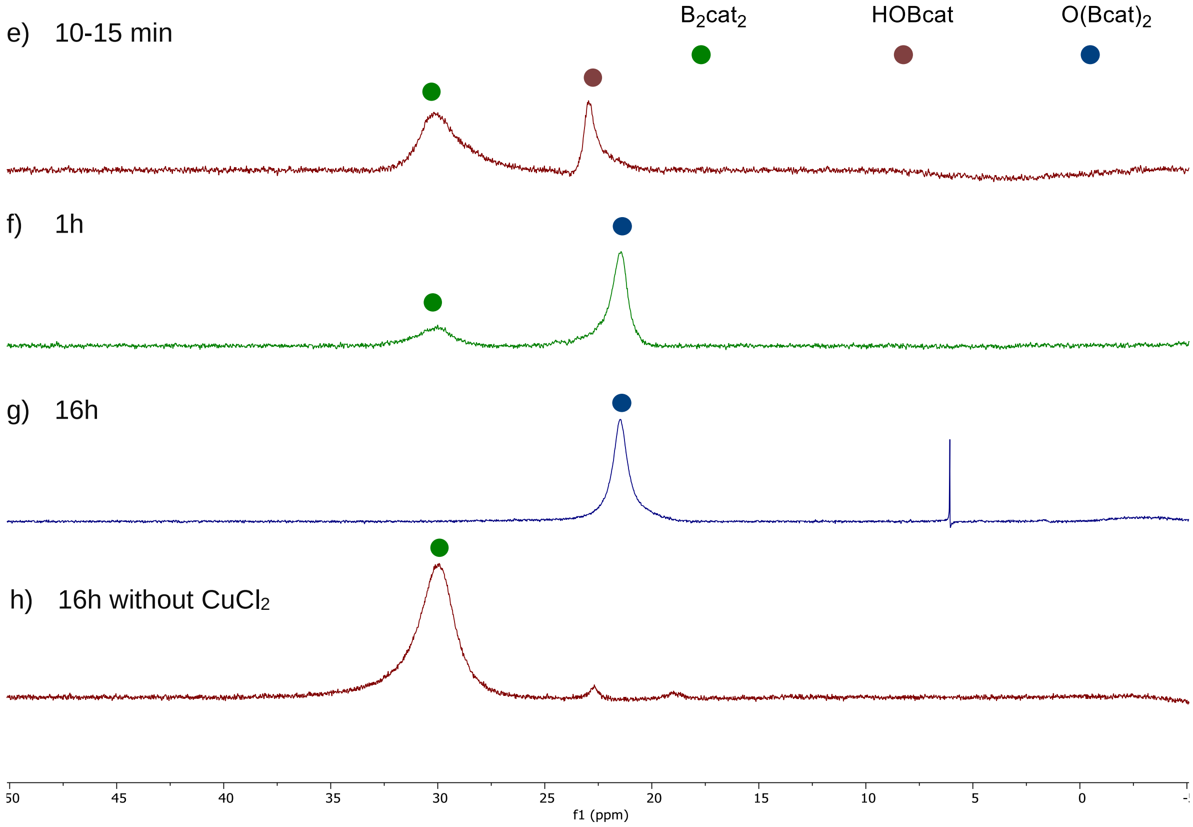

Figure S7. ^{11}**B NMR spectra of** B_2cat_2 **and** $CuCl_2$ **in** CD_3CN

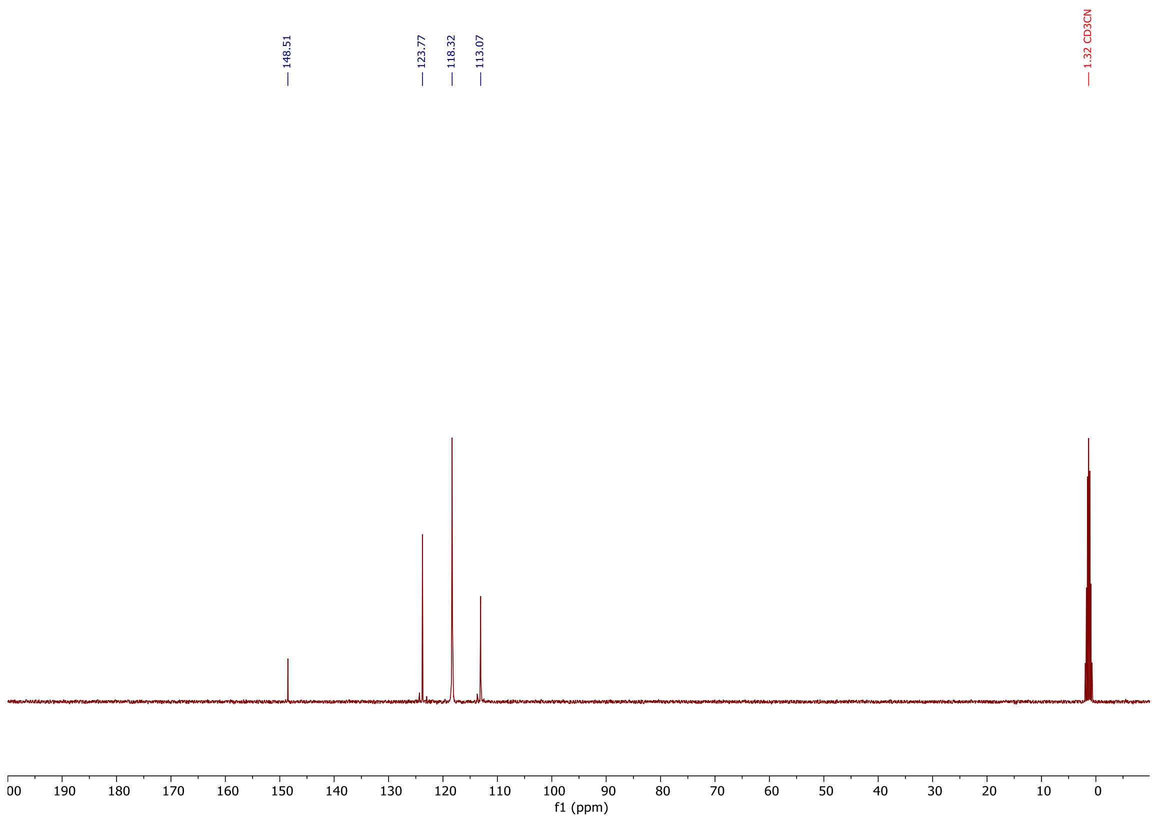

Figure S8. ^{13}**C NMR spectra of** B_2cat_2 **and** $CuCl_2$ **in** CD_3CN **after 16 h**

Figure S6 shows the ^{1}H NMR spectra of the reaction solution of $CuCl_2$ and B_2cat_2 in CD_3CN at the time points of 10-15 minutes, 1 h, and 16 h. After 10-15 minutes, B_2cat_2 was observed to be partially converted to HOBcat. After 1 h, B_2cat_2 was observed to be partially converted to $O(Bcat)_2$. After 16 h, B_2cat_2 was observed to be fully converted to $O(Bcat)_2$, whereas no B–B cleavage occurred in the absence of $CuCl_2$. This is also seen in the ^{11}B NMR spectra (Figure S7). A ^{13}C NMR spectrum was also measured to confirm the product ($O(Bcat)_2$) at the time point of 16 h (Figure S8).

3.2.2. H_2 gas detection

Under an ambient atmosphere, a J. Young NMR tube was charged with $CuCl_2$ (40 mg, 0.30 mmol, 1.0 equiv.), and B_2cat_2 (71 mg, 0.30 mmol, 1.0 equiv.). The NMR tube was transferred into an anhydrous, argon-filled glovebox where CD_3CN (1.5 mL, c = 0.20 M) was added. The NMR tube was capped before vigorously shaking for 1 min. The sample was removed from the glovebox and analysed by ^{1}H NMR (Figure S9a and S9b). After that, HBcat (60 mg, 0.5 mmol, 2.0 equiv.) and H_2O (18 mg, 18 µl, 1.0 mmol, 4.0 equiv.) were added to the NMR tube. The tube was vigorously shaken for 1 min before further ^{1}H NMR analysis (Figure S9c).

As shown below (Figure S9b), a new signal at 4.56 ppm was observed by ^{1}H NMR after 1 h. This peak corresponds to H_2 gas according to the reported chemical shift of H_2 gas in CD_3CN.[24] An identical peak (δ_H = 4.56 ppm) was observed upon addition of HBcat and H_2O, which are known to react to generate H_2 gas.

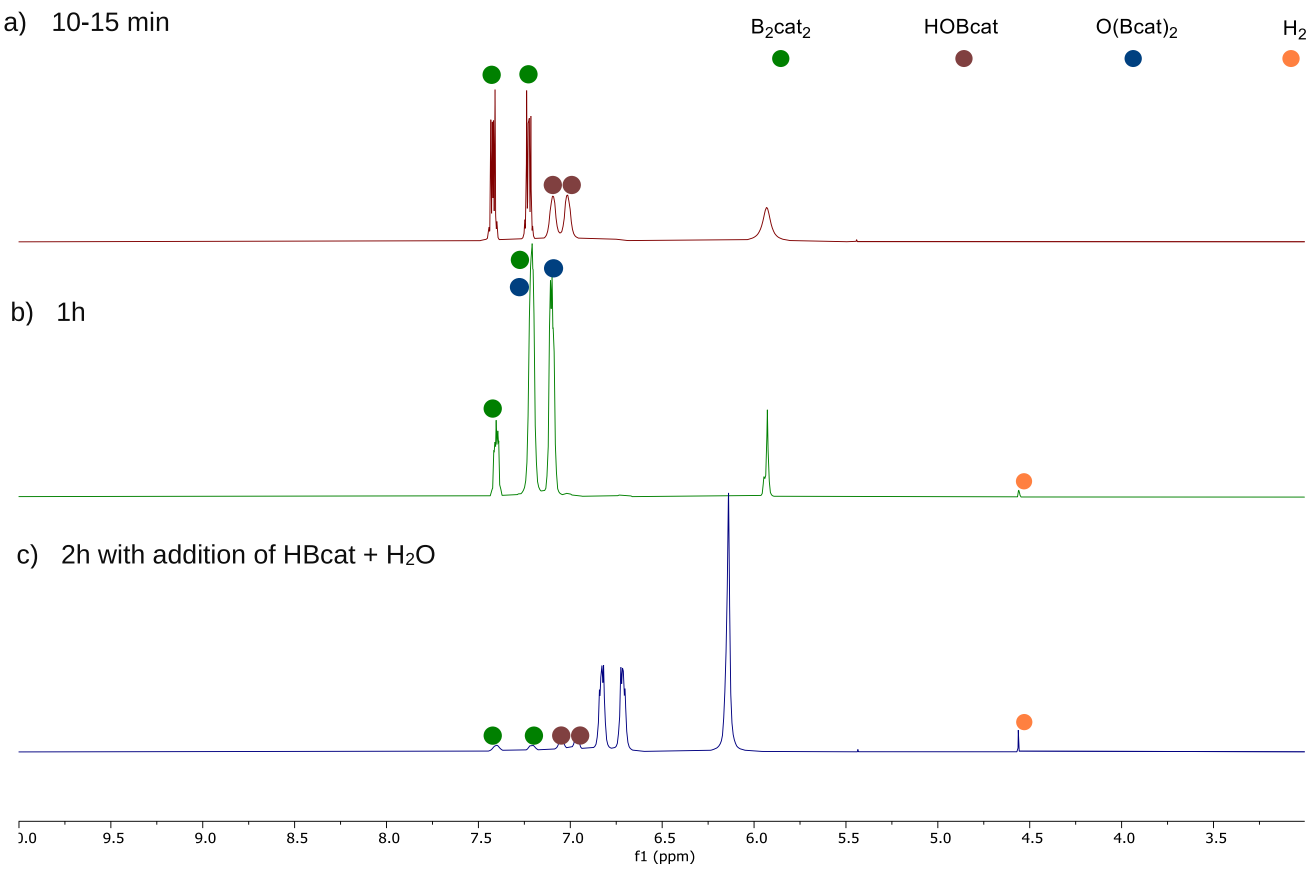

Figure S9. Detection of H_2 gas by ^{1}H NMR

3.3. Investigations into the Use of ClBcat as the Borylating Agent

3.3.1. NMR spectra of ClBcat in CD_3CN

Figure S10 shows the ^{1}H NMR spectra of ClBcat in CD_3CN at 150 and 40 mM (reaction concentration). At 150 mM, ClBcat can be observed, but trace water in the solvent has resulted in significant hydrolysis to $O(Bcat)_2$ and two other unidentified species. At 40 mM, complete hydrolysis of ClBcat occurs to form $O(Bcat)_2$. The ^{1}H and ^{11}B NMR spectra (Figure S11) of $O(Bcat)_2$ formed form ClBcat were compared to those of $O(Bcat)_2$ generated by $CuCl_2$-catalysed oxidation of B_2cat_2. All spectroscopic data matched those of the independently synthesised $O(Bcat)_2$.

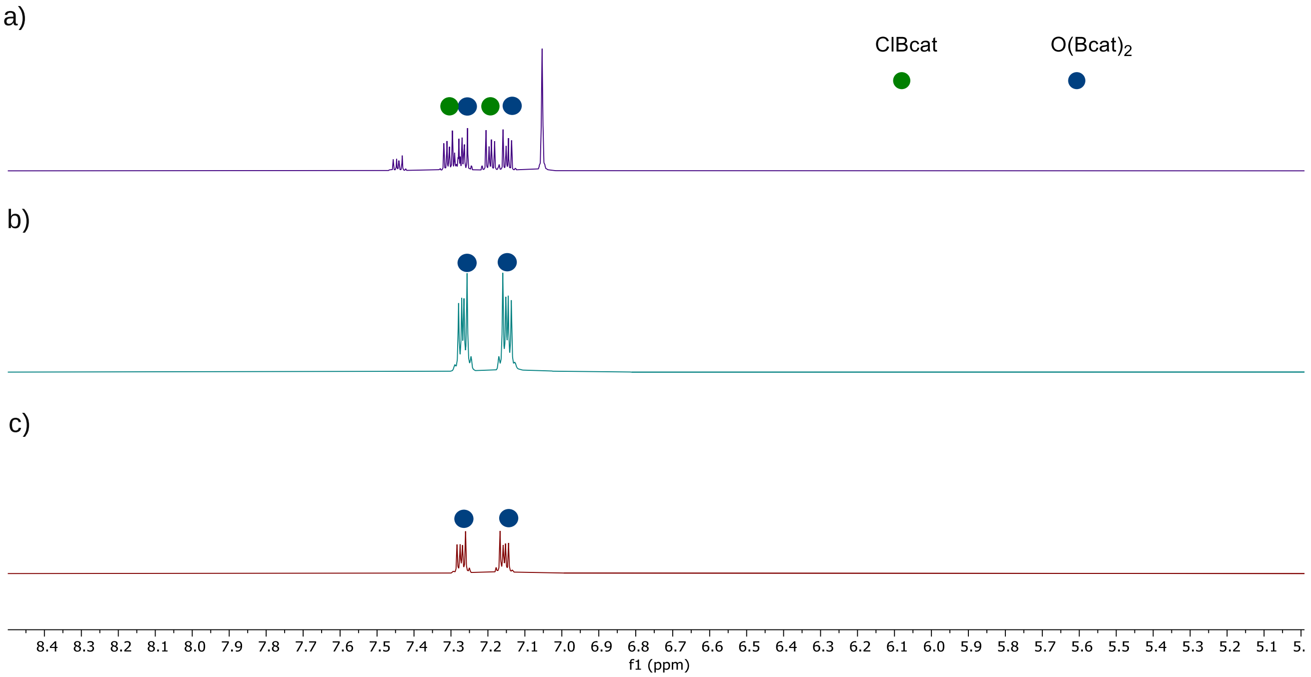

Figure S10. ^{1}H NMR spectra of ClBcat in CD_3CN

a) ClBcat in CD_3CN (0.15 M); b) ClBcat in CD_3CN (0.04 M); c) mixture of B_2cat_2 and $CuCl_2$ in CD_3CN (0.2 M) after 16 h.

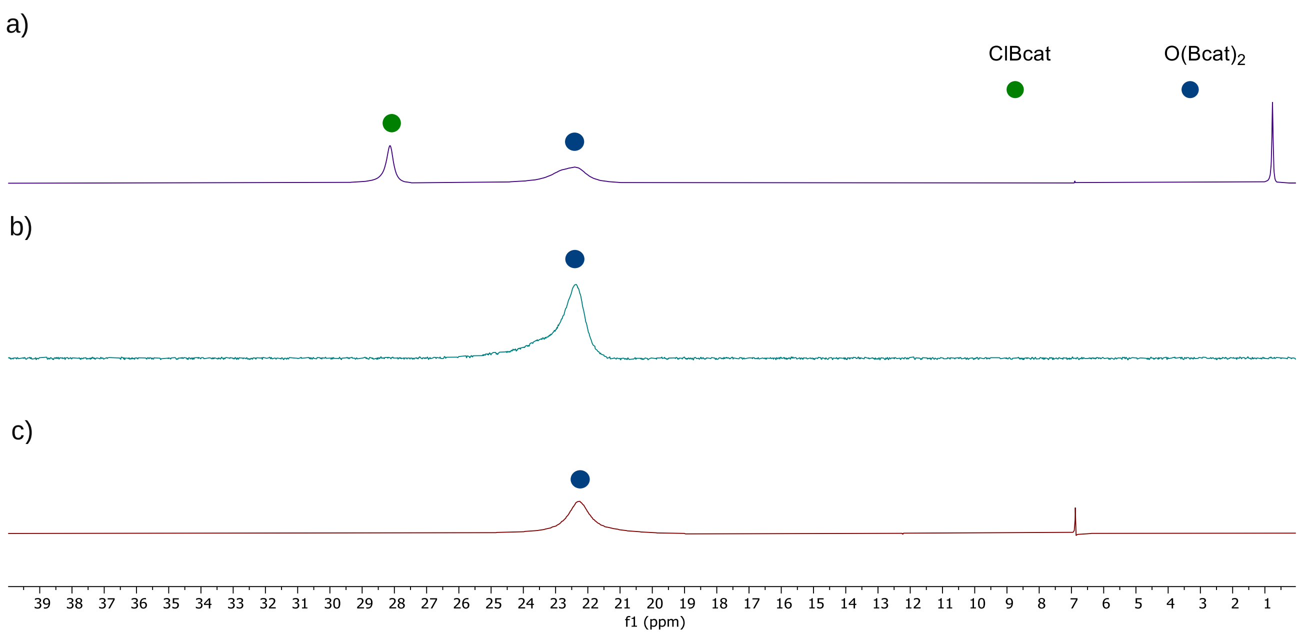

Figure S11. ^{11}B NMR spectra of ClBcat in CD_3CN

a) ClBcat in CD_3CN (0.15 M); b) ClBcat in CD_3CN (0.04 M); c) mixture of B_2cat_2 and $CuCl_2$ in CD_3CN (0.2 M) after 16 h.

3.3.2. Investigation into the reactivity of ClBcat in the borylation of cyclohexane

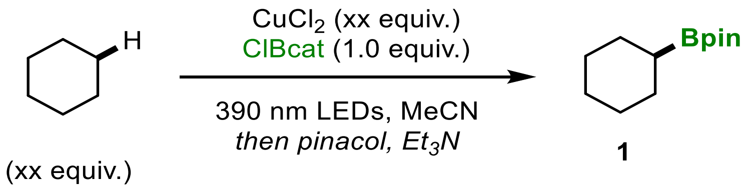

Under an ambient atmosphere, a flame dried 8 mL Biotage® microwave reaction vial equipped with a magnetic stir bar was charged with $CuCl_2$, and ClBcat (93 mg, 0.60 mmol, 1.0 equiv.). The vial was transferred into an anhydrous, argon-filled glovebox where anhydrous solvent was added followed by cyclohexane The vial was sealed with a cap with septum and placed 4 cm away from two purple LEDs (Kessil PR160-390 nm LEDs). The reaction was stirred at a speed of 1200 rpm and irradiated for 16 h at 40 °C. After irradiation, a solution of pinacol (106 mg, 0.900 mmol, 1.50 equiv.) and Et_3N (0.84 mL, 6.0 mmol, 10 equiv.) in DCM (1 mL) was added and stirring was continued for 1 h. 1,3,5-trimethoxybenzene (100 mg, 0.600 mmol, 1.00 equiv.) as the internal standard was added. After vigorously shaking for 3 min, the crude reaction mixture was filtered through a short plug of silica and the yield was determined by GC-FID analysis.

Entry	$CuCl_2$ (xx equiv.)	cyclohexane (xx equiv.)	GC-FID yield
1	0.2	5.0	45%
2[a]	0.2	5.0	36%
3	0.5	2.5	48%
4	0.5	5.0	50%
5	1.0	5.0	48%
6[a]	1.0	5.0	<5%

Table S20. Reaction optimization using ClBcat as borylating reagent

3.3.3. NMR studies into the borylation reaction with ClBcat

Figure S12 shows the ^{11}B NMR spectra of the reaction solution using ClBcat under the procedure above in CD_3CN. After 16 h irradiation, it is observed that B_2cat_2 is fully converted to the alkyl boronic ester product and $O(Bcat)_2$. The unconverted $O(Bcat)_2$ was determined and confirmed by ^1H and ^{13}C NMR (Figures S12 and S13). To investigate the regeneration of $O(Bcat)_2$, extra HOBcat was added into the above reaction solution. HOBcat was found to be rapidly converted to $O(Bcat)_2$ and no HOBcat was observed.

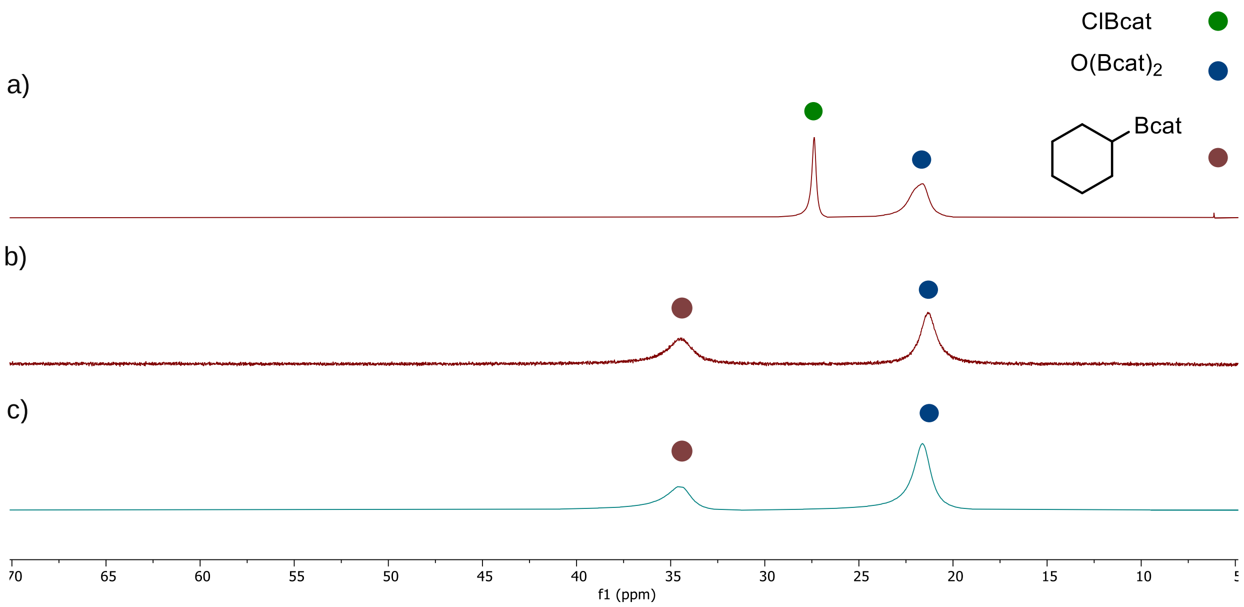

Figure S12. ^{11}B NMR spectra of the reaction with ClBcat

a) ^{11}B NMR spectra of the reaction with ClBcat without irradiation; b) ^{11}B NMR spectra of the reaction with ClBcat after 16 h irradiation c) ^{11}B NMR spectra of the reaction with ClBcat after 16 h irradiation followed by addition of HOBcat (0.1 mmol)

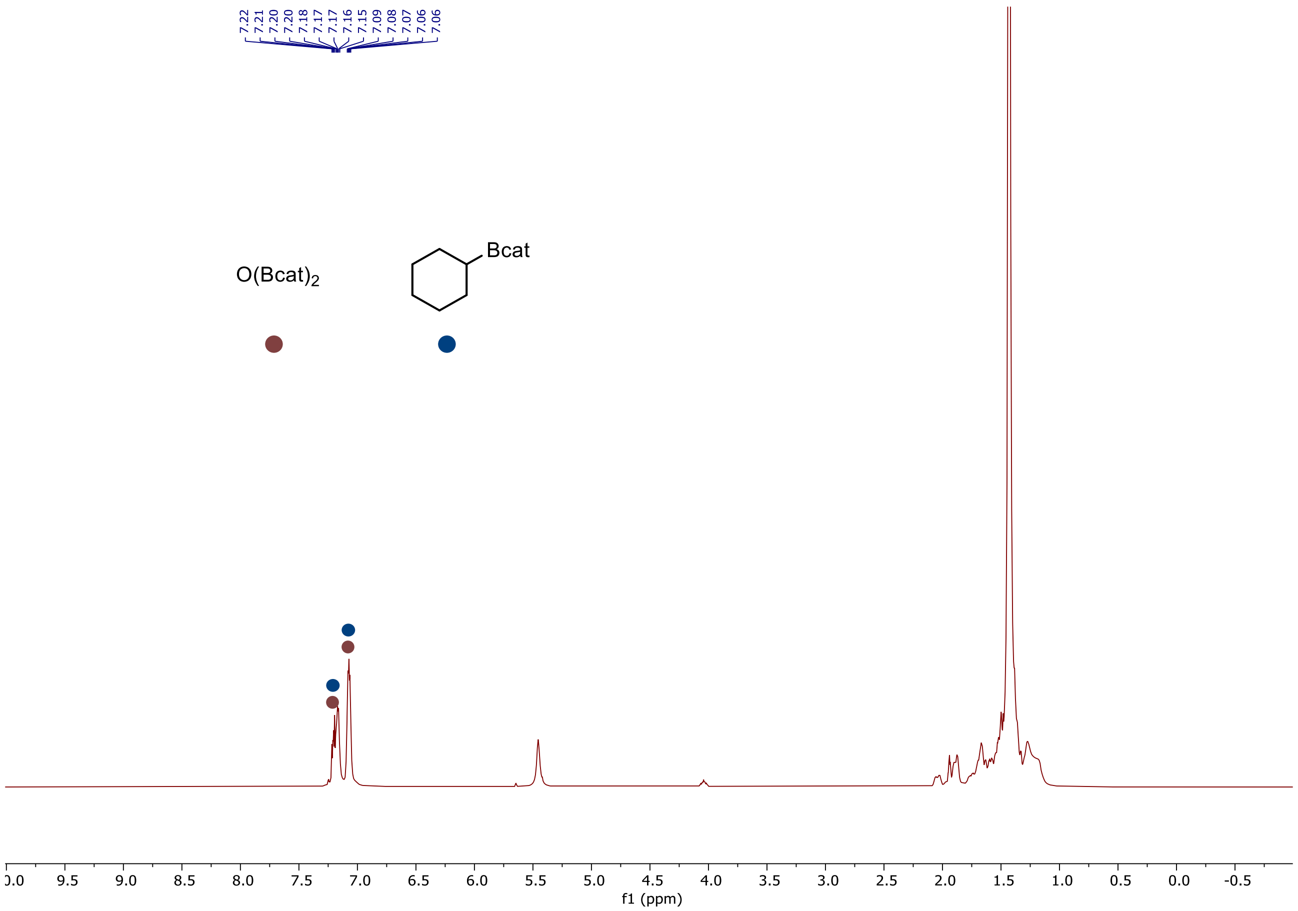

Figure S13. ^{1}H NMR spectra of the reaction with ClBcat after 16 h irradiation

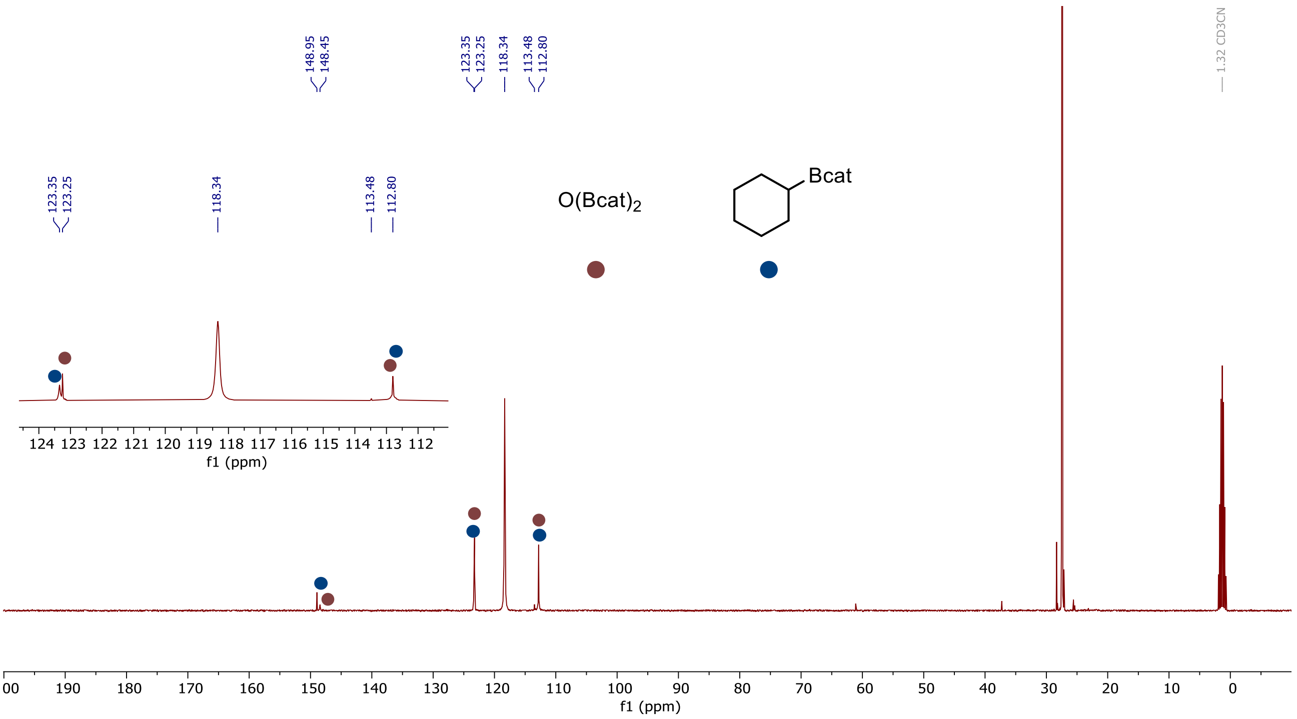

Figure S14. ^{13}C NMR spectra of the reaction with ClBcat after 16 h irradiation

3.4. Investigations into the Formation and Reactivity of $O(Bcat)_2$

3.4.1. Independent synthesis of HOBcat

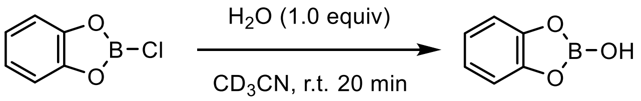

(1.0 equiv.) **HOBcat**

A 5.0 mL vial equipped with a stirrer bar was charged with 2-chloro-1,3,2-benzodioxaborole (150 mg, 1.00 mmol, 1.00 equiv.), H_2O (18 mg, 1.0 mmol, 1.0 equiv.) and CD_3CN (1.5 mL, c = 0.67 M) under an argon atmosphere. The mixture was stirred for 20 min and then was analysed by NMR spectroscopy without further purification.

NMR Spectroscopy (*see spectra*):

^{1}H NMR (400 MHz, CD_3CN) δ_H 7.13 – 7.07 (m, 2H), 7.05 – 6.98 (m, 2H) ppm;

^{13}C NMR (101 MHz, CD_3CN) δ_C 148.9, 123.2, 112.9 ppm;

^{11}B NMR (128 MHz, CD_3CN) δ_B 23.48 ppm.

3.4.2. Independent synthesis of $O(Bcat)_2$

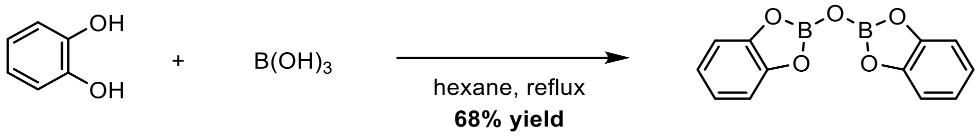

(1.0 equiv.) (1.0 equiv.) **$O(Bcat)_2$**

A round-bottomed flask equipped with a stirrer bar, Dean-Stark trap and reflux condenser capping with a drying tube was charged with boric acid (3.1 g, 50 mmol, 1.0 equiv.), catechol (5.5 g, 50 mmol, 1.0 equiv.) and hexane (30 mL, *c* = 1.7 M). The mixture was refluxed for 6 h until the water was separated. The solvent was removed and bis(catecholboryl) oxide $O(Bcat)_2$ (8.6 g, 68%) was obtained as a white solid with HOBcat impurity, which was kept under inert conditions and used without further purification.

NMR Spectroscopy (*see spectra*):

^{1}H NMR (400 MHz, CD_3CN) δ_H 7.24 (dt, J = 8.0, 4.0 Hz, 4H), 7.12 (dt, J = 8.0, 4.0 Hz, 4H) ppm;

^{13}C NMR (101 MHz, CD_3CN) δ_C 148.6, 123.8, 113.2 ppm;

^{11}B NMR (128 MHz, CD_3CN) δ_B 22.3 ppm.

3.4.3. NMR comparison of $O(Bcat)_2$

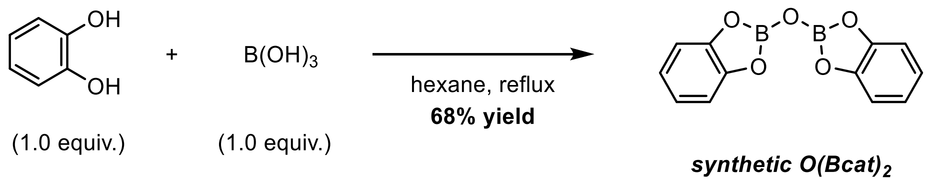

synthetic O(Bcat)$_2$

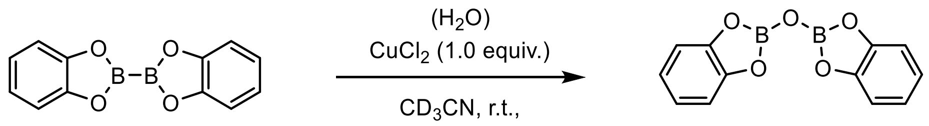

O(Bcat)$_2$ formed *in situ*

δH synthetic $O(Bcat)_2$ (400 MHz, CD_3CN)	δH $O(Bcat)_2$ formed *in situ* (400 MHz, CD_3CN)	Δ (ppm)
7.12	7.11	0.01
7.24	7.23	0.01

Table S21. ^{1}H NMR comparison for synthetic and in situ formed $O(Bcat)_2$

δC synthetic $O(Bcat)_2$ (101 MHz, CD_3CN)	δC $O(Bcat)_2$ formed *in situ* (101 MHz, CD_3CN)	Δ (ppm)
148.6	148.5	0.1
132.8	132.7	0.1
113.1	113.0	0.1

Table S22. ^{13}C NMR comparison synthetic and in situ formed $O(Bcat)_2$

The ^{1}H and ^{13}C NMR spectra of $O(Bcat)_2$ formed by the $CuCl_2$-catalysed method were compared with those of $O(Bcat)_2$ synthesised independently. All spectroscopic data matched.

3.4.4. Investigation into the reactivity of independently synthesised $O(Bcat)_2$

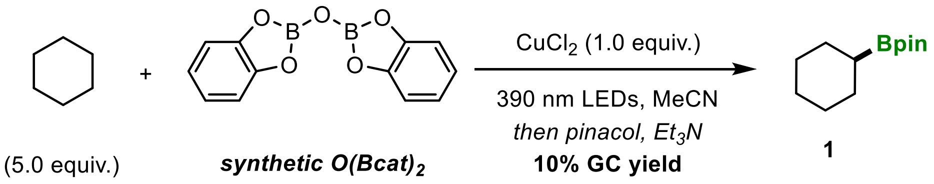

Under an ambient atmosphere, a flame dried 8 mL Biotage® microwave reaction vial equipped with a magnetic stir bar was charged with $CuCl_2$ (40 mg, 0.30 mmol, 1.0 equiv.) and $O(Bcat)_2$ (76 mg, 0.30 mmol, 1.0 equiv.). The vial was transferred to an anhydrous, argon-filled glovebox where anhydrous MeCN (1.5 mL, *c* = 0.2 M) was added followed by cyclohexane (126 mg, 162 μL, 1.50 mmol, 5.00 equiv.). The vial was sealed with a cap with septum and placed 4 cm away from two purple LEDs (Kessil PR160-390 nm LEDs). The reaction was stirred at a speed of 1200 rpm and irradiated for 16 h at 40 °C. After irradiation, a solution of pinacol (106 mg, 0.900 mmol, 3.00 equiv.) and Et_3N (0.84 mL, 6.0 mmol, 20 equiv.) in DCM (1 mL) was added and stirring was continued for 1 h. 1,3,5-Trimethoxybenzene (50 mg, 0.30 mmol, 1.0 equiv.) as the internal standard was added. After vigorously shaking for 3 min, the crude reaction mixture was filtered through a short plug of silica and the yield (10%) was determined by GC-FID analysis.

Note: The isolated $O(Bcat)_2$ was found to be extremely sensitive to the air and moisture, which leads to the formation of HOBcat, $B(OH)_3$ and other side products. As a result, a pure sample of $O(Bcat)_2$ could not be isolated. The use of impure $O(Bcat)_2$ in the borylation of cyclohexane is likely responsible for the low yield of borylated product (10%).

3.4.5. Reaction monitoring of the $O(Bcat)_2$ formation

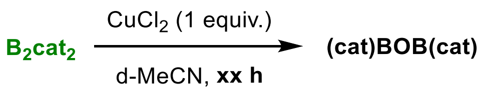

Under an ambient atmosphere, a flame dried 8 mL Biotage® microwave reaction vial equipped with a magnetic stir bar was charged with $CuCl_2$ (20 mg, 0.15 mmol, 1.0 equiv.), and B_2cat_2 (36 mg, 0.15 mmol, 1.0 equiv.). The vial was transferred into an anhydrous, argon-filled glovebox. Under argon atmosphere, anhydrous CD_3CN (0.75 mL, c = 0.20 M) was added. The reaction mixture was stirred for a specific amount of time, after which dibromomethane (42 μL, 0.6 mmol) was added as the internal standard. The reaction solution was then transferred to NMR tubes for ^1H NMR measurement to determine the yield.

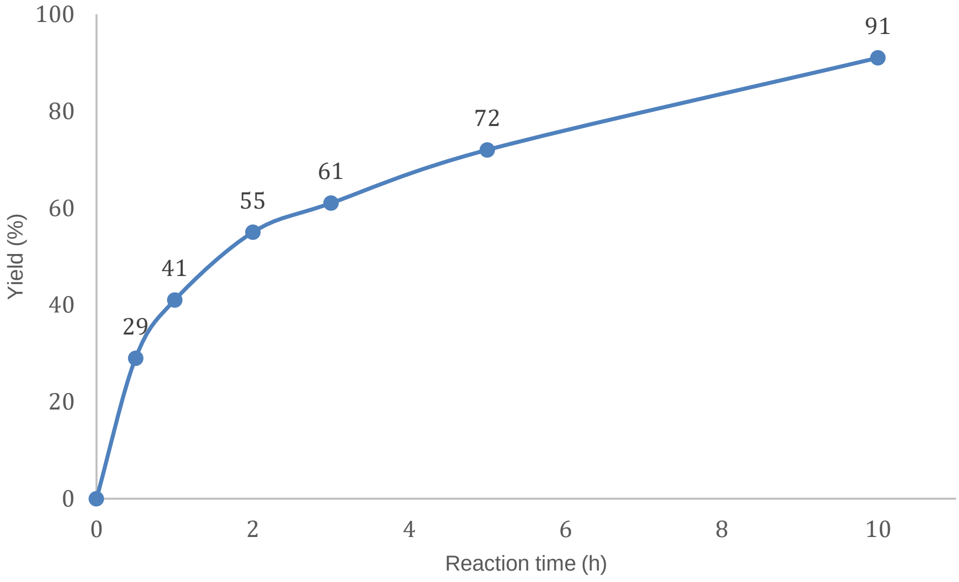

Figure S15. Reaction monitoring of the $O(Bcat)_2$ formation

3.4.6. Investigation into the effects of water concentration on $O(Bcat)_2$ formation

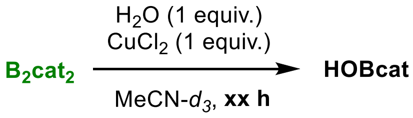

Time (h)	^{1}H NMR yields (%)[a]			^{11}B NMR ratio[b]	
	$O(Bcat)_2$	B_2cat_2	HOBcat	$B_2(OR)_4$	$B(OR)_3$
0.5	0	28	120	33	67
1.0	0	30	120	-	-
2.0	0	29	120	-	-
3.0	0	30	128	-	-
5.0	0	30	136	34	66
10.0	0	30	136	34	66

Table S23. Effects of water (1 equiv.) on $O(Bcat)_2$ formation

[a] Yields were determined by ^{1}H NMR using an internal standard. The yield of HOBcat is based on the molar equivalents of B_2cat_2, with a maximum theoretical yield of 200% because two equivalents of HOBcat can be generated from 1 equivalent of B_2cat_2. [b] The ^{11}B NMR ratio shows the conversion of diboron species [$B_2(OR)_4$] to borate species [$B(OR)_3$] and was determined by integrating the signals at 26-36 ppm (diboron) and 18.5-24.5 ppm (borate). These ratios were only measured at 0.5, 5 and 10 h.

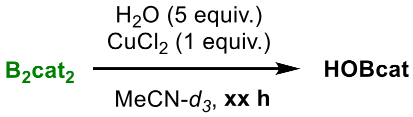

Time (h)	^{1}H NMR yields (%)[a]				^{11}B NMR ratio[b]	
	$O(Bcat)_2$	B_2cat_2	HOBcat	catechol	$B_2(OR)_4$	$B(OR)_3$
0.5	0	0	18	180	87	13
1.0	0	0	18	180	-	-
2.0	0	0	18	180	-	-
3.0	0	0	24	176	-	-
5.0	0	0	20	180	84	16
10.0	0	0	20	180	86	14

Table S24. Effects of water (5 equiv.) on $O(Bcat)_2$ formation

[a] Yields were determined by ^{1}H NMR using an internal standard. Yields of HOBcat and catechol are based on the molar equivalents of B_2cat_2, with maximum theoretical yields of 200% because two equivalents of HOBcat or catechol can be generated from 1 equivalent of B_2cat_2. [b] The ^{11}B NMR ratio shows the conversion of diboron species [$B_2(OR)_4$] to borate species [$B(OR)_3$] and was determined by integrating the signals at 26-36 ppm (diboron) and 18.5-24.5 ppm (borate). These ratios were only measured at 0.5, 5 and 10 h.

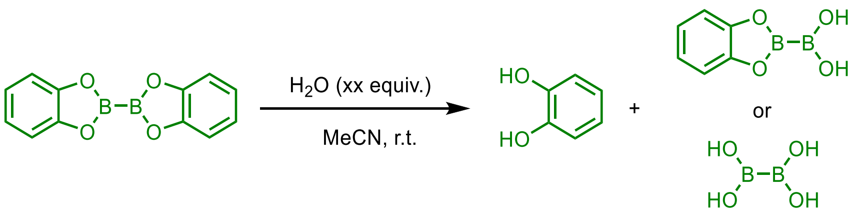

Time (h)	*Yield of catechol (%)*[a]			*B–B bond cleavage (%)*[b]		
	**No water, No $CuCl_2$**	**1 equiv. water, no $CuCl_2$**	**1 equiv. water, 1 equiv. $CuCl_2$**	**No water, No $CuCl_2$**	**1 equiv. water, no $CuCl_2$**	**1 equiv. water, 1 equiv. $CuCl_2$**
0.5	2	47	0	<1	1	67
4	4	84	0	1	2	66
10	4	88	0	1	2	66

Table S25. Control experiment on B–B bond cleavage

[a] The yield of hydrolysis was measured by the amount of catechol formed, which was determined by ^{1}H NMR using dibromomethane as an internal standard. [b] The amount of B–B bond cleavage shows the conversion of diboron species [$B_2(OR)_4$] to borate species [$B(OR)_3$] and was determined by ^{11}B NMR by integrating the signals at 26-36 ppm (diboron) and 18.5-24.5 ppm (borate).

3.5. NMR Studies of the Borylation Reaction

Under an ambient atmosphere, a flame dried 8 mL Biotage® microwave reaction vial equipped with a magnetic stir bar was charged with $CuCl_2$ (40 mg, 0.30 mmol, 1.0 equiv.), and B_2cat_2 (71 mg, 0.30 mmol, 1.0 equiv.). The vial was transferred into an anhydrous, argon-filled glovebox where anhydrous MeCN was added followed by cyclohexane (126 mg, 162 µL, 1.50 mmol, 5.00 equiv.). The vial was sealed with a cap with septum and placed 4 cm away from two purple LEDs (Kessil PR160-390 nm LEDs). The reaction was stirred at a speed of 1200 rpm and irradiated at 40 °C. After specific irradiation times, the portions of the reaction solution were transferred into NMR tubes for ^{11}B NMR analysis.

Figure S16 shows the ^{11}B NMR spectra of the reaction solution containing $CuCl_2$ and B_2cat_2 in CD_3CN at different time points from 1 h to 16 h. After 1 h, B_2cat_2 is observed to be partially converted to HOBcat. As the reaction proceeded, B_2cat_2 was gradually consumed and the formation of boronic ester increased. After 16 h irradiation, B_2cat_2 was fully consumed and only the boronic ester product and $O(Bcat)_2$ were observed. Notably, $O(Bcat)_2$ was not fully consumed. The identity of the unconverted $O(Bcat)_2$ was confirmed by ^1H and ^{13}C NMR, where all spectroscopic data matched those of the independently synthesised $O(Bcat)_2$.

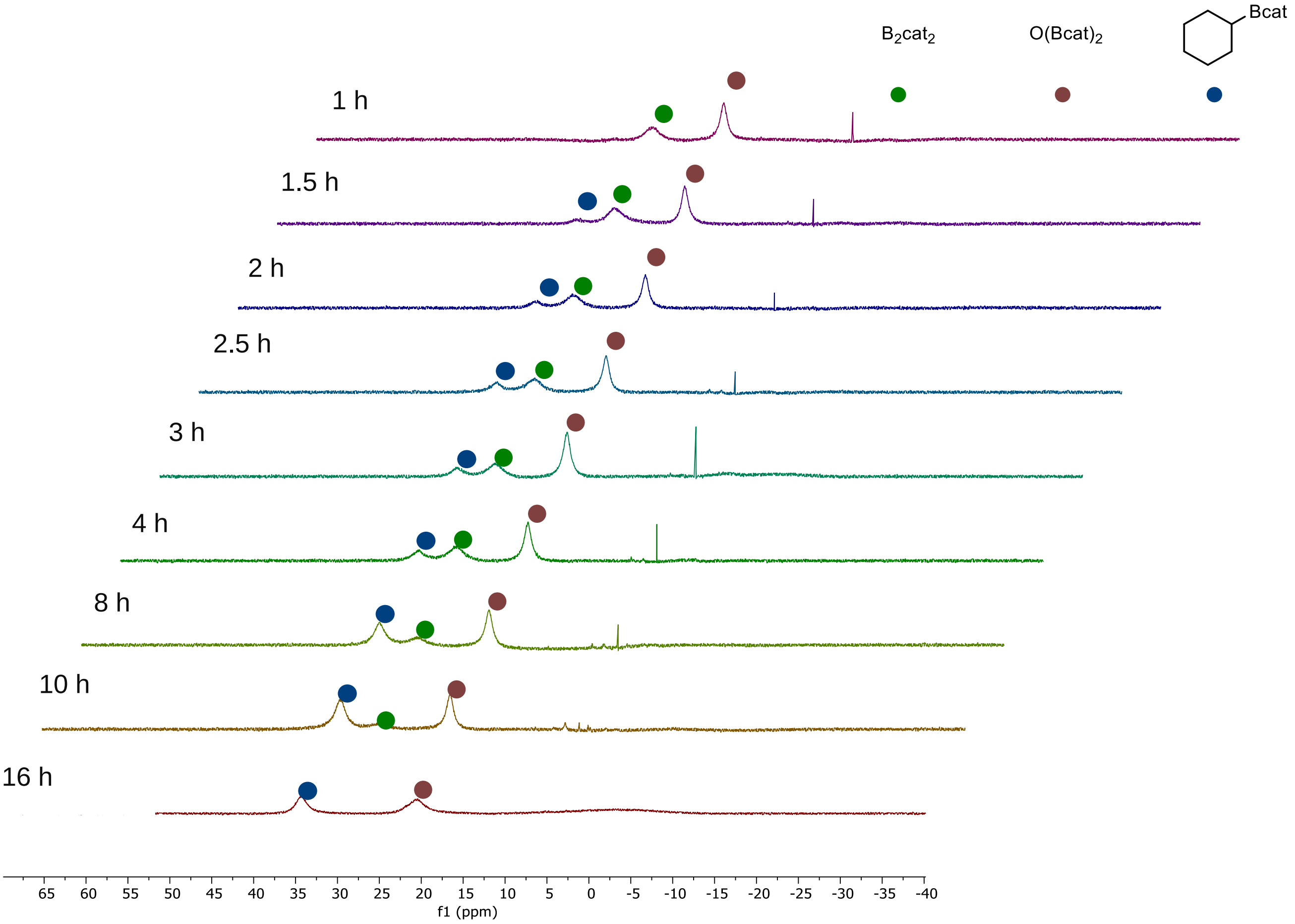

Figure S16. ^{11}B NMR spectra of the borylation reaction in CD_3CN

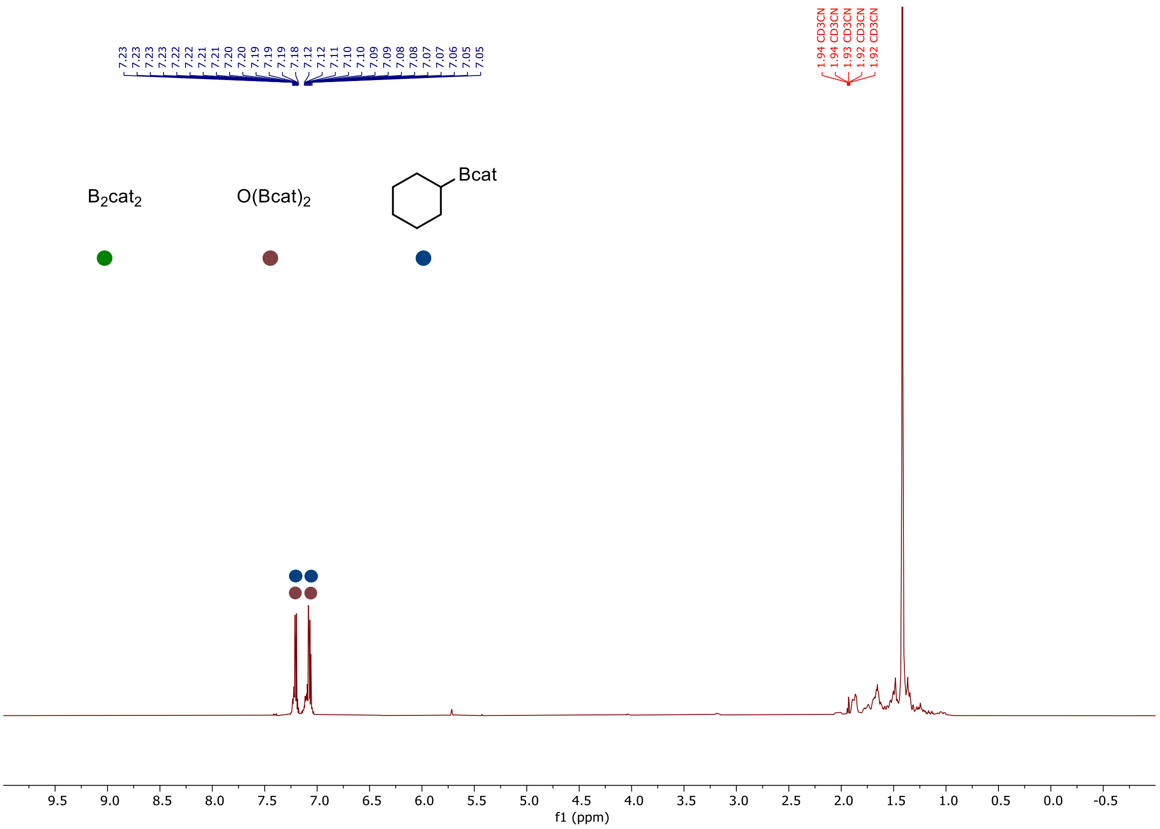

Figure S17. ^{1}H **NMR spectra of the borylation reaction in** CD_3CN **after 16 h irradiation**

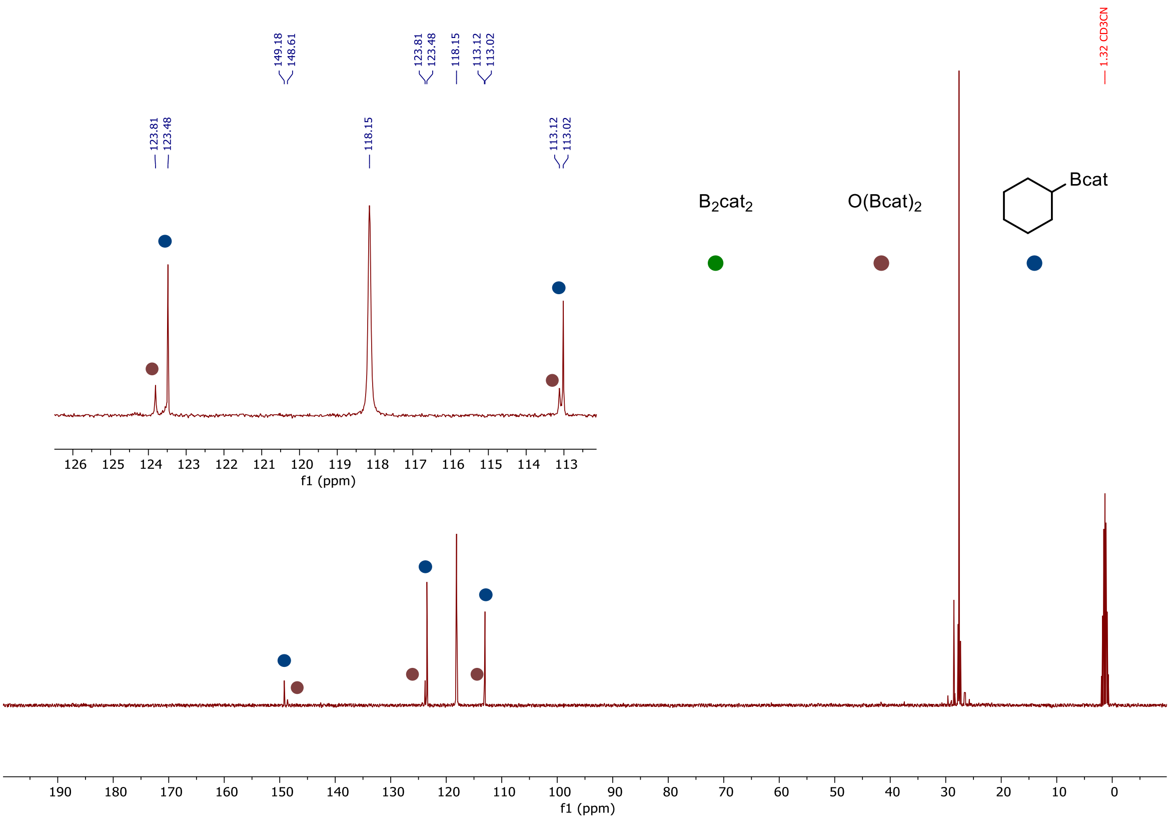

Figure S18. ^{13}C **NMR spectra of the borylation reaction in** CD_3CN **after 16 h irradiation**

3.6. Effect of Portionwise Addition of $CuCl_2$

With 1.0 equivalent of $CuCl_2$:

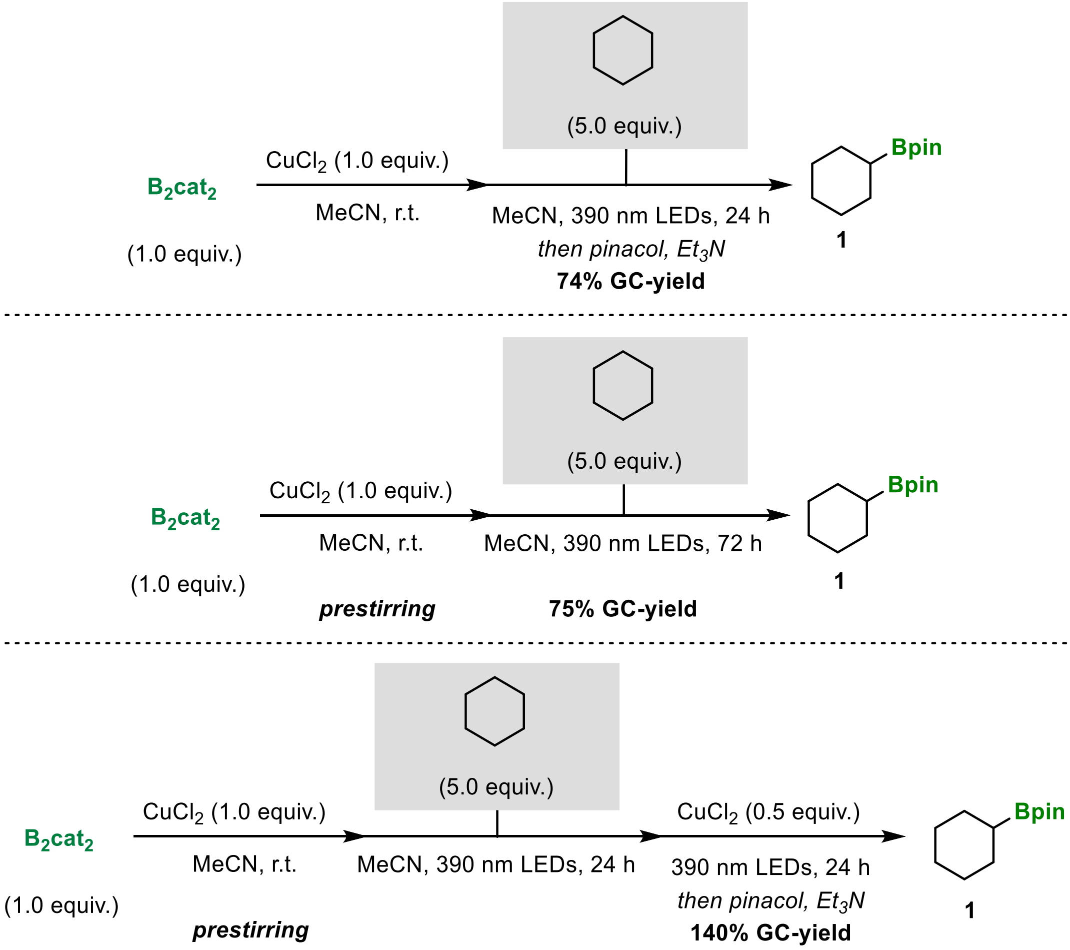

Performed following **General Procedure B**, using cyclohexane (126 mg, 162 µL, 1.50 mmol, 5.00 equiv.), $CuCl_2$ (40 mg, 0.30 mmol, 1.0 equiv.) and B_2cat_2 (71 mg, 0.30 mmol, 1.0 equiv.) in MeCN (1.5 mL, c = 0.20 M), with irradiation times of 24 and 72 h. ^{11}B NMR analysis was performed before the addition of pinacol and Et_3N (Figure S19a). The yield is based on the moles of product per mole of B_2cat_2; a yield above 100% reflects the conversion of the HO(Bcat) intermediate and by-product to the boronic ester desired borylated product.

For the portionwise addition of $CuCl_2$, after the initial 24 h irradiation, the vial was transferred into a glovebox, opened and $CuCl_2$ (20 mg, 0.15 mmol, 0.50 equiv.) added. The vial was re-sealed with a cap with septum, removed from the glovebox, placed 4 cm away from two purple LEDs (Kessil PR160-390 nm LEDs), and irradiated for a further 24 h. A sample was analysed by ^{11}B NMR (Figure S19b). To the combined reaction and NMR sample was added a solution of pinacol (106 mg, 0.900 mmol, 1.50 equiv.) and Et_3N (0.84 mL, 6.0 mmol, 10 equiv.) in DCM (1 mL) and stirring was continued for 1 h. 1,3,5-Trimethoxybenzene (100 mg, 0.600 mmol, 1.00 equiv.) as the internal standard was added. After vigorously shaking for 3 min, the crude reaction mixture was filtered through a short plug of silica and the yield was determined by GC-FID analysis.

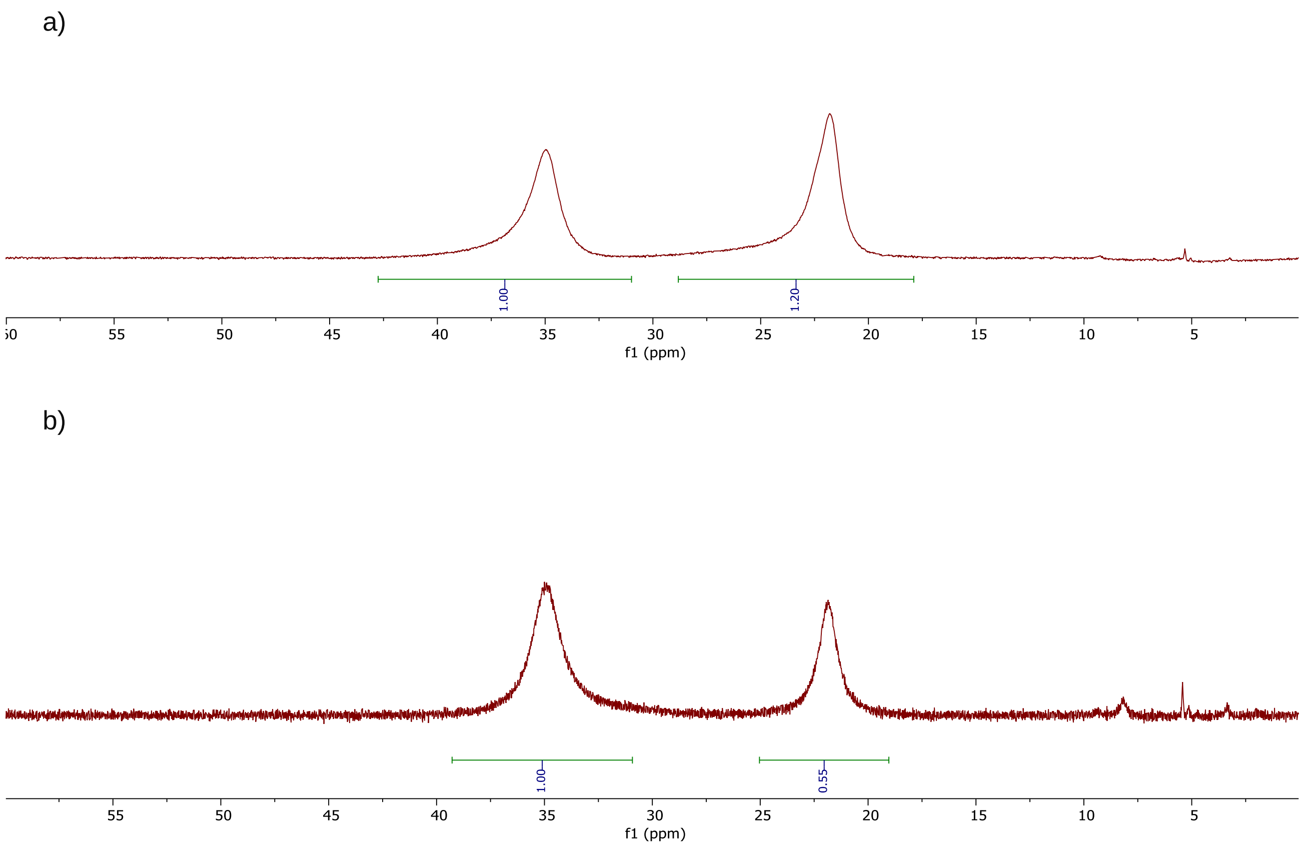

Figure S19. ^{11}B NMR spectra of the borylation reaction with additional $CuCl_2$

a) the reaction after 24 h irradiation; b) the reaction with extra $CuCl_2$ (0.5 equiv.) added after 24 h then irradiated for a further 16 h.

These results show that the boronic ester product was formed in 74% and 75% yield after 24 h and 72 h irradiation, respectively, under **General Procedure B** with 1.0 equivalent of $CuCl_2$. Therefore, simple extension of the reaction time does not increase the yield. With the addition of extra $CuCl_2$ after 24 h irradiation, another 24 h irradiation could give 140% yield of the boronic ester product. The change in relative amounts of boronic ester and $O(Bcat)_2$ was also confirmed by integrating their respective signals in the ^{11}B NMR (Figure S19). This result is consistent with the EPR study in Figure S21, which proves that Cu(II) was fully consumed after 16-24 h irradiation, thus preventing further chlorine radical formation by LMCT of $CuCl_2$. The addition of extra $CuCl_2$ could restart the LMCT process to enable further conversion of $O(Bcat)_2$ and formation of more boronic ester product.

With 0.2 equivalents of $CuCl_2$:

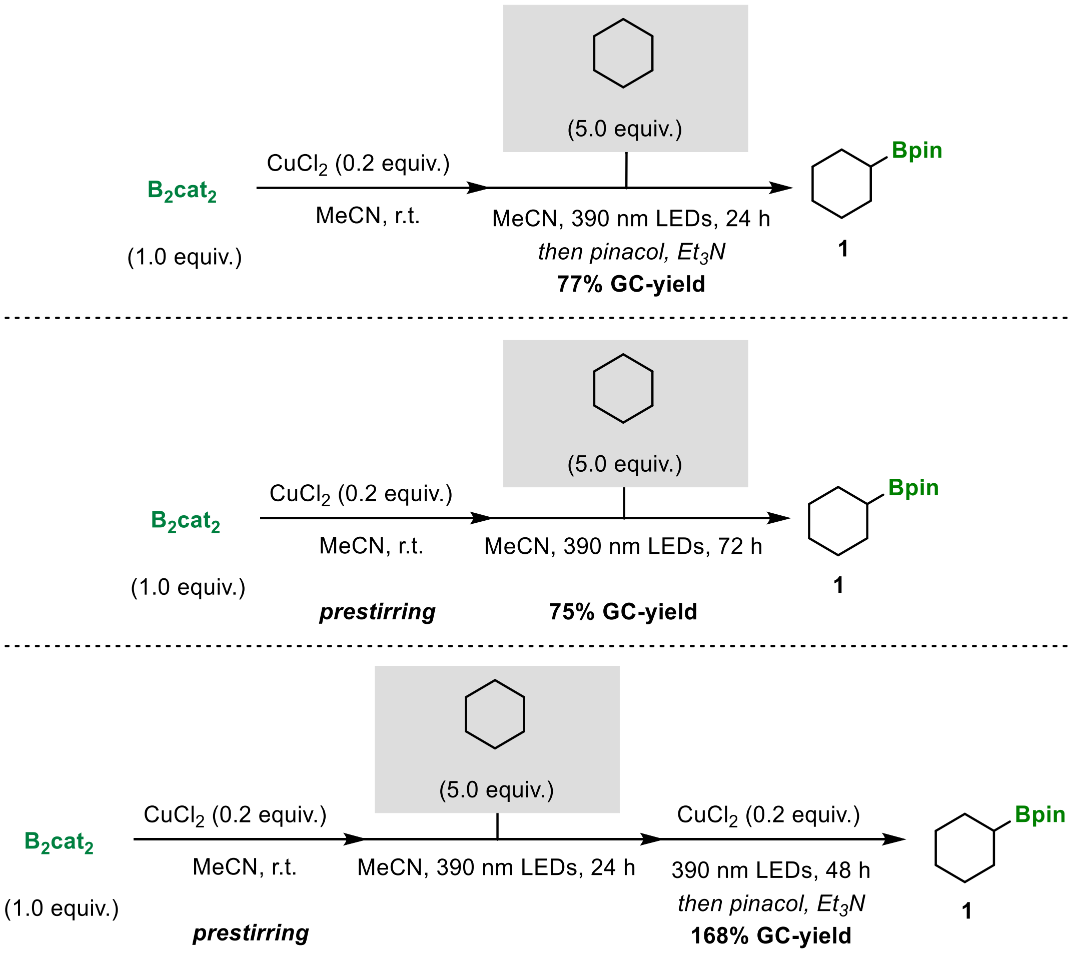

Performed following **General Procedure A-prestirring**, using cyclohexane (126 mg, 162 µL, 1.50 mmol, 5.00 equiv.), $CuCl_2$ (8 mg, 0.06 mmol, 0.20 equiv.) and B_2cat_2 (71 mg, 0.30 mmol, 1.0 equiv.) in MeCN (1.5 mL, *c* = 0.20 M), with a reaction time of 72 h. The yield is based on the moles of product per mole of B_2cat_2; a yield above 100% reflects the conversion of the HO(Bcat) intermediate and by-product to the boronic ester desired borylated product.

For the portionwise addition of $CuCl_2$, after an initial 24 h irradiation, the vial was transferred into a glovebox, opened and $CuCl_2$ (8 mg, 0.06 mmol, 0.20 equiv.) added. The vial was sealed with a cap with septum, placed 4 cm away from two purple LEDs (Kessil PR160-390 nm LEDs), and irradiated for a further 48 h. A solution of pinacol (106 mg, 0.900 mmol, 1.50 equiv.) and Et_3N (0.84 mL, 6.0 mmol, 10 equiv.) in DCM (1 mL) was added and stirring was continued for 1 h. 1,3,5-Trimethoxybenzene (100 mg, 0.600 mmol, 1.00 equiv.) as the internal standard was added. After vigorously shaking for 3 min, the crude reaction mixture was filtered through a short plug of silica and the yield was determined by GC-FID analysis.

These results show that boronic ester product was formed in 77% and 75% yield within 24 h and 72 h irradiation, respectively, under **General Procedure A-prestirring** with 0.2 equivalents of $CuCl_2$. Therefore, simple extension of the reaction time does not increase the yield. With the addition of extra $CuCl_2$ after 24 h irradiation, another 48 h irradiation could give 168% yield of boronic ester product.

3.7. Competition Experiments

3.7.1. Competition experiment with 2,3-dimethylbutane

In the absence of B_2cat_2, following modified literature conditions for the reaction of 2,3-dimethylbutane with ethyl acrylate,[25] alkylated product was formed in 64% yield with 19:81 regioselectivity for functionalisation of primary:tertiary positions. After correcting for the number of hydrogen atoms, this gives a primary:tertiary C–H selectivity of 4:96.

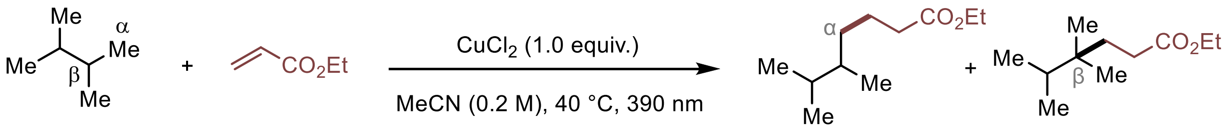

(5.0 equiv.) (1.0 equiv.) **74a** **74b**

64% yield *α:β = 19:81*

Under an ambient atmosphere, a flame dried 8 mL Biotage® microwave reaction vial equipped with a magnetic stir bar was charged with $CuCl_2$ (40 mg, 0.30 mmol, 1.0 equiv.). The vial was transferred into an anhydrous, argon-filled glovebox where anhydrous acetonitrile was added (1.5 mL, *c* = 0.20 M) followed by 2,3-dimethylbutane (129 mg, 1.50 mmol, 5.00 equiv.) and ethyl acrylate (30 mg, 0.30 mmol, 1.0 equiv.) The vial was sealed with a cap with septum and placed 4 cm away from two purple LEDs (Kessil PR160-390 nm LEDs). The reaction was stirred at a speed of 1200 rpm and irradiated for 18 h at 40 °C. Diethyl phthalate as an internal standard was subsequently added. After vigorously shaking for 3 min, the crude reaction mixture was filtered through a short plug of silica and the r.r. (*α*:*β* = 19:81) and yield (64%) of alkylated product **74** were determined by GC-FID analysis (***see spectrum***). The reaction mixture was concentrated *in vacuo* and purified by flash column chromatography, eluting with Et_2O/pentane (5/95) to give **74** (25 mg, 42%,) as a colorless oil.

R*f* =0.5 (25:75 Et_2O/pentane, CAM)

NMR Spectroscopy of 74b (*see spectra*):

^{1}H NMR (400 MHz, $CDCl_3$) δ_H 4.12 (q, *J* = 7.0 Hz, 2H), 2.26 – 2.21 (m, 2H), 1.61 – 1.53 (m, 2H), 1.49 – 1.41 (m, 1H), 1.25 (t, *J* = 7.0 Hz, 3H), 0.87 – 0.81 (m, 6H), 0.81-0.77 (s, 6H).

^{13}C NMR (101 MHz, $CDCl_3$) δ_C 174.8, 60.4, 35.5, 35.1, 34.8, 29.6, 23.9, 17.5, 14.4 ppm.

NMR Spectroscopy of 74a (*see spectra*):

^{1}H NMR (400 MHz, $CDCl_3$) δ_H 4.12 (q, *J* = 7.0 Hz, 2H), 2.30 – 2.26 (m, 2H), 1.61 – 1.53 (m, 3H), 1.25 (t, *J* = 7.0 Hz, 4H), 0.87 – 0.77 (m, 12H).

^{13}C NMR (101 MHz, $CDCl_3$) δ_C 174.0, 60.3, 38.5, 34.9, 33.7, 32.0, 23.2, 20.3, 18.1, 15.4, 14.4 ppm.

IR (film) ν_{max}: 2961, 2875, 1736, 1465, 1369, 1170, 1098, 1027, 857, 777 cm^{-1}.

HRMS (EI$^+$): m/z calc'd for $C_{11}H_{22}O_2$ [M-Me]$^+$, 171.1380; found, 171.1378.

Evidence for the formation of hindered tertiary alkyl radicals was provided upon reacting 2,3-dimethylbutane with B_2cat_2 in the presence of ethyl acrylate. Boronic ester **11** was formed in 50% yield with >97:3 regioselectivity for borylation of the primary positions. Alkylated product was formed in 39% yield with 29:71 regioselectivity for functionalisation of primary:tertiary positions. After correcting for the number of hydrogen atoms, this gives a primary:tertiary C–H selectivity of 24:76.

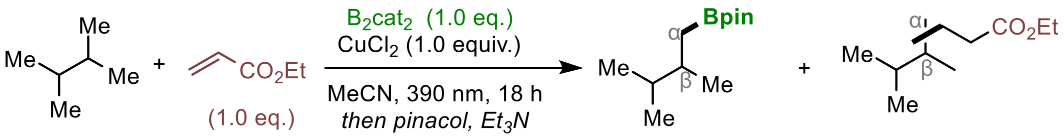

11 **74**

50% yield *α:β* > 97:3 39% yield *α:β* = 29:71

Under an ambient atmosphere, a flame dried 8 mL Biotage® microwave reaction vial equipped with a magnetic stir bar was charged with $CuCl_2$ (40 mg, 0.30 mmol, 1.0 equiv.) and B_2cat_2 (71 mg, 0.30 mmol, 1.0 equiv.). The vial was transferred into an anhydrous, argon-filled glovebox where anhydrous acetonitrile was added (1.5 mL, *c* = 0.20 M) followed by 2,3-dimethylbutane (129 mg, 1.50 mmol, 5.00 equiv.) and ethyl acrylate (30 mg, 0.30 mmol, 1.0 equiv.) The vial was sealed with a cap with septum and placed 4 cm away from two purple LEDs (Kessil PR160-390 nm LEDs). The reaction was stirred at a speed of 1200 rpm and irradiated for 18 h at 40 °C. A solution of pinacol (106 mg, 0.900 mmol, 3.00 equiv.) and Et_3N (0.84 mL, 6.0 mmol, 20 equiv.) in DCM (1 mL) was then added and stirring was continued for 1 h. Diethyl phthalate as an internal standard was subsequently added. After vigorously shaking for 3 min, the crude reaction mixture was filtered through a short plug of silica and the r.r. (*α*:*β* = 29:71) and yield (39%) of alkylated product **74** and the r.r. (*α*:*β* > 97:3) and yield (50%) of borylated product **11** were determined by GC-FID analysis (***see spectrum***). The reaction mixture was concentrated *in vacuo* and purified by flash column chromatography, eluting with Et_2O/pentane (5/95) to give **74** (22 mg, 37%) as a colorless oil and **11** (33 mg, 39%) as a colorless oil.

3.7.2. Competition experiment with THF

Following **General Procedure C** but replacing B_2cat_2 with ethyl acylate gave alkylation product **75** 80% yield with a 86:14 regioselectivity for functionalisation of α:β positions of THF.

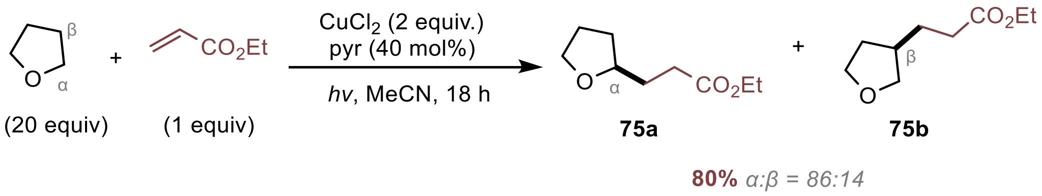

Under an ambient atmosphere, a flame dried 7 mL vial equipped with a magnetic stir bar was charged with $CuCl_2$ (80 mg, 0.60 mmol, 2.0 equiv.). The vial was transferred into an anhydrous, argon-filled glovebox where anhydrous MeCN was added (5.0 mL, *c* = 60 mM), followed by ethyl acylate (30 mg, 33 µL, 0.30 mmol, 1.0 equiv.), THF (432 mg, 486 µL, 6.00 mmol, 20.0 equiv.) and pyridine (9.5 mg, 9.7 µL, 0.12 mmol, 0.40 equiv.). The vial was tightly sealed and then removed from the glovebox. The reaction was stirred at a speed of 1400 rpm and irradiated for 18 h at 35 °C in a PhotoCube® reactor. Diethyl phthalate as an internal standard was subsequently added. After vigorously shaking for 3 min, the crude reaction mixture was filtered through a short plug of silica and the r.r. (86:14 α:β, **75a**:**75b**) and yield (80%) of alkylated product **75** were determined by GC-FID analysis (***see spectrum***). The reaction mixture was then concentrated *in vacuo* and purified by Biotage Isolera® flash purification (SNAP 5 g silica cartridge), eluting with Et_2O/pentane (0 – 5%, v/v), to give **75** (37.6 mg, 71%) as a colorless oil. Only the major product, *α*-isomer (**75a**) is listed for characterization.

R*f* = 0.21 (1:20 EtOAc/pentane, CAM)

NMR Spectroscopy of 75a (*see spectra*):

^{1}H NMR (400 MHz, $CDCl_3$): δ_H 4.10 (q, *J* = 7.0, 2H), 3.93 – 3.76 (m, 2H), 3.73 – 3.62 (m, 1H), 2.48 – 2.28 (m, 2H), 2.16 – 1.93 (m, 1H), 1.93 – 1.69 (m, 4H), 1.52 – 1.38 (m, 1H), 1.23 (t, *J* = 7.0 Hz, 3H) ppm;

^{13}C NMR (101 MHz, $CDCl_3$): δ_C 173.8, 78.3, 67.8, 60.4, 31.3, 30.8, 25.8, 14.3 ppm.

All recorded spectroscopic data matched those previously reported in the literature.[16]

Evidence for the formation of α-oxy radicals was provided upon reacting THF with B_2cat_2 in the presence of ethyl acrylate. Boronic ester **28** was formed in 27% yield with >97:3 regioselectivity for borylation of the β position. Alkylated product **75** was formed in 46% yield with 9:91 regioselectivity for functionalisation of β:α positions.

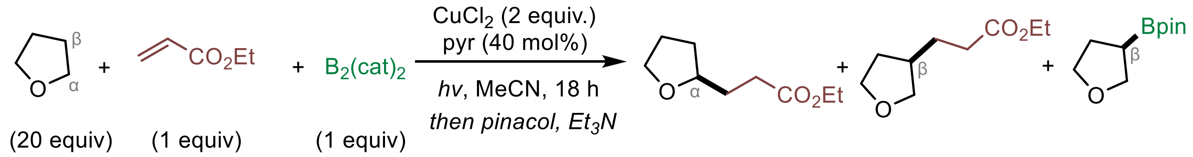

75
46% *α:β = 91:9*

28, 27%

Under an ambient atmosphere, a flame dried 7 mL vial equipped with a magnetic stir bar was charged with $CuCl_2$ (80 mg, 0.60 mmol, 2.0 equiv.) and B_2cat_2 (71 mg, 0.30 mmol, 1.0 equiv.). The vial was transferred into an anhydrous, argon-filled glovebox where anhydrous MeCN were added (5.0 mL, *c* = 60 mM) was added, followed by ethyl acylate (30 mg, 33 μL, 0.30 mmol, 1.0 equiv.), THF (432 mg, 486 μL, 6.00 mmol, 20.0 equiv.) and pyridine (9.5 mg, 9.7 μL, 0.12 mmol, 0.40 equiv.). The vial was tightly sealed and then moved from the glovebox. The reaction was stirred at a speed of 1400 rpm and irradiated for 18 h at 35 °C in a PhotoCube® reactor. A solution of pinacol (106 mg, 0.900 mmol, 3.00 equiv.) and Et_3N (0.84 mL, 6.0 mmol, 20 equiv.) in DCM (1 mL) was then added and stirring was continued for 1 h. Diethyl phthalate as an internal standard was subsequently added. After vigorously shaking for 3 min, the crude reaction mixture was filtered through a short plug of silica and the r.r. (91:9 α:β) and yield (46%) of alkylated product **75** and the yield (27%) of borylated product **28** were determined by GC-FID analysis (***see spectrum***).

3.7.3. Reaction monitoring of the competition reaction with 2,3-dimethylbutane.

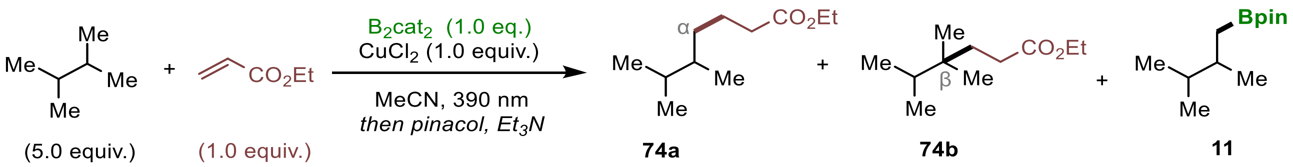

Entry	Time (h)	Yield[a]			*α* (**11+74a**):*β* (**74b**)
		**74a**	**74b**	**11**	
1	0.5	0.1%	1.1%	2%	1.9:1
2	1	0.4%	4%	7%	1.9:1
3	1.5	0.3%	3%	6%	1.9:1
4	2	0.6%	7%	13%	1.8:1
5 [b]	3	0.6-0.8%	6-8%	11-13%	1.7:1-1.9:1
6	4	0.6%	8%	13%	1.7:1
7 [b]	6	1.1-1.5%	10-15%	23-29%	2-2.4:1
8 [b]	8	1.3-2.7%	16-21%	27-29%	1.8:1-2:1
9 [b]	18	11%	28%	50%	2.2:1
10 [b]	24	15-16%	32-35%	45-51%	1.9:1
11 [c]	18	12%	52%	-	1:4.3
12 [d]	18	21%	58%	93%	2:1

Table S26. Time study for the competition reaction with 2,3-dimethylbutane

[a] The yield was determined by GC-FID analysis using diethyl phthalate as an internal standard. [b] Reaction performed twice. [c] Reaction performed in the absence of B_2cat_2. [d] Reaction performed with pre-stirring of B_2cat_2 and $CuCl_2$ for 8 h.

Procedure: Under an ambient atmosphere, a flame dried 8 mL Biotage® microwave reaction vial equipped with a magnetic stir bar was charged with $CuCl_2$ (40 mg, 0.30 mmol, 1.0 equiv.) and B_2cat_2 (71 mg, 0.30 mmol, 1.0 equiv.). The vial was transferred into an anhydrous, argon-filled glovebox where anhydrous acetonitrile was added (1.5 mL, c = 0.20 M) followed by 2,3-dimethylbutane (129 mg, 1.50 mmol, 5.00 equiv.) and ethyl acrylate (30 mg, 0.30 mmol, 1.0 equiv.). The vial was sealed with a cap with septum and placed 4 cm away from two purple LEDs (Kessil PR160-390 nm LEDs) and the reaction was stirred at a speed of 1200 rpm. After specific irradiation time (0.5-18 h), a solution of pinacol (106 mg, 0.900 mmol, 3.00 equiv.) and Et_3N (0.84 mL, 6.0 mmol, 20 equiv.) in DCM (1 mL) was added and stirring was continued for 1 h. Diethyl phthalate as an internal standard was subsequently added. After vigorously shaking for 3 min, the crude reaction mixture was filtered through a short plug of silica and transferred into a GC vial for GCMS-FID analysis to determine the regioselectivity and the yield of alkylated product **74** and borylated product **11**.

For entry 11 with pre-stirring: Under an ambient atmosphere, a flame dried 8 mL Biotage® microwave reaction vial equipped with a magnetic stir bar was charged with $CuCl_2$ (8 mg, 0.06 mmol, 0.2 equiv.) and B_2cat_2 (71 mg, 0.30 mmol, 1.0 equiv.). The vial was transferred into an anhydrous, argon-filled glovebox where anhydrous acetonitrile was added (1.5 mL, *c* = 0.20 M) and the vial was sealed by a Suba Seal septum. After stirring in the glovebox for 8 h, 2,3-dimethylbutane (129 mg, 1.50 mmol, 5.00 equiv.) and ethyl acrylate (30 mg, 0.30 mmol, 1.0 equiv.) were added. The vial was subsequently sealed with a cap with septum and placed 4 cm away from two purple LEDs (Kessil PR160-390 nm LEDs). The reaction was stirred at a speed of 1200 rpm and irradiated for 18 h at 40 °C. A solution of pinacol (106 mg, 0.900 mmol, 3.00 equiv.) and Et_3N (0.84 mL, 6.0 mmol, 20 equiv.) in DCM (1 mL) was then added and stirring was continued for 1 h. Diethyl phthalate as an internal standard was subsequently added. After vigorously shaking for 3 min, the crude reaction mixture was filtered through a short plug of silica and transferred into a vial for GCMS-FID analysis to determine the regioselectivity and the yield of alkylated product **74** and borylated product **11**.

3.7.4. Evidence for oxidation of tertiary alkyl radical intermediates

Further evidence for the formation of tertiary alkyl radicals was provided by the observation of trace amounts of alkene side-products by GC-MS analysis of the crude reaction mixture (see Figure S20 and the following GC-MS traces).

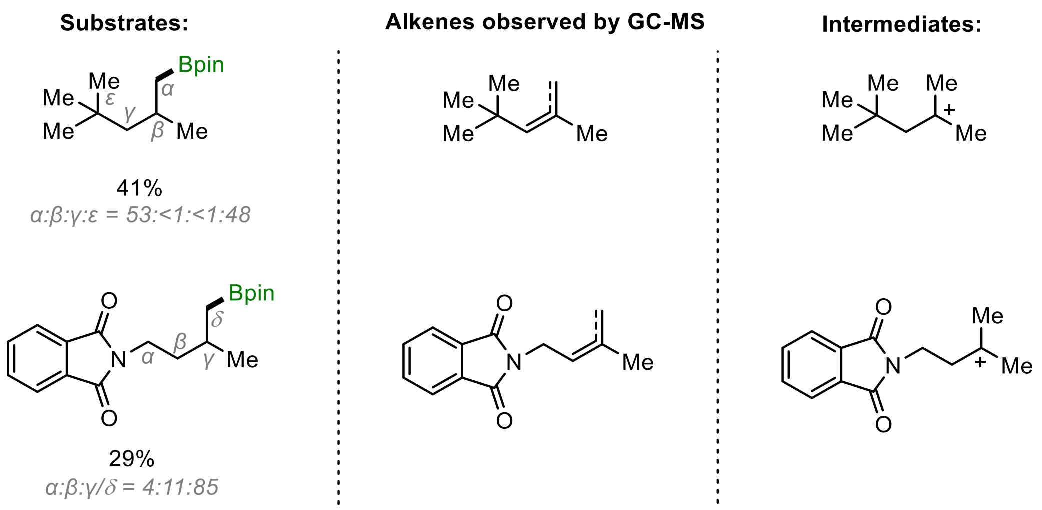

Figure S20. Observation of alkene side-products derived from tertiary alkyl radicals

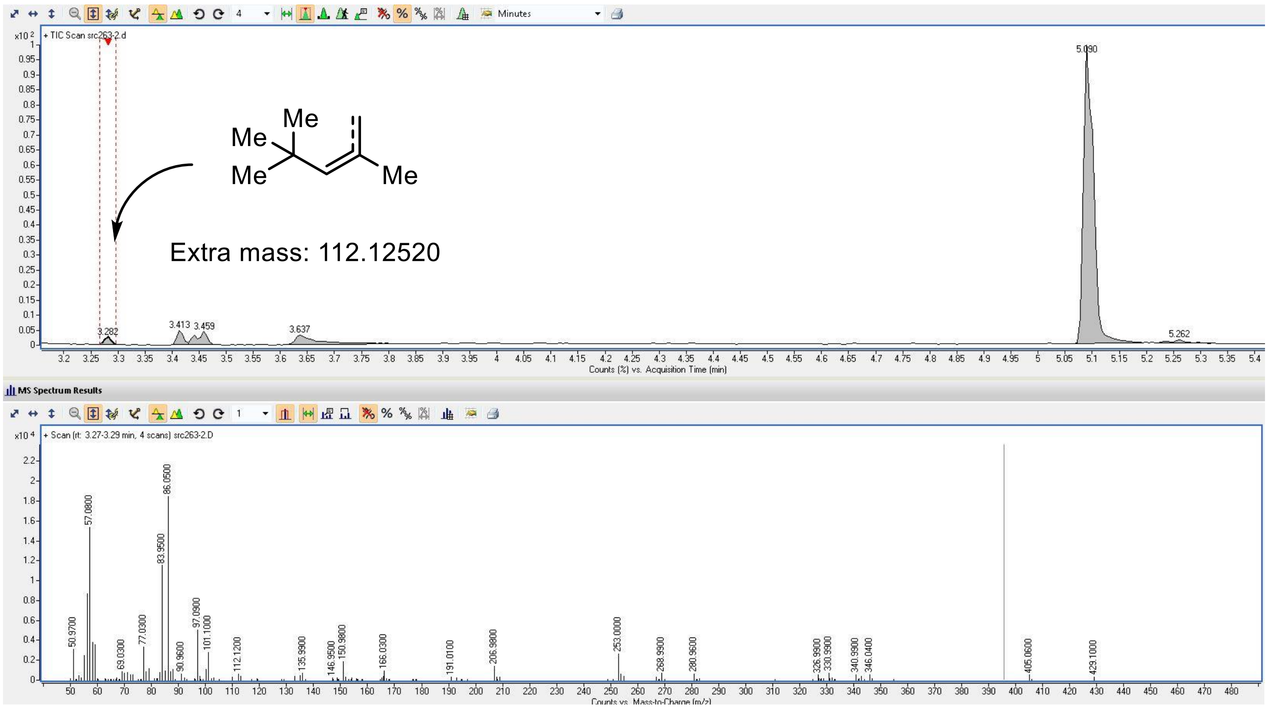

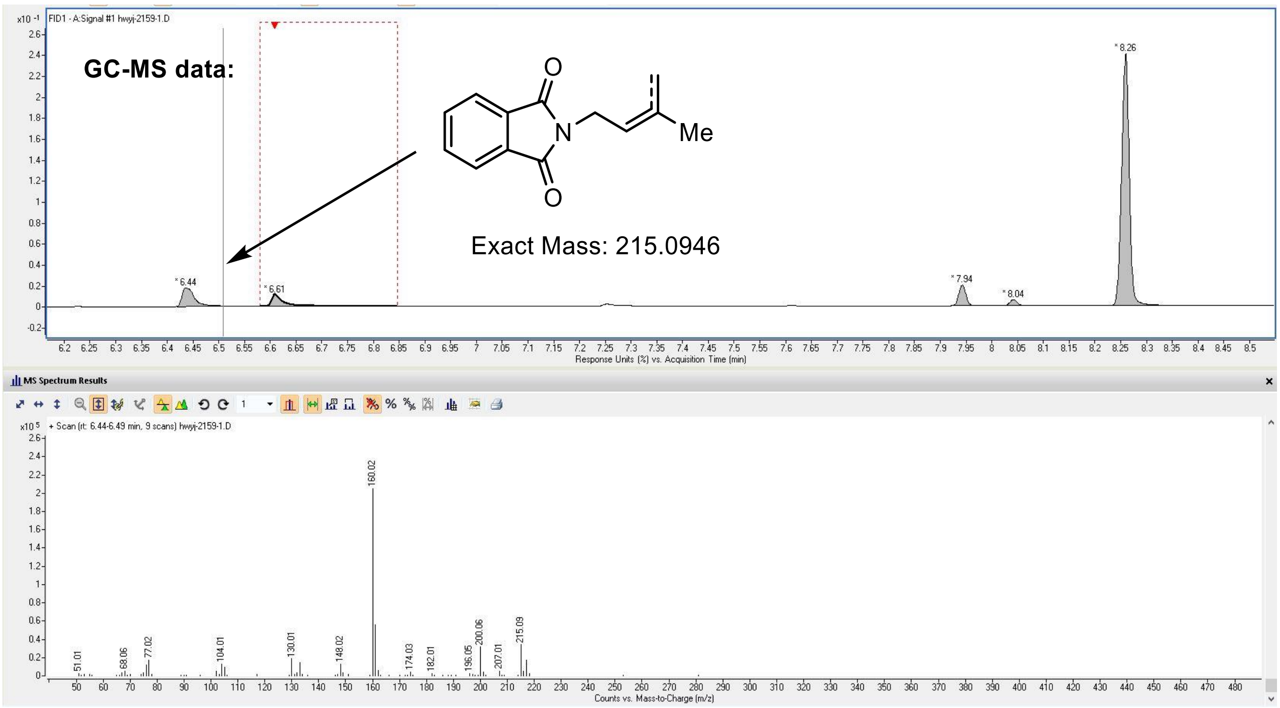
GC-MS data:
Me
Exact Mass: 215.0946
FID1 - A:Signal #1 hwyj-2159-1.D
Response Units (%) vs. Acquisition Time (min)
MS Spectrum Results
+ Scan (rt: 6.44-6.49 min, 9 scans) hwyj-2159-1.D
160.02
215.09
200.06
Counts vs. Mass-to-Charge (m/z)

3.7.5. Evidence for oxidation of benzylic radical intermediates

To support the hypothesis that oxidation of benzylic radicals to carbocations (pathway III) outcomes borylation, a nucleophile trapping study was conducted. MeCN was utilized as the nucleophile to trap the carbocations via a Ritter-type reaction. In the reaction of ethylbenzene, the amide trapping product was observed by GC-MS and isolated, which provides evidence for the formation and subsequent oxidation of benzylic radicals.

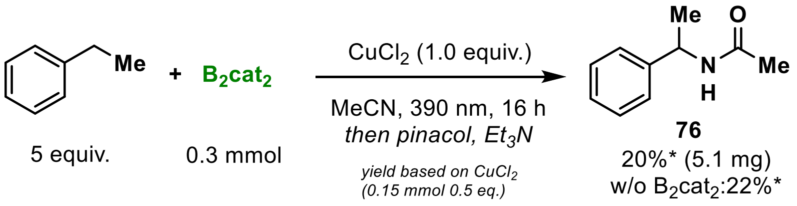

76
20%* (5.1 mg)
w/o B_2cat_2:22%*

Prepared following **General Procedure B**, using distilled ethylbenzene (159 mg, 183 μL, 1.50 mmol, 5.00 equiv.), $CuCl_2$ (40 mg, 0.30 mmol, 1.0 equiv.), and B_2cat_2 (71 mg, 0.30 mmol, 1.0 equiv.) in MeCN (3.0 mL, *c* = 0.10 M). Biotage Isolera™ flash purification on silica gel (SNAP 5 g silica cartridge), eluting with EtOAc/pentane (10 – 50%, v/v), gave **76** (5.1mg, 21%) as a colorless oil.

Prepared following **General Procedure B** without B_2cat_2, Ritter-type product **76** was isolated in 22% yield (5.4 mg).

R_f = 0.40 (1:2 EtOAc/pentane, UV)

NMR Spectroscopy (*see spectra*):

> **^{1}H NMR** (400 MHz, $CDCl_3$): δ_H 1H 7.52 – 7.22 (m, 5H), 5.70 (brs, 1H), 5.13 (q, *J* = 7.4 Hz, 1H), 1.99 (s, 3H), 1.49 (d, *J* = 6.9 Hz, 3H) ppm;
>
> **^{13}C NMR** (101 MHz, $CDCl_3$): δ_C 169.3, 143.2, 128.8, 127.6, 126.4, 49.0, 23.6, 21.8 ppm.

All recorded spectroscopic data matched those previously reported in the literature. [26]

3.8. EPR Studies

Spectrometer Details: Electron paramagnetic resonance (EPR) spectra were recorded using an Active Spectrum X-band Micro-ESR Spectrometer.

Run Details: O.D. 2 mm, L 250 mm Wilmad® quartz (CFQ) EPR tubes, 10 scans, 0 ms delay, field sweep 3000–4000 G, microwave power 50 mW (as indicated on spectra), modulation amplitude 100%, microwave frequency 9.78625 GHz, reflected power –13.74 dBm, temperature 25°C.

Sample preparation:

a) $CuCl_2$-dark-18h

Under an ambient atmosphere, a flame dried 8 mL Biotage® microwave reaction vial equipped with a magnetic stir bar was charged with $CuCl_2$ (14 mg, 0.10 mmol, 1.0 equiv.). The vial was transferred into an anhydrous, argon-filled glovebox where anhydrous acetonitrile was added (5.0 mL, c = 20 mM). The vial was sealed with a cap with septum and covered with foil to protect it from ambient light. The reaction was allowed to stir at room temperature in the dark for 18 h before a sample was transferred into an EPR tube. The tube was removed from the glovebox for EPR analysis (Figure S21).

b) $CuCl_2$-*hv*-18h

Under an ambient atmosphere, a flame dried 8 mL Biotage® microwave reaction vial equipped with a magnetic stir bar was charged with $CuCl_2$ (14 mg, 0.10 mmol, 1.0 equiv.). The vial was transferred into an anhydrous, argon-filled glovebox where anhydrous acetonitrile was added (5.0 mL, c = 20 mM). The vial was sealed with a cap with septum, removed from the glovebox, and placed 4 cm away from two purple LEDs (Kessil PR160-390 nm LEDs). The reaction was stirred at a speed of 1200 rpm and irradiated for 18 h at 40 °C. The vial was transferred back into the glovebox and a sample of the reaction was transferred into an EPR tube. The tube was removed from the glovebox for EPR analysis (Figure S21).

c) 1:1:5 $CuCl_2$/B_2cat_2/cyclohexane-*hv*-18h

Under an ambient atmosphere, a flame dried 8 mL Biotage® microwave reaction vial equipped with a magnetic stir bar was charged with $CuCl_2$ (14 mg, 0.10 mmol, 1.0 equiv.) and B_2cat_2 (24 mg, 0.10 mmol, 1.0 equiv.). The vial was transferred into an anhydrous, argon-filled glovebox where anhydrous acetonitrile was added (5 mL, *c* = 20 mM) followed by cyclohexane (42.1 mg, 54.6 µL, 0.500 mmol, 5.00 equiv.). The vial was sealed with a cap with septum, removed from the glovebox, and placed 4 cm away from two purple LEDs (Kessil PR160-390 nm LEDs). The reaction was stirred at a speed of 1200 rpm and irradiated for 18 h at 40 °C. The vial was transferred back into the glovebox and a sample of the reaction was transferred into an EPR tube. The tube was removed from the glovebox for EPR analysis (Figure S21).

d) 1:1 $CuCl_2$/B_2cat_2-dark-18h

Under an ambient atmosphere, a flame dried 8 mL Biotage® microwave reaction vial equipped with a magnetic stir bar was charged with $CuCl_2$ (14 mg, 0.10 mmol, 1.0 equiv.) and B_2cat_2 (24 mg, 0.10 mmol, 1.0 equiv.). The vial was transferred into an anhydrous, argon-filled glovebox where then anhydrous acetonitrile was added (50

mL, *c* = 20 mM). The vial was sealed with a cap with septum and covered with foil to protect it from ambient light. The reaction was allowed to stir at room temperature in the dark for 18 h before a sample was transferred into an EPR tube. The tube was removed from the glovebox for EPR analysis (Figure S21).

e) 1:1 $CuCl_2$/B_2cat_2-*hv*-18h

Under an ambient atmosphere, a flame dried 8 mL Biotage® microwave reaction vial equipped with a magnetic stir bar was charged with $CuCl_2$ (14 mg, 0.10 mmol, 1.0 equiv.) and B_2cat_2 (24mg, 0.10 mmol, 1.0 equiv.). The vial was transferred into an anhydrous, argon-filled glovebox where anhydrous acetonitrile was added (5 mL, *c* = 20 mM). The vial was sealed with a cap with septum, removed from the glovebox, and placed 4 cm away from two purple LEDs (Kessil PR160-390 nm LEDs). The reaction was stirred at a speed of 1200 rpm and irradiated for 18 h at 40 °C. The vial was transferred back into the glovebox and a sample of the reaction was transferred into an EPR tube. The tube was removed from the glovebox for EPR analysis (Figure S21).

f) 1:5 $CuCl_2$/cyclohexane-*hv*-18h

Under an ambient atmosphere, a flame dried 8 mL Biotage® microwave reaction vial equipped with a magnetic stir bar was charged with $CuCl_2$ (14 mg, 0.10 mmol, 1.0 equiv.). The vial was transferred into an anhydrous, argon-filled glovebox where anhydrous acetonitrile was added (5.0 mL, *c* = 20 mM) followed by cyclohexane (42.1 mg, 54.6 μL, 0.500 mmol, 5.00 equiv.). The vial was sealed with a cap with septum, removed from the glovebox, and placed 4 cm away from two purple LEDs (Kessil PR160-390 nm LEDs). The reaction was stirred at a speed of 1200 rpm and irradiated for 18 h at 40 °C. The vial was transferred back into the glovebox and a sample of the reaction was transferred into an EPR tube. The tube was removed from the glovebox for EPR analysis (Figure S21).

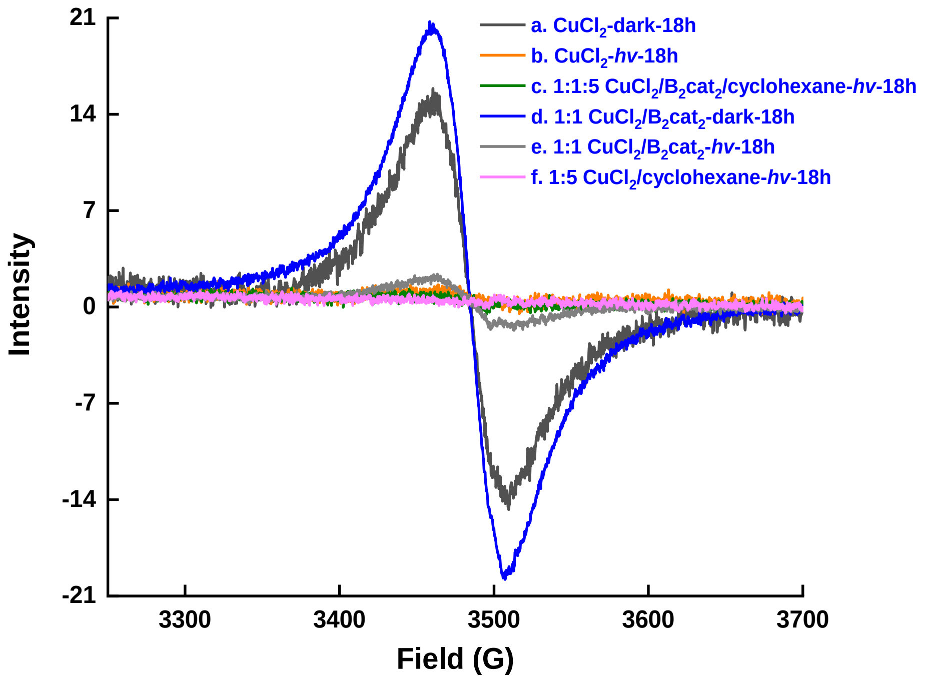

Figure S21. X-band EPR measurements recorded at 25 °C

The EPR measurements show the signal for a solution of $CuCl_2$ (0.1 mmol, 1 equiv.) in MeCN (5 mL, *c* = 0.02 M) without irradiation (Figure S21, a). A slightly stronger signal was observed with the addition of B_2cat_2 (0.1 mmol, 1 equiv.), which shows no reduction of Cu(II) by B_2cat_2 occurred (Figure S21, d). Irradiation both with and without cyclohexane resulted in almost complete loss of the Cu(II) signals due to reduction of Cu(II) to Cu(I) (Figure S21, b,c,f). A decrease in signal intensity was also observed for irradiation in the presence of B_2cat_2 (Figure S21, e).

3.9. Kinetic Isotope Effect Experiments

3.9.1. Determination of the KIE for the borylation of THF

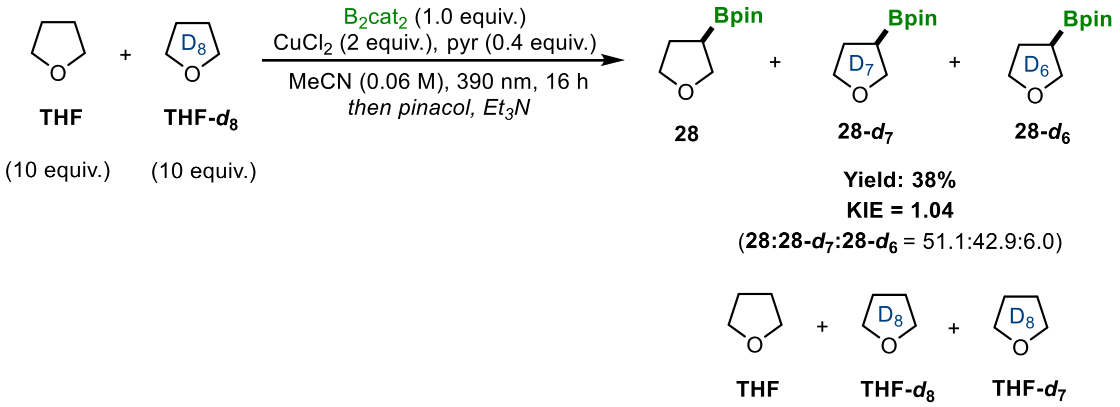

Prepared using **General Procedure C**, from a 1:1 mixture of **THF** and **THF-*d*$_8$** (10 + 10 equiv.). The crude reaction mixture was filtered through a short plug of silica and transferred into a GC vial for HR-GCMS analysis. The H/D exchange product **28-*d*$_6$** was observed by EI-GCMS (Figure S22) and is indicative of reversible HAT. The ratio of **28-*d*$_7$** and **28-*d*$_6$** (87.7:12.3) was determined by EI-GCMS using the ^{11}B isotope peaks (Figure S23). In addition, **THF-*d*$_7$** was observed by EI-GCMS (Figure S24), which is also indicative of reversible HAT. However, the relative ratios of **THF-*d*$_8$** and **THF-*d*$_7$** could not be determined due to overlapping signals. Similarly, we could not determine whether any **THF-*d*$_1$** was formed.

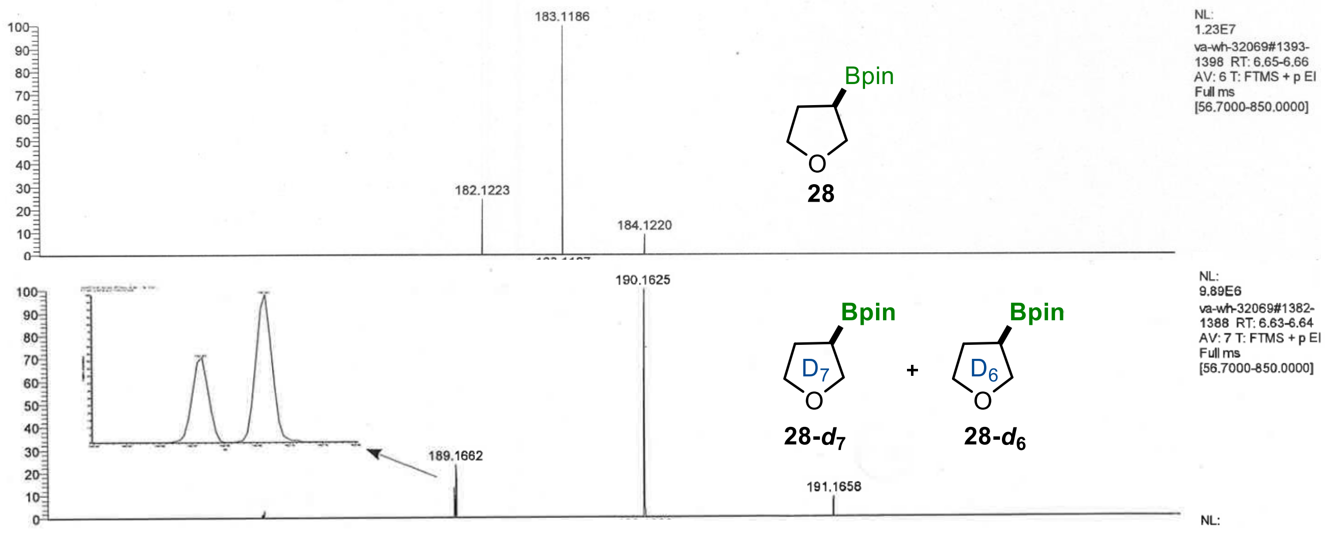

Figure S22. HR-GCMS trace for the KIE experiments with THF

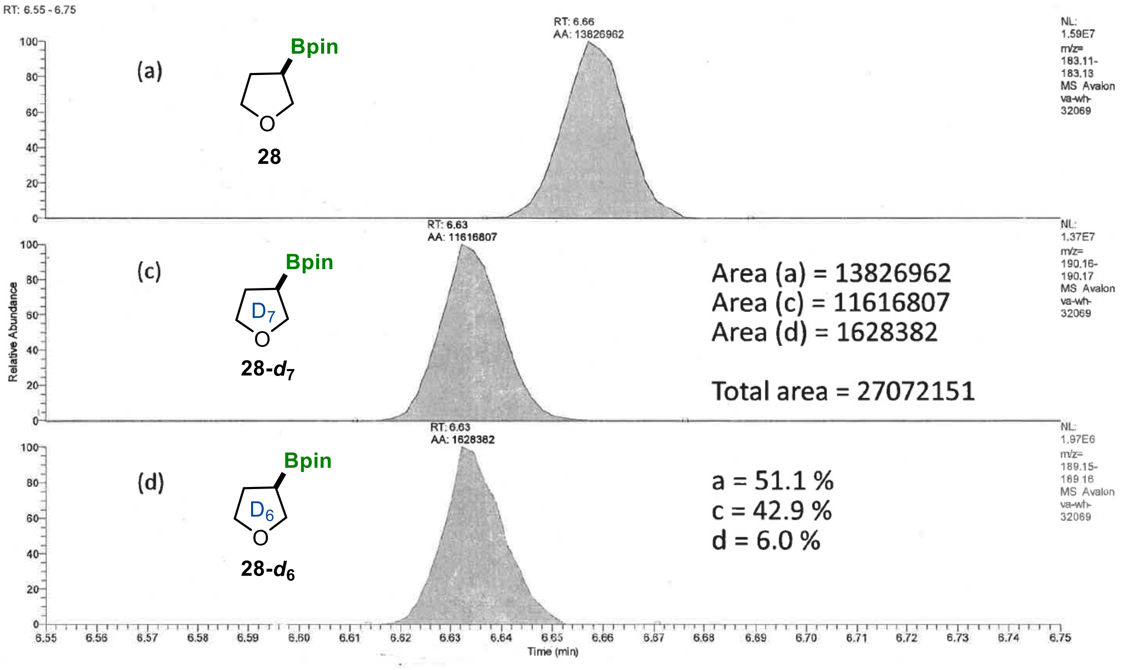

Figure S23. HR-GCMS quantification for the KIE experiments with THF

The ratio of products was determined by ultra-high-resolution GC-MS using an Orbitrap mass analyzer. The ultra-high-resolution in the mass dimension enables analysis of the data using exact mass extracted ion chromatography, which displays peak areas in the chromatogram based on their exact mass. With this method, we can separate ions in the mass dimension even when they overlap in the chromatogram.

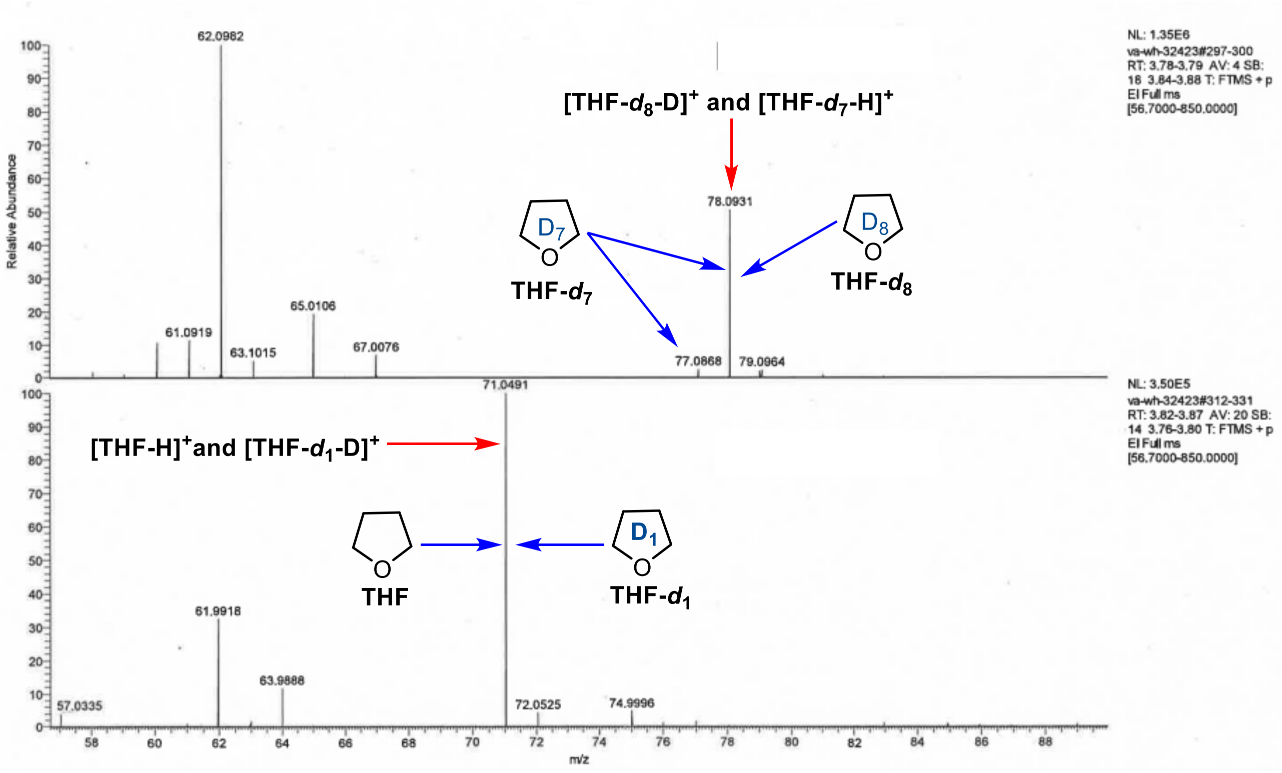

Figure S24. HR-GCMS trace for the recovery of the starting material

3.9.2. Determination of the KIE for the alkylation of THF with ethyl acrylate

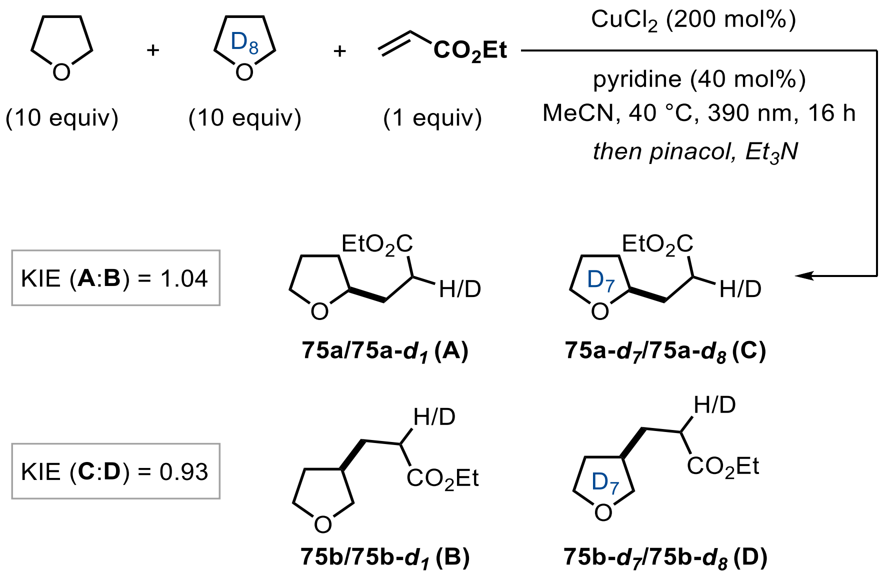

Under an ambient atmosphere, a flame dried 7 mL vial equipped with a magnetic stir bar was charged with $CuCl_2$ (80 mg, 0.60 mmol, 2.0 equiv.). The vial was transferred into an anhydrous, argon-filled glovebox where anhydrous MeCN was added (5.0 mL, *c* = 60 mM followed by ethyl acrylate (30 mg, 33 µL, 0.30 mmol, 1.0 equiv.), THF (216 mg, 243 µL, 3.00 mmol, 10.0 equiv.), THF-d_8 (240 mg, 244 µL, 3.00 mmol, 10.0 equiv.) and pyridine (9.5 mg, 9.7 µL, 0.12 mmol, 0.40 equiv.). The vial was tightly sealed and then removed from the glovebox. The reaction was stirred at a speed of 1400 rpm and irradiated for 16 h at 40 °C in a PhotoCube® reactor. The crude reaction mixture was filtered through a short plug of silica and transferred into a GC vial for HR-GCMS analysis. The alkylated products **75a** and **75b** were observed by EI-GCMS (Figure S25) and the ratio was determined by EI-GCMS using the ^{11}B isotope peaks (Figure S26).

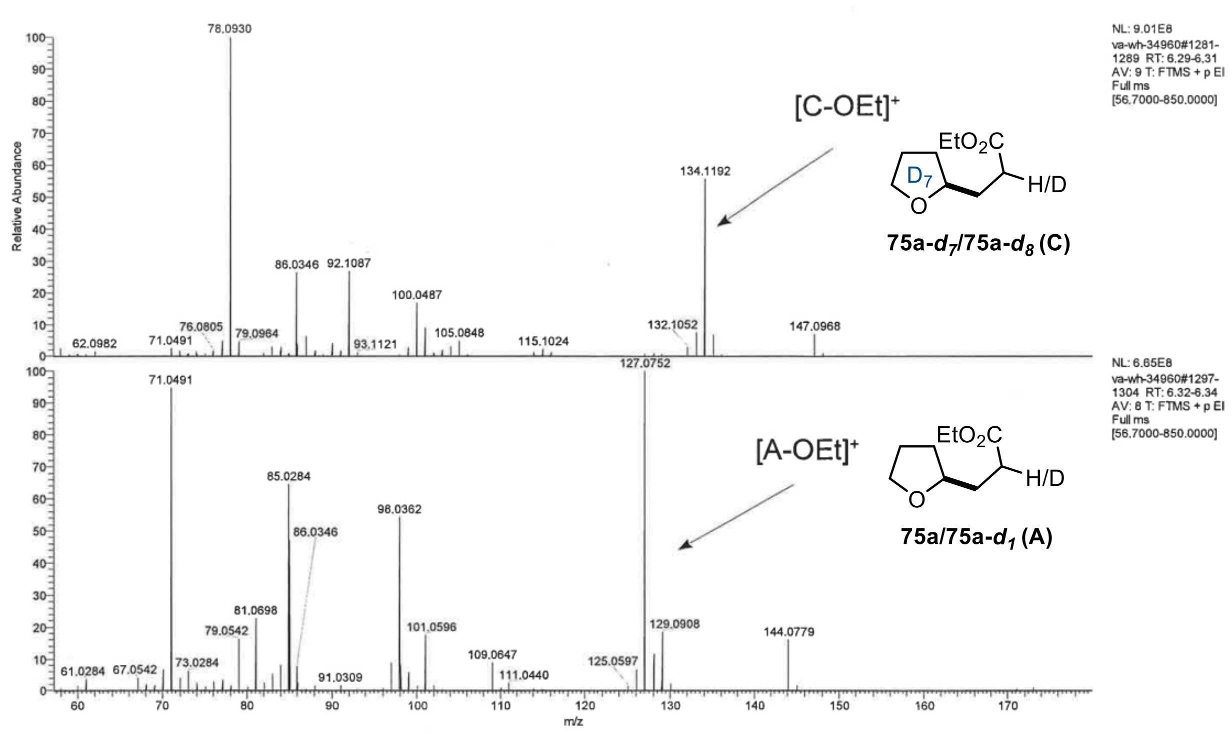

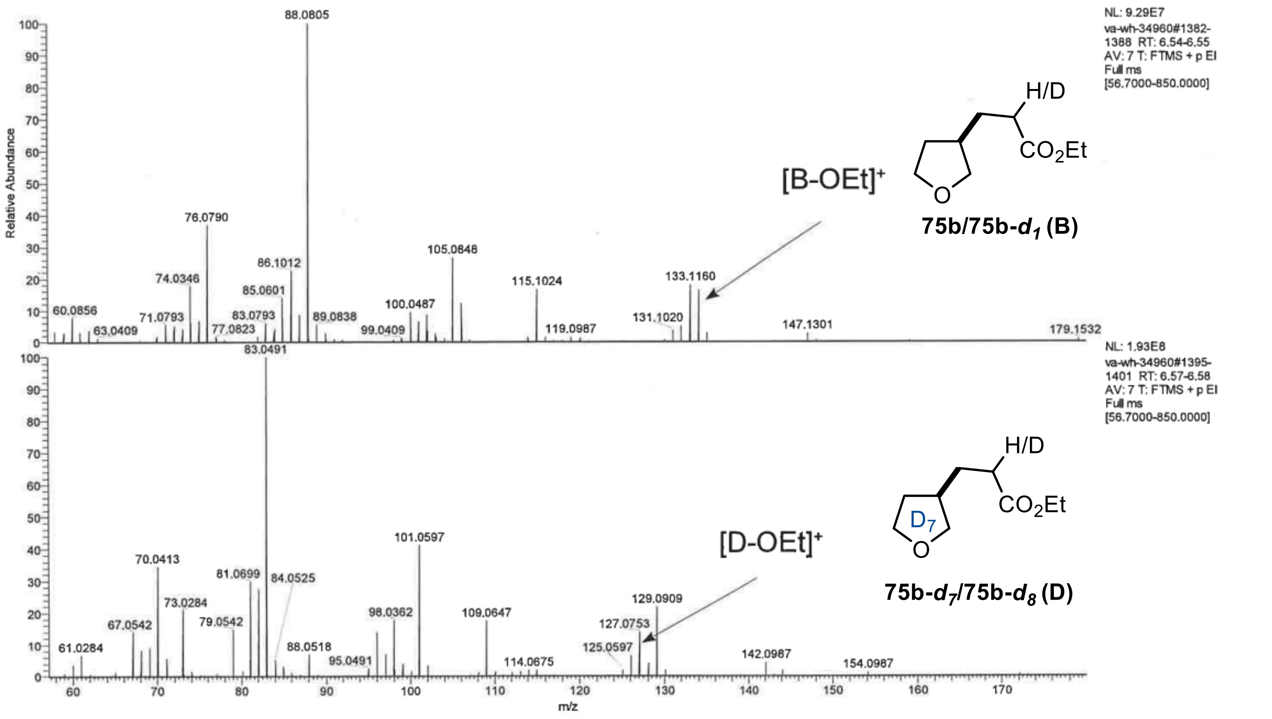

Figure S25. HR-GCMS trace for the KIE experiments with THF and ethyl acrylate

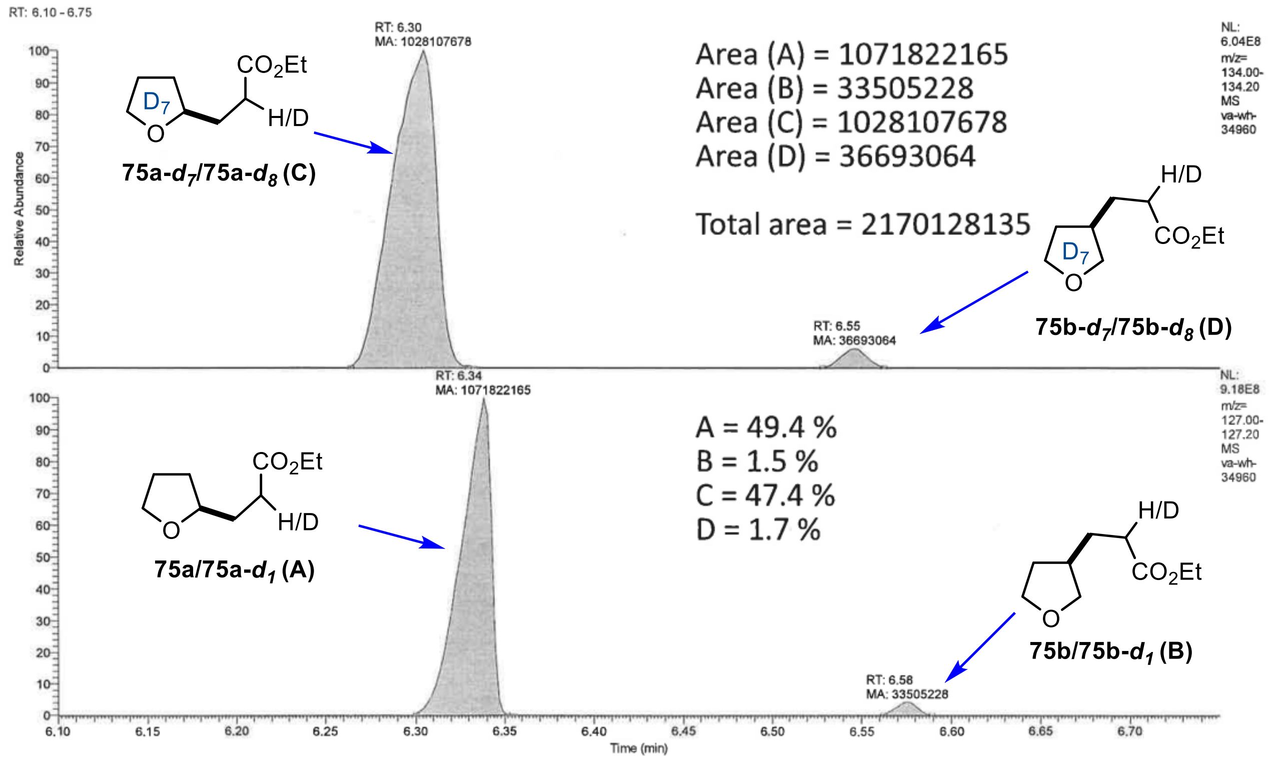

Figure S26. HR-GCMS quantification for the KIE experiments with THF and ethyl acrylate

The ratio of products was determined by ultra-high-resolution GC-MS using an Orbitrap mass analyzer. The ultra-high-resolution in the mass dimension enables analysis of the data using exact mass extracted ion chromatography, which displays peak areas in the chromatogram based on their exact mass. With this method, we can separate ions in the mass dimension even when they overlap in the chromatogram.

3.9.3. Determination of the KIE for the borylation of cyclohexane

Using General Procedure A:

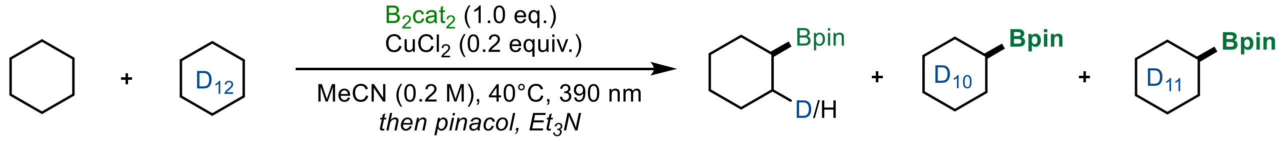

(5.0 equiv.) (5.0 equiv.) **1**, 36.4% **1-*d*$_{10/11}$**, 33.3%

KIE = 1.09

Prepared using **General Procedure A**, from a 1:1 mixture of cyclohexane and cyclohexane-d_{12} (total alkane = 5 + 5 equiv.). 1,3,5-Trimethoxybenzene as an internal standard was subsequently added. After vigorously shaking for 3 min, the crude reaction mixture was filtered through a short plug of silica for GC-FID analysis (***see spectrum***).

Using General Procedure A-Prestirring:

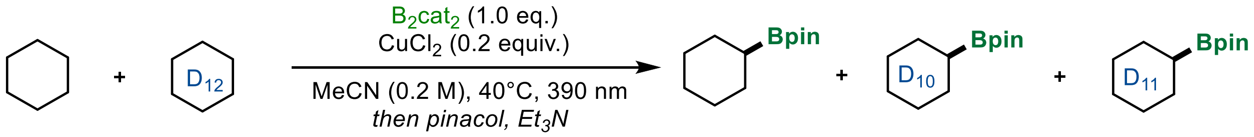

(5.0 equiv.) (5.0 equiv.) **1**, 49.2% **1-*d*$_{10/11}$**, 43.8%

KIE = 1.12

Prepared using **General Procedure A-prestirring**, from a 1:1 mixture of cyclohexane and cyclohexane-d_{12} (total alkane = 5 + 5 equiv.). 1,3,5-Trimethoxybenzene as an internal standard was subsequently added. After vigorously shaking for 3 min, the crude reaction mixture was filtered through a short plug of silica for GC-FID (***see spectrum***) and HR-GCMS analysis. The yields of **1** and **1-*d*$_{10/11}$** were determined by GC-FID analysis. The H/D exchange product **1-*d*$_{10}$** was observed by EI-GCMS (Figure S27) and is indicative of reversible HAT. The ratio of **1-*d*$_{11}$** and ***1-d*$_{10}$** (81.7:18.3) was determined by EI-GCMS using the ^{11}B isotope peaks (Figure S28).

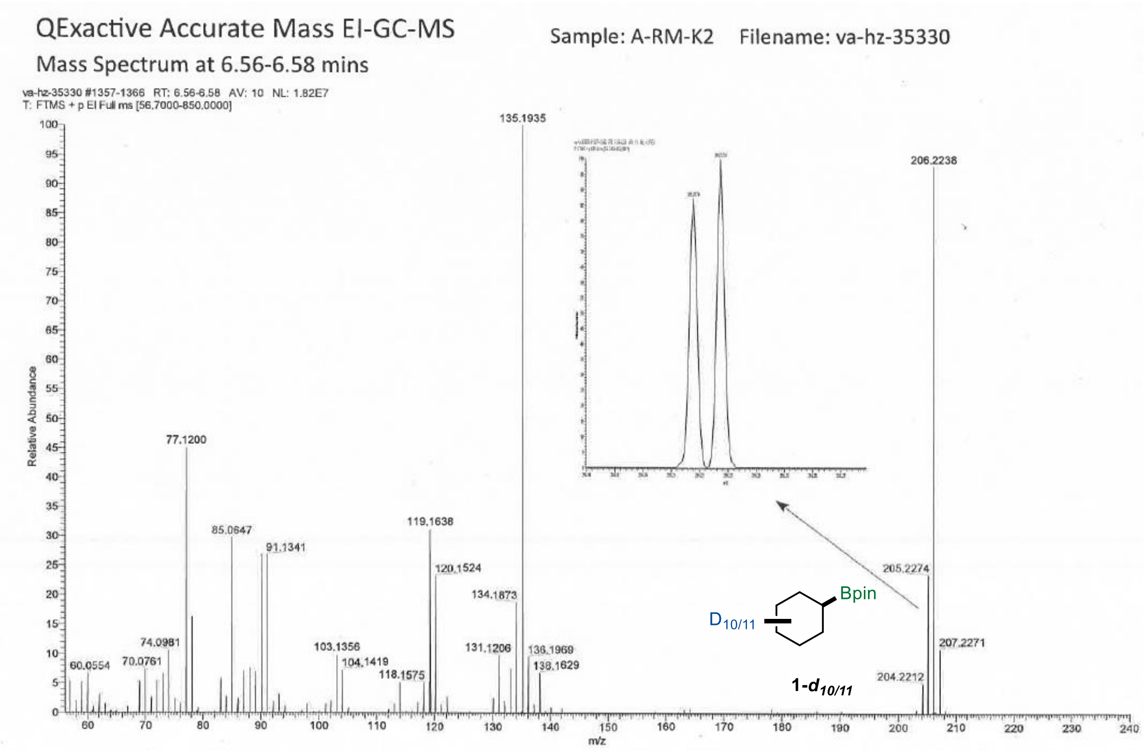

Observed m/z	Theoretical Mass	Error (ppm)	Formula	ID
206.2238	206.2241	1.45	$C_{12}H_{12}D_{11}BO_2$	$[B\text{-}CH_3]^+$
205.2178	205.2179	0.49	$C_{12}H_{13}D_{10}BO_2$	$[C\text{-}CH_3]^+$

Figure S27. HR-GCMS data for the KIE experiments with cyclohexane

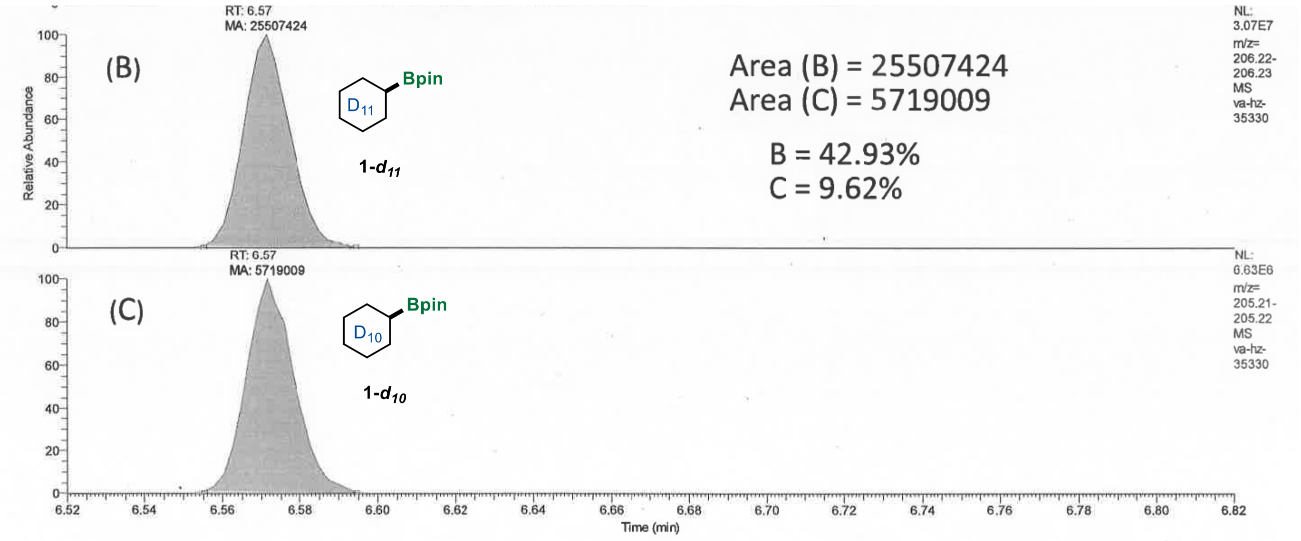

Figure S28. HR-GCMS quantification for the KIE experiments with cyclohexane

The ratio of products was determined by ultra-high-resolution GC-MS using an Orbitrap mass analyzer. The ultra-high-resolution in the mass dimension enables analysis of the data using exact mass extracted ion chromatography, which displays peak areas in the chromatogram based on their exact mass. With this method, we can separate ions in the mass dimension even when they overlap in the chromatogram.

3.9.4. Parallel KIE study for the borylation of cyclohexane

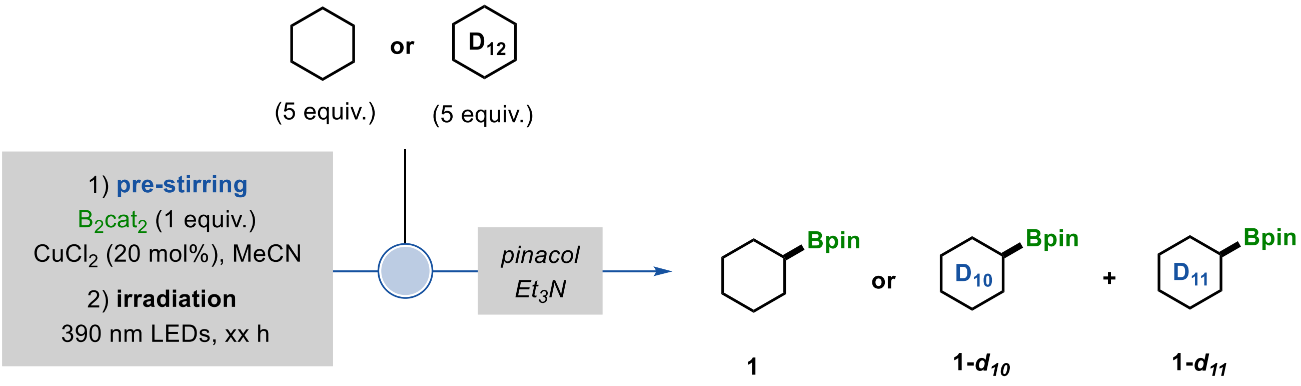

Under an ambient atmosphere, a flame dried 8 mL Biotage® microwave reaction vial equipped with a magnetic stir bar was charged with $CuCl_2$ (8 mg, 0.06 mmol, 0.2 equiv.) and B_2cat_2 (71 mg, 0.30 mmol, 1.0 equiv.). The vial was transferred into an anhydrous, argon-filled glovebox where anhydrous acetonitrile was added (1.5 mL, *c* = 0.20 M) and the vial was sealed by a Suba Seal septum. After stirring in the glovebox for 16 h, cyclohexane or cyclohexane-d_{12} (1.5 mmol, 5.0 equiv.) was added. The vial was subsequently sealed with a cap with septum, removed from the glovebox, and placed 4 cm away from two purple LEDs (Kessil PR160-390 nm LEDs). The reaction was stirred at a speed of 1200 rpm and irradiated for specific time at 40 °C. After irradiation, while maintaining an argon atmosphere, a solution of pinacol (106 mg, 0.900 mmol, 3.00 equiv.) and Et_3N (0.84 mL, 6.0 mmol, 20 equiv.) in DCM (1 mL) was added and stirring was continued for 1 h. Diethyl phthalate as an internal standard was subsequently added. After vigorously shaking for 3 min, the crude reaction mixture was filtered through a short plug of silica and the yield was determined by GC-FID analysis.

Time (h)	*GC-FID yields (%)*[a]		*KIE (1:1-$d_{10/11}$)*
	**1**	**1-d_{10}**	
2	20.7	15.6	1.33
4	25.2 (25.0)[b]	20.3	1.24
6	36.6	32.1	1.14

Table S27. Parallel KIE study for the borylation of cyclohexane

[a] The yields were determined by GC-FID analysis. The reported yields were average yields from duplicate reactions. [b] Reaction without pre-stirring.

3.10. Further Discussion of the Proposed Mechanism

3.10.1. Mechanistic proposals for O(Bcat)$_2$ formation

$$B_2(cat)_2 \xrightarrow[\text{MeCN (0.2 M), r.t., 2 h}]{\text{CuCl}_2\text{ (1.0 equiv.). (H}_2\text{O)}} O(Bcat)_2 + H_2$$

The NMR experiments in Figures S5 and S6 (Section 3.2.1) show the new boron species formed after mixing B$_2$cat$_2$ and CuCl$_2$ in MeCN at different time points. According to the NMR studies, B$_2$cat$_2$ was partially converted to HOBcat after 10 to 15 minutes. After 1 h, the HOBcat peak disappeared, and O(Bcat)$_2$ was observed. After 10 h, all the B$_2$cat$_2$ was found to be converted to O(Bcat)$_2$. H_2 gas was also detected by ^{1}H NMR (Figure S9).

Note:

1. HOBcat and O(Bcat)$_2$ were also synthesised independently. We compared the chemical shifts to confirm the formation of HOBcat and O(Bcat)$_2$ in the above reaction.
2. The chemical shift of the H_2 gas peak is consistent with the reported chemical shift in CD_3CN.[23] Addition of HBcat and H_2O led to an increase in intensity of this peak, thus confirming this peak corresponds to H_2 gas.

Based on the NMR study and independent experiment, we can fully confirm O(Bcat)$_2$ and H_2 gas formed after mixing of B$_2$cat$_2$ and CuCl$_2$ in MeCN. The water required for this reaction is likely present as a trace impurity in the MeCN solvent or commercially available B$_2$cat$_2$.

To evaluate the reactivity of O(Bcat)$_2$ in our transformation, the borylation of cyclohexane was carried out with independently synthesised O(Bcat)$_2$ and commercially available ClBcat as the borylating gents (Sections 3.3 and 3.4).

O(Bcat)$_2$ was prepared by condensation of $B(OH)_3$ and catechol in 68% yield. As the borylating reagent, it gave 10% boronic ester product. Since O(Bcat)$_2$ was found to be extremely sensitive to air and moisture, a pure sample could not be obtained for use in the borylation reaction. The use of impure O(Bcat)$_2$ is likely responsible for the low yield of borylated product (10%).

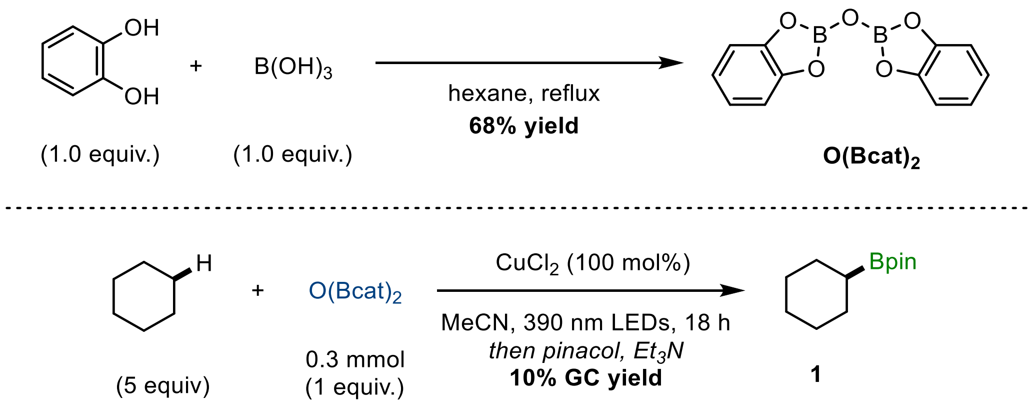

We also examined the reactivity of ClBcat. The NMR experiments in Figures S9-10 show that hydrolysis of ClBcat occurs due to the presence of water impurities in the MeCN solvent, which leads to the formation of $O(Bcat)_2$ and HCl. Since ClBcat can be converted to $O(Bcat)_2$ *in situ*, the use of ClBcat in the borylation of cyclohexane led to productive borylation, with boronic ester product **1** formed in 48% yield (Table S20). We also studied this reaction by NMR and found that after irradiation the only observable products were the boronic ester and incompletely consumed $O(Bcat)_2$ (Figures S11-13).

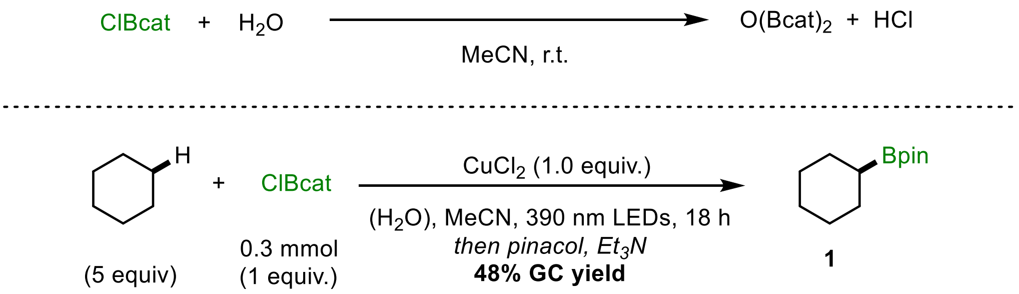

Based on these results, we can confirm that B_2cat_2 undergoes Cu-catalysed oxidative cleavage of the B–B bond in the presence of water impurities to generate $O(Bcat)_2$, and that this bis-boryloxide is the key borylating agent in the subsequent $CuCl_2$-catalysed C-H borylation reaction.

3.10.2. Mechanistic proposals for the observed regioselectivities

In this C-H borylation reaction, there are several possible radical species that could undergo HAT to generate alkyl radicals from the alkane substrate (Figure S29).

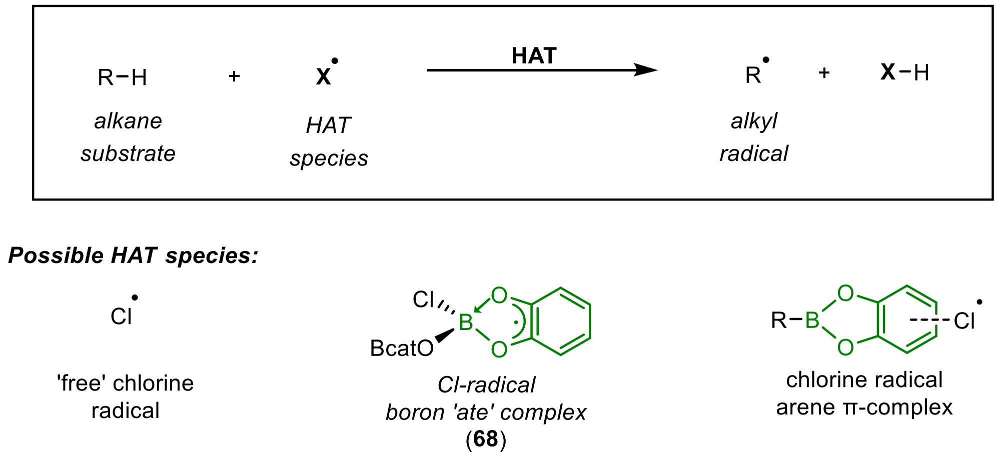

Figure S29. Possible HAT species

To provide insight into which radical species are involved, we compared the regioselectivities of the borylations of 2,3-dimethylbutane and THF with those obtained for Giese reactions with ethyl acrylate, both in isolation and in competition experiments (Section 3.7). The following sections discuss the results of these studies and the insights that they provide into the identity of the HAT species.

Competition experiment with 2,3-dimethylbutane

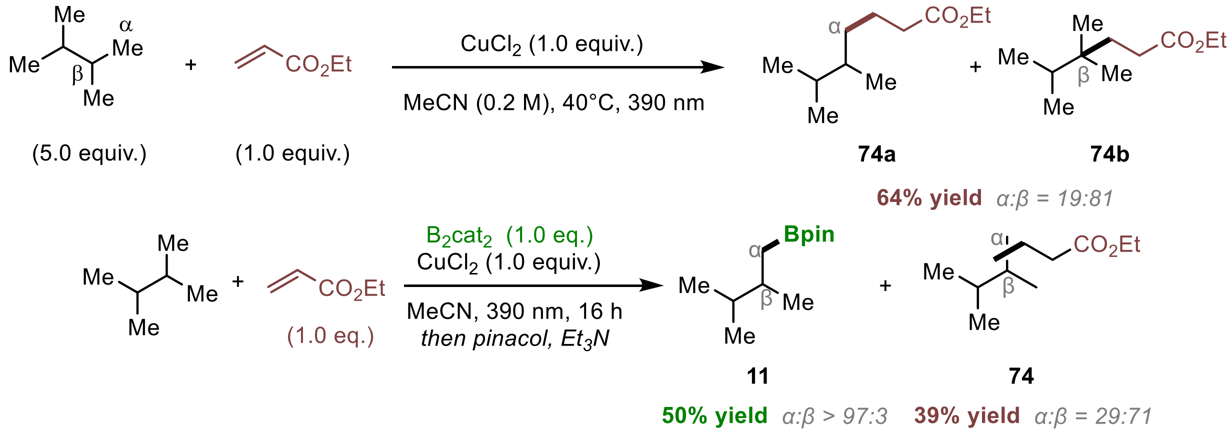

The alkylation of 2,3-dimethylbutane with ethyl acrylate gave a 19:81 regioselectivity for functionalisation of primary:tertiary positions (**74a:74b**), which is consistent with the regioselectivity of 'free' chlorine radicals in HAT reactions with 2,3-dimethylbutane.[27] The competition experiment showed a higher primary selectivity of 69:31 (Figure S30), thus the tertiary:primary selectivity changed from 26:1 to 3:1 in the presence of B_2cat_2.

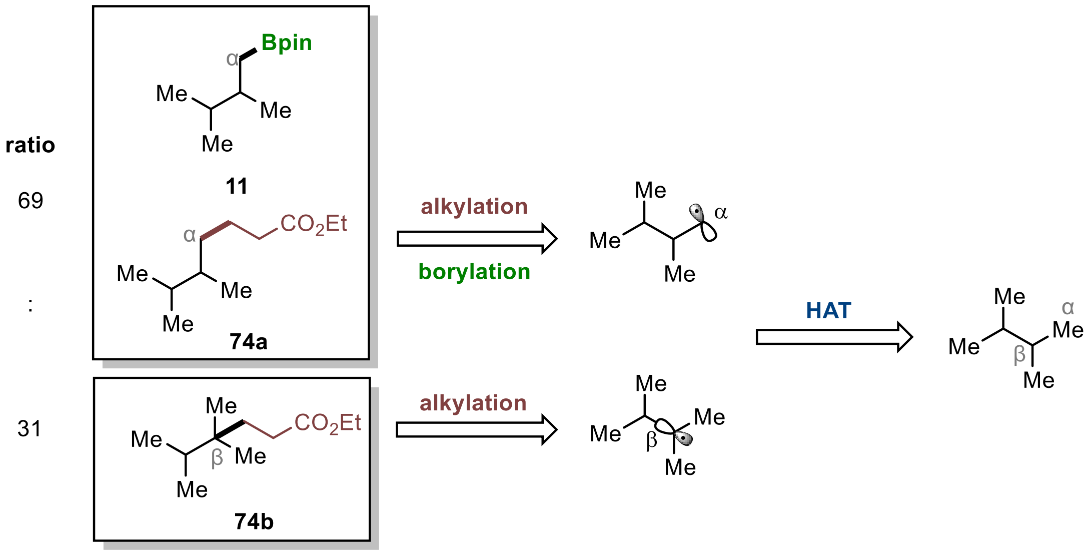

Figure S30. Proposed pathways for the borylation and alkylation of 2,3-dimethylbutane

These results provide evidence for tertiary radical formation during the borylation reaction. The increased primary selectivity indicates that a HAT species is formed that prefers C–H abstraction from primary positions. Based on our previously reported metal-free C–H borylation method, which shows similar primary C–H selectivity, a chlorine radical–boron 'ate' complex, is considered as a HAT reagent.[3] Therefore, we propose that chlorine radicals formed via LMCT react with $O(Bcat)_2$ to afford a similar chlorine radical–boron 'ate' complex, which serves as the HAT species.

Time study for the competition experiment with 2,3-dimethylbutane

The results of the time study of the competition reaction with 2,3-dimethylbutane (Table S26) show that the primary and tertiary alkylated products were generated simultaneously with the borylated product, which means primary and tertiary radicals were generated simultaneously. The ratio of products derived from primary and tertiary radicals remains approximately constant (1°:3° = 2:1) over the course of the reaction, which suggests only one single HAT species is involved in the C–H abstraction.

In addition, the ratio of primary and tertiary alkylated products (1°:3° = 1:10) and the ratio of primary alkylated product to boronic ester (**74a:11** = 1:20) both remain approximately constant during the first 8 h of the reaction. This suggests that the primary radical is trapped by borylating reagent approximately 20 times faster than ethyl acrylate. For primary radicals, the borylation is much faster than alkylation, whereas, for tertiary radicals, the borylation is much slower than alkylation.

After pre-stirring for 8 h, the yields of **74** and **11** are all doubled, while the ratio of 1° to 3° products remains unchanged at 2:1. Thus, pre-stirring improves the efficiency of both the borylation and alkylation processes but the regioselectivity is unaltered, suggesting that the chlorine radical–boron 'ate' complex is the HAT species both with and without the pre-stirring protocol.

'Free' chlorine radicals

The reactivity of 'free' chlorine radicals in HAT processes has been extensively studies. The regioselectivity for HAT from 2,3-dimethylbutane was reported by Russel to be 81:19 (approximately 4:1, 3° to 1°) in favour of tertiary C–H bonds for photochlorinations with Cl_2 in neat 2,3-dimethylbutane at 25 °C,[29] which is constant with the regioselectivity we obtained in the alkylation reaction with ethyl acrylate in absence of B_2cat_2. However, as described above, not only did we observe complete primary selectivity in the borylation reaction, but we also observed increased primary selectivity in the competition experiment with ethyl acrylate. Therefore, it is unlikely that the 'free' chlorine radicals generated via LMCT of $CuCl_2$ are the predominant HAT species in our transformation. However, the observation of decreased regioselectivity with increasing $CuCl_2$ loading (Sections 3.1.2-3.1.4) suggests that HAT to 'free' chlorine radicals becomes competitive at higher $CuCl_2$ loadings.

Chlorine radical complexes

The regioselectivities in this borylation of alkanes follow the reactivity trend of methylene > methine, which is the opposite reactivity to that of 'free' chlorine radicals. We observed similar primary C–H selectivity in our previously reported metal-free C–H borylation method, which was attributed to a chlorine radical–boron 'ate' complex as the predominant HAT species. Therefore, by analogy, we propose the formation of a related complex between a chlorine radical and $O(Bcat)_2$ in this work (Figure S31). The chlorine radicals generated by LMCT of $CuCl_2$ can reacts with $O(Bcat)_2$ to chlorine radical–boron 'ate' complex **68**, where the unpaired electron is delocalised onto the catecholate ligand.[30] We believe that the radical 'ate' complex is the predominant HAT species involved in our borylation reaction, where the increased steric hindrance around the chlorine atom results in regioselective abstraction of the least sterically hindered hydrogen atoms.

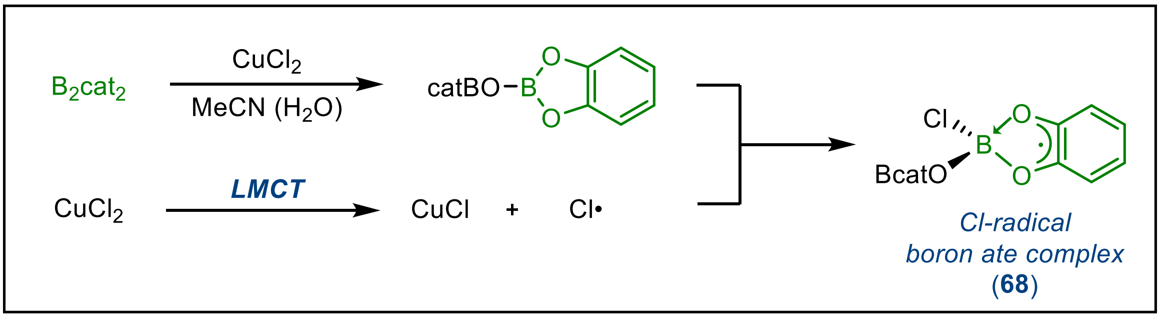

Figure S31. **Proposed pathway for formation of the HAT species**

Regioselectivity in the borylation step

In the competition experiment with 2,3-dimethylbutane, the chlorine radical–boron ‘ate’ complex complex **63** serves as the predominant HAT species (Figure S32). Both 1° radicals and 3° radicals are generated, but more 1° radical is formed due to the steric influence of **68**. There are three pathways subsequent reaction of the two alkyl radicals: 1) borylation; 2) alkylation; and 3) single-electron oxidation. According to the ratio of product derived from the primary alkyl radical in the competition experiment (**11**>**74a**), for less sterically hindered primary radicals, the relative rate of the three possible reaction pathways is: k_5 (borylation) > k_1 (alkylation) > k_4 (oxidation). As no tertiary boronic ester product was observed but tertiary alkylated product was, for more sterically hindered tertiary radicals, the relative rate of the three possible reaction pathways is: k_2 (alkylation) > k_3 (oxidation) > k_6 (borylation). The low rate of borylation of tertiary alkyl radicals leads to enhanced primary selectivity in the borylation reactions due to competing single-electron oxidation to tertiary carbocations, whereas, in the presence of ethyl acrylate alkylation outcompetes single-electron oxidation.

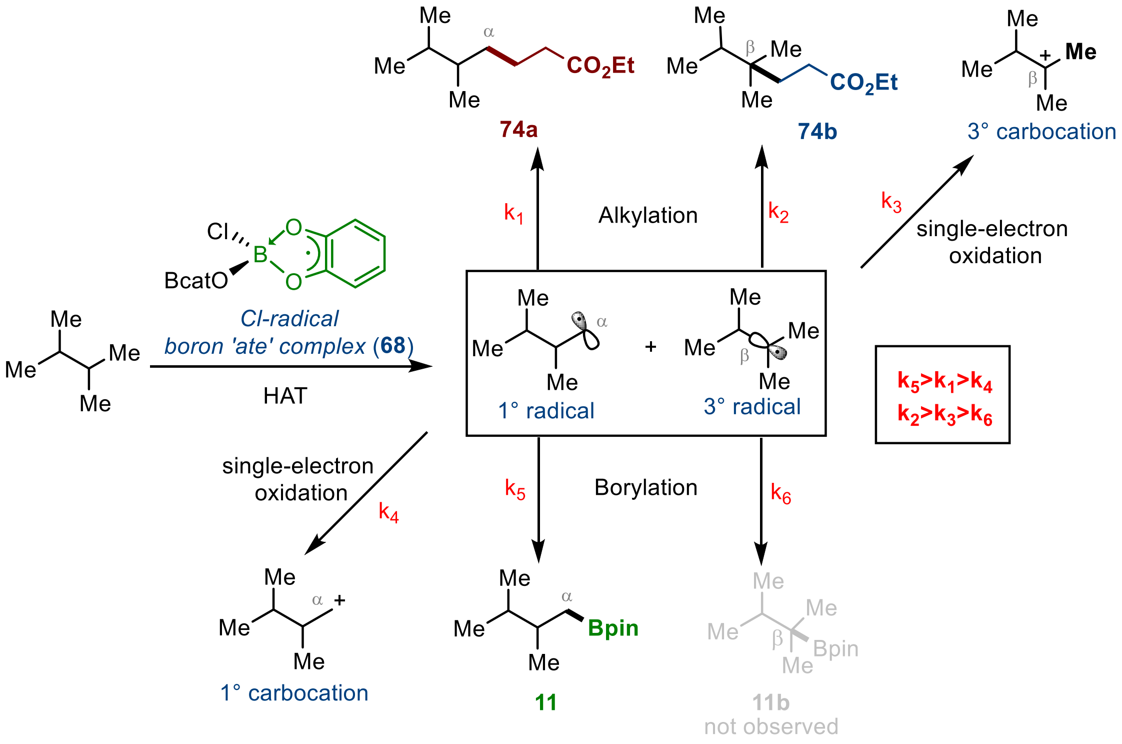

Figure S32. **Competing pathways for the reaction of primary and tertiary radicals**

Based on the results above, we conclude that the regioselectivity of the borylation reactions is determined by both the HAT and borylation steps:

1. HAT process: The steric influence of chlorine radical–boron ‘ate’ complex complex **68** results in HAT at less sterically hindered positions, which leads to good selectivity for the formation of primary alkyl radicals. However, secondary and tertiary alkyl radicals are also generated.
2. Borylation process: The borylation step is strongly influenced by steric hindrance, which results in a slow rate of borylation of tertiary alkyl radicals compared to those of secondary and primary alkyl radicals. This allows single-electron oxidation of tertiary alkyl radicals to outcome borylation, leading to the formation of alkene and chlorinated side-products. For α-oxy radicals, such as those formed in the borylation of THF, the high rate of single-electron oxidation to a stabilised oxocarbenium ion also prevents borylation.

3.10.3. Mechanistic proposals for Cu(II) consumption

To gain insight into the Cu(II) consumption, several EPR experiments were performed. In our transformation, Cu(II) was supposed to be reduced to Cu(I) by three pathways: (a) photoreduction of Cu(II)→Cu(I) via photoinduced LMCT to generate chlorine radicals; (b) reduction of Cu(II)→Cu(I) during the single-electron oxidation of alkyl radicals to carbocations; and (c) reduction of Cu(II) by B_2cat_2 (Figure S33).

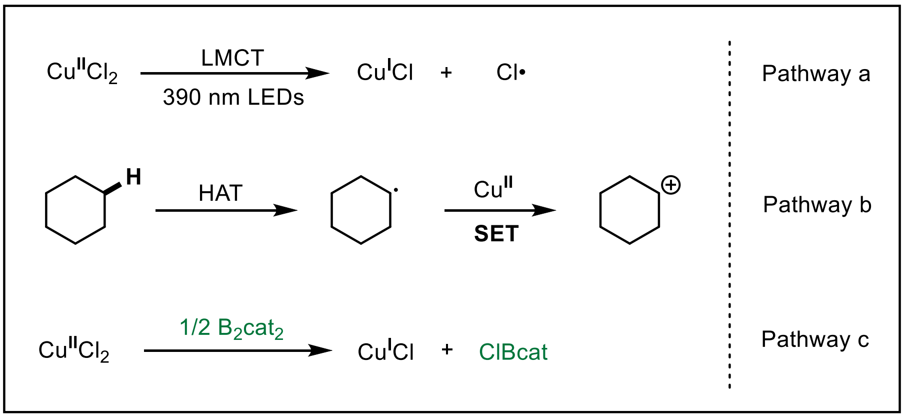

Figure S33. Possible pathways for the consumption of $CuCl_2$

In section 3.8, our EPR results show that irradiation resulted in almost complete loss of Cu(II) signals due to reduction to Cu(I). This was observed after irradiating a solution of $CuCl_2$ in MeCN in the absence of alkane or B_2cat_2 (Figure S21, b), which suggests that photoreduction of Cu(II)→Cu(I) occurs through a photoinduced LMCT process (Pathway a, Figure S33). No Cu(II) signals were observed after irradiating a solution containing $CuCl_2$, cyclohexane and B_2cat_2 (Figure S21, f), which indicated the $CuCl_2$ was fully consumed after 18 h under the standard borylation conditions. Therefore, this explains why the borylation reaction stops proceeding after 18 h irradiation, since the complete consumption of $CuCl_2$ prevents LMCT to generate chlorine radicals for subsequent HAT reactions. As a result, $O(Bcat)_2$ could not be fully converted due to interruption of the HAT processes. The single-electron oxidation of secondary and tertiary alkyl radical intermediates to produce carbocations likely contributes to the reduction of Cu(II) to Cu(I) during the borylation reaction (Pathway b,

Figure S33). Conversely, no decrease in the intensity of the Cu(II) signals was observed after stirring $CuCl_2$ and B_2cat_2 in the dark (Figure S21, d), which indicated that $O(Bcat)_2$ formation did not consume Cu(II), therefore the reduction of Cu(II) by B_2cat_2 could be excluded (Pathway c, Figure S33).

To restart the reaction and promote further conversion of $O(Bcat)_2$, we evaluated the effects of adding extra $CuCl_2$ after the initial 24 h irradiation (Section 3.6). Control experiments showed that extension of irradiation time did not enhance the conversion of $O(Bcat)_2$, but the addition of an extra portion of $CuCl_2$ led to a dramatic increase in yield of the boronic ester product. This provides evidence that the incomplete conversion of $O(Bcat)_2$ is a result of the full consumption of $CuCl_2$.

3.10.4. Mechanistic proposals for byproduct formation

$$[Cl–Cu^{II}–OBcat] + HCl \longrightarrow HOBcat + CuCl_2$$

not observed

$$HOBcat \xrightarrow{HCl,\ [Cu]} 1/2\ O(Bcat)_2 + 1/2\ H_2O$$

observed

Based on our proposed mechanism, we expected the byproduct of the borylation reaction with $O(Bcat)_2$ to be HOBcat, resulting from a ligand exchange between HCl and Cl-Cu-OBcat. However, this was not observed upon analysis of the reaction mixtures by NMR. Instead, incomplete conversion of $O(Bcat)_2$ was observed, where the spectroscopic data matched those of the independently synthesised $O(Bcat)_2$. We reason that condensation of two molecules of HOBcat occurs rapidly in the presence of HCl and $CuCl_2$ as dehydrating agents, thus leading to the regeneration of $O(Bcat)_2$. This was confirmed upon addition of HOBcat to the crude reaction mixture, which was also fully converted to $O(Bcat)_2$. Given that H_2O is consumed during the oxidation of B_2cat_2 to form $O(Bcat)_2$ and is regenerated during the condensation of HOBcat, where we believe H_2O acts as a catalyst to promote the overall transformation.

4. DFT CALCULATIONS

4.1. General Notes

Geometry optimizations were run using PBE0[31-32] with the def2-TZVP[33] basis set and D3 dispersion correction[34] in the gas phase using Gaussian 16,[35] a level of theory used to investigate similar systems.[36] The resulting structures were confirmed as minima using frequency calculations, and transition states were confirmed to connect the expected minima with intrinsic reaction coordinate calculation.[37-39]

4.2. Borylation with B_2cat_2

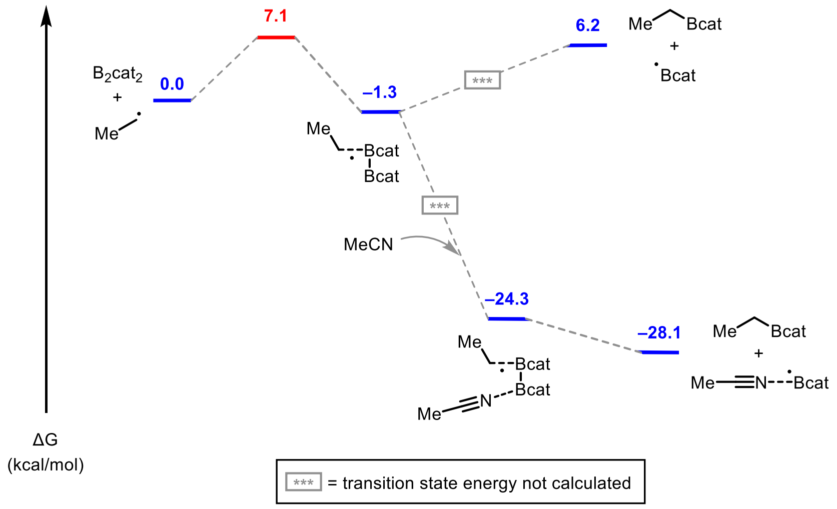

Figure S34. Calculated free energies for the borylation of an ethyl radical with B_2cat_2

Structure	Name	Energy (Hartrees)
Ethyl radical	Et_rad	-79.047940
B_2cat_2	catBBcat	-812.120160
Transition state for ethyl radical addition to B_2cat_2	CatBBCat_Et_addition_rad_TSS	-891.156858
B_2cat_2 ethyl radical adduct	CatBBCat_Et_rad	-891.170124
Acetonitrile	ACN	-132.619581
B_2cat_2 ethyl radical adduct acetonitrile complex	CatBBCat_Et_rad_ACN	-1023.826436
Bcat radical	CatB_rad	-405.981482
Bcat radical acetonitrile complex	CatB_rad_ACN	-538.655630
EtBcat	CatB_Et	-485.176783

4.3. Borylation with OB(cat)$_2$

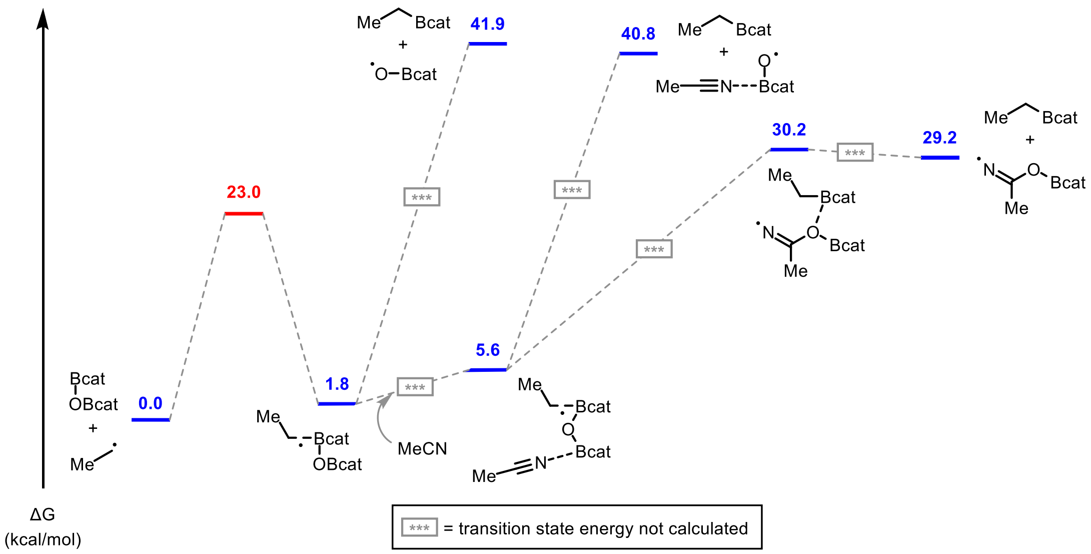

Figure S35. Calculated free energies for the borylation of an ethyl radical with O(Bcat)$_2$

**Structure**	**Name**	**Energy (Hartrees)**
Ethyl radical	Et_rad	-79.047940
O(Bcat)$_2$	CatBOBCat	-887.403200
Transition state for ethyl radical addition to O(Bcat)$_2$	CatBBCat_Et_addition_rad_TSS	-966.414536
O(Bcat)$_2$ ethyl radical adduct	CatBOBCat_Et_rad	-966.448221
Acetonitrile	ACN	-132.619581
O(Bcat)$_2$ ethyl radical adduct acetonitrile complex	CatBOBCat_Et_rad_ACN_complex	-1099.061865
O(Bcat)$_2$ ethyl radical adduct with O–C bond formed to acetonitrile	CatBOBCat_Et_rad_ACN_bound	-1099.022656
OBcat radical	CatBO_rad	-481.207518
OBcat radical acetonitrile complex	CatBO_rad_ACN_complex	-613.828954
OBcat radical with O–C bond formed to acetonitrile	CatBO_rad_ACN_bound	-613.847404
EtBcat	CatB_Et	-485.176783

4.4. Borylation with OB(cat)$_2$ in the Presence of CuCl

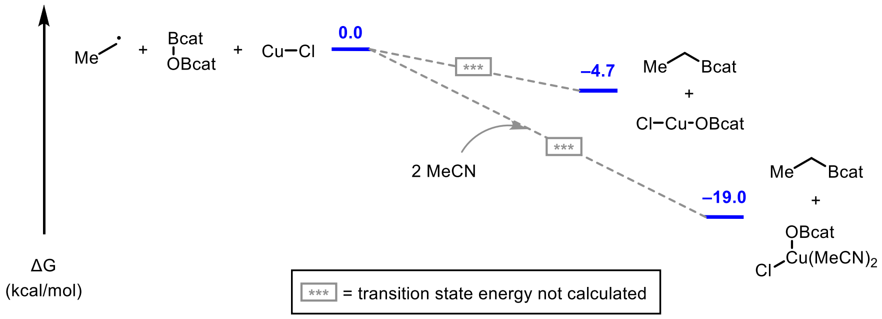

Figure S36. Calculated free energies for the borylation of an ethyl radical with O(Bcat)$_2$ and CuCl

Structure	Name	Energy (Hartrees)
Ethyl radical	Et_rad	-79.047940
O(Bcat)$_2$	CatBOBCat	-887.403200
Copper(I) chloride	CuCl	-2100.395943
Acetonitrile	ACN	-132.619581
EtBcat	CatB_Et	-485.176783
Cl–Cu–OBcat	CatBO_rad_CuCl	-2581.677804
Cl–Cu–OBcat acetonitrile complex	CatBO_rad_CuCl_2ACN	-2846.939678

4.5. Discussion of Computational Results

Radical addition to B_2cat_2 vs. $O(Bcat)_2$

The calculation results show that the thermodynamics for the addition of an ethyl radical to B_2cat_2 and $O(Bcat)_2$ do not differ significantly, with addition to B_2cat_2 slightly exergonic (ΔG = –1.3 kcal/mol) and addition to $O(Bcat)_2$ slightly endergonic (ΔG = +1.8 kcal/mol). However, the kinetics of the two radical additions differ significantly, with the large energy barrier calculated for the formation of the $O(Bcat)_2$ ethyl radical adduct ($\Delta G^\ddagger$ = +23.0 kcal/mol) indicating that this process is kinetically disfavoured relative to the formation of the B_2cat_2 ethyl radical adduct ($\Delta G^\ddagger$ = +7.1 kcal/mol).

Ethyl boronic ester formation

Investigation of the thermodynamics for the transformation of the B_2cat_2 ethyl radical adduct to the ethyl boronic ester product and a Bcat radical show that B–B bond cleavage becomes thermodynamically favoured upon complexation with a molecule of acetonitrile (ΔG = –26.8 kcal/mol). This is in agreement with previous calculations by Studer and co-workers, who showed that DMF also promoted B–B bond cleavage.[36] Conversely, transformation of the $O(Bcat)_2$ ethyl radical adduct to the ethyl boronic ester product and an OBcat radical was found to be strongly thermodynamically disfavoured (ΔG = –26.8 kcal/mol). This suggest that borylation is unlikely to occur by direct homolytic substitution of $O(Bcat)_2$.

Borylation with $O(Bcat)_2$ in the presence of CuCl

Given that direct homolytic substitution of $O(Bcat)_2$ by alkyl radicals was calculated to be a highly endergonic process, we postulated that copper must play a crucial role in C–B bond formation. We calculated the thermodynamics of the reaction of an ethyl radical with $O(Bcat)_2$ and copper(I) chloride and found this to be exergonic, both with and without ligation of acetonitrile to copper (–19.0 and –4.7 kcal/mol, respectively). Based on the above results, we suggest that copper(I)-ligated $O(Bcat)_2$ is likely the active borylating agent.

4.6. XYZ Coordinates

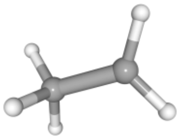

Et_rad Energy: -79.047940 Hartrees

7

C	-0.00987	0.78974	-0.00000
C	-0.00987	-0.68940	0.00000
H	0.05806	1.34573	0.92628
H	0.05806	1.34573	-0.92628
H	-0.50496	-1.09705	-0.88556
H	1.01219	-1.09946	0.00000
H	-0.50496	-1.09705	0.88556

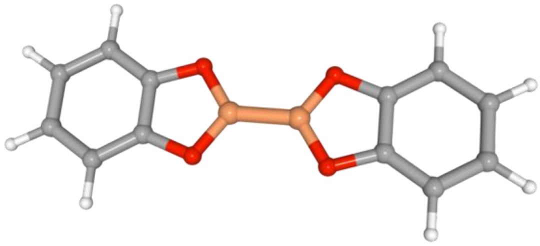

CatBBCat Energy: -812.120160 Hartrees

26

C	2.92224	0.69364	-0.00006
C	2.92225	-0.69365	-0.00001
C	4.08913	-1.42435	0.00013
C	5.27589	-0.69604	0.00022
C	5.27587	0.69609	0.00017
C	4.08909	1.42437	0.00003
H	4.07657	-2.50683	-0.00012
H	6.22075	-1.22625	0.00042
H	6.22072	1.22632	0.00033
H	4.07651	2.50685	-0.00030
O	1.63072	1.13929	-0.00020
O	1.63075	-1.13933	-0.00013
B	0.84330	-0.00003	-0.00010
B	-0.84330	-0.00004	-0.00010
O	-1.63075	-1.13934	-0.00020
O	-1.63071	1.13929	-0.00013
C	-2.92226	-0.69365	-0.00006
C	-2.92224	0.69364	-0.00001
C	-4.08913	-1.42435	0.00003
C	-4.08909	1.42437	0.00013
C	-5.27589	-0.69604	0.00017
H	-4.07657	-2.50683	-0.00030
C	-5.27587	0.69610	0.00022
H	-4.07650	2.50686	-0.00012
H	-6.22076	-1.22624	0.00033
H	-6.22072	1.22633	0.00042

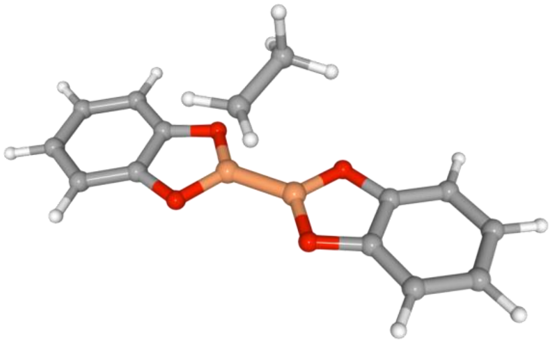

CatBBCat_Et_addition_rad_TSS Energy: -891.156858 Hartrees

33

O	-1.65182	-0.81070	-0.75247
B	-0.85793	0.20238	-0.22755
O	-1.65220	1.23072	0.26788
B	0.81907	0.16334	-0.15050
O	1.62011	-0.85377	-0.68637
O	1.61969	1.24551	0.23558
C	-2.94151	-0.40341	-0.56754
C	-2.94137	0.83838	0.05295
C	-4.10864	-1.04960	-0.90656
C	-5.29685	-0.39265	-0.59378
C	-4.10898	1.49727	0.36512
C	-5.29686	0.85215	0.02758
H	-4.09619	-2.01720	-1.39215
H	-6.24120	-0.86253	-0.84150
H	-4.09710	2.46659	0.84721
H	-6.24133	1.33234	0.25422
C	2.90160	0.84949	0.01683
C	4.07099	1.53169	0.27279
C	2.90216	-0.42417	-0.54234
C	4.07158	-1.07405	-0.87125
C	5.25832	0.88205	-0.05558
C	5.25868	-0.39225	-0.61511
H	4.05860	2.52355	0.70674
H	4.05979	-2.06451	-1.30847
H	6.20221	1.38109	0.12900
H	6.20278	-0.86540	-0.85768
C	0.37857	-0.80844	1.96372
H	-0.27253	-0.07000	2.41943
C	-0.09327	-2.20205	1.79238
H	-1.14540	-2.25059	1.50792
H	0.01667	-2.75842	2.73459
H	0.49354	-2.72997	1.03751
H	1.44172	-0.63412	2.10128

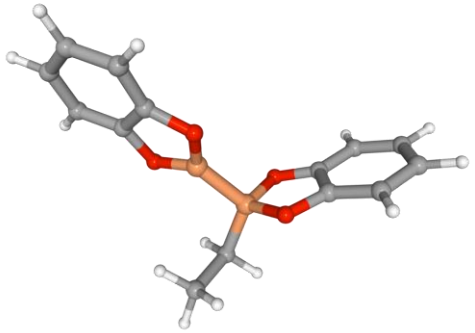

CatBBCat_Et_rad Energy: -891.170124 Hartrees

33

C	-2.61569	-0.17440	0.60503
C	-2.57859	-0.22655	-0.81496
C	-3.51660	-0.96504	-1.53553
C	-4.47663	-1.63428	-0.80798
C	-4.51377	-1.58229	0.60120
C	-3.59247	-0.85931	1.32720
H	-3.47873	-0.99965	-2.61671
H	-5.22603	-2.21948	-1.32776
H	-5.29040	-2.12954	1.12235
H	-3.61084	-0.81515	2.40851
O	-1.64815	0.57072	1.06332
O	-1.58626	0.48551	-1.27586
B	-0.85004	1.07439	-0.10264
B	0.75820	0.42268	-0.05648
O	1.17966	-0.65608	0.71008
O	1.81673	0.92649	-0.80120
C	2.50775	-0.81601	0.42949
C	2.89497	0.14677	-0.49127
C	3.39183	-1.74523	0.92820
C	4.18590	0.22914	-0.96089
C	4.70234	-1.67226	0.45973
H	3.07870	-2.49165	1.64728
C	5.09041	-0.70721	-0.46364
H	4.47682	0.98496	-1.67945
H	5.43398	-2.38368	0.82397
H	6.11858	-0.68126	-0.80466
C	-0.82942	2.69314	-0.16265
C	-0.15366	3.33353	1.03955
H	-1.86990	3.03209	-0.24506
H	-0.32324	2.98242	-1.08860
H	0.90056	3.04832	1.10180
H	-0.19253	4.42523	0.98836
H	-0.63190	3.02897	1.97388

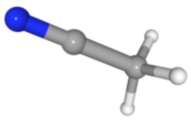

ACN Energy: -132.619581 Hartrees

6

C	-0.00000	0.00000	0.27865
N	-0.00000	0.00000	1.42768
C	0.00000	-0.00000	-1.17121
H	0.00000	1.02475	-1.54613
H	0.88746	-0.51238	-1.54613
H	-0.88746	-0.51238	-1.54613

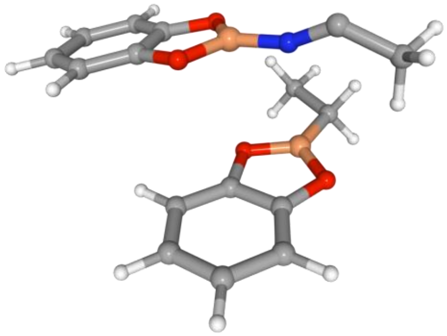

CatBBCat_Et_rad_ACN Energy: -1023.826436 Hartrees

39

O	1.44381	1.70145	-0.48557
B	0.56822	0.96091	-1.27447
O	1.06947	-0.30683	-1.54723
B	-1.72071	0.80464	1.28607
O	-2.78830	0.11217	0.72232
O	-0.61792	-0.02863	1.41946
C	2.48224	0.85054	-0.23213
C	2.25612	-0.36112	-0.87489
C	3.60513	1.07052	0.52972
C	4.51098	0.01429	0.62883
C	3.14242	-1.40858	-0.78513
C	4.28522	-1.19683	-0.01347
H	3.76934	2.01726	1.02859
H	5.40908	0.14331	1.22100
H	2.95328	-2.34750	-1.29010
H	5.01101	-1.99522	0.08562
C	-1.00705	-1.24012	0.91792
C	-0.27623	-2.39909	0.80568
C	-2.32367	-1.15294	0.49037
C	-2.97750	-2.22447	-0.07254
C	-0.92808	-3.49158	0.23711
C	-2.24792	-3.40639	-0.19198
H	0.75528	-2.44632	1.13146
H	-4.00552	-2.14808	-0.40380
H	-0.39227	-4.42662	0.12483
H	-2.72152	-4.27677	-0.63035
C	-1.77345	2.29360	1.73087
H	-2.06953	2.89313	0.86117
C	-0.47573	2.82764	2.32795
H	0.34051	2.76387	1.60629
H	-0.17870	2.25000	3.20619
N	-0.70563	1.39303	-1.65255
C	-1.54663	1.88348	-2.37893
C	-3.01010	2.04219	-2.23742
H	-3.38212	1.57082	-1.32191
H	-3.25302	3.10757	-2.23761
H	-3.50434	1.60730	-3.10874
H	-0.57745	3.87136	2.63324
H	-2.60314	2.40482	2.44033

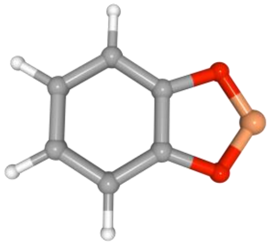

CatB_rad Energy: -405.981482 Hartrees

13			
C	0.00000	0.69299	0.32707
C	-0.00000	-0.69299	0.32707
C	0.00000	-1.42444	-0.83722
C	0.00000	-0.69531	-2.02441
C	0.00000	0.69531	-2.02441
C	0.00000	1.42444	-0.83722
H	0.00000	-2.50661	-0.82397
H	0.00000	-1.22531	-2.96912
H	0.00000	1.22531	-2.96912
H	0.00000	2.50661	-0.82397
O	0.00000	1.14363	1.62897
O	-0.00000	-1.14363	1.62897
B	-0.00000	-0.00000	2.38749

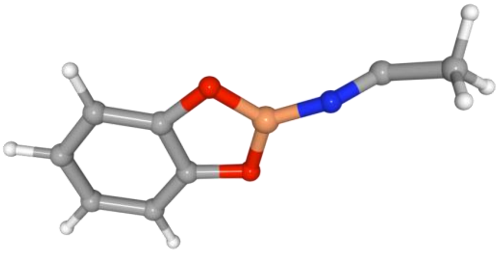

CatB_rad_ACN Energy: -538.655630 Hartrees

19			
B	1.03443	0.00061	-0.02066
O	0.25265	1.15141	-0.01068
O	0.25322	-1.15059	-0.01062
C	-1.03556	0.69504	0.00138
C	-2.20277	1.42188	0.01082
C	-1.03526	-0.69481	0.00145
C	-2.20209	-1.42225	0.01083
C	-3.39261	0.69418	0.02105
C	-3.39229	-0.69515	0.02102
H	-2.19068	2.50446	0.01018
H	-2.18938	-2.50482	0.01009
H	-4.33644	1.22622	0.02891
H	-4.33587	-1.22762	0.02882
N	2.43110	0.00085	0.03698
C	3.55094	-0.00052	-0.43262
C	4.89142	-0.00050	0.19135
H	4.83276	0.00007	1.28510
H	5.44603	-0.87753	-0.14969
H	5.44607	0.87633	-0.15027

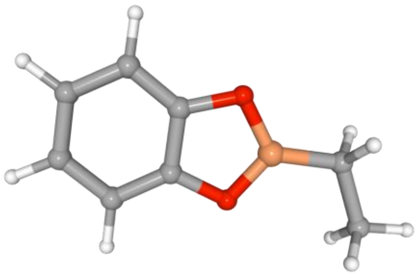

CatB_Et Energy: -485.176783 Hartrees

20

C	0.54339	0.61853	0.00011
C	0.73749	-0.75568	-0.00005
C	1.99510	-1.31332	-0.00019
C	3.07025	-0.42599	-0.00017
C	2.87544	0.95061	-0.00002
C	1.59594	1.50399	0.00013
H	2.13543	-2.38686	-0.00030
H	4.07966	-0.81979	-0.00027
H	3.73572	1.60929	-0.00002
H	1.43200	2.57419	0.00025
O	-0.79863	0.88035	0.00025
O	-0.47815	-1.37964	-0.00001
B	-1.42159	-0.35983	0.00019
C	-2.96093	-0.58288	0.00044
C	-3.79324	0.69474	-0.00057
H	-3.21117	-1.20606	0.86792
H	-3.21122	-1.20752	-0.86600
H	-3.57803	1.30673	-0.87925
H	-4.86300	0.47524	-0.00031
H	-3.57788	1.30822	0.87702

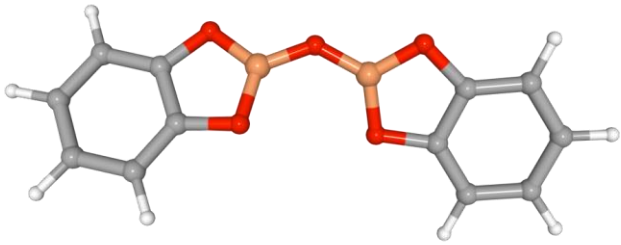

CatBOBCat Energy: -887.403200 Hartrees

27

C	-3.39573	-0.65142	0.16931
C	-2.88831	0.58184	-0.21875
C	-3.70014	1.66941	-0.43458
C	-5.06755	1.47252	-0.24411
C	-5.57568	0.23880	0.14401
C	-4.74118	-0.85740	0.36012
H	-3.29224	2.62565	-0.73647
H	-5.74682	2.30151	-0.40258
H	-6.64400	0.12346	0.28236
H	-5.12569	-1.82313	0.66249
O	-2.36503	-1.54253	0.31014
O	-1.52505	0.49502	-0.33159
B	-1.23104	-0.81617	-0.00116
O	-0.00000	-1.37227	0.00002
B	1.23103	-0.81617	0.00118
O	2.36503	-1.54253	-0.31014
O	1.52505	0.49502	0.33161

C	3.39573	-0.65142	-0.16931
C	2.88831	0.58184	0.21876
C	4.74117	-0.85740	-0.36014
C	3.70015	1.66941	0.43458
C	5.57567	0.23879	-0.14403
H	5.12568	-1.82313	-0.66253
C	5.06755	1.47252	0.24410
H	3.29225	2.62564	0.73648
H	6.64400	0.12346	-0.28240
H	5.74683	2.30151	0.40257

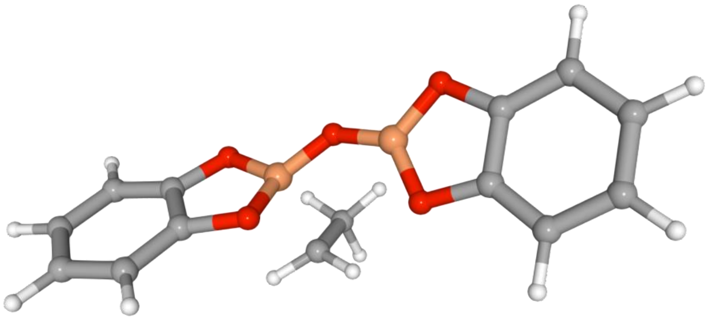

CatBOBCat_Et_addition_rad_TSS Energy: -966.414536 Hartrees

34

C	3.30499	0.29184	-0.48743
C	2.79253	-0.91594	-0.01266
C	3.61273	-1.97933	0.29728
C	4.97961	-1.79566	0.10591
C	5.49093	-0.59126	-0.36746
C	4.65782	0.48235	-0.67062
H	3.20483	-2.91247	0.66516
H	5.65888	-2.60919	0.33120
H	6.56054	-0.48537	-0.50424
H	5.04595	1.42305	-1.04074
O	2.29353	1.16389	-0.71231
O	1.44282	-0.83833	0.07606
B	1.12642	0.50090	-0.26194
O	-0.09346	0.86209	-0.79506
B	-1.33814	0.43334	-0.54418
O	-1.73324	-0.34456	0.53922
O	-2.41601	0.76301	-1.35306
C	-3.08491	-0.50065	0.38470
C	-3.49578	0.17006	-0.76033
C	-3.96699	-1.19015	1.18188
C	-4.81056	0.18250	-1.16193
C	-5.30374	-1.18385	0.78405
H	-3.63514	-1.71160	2.07087
C	-5.71542	-0.51325	-0.36068
H	-5.12109	0.70741	-2.05650
H	-6.03479	-1.71498	1.38179
H	-6.76219	-0.53043	-0.63967
C	1.03295	1.30621	1.67319
C	0.79768	2.76757	1.57362
H	2.02146	0.97468	1.98209
H	0.21656	0.67859	2.02106
H	-0.14811	3.00003	1.08050
H	0.74766	3.20340	2.58221
H	1.60388	3.27411	1.04088

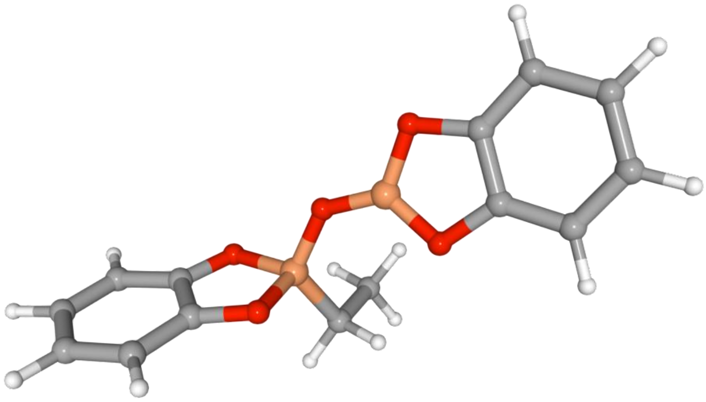

CatBOBCat_Et_rad Energy: -966.448221 Hartrees

34

C	3.29215	0.15445	-0.53995
C	2.92193	-0.65087	0.59645
C	3.84971	-1.50800	1.20284
C	5.11056	-1.54398	0.66384
C	5.47612	-0.74866	-0.45572
C	4.59022	0.10290	-1.06512
H	3.56288	-2.10940	2.05568
H	5.85844	-2.19622	1.09930
H	6.48918	-0.82398	-0.83303
H	4.85960	0.71169	-1.91839
O	2.30352	0.86787	-0.94588
O	1.69524	-0.45241	0.92544
B	1.10232	0.64207	0.00037
O	0.05677	0.08673	-0.78156
B	-1.23310	-0.06277	-0.50074
O	-2.15202	-0.56957	-1.42081
O	-1.86229	0.24272	0.71061
C	-3.34908	-0.56971	-0.76799
C	-3.17576	-0.07817	0.52124
C	-4.58015	-0.97158	-1.23022
C	-4.22347	0.03399	1.40393
C	-5.65131	-0.86248	-0.34261
H	-4.70441	-1.35247	-2.23615
C	-5.47738	-0.37120	0.94466
H	-4.07541	0.41886	2.40516
H	-6.63903	-1.16795	-0.66709
H	-6.33135	-0.29960	1.60778
C	0.81923	1.96689	0.85129
C	0.25269	3.10195	0.00739
H	1.74672	2.28631	1.34053
H	0.12001	1.70621	1.65208
H	-0.69885	2.81922	-0.45073
H	0.06970	3.99819	0.60631
H	0.93405	3.37650	-0.80216

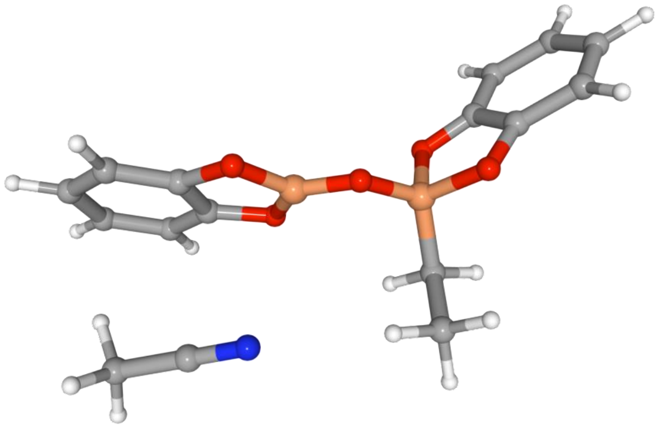

CatBOBCat_Et_rad_ACN_complex Energy: -1099.061865 Hartrees

40

C	3.75390	0.27226	-0.48349
C	3.34053	-0.75903	0.43359
C	4.23707	-1.75286	0.84842
C	5.51007	-1.69725	0.33998
C	5.91798	-0.67926	-0.56311
C	5.06335	0.30899	-0.98114
H	3.91799	-2.52372	1.53786
H	6.23431	-2.44882	0.63159
H	6.93836	-0.69166	-0.92789
H	5.36488	1.08838	-1.66884
O	2.78988	1.08265	-0.73791
O	2.11023	-0.61078	0.77462
B	1.56545	0.68697	0.11474
O	0.51571	0.35953	-0.78373
B	-0.68785	-0.12875	-0.52092
O	-1.59915	-0.49566	-1.51845
O	-1.20556	-0.43060	0.74779
C	-2.67118	-0.99828	-0.85734
C	-2.43425	-0.95956	0.51604
C	-3.84662	-1.49278	-1.37383
C	-3.36193	-1.41246	1.42487
C	-4.79343	-1.96018	-0.45919
H	-4.02003	-1.51875	-2.44238
C	-4.55687	-1.92086	0.90940
H	-3.16550	-1.37734	2.48919
H	-5.72985	-2.36305	-0.82694
H	-5.31140	-2.29351	1.59218
C	1.30083	1.78966	1.24161
C	0.92480	3.14769	0.66082
H	2.18815	1.87776	1.87993
H	0.49550	1.41266	1.88178
H	0.01788	3.08038	0.05750
H	0.73959	3.88161	1.45050
H	1.72309	3.54017	0.02514
C	-3.60054	2.12308	-0.11182
N	-2.51149	2.43604	-0.30377
C	-4.97099	1.72319	0.12959
H	-5.33903	2.18624	1.04637
H	-5.60239	2.03419	-0.70402
H	-5.02429	0.63703	0.23051

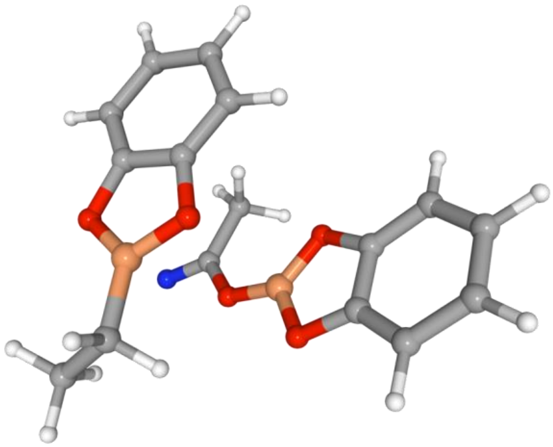

CatBOBCat_Et_rad_ACN_bound Energy: -1099.022656 Hartrees

40

C	2.44024	-0.55414	0.93039
C	1.11647	-0.87057	1.20363
C	0.65464	-2.16633	1.18683
C	1.58793	-3.15459	0.87681
C	2.91379	-2.83827	0.59820
C	3.36904	-1.52086	0.61965
H	-0.38340	-2.39565	1.39227
H	1.27133	-4.19036	0.84868
H	3.60948	-3.63296	0.35657
H	4.39581	-1.26192	0.39513
O	2.60147	0.79805	0.99990
O	0.42069	0.28288	1.43390
B	1.34956	1.31698	1.29916
O	0.36829	1.07526	-1.64921
B	-0.81012	0.62279	-1.15495
O	-1.29114	-0.67500	-1.16169
O	-1.69648	1.50656	-0.56712
C	-2.49488	-0.59655	-0.50788
C	-2.73642	0.72260	-0.14859
C	-3.37420	-1.60937	-0.21130
C	-3.87446	1.09282	0.52687
C	-4.53268	-1.24530	0.47541
H	-3.17324	-2.63356	-0.49935
C	-4.77594	0.07426	0.83545
H	-4.05392	2.12360	0.80458
H	-5.25630	-2.00946	0.73237
H	-5.68627	0.32025	1.36882
C	1.01989	2.82551	1.44860
C	1.98411	3.74539	0.70387
H	1.01588	3.06693	2.52015
H	-0.01048	2.98291	1.11383
H	1.96987	3.54020	-0.36879
H	1.72328	4.79587	0.84860
H	3.01128	3.60341	1.04609
C	1.36383	0.38780	-2.28111
N	2.37260	1.02758	-2.66211
C	1.24043	-1.08472	-2.49747
H	1.15374	-1.60120	-1.53947
H	0.34899	-1.30689	-3.08659
H	2.12508	-1.44112	-3.02168

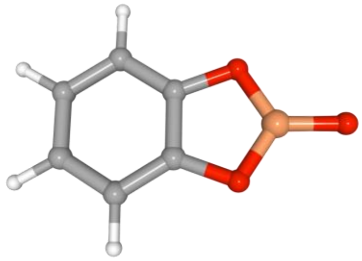

CatBO_rad Energy: -481.207518 Hartrees

14			
C	0.00000	0.69369	-0.10467
C	-0.00000	-0.69369	-0.10467
C	0.00000	-1.42319	-1.26878
C	0.00000	-0.69449	-2.45812
C	0.00000	0.69449	-2.45812
C	0.00000	1.42319	-1.26878
H	0.00000	-2.50553	-1.25525
H	0.00000	-1.22661	-3.40180
H	0.00000	1.22661	-3.40180
H	0.00000	2.50553	-1.25525
O	0.00000	1.15510	1.19151
O	-0.00000	-1.15510	1.19151
B	0.00000	0.00000	1.94893
O	0.00000	0.00000	3.31053

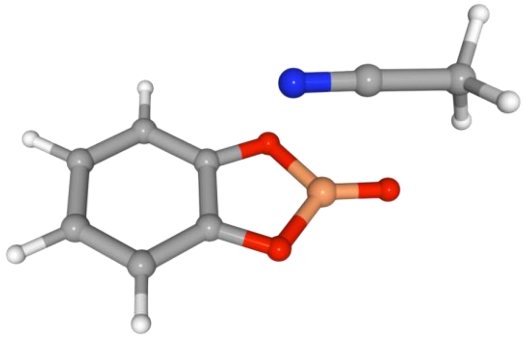

CatBO_rad_ACN_complex Energy: -613.828954 Hartrees

20			
O	2.12406	1.80255	-0.00334
B	0.96928	1.31617	-0.00250
O	0.12525	0.94664	-1.16440
O	0.12383	0.95362	1.16047
C	-0.96353	0.40016	-0.71871
C	-0.96445	0.40444	0.71667
C	-2.02941	-0.13476	-1.44335
C	-2.03123	-0.12607	1.44317
C	-3.06687	-0.65393	-0.71020
H	-2.01844	-0.13963	-2.52522
C	-3.06778	-0.64964	0.71184
H	-2.02164	-0.12441	2.52506
H	-3.91728	-1.08729	-1.22313
H	-3.91886	-1.07987	1.22628
C	2.51594	-1.45087	0.00385
N	1.45044	-1.88464	0.00584
C	3.83906	-0.86415	0.00103
H	4.38779	-1.18159	-0.88683
H	3.72522	0.22462	-0.00361
H	4.38812	-1.17383	0.89139

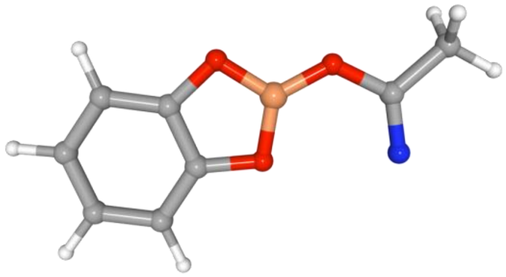

CatBO_rad_ACN_bound Energy: -613.847404 Hartrees

20

O	2.10307	-0.66738	-0.00009
B	0.77094	-0.38075	-0.00017
O	0.17608	0.86169	0.00040
O	-0.15119	-1.40995	-0.00060
C	-1.17049	0.59640	0.00035
C	-1.36710	-0.77777	-0.00026
C	-2.21897	1.48458	0.00077
C	-2.62455	-1.33306	-0.00048
C	-3.49945	0.93299	0.00056
H	-2.05195	2.55415	0.00123
C	-3.69726	-0.44246	-0.00005
H	-2.76807	-2.40612	-0.00096
H	-4.35810	1.59359	0.00087
H	-4.70741	-0.83408	-0.00020
C	3.11518	0.25067	-0.00047
N	2.94589	1.49082	-0.00250
C	4.46443	-0.38637	0.00173
H	4.56566	-1.01679	0.88778
H	4.56666	-1.02079	-0.88135
H	5.24300	0.37347	0.00050

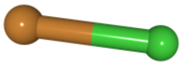

CuCl Energy: -2100.395943 Hartrees

2

Cl	0.00000	0.00000	-1.30887
Cu	-0.00000	-0.00000	0.76727

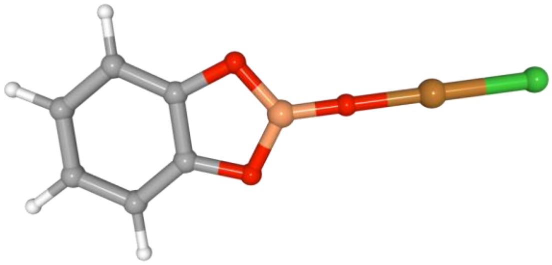

CatBO_rad_CuCl Energy: -2581.677804 Hartrees

16

B	-0.34325	0.00008	0.00007
O	-1.12642	-1.15135	-0.00006
O	-1.12644	1.15147	0.00023
O	0.98497	-0.00010	0.00012
C	-2.41647	-0.69469	0.00003
C	-3.58292	-1.42173	0.00004
C	-2.41650	0.69480	-0.00018
C	-3.58296	1.42182	-0.00016

C	-4.77346	-0.69438	-0.00003
C	-4.77349	0.69444	-0.00014
H	-3.57068	-2.50431	-0.00011
H	-3.57077	2.50440	0.00003
H	-5.71697	-1.22701	-0.00008
H	-5.71700	1.22707	-0.00008
Cl	4.76505	0.00043	-0.00030
Cu	2.71393	-0.00033	0.00018

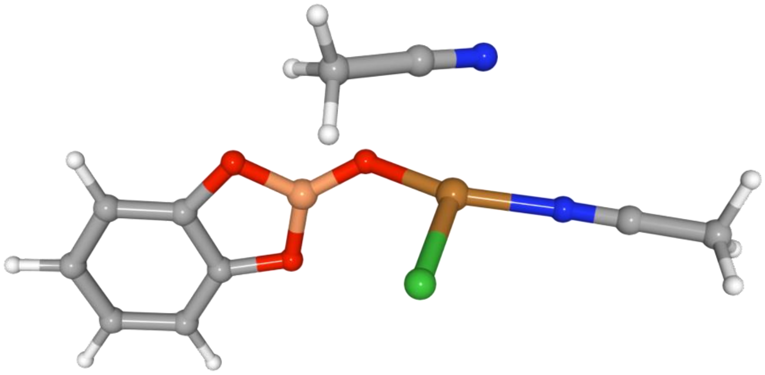

CatBO_rad_CuCl_2ACN Energy: -2846.939678 Hartrees

28

B	1.12756	0.09993	0.76107
O	1.71442	-1.16803	0.60888
O	2.09948	1.09809	0.53301
O	-0.12004	0.36322	1.09868
C	3.01093	-0.93926	0.27785
C	4.00555	-1.85207	0.01164
C	3.24706	0.43307	0.23625
C	4.48644	0.94672	-0.06870
C	5.26512	-1.34055	-0.30036
C	5.50077	0.02821	-0.33965
H	3.81042	-2.91669	0.04381
H	4.65918	2.01550	-0.09642
H	6.07546	-2.02666	-0.51716
H	6.49150	0.39156	-0.58618
Cl	-1.20252	-0.51616	-1.76403
Cu	-1.67052	-0.19261	0.36173
C	-4.57268	-1.18074	0.21925
N	-3.48906	-0.86407	0.39308
C	-1.78040	2.57344	-0.03463
N	-2.71978	2.18769	0.50941
C	-0.58454	2.99351	-0.72744
H	0.27306	2.88326	-0.06156
H	-0.44091	2.33312	-1.58622
H	-0.67792	4.02797	-1.05988
C	-5.94006	-1.57107	-0.01609
H	-6.04629	-1.90795	-1.04876
H	-6.21613	-2.38207	0.65950
H	-6.59653	-0.71643	0.15531

5. NMR SPECTROSCOPIC DATA

^{1}H NMR (400 MHz, $CDCl_3$) of **1** (***see procedure***)

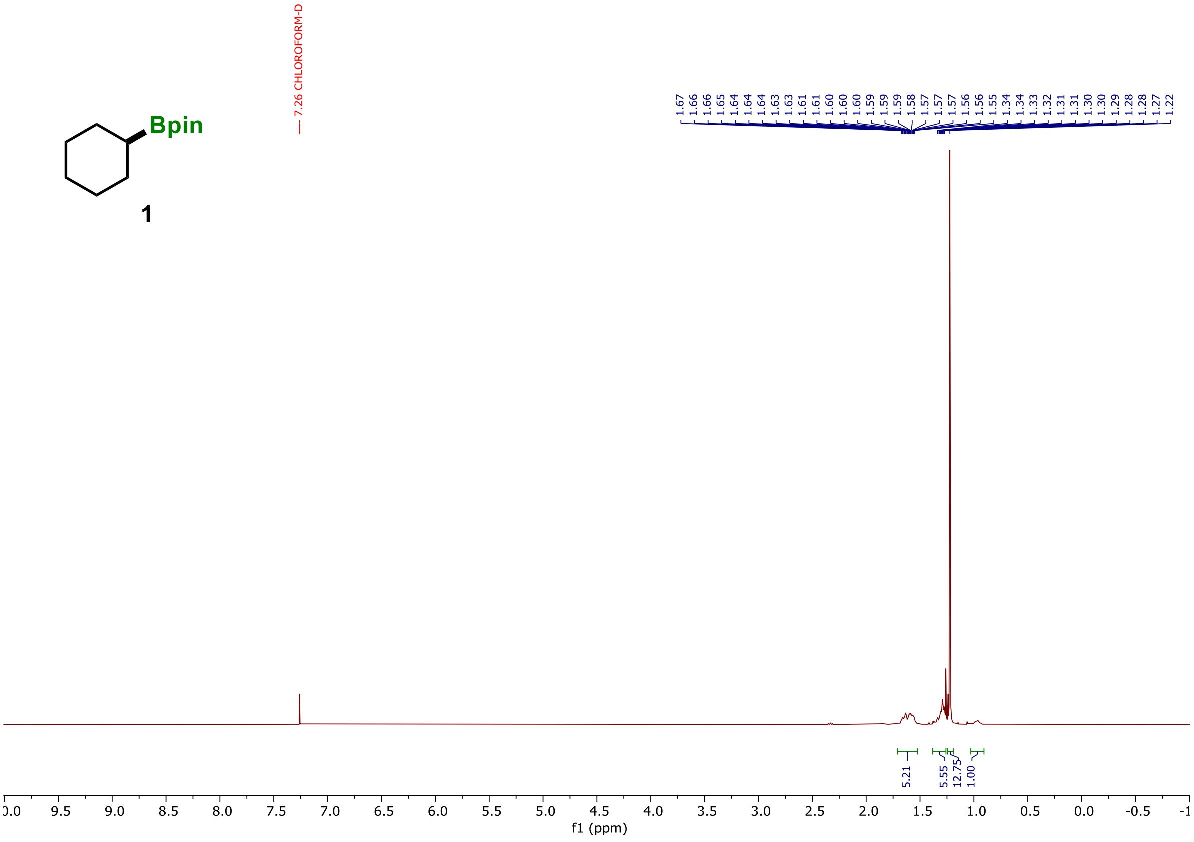

^{13}C NMR (101 MHz, $CDCl_3$) of **1**

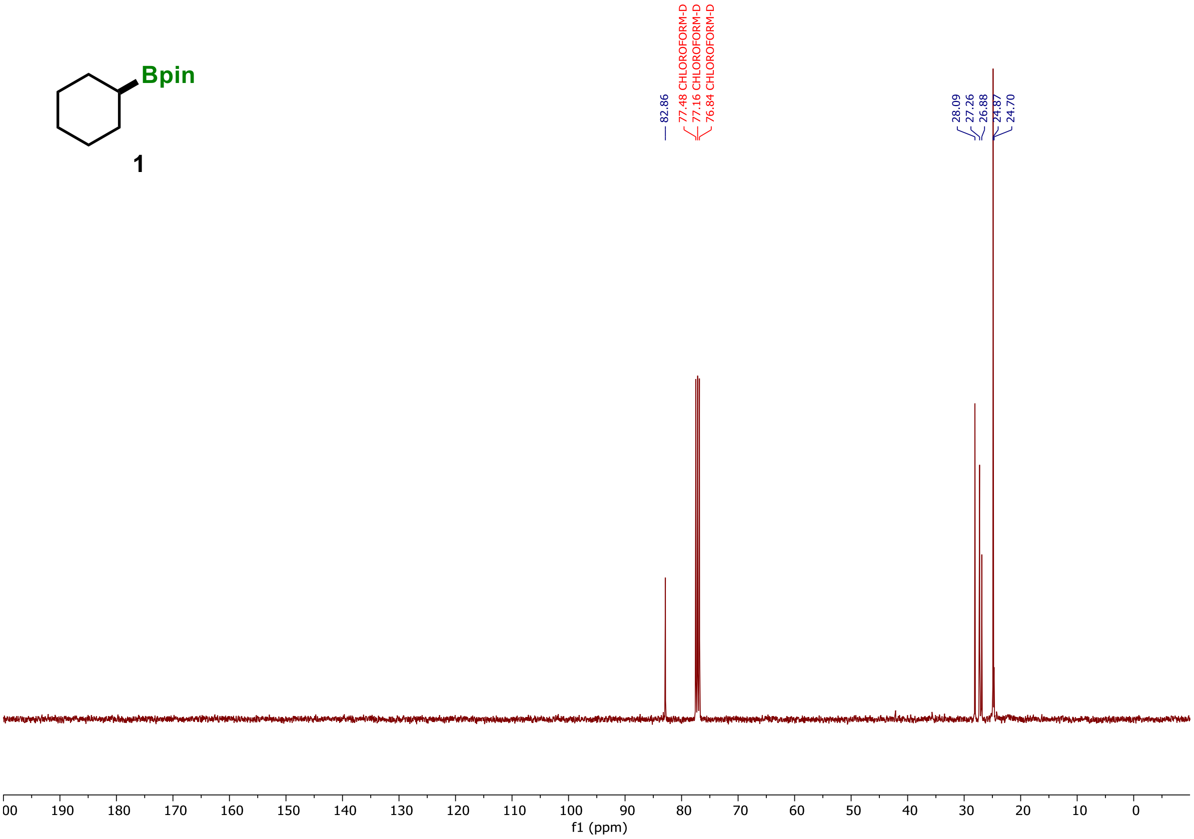

^{1}H NMR (400 MHz, $CDCl_3$) of **2** (***see procedure***)

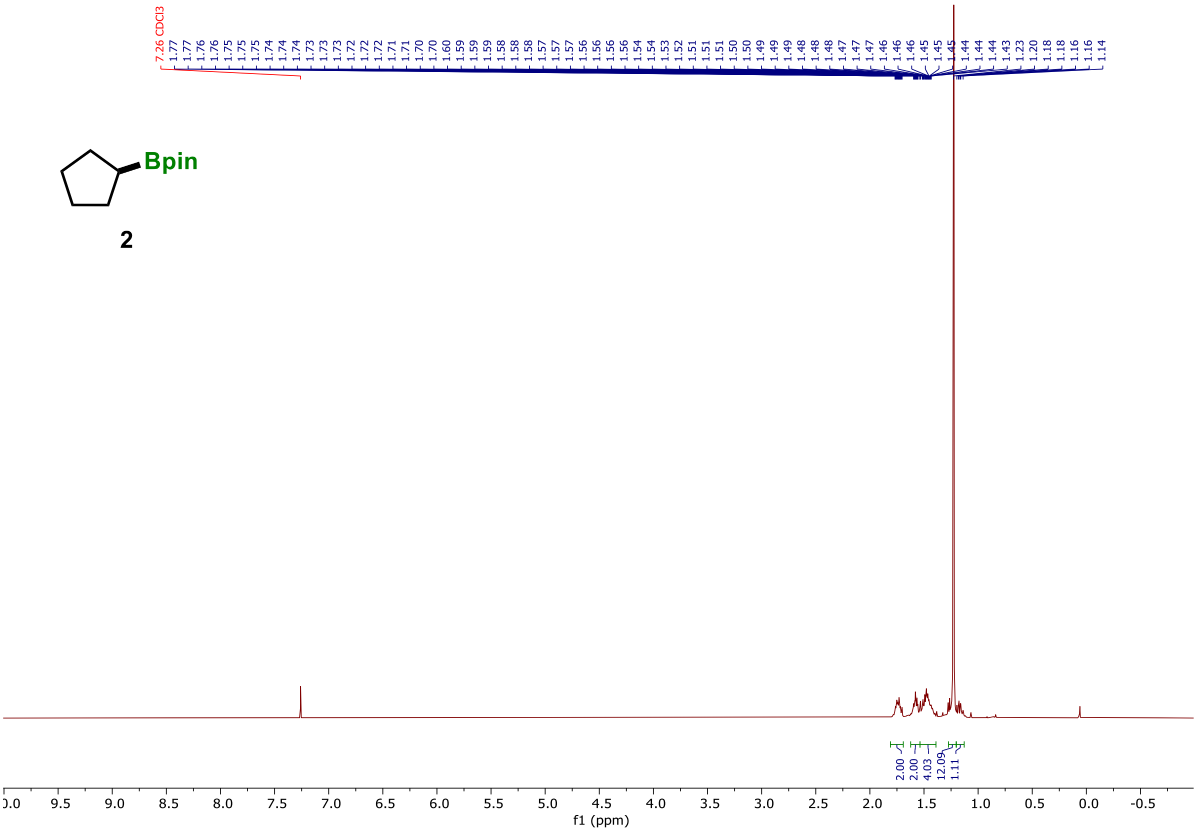

^{13}C NMR (101 MHz, $CDCl_3$) of **2**

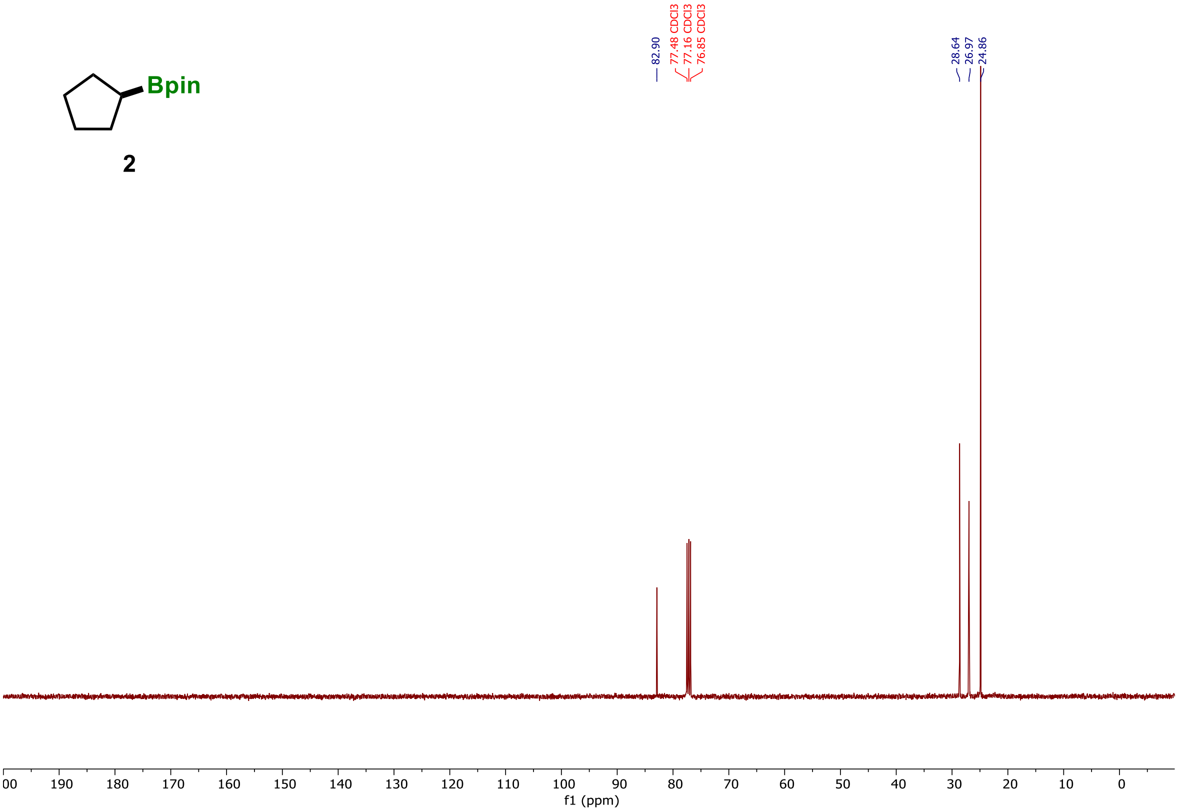

^{1}H NMR (400 MHz, $CDCl_3$) of **3** (*see procedure*)

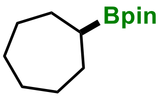

3

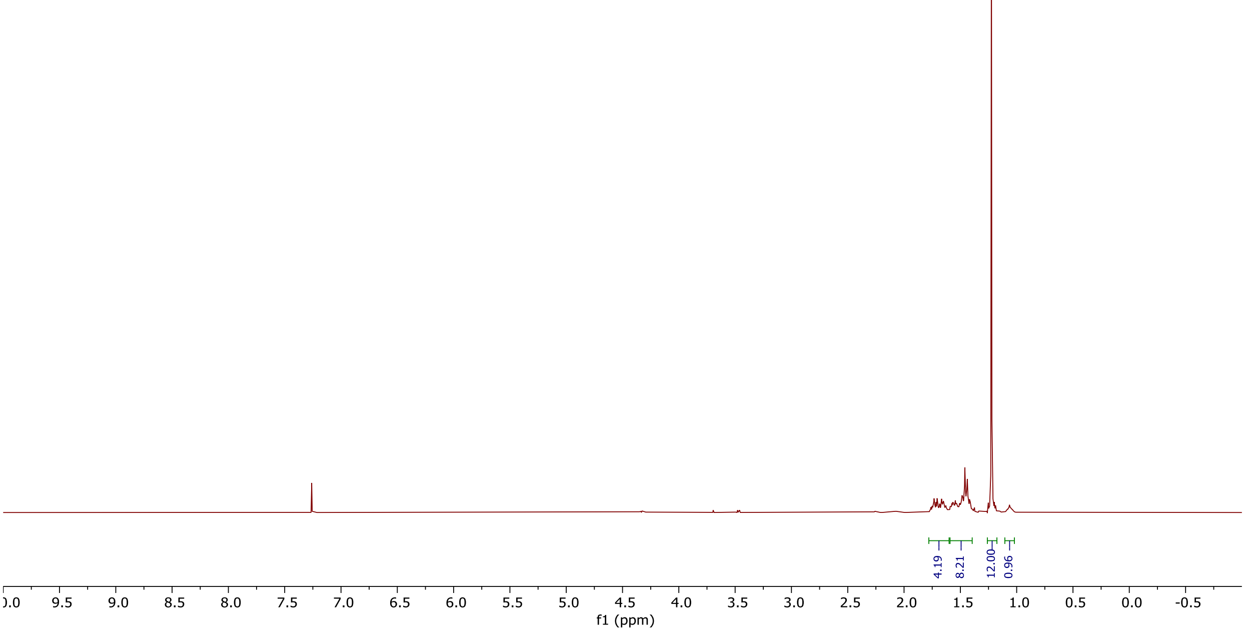

^{13}C NMR (101 MHz, $CDCl_3$) of **3**

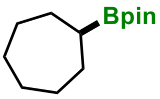

3

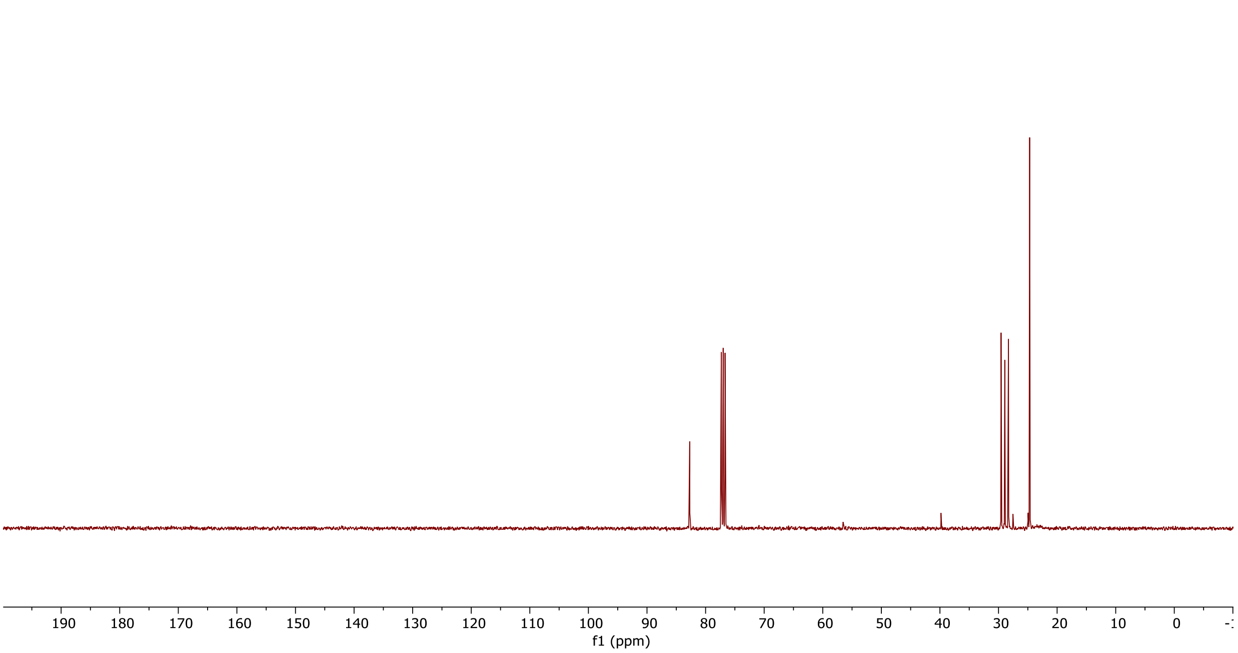

^{1}H NMR (400 MHz, $CDCl_3$) of **4** (***see procedure***)

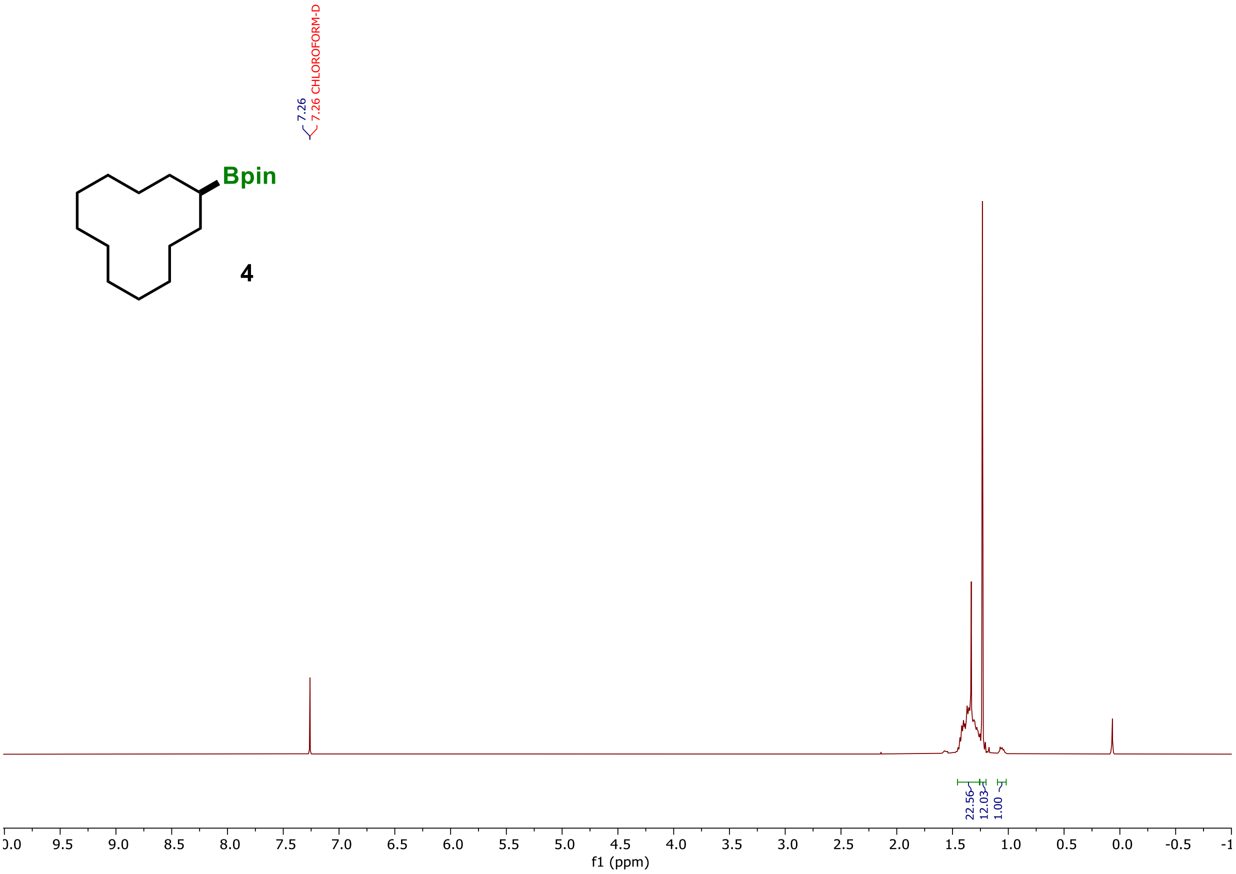

^{13}C NMR (101 MHz, $CDCl_3$) of **4**

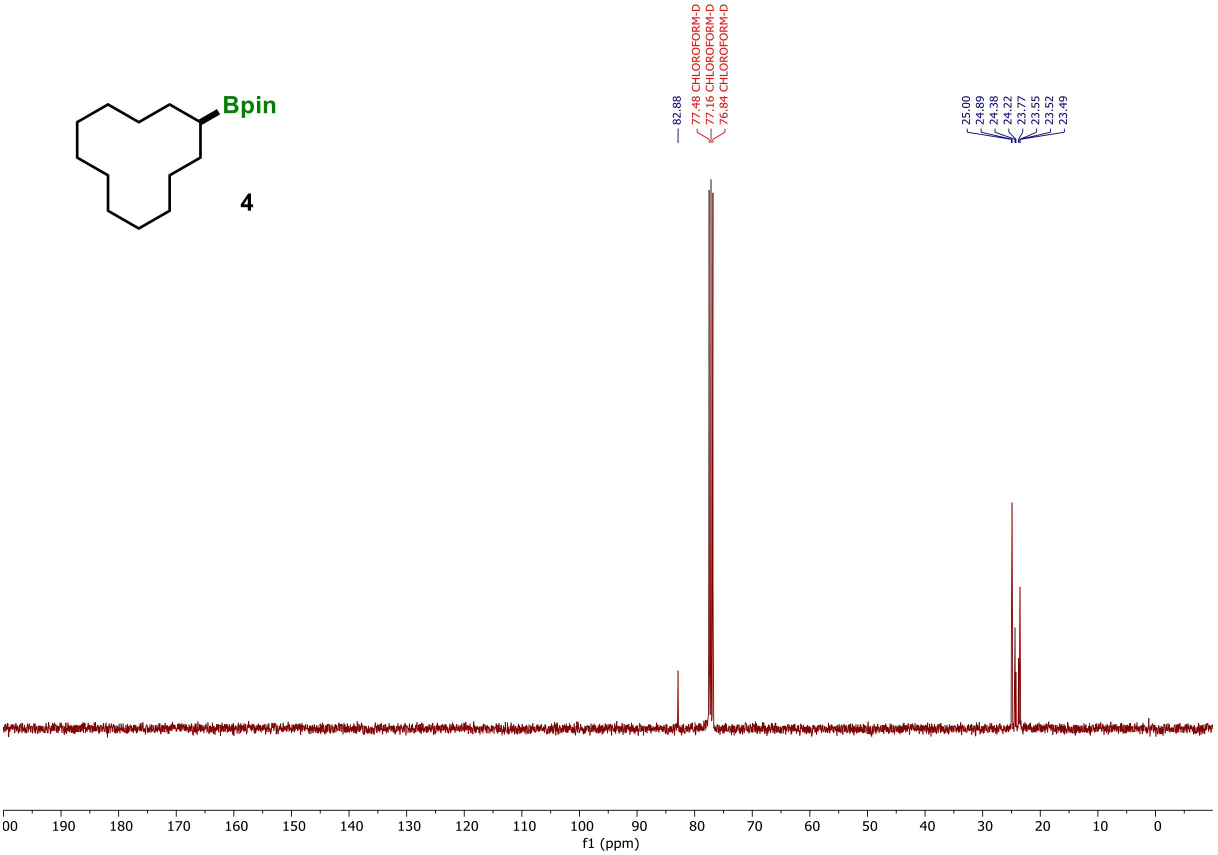

^{1}H NMR (400 MHz, $CDCl_3$) of **5** (*see procedure*)

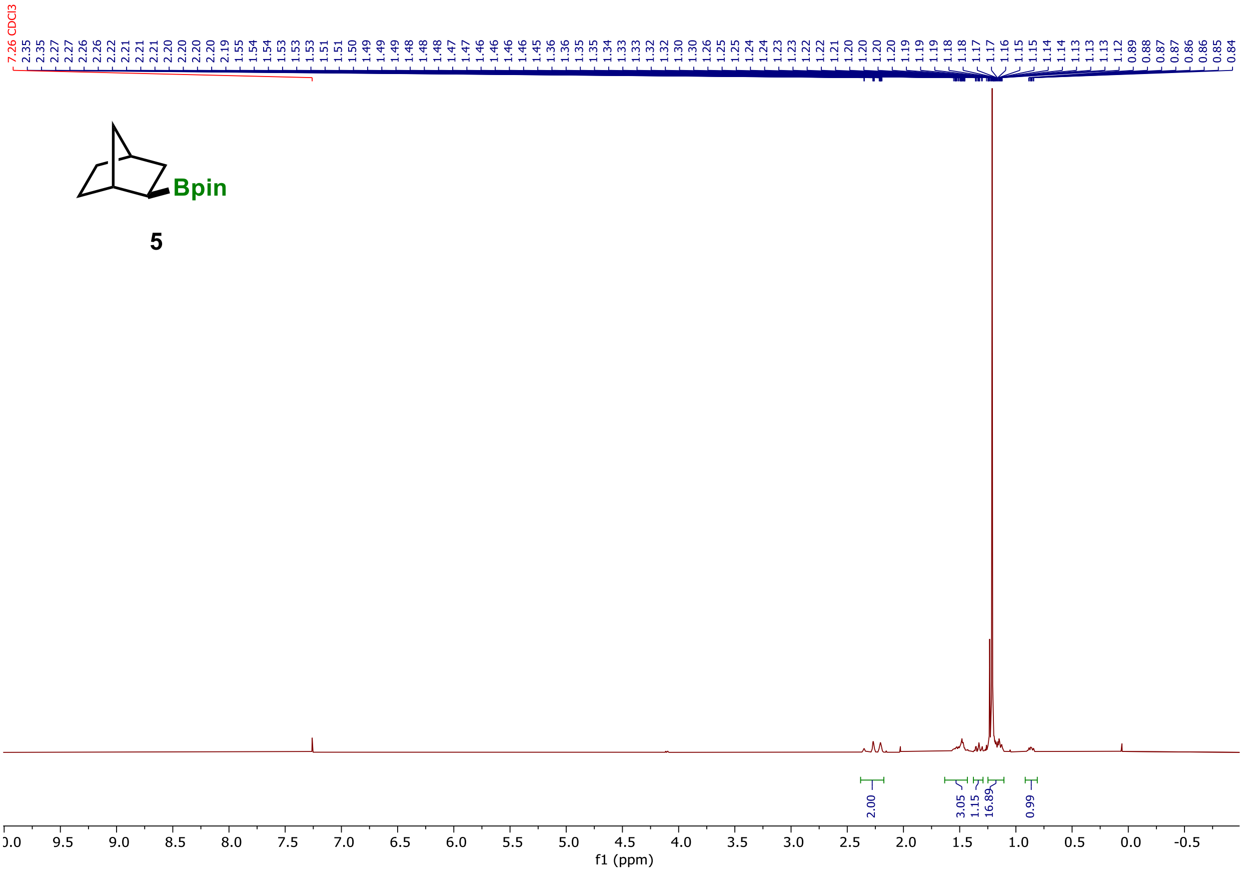

^{13}C NMR (101 MHz, $CDCl_3$) of **5**

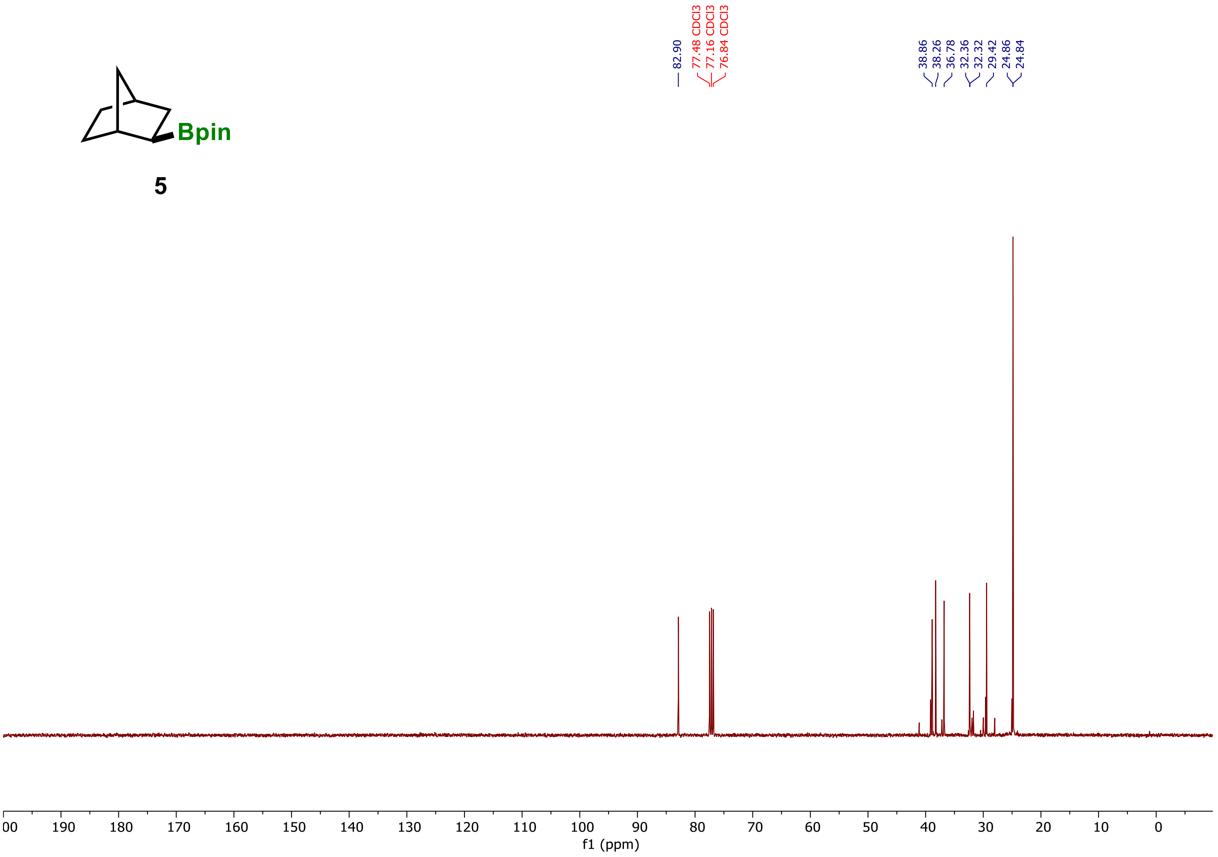

^{1}H NMR (400 MHz, $CDCl_3$) of **6-ox-a** (*see procedure*)

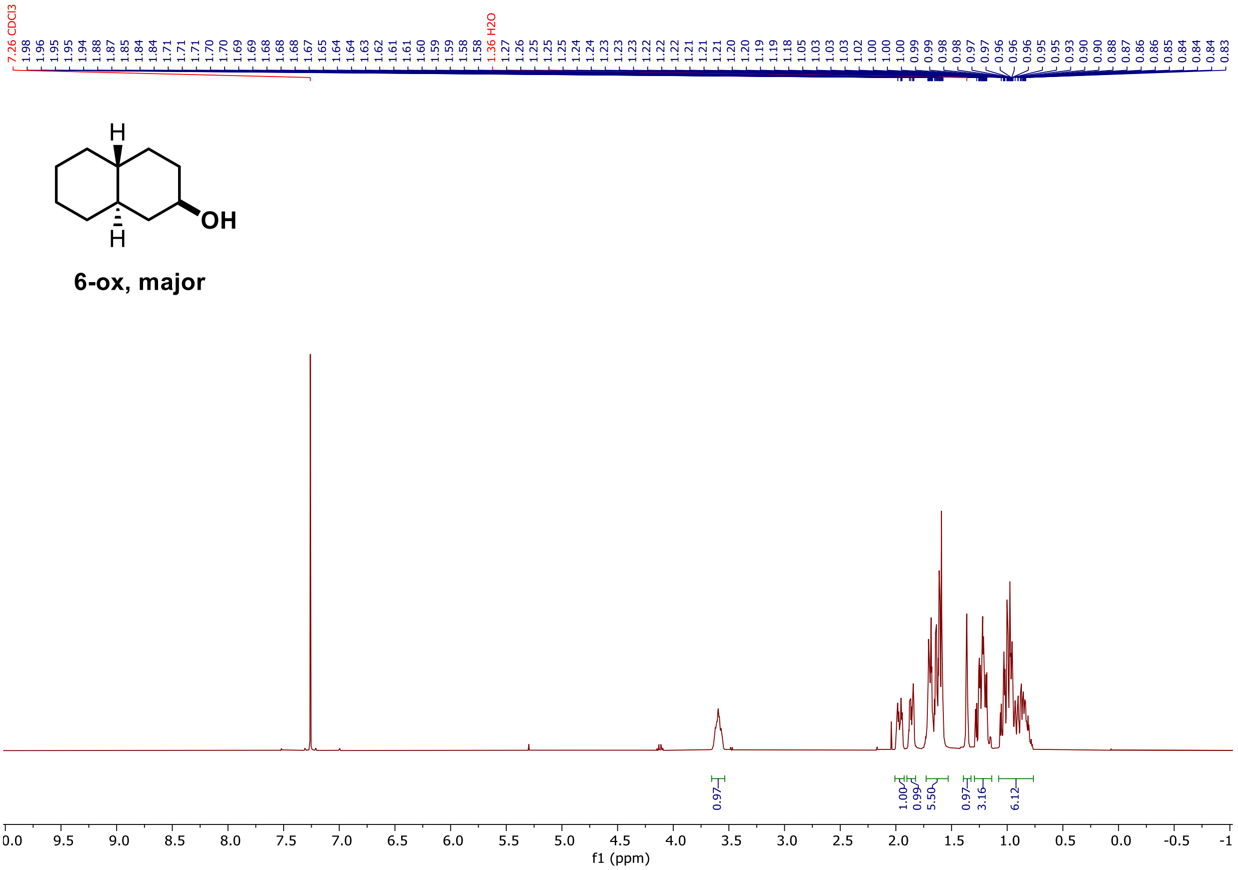

^{13}C NMR (101 MHz, $CDCl_3$) of **6-ox-a**

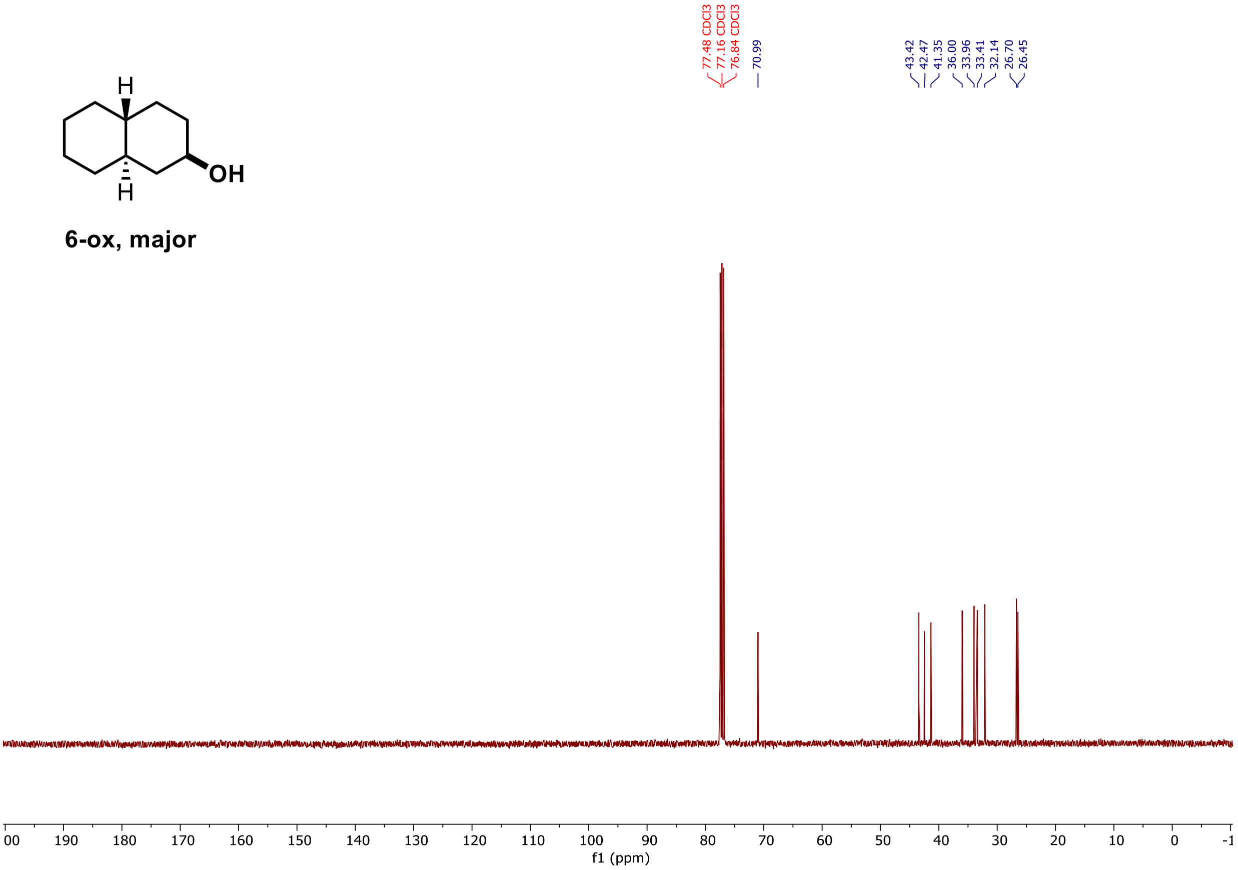

^{1}H NMR (400 MHz, $CDCl_3$) of **6-ox-b, minor** (*see procedure*)

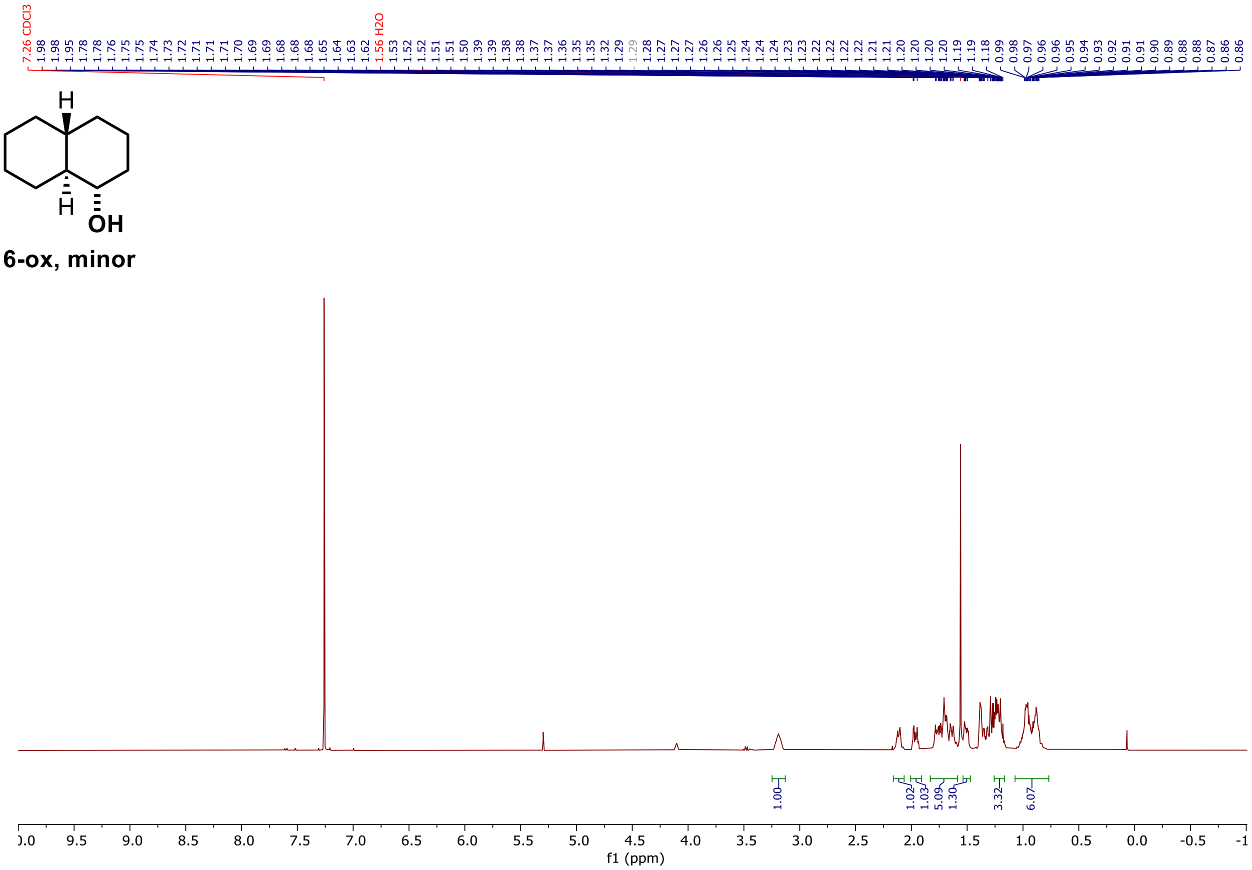

6-ox, minor

^{13}C NMR (101 MHz, $CDCl_3$) of **6-ox-b, minor**

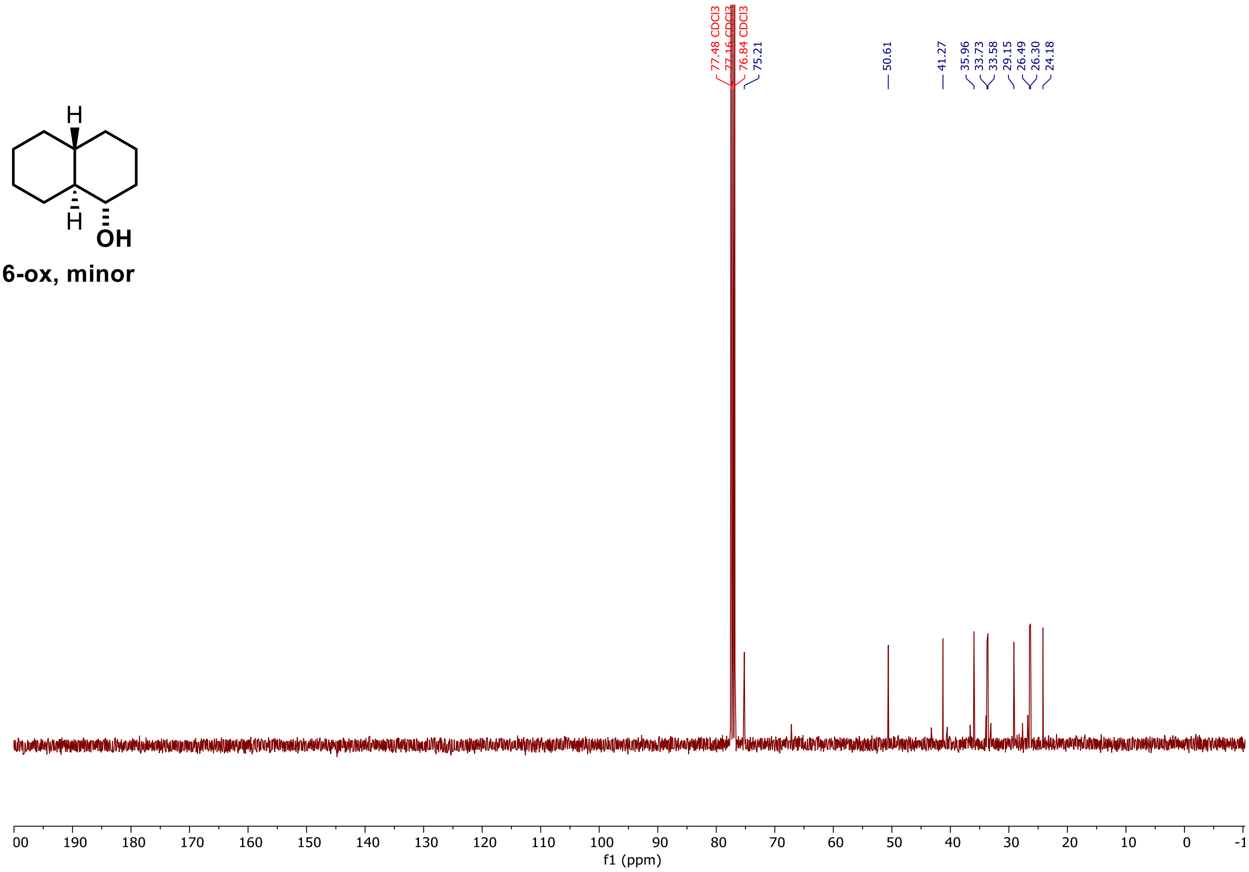

6-ox, minor

^{1}H NMR (400 MHz, $CDCl_3$) of crude **7-ox** (***see procedure***)

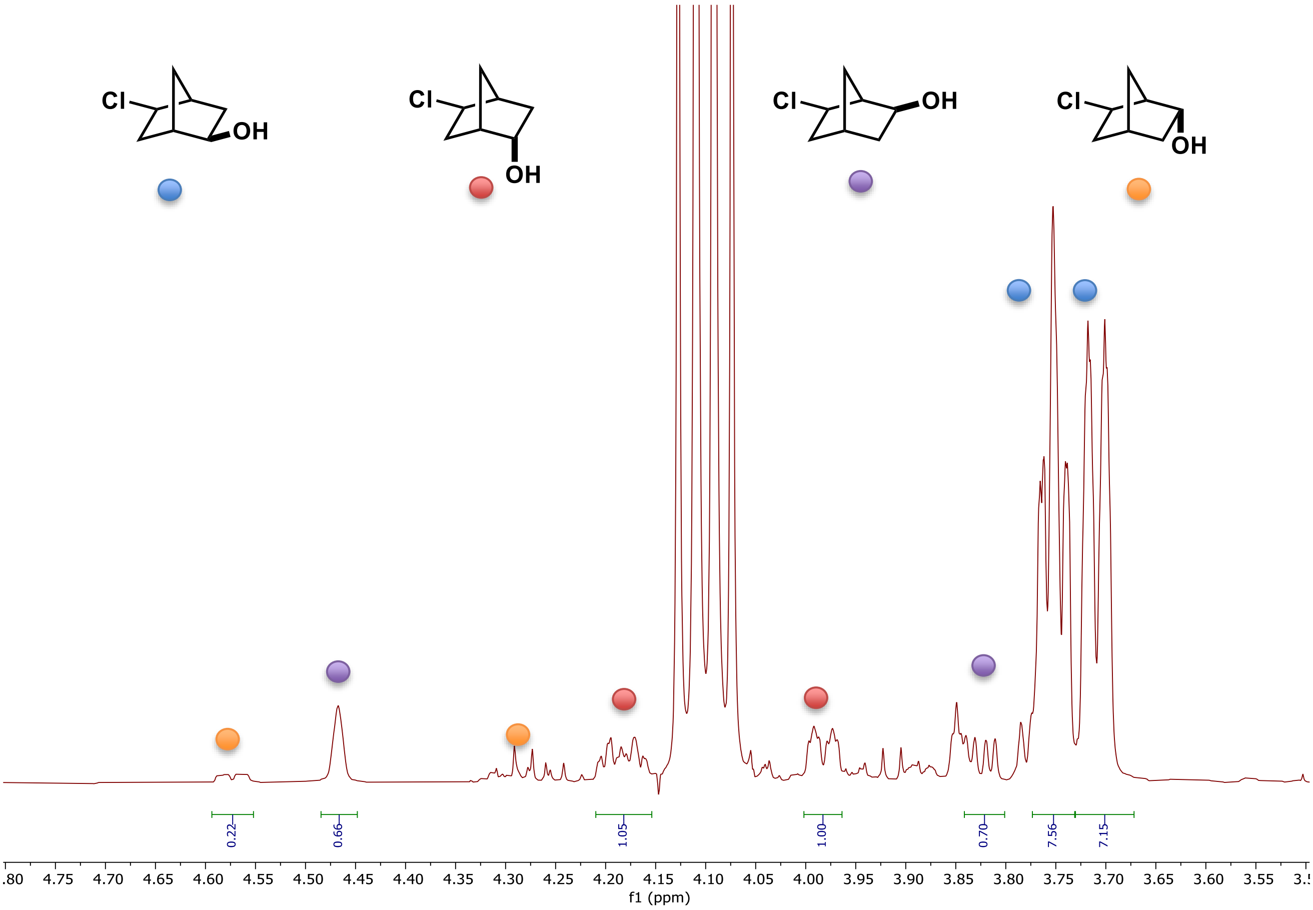

^{1}H NMR (400 MHz, $CDCl_3$) of **7-ox** (*see procedure*)

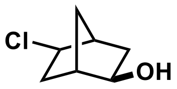

7-ox, major

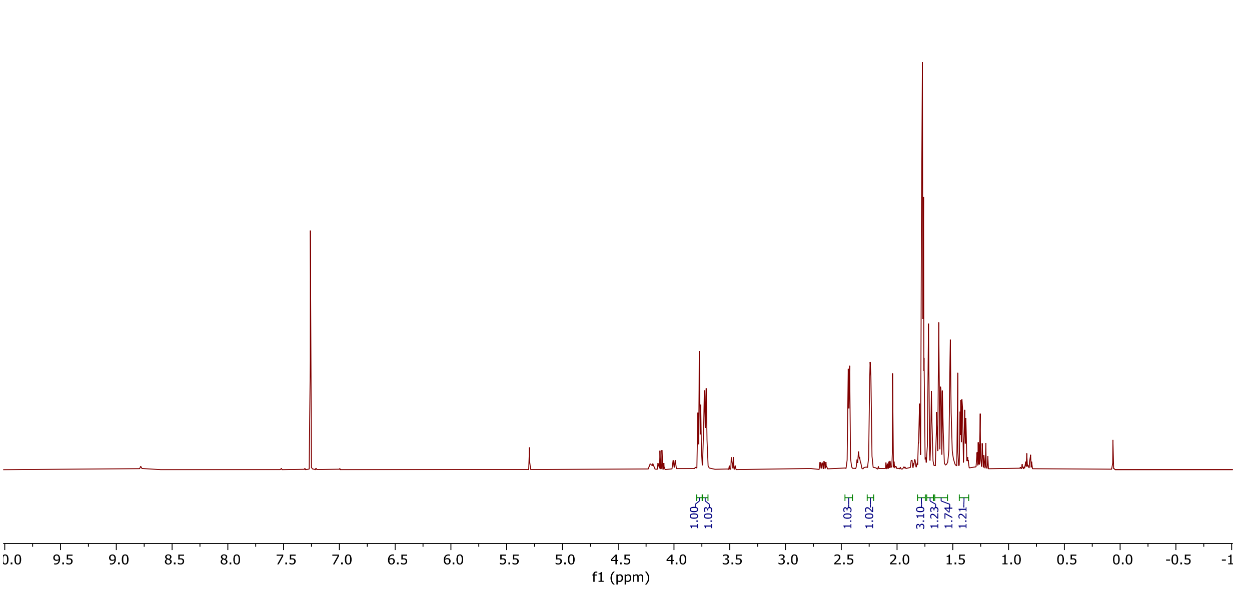

^{13}C NMR (101 MHz, $CDCl_3$) of **7-ox**

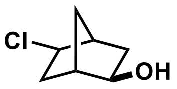

7-ox, major

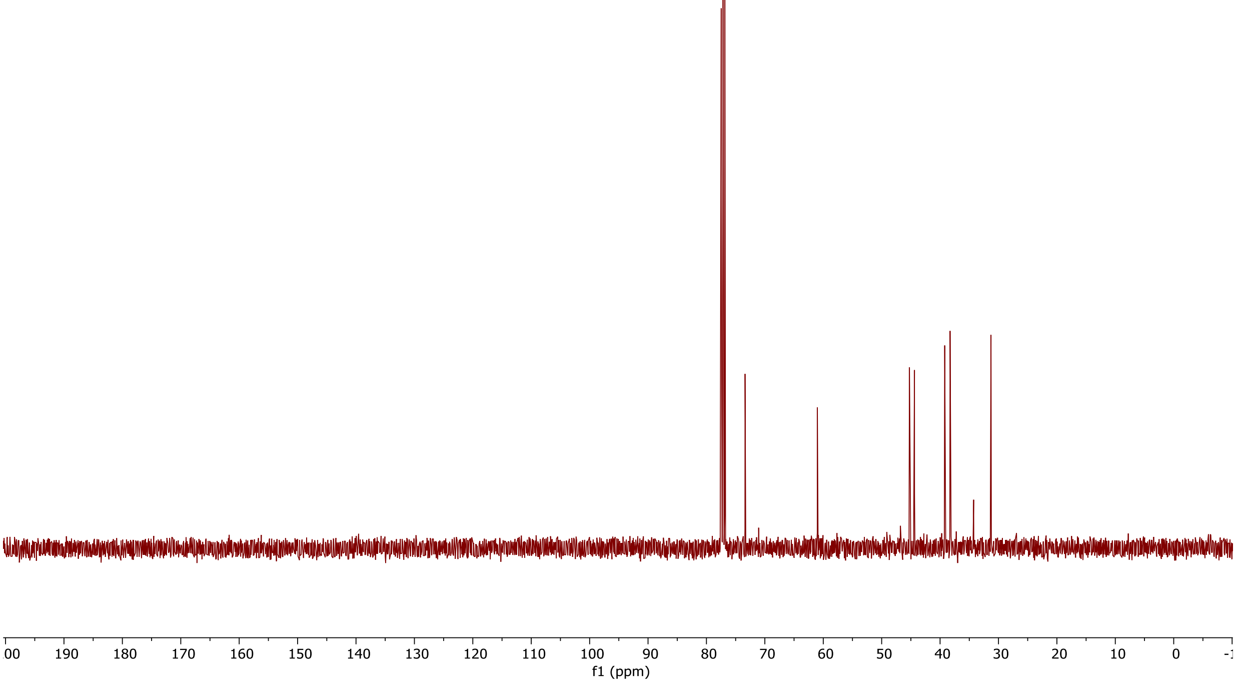

^{1}H NMR (400 MHz, $CDCl_3$) of crude **8-ox** (***see procedure***)

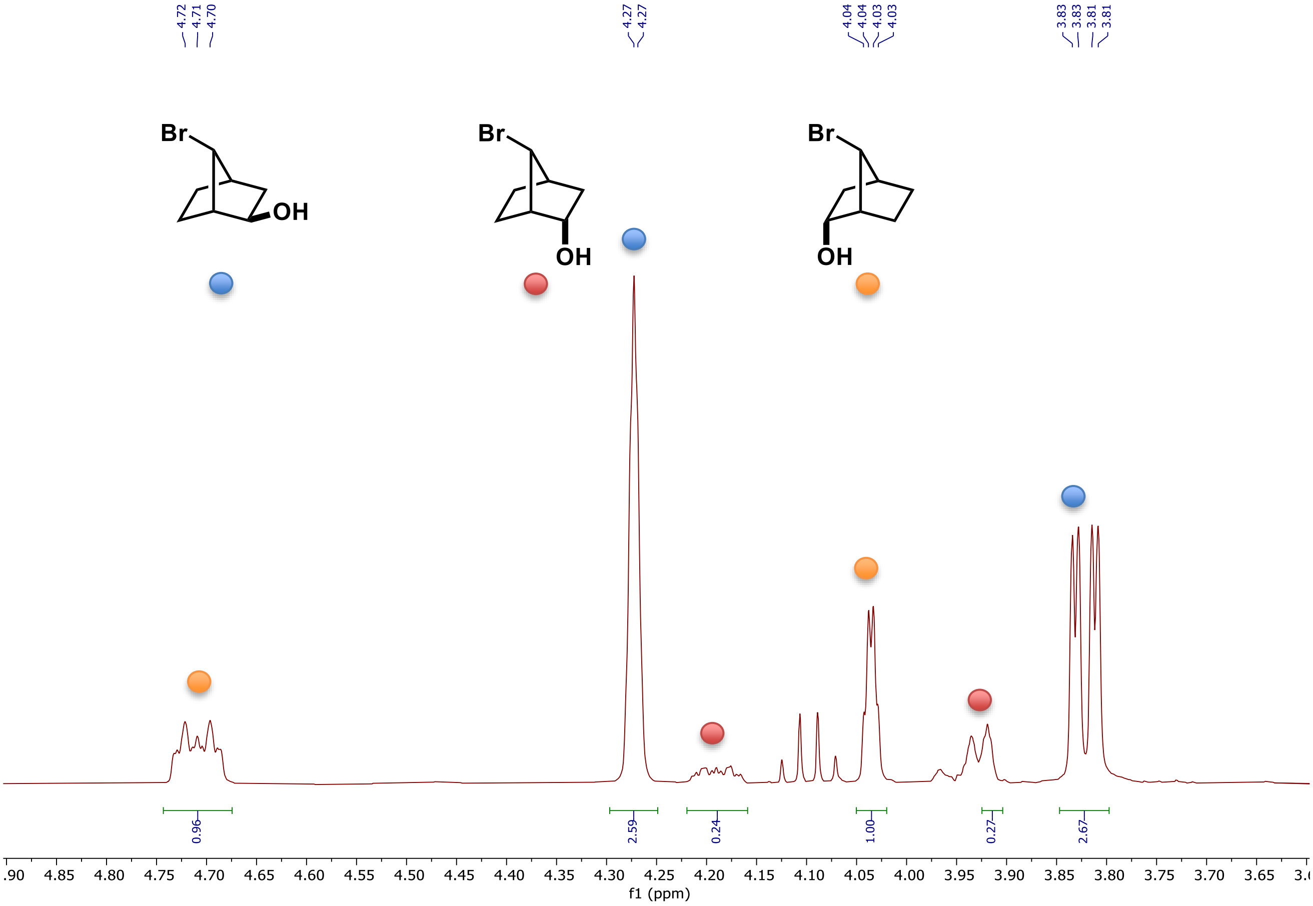

^{1}H NMR (400 MHz, $CDCl_3$) of **8-ox-a** (*see procedure*)

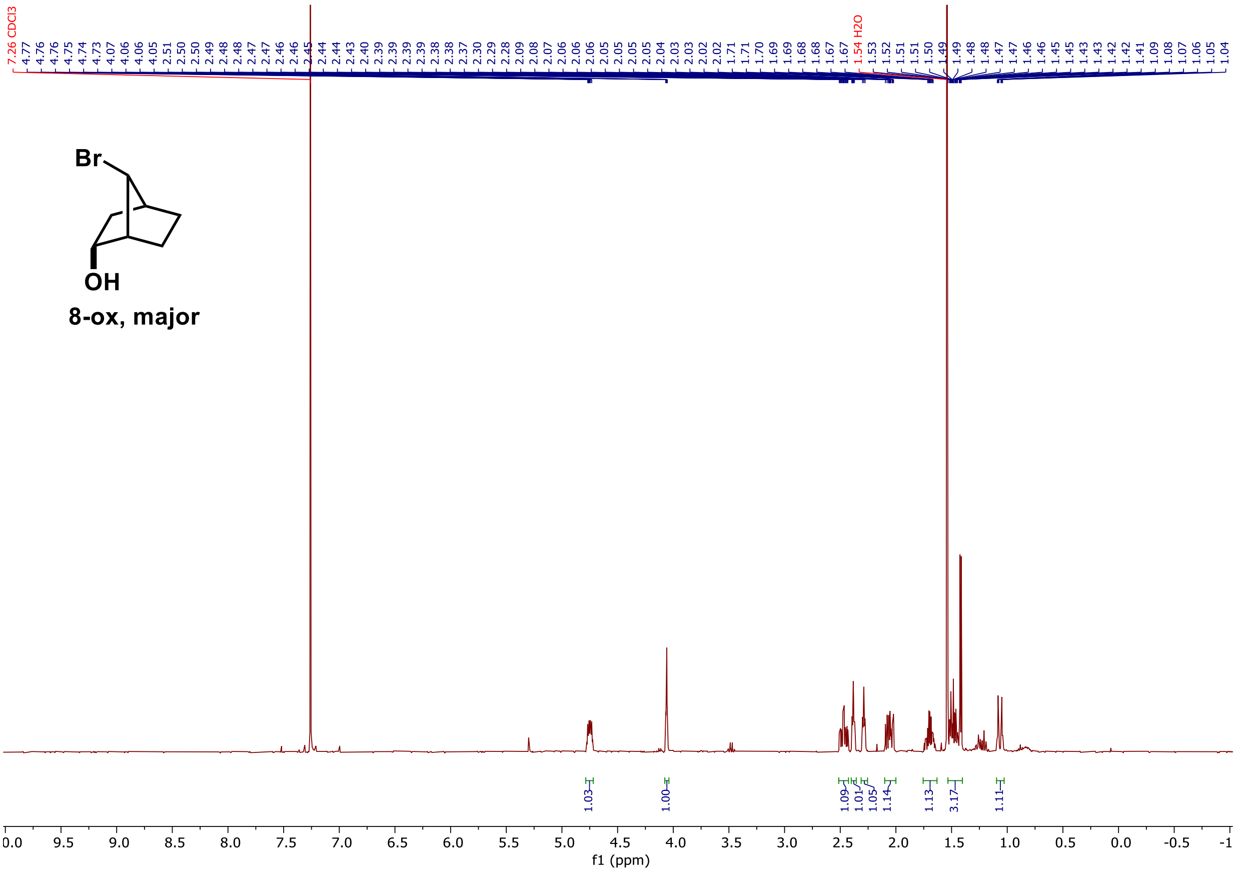

^{13}C NMR (101 MHz, $CDCl_3$) of **8-ox-a**

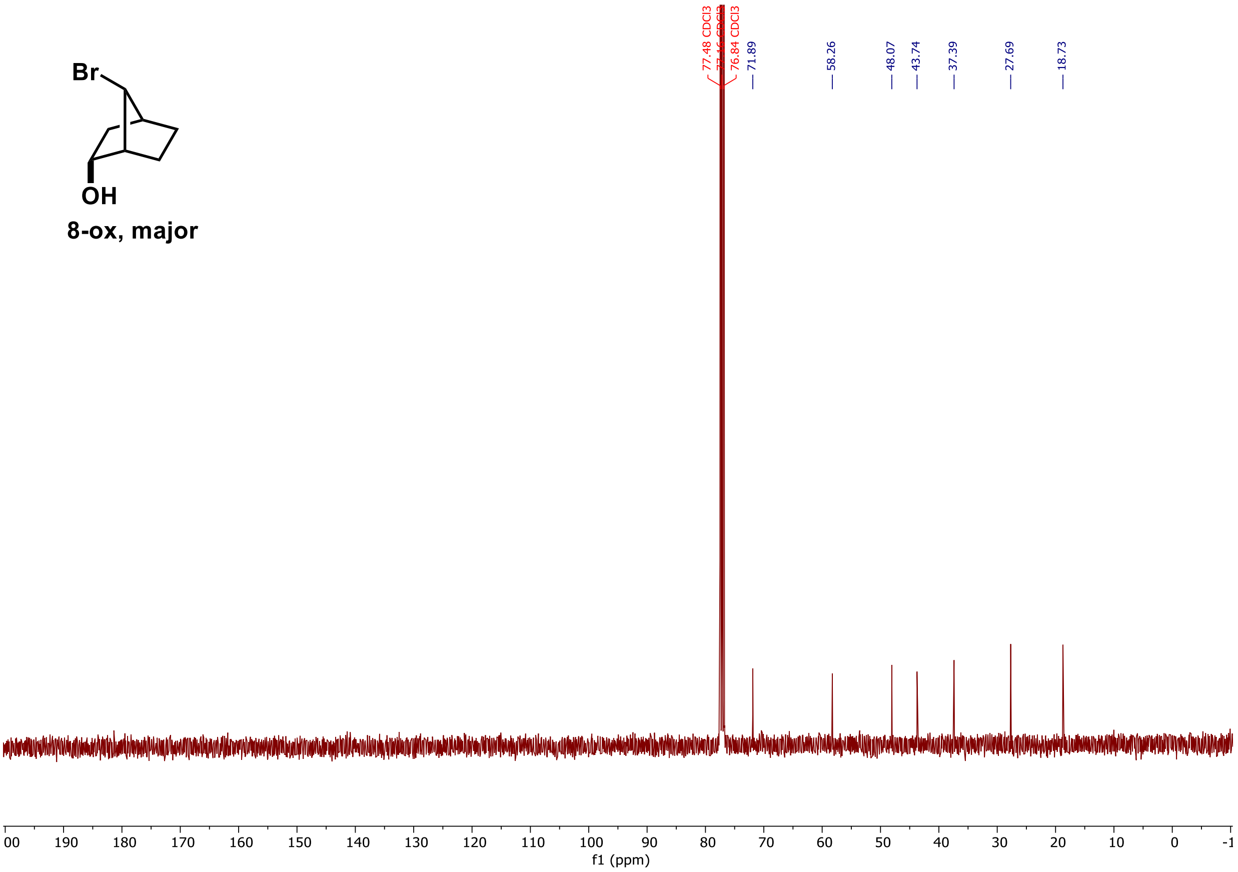

^{1}H NMR (400 MHz, CDCl$_3$) of **8-ox-b** (*see procedure*)

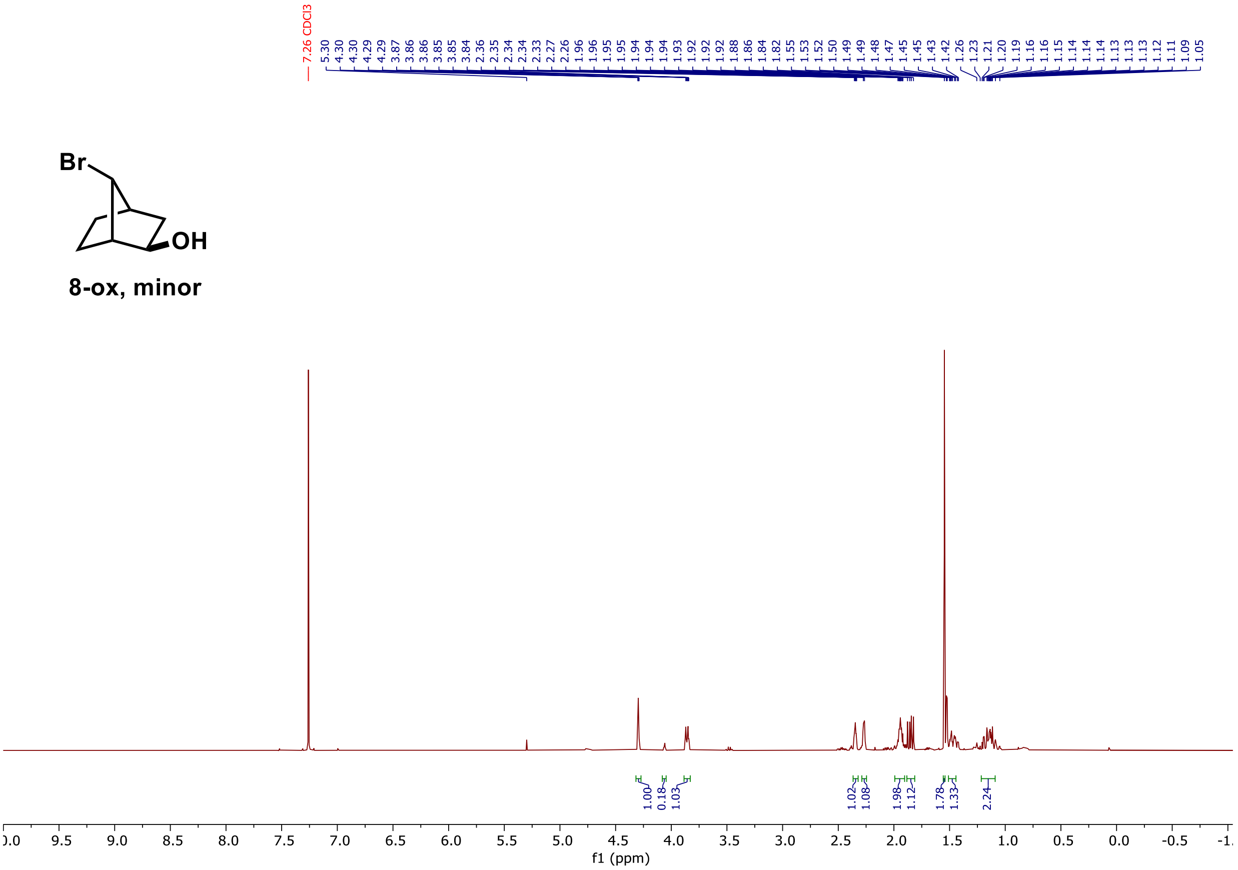

^{13}C NMR (101 MHz, CDCl$_3$) of **8-ox-b**

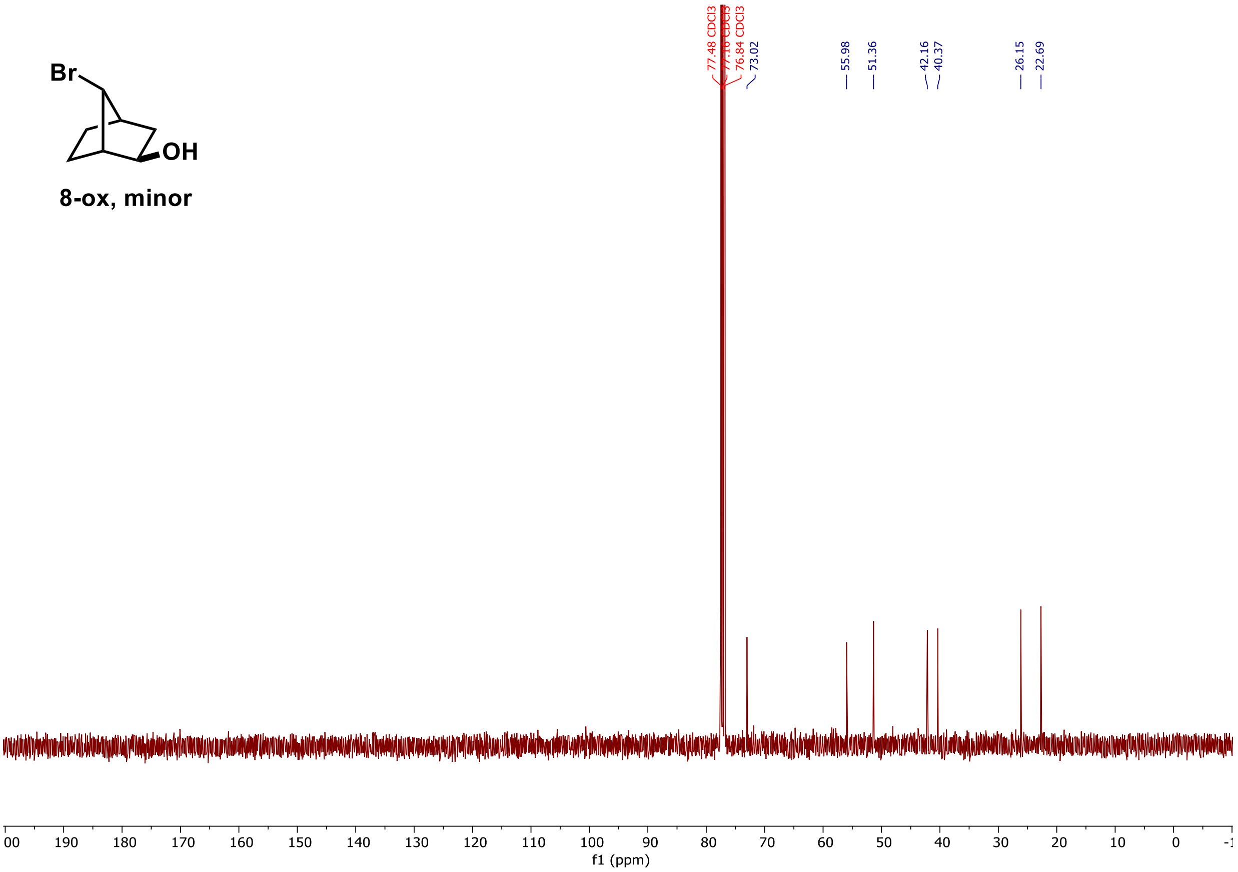

^{1}H NMR (400 MHz, $CDCl_3$) of **9a** and **9b** (*see procedure*)

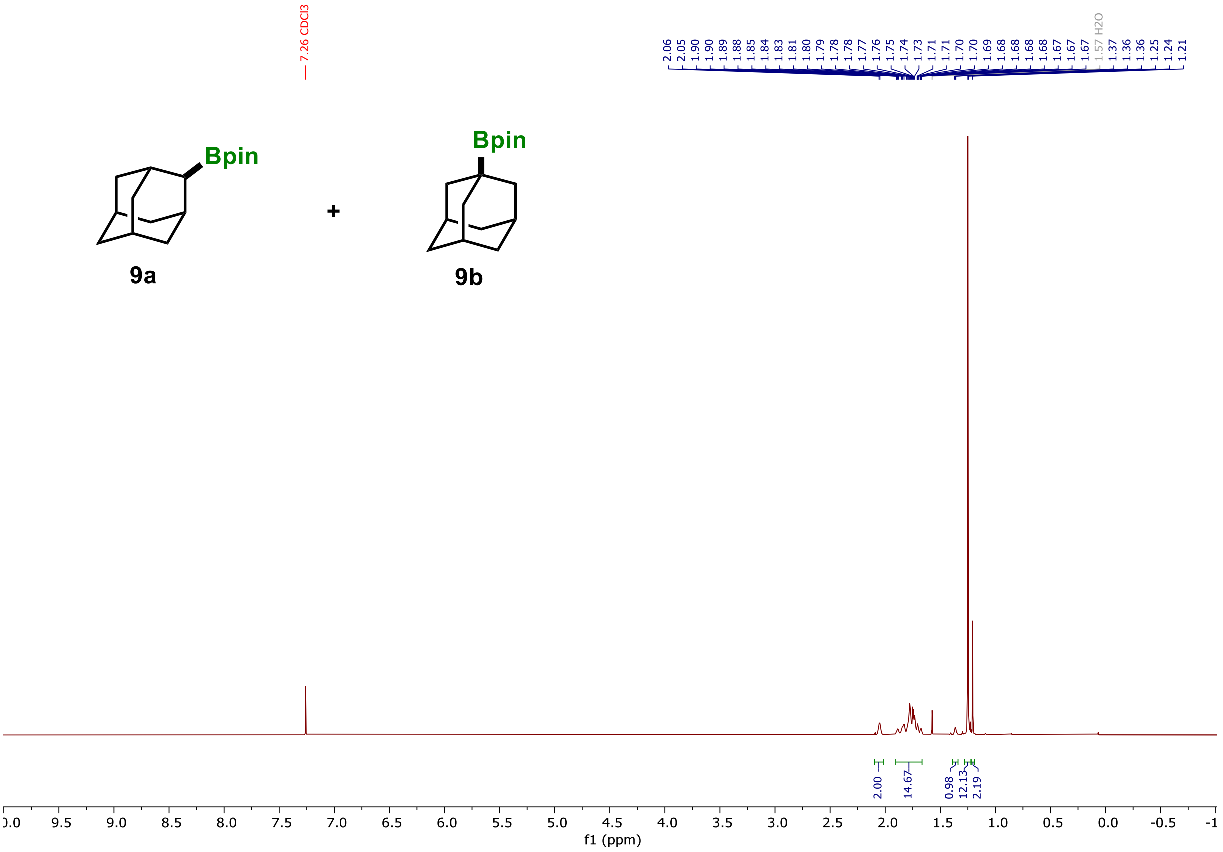

^{13}C NMR (101 MHz, $CDCl_3$) of **9a** and **9b**

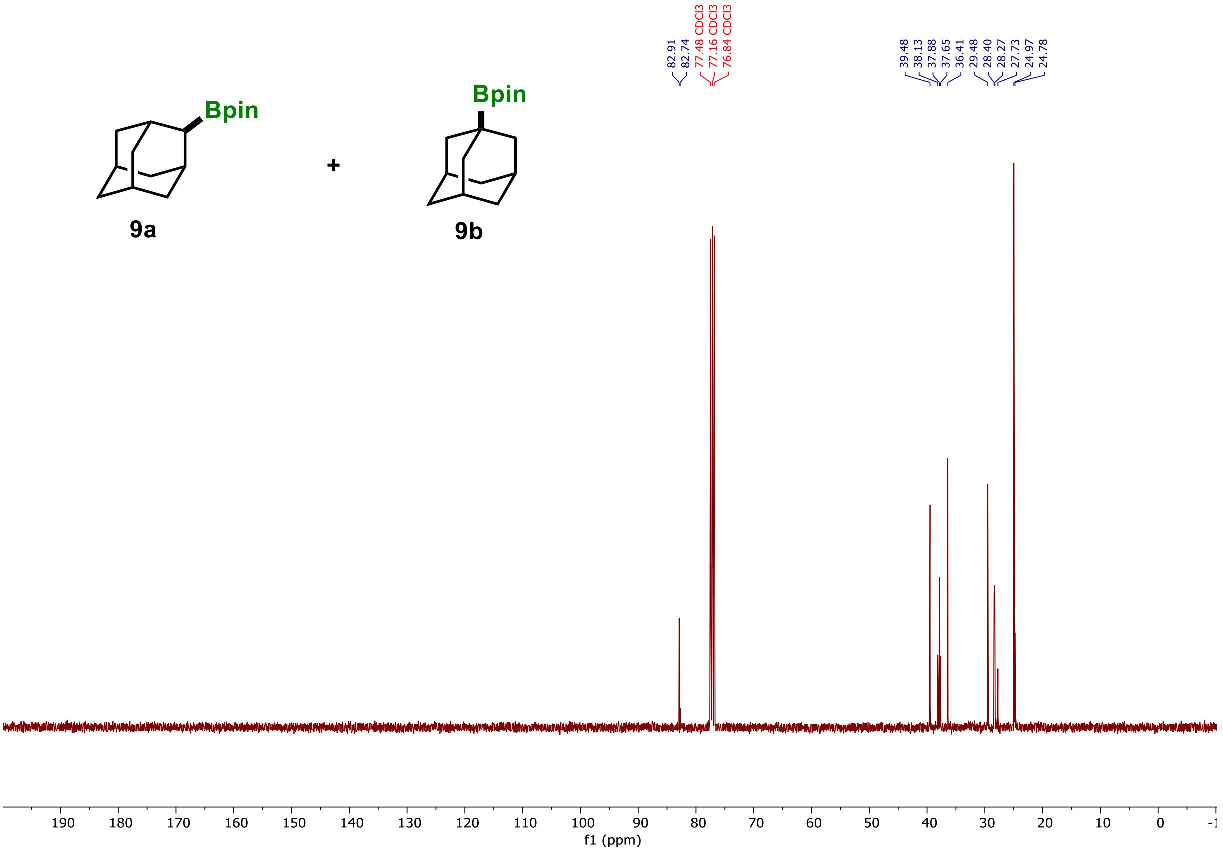

^{1}H NMR (400 MHz, $CDCl_3$) of **10a, 10b and 10c** (*see procedure*)

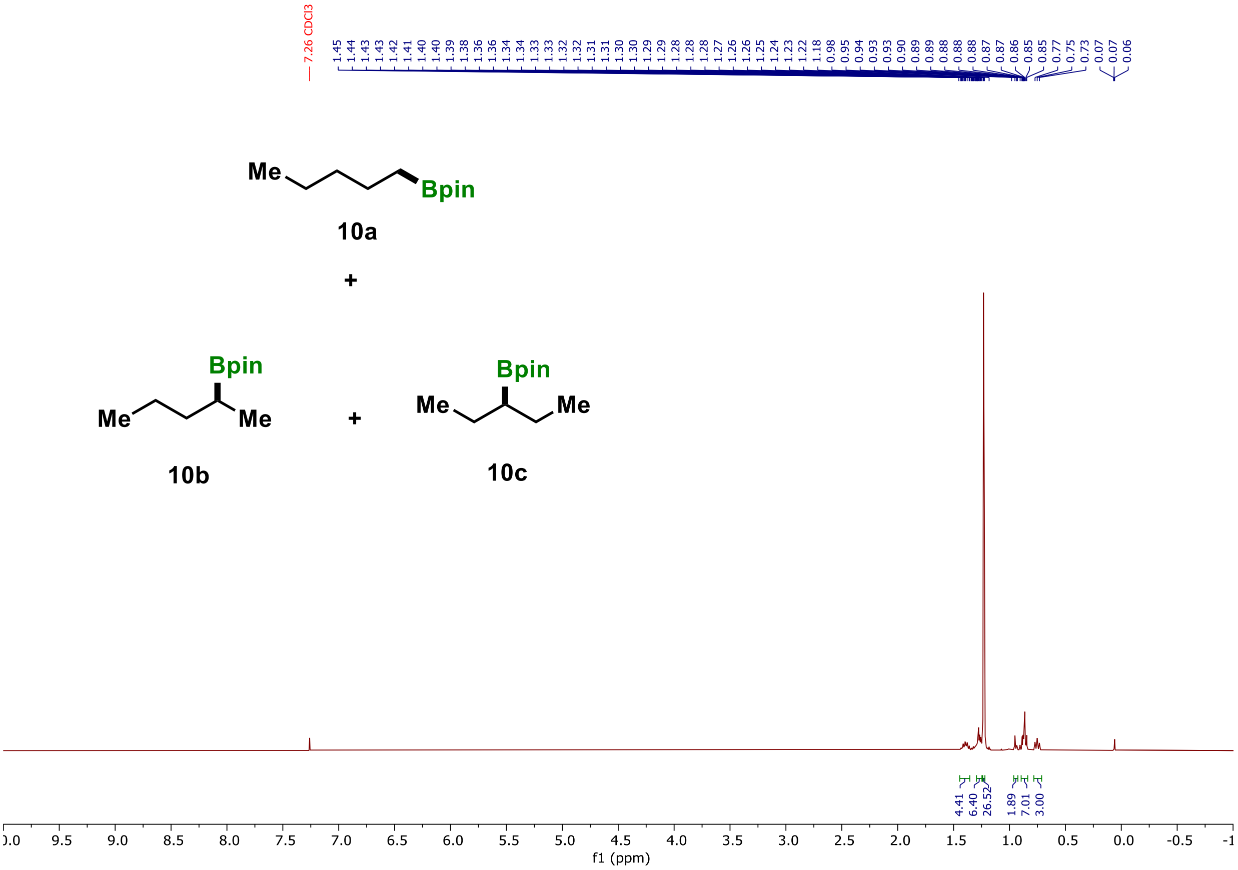

^{13}C NMR (101 MHz, $CDCl_3$) of **10a, 10b and 10c**

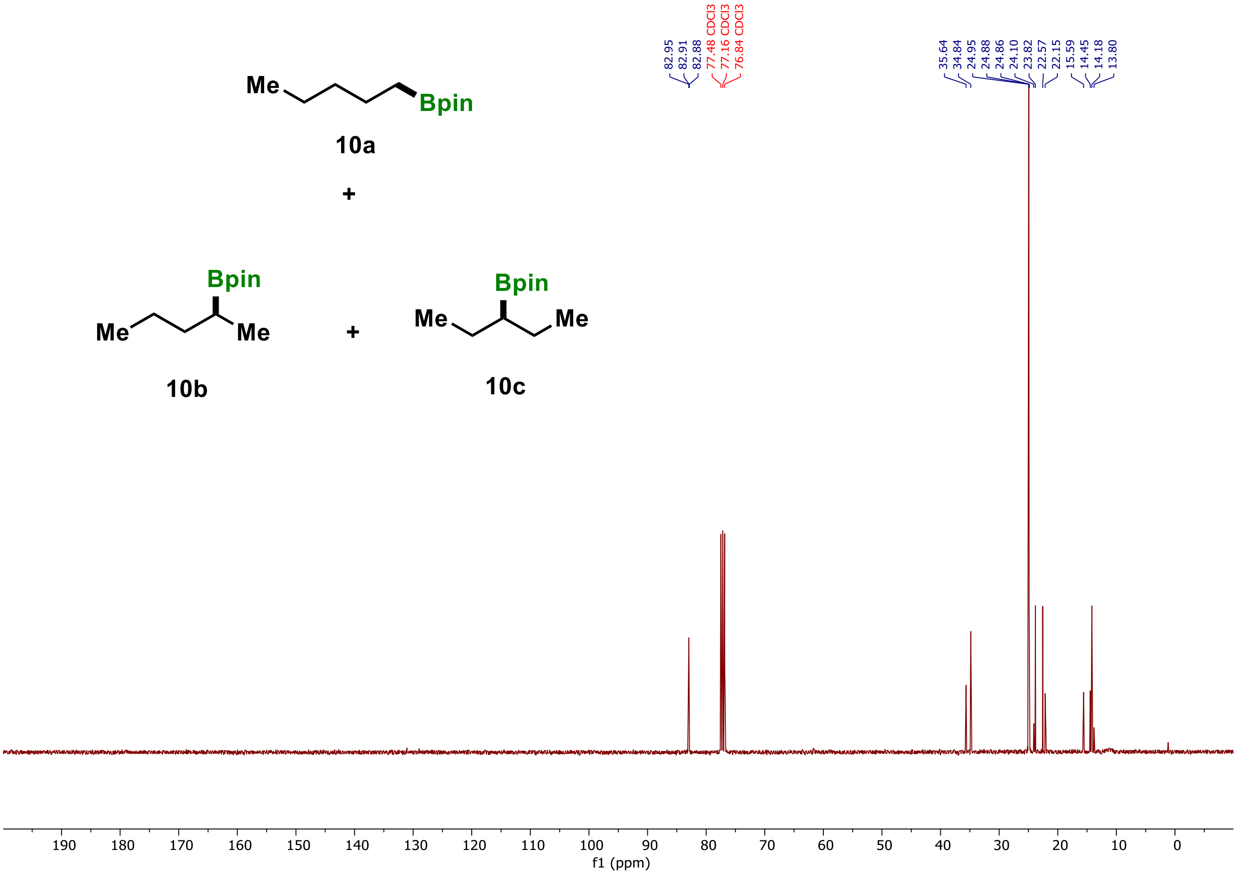

^{1}H NMR (400 MHz, $CDCl_3$) of **11** (***see procedure***)

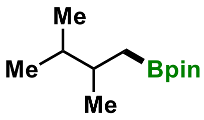

11

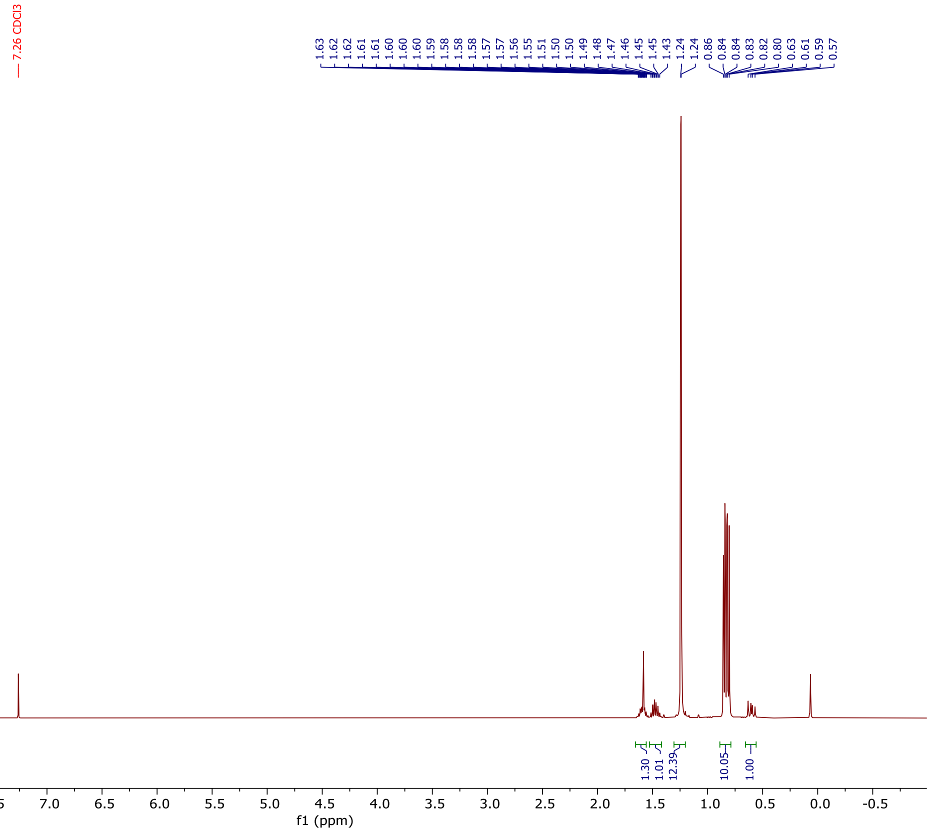

^{13}C NMR (101 MHz, $CDCl_3$) of **11**

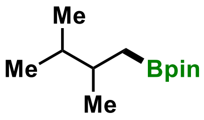

11

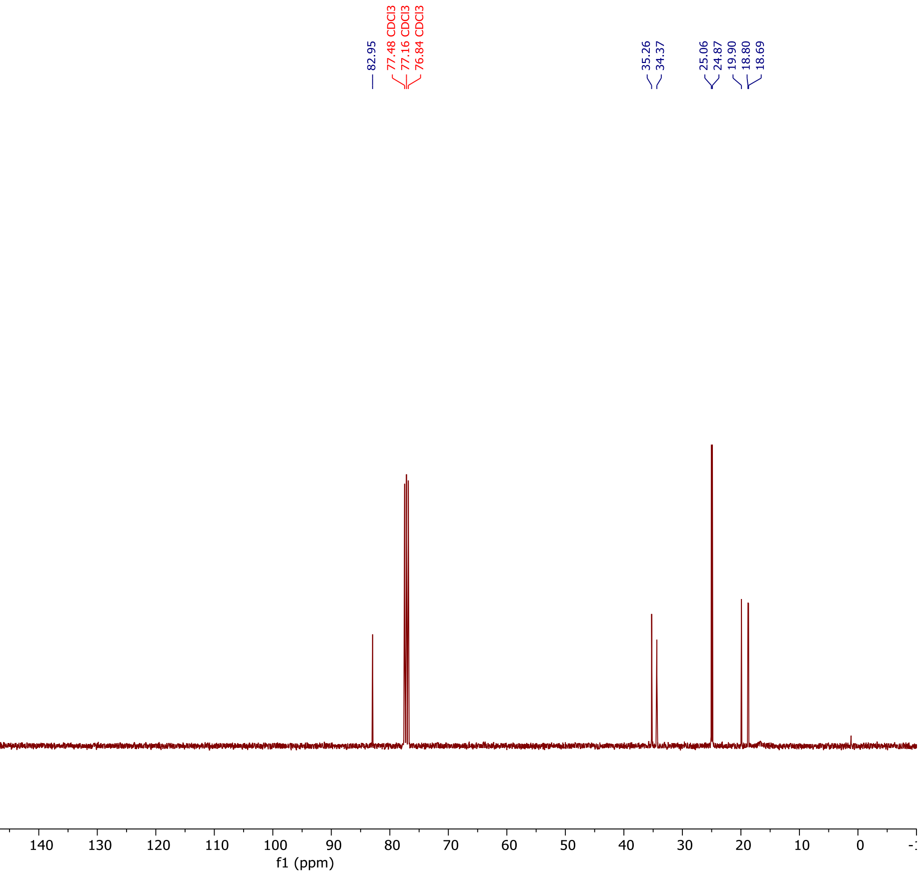

^{1}H NMR (400 MHz, $CDCl_3$) of **12** (***see procedure***)

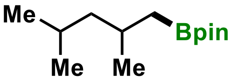

12

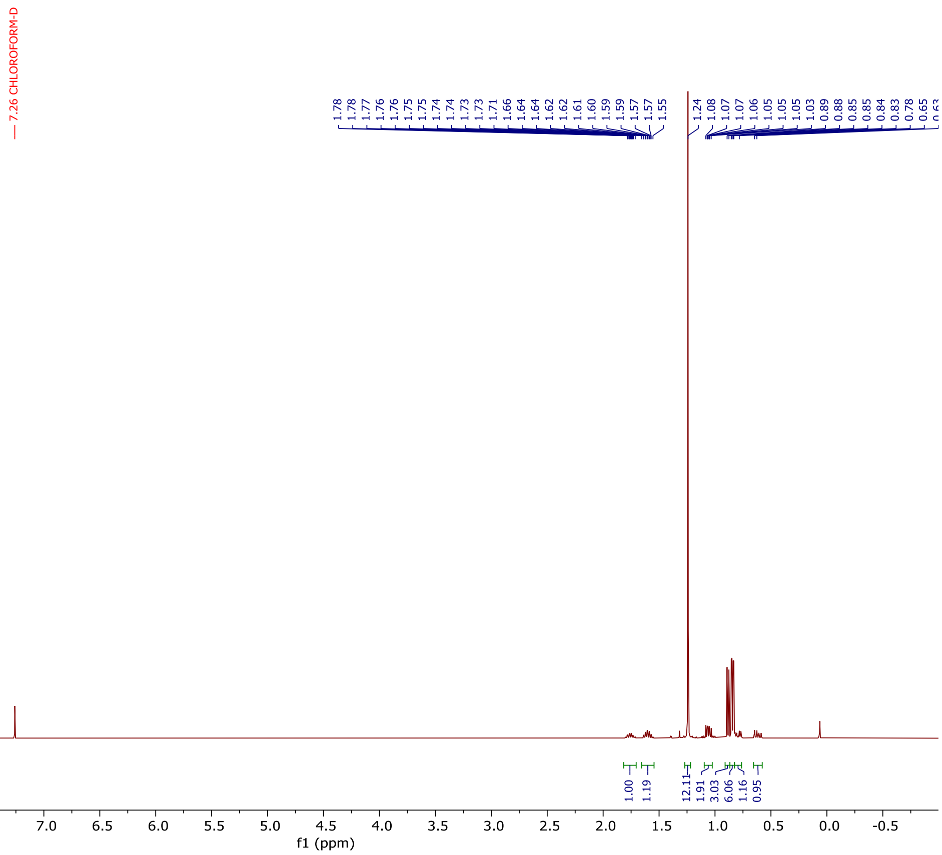

^{13}C NMR (101 MHz, $CDCl_3$) of **12**

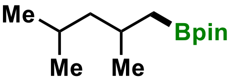

12

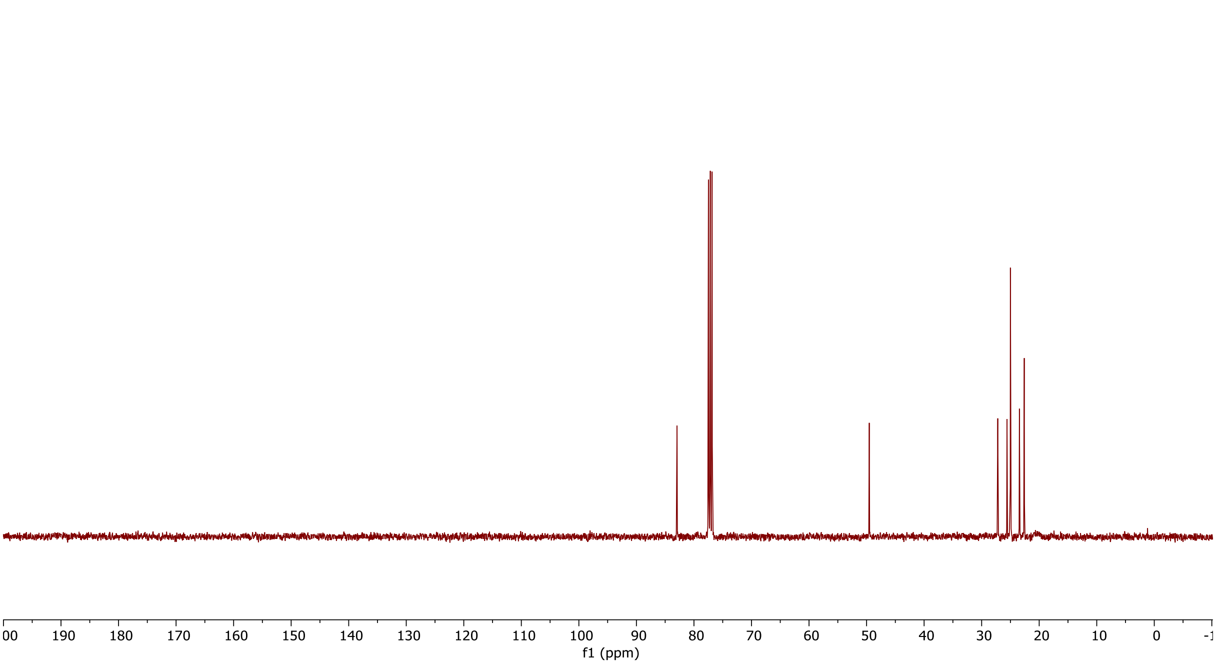

^{1}H NMR (400 MHz, $CDCl_3$) of **13a** and **13b** (***see procedure***)

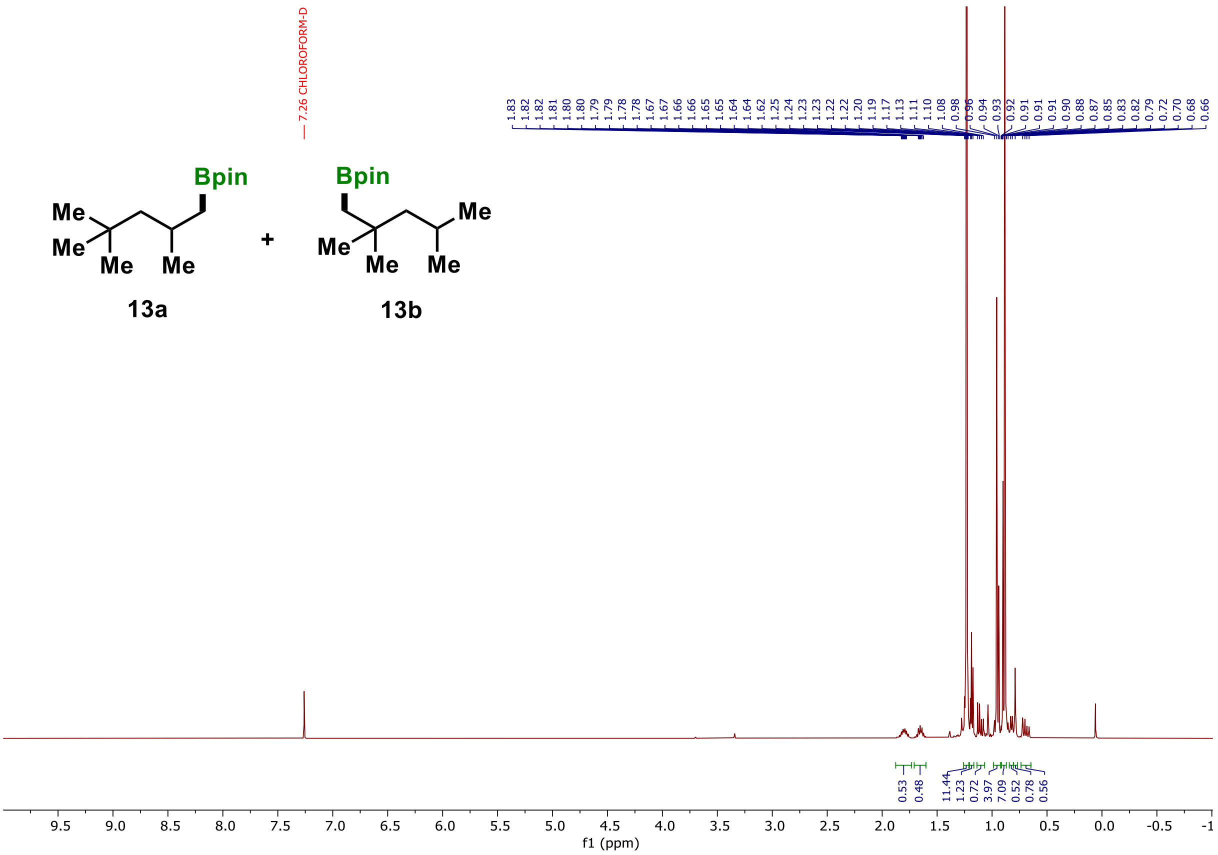

^{13}C NMR (101 MHz, $CDCl_3$) of **13a** and **13b**

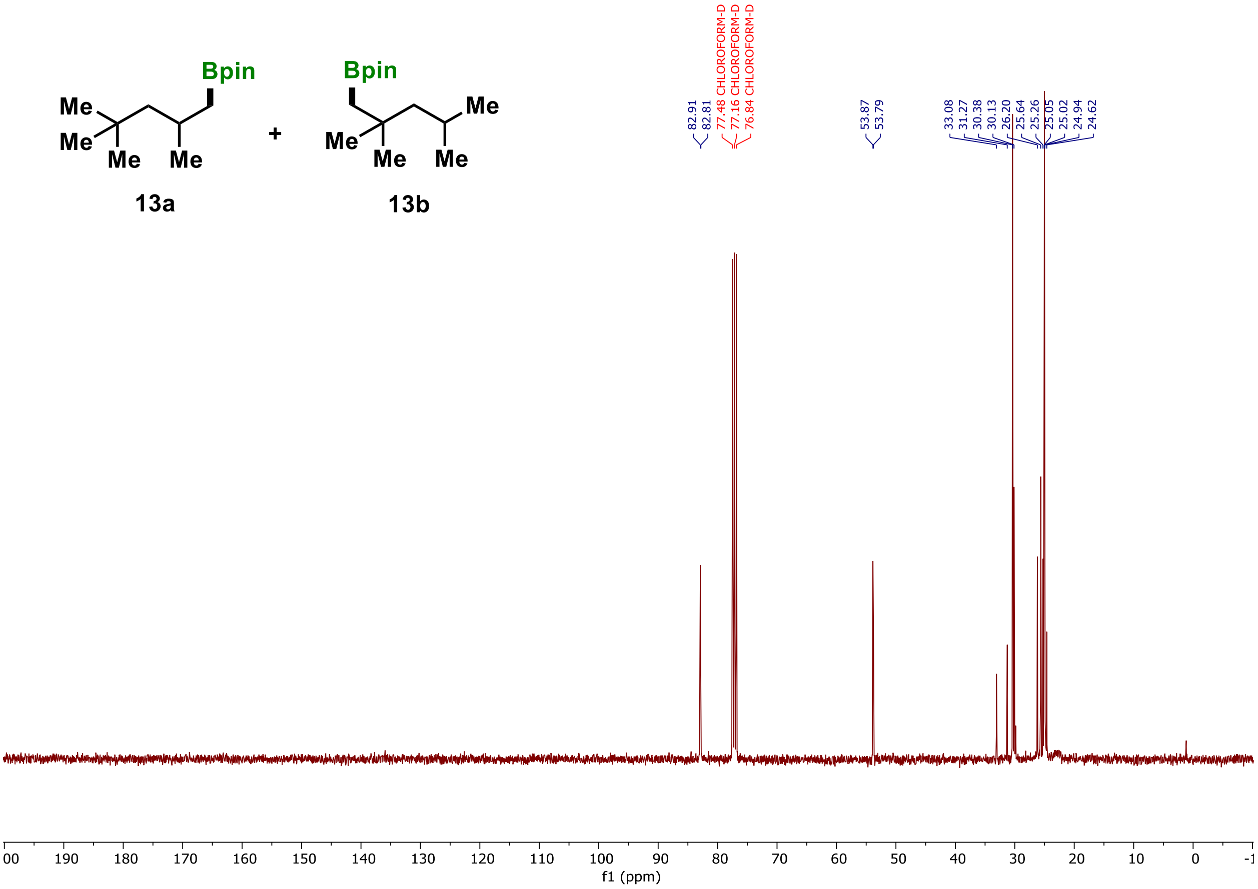

^{1}H NMR (400 MHz, $CDCl_3$) of **14** (***see procedure***)

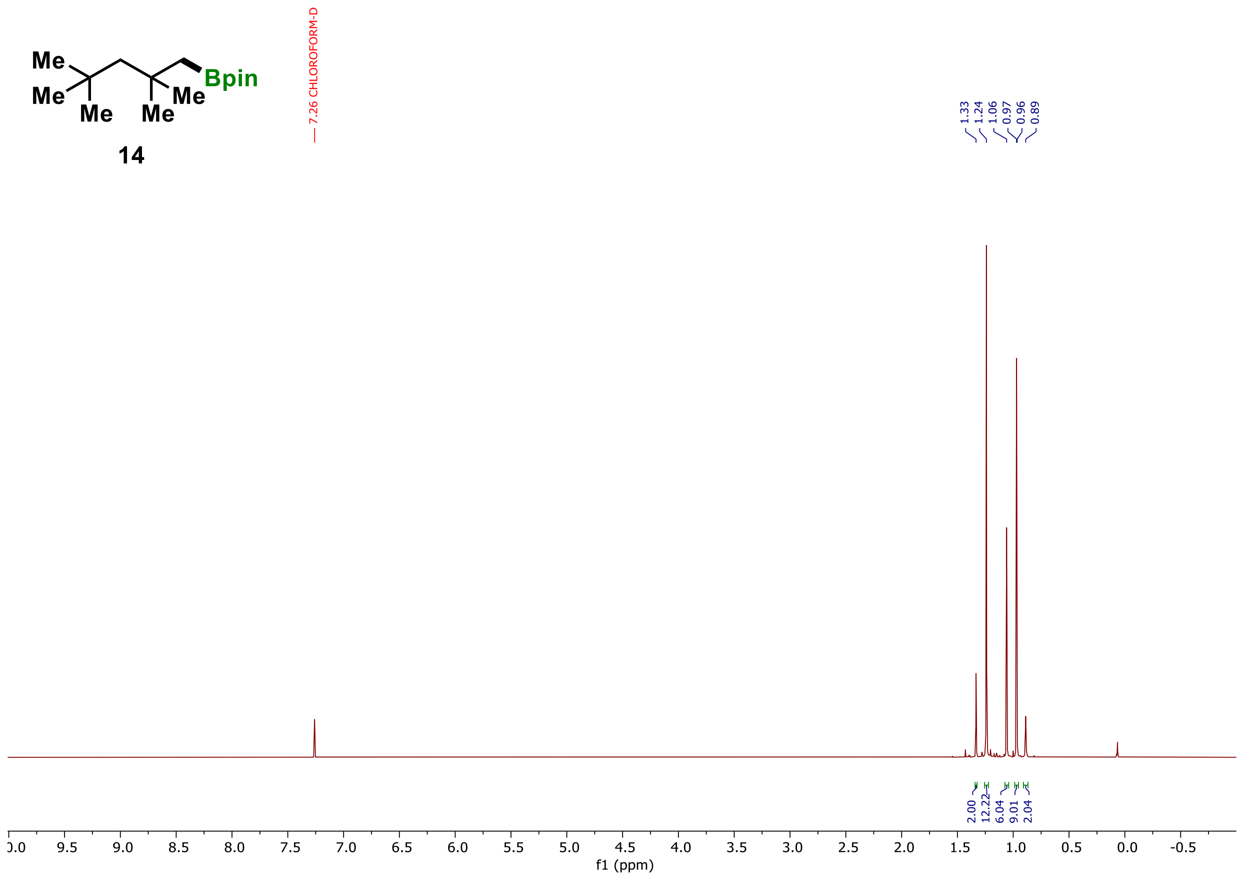

^{13}C NMR (101 MHz, $CDCl_3$) of **14**

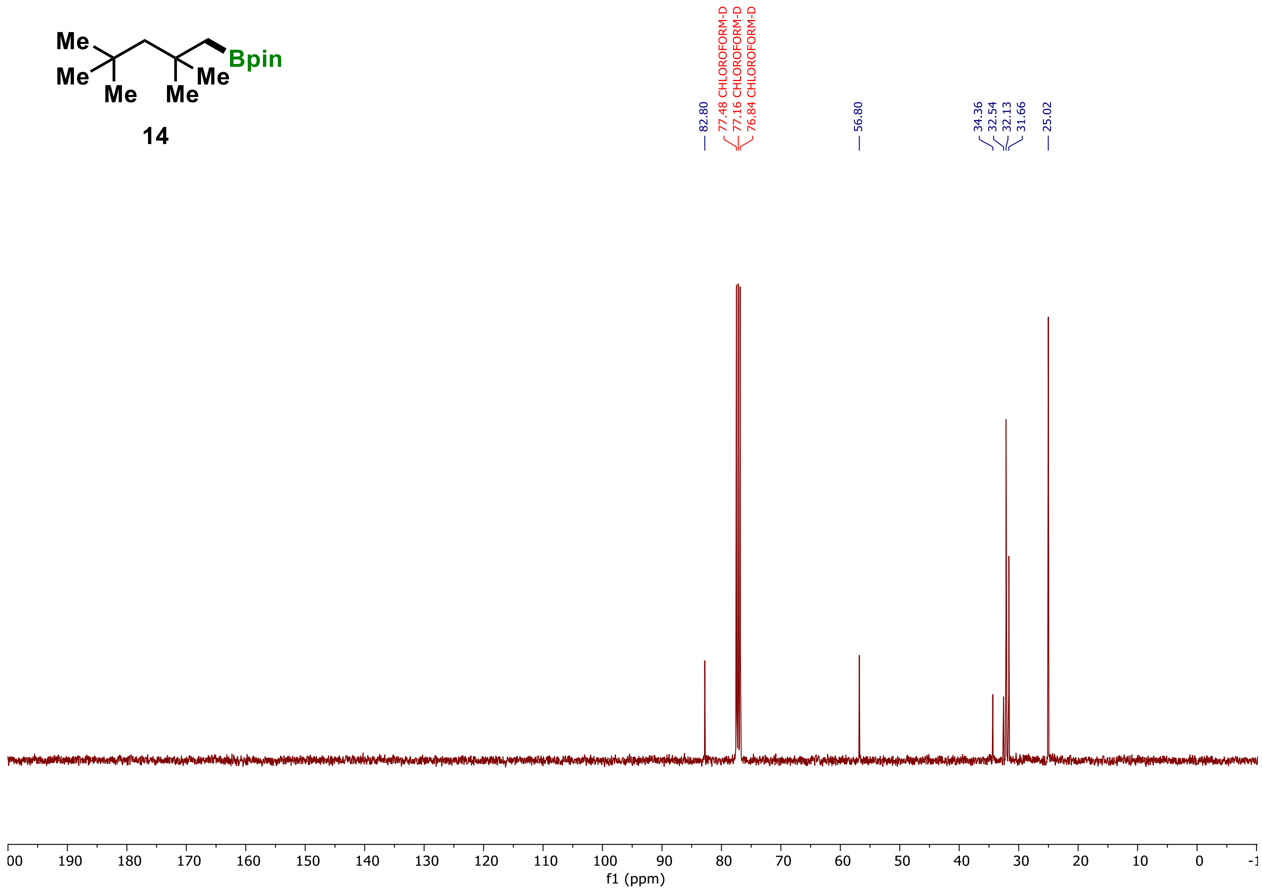

^{1}H NMR (400 MHz, $CDCl_3$) of **15** (*see procedure*)

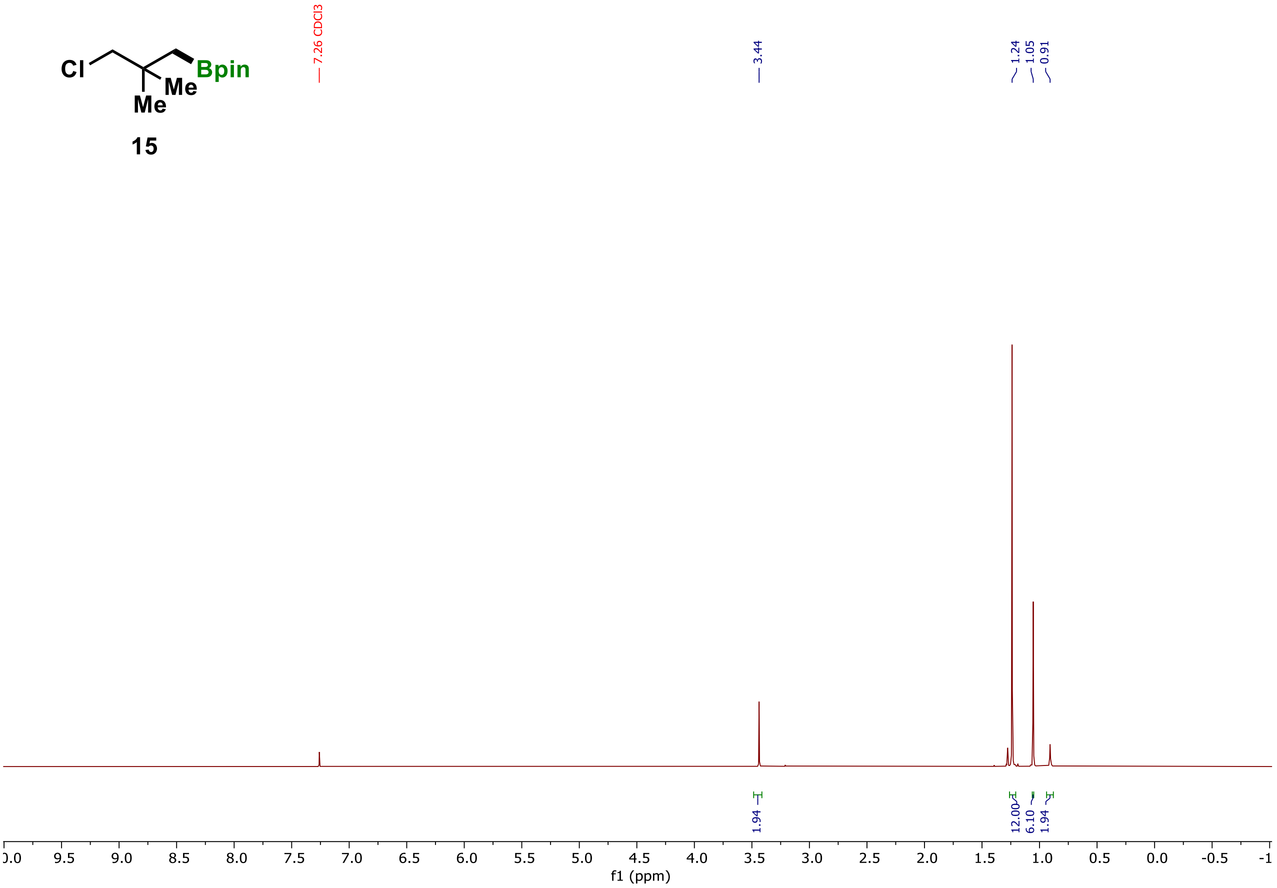

^{13}C NMR (101 MHz, $CDCl_3$) of **15**

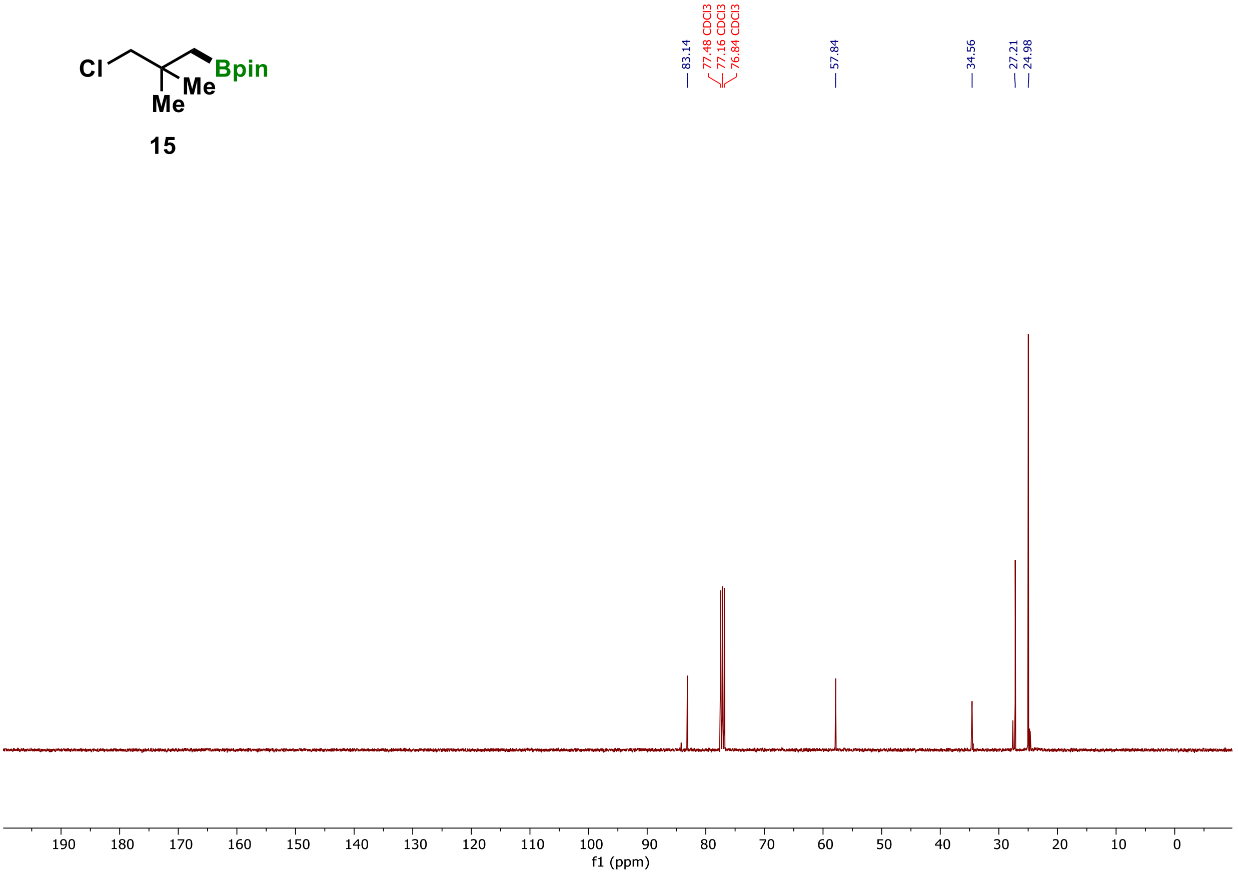

^{1}H NMR (400 MHz, $CDCl_3$) of **16** (***see procedure***)

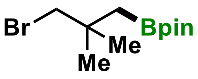

16

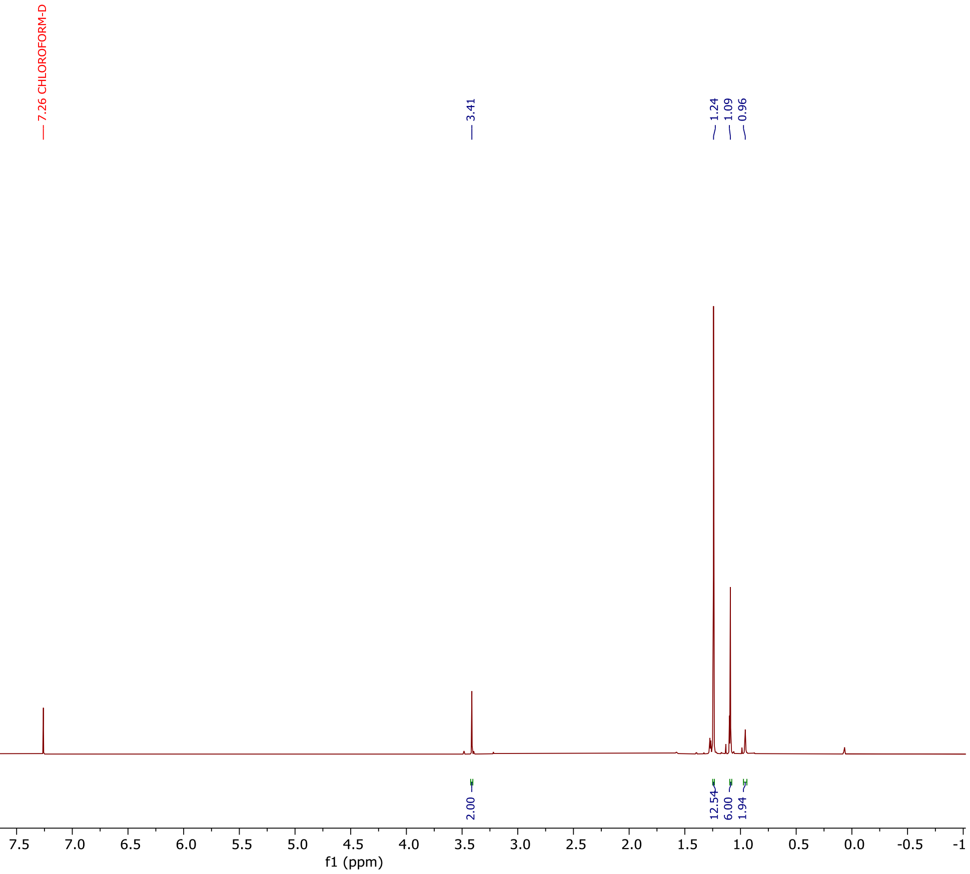

^{13}C NMR (101 MHz, $CDCl_3$) of **16**

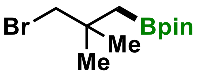

16

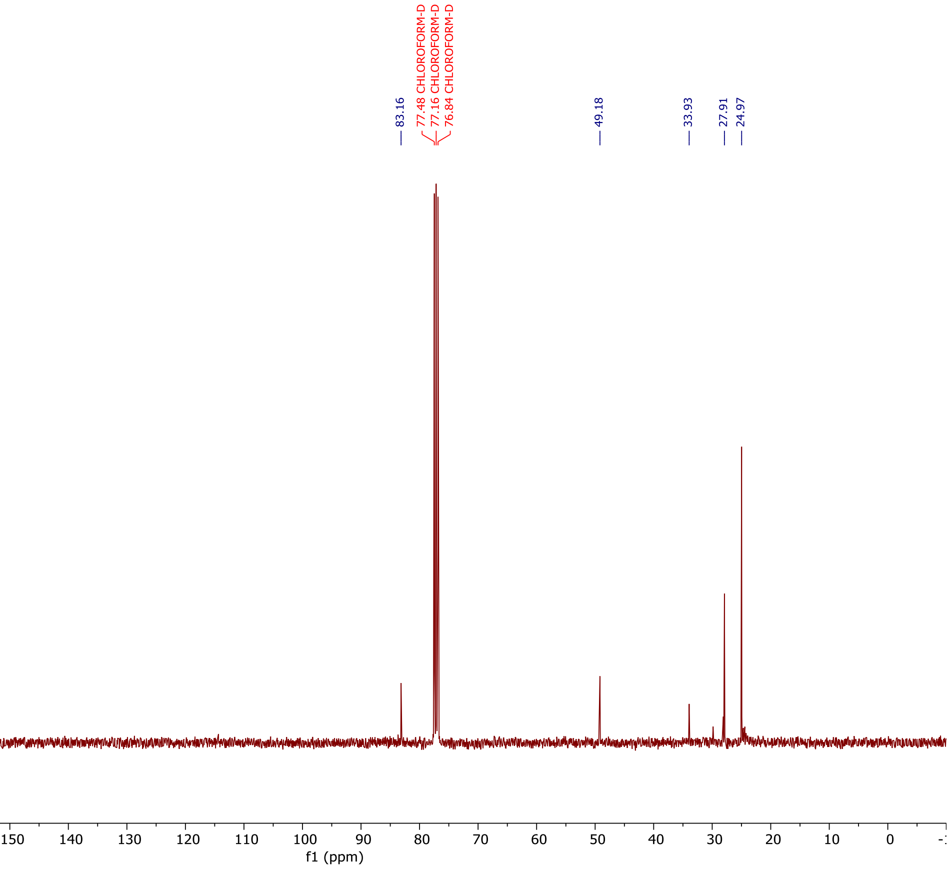

^{1}H NMR (400 MHz, $CDCl_3$) of **17** (***see procedure***)

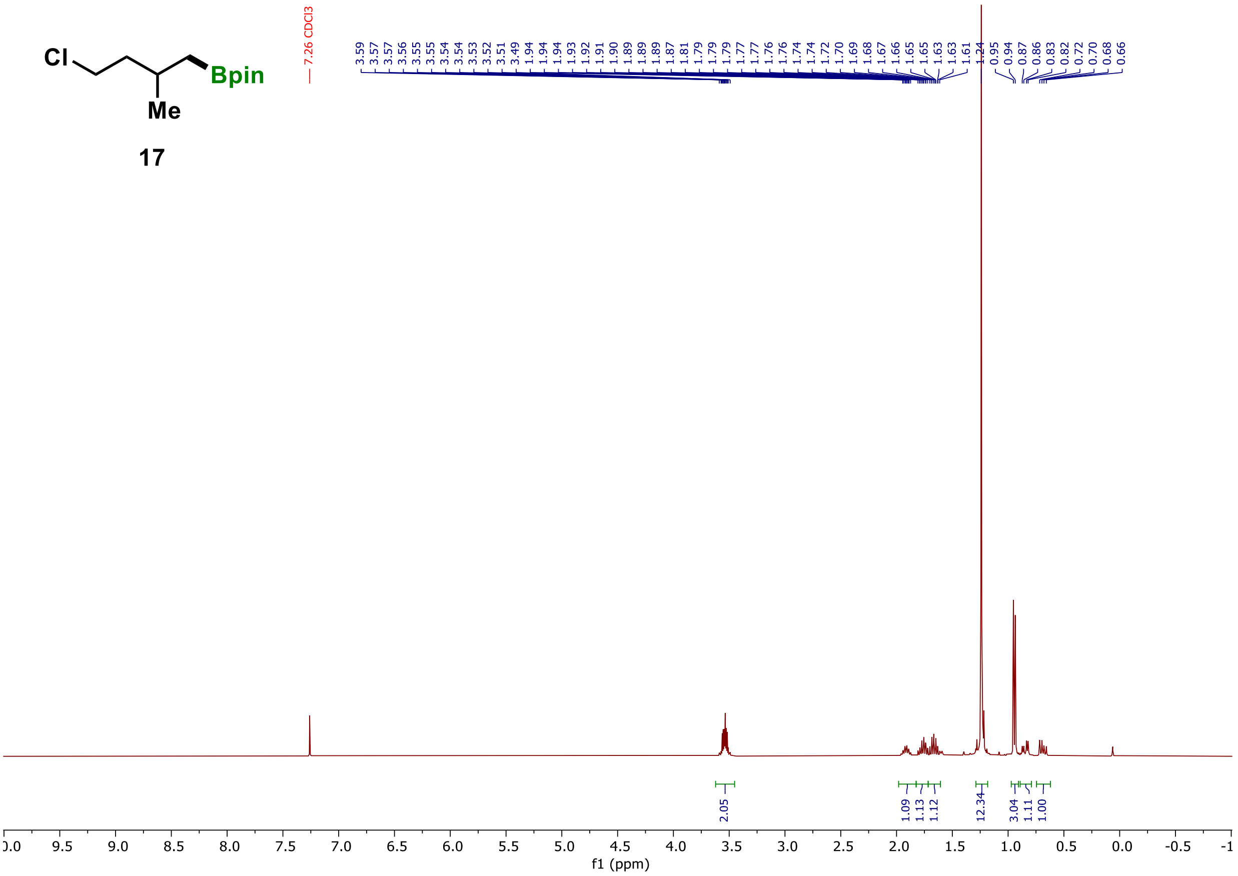

^{13}C NMR (101 MHz, $CDCl_3$) of **17**

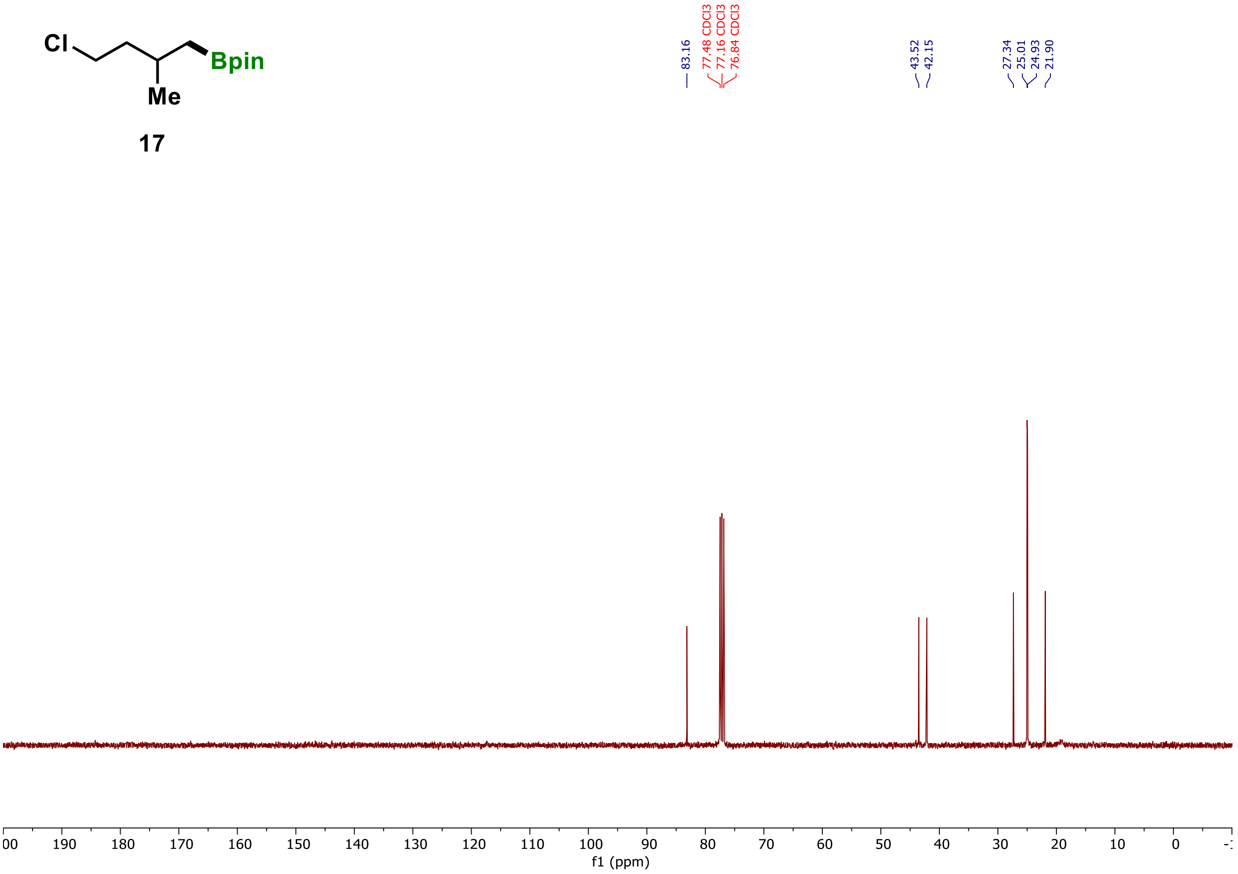

^{11}B NMR (128 MHz, $CDCl_3$) of **17**

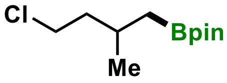

17

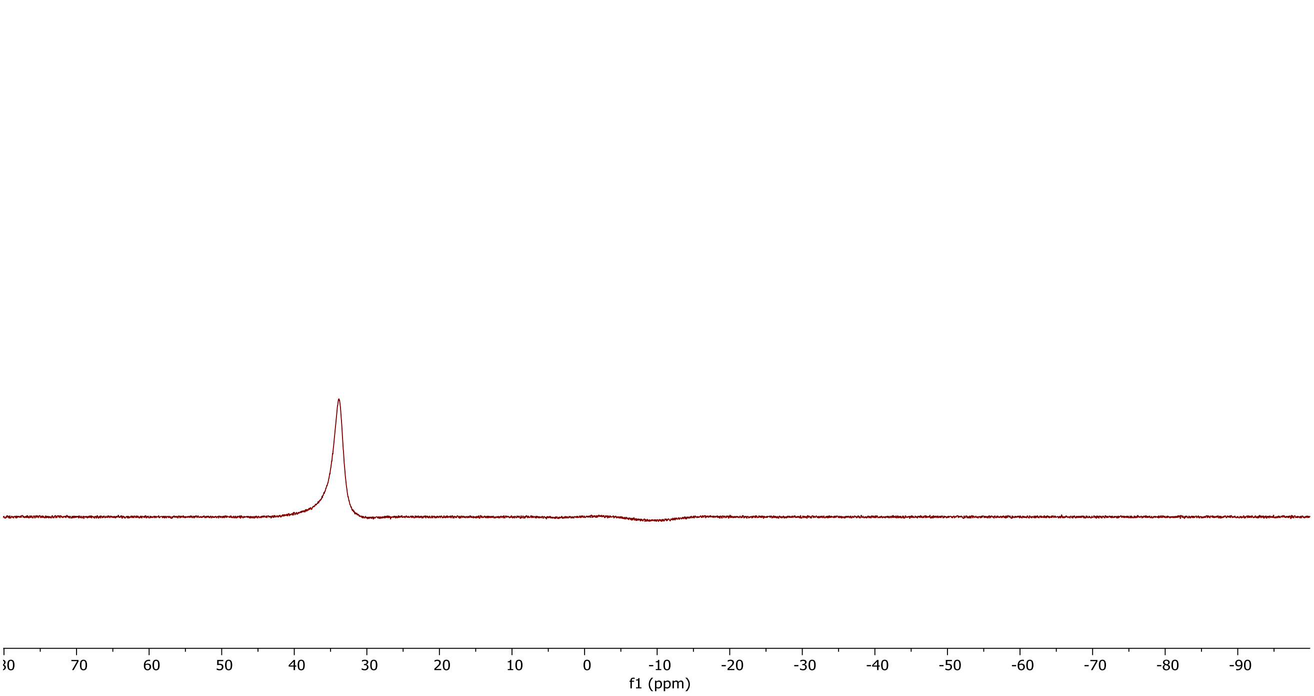

^{1}H NMR (400 MHz, $CDCl_3$) of **18** (*see procedure*)

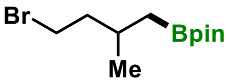

18

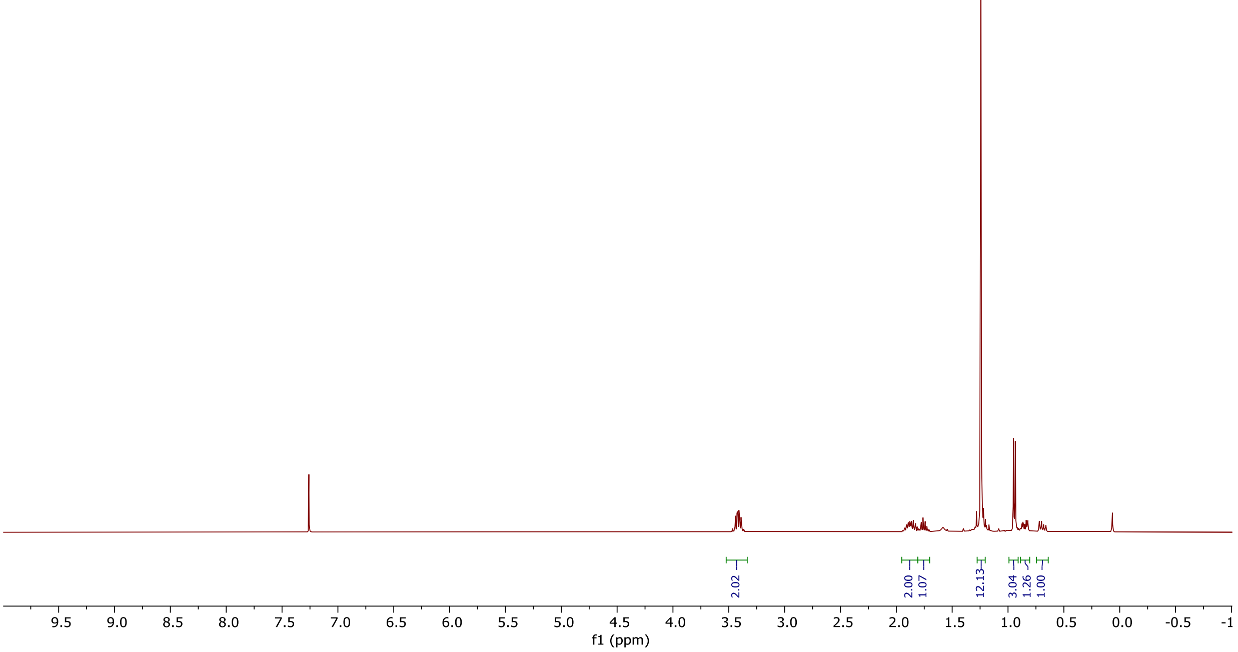

^{13}C NMR (101 MHz, $CDCl_3$) of **18**

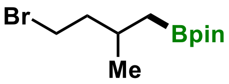

18

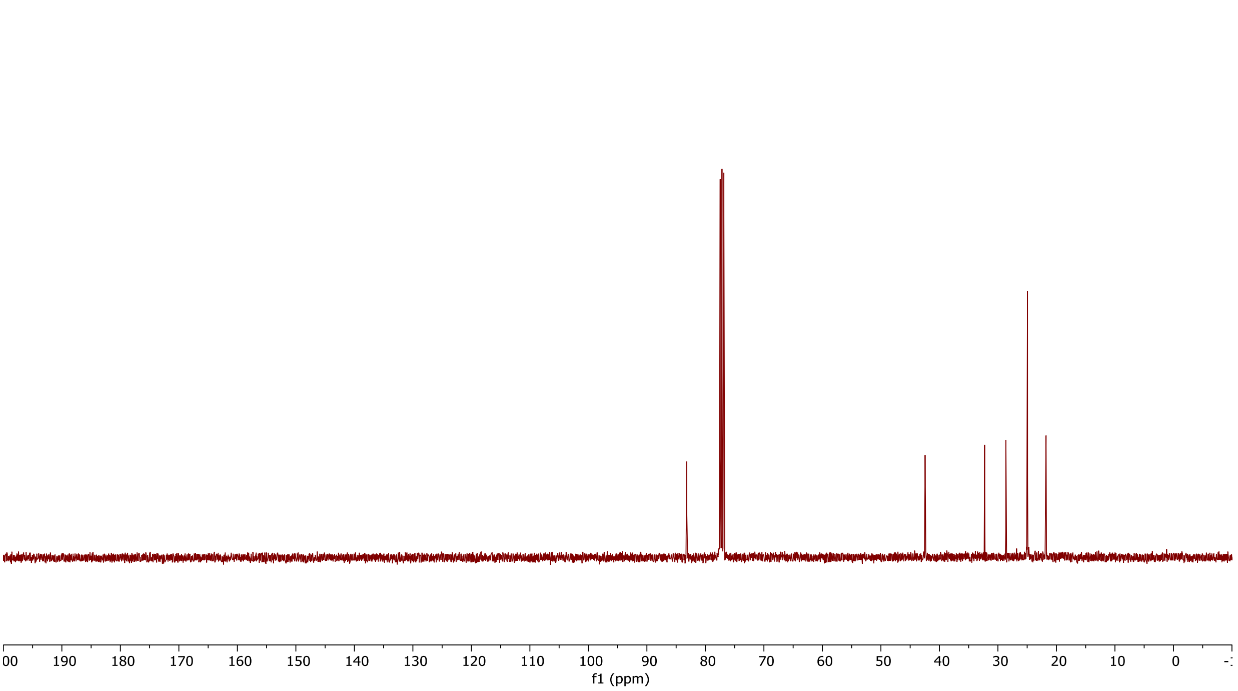

^{11}B NMR (128 MHz, $CDCl_3$) of **18**

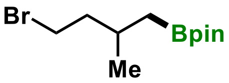

18

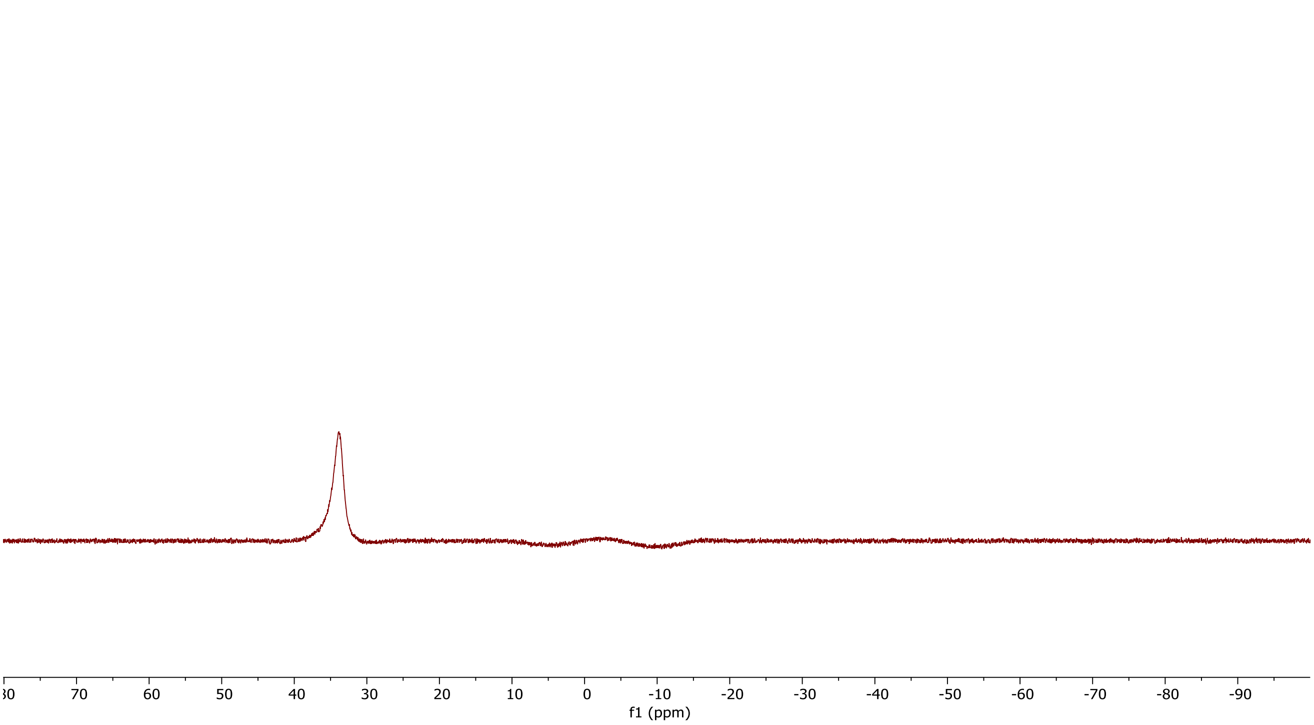

^{1}H NMR (400 MHz, $CDCl_3$) of **19** (*see procedure*)

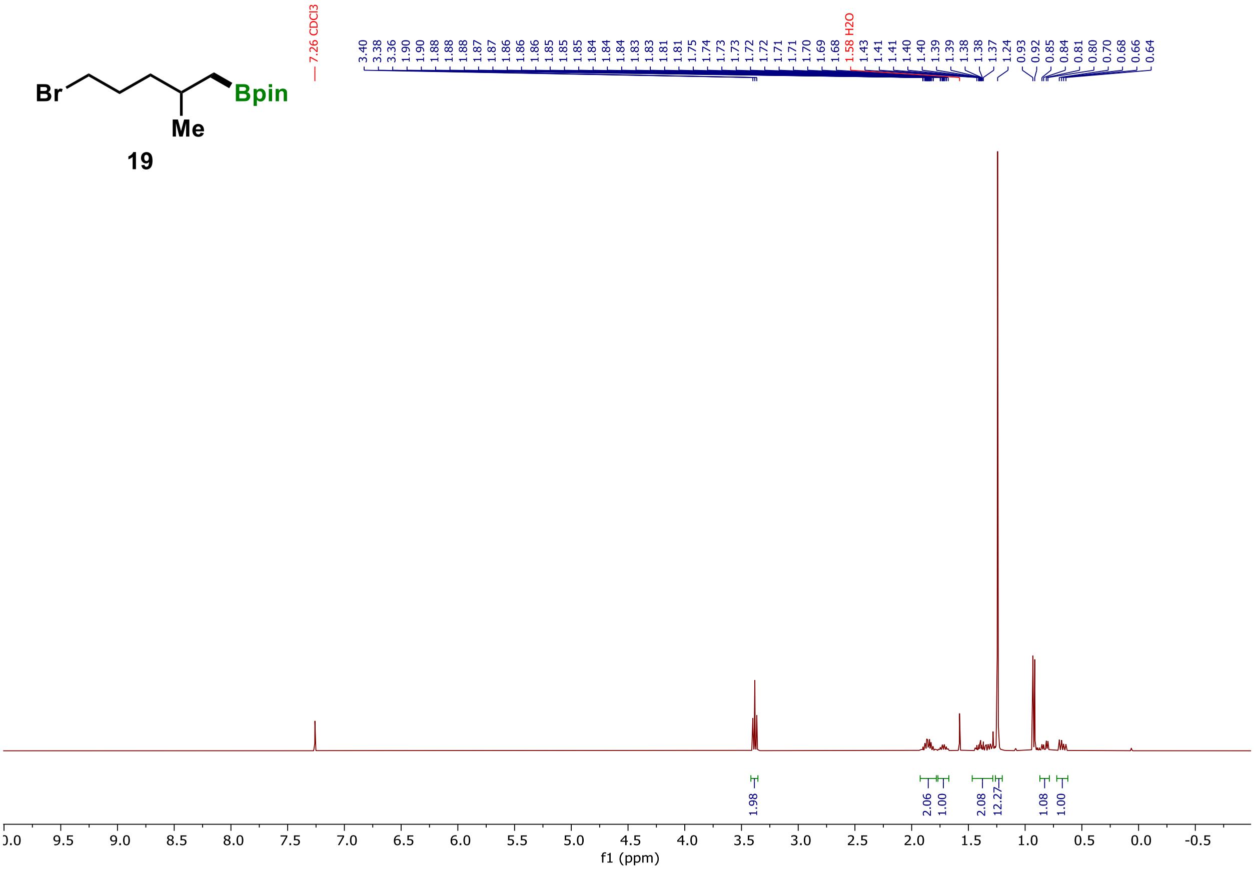

^{13}C NMR (101 MHz, $CDCl_3$) of **19**

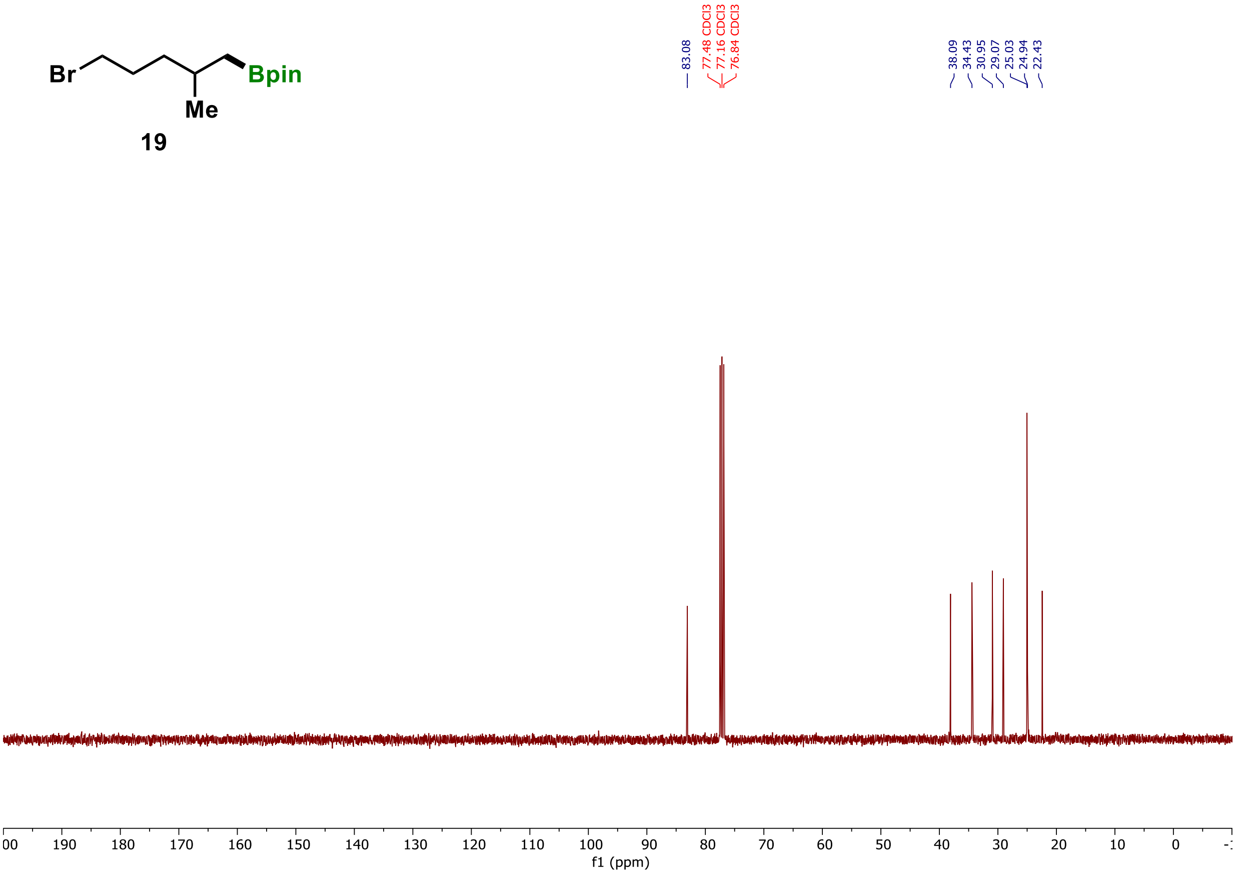

^{1}H NMR (400 MHz, $CDCl_3$) of **20a** (*see procedure*)

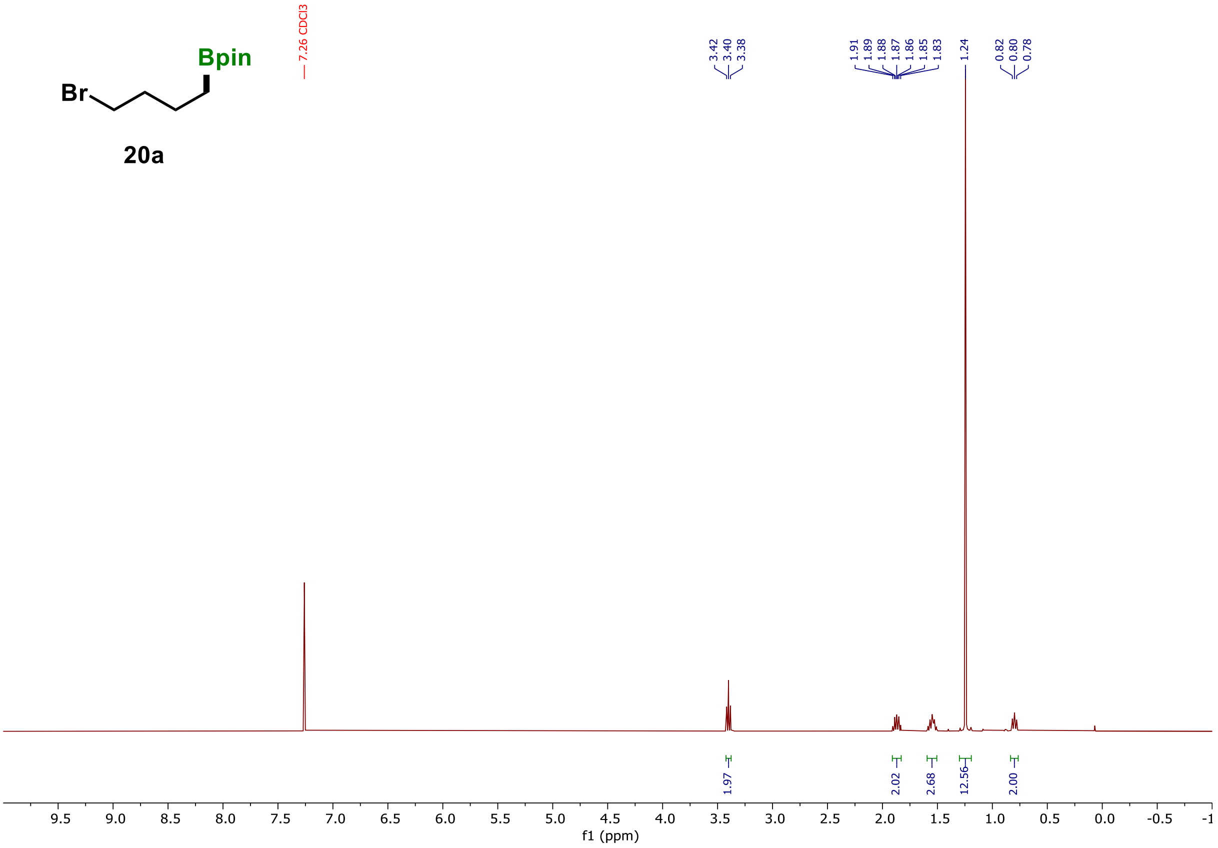

^{13}C NMR (101 MHz, $CDCl_3$) of **20a**

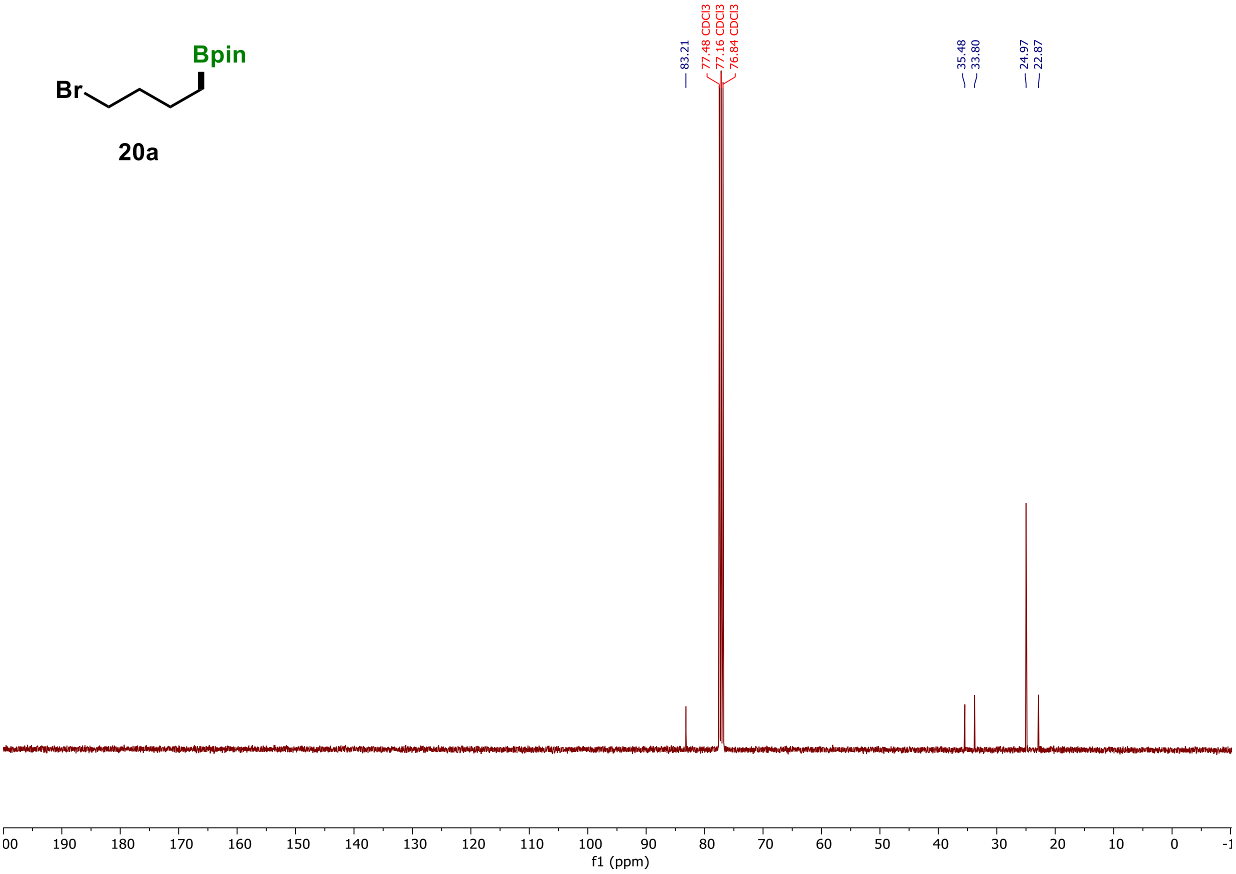

^{1}H NMR (400 MHz, $CDCl_3$) of **20b** (*see procedure*)

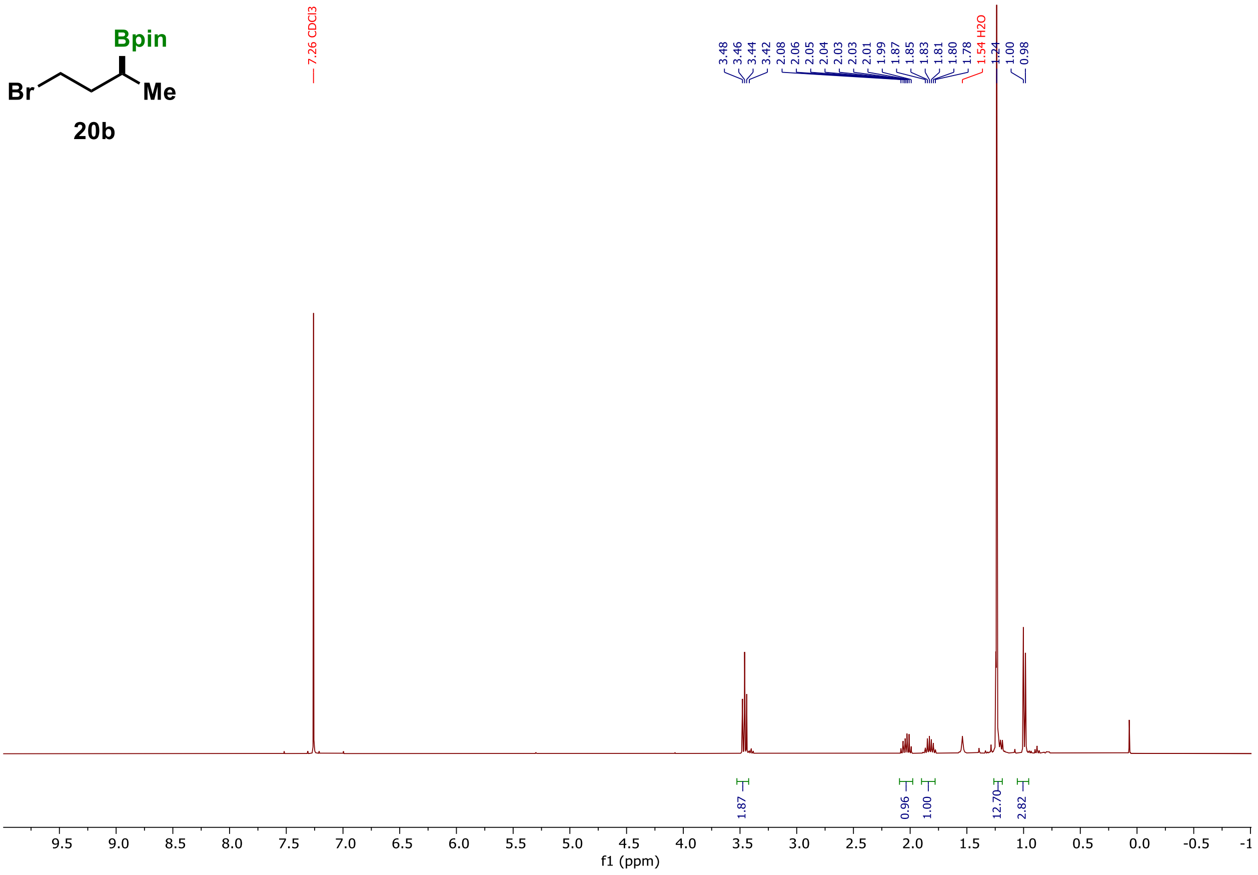

^{13}C NMR (101 MHz, $CDCl_3$) of **20b**

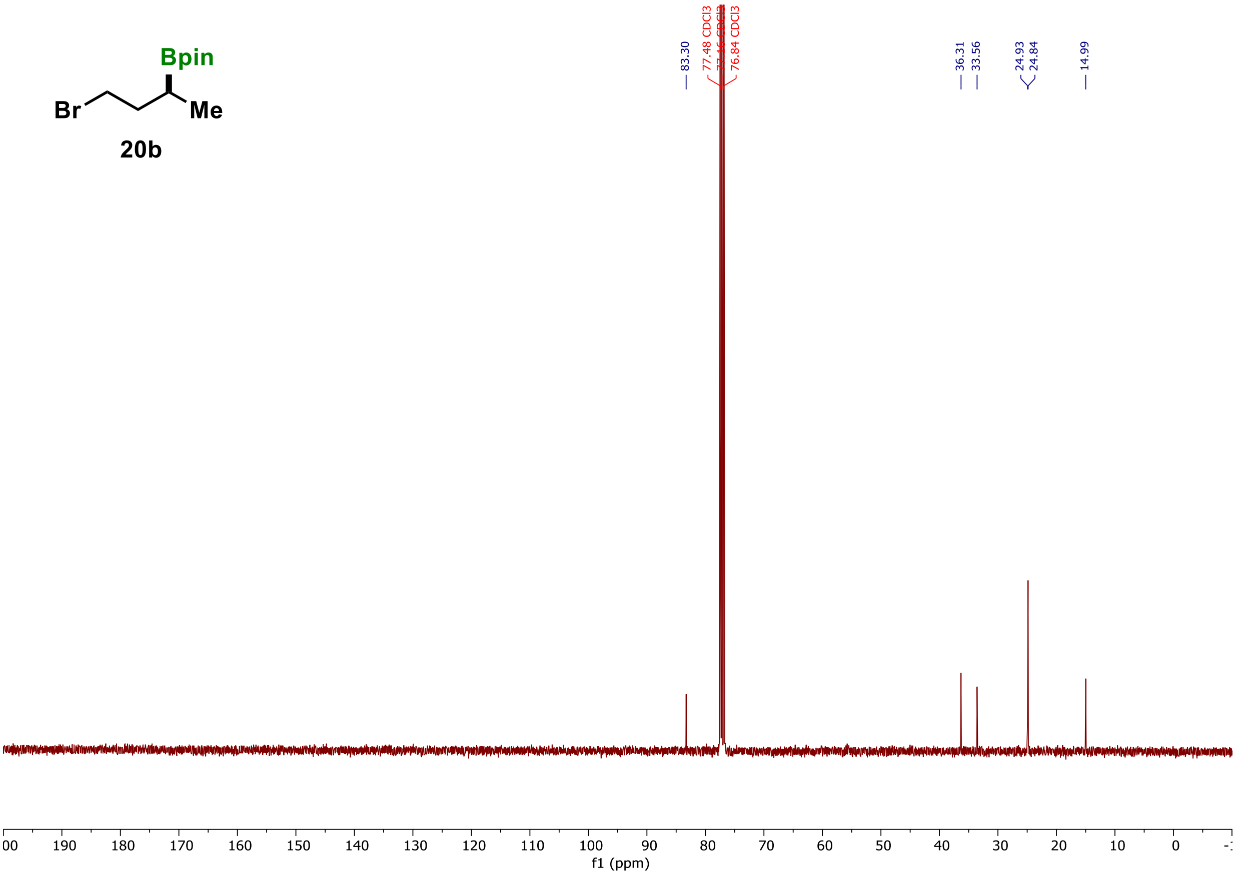

^{11}B NMR (128 MHz, $CDCl_3$) of **20b**

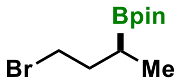

20b

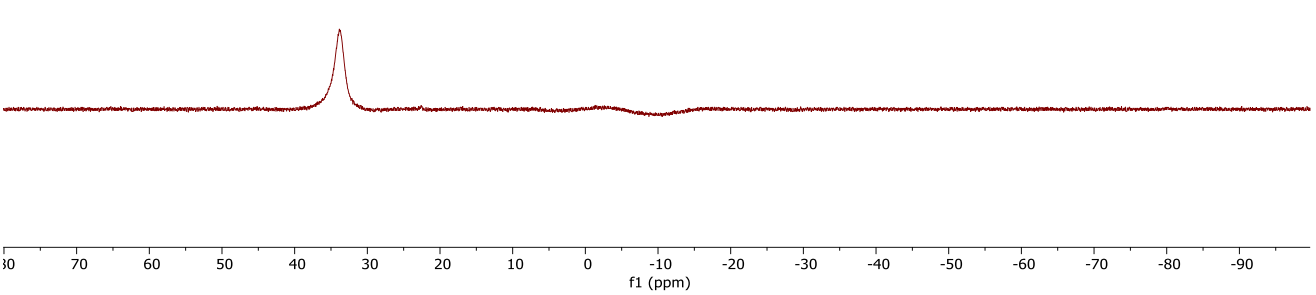

^{1}H NMR (400 MHz, CDCl3) of **21a** and **21b** (*see procedure*)

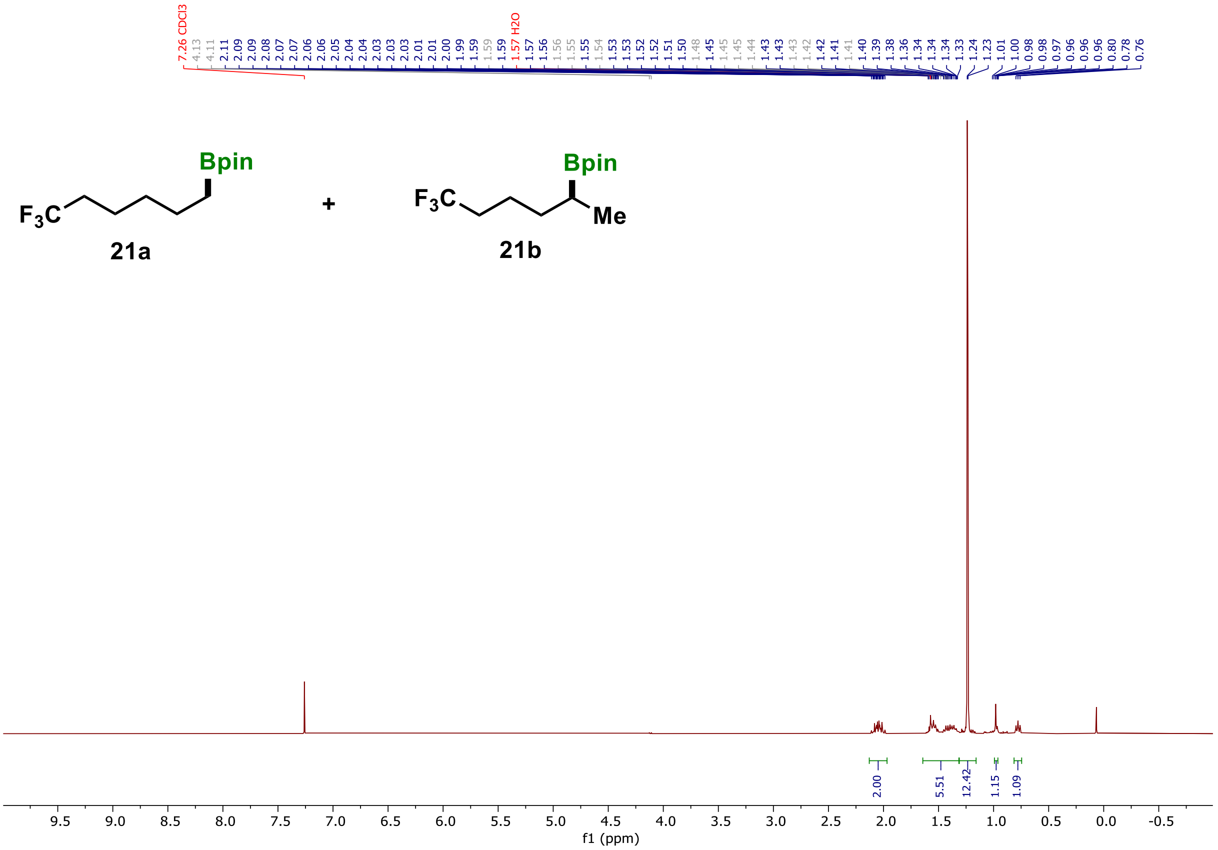

21a + **21b**

^{13}C NMR (101 MHz, CDCl3) of **21a** and **21b**

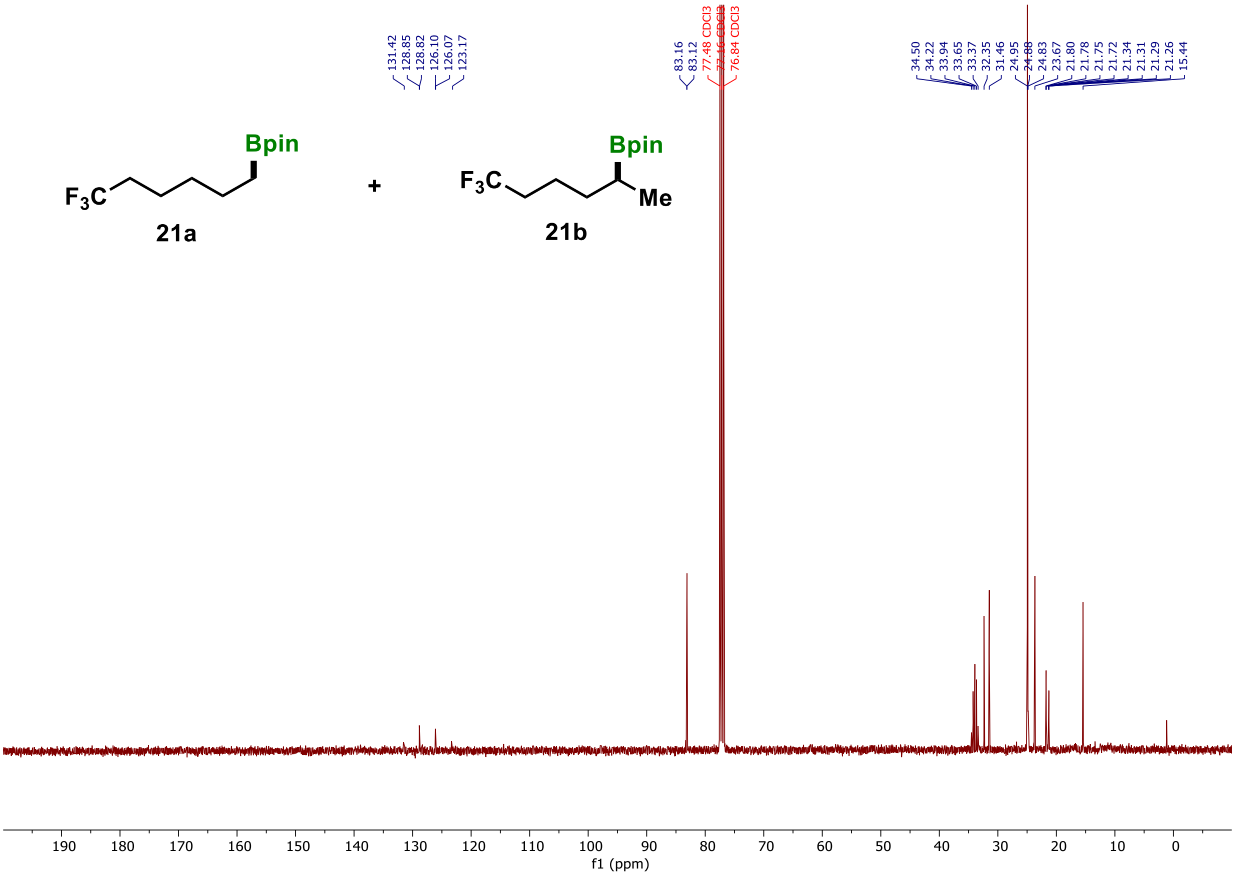

21a + **21b**

^{19}F NMR (377 MHz, $CDCl_3$) of **21a** and **21b**

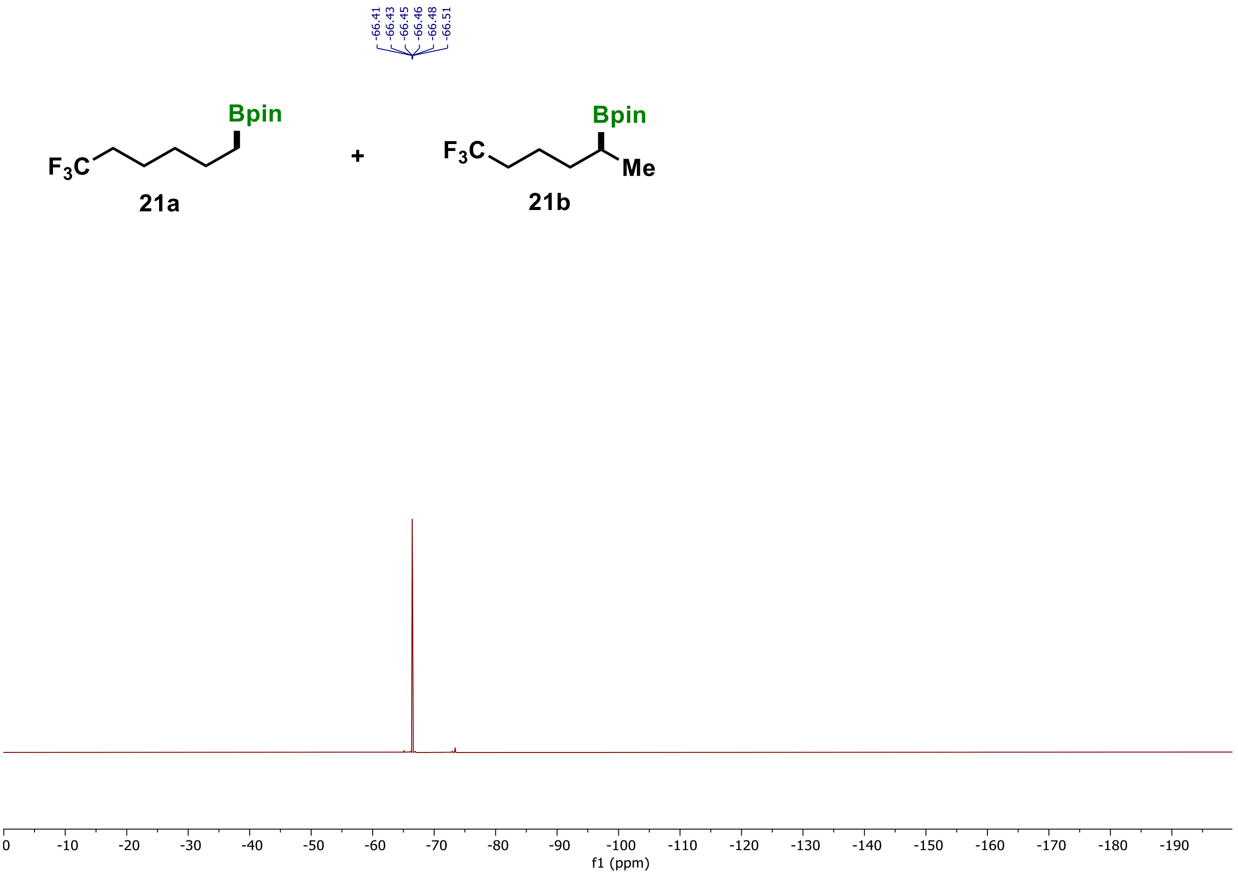

^{11}B NMR (128 MHz, $CDCl_3$) of **21a** and **21b**

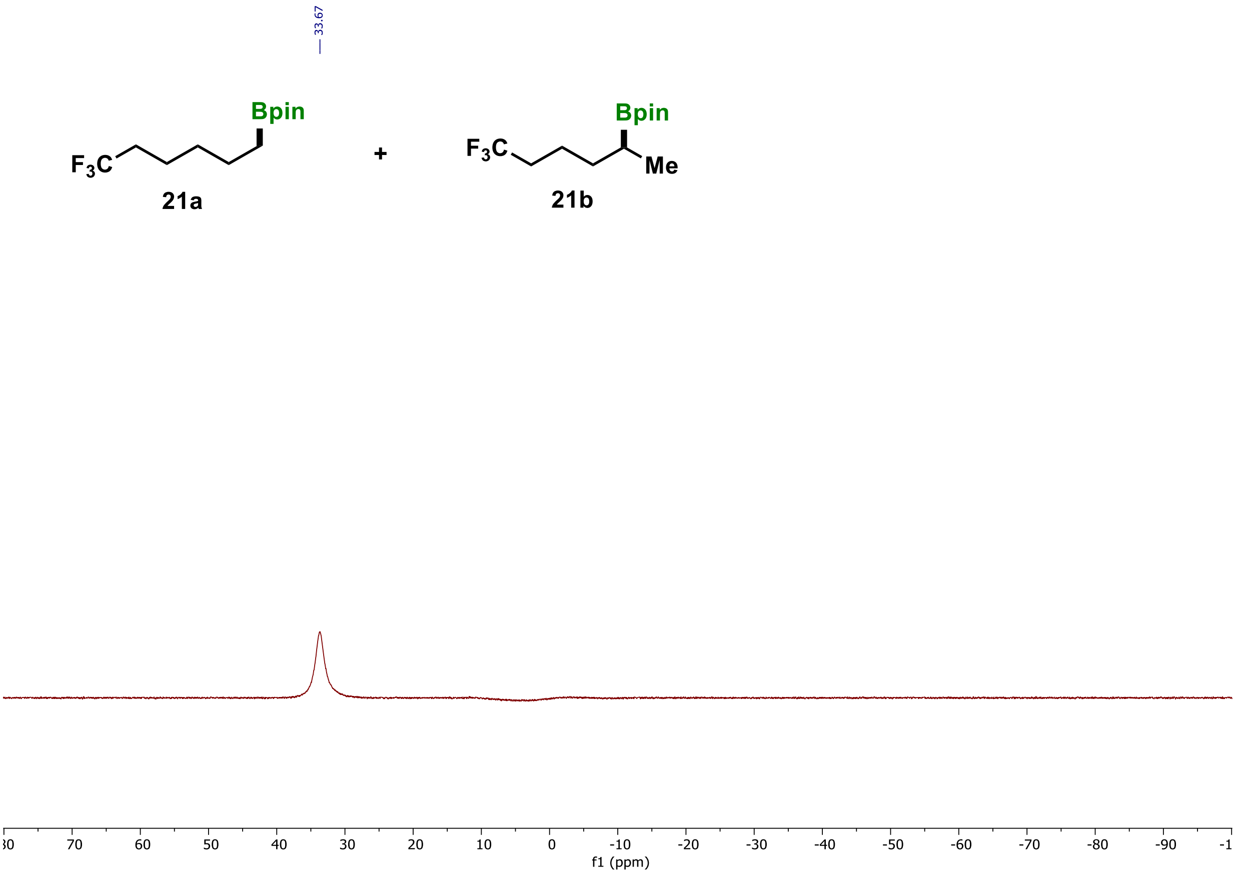

^{1}H NMR (400 MHz, $CDCl_3$) of **22** (***see procedure***)

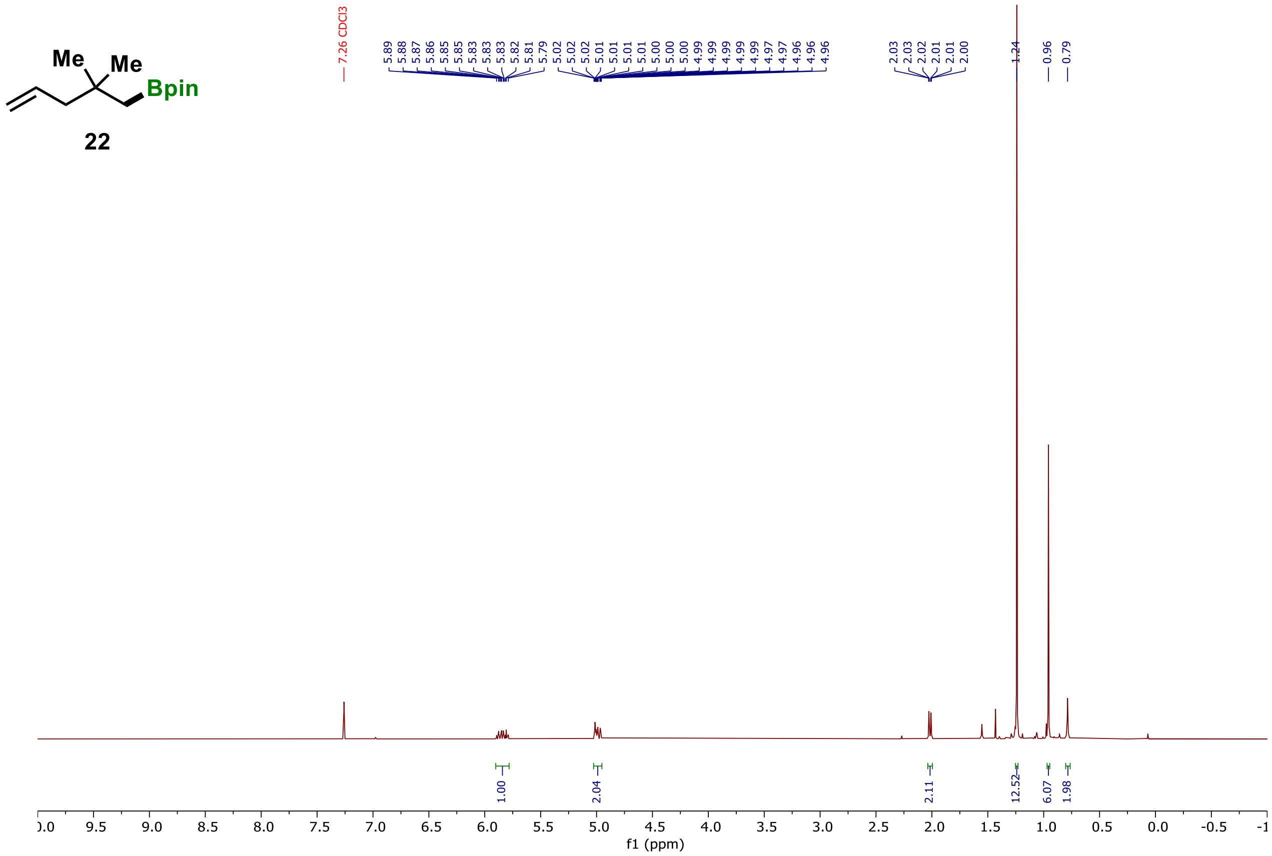

^{13}C NMR (101 MHz, $CDCl_3$) of **22**

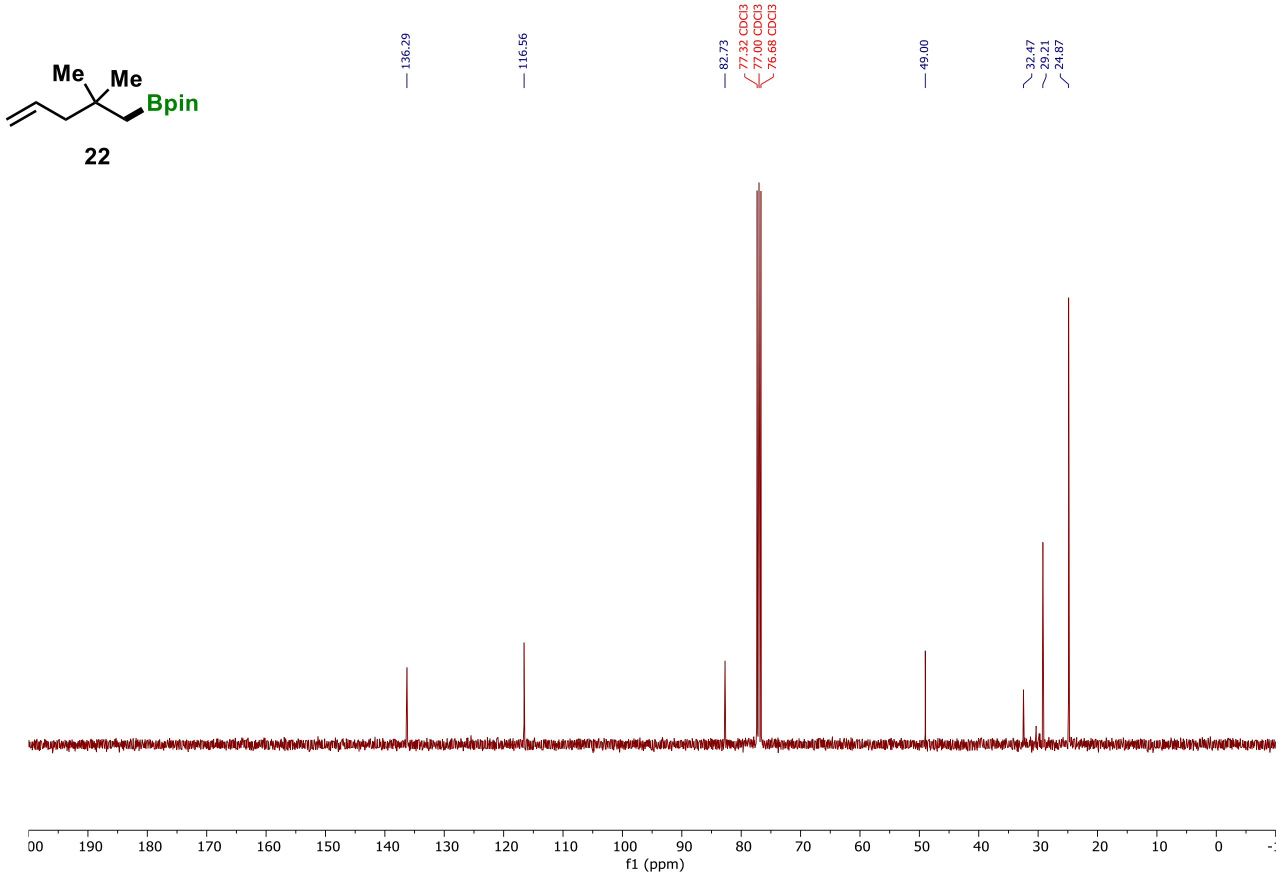

^{1}H NMR (400 MHz, $CDCl_3$) of **23** (*see procedure*)

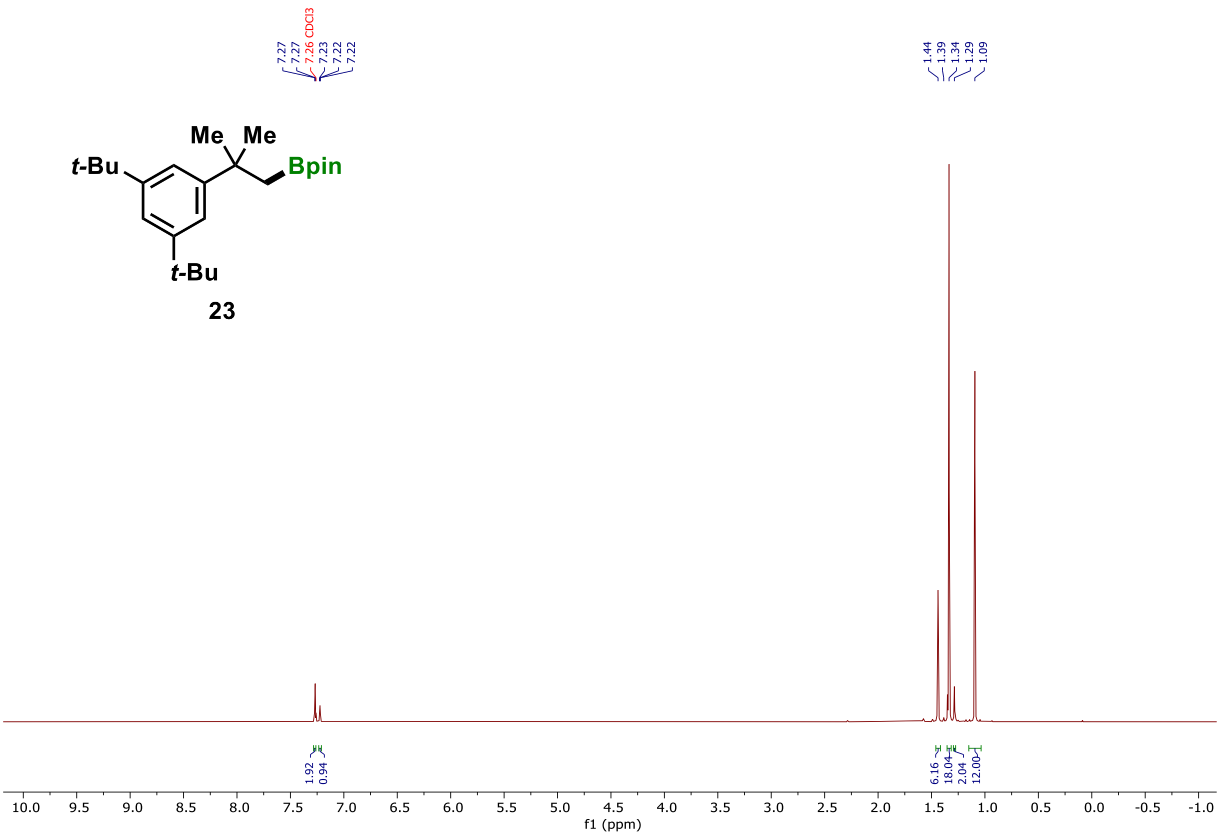

^{13}C NMR (101 MHz, $CDCl_3$) of **23**

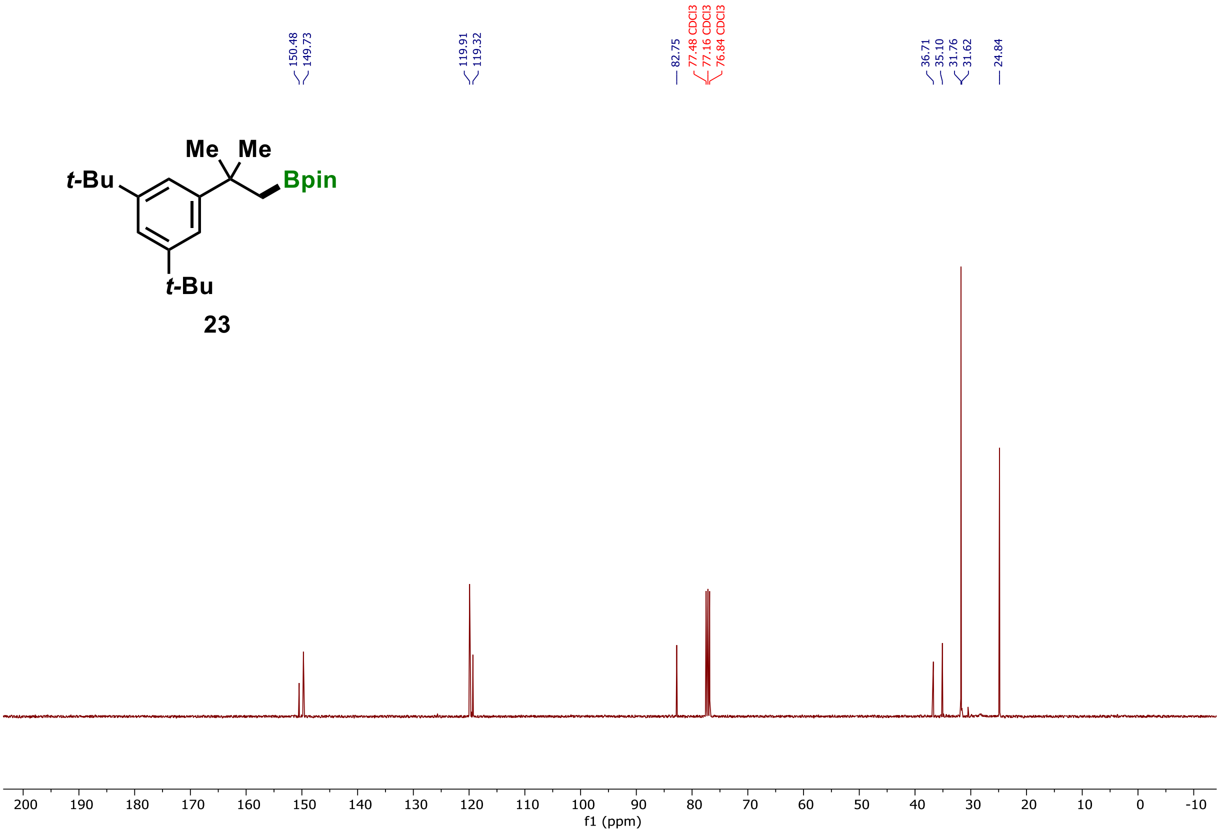

^{11}B NMR (128 MHz, $CDCl_3$) of **23**

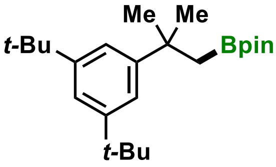
Me
Me
t-Bu
Bpin
t-Bu

23

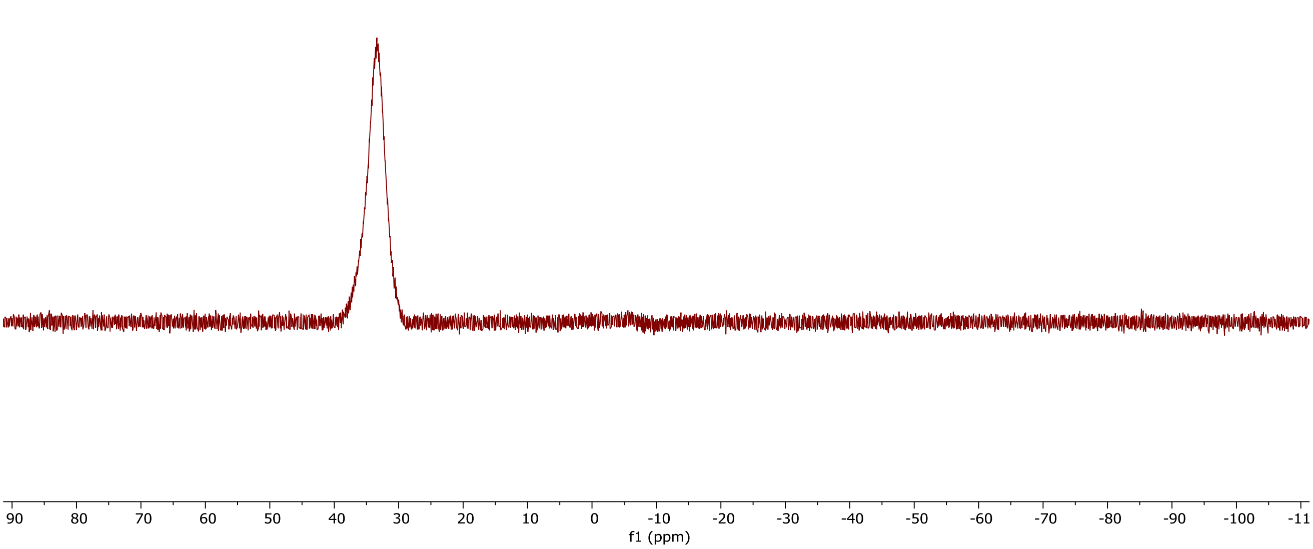
33.39
90
80
70
60
50
40
30
20
10
0
-10
-20
-30
-40
-50
-60
-70
-80
-90
-100
f1 (ppm)

^{1}H NMR (400 MHz, $CDCl_3$) of **24** (***see procedure***)

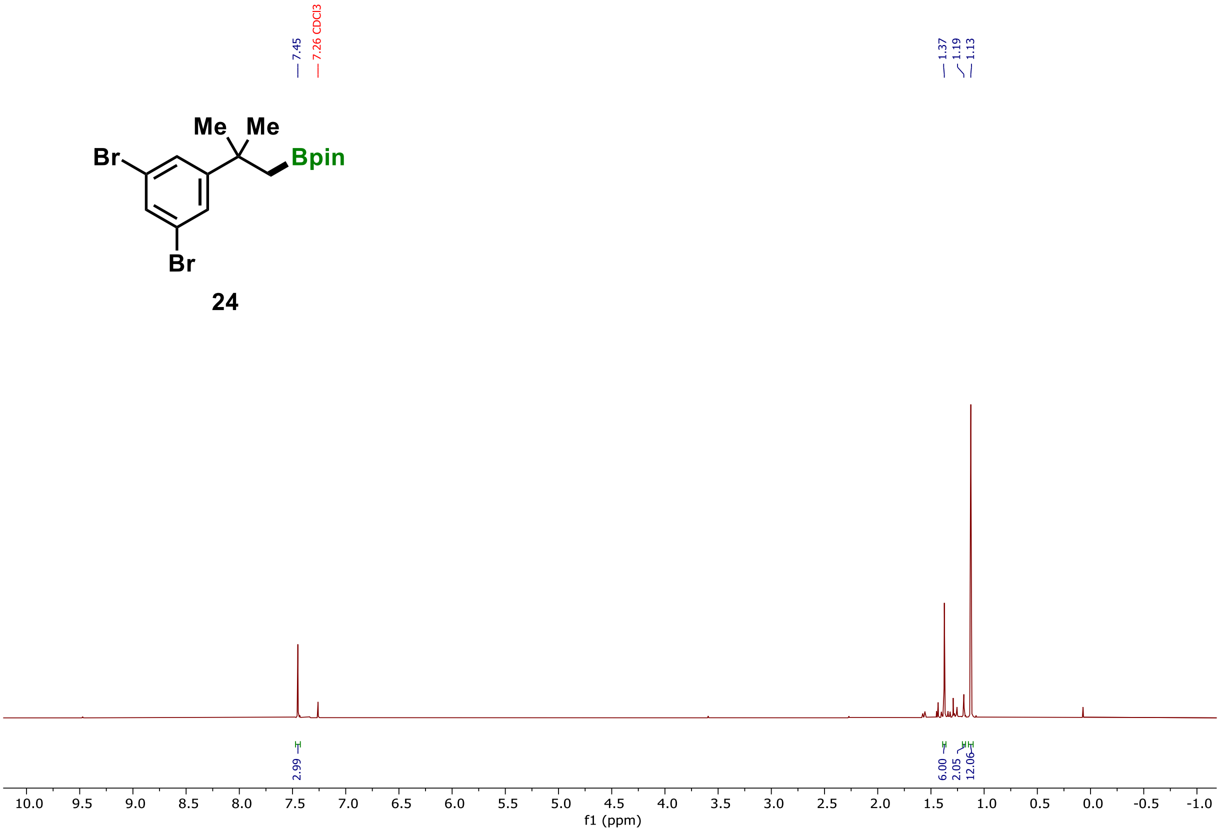

^{13}C NMR (101 MHz, $CDCl_3$) of **24**

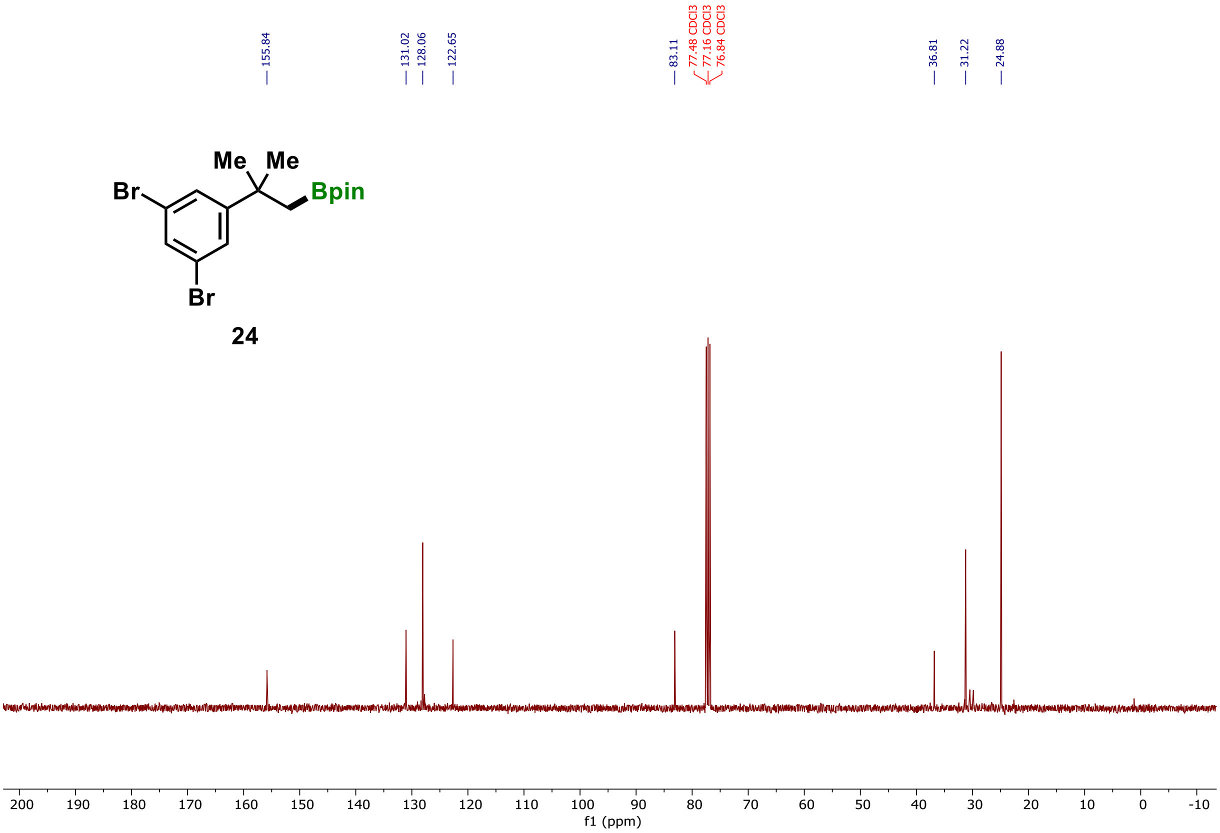

^{11}B NMR (128 MHz, $CDCl_3$) of **24**

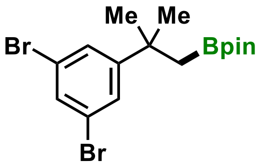

24

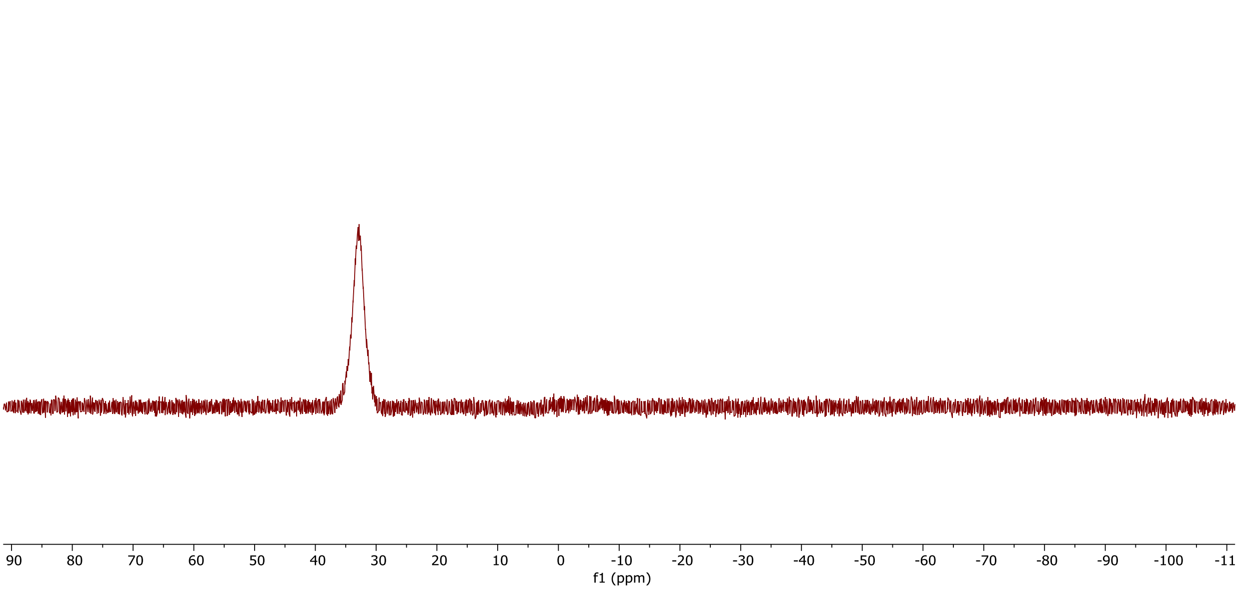
32.89
90
80
70
60
50
40
30
20
10
0
-10
-20
-30
-40
-50
-60
-70
-80
-90
-100
-11
f1 (ppm)

^{1}H NMR (400 MHz, $CDCl_3$) of **25** (*see procedure*)

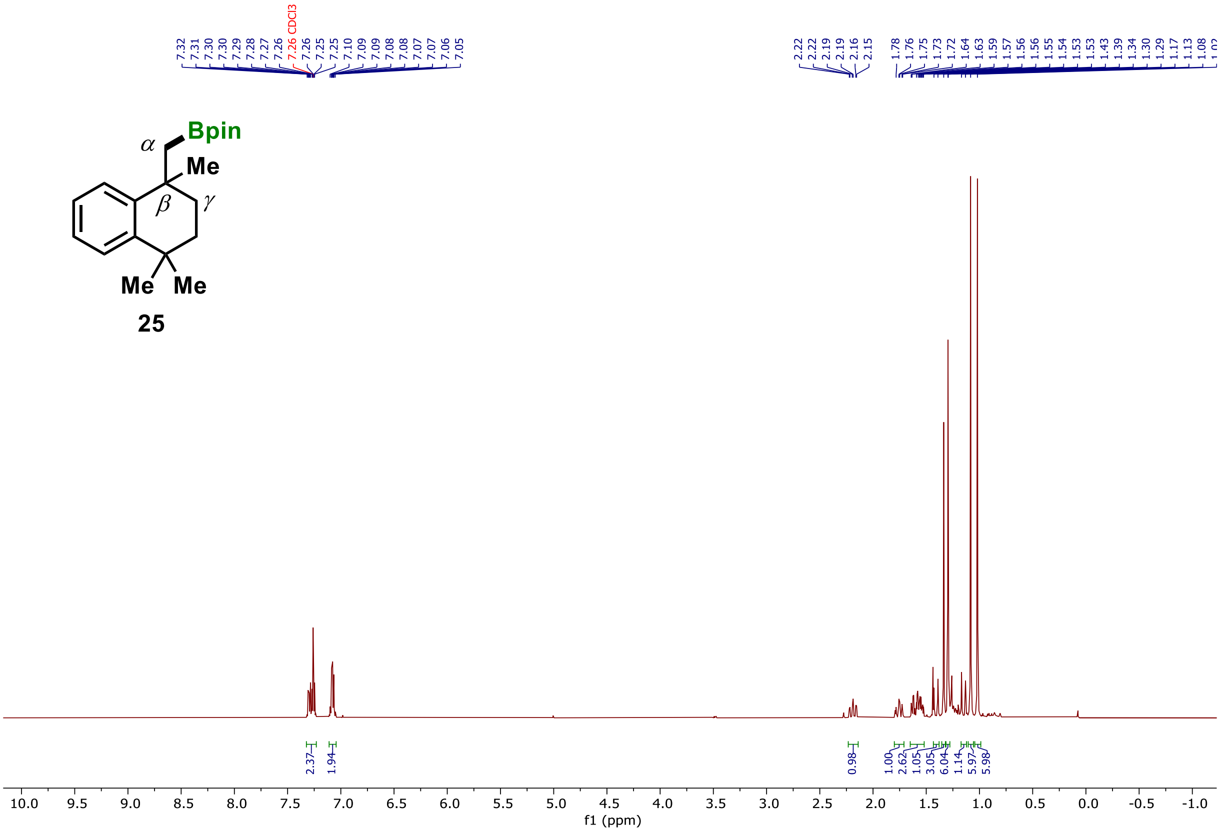

^{13}C NMR (101 MHz, $CDCl_3$) of **25**

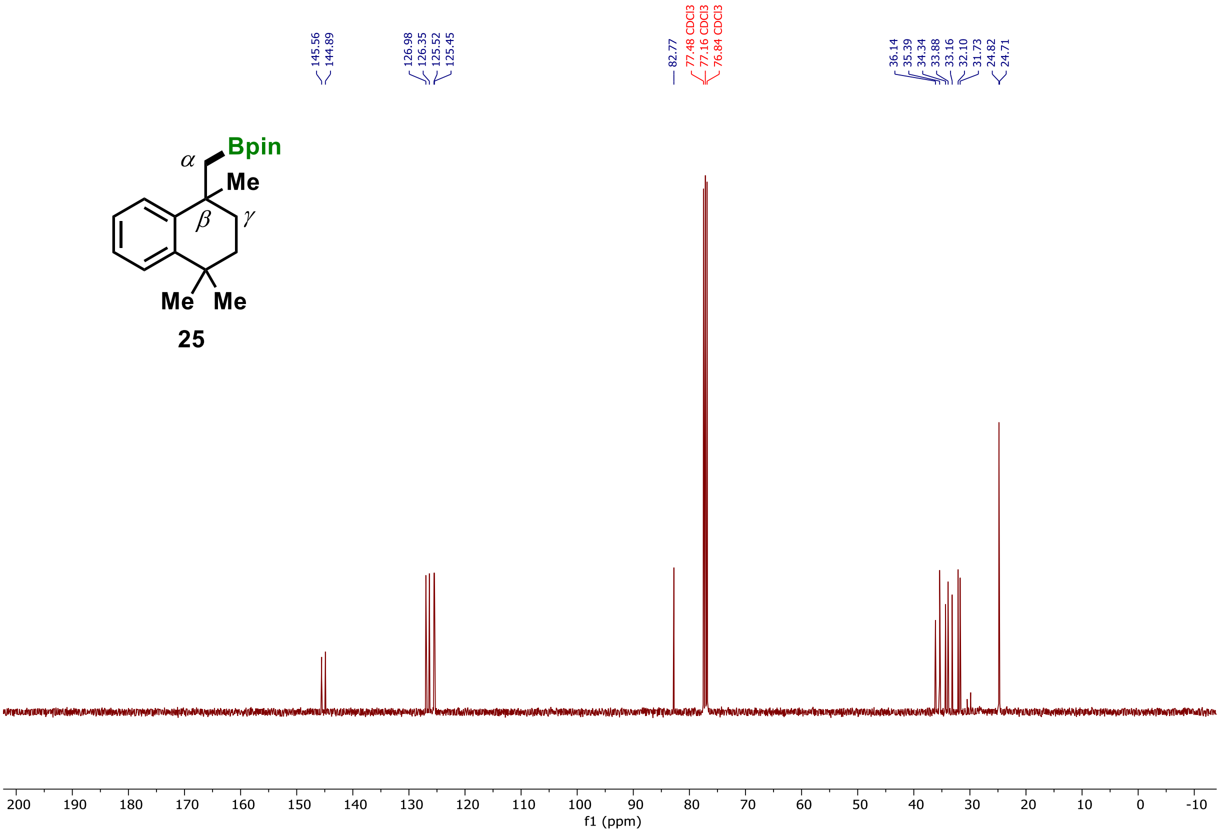

^{1}H NMR (400 MHz, $CDCl_3$) of **26** (*see procedure*)

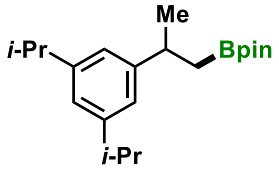

26

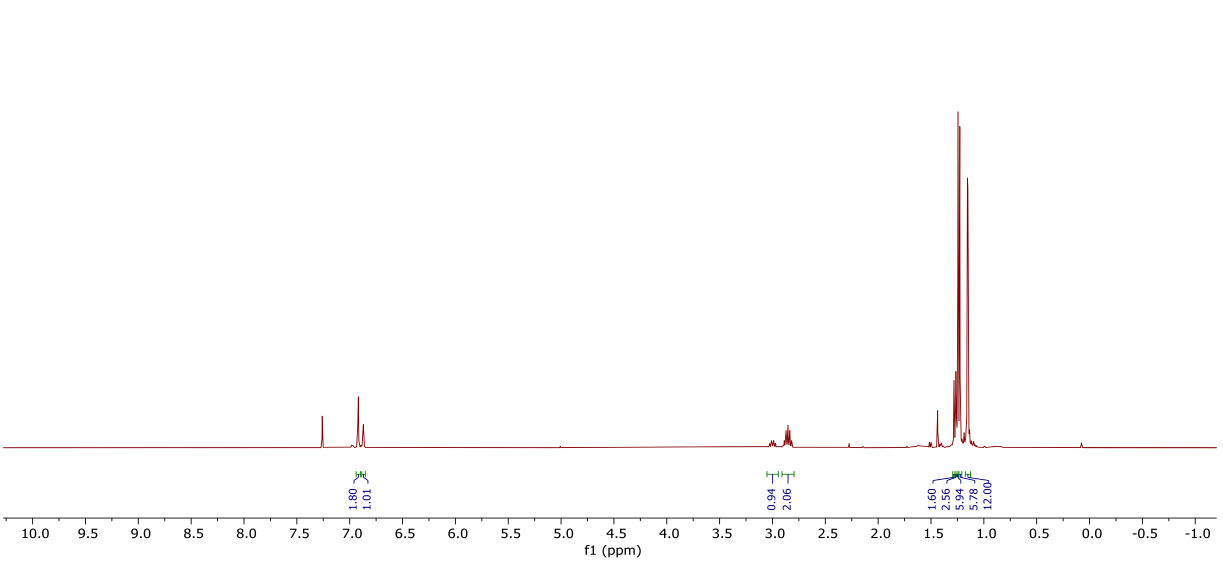

^{13}C NMR (101 MHz, $CDCl_3$) of **26**

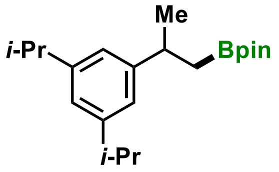

26

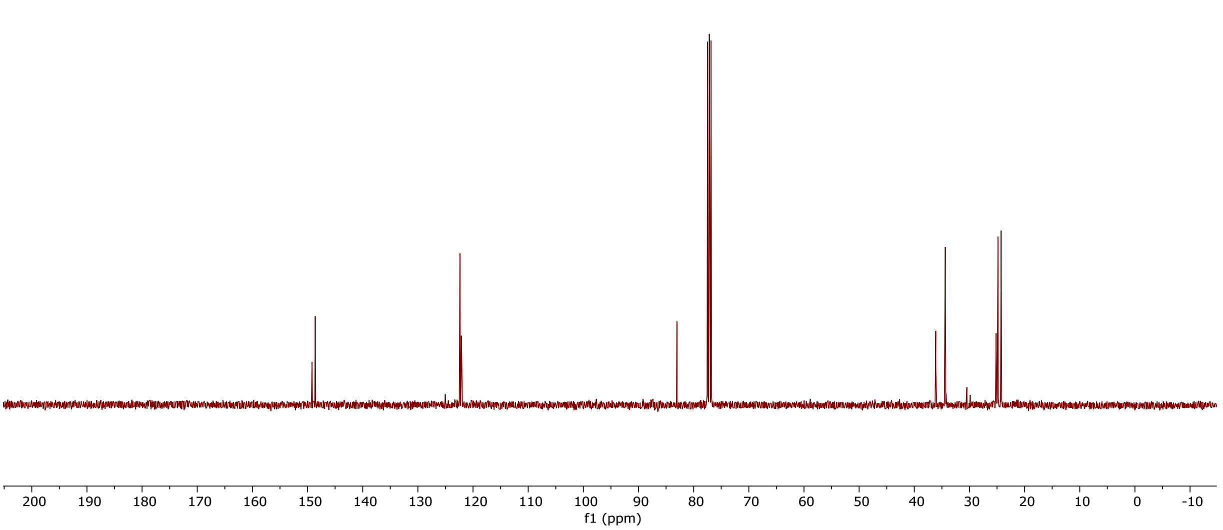

^{1}H NMR (400 MHz, $CDCl_3$) of **27** (*see procedure*)

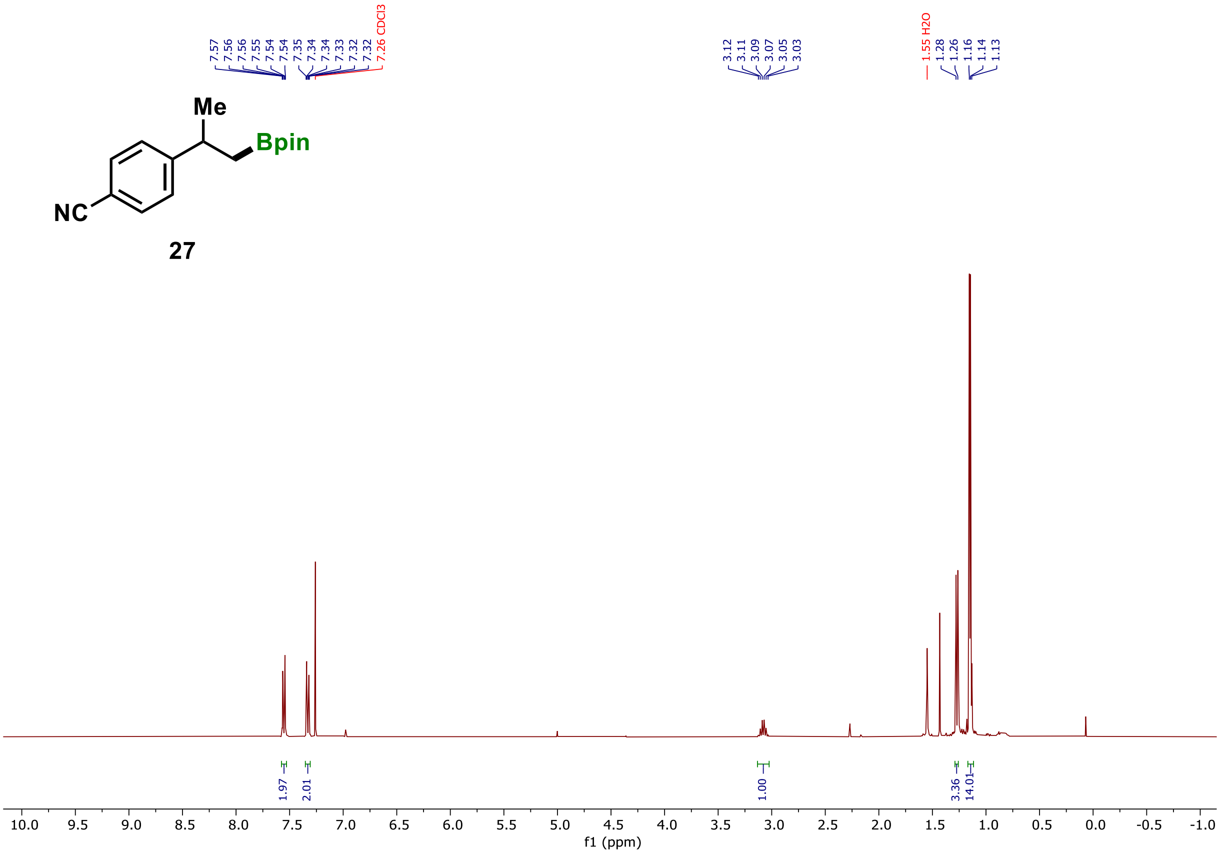

^{13}C NMR (101 MHz, $CDCl_3$) of **27**

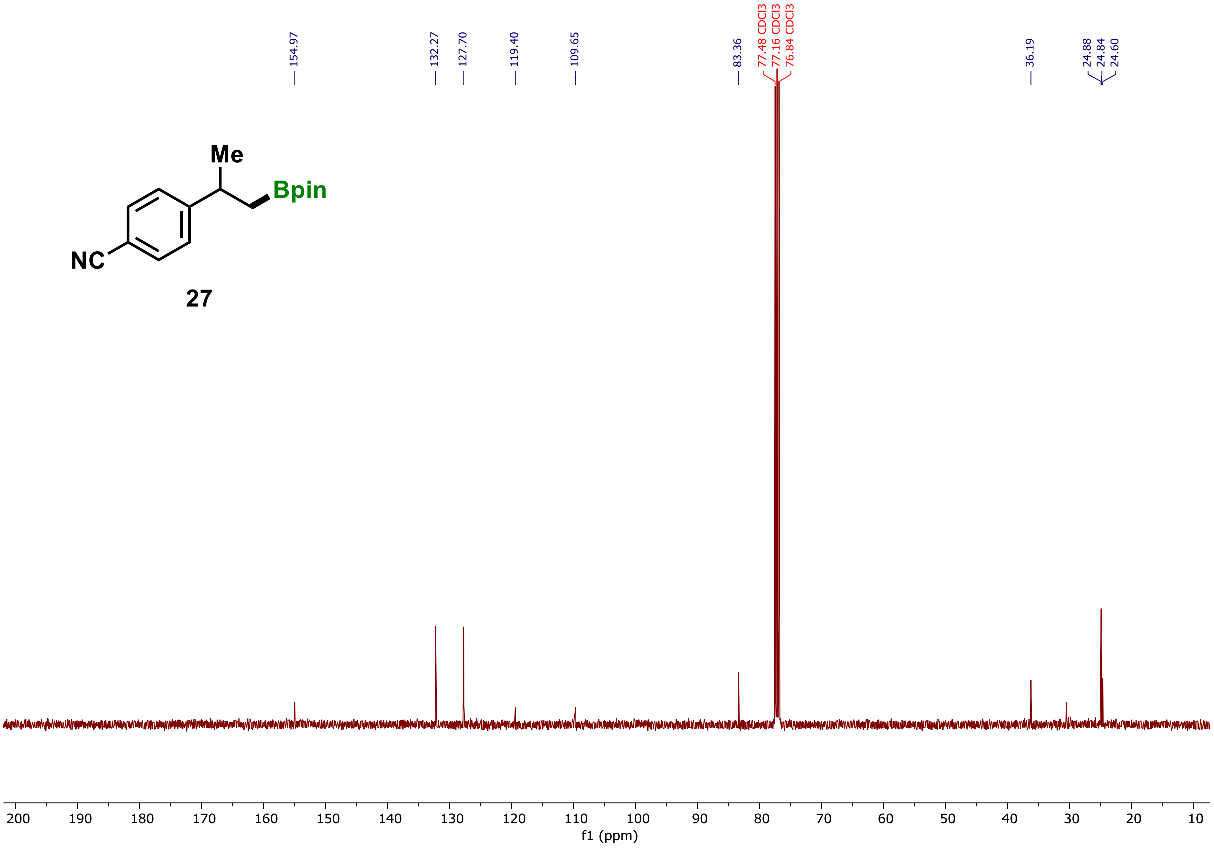

^{1}H NMR (400 MHz, $CDCl_3$) of **28** (*see procedure*)

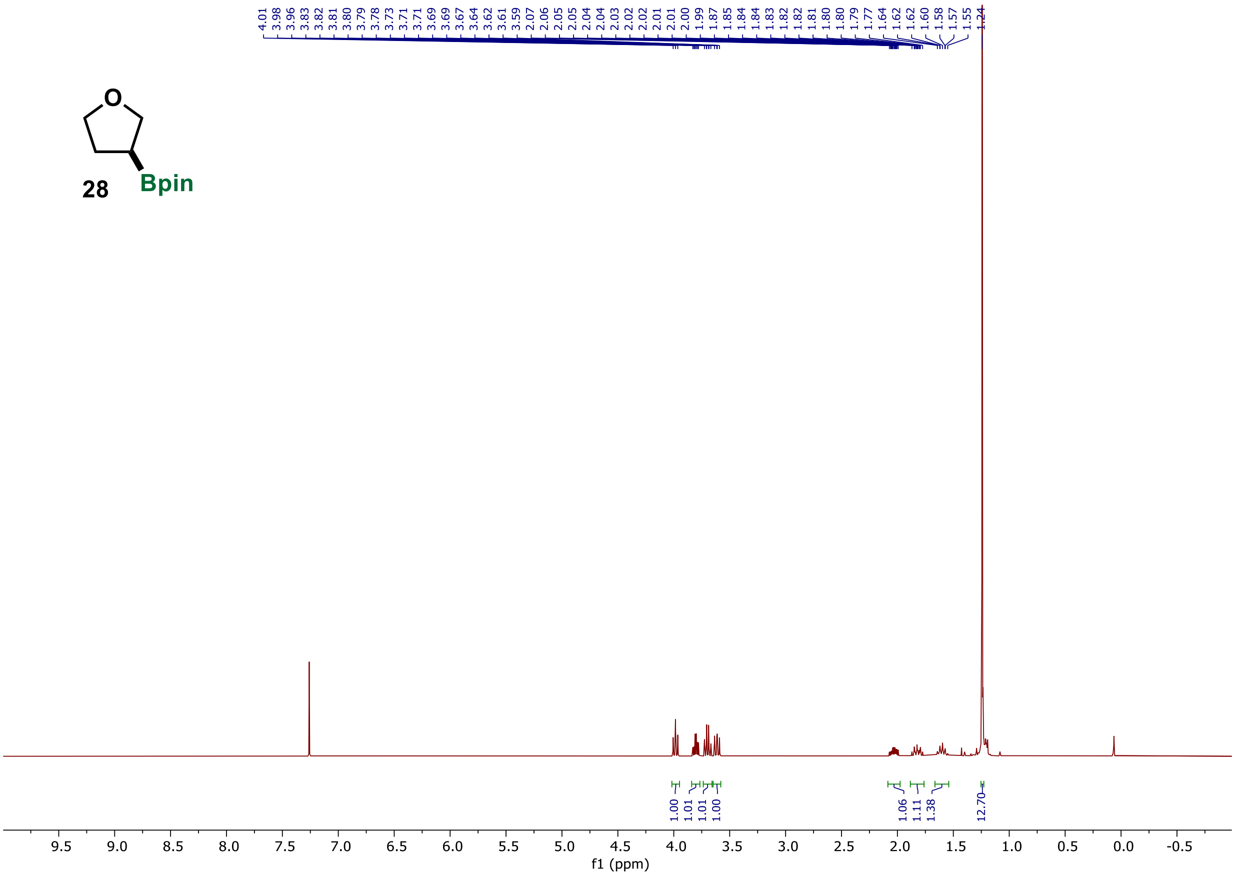

^{13}C NMR (101 MHz, $CDCl_3$) of **28**

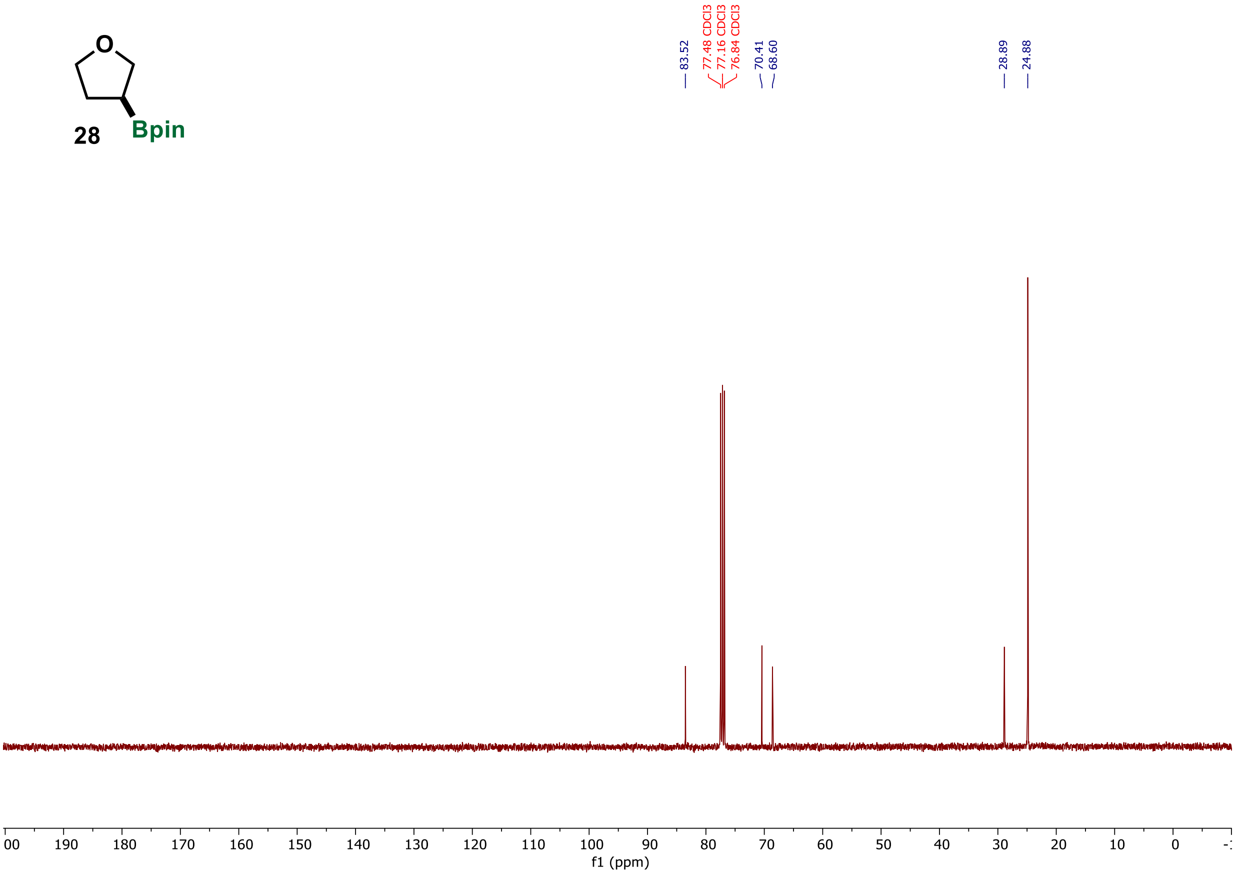

^{1}H NMR (400 MHz, $CDCl_3$) of **29a** and **29b** (*see procedure*)

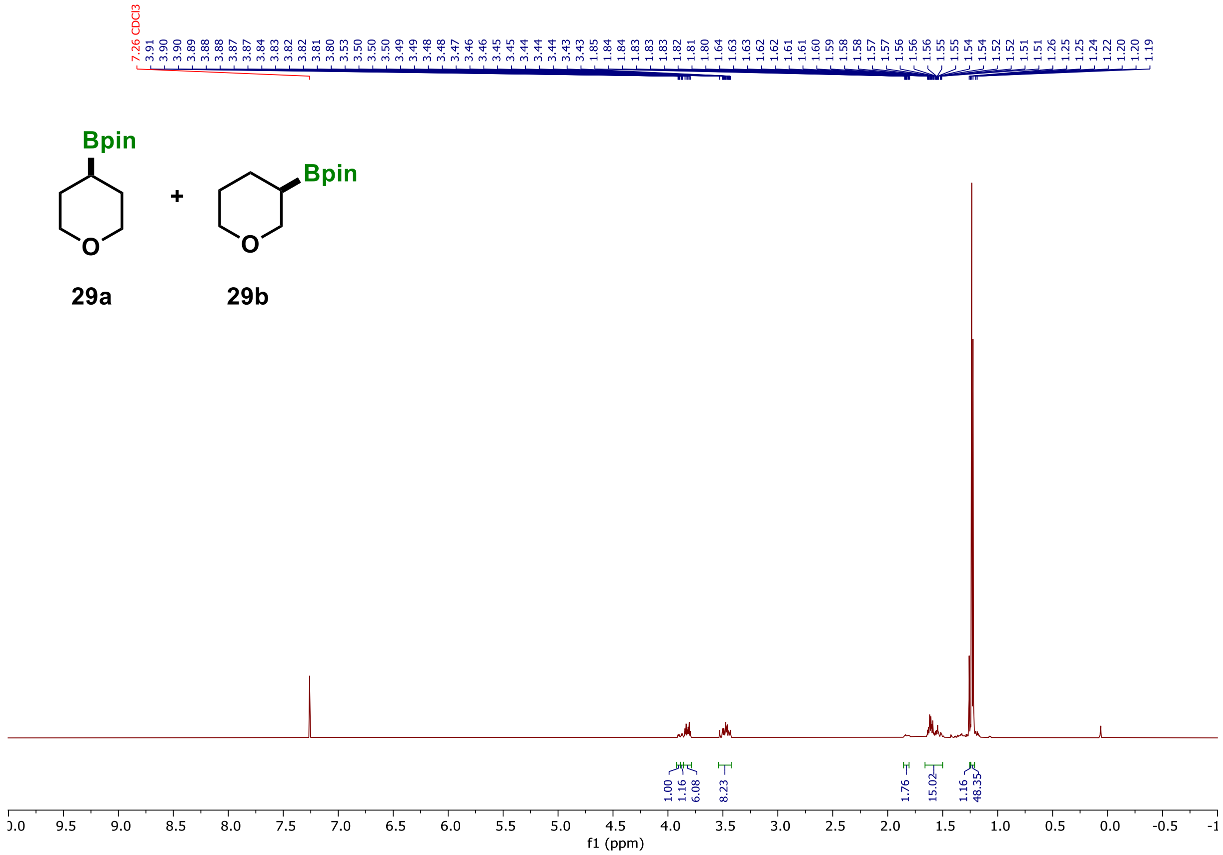

^{13}C NMR (101 MHz, $CDCl_3$) of **29a** and **29b**

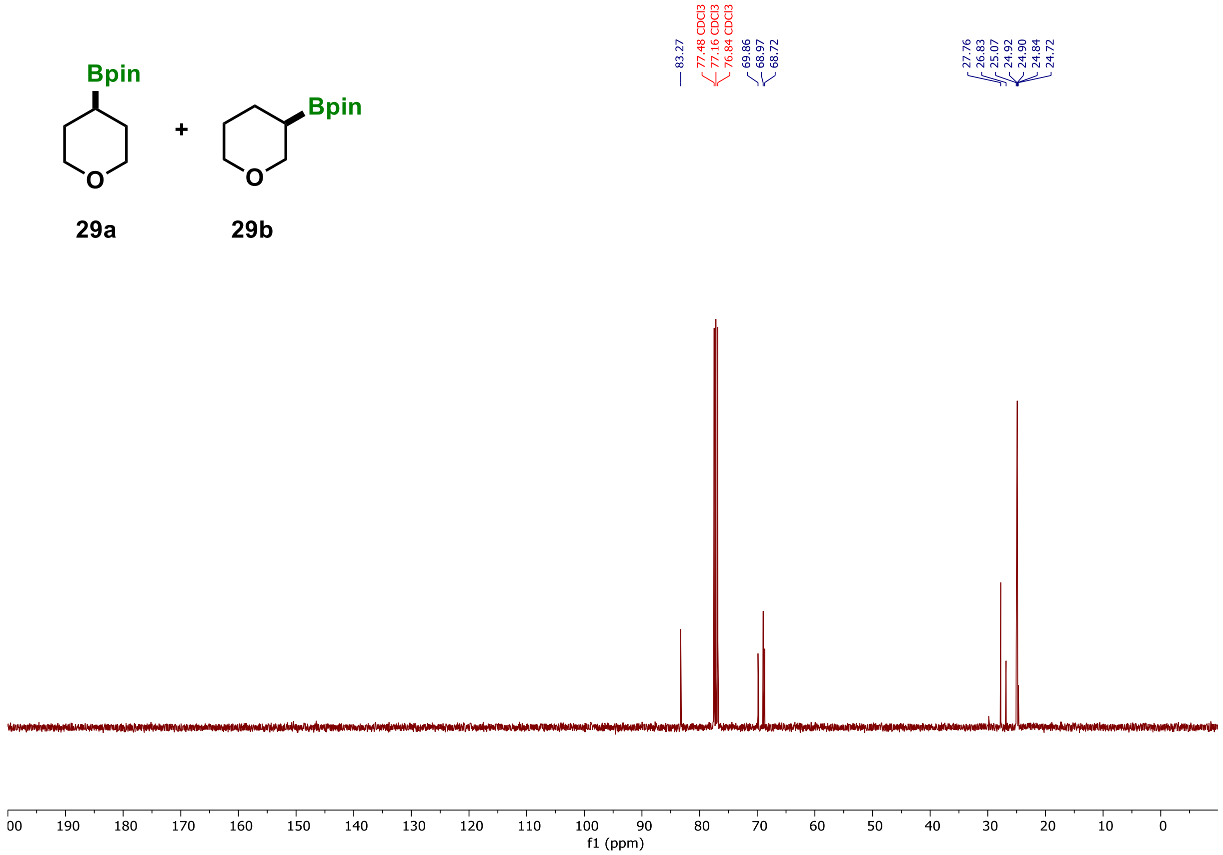

^{1}H NMR (400 MHz, $CDCl_3$) of **30** (*see procedure*)

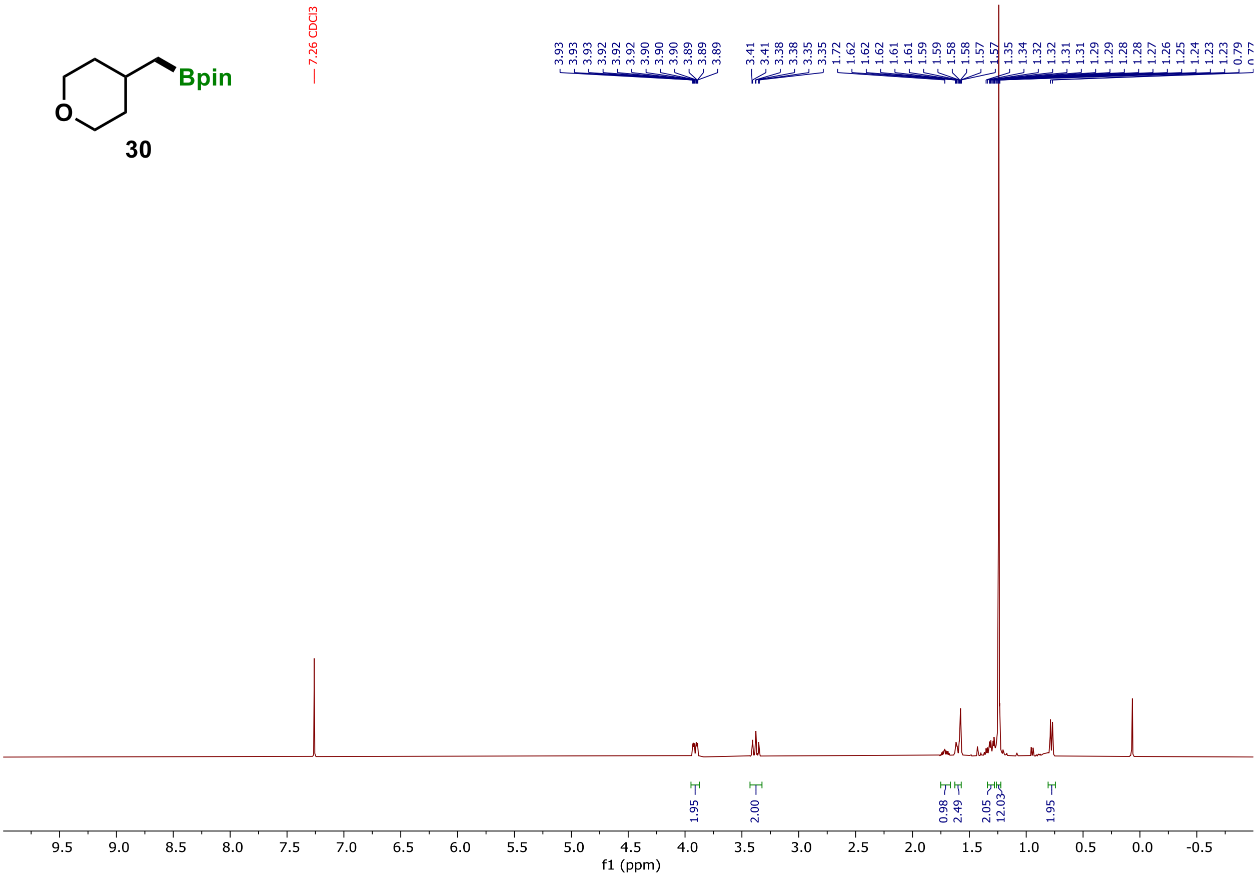

^{13}C NMR (101 MHz, $CDCl_3$) of **30**

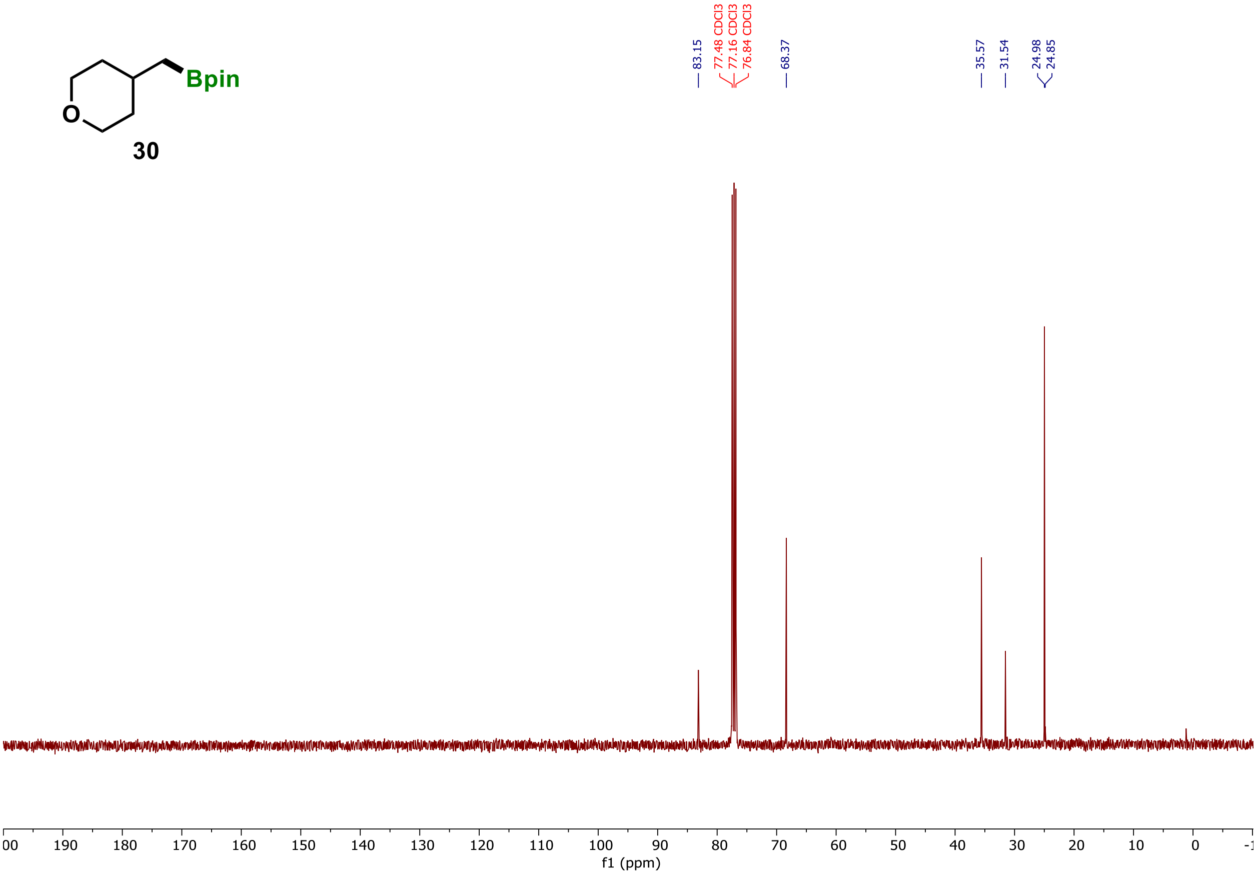

^{1}H NMR (400 MHz, $CDCl_3$) of **31** (***see procedure***)

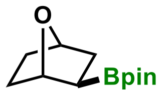

31

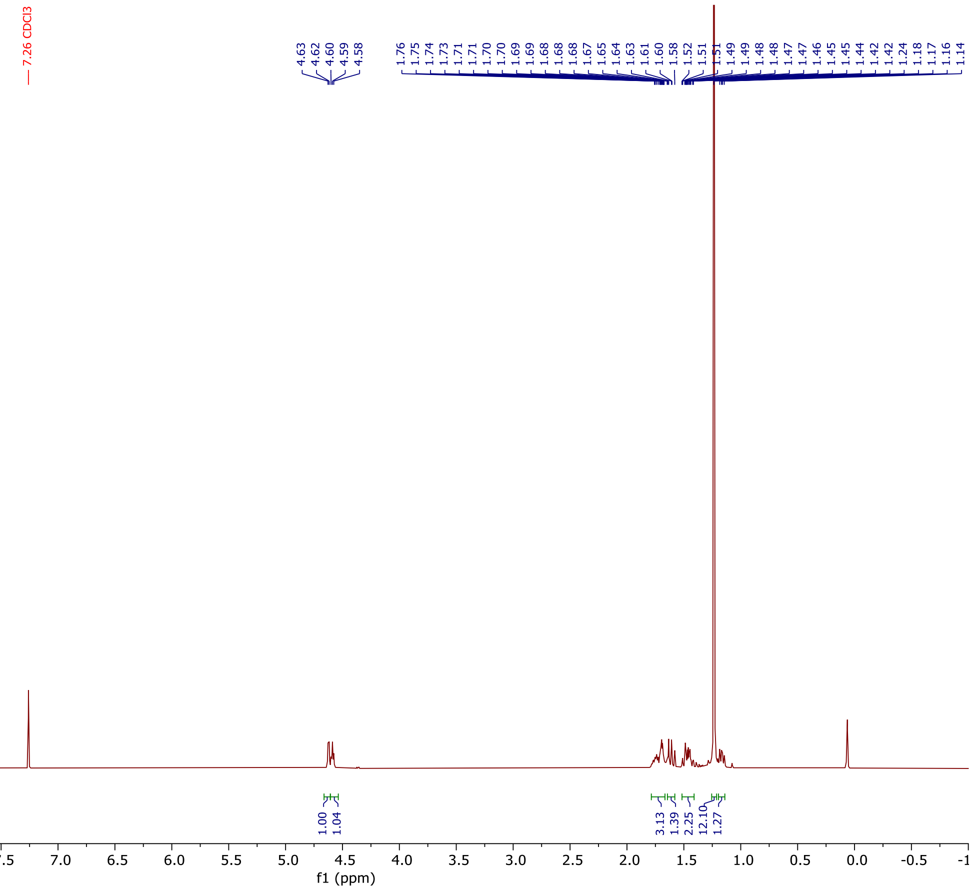

^{13}C NMR (101 MHz, $CDCl_3$) of **31**

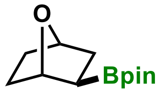

31

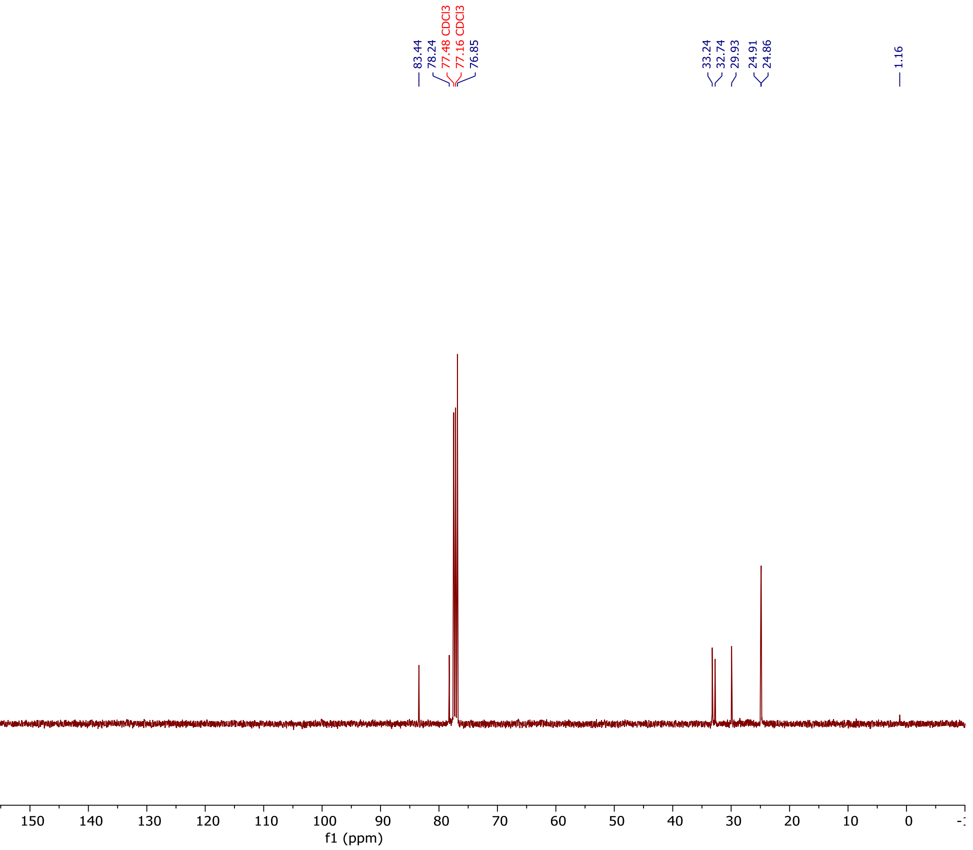

^{11}B NMR (128 MHz, $CDCl_3$) of **31**

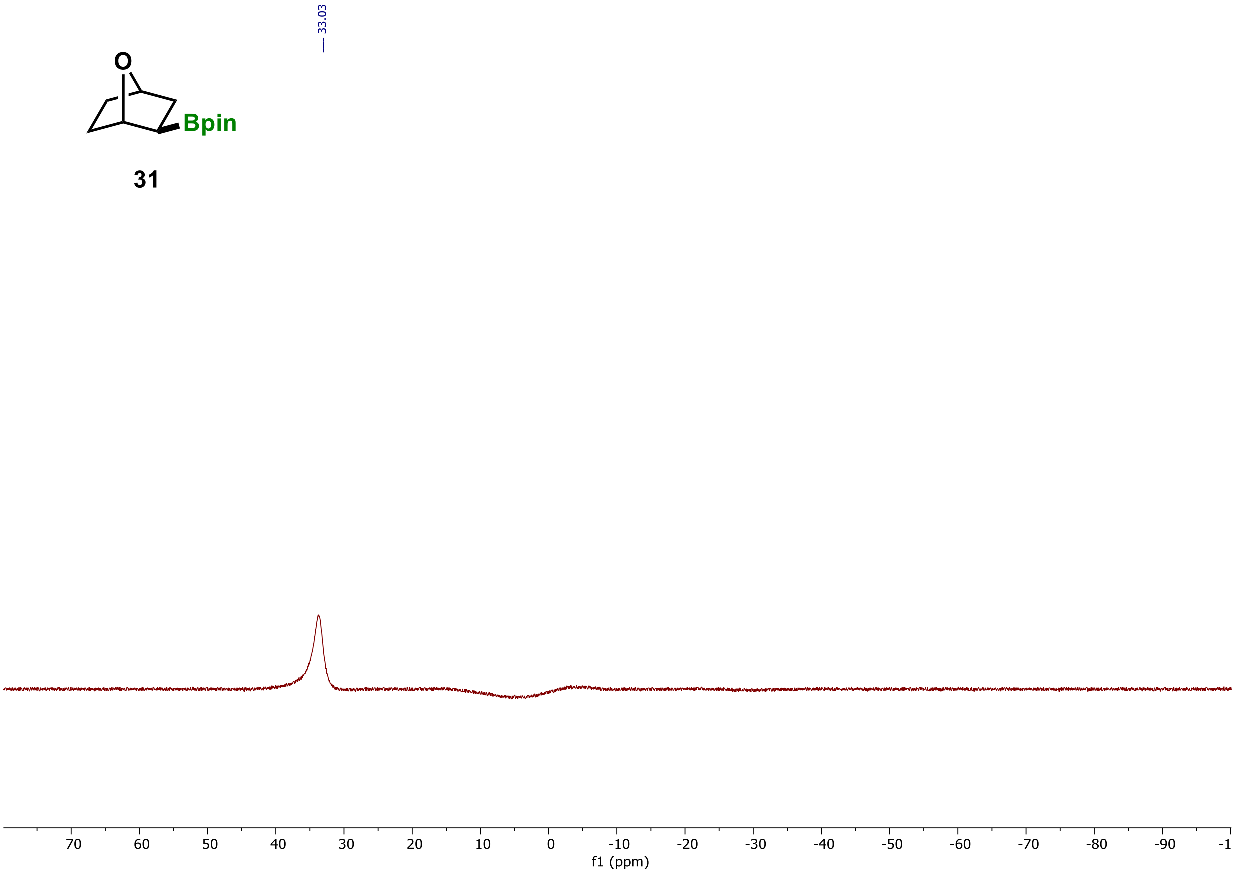

Key TOCSY ^1H-^1H Correlation of **31**

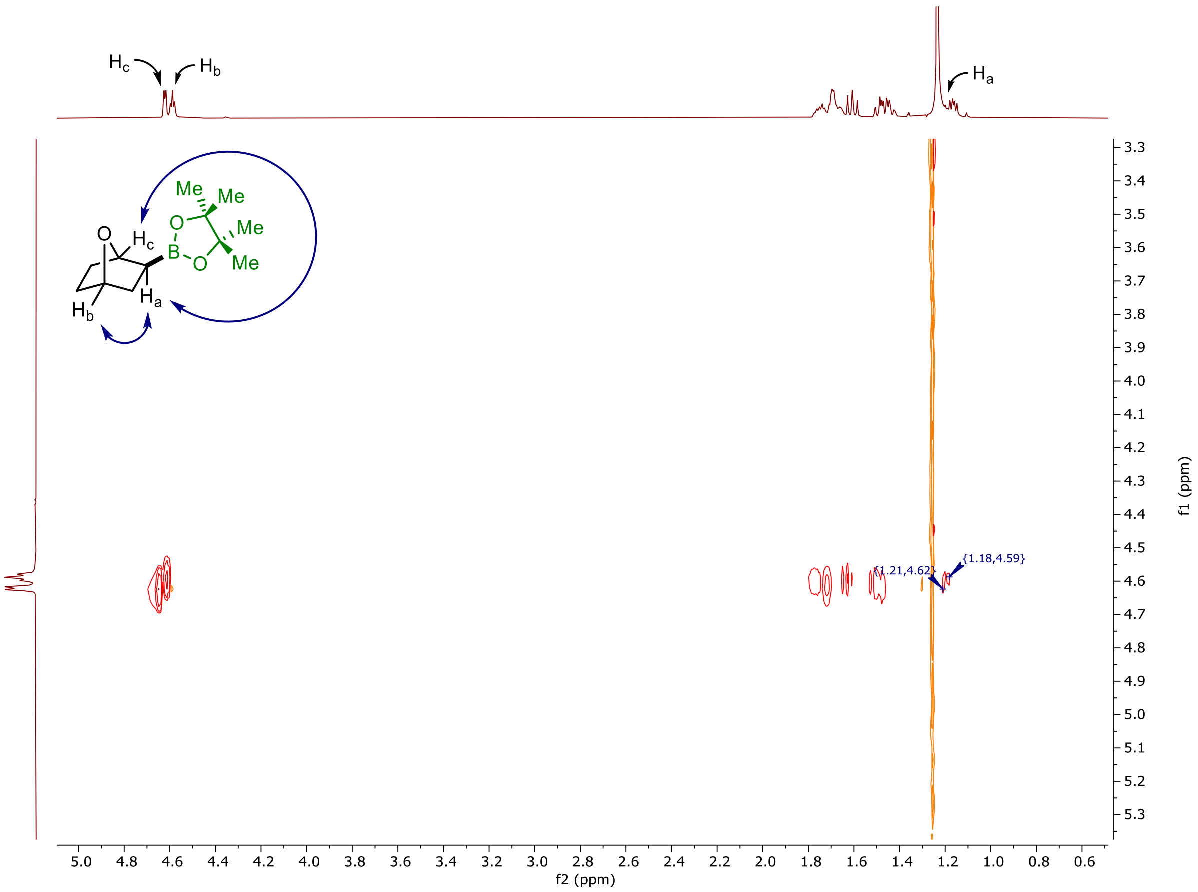

^{1}H NMR (400 MHz, $CDCl_3$) of **32** (*see procedure*)

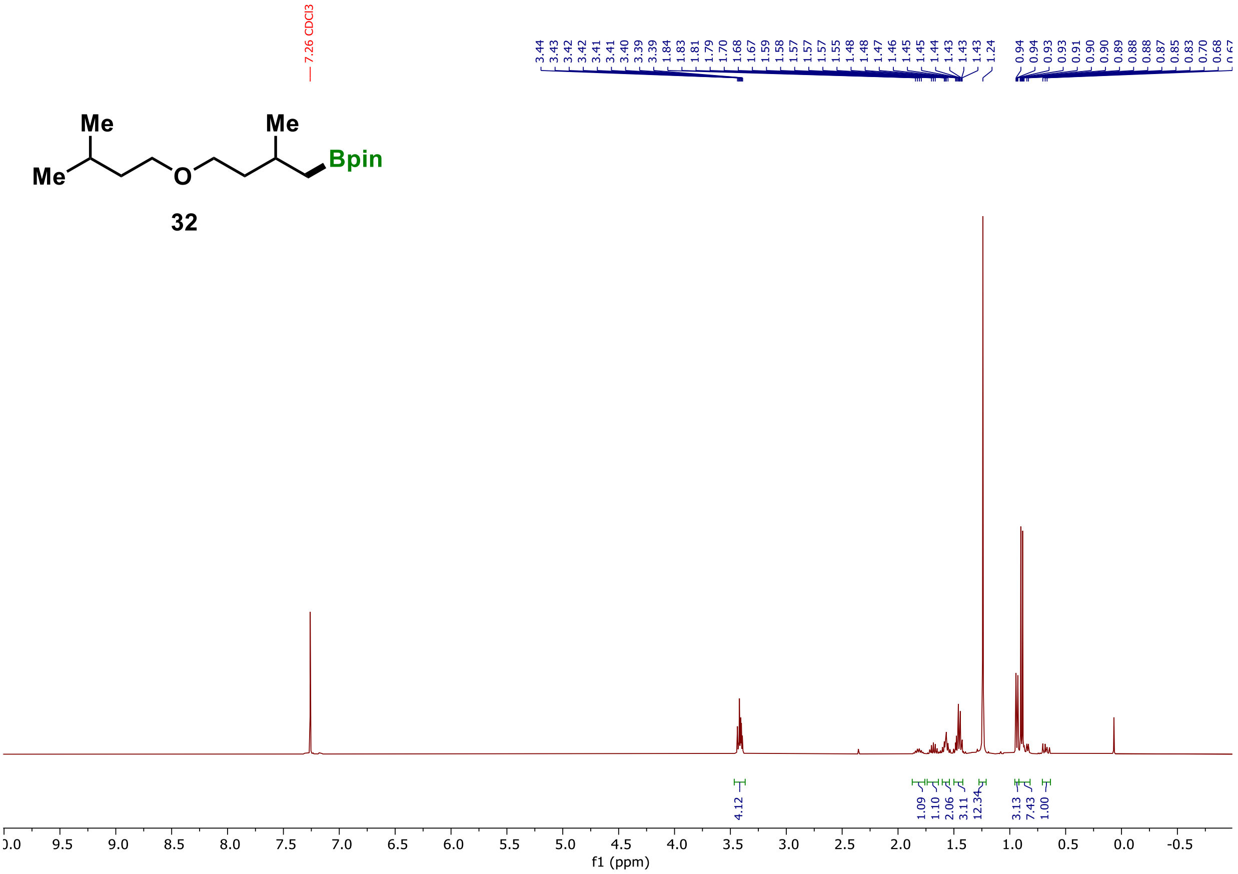

^{13}C NMR (101 MHz, $CDCl_3$) of **32**

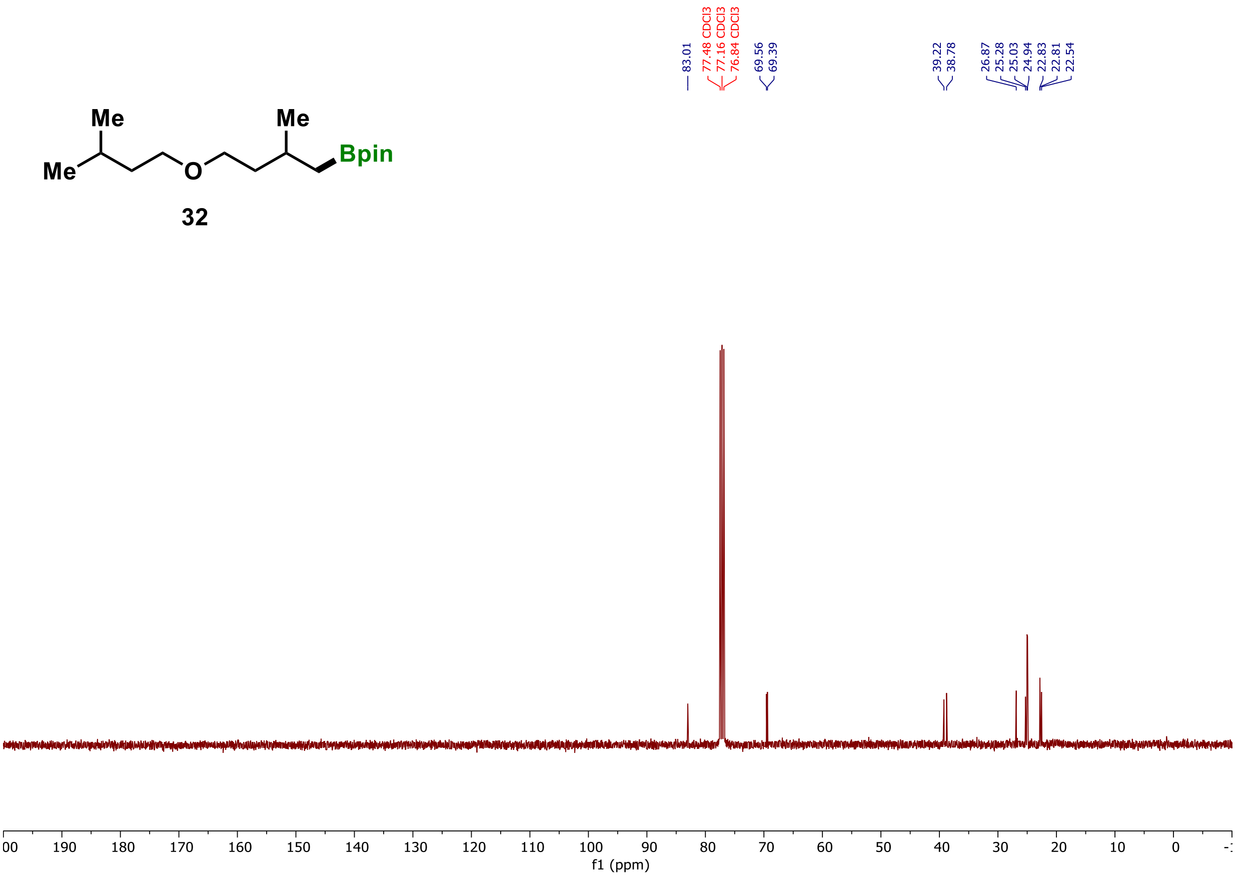

^{1}H NMR (400 MHz, $CDCl_3$) of **33a** (*see procedure*)

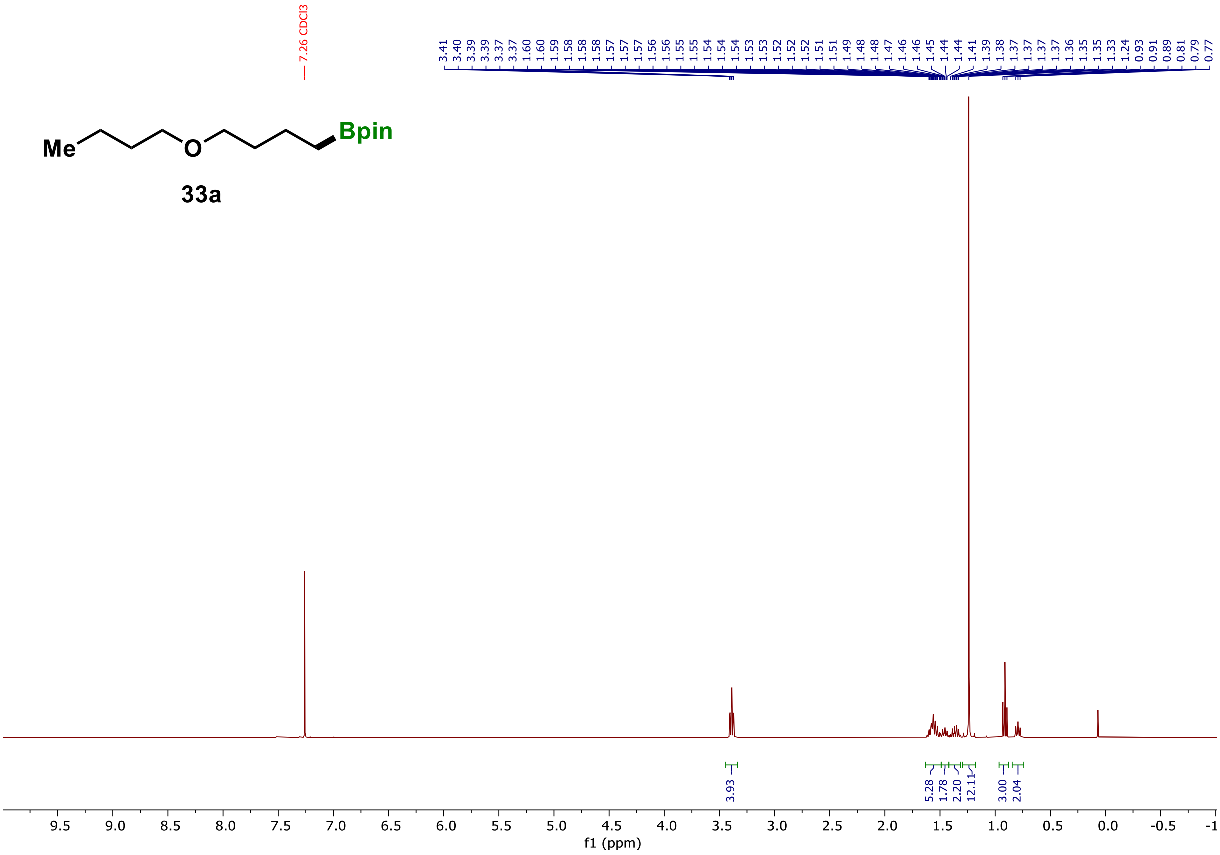

^{13}C NMR (101 MHz, $CDCl_3$) of **33a**

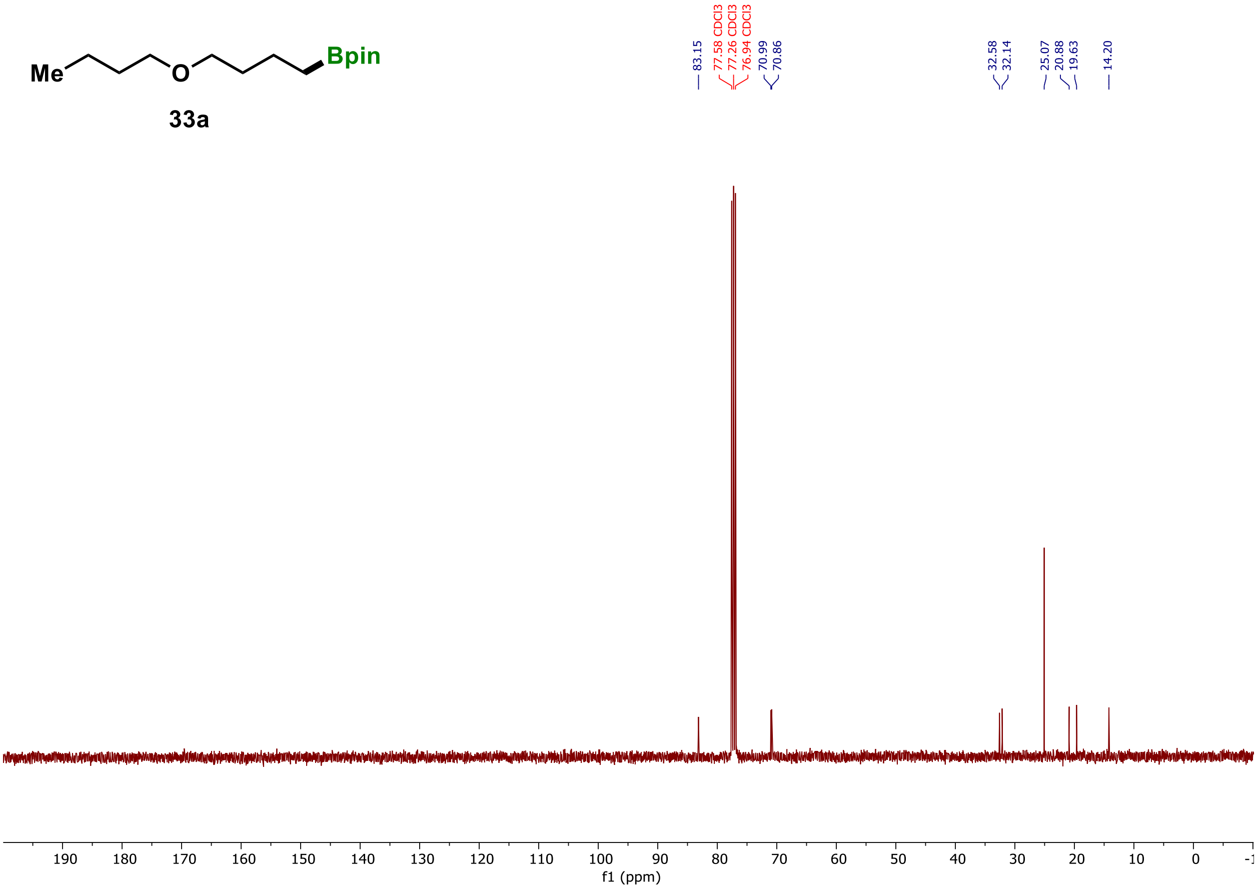

^{1}H NMR (400 MHz, $CDCl_3$) **33b** (*see procedure*)

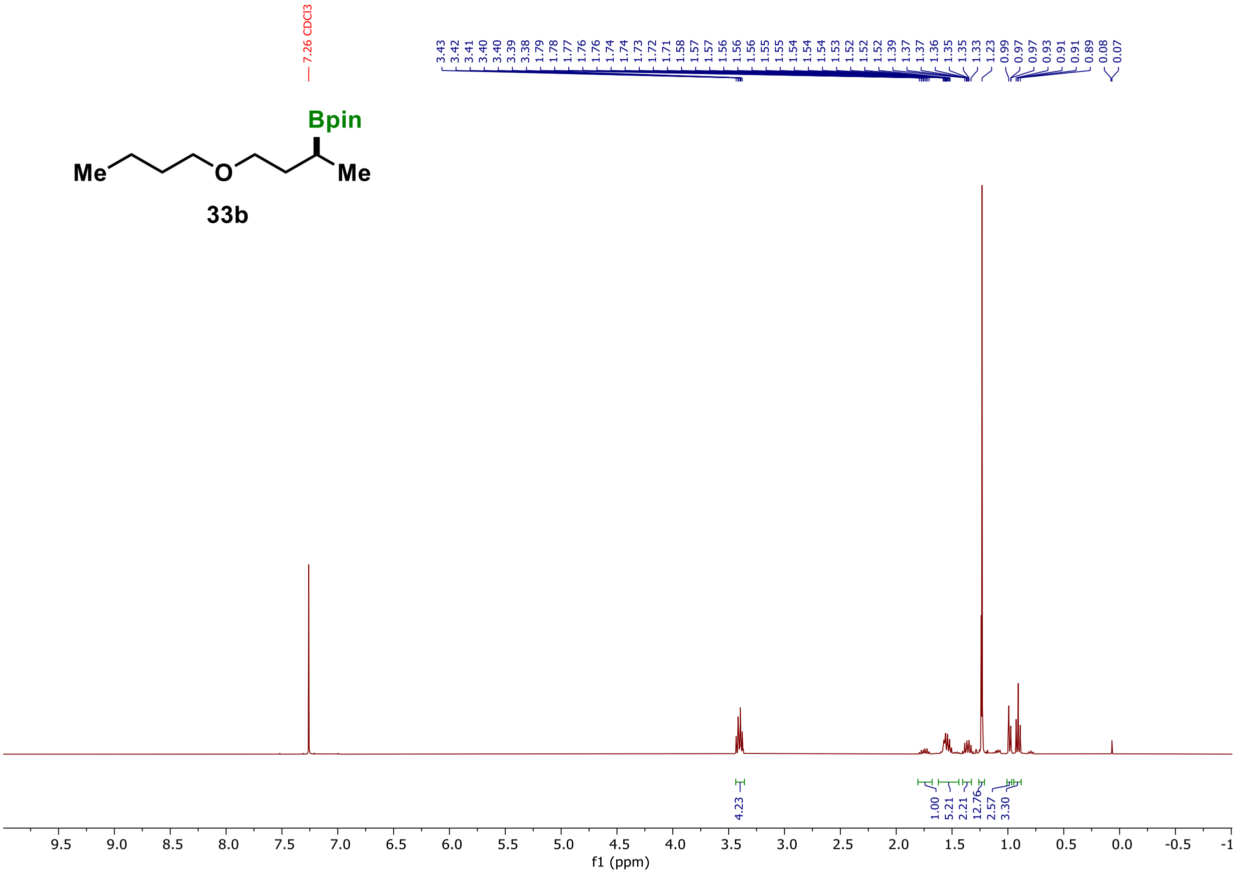

^{13}C NMR (101 MHz, $CDCl_3$) of **33b**

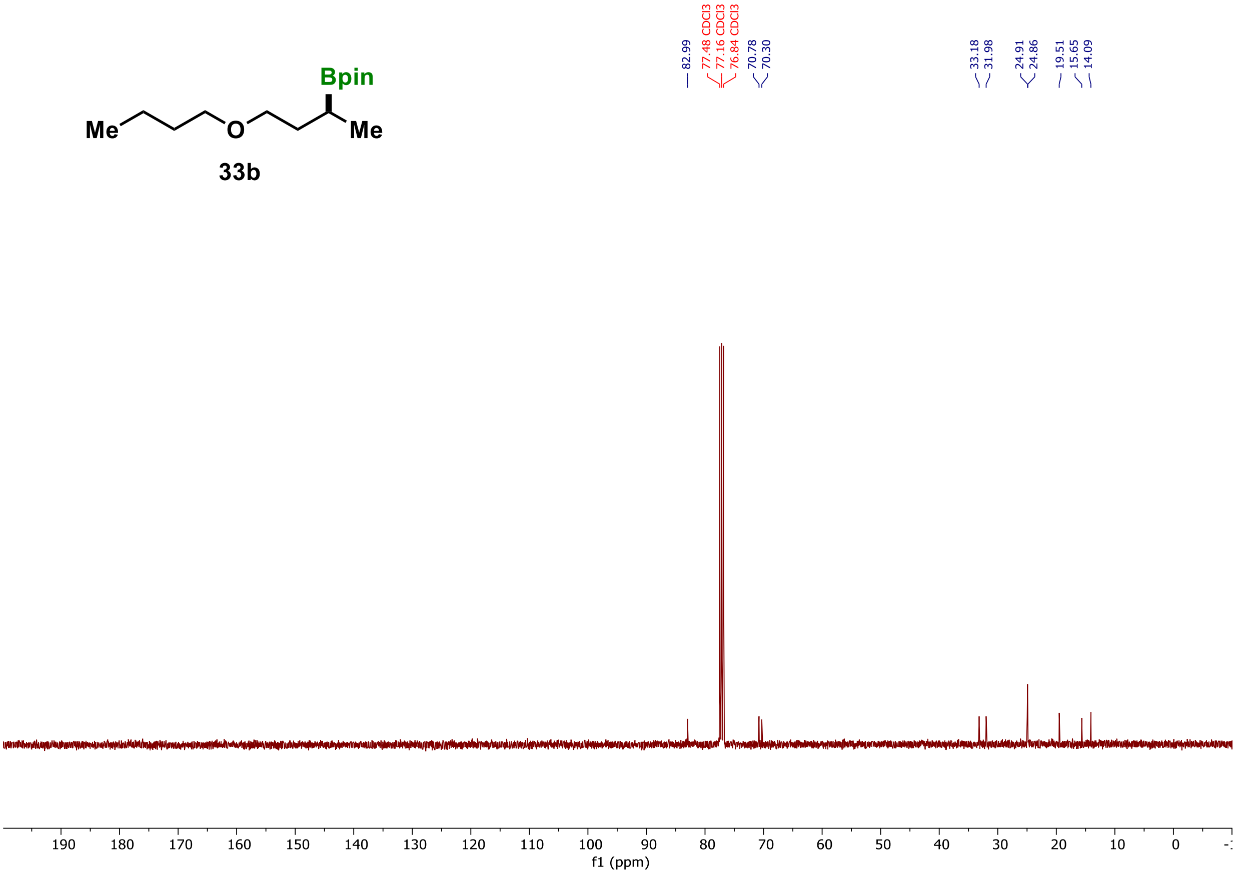

^{11}B NMR (128 MHz, $CDCl_3$) of **33b**

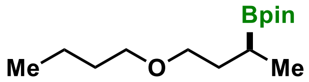

33b

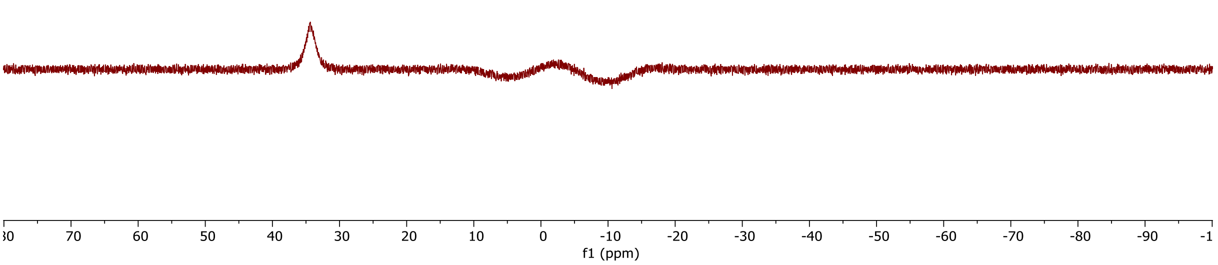

^{1}H NMR (400 MHz, $CDCl_3$) of **34** (*see procedure*)

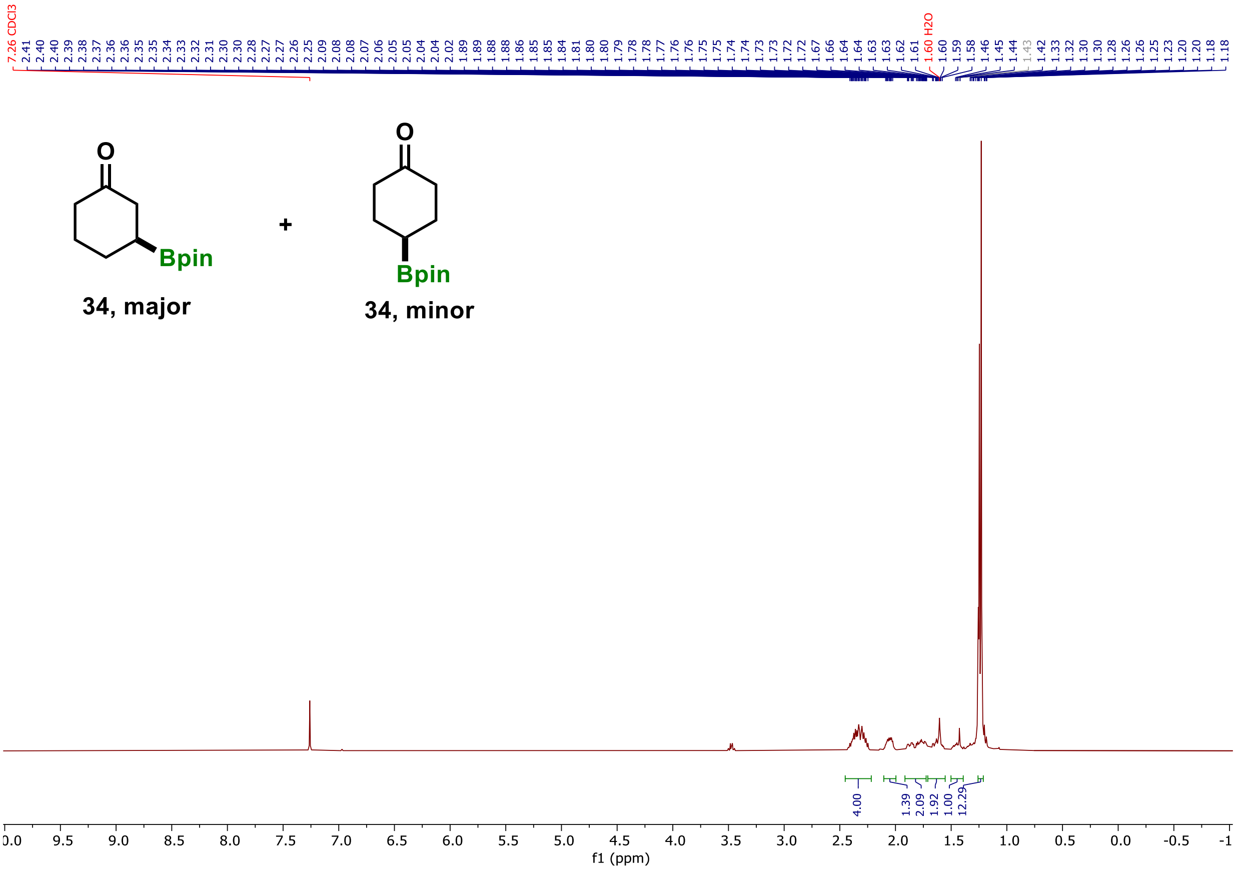

^{13}C NMR (101 MHz, $CDCl_3$) of **34**

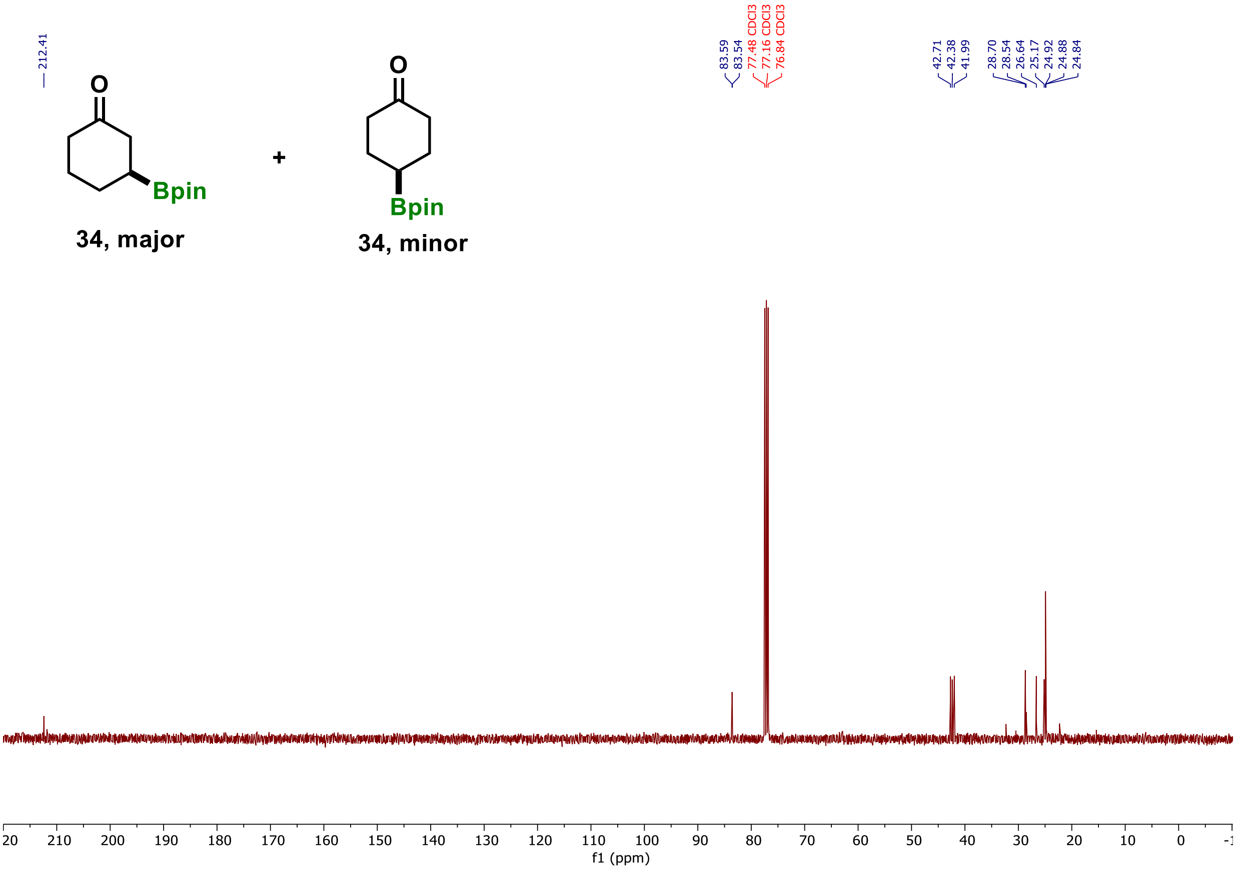

^{11}B NMR (128 MHz, $CDCl_3$) of **34**

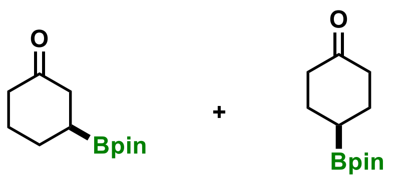

34, major **34, minor**

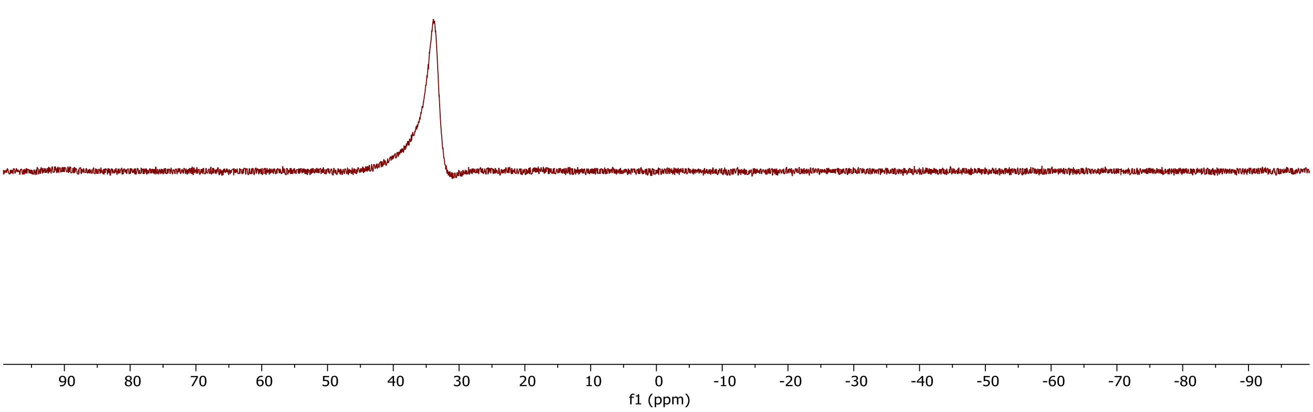

^{1}H NMR (400 MHz, $CDCl_3$) of **35** (***see procedure***)

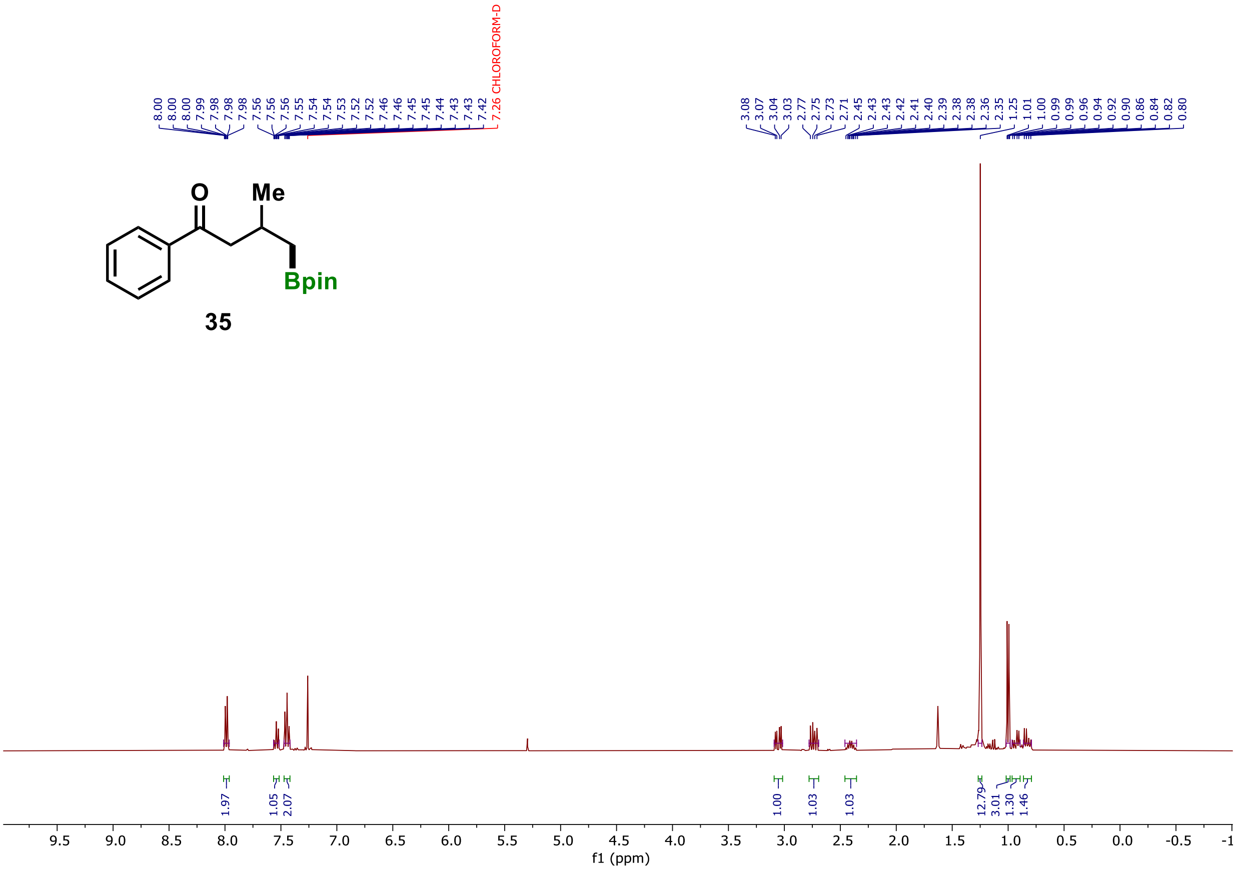

^{13}C NMR (101 MHz, $CDCl_3$) of **35**

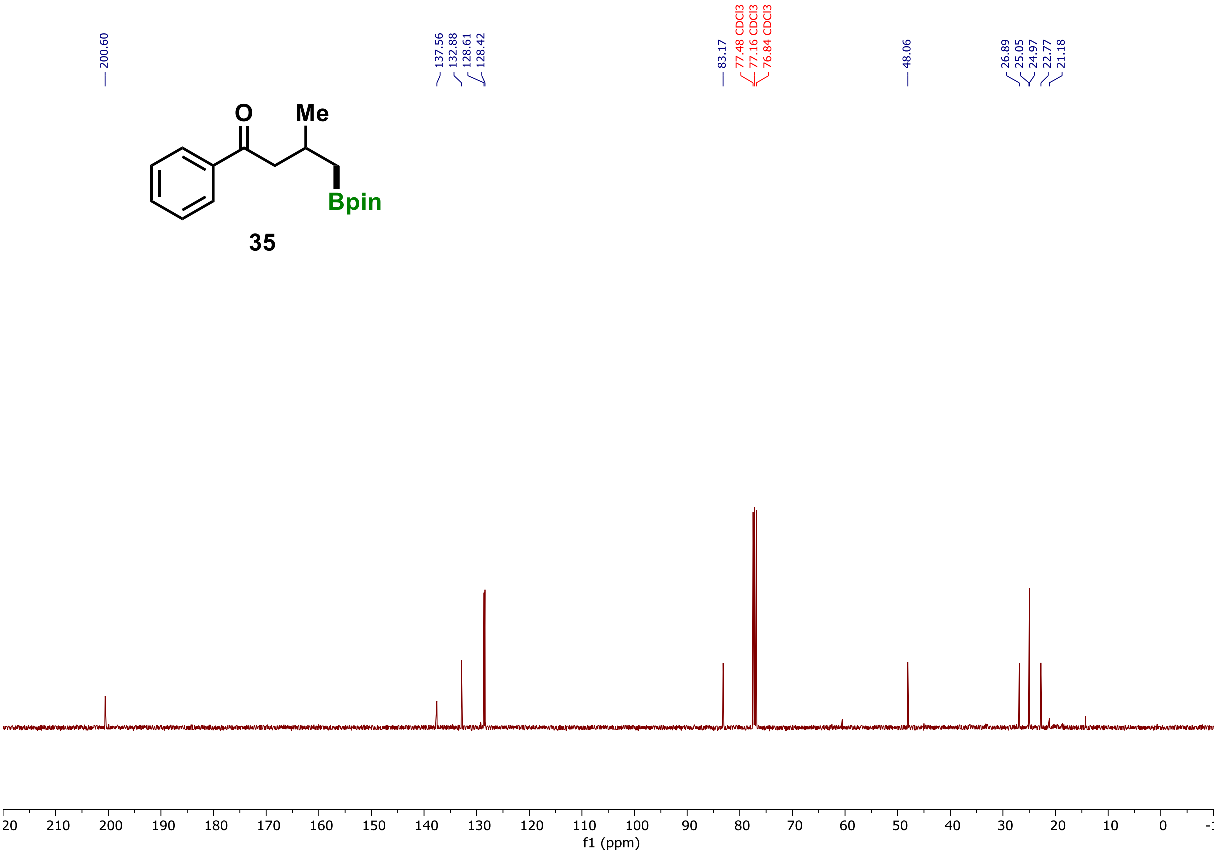

^{11}B NMR (128 MHz, CDCl3) of **35**

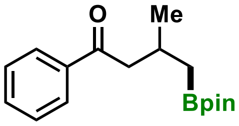

35

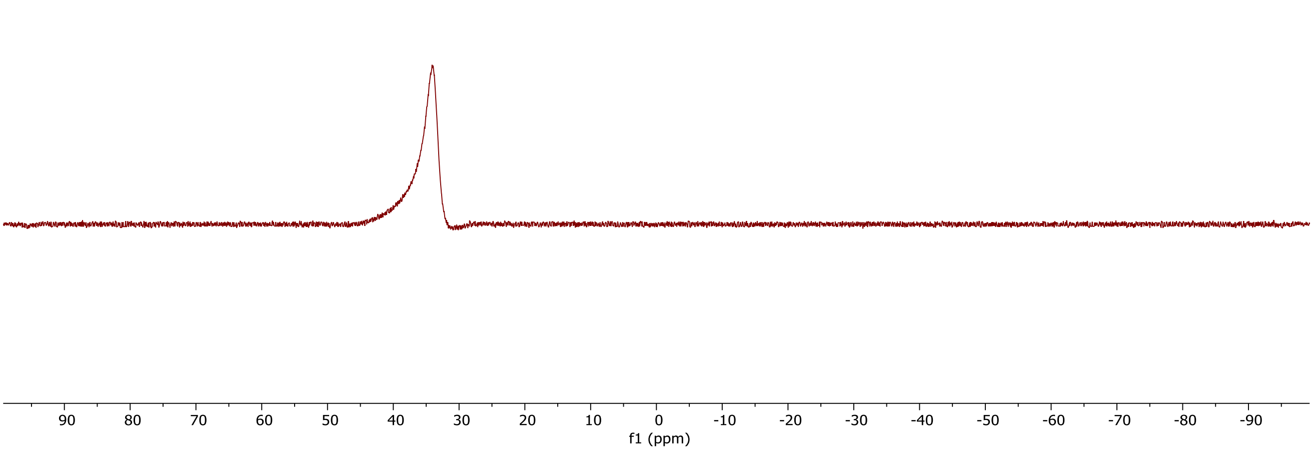

^{1}H NMR (400 MHz, $CDCl_3$) of **36** (*see procedure*)

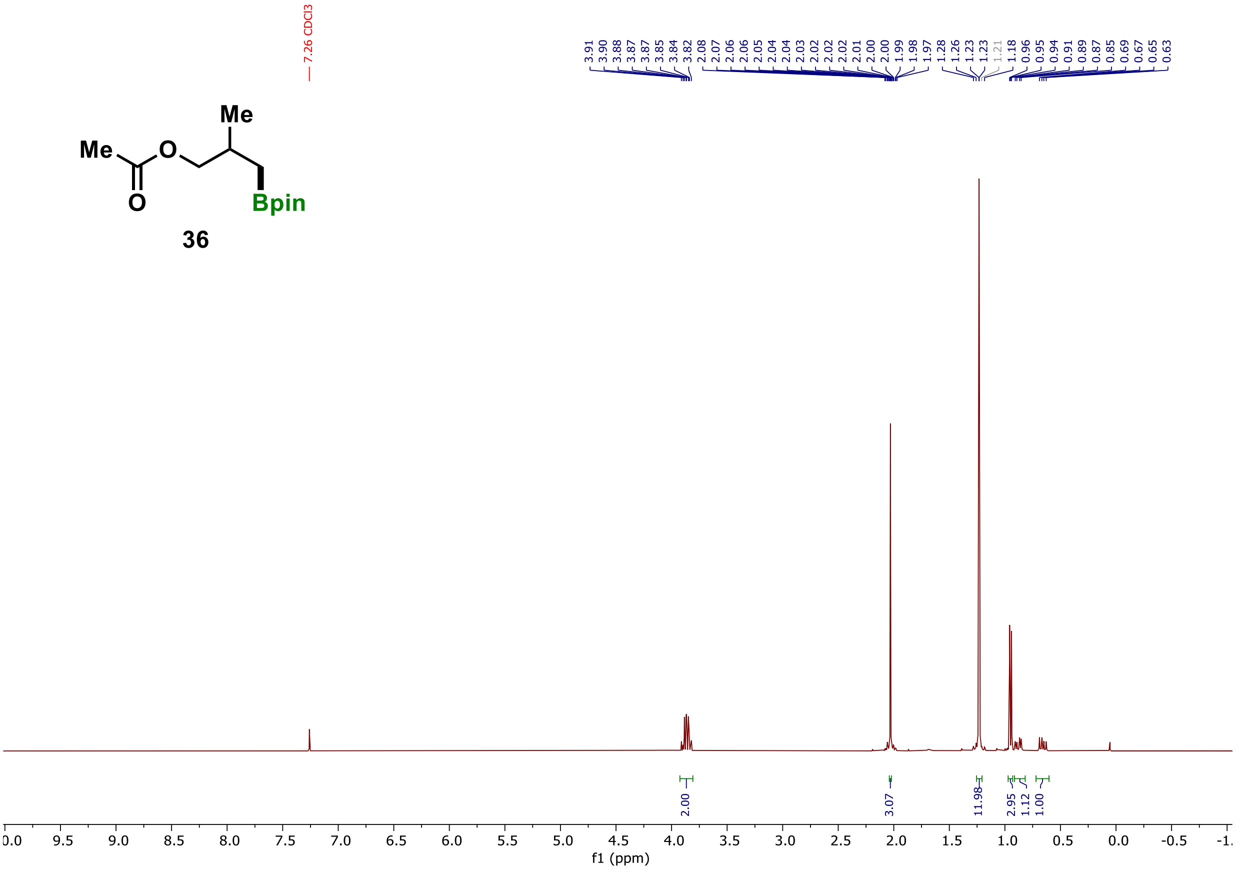

^{13}C NMR (101 MHz, $CDCl_3$) of **36**

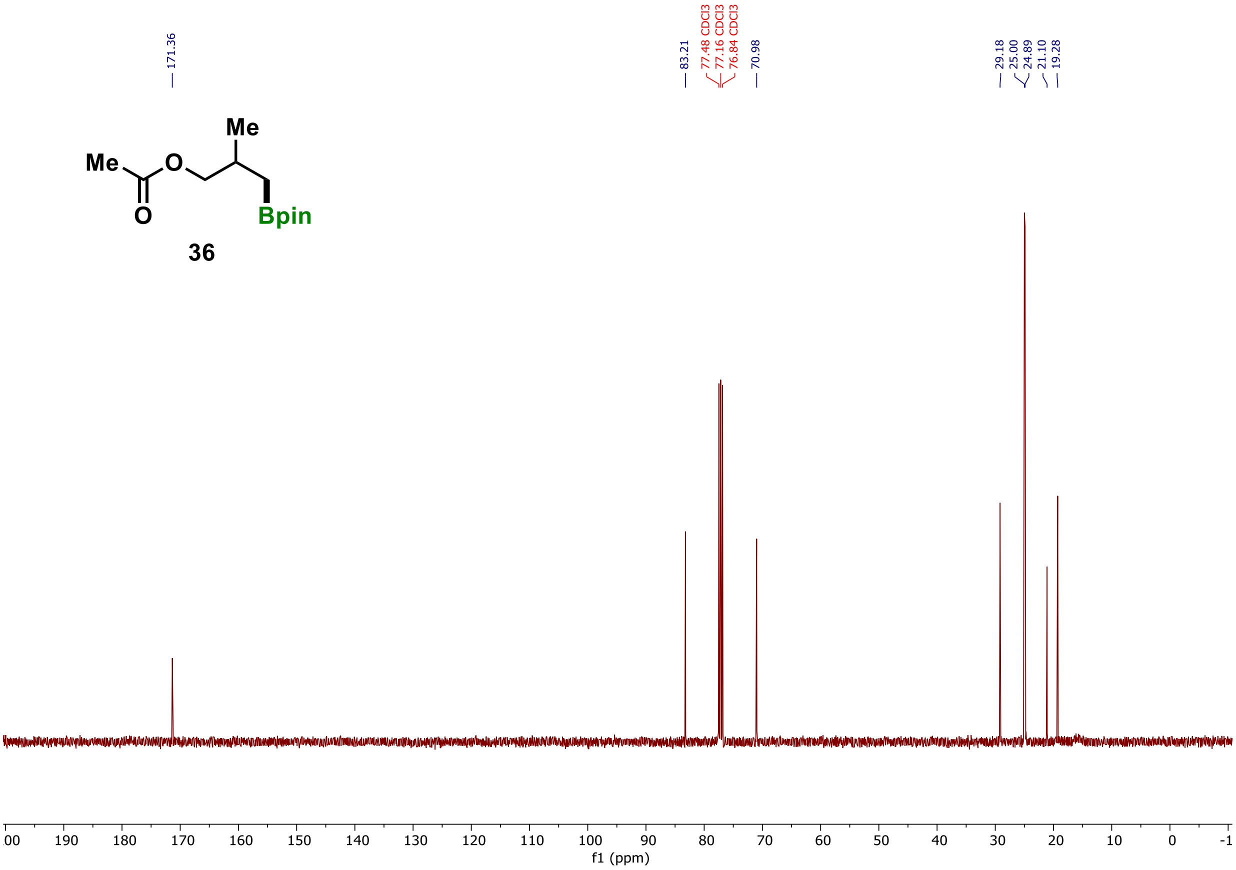

^{11}B NMR (128 MHz, CDCl3) of **36**

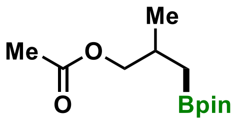

36

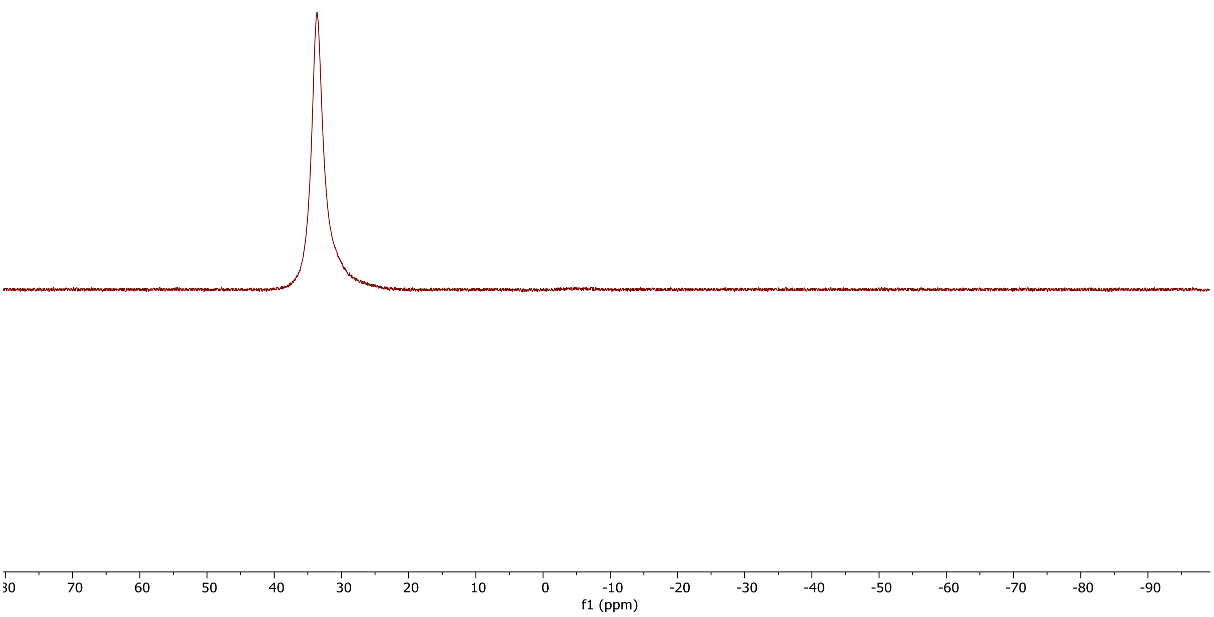

^{1}H NMR (400 MHz, $CDCl_3$) of **37** (*see procedure*)

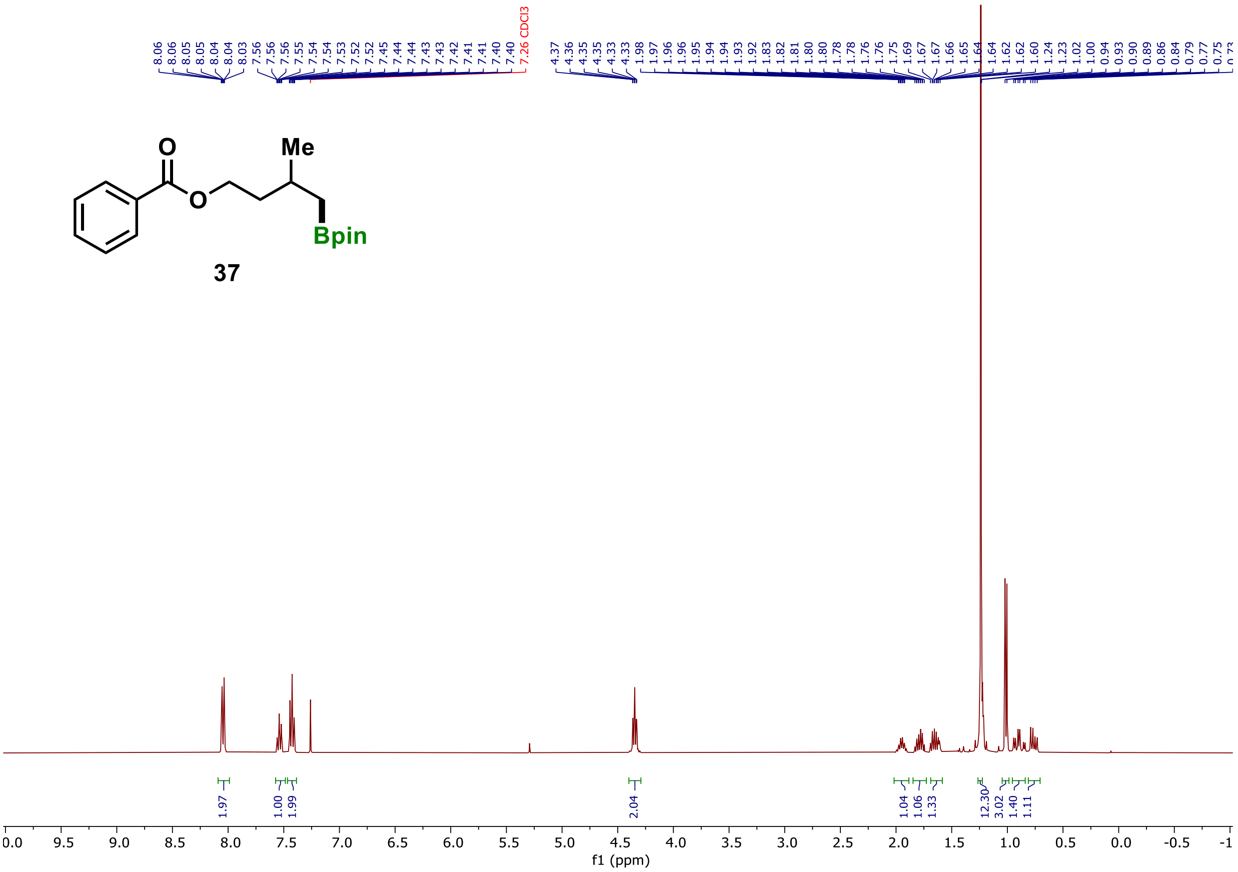

^{13}C NMR (101 MHz, $CDCl_3$) of **37**

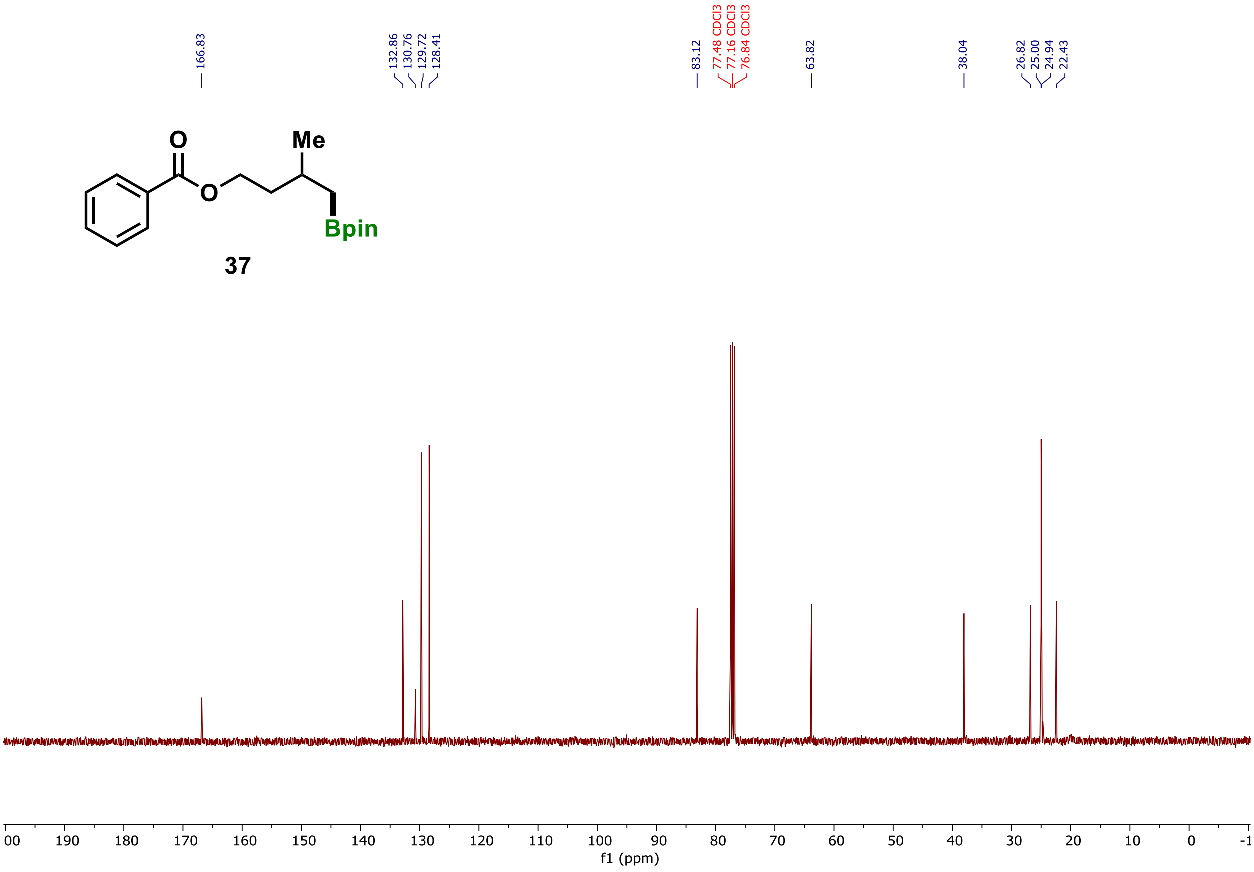

^{11}B NMR (128 MHz, $CDCl_3$) of **37**

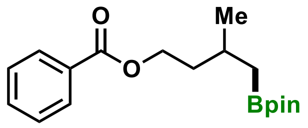

37

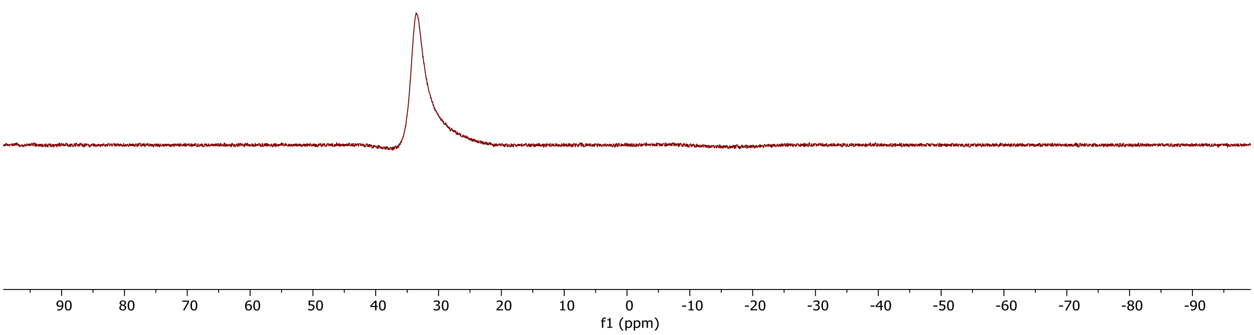

^{13}C NMR (101 MHz, $CDCl_3$) of crude **38** (***see procedure***)

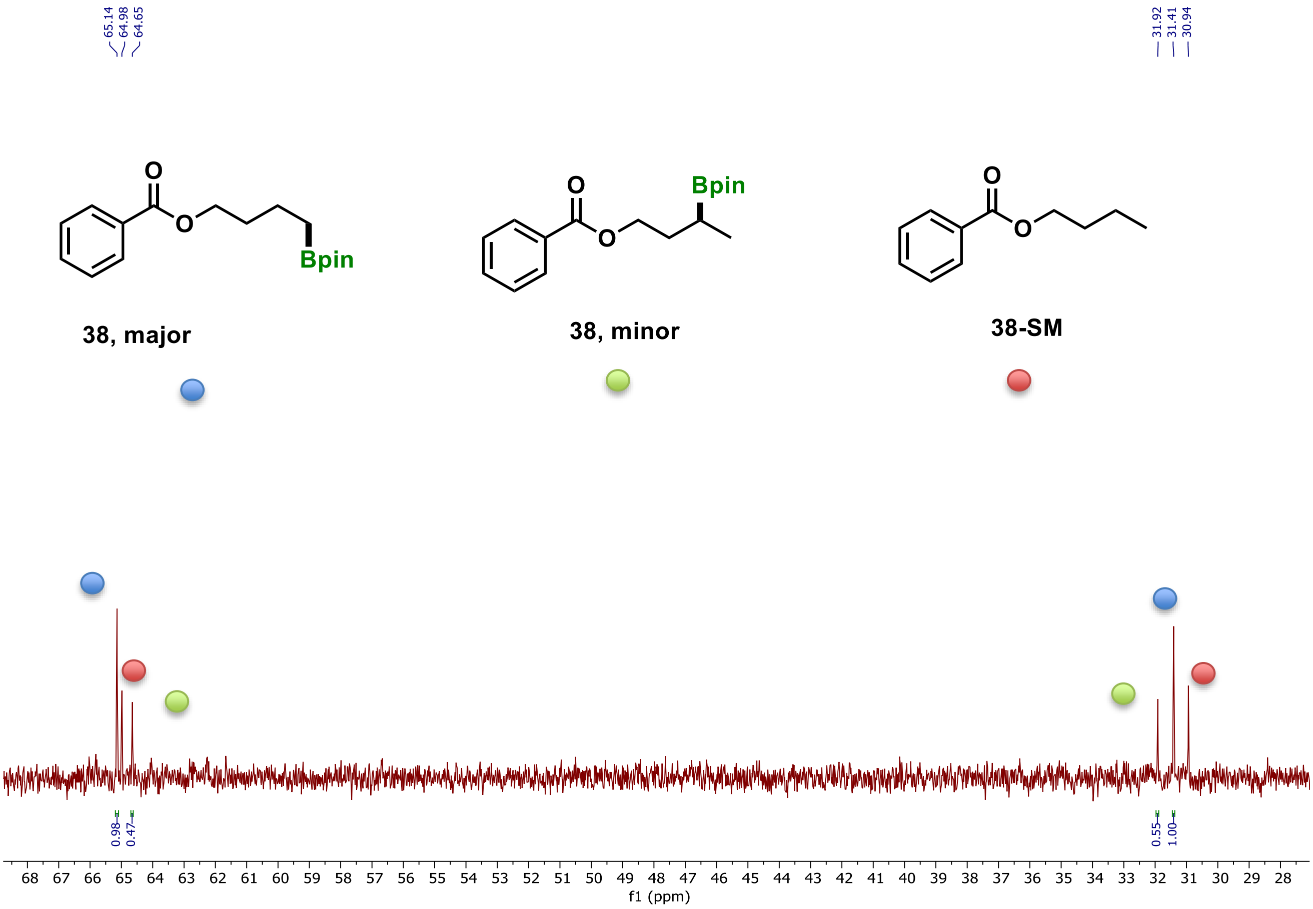

^{1}H NMR (400 MHz, $CDCl_3$) of **38** (*see procedure*)

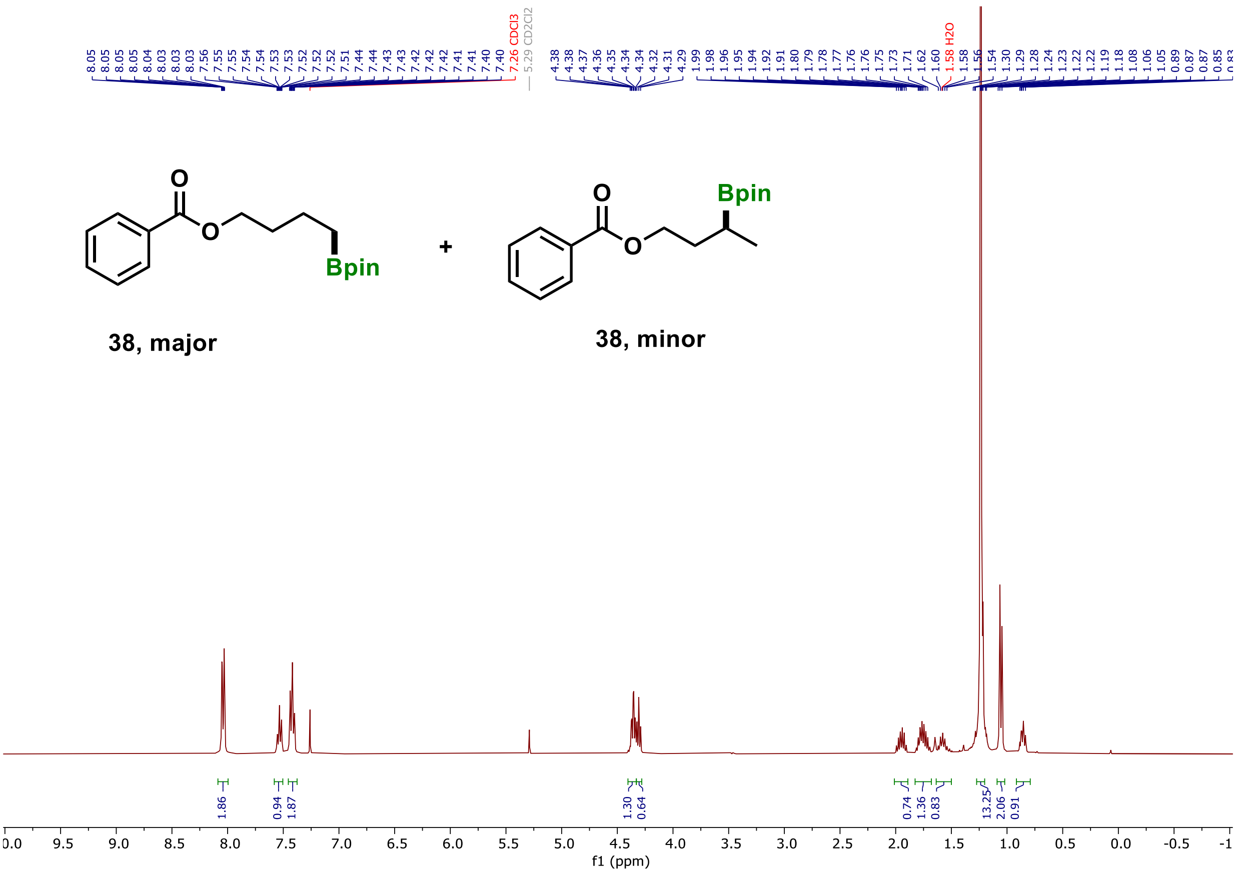

^{13}C NMR (101 MHz, $CDCl_3$) of **38**

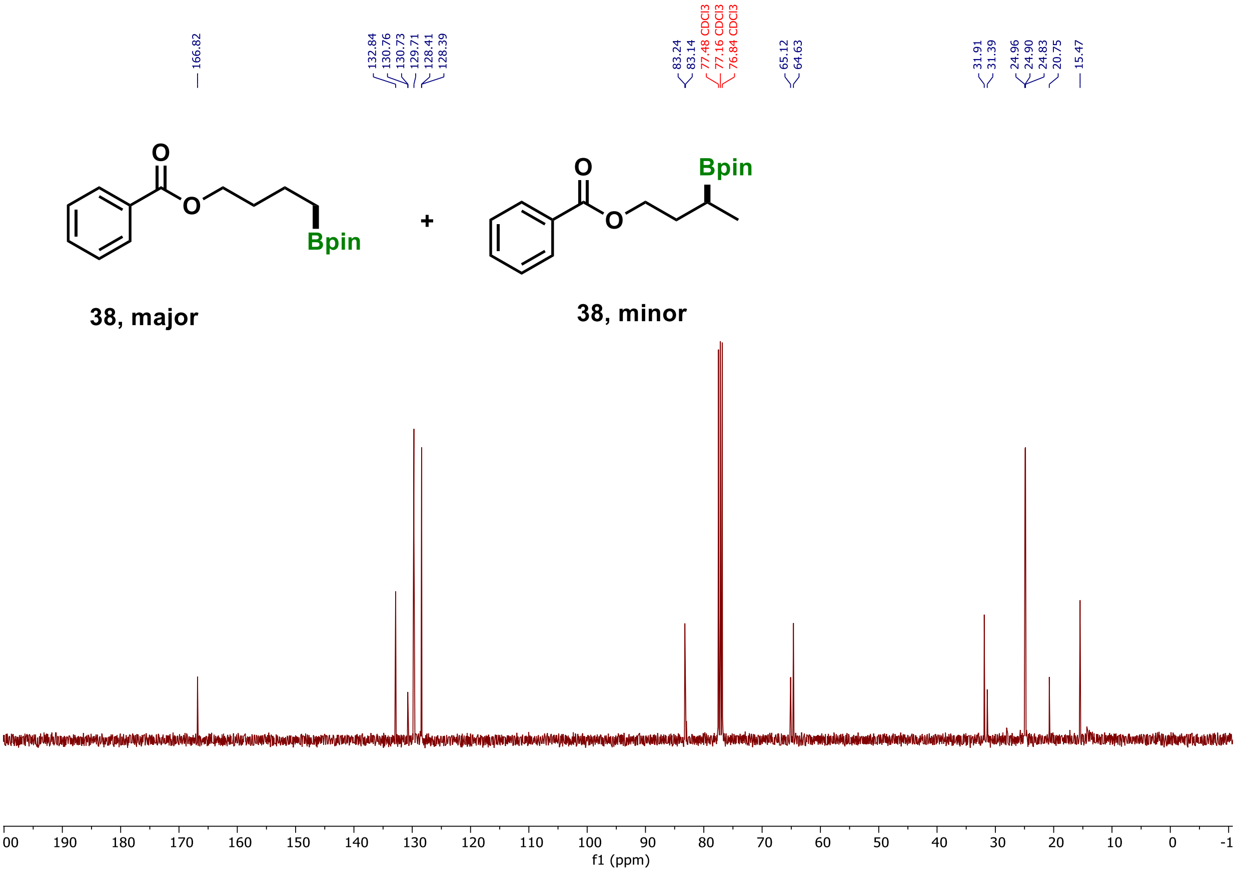

^{11}B NMR (128 MHz, $CDCl_3$) of **38**

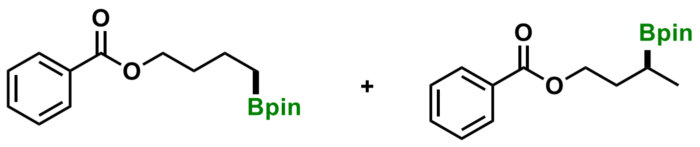

38, major

38, minor

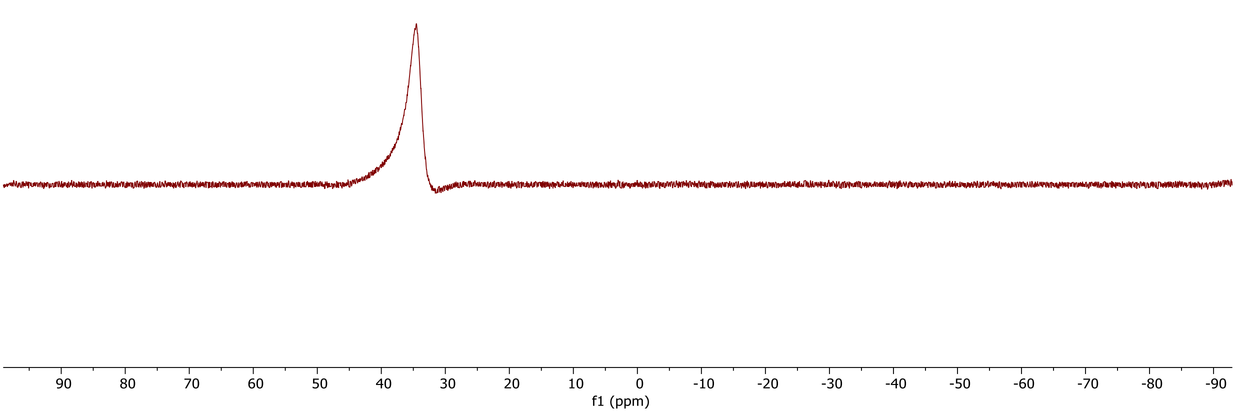

^{1}H NMR (400 MHz, $CDCl_3$) of **39-S** (*see procedure*)

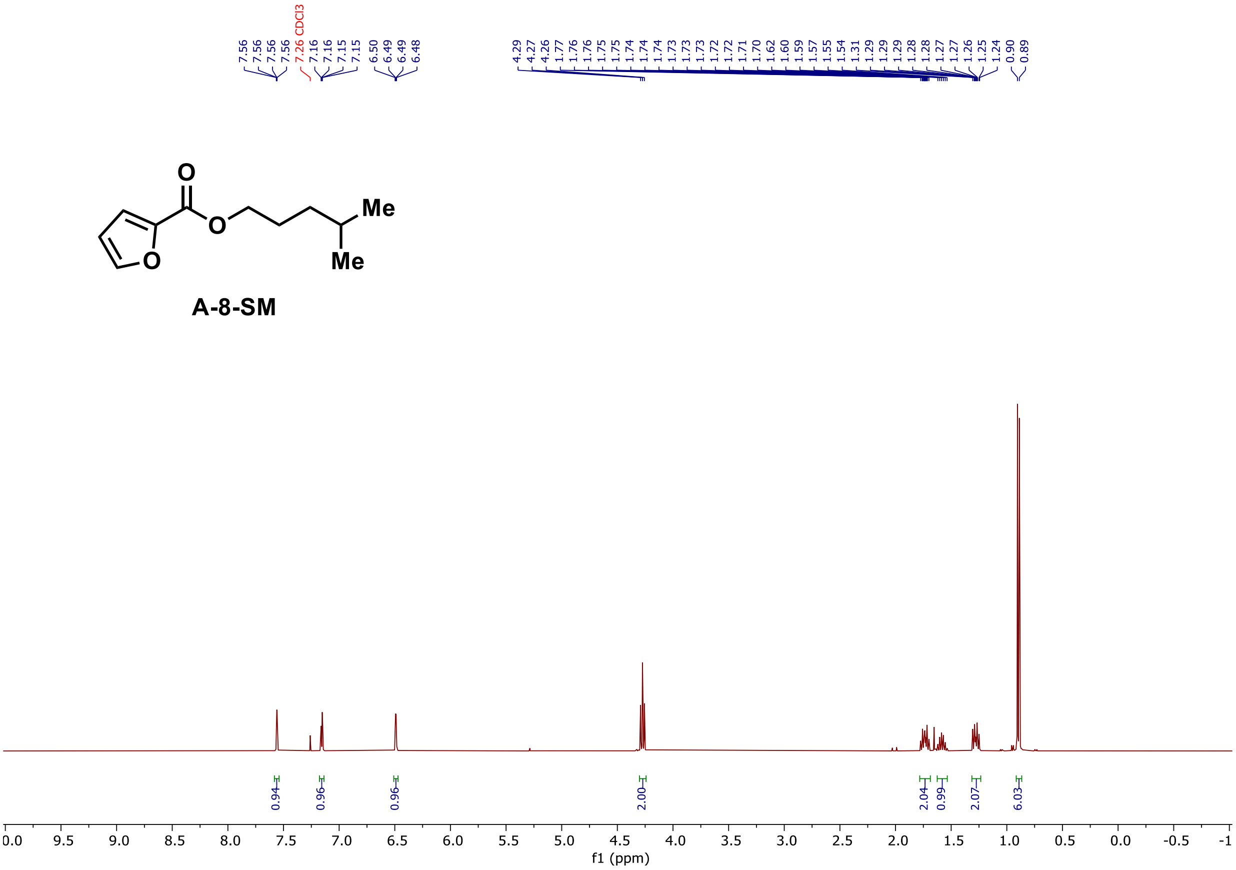

^{13}C NMR (101 MHz, $CDCl_3$) of **39-S**

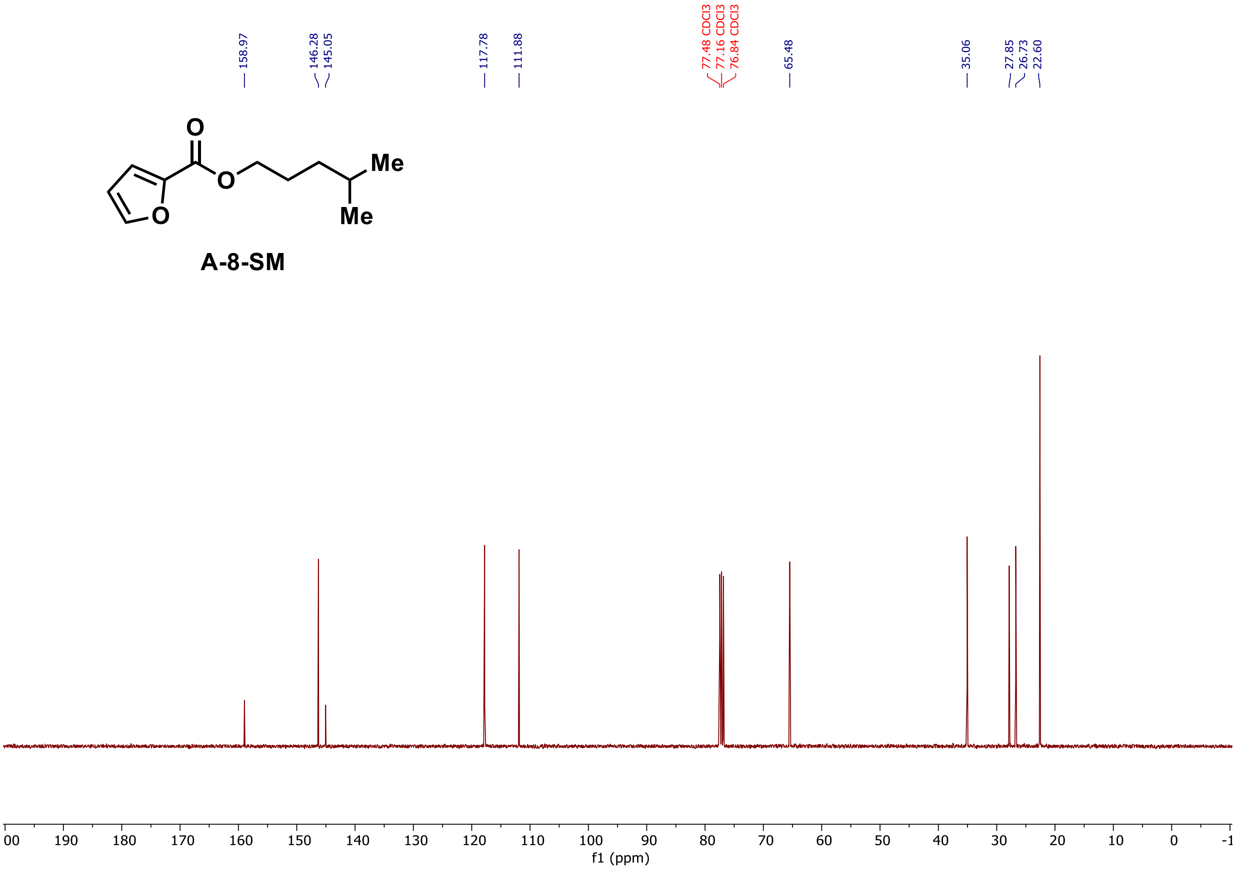

^{1}H NMR (400 MHz, $CDCl_3$) of **39** (*see procedure*)

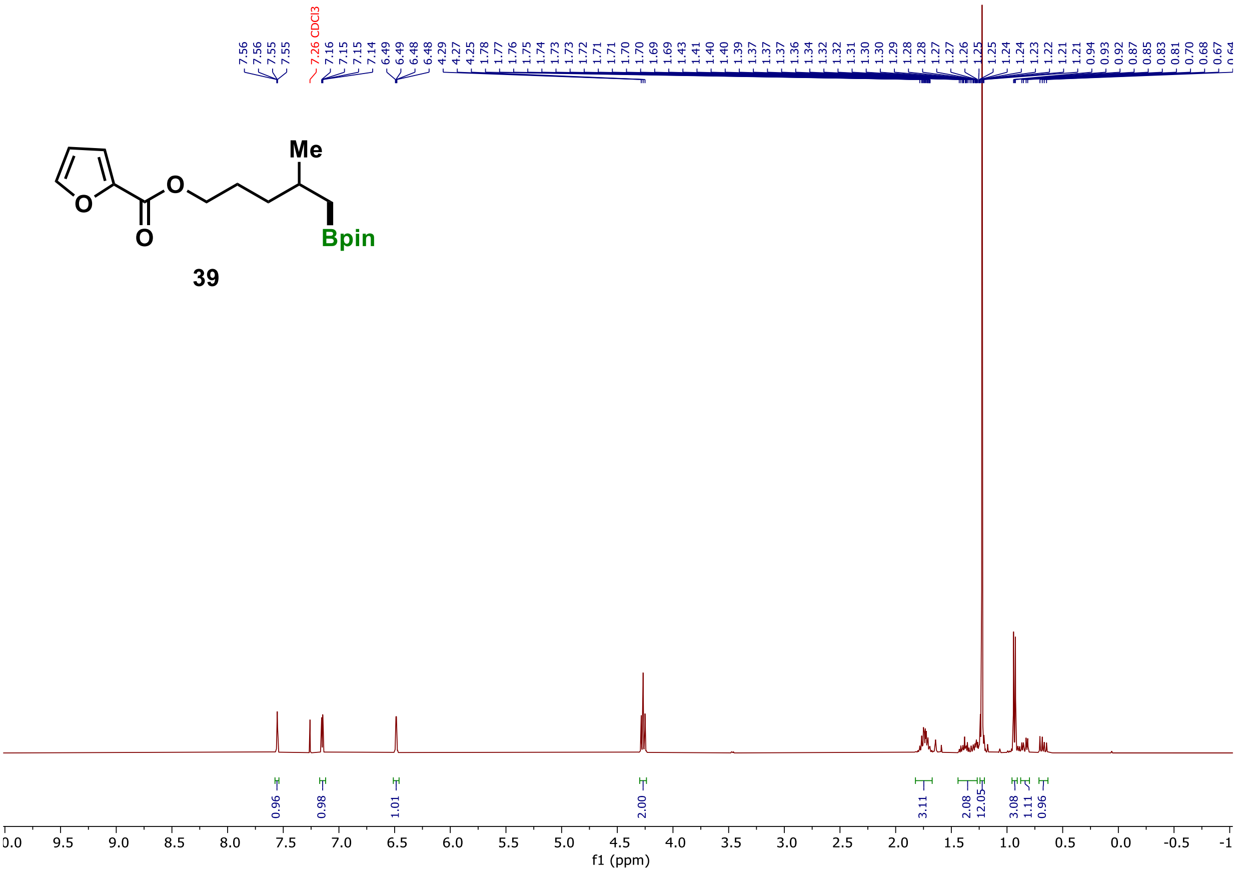

^{13}C NMR (101 MHz, $CDCl_3$) of **39**

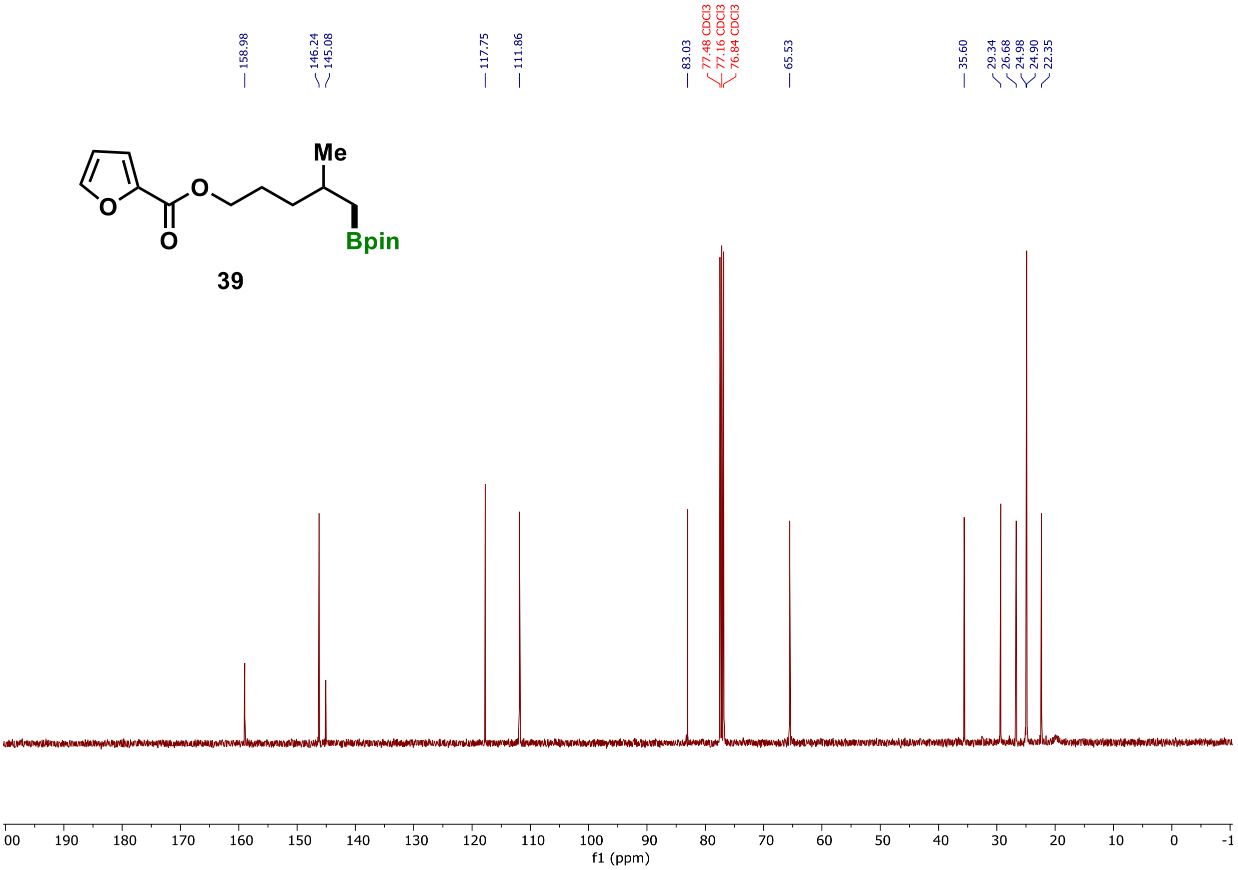

^{1}H NMR (400 MHz, $CDCl_3$) of **40-S** (*see procedure*)

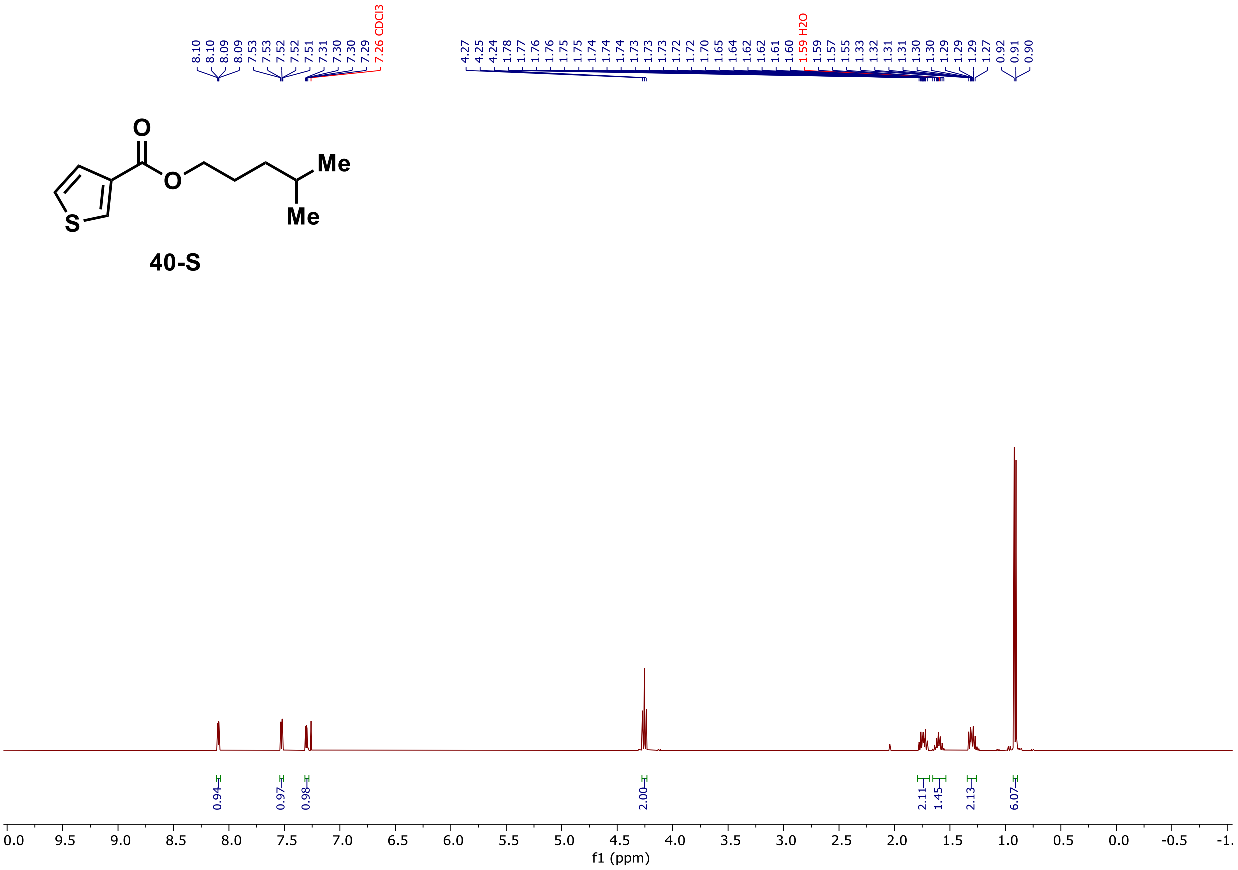

^{13}C NMR (101 MHz, $CDCl_3$) of **40-S**

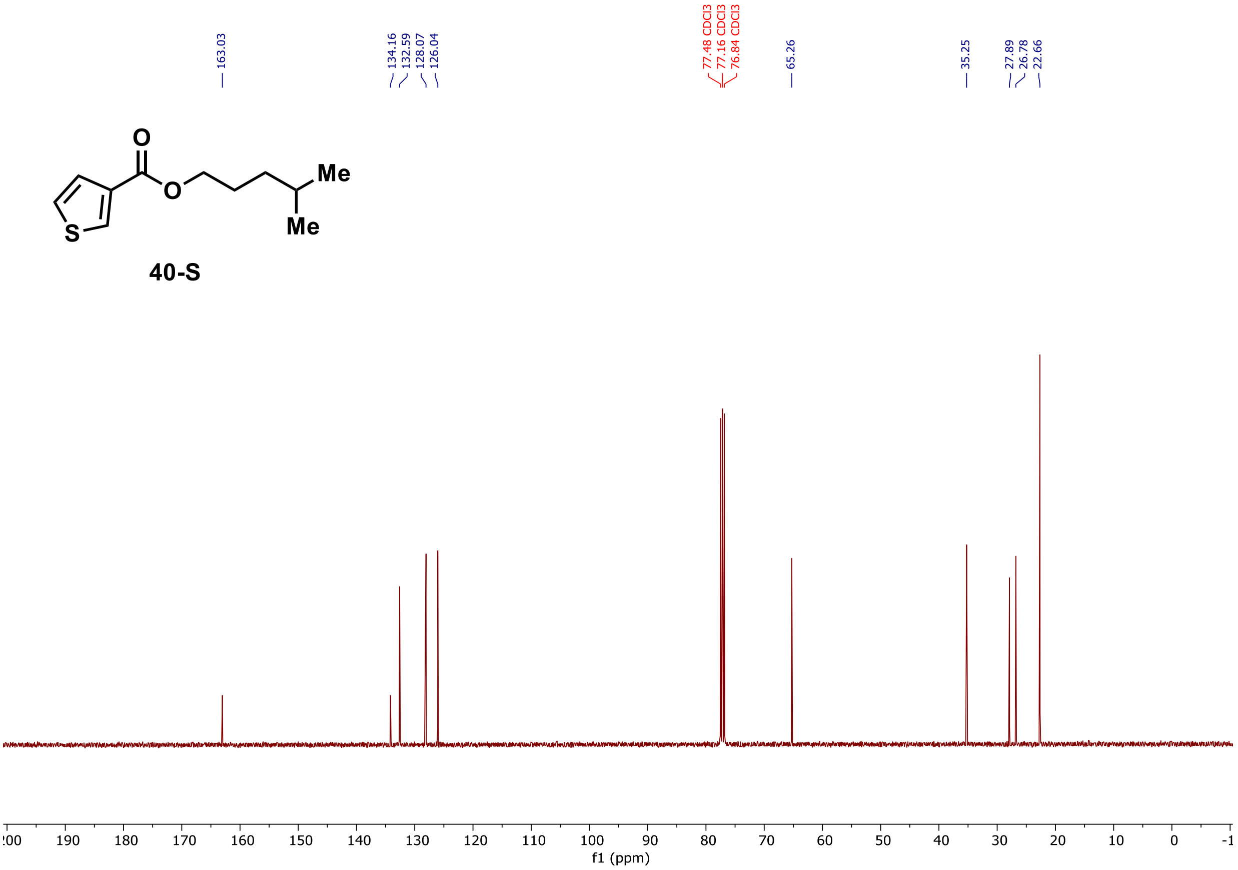

^{1}H NMR (400 MHz, $CDCl_3$) of **40** (*see procedure*)

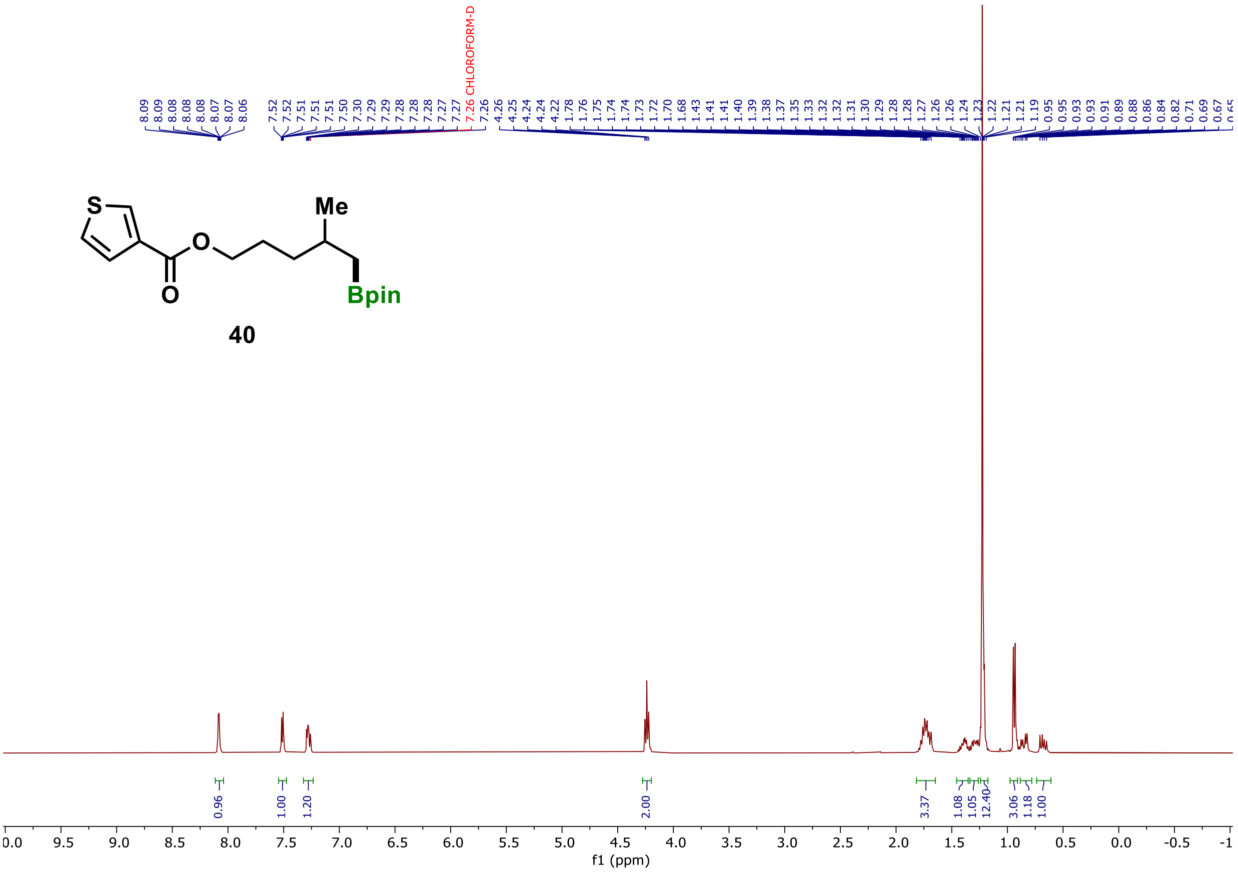

^{13}C NMR (101 MHz, $CDCl_3$) of **40**

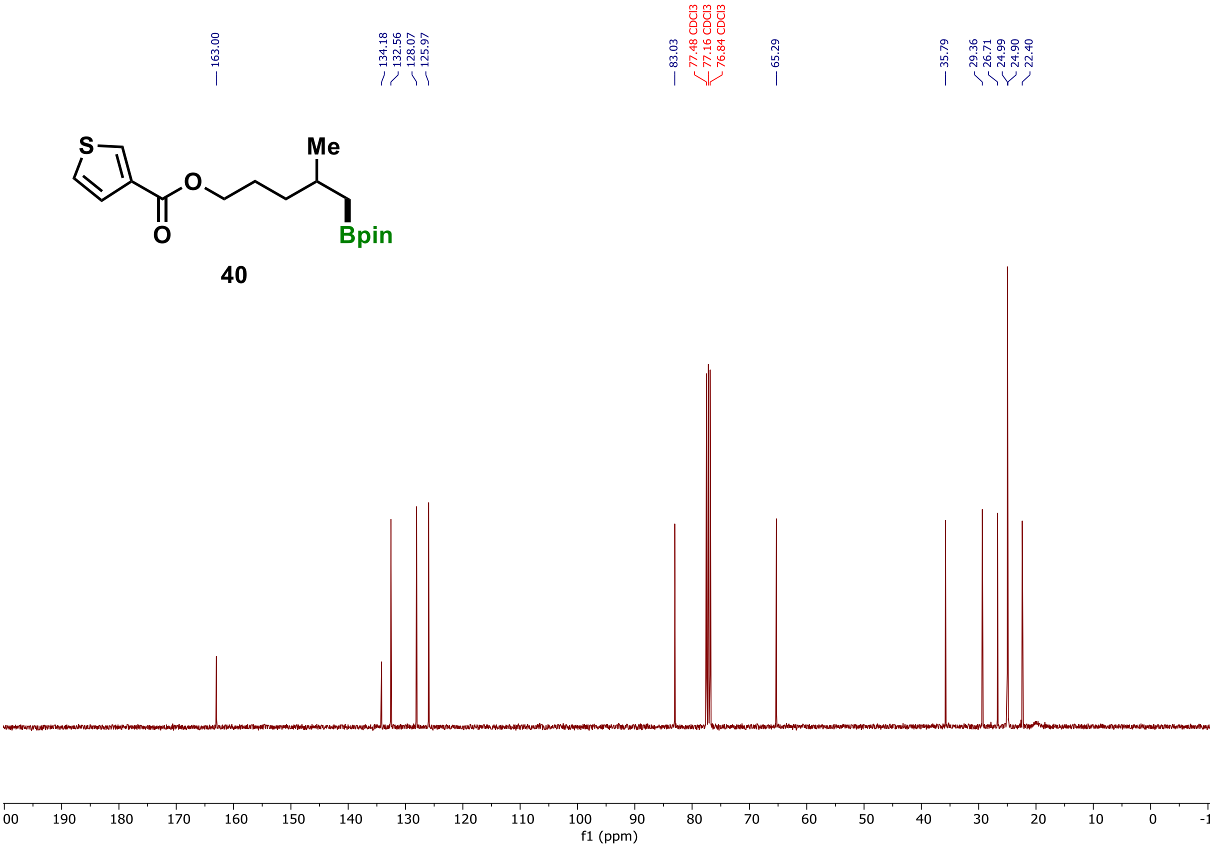

^{1}H NMR (400 MHz, CDCl3) of **41b-ol** (*see procedure*)

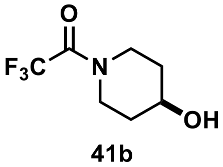

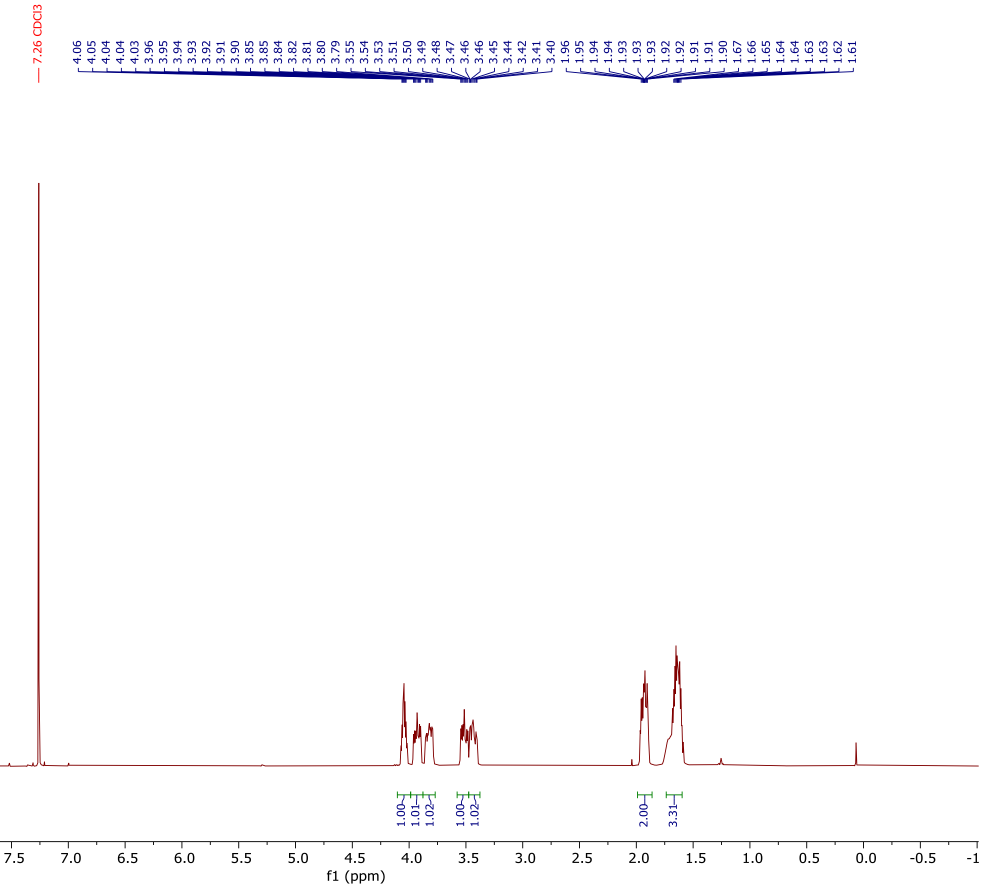

^{13}C NMR (101 MHz, CDCl3) of **41b-ol**

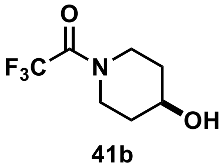

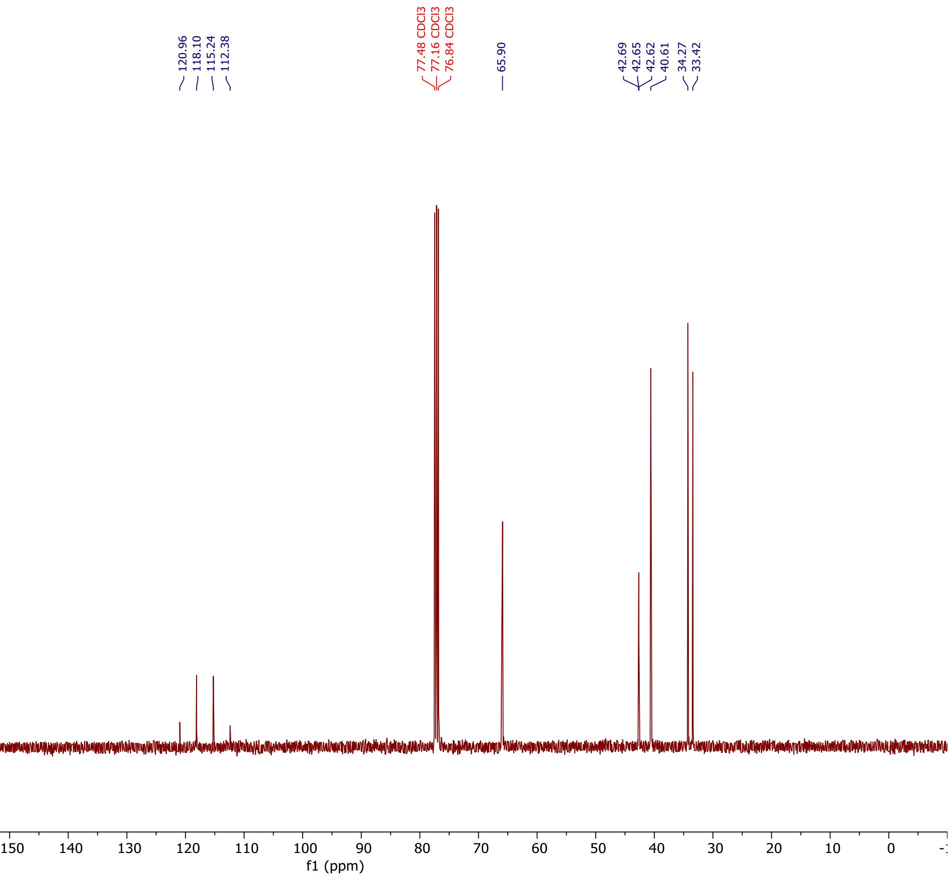

^{19}F NMR (377 MHz, $CDCl_3$) of **41b-ol**

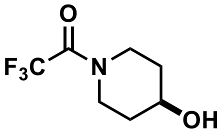

41b

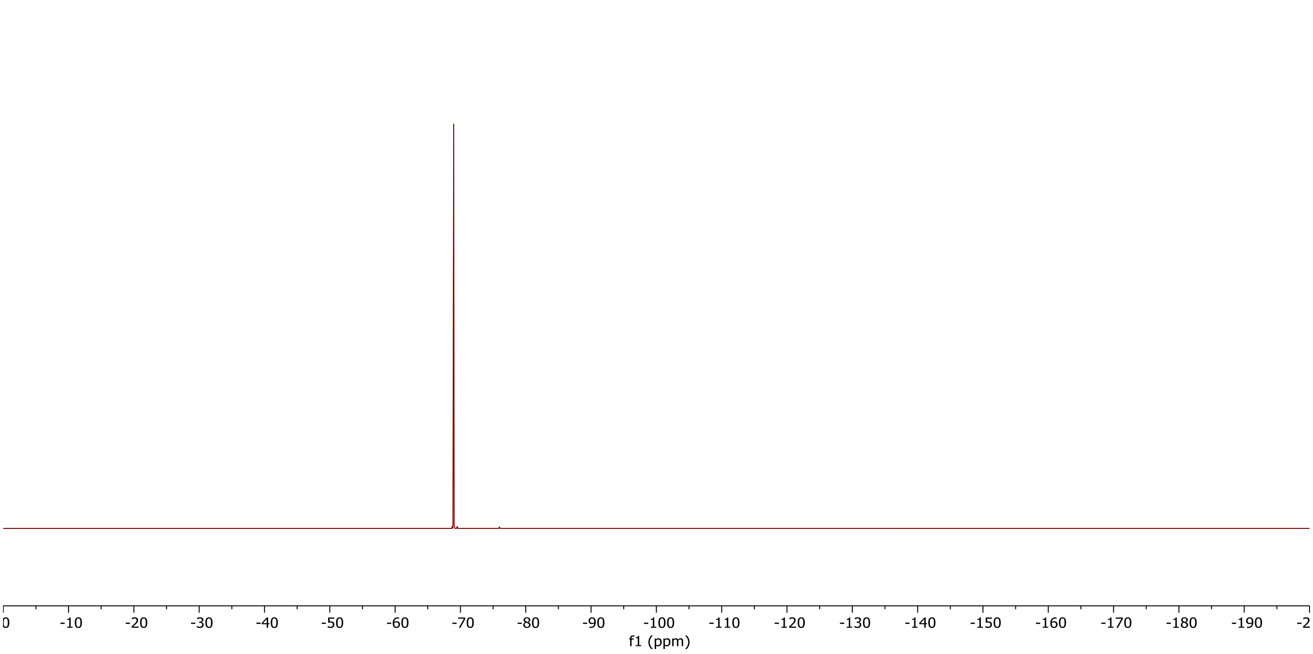

^{1}H NMR (400 MHz, $CDCl_3$) of **41a-ol** (*see procedure*)

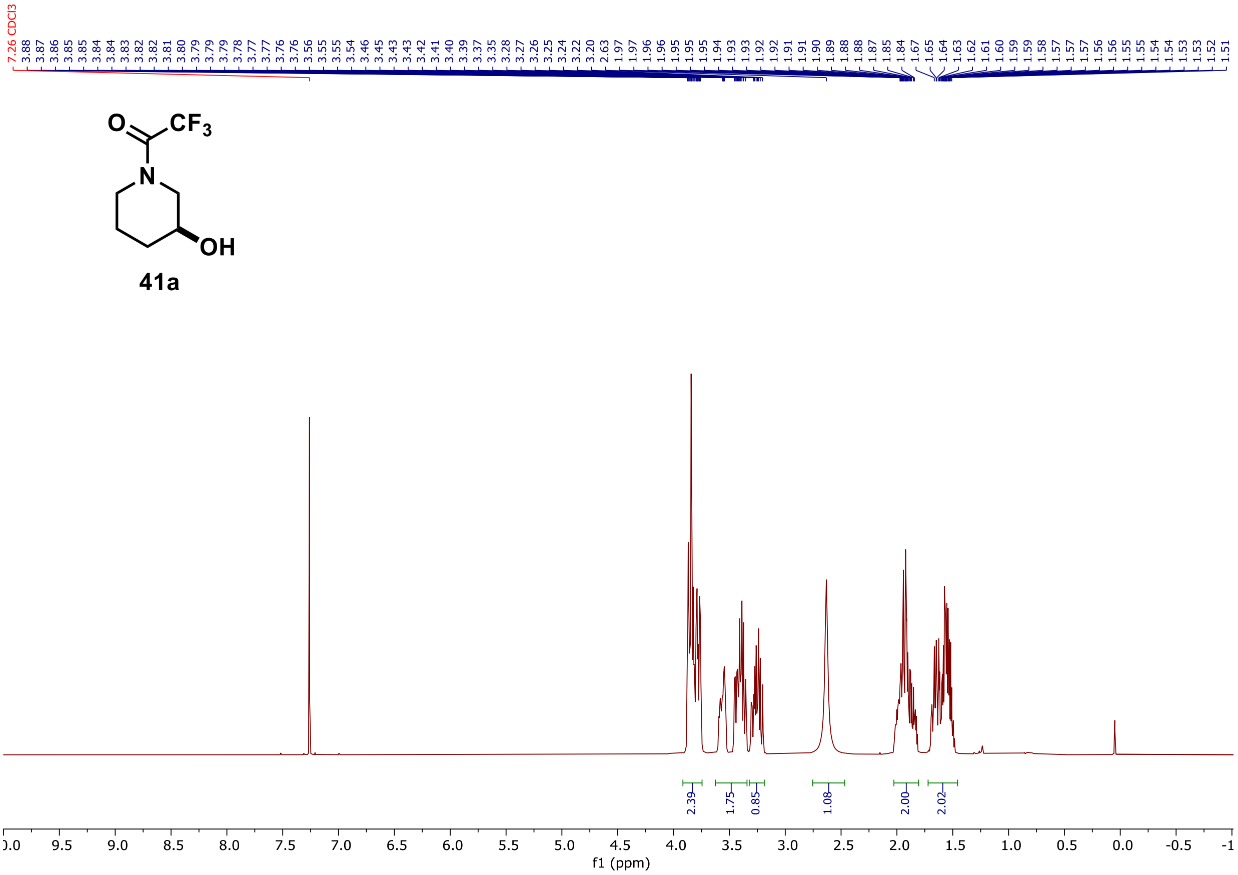

41a

^{13}C NMR (101 MHz, $CDCl_3$) of **41a-ol**

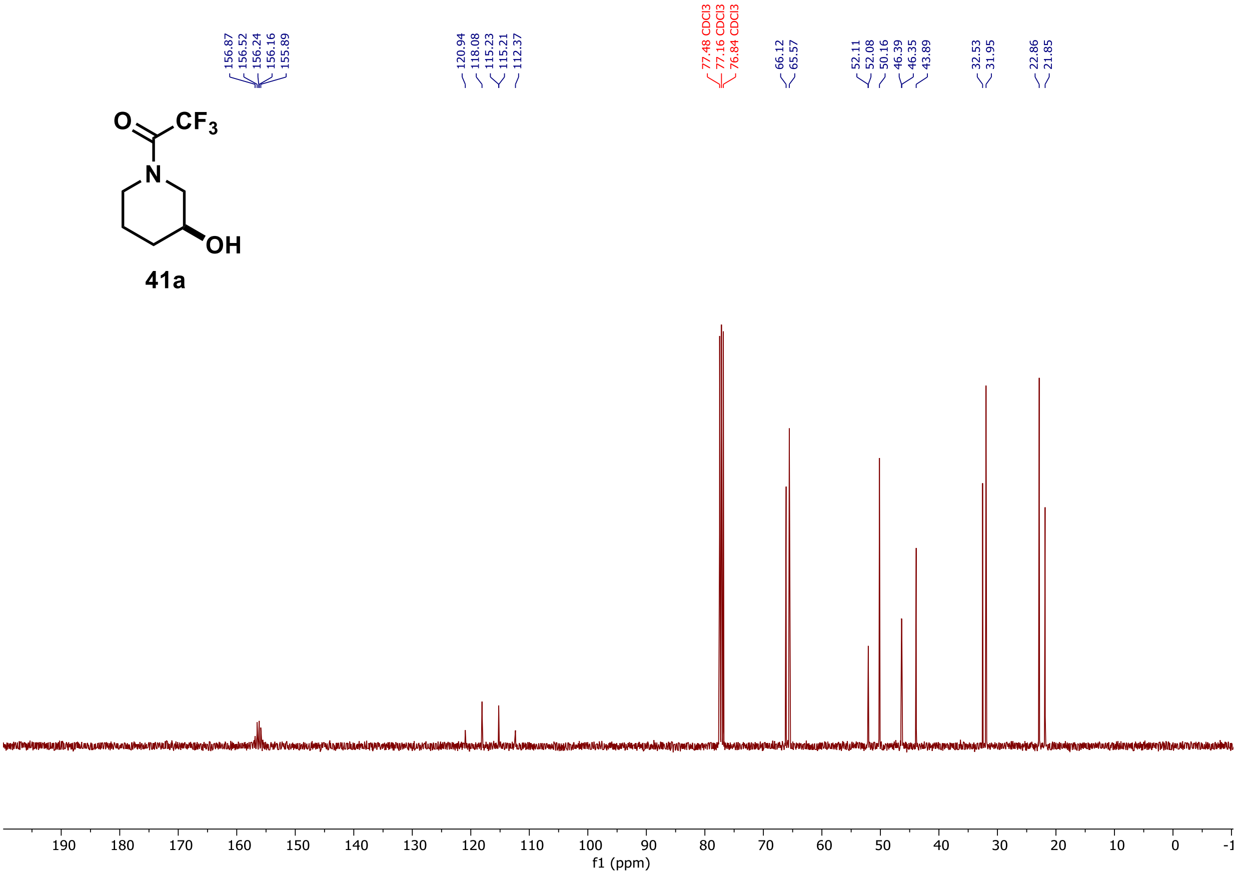

41a

^{19}F NMR (377 MHz, $CDCl_3$) of **41a-ol**

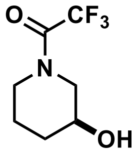

41a

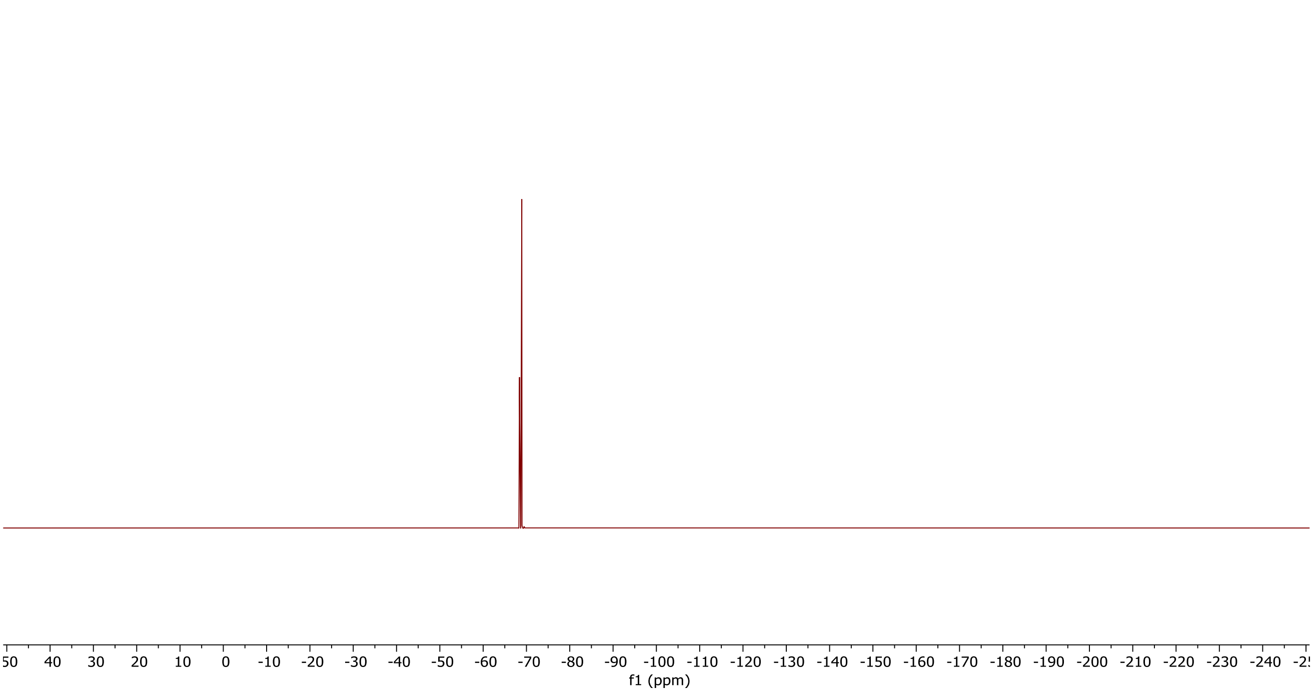

^{1}H NMR (400 MHz, $CDCl_3$) of **42** (***see procedure***)

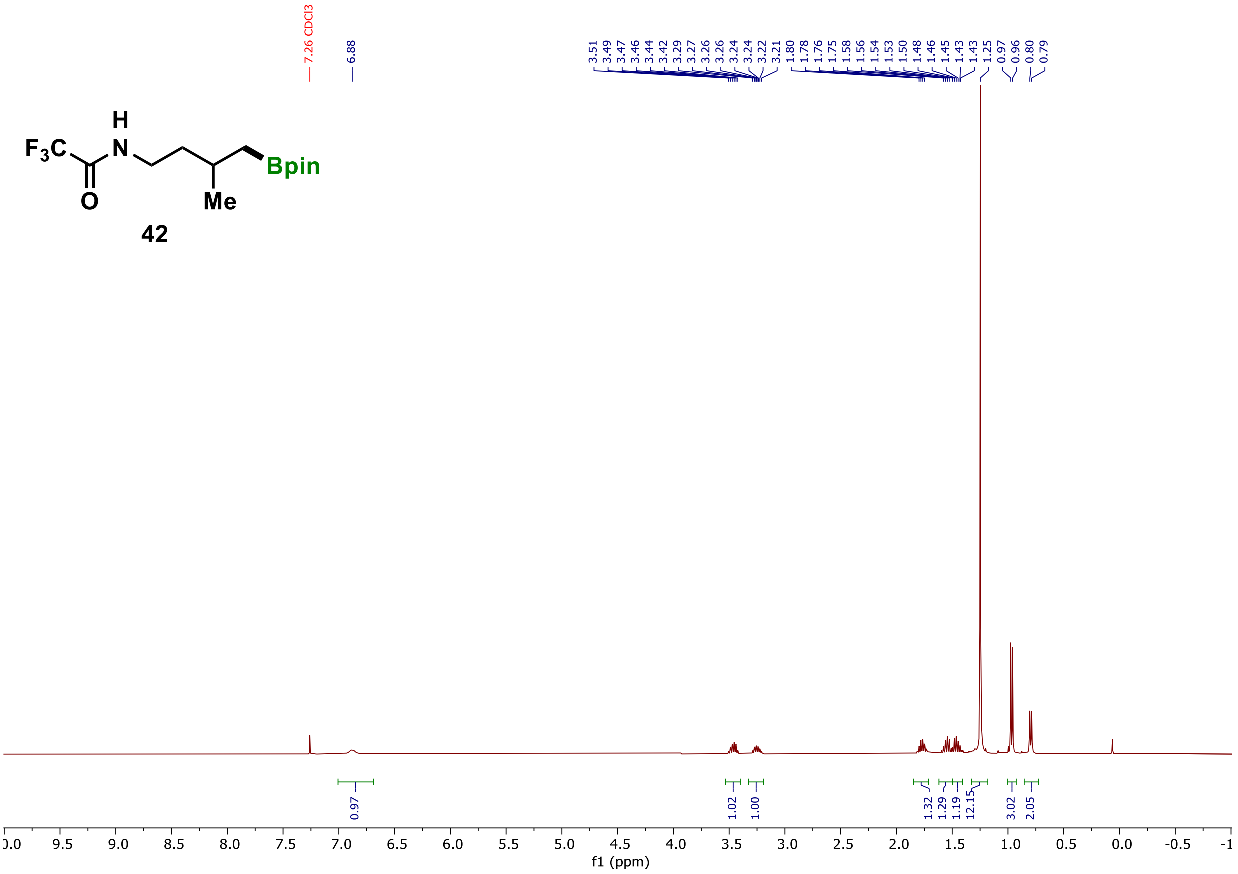

^{13}C NMR (101 MHz, $CDCl_3$) of **42**

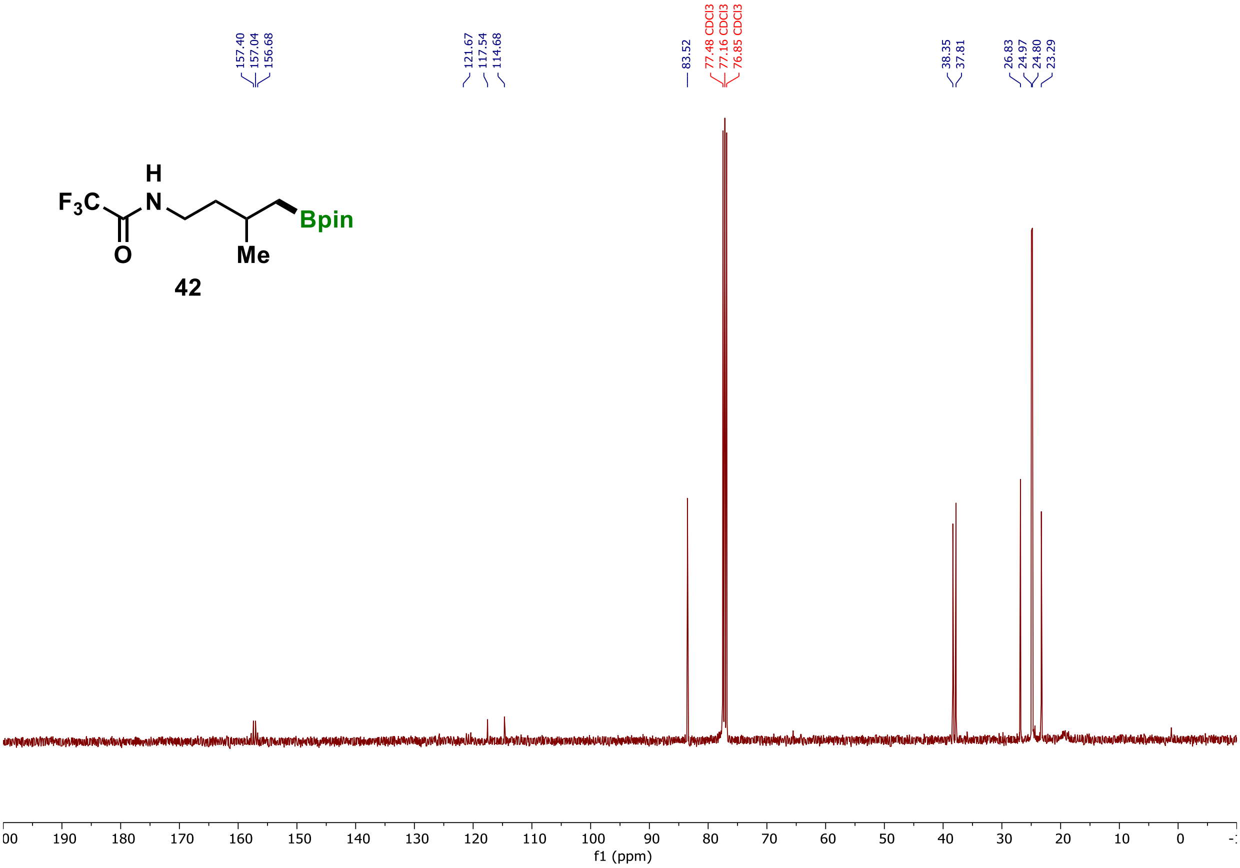

^{19}F NMR (377 MHz, $CDCl_3$) of **42**

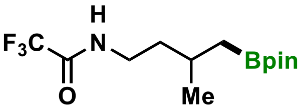

42

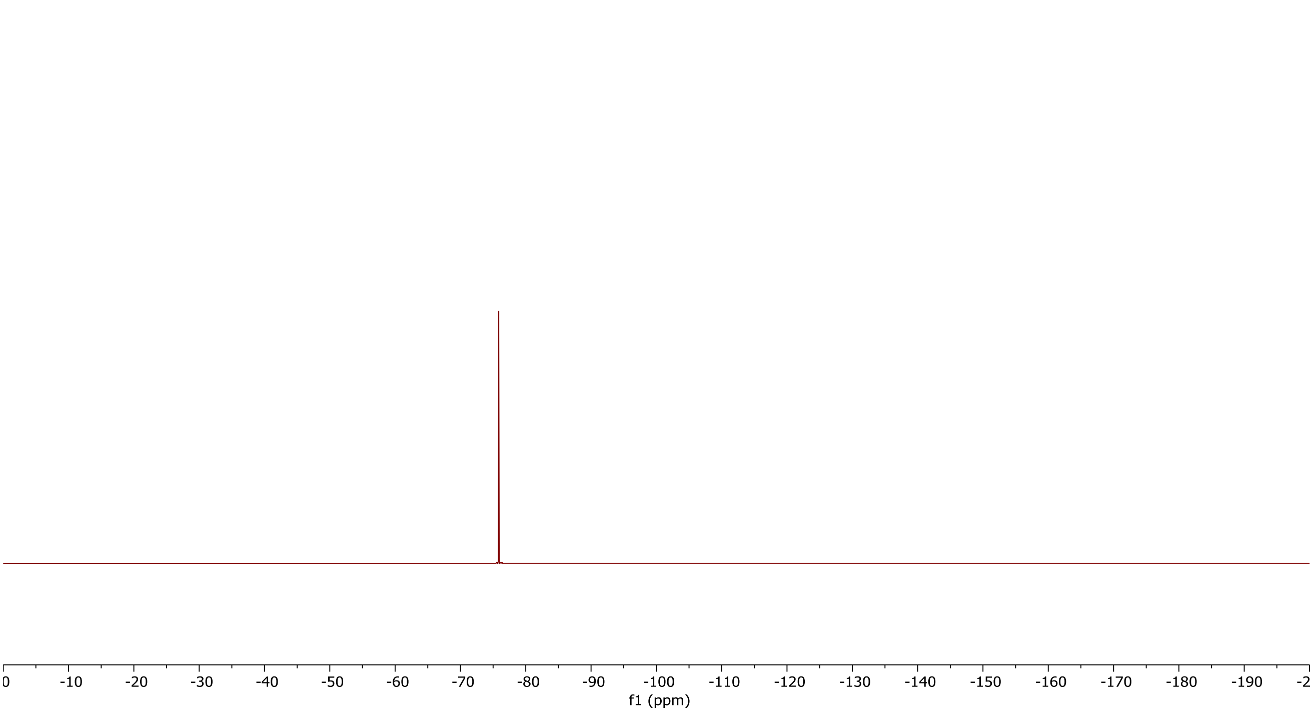

^{1}H NMR (400 MHz, CDCl$_3$) of **43-S** (***see procedure***)

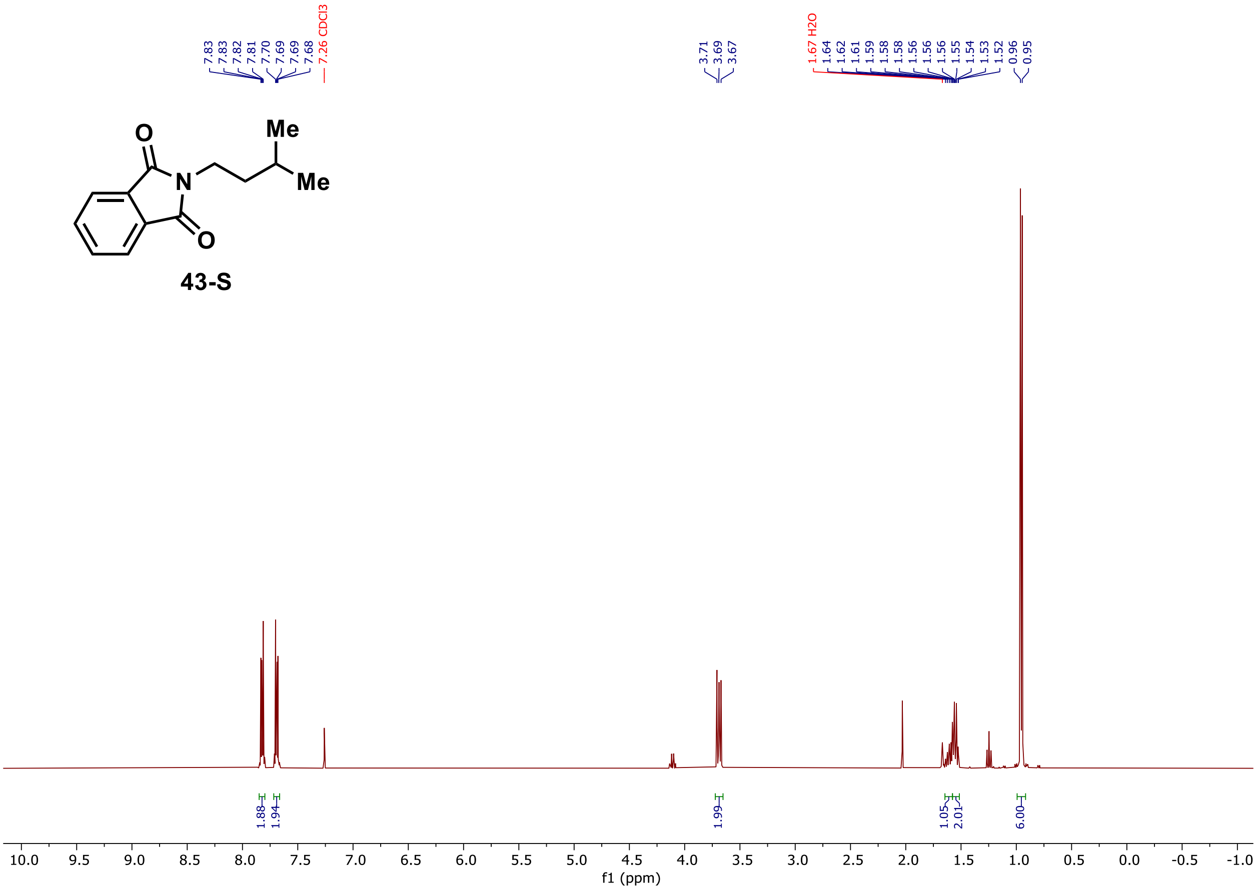

^{13}C NMR (101 MHz, CDCl$_3$) of **43-S**

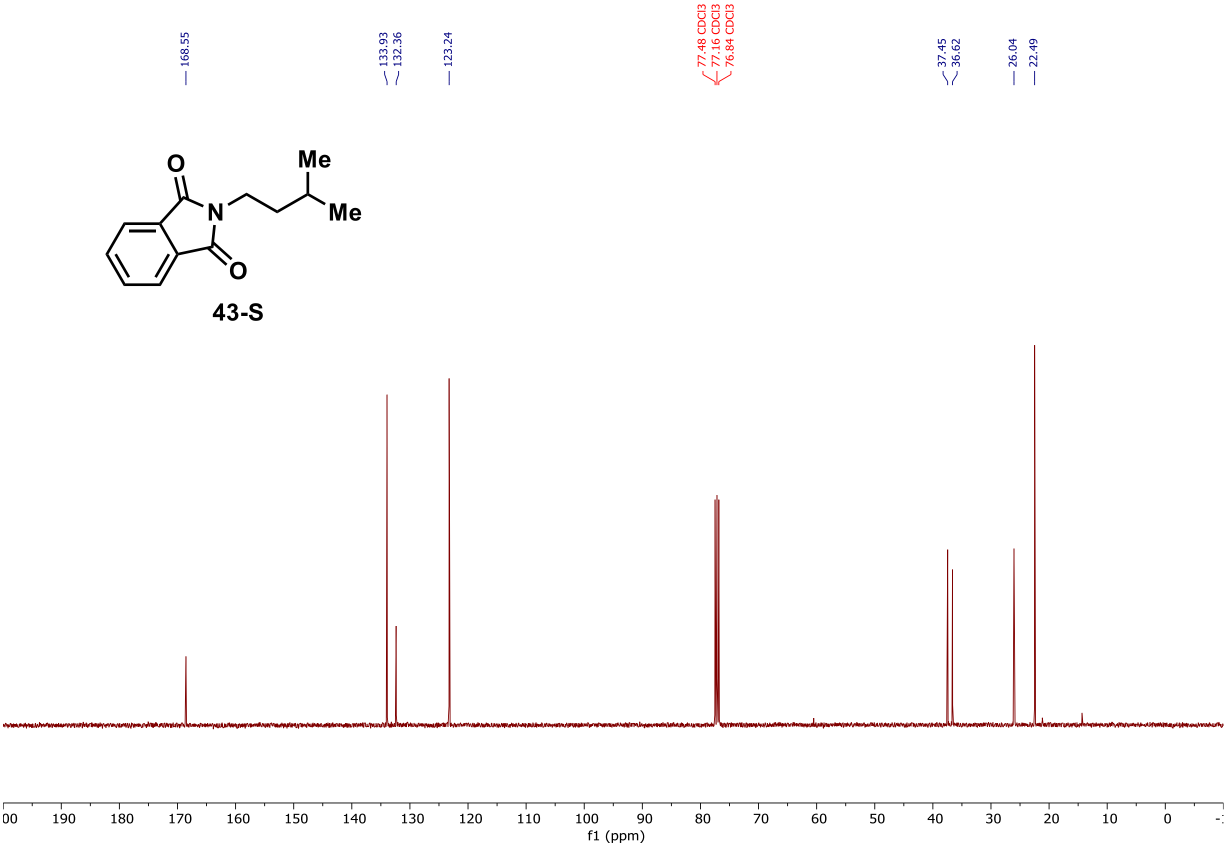

^{1}H NMR (400 MHz, $CDCl_3$) of **43** (*see procedure*)

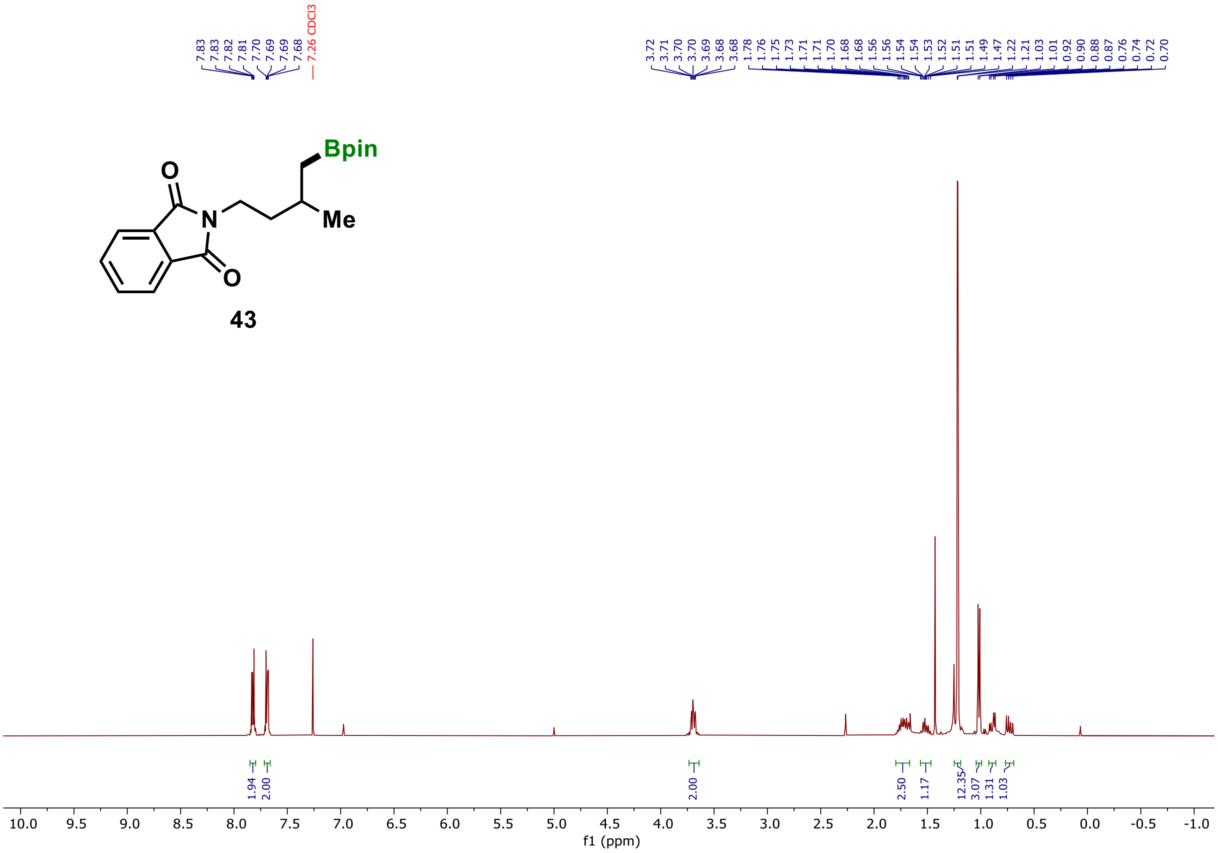

^{13}C NMR (101 MHz, $CDCl_3$) of **43**

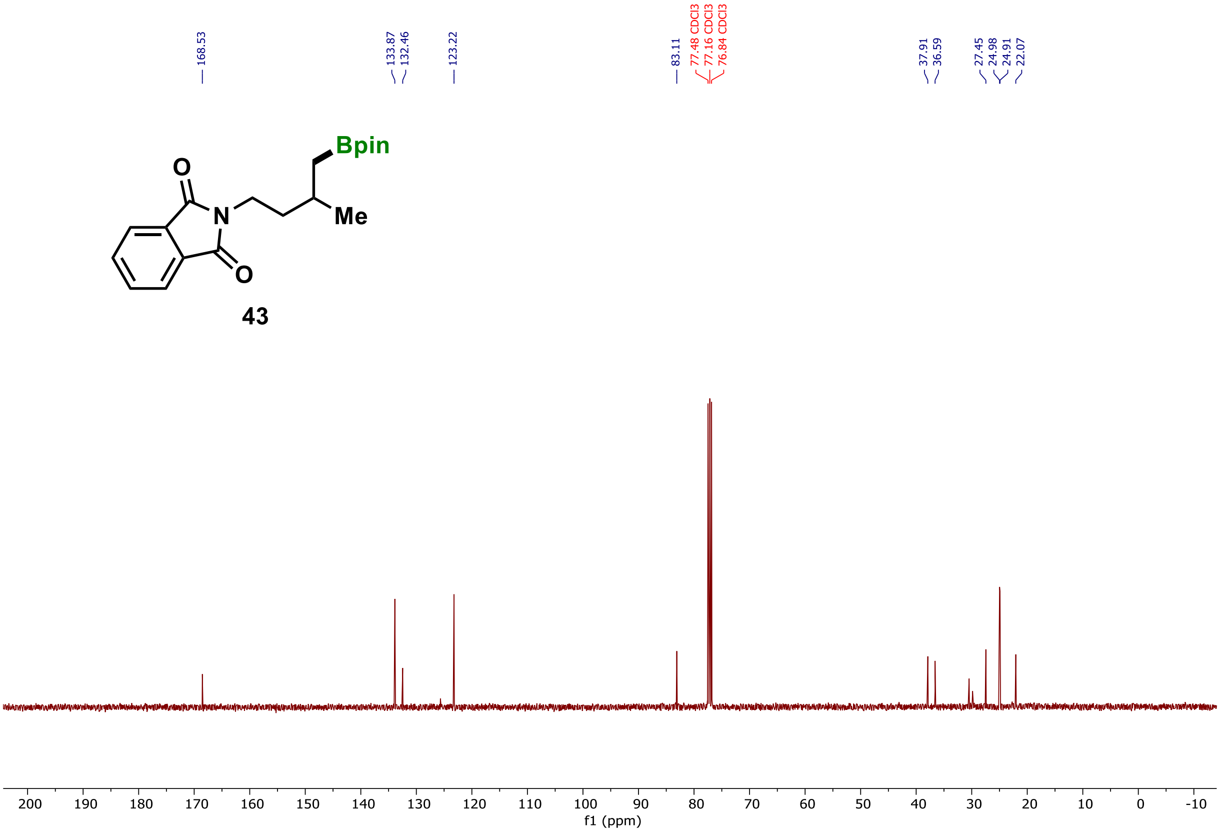

^{11}B NMR (128 MHz, $CDCl_3$) of **43**

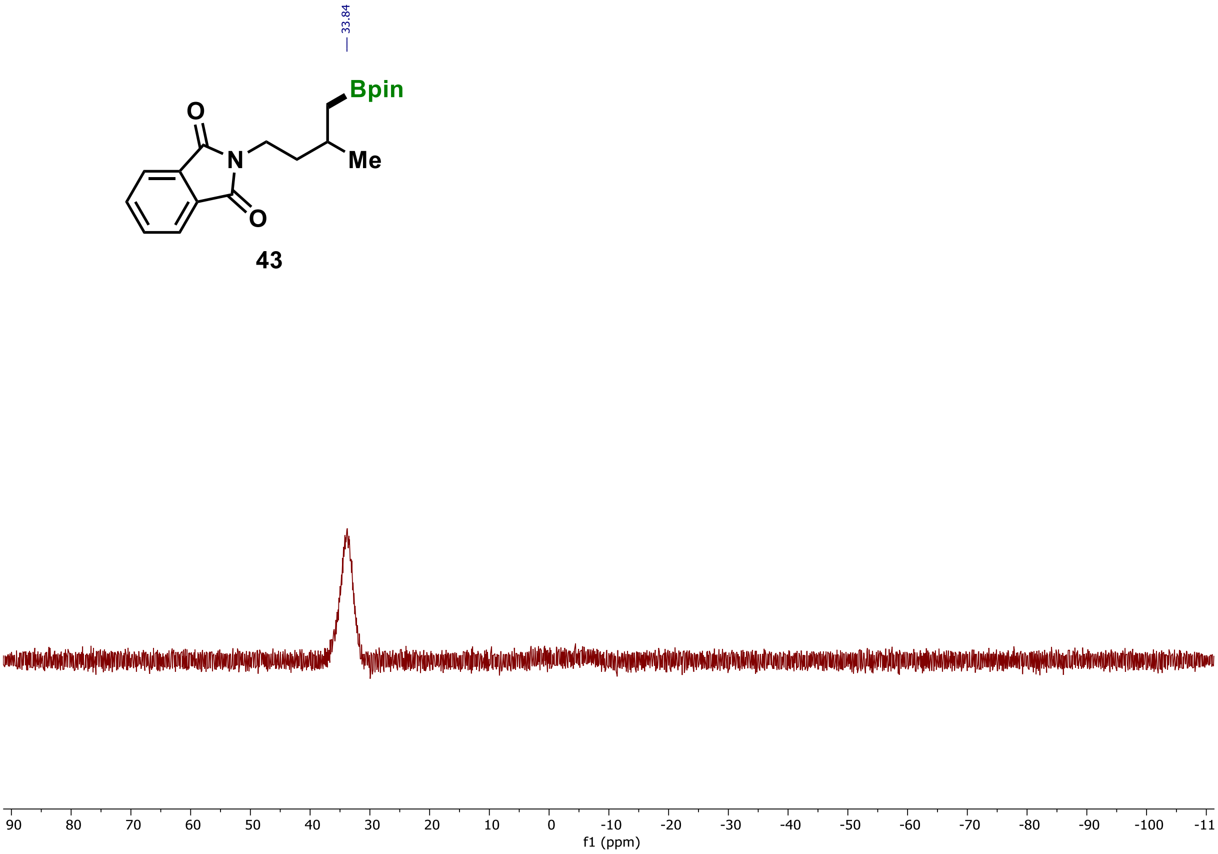

^{1}H NMR (400 MHz, $CDCl_3$) of **44** (*see procedure*)

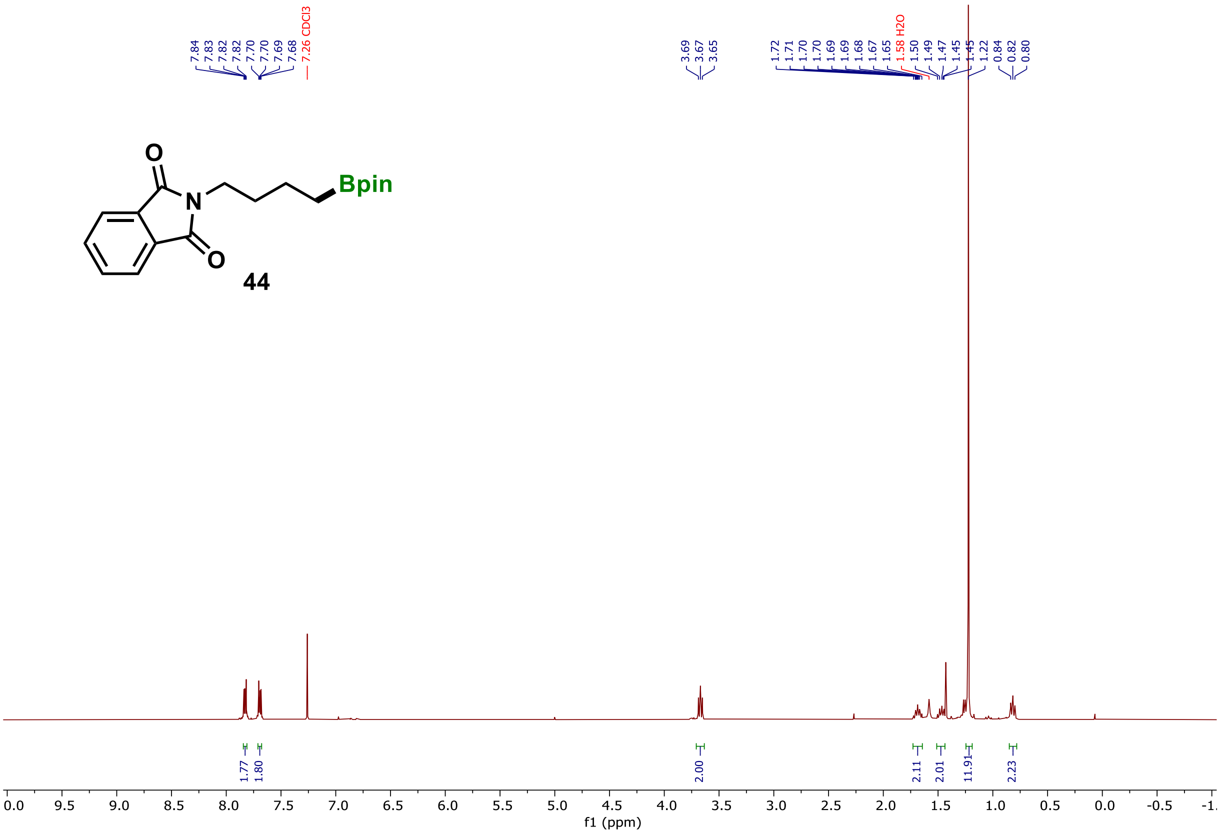

^{13}C NMR (101 MHz, $CDCl_3$) of **44**

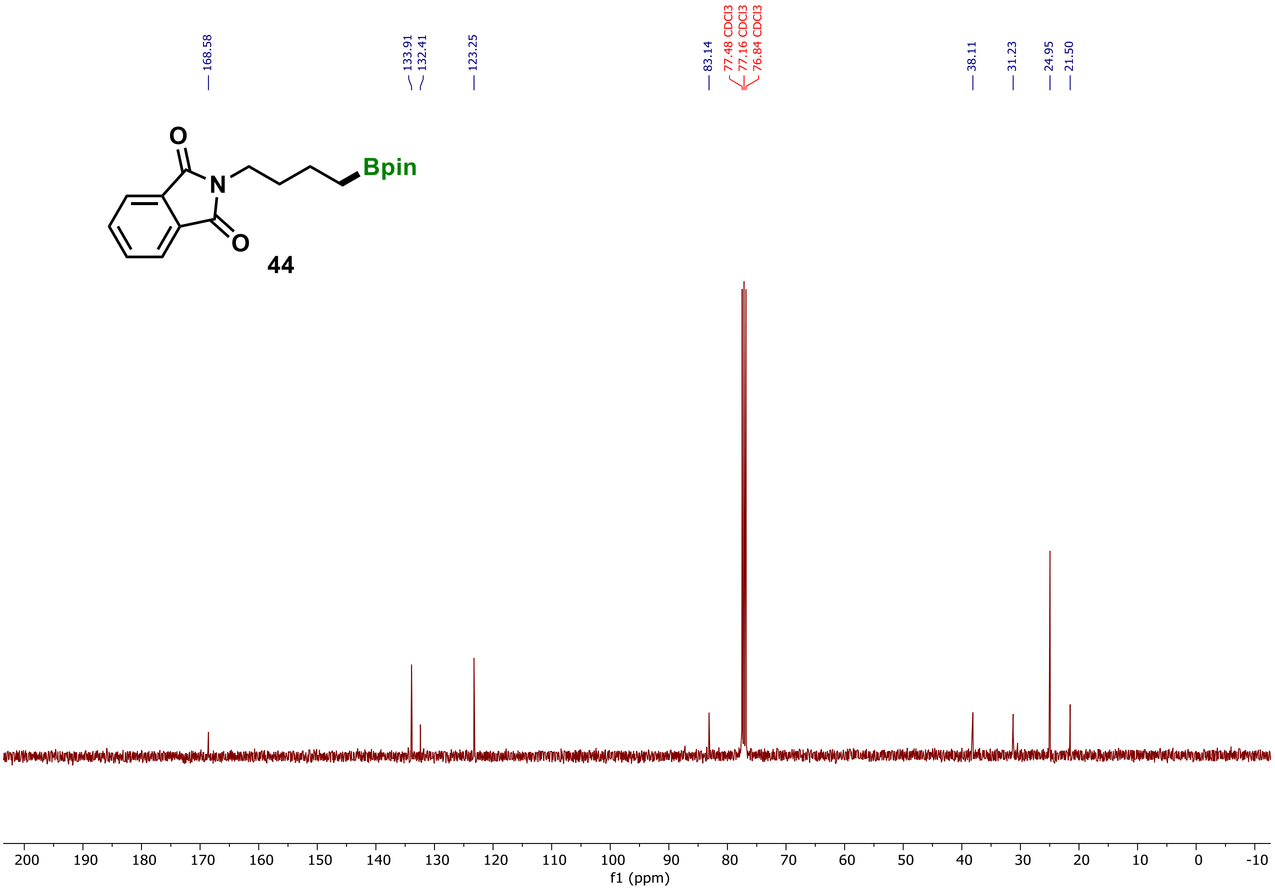

^{1}H NMR (400 MHz, $CDCl_3$) of **45** (*see procedure*)

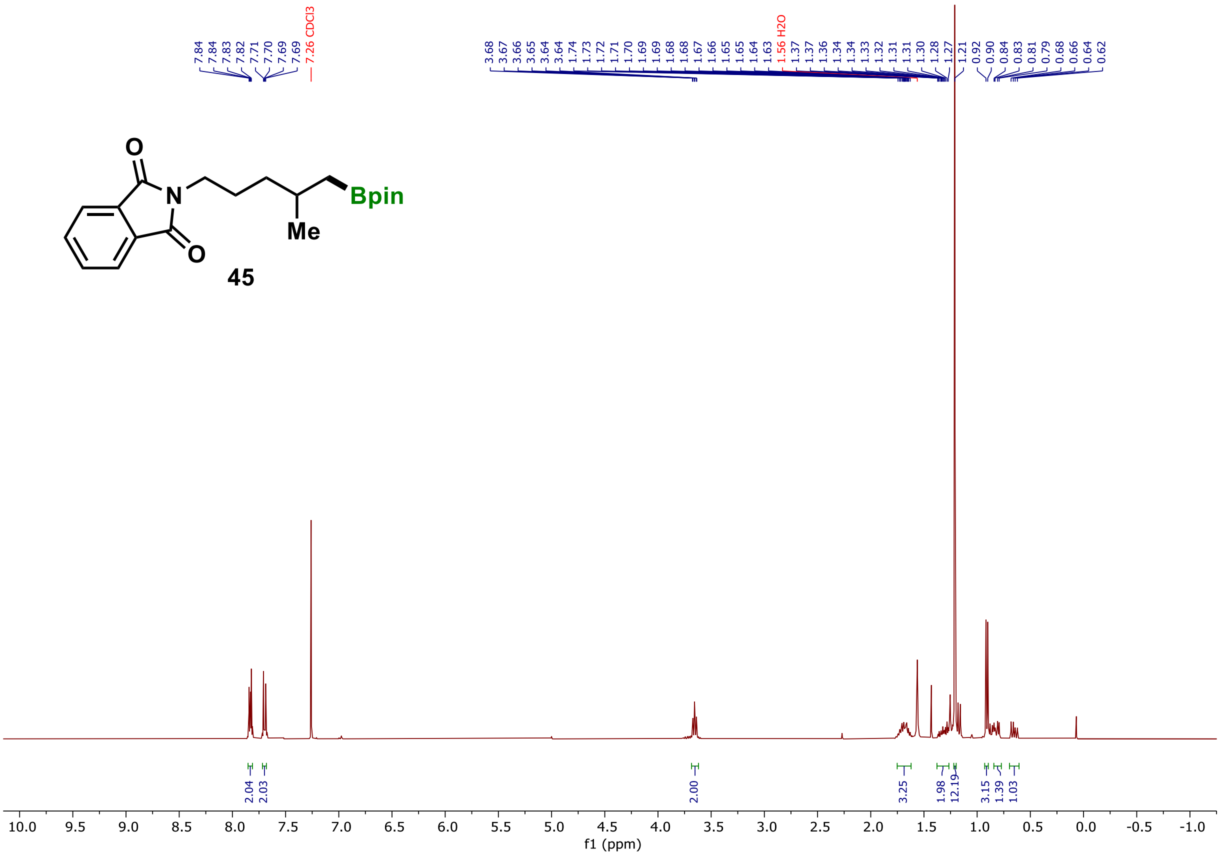

^{13}C NMR (101 MHz, $CDCl_3$) of **45**

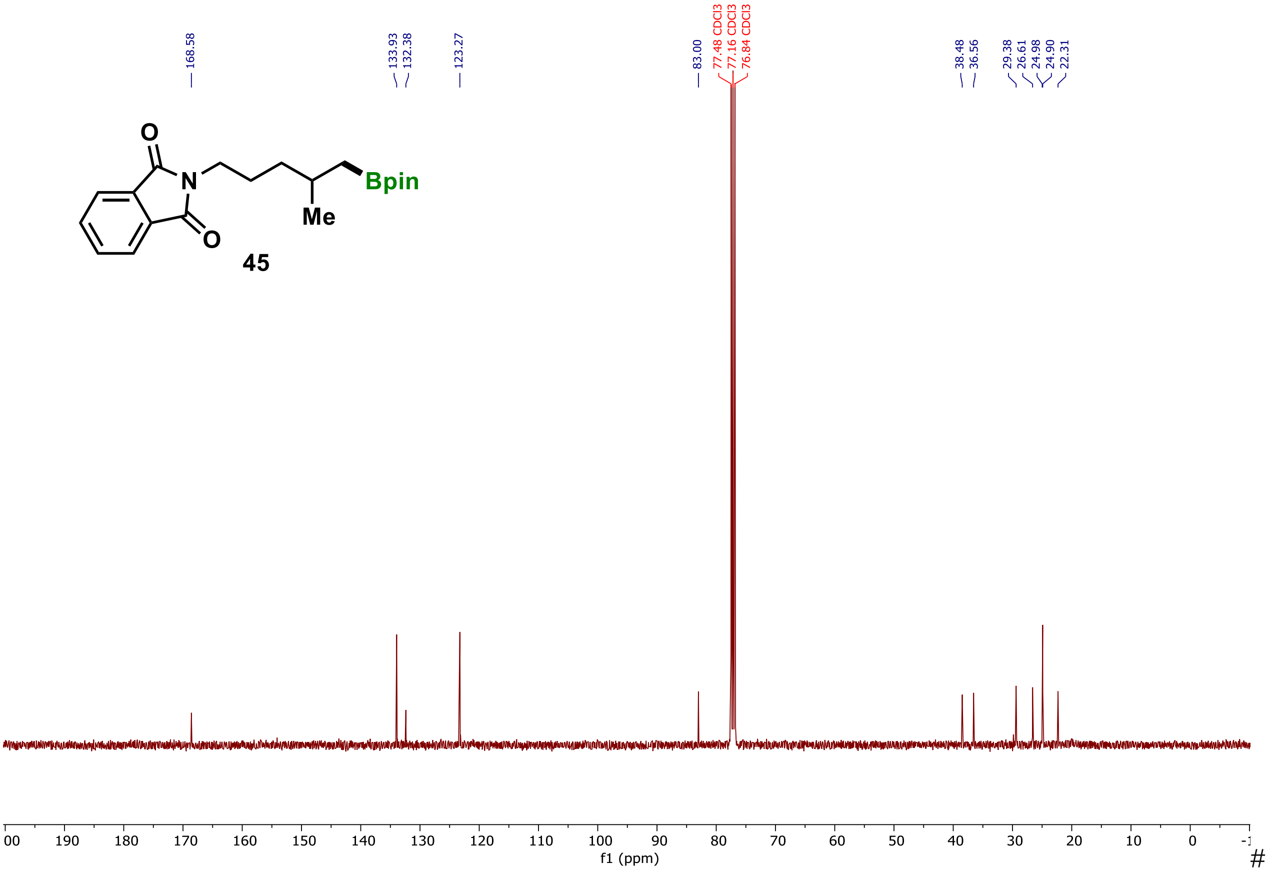

^{1}H NMR (400 MHz, $CDCl_3$) of **46-S** (*see procedure*)

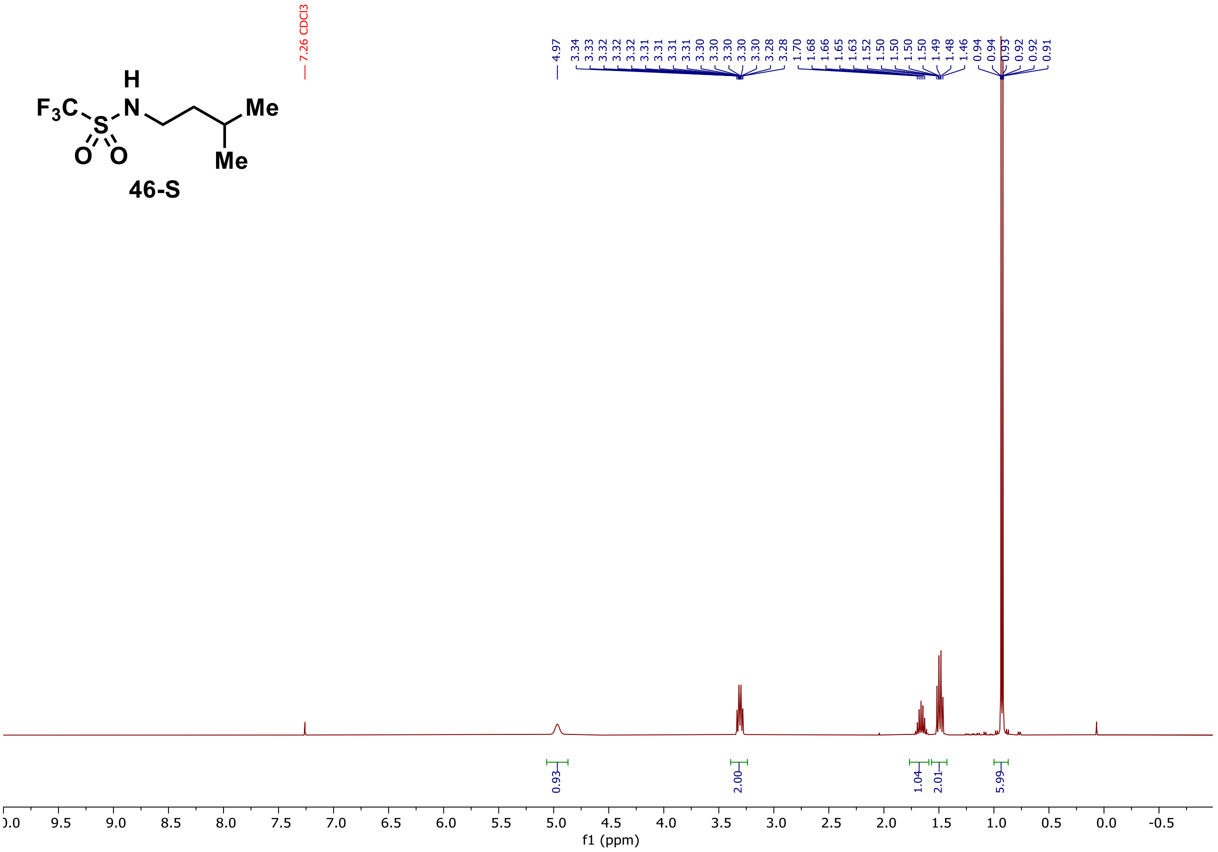

^{13}C NMR (101 MHz, $CDCl_3$) of **46-S**

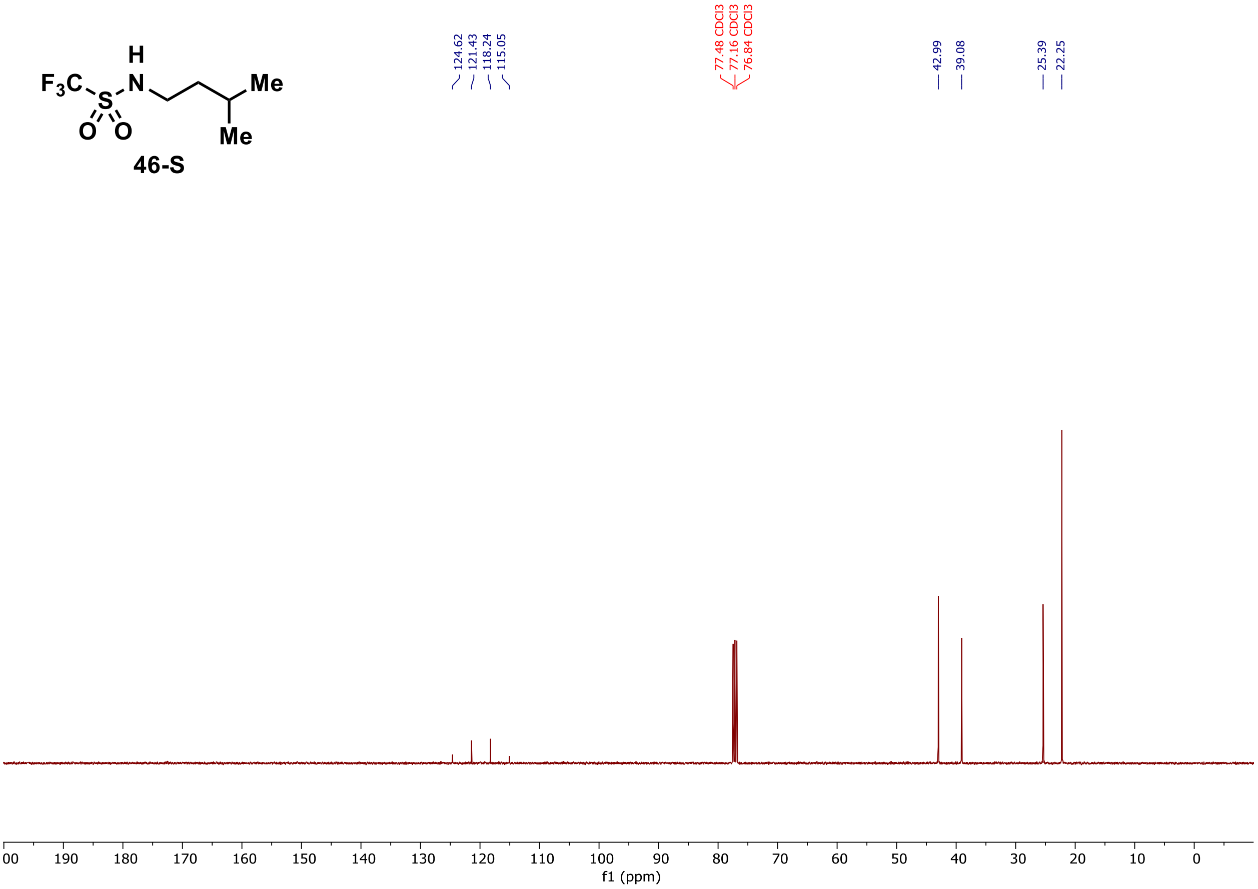

^{19}F NMR (377 MHz, $CDCl_3$) of **46-S**

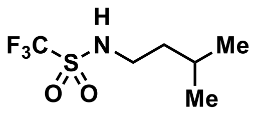

46-S

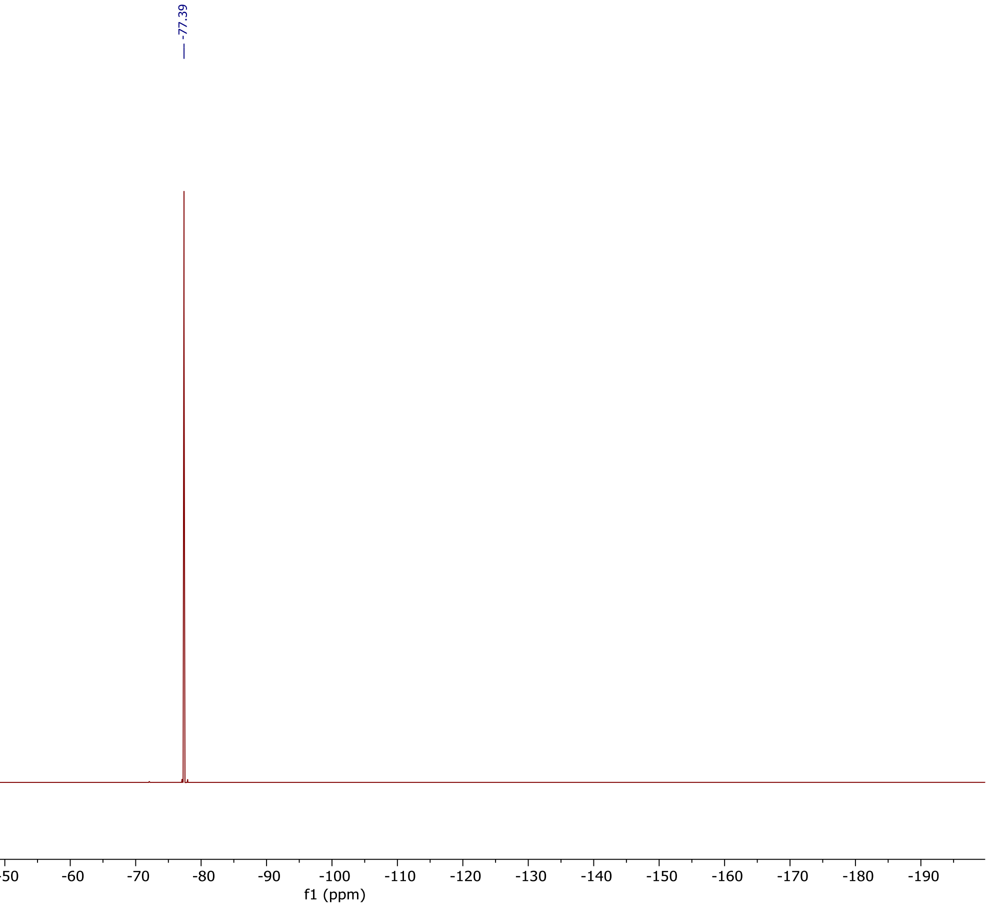

^{1}H NMR (400 MHz, $CDCl_3$) of **46** (***see procedure***)

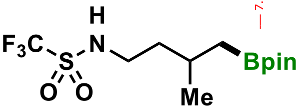

46

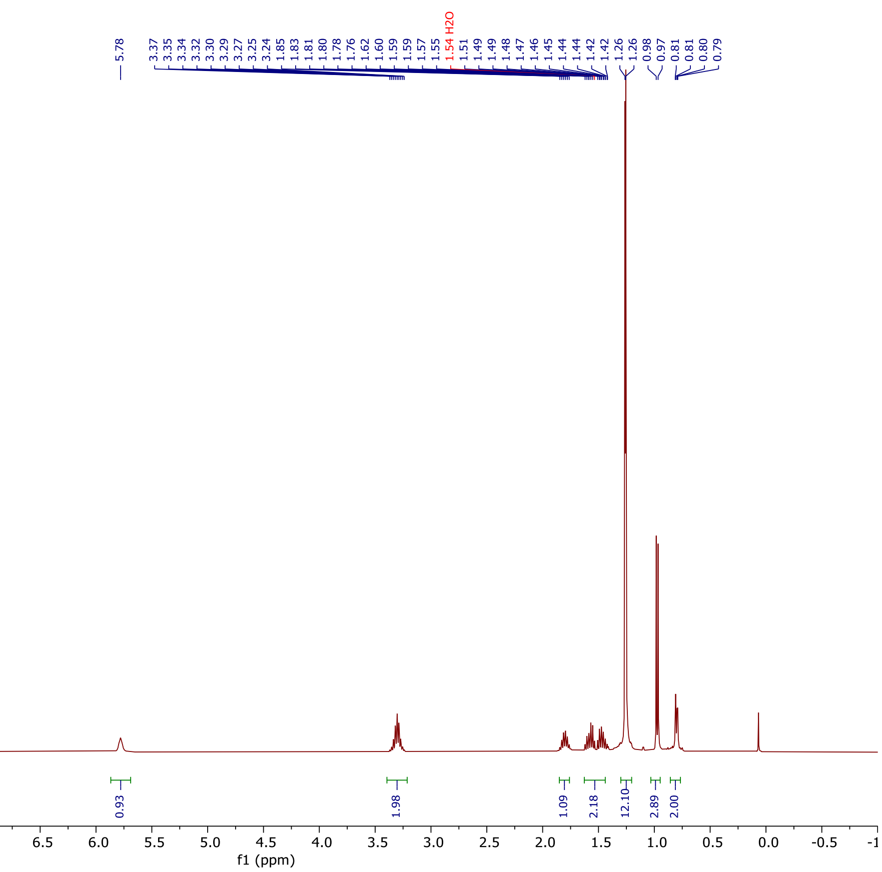

^{13}C NMR (101 MHz, $CDCl_3$) of **46**

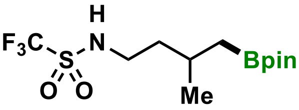

46

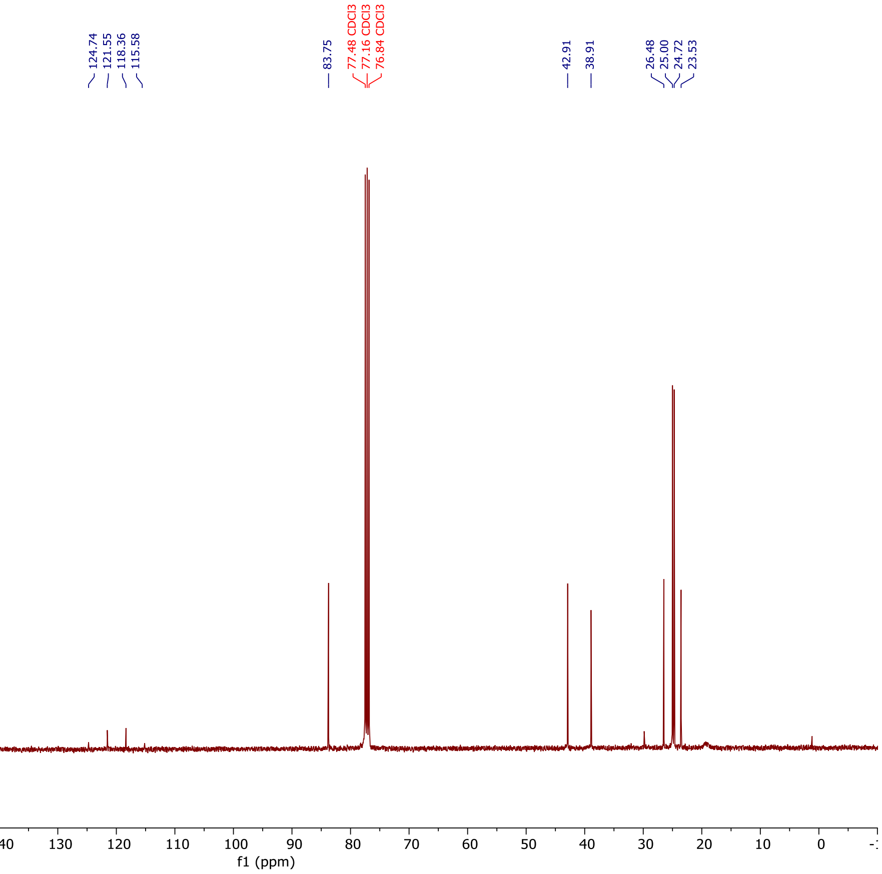

^{19}F NMR (377 MHz, $CDCl_3$) of **46**

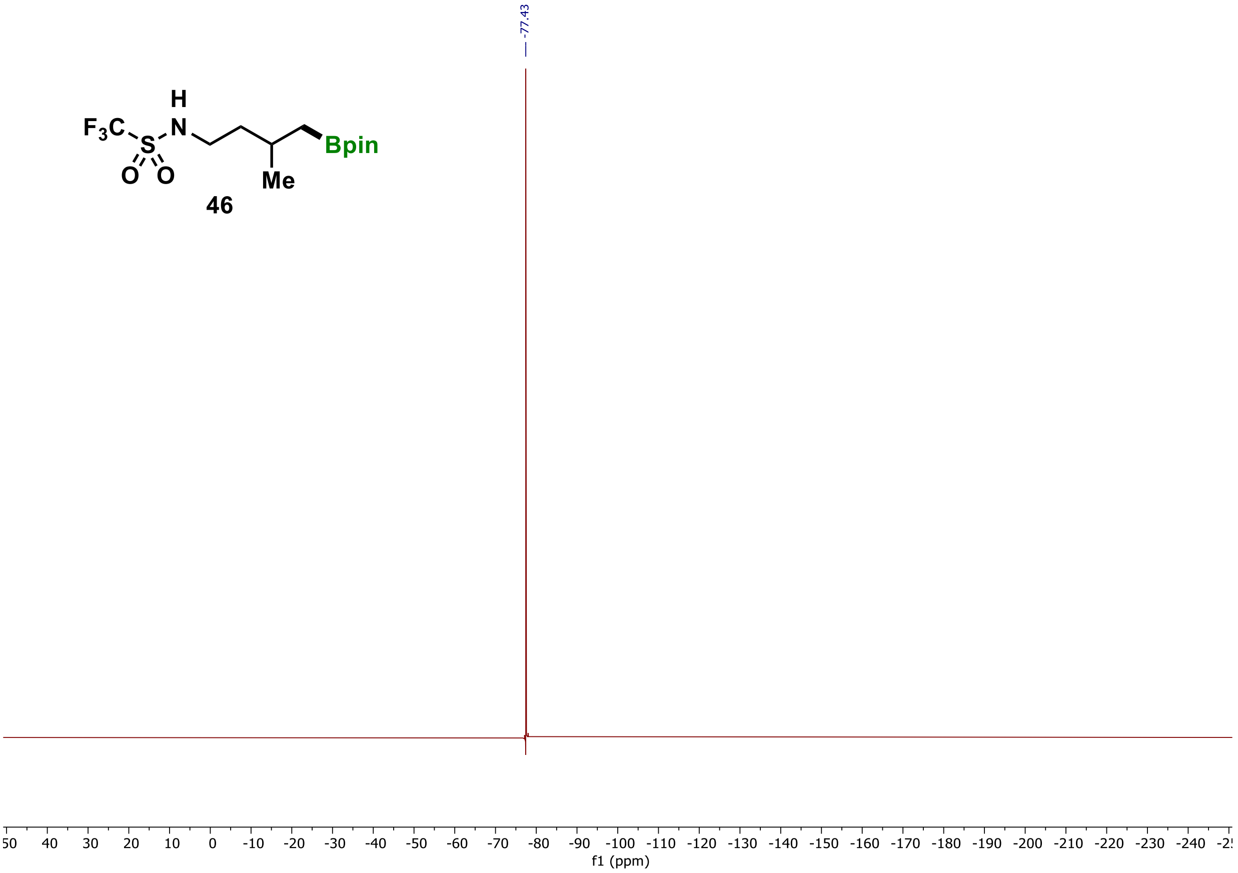

^{11}B NMR (128 MHz, $CDCl_3$) of **46**

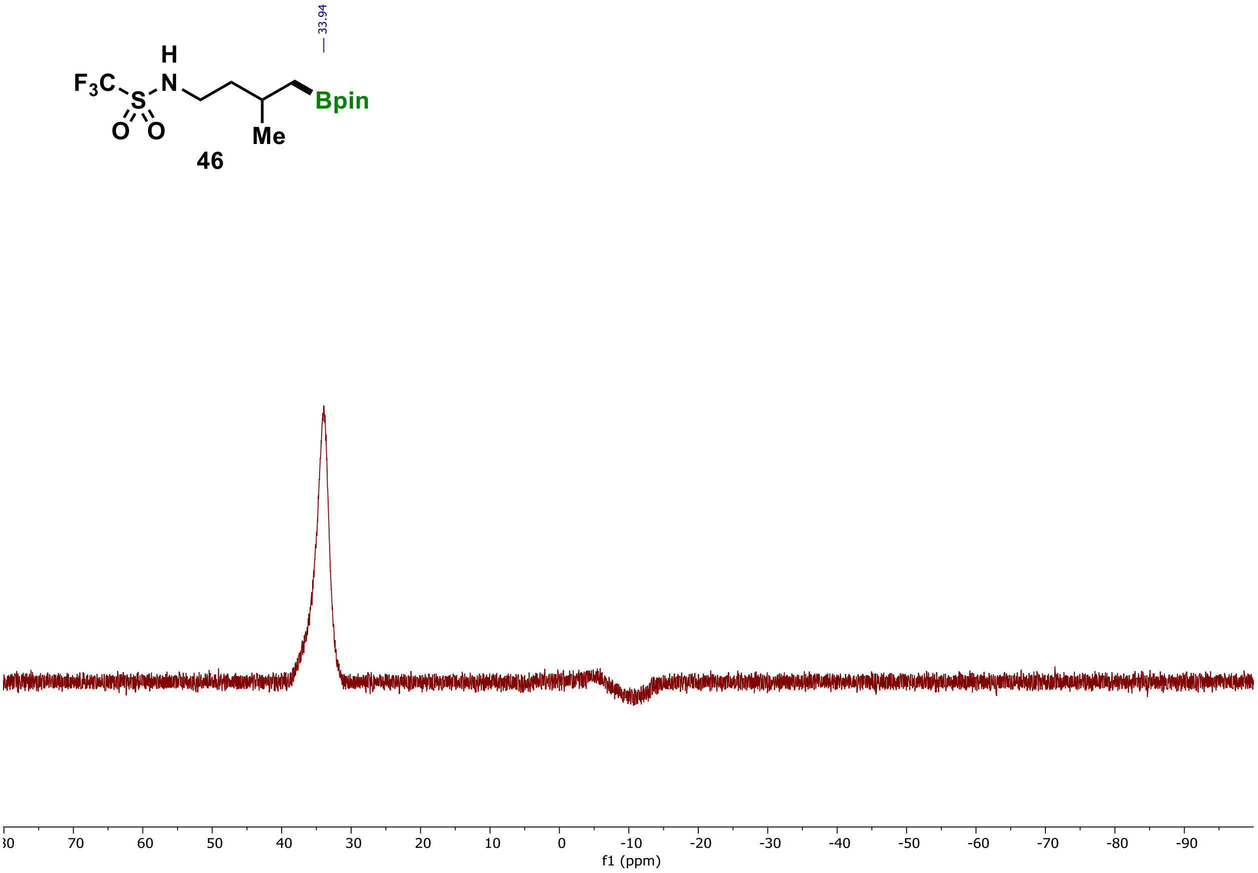

^{1}H NMR (400 MHz, $CDCl_3$) of **47** (*see procedure*)

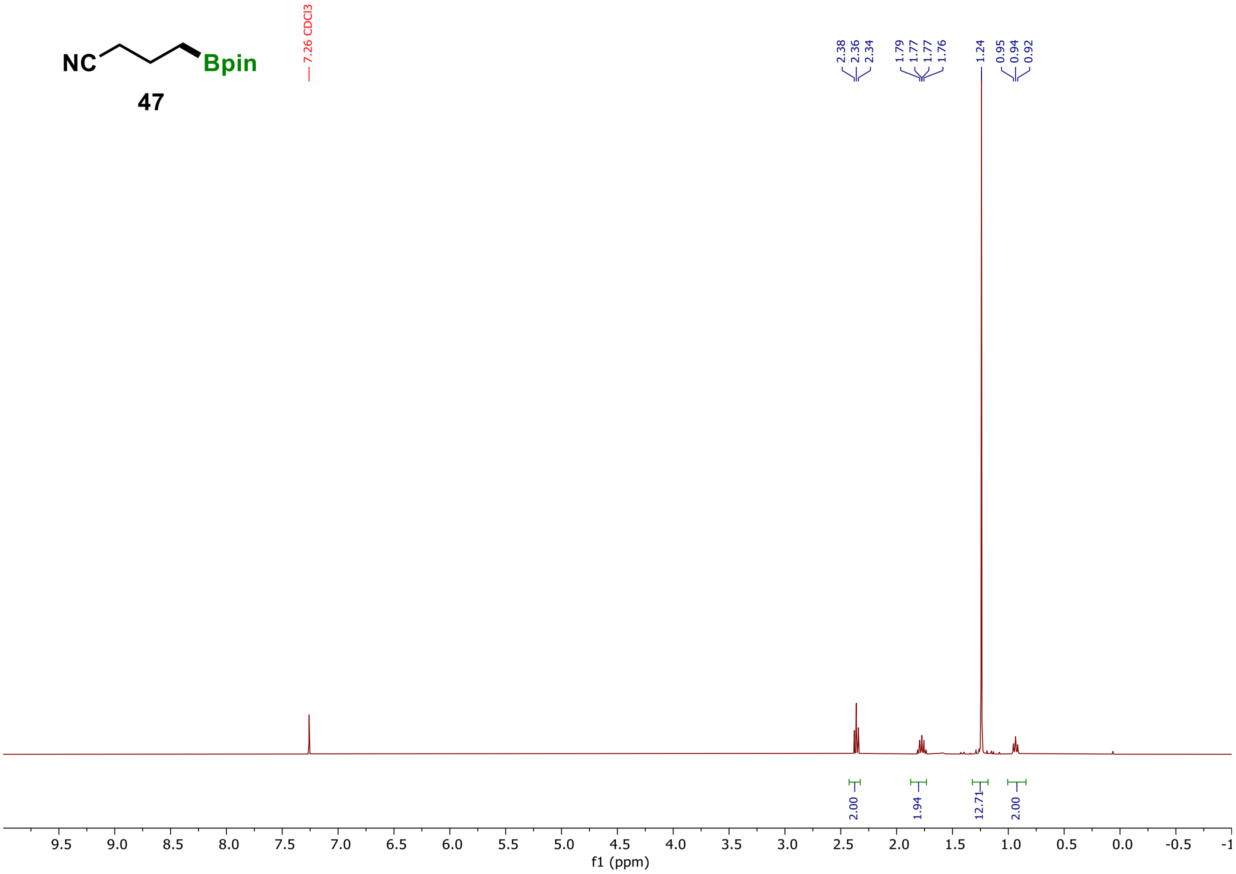

^{13}C NMR (101 MHz, $CDCl_3$) of **47**

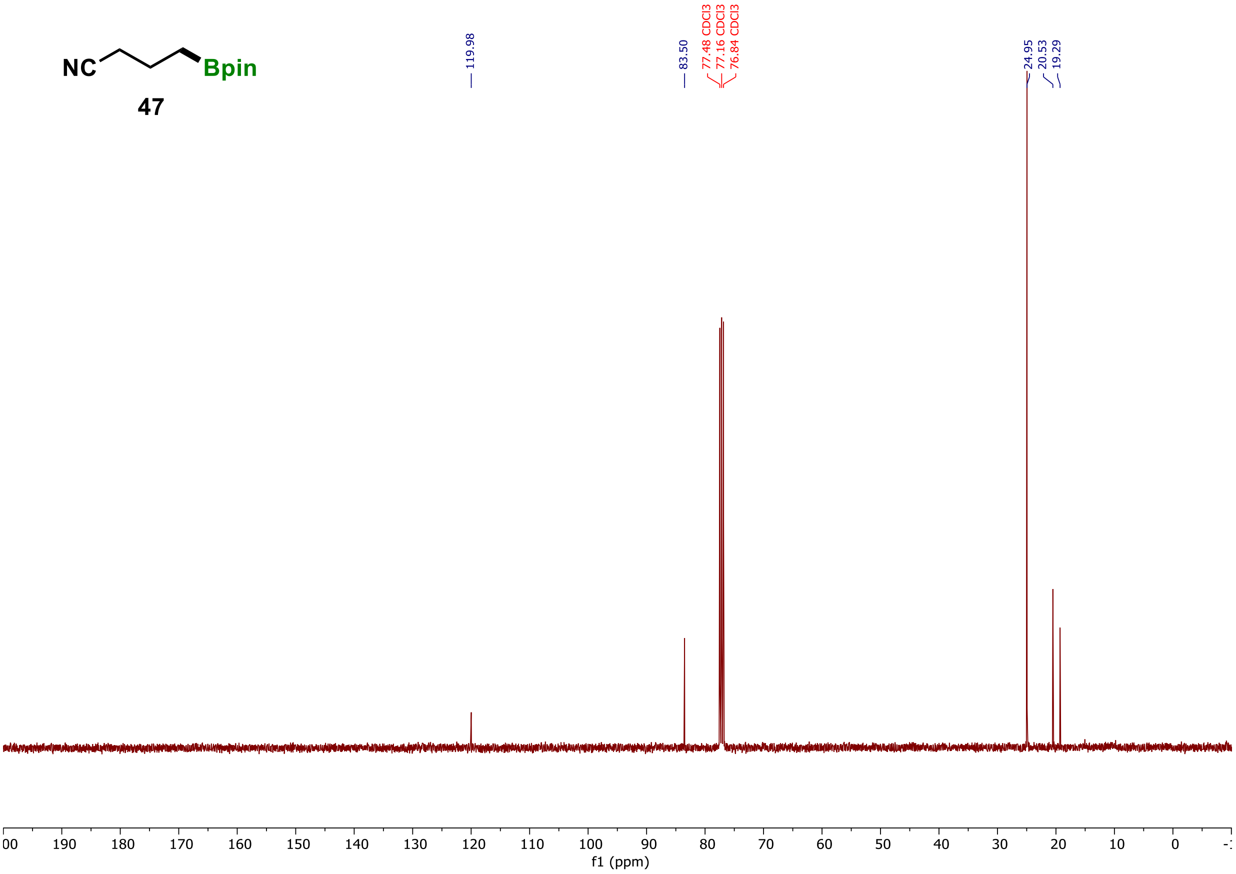

^{1}H NMR (400 MHz, $CDCl_3$) of **48** (*see procedure*)

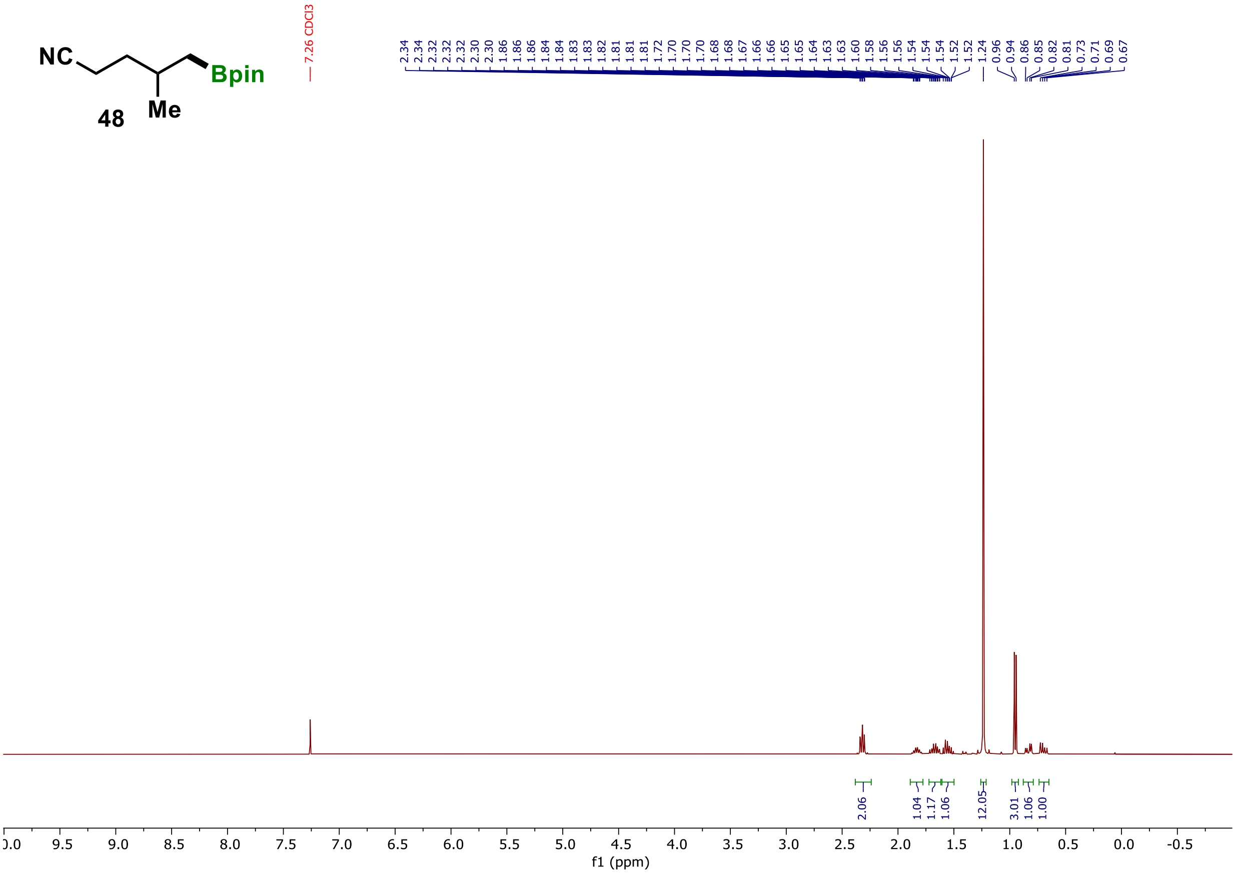

^{13}C NMR (101 MHz, $CDCl_3$) of **48**

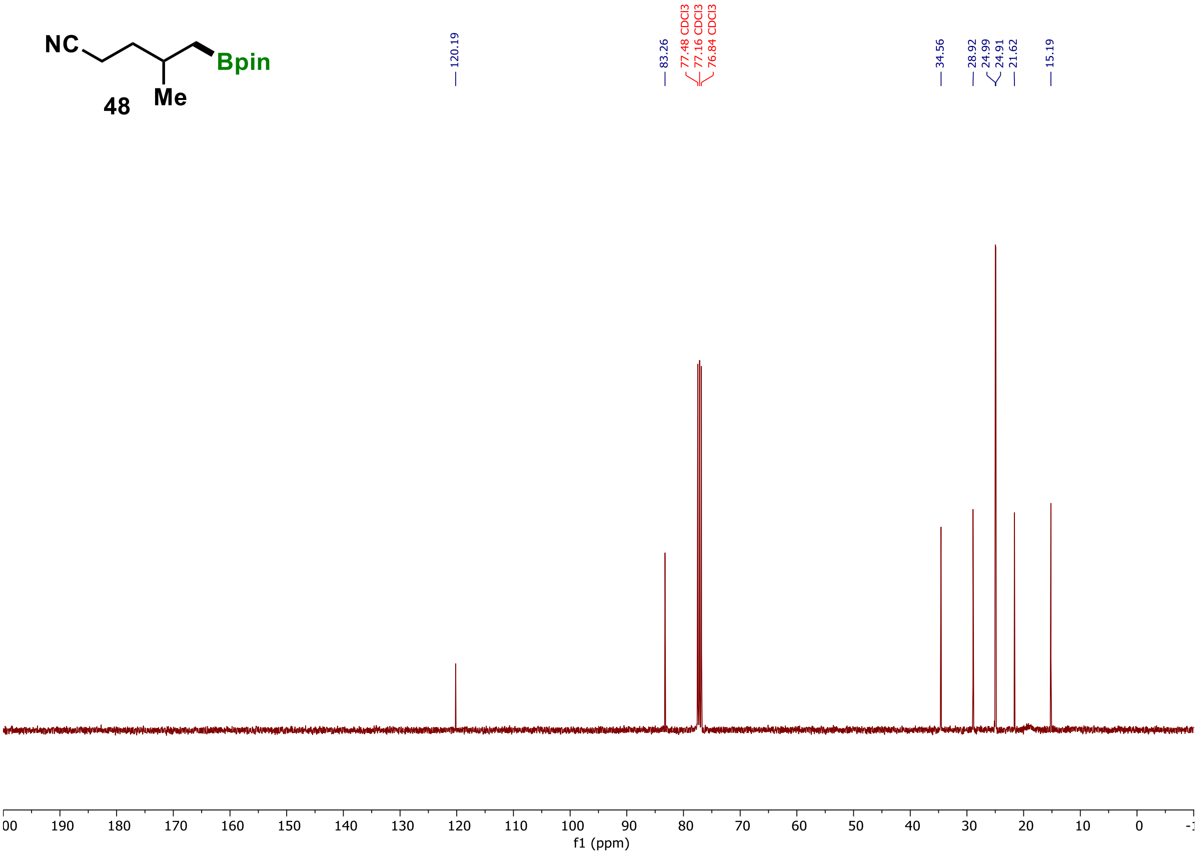

^{11}B NMR (128 MHz, $CDCl_3$) of **48**

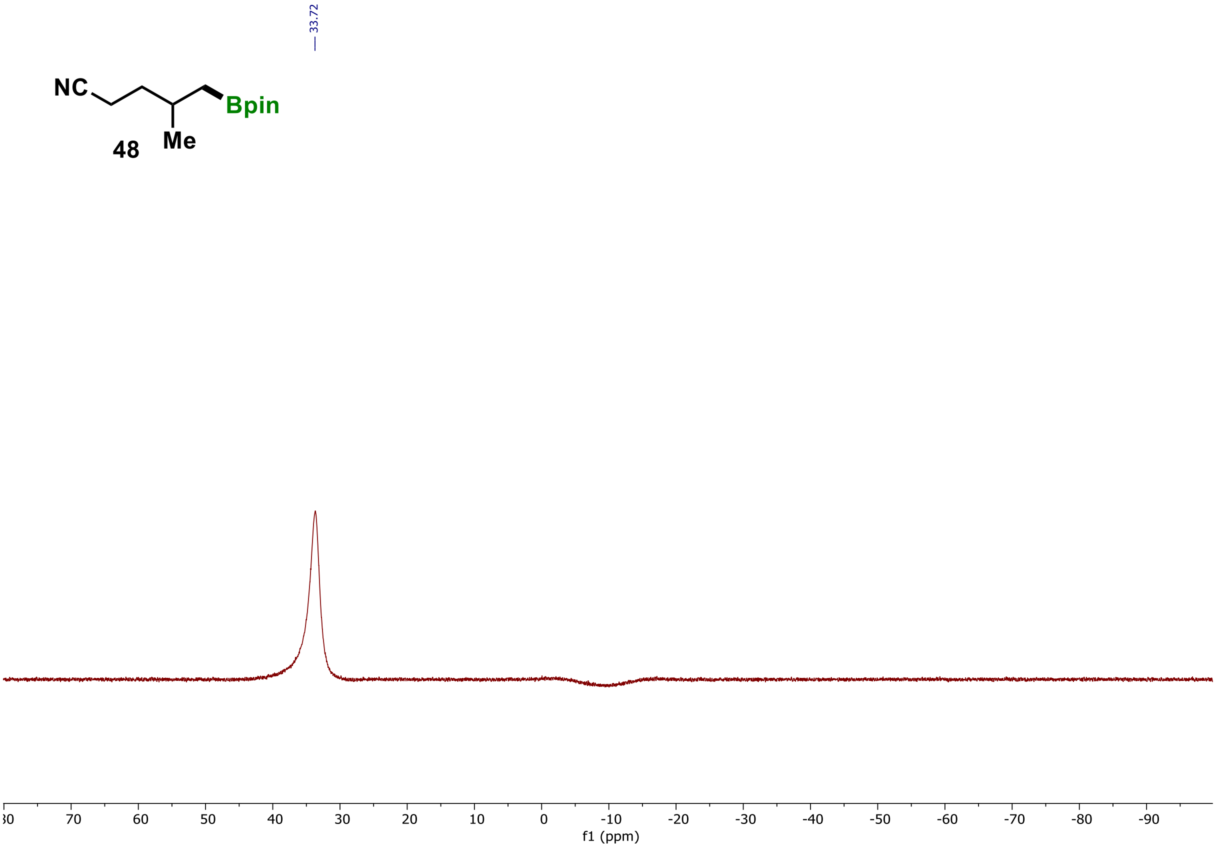

^{1}H NMR (400 MHz, $CDCl_3$) of **49** (***see procedure***)

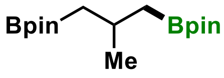

49

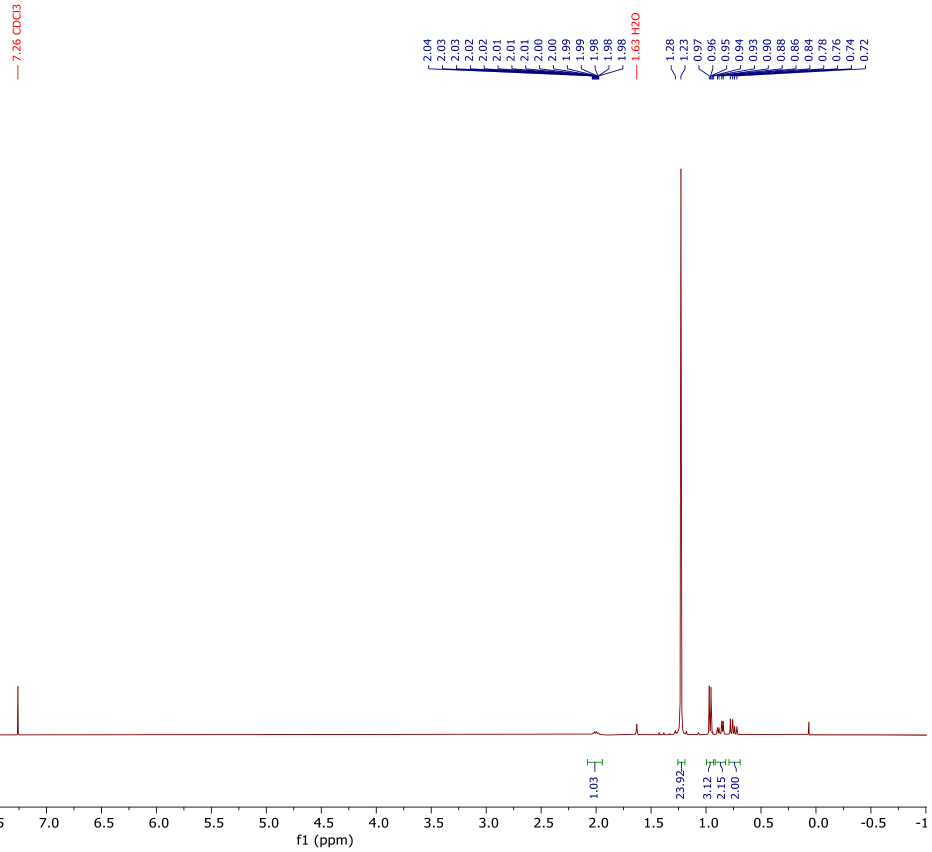

^{13}C NMR (101 MHz, $CDCl_3$) of **49**

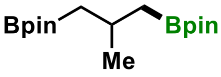

49

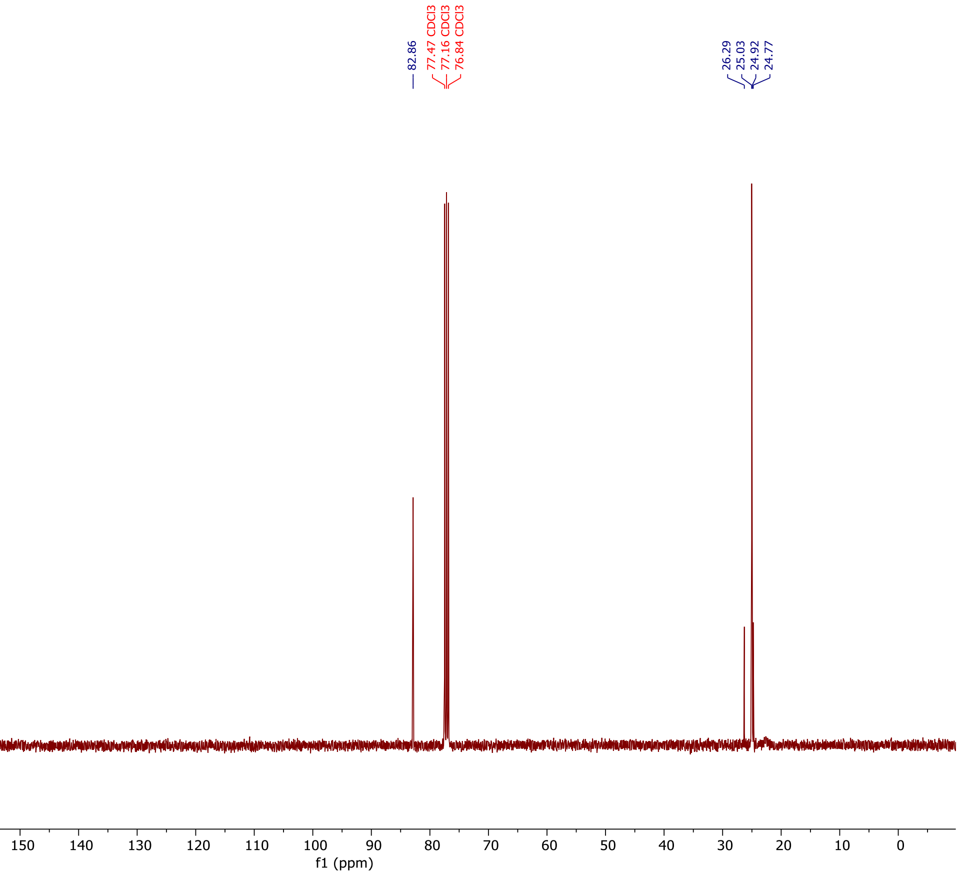

^{11}B NMR (128 MHz, $CDCl_3$) of **49**

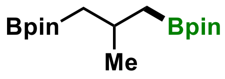

49

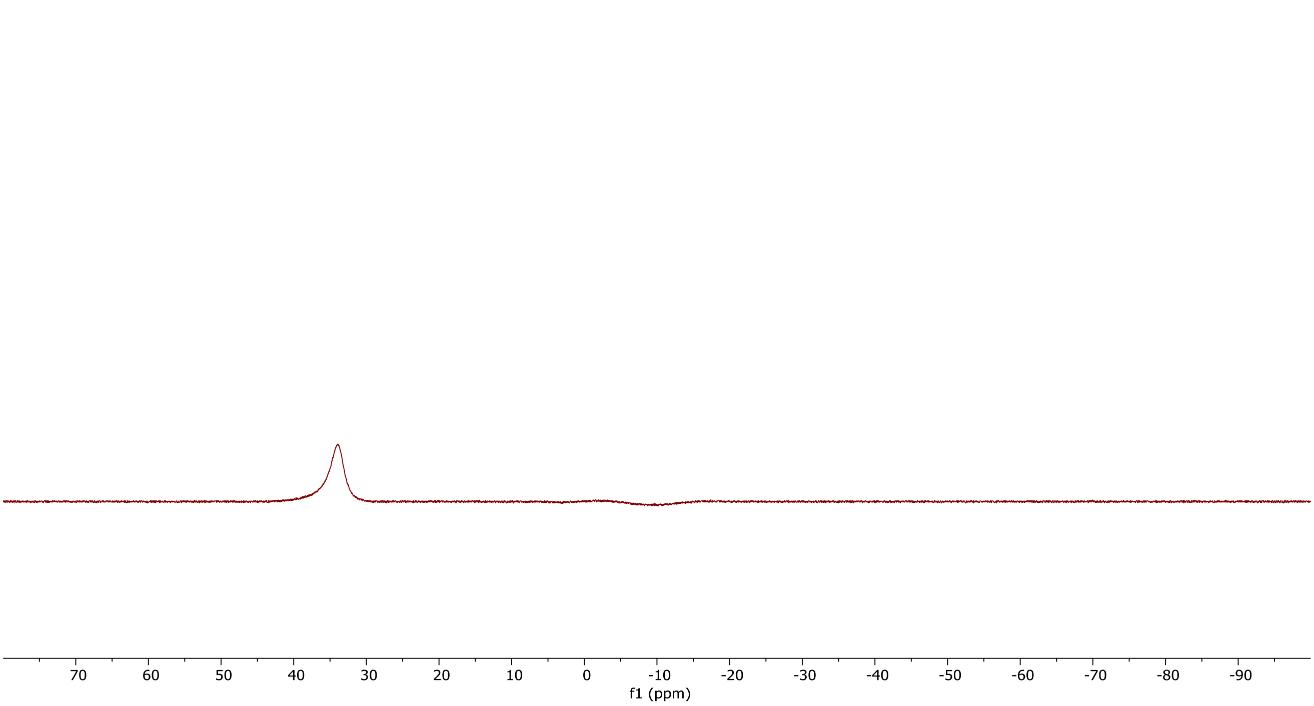

^1H NMR (400 MHz, $CDCl_3$) of **50** (*see procedure*)

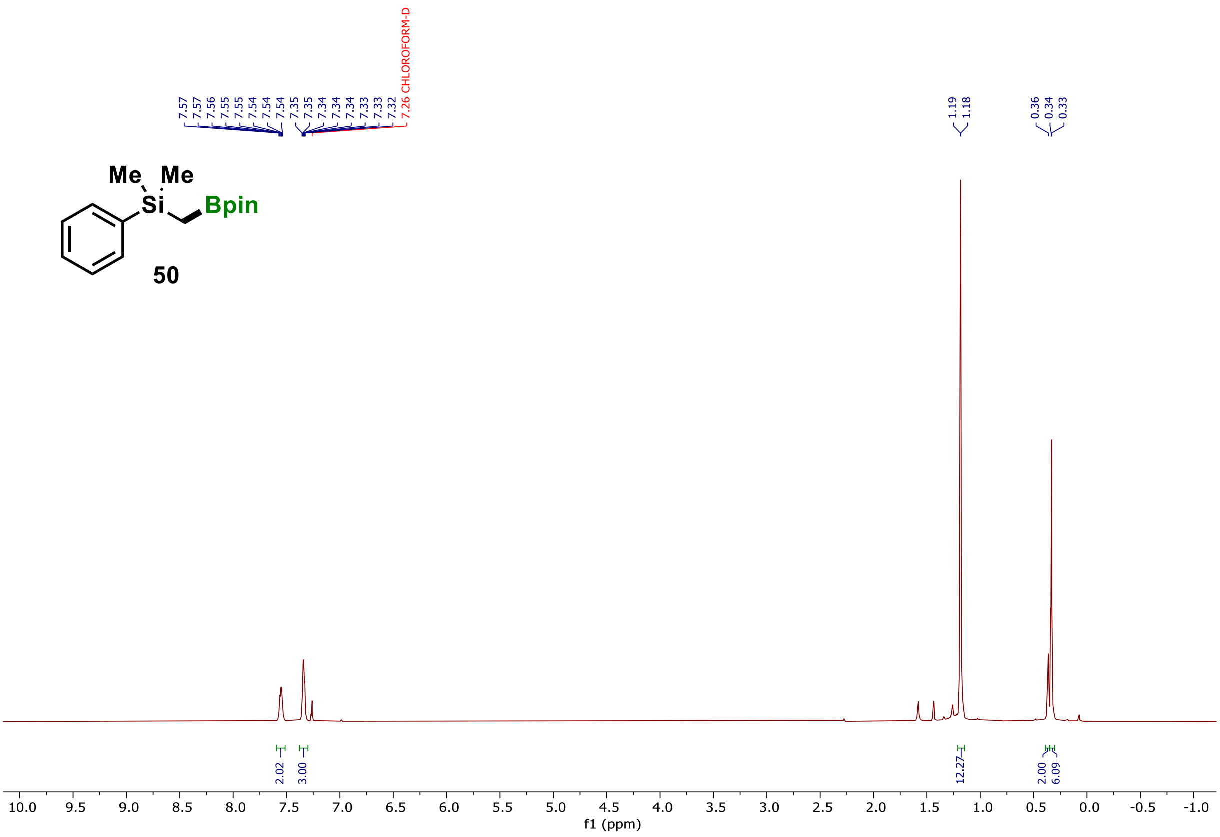

^{13}C NMR (101 MHz, $CDCl_3$) of **50**

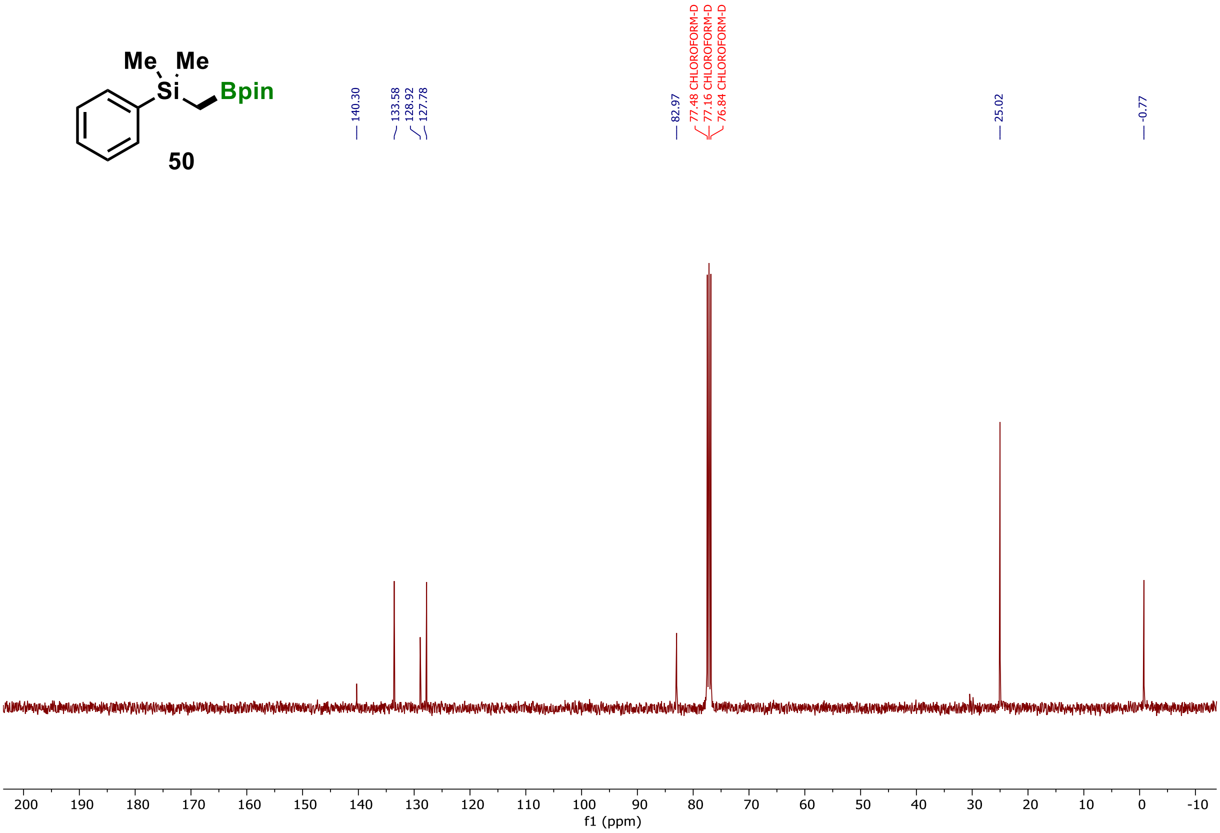

^{1}H NMR (400 MHz, $CDCl_3$) of **51** (*see procedure*)

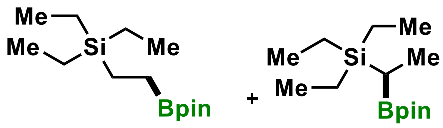

51a + **51b**

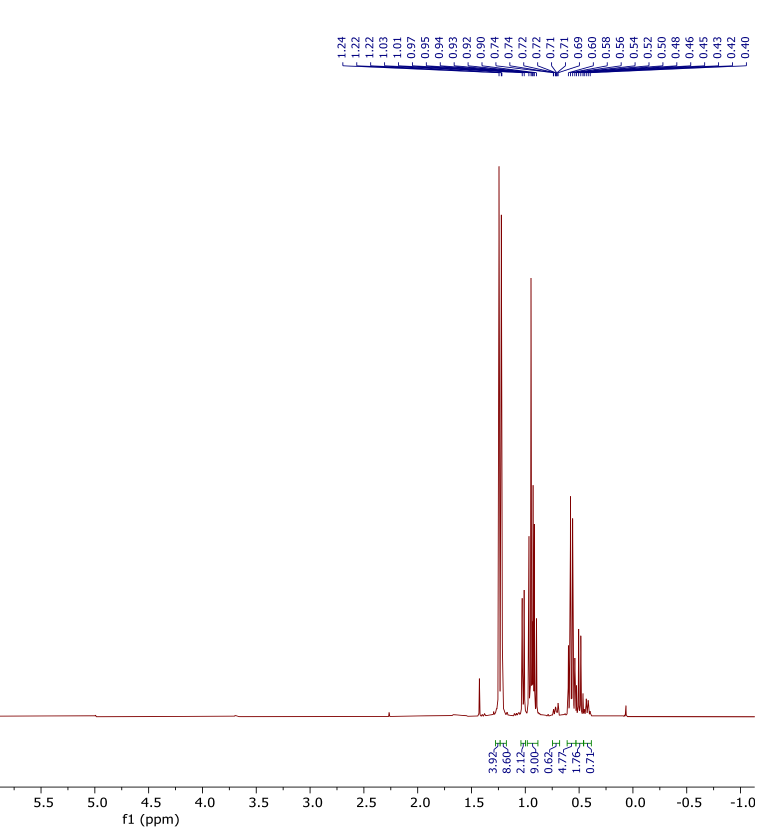

^{13}C NMR (101 MHz, $CDCl_3$) of **51**

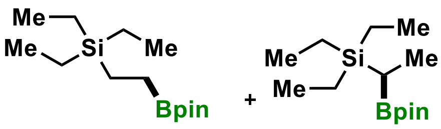

51a + **51b**

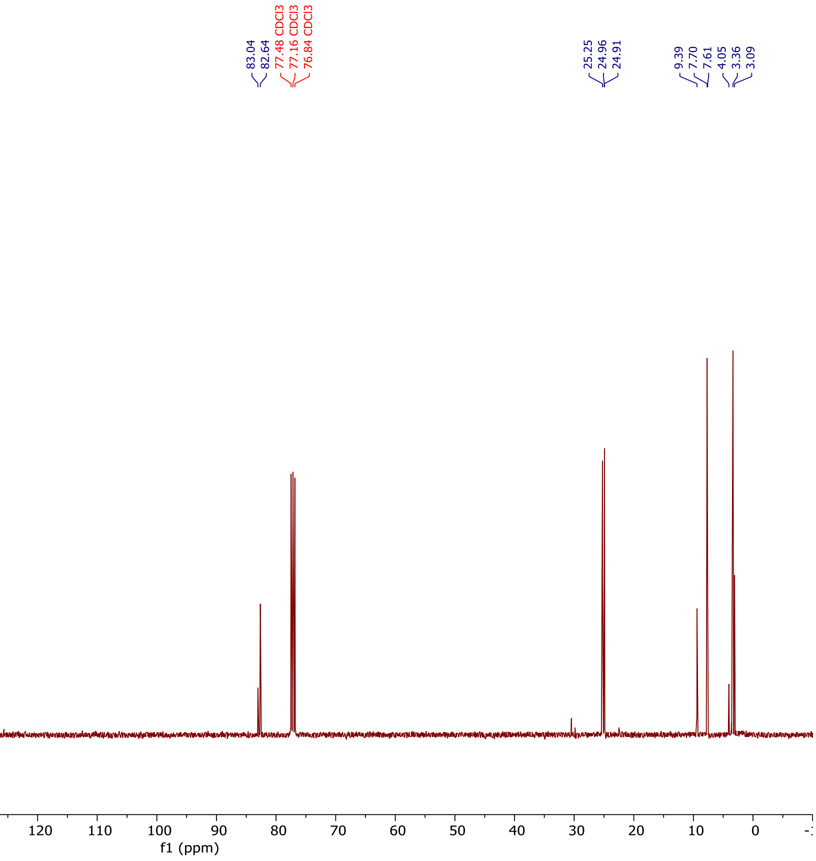

^{1}H NMR (400 MHz, $CDCl_3$) of **52-S** (*see procedure*)

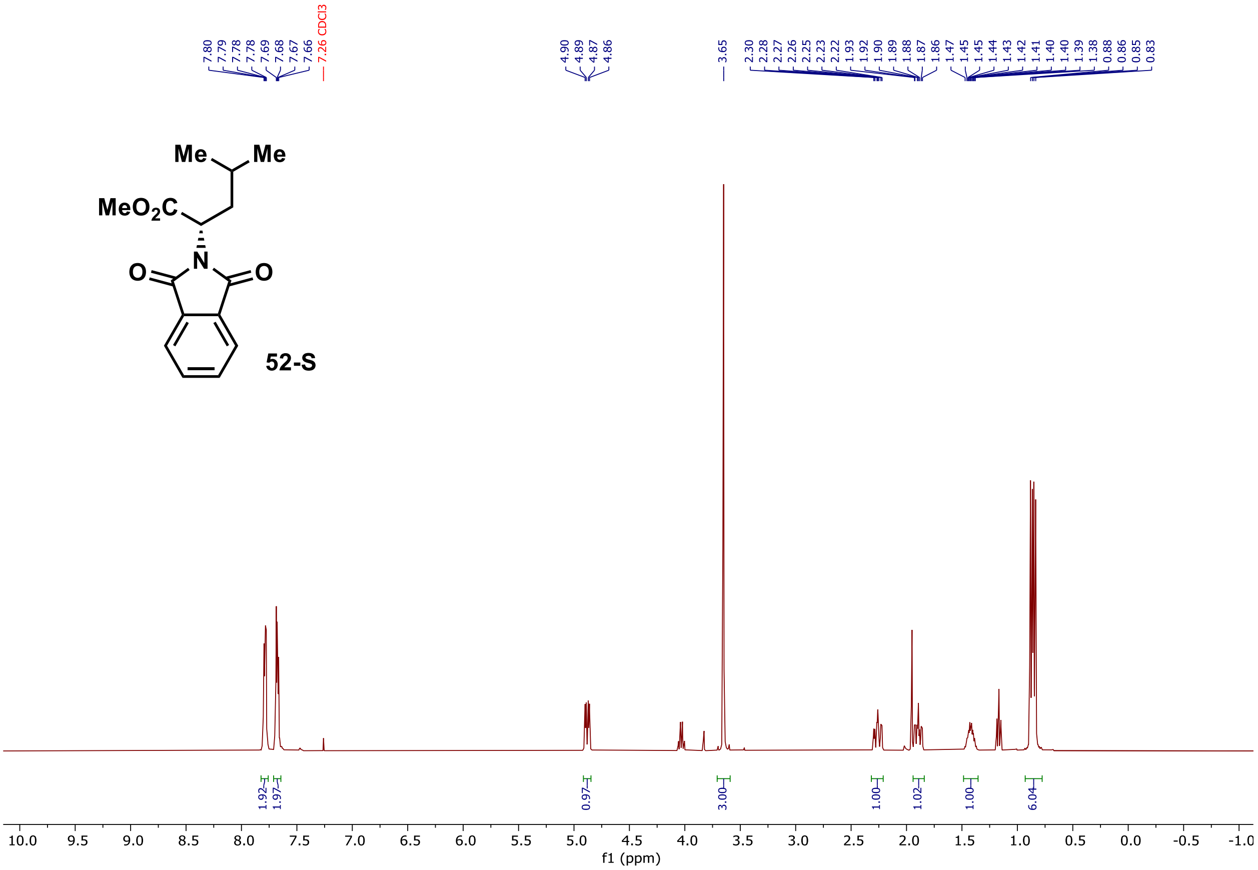

^{13}C NMR (101 MHz, $CDCl_3$) of **52-S**

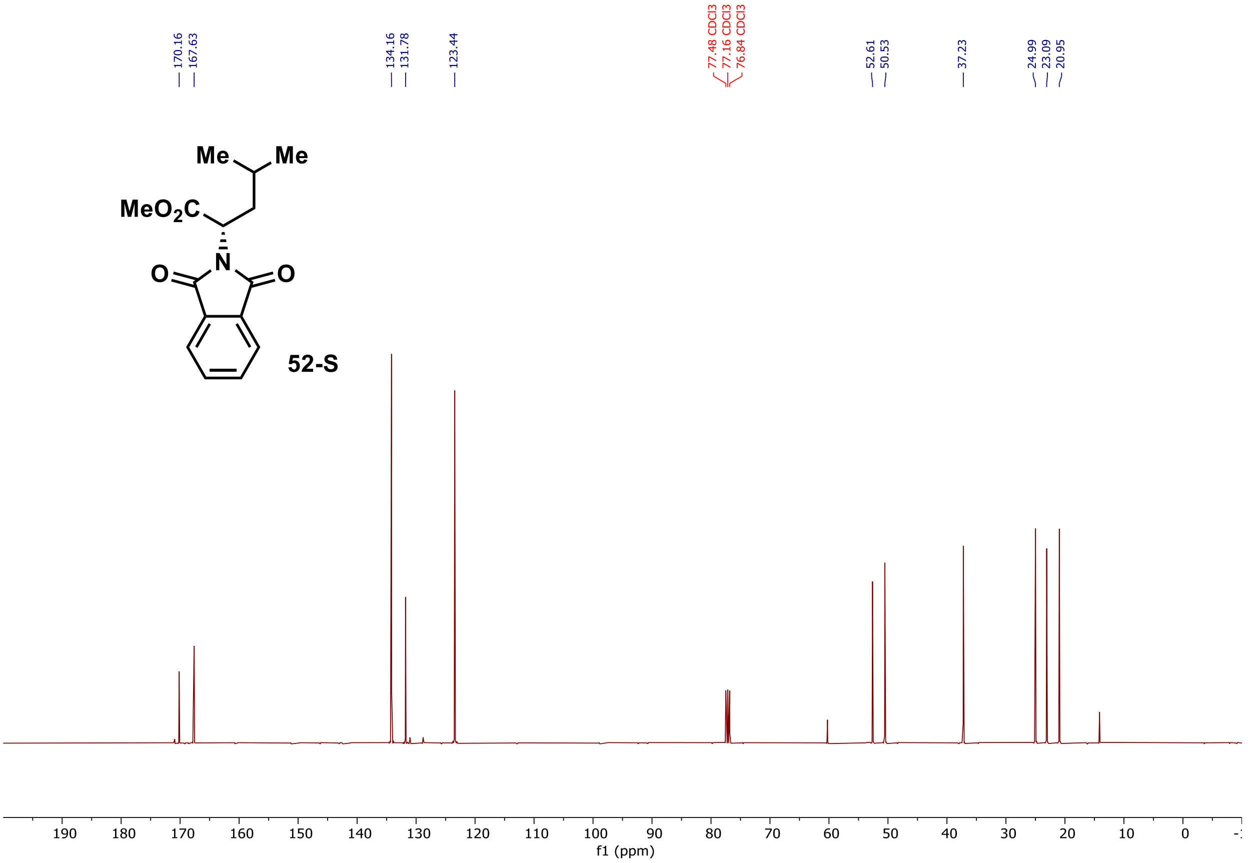

^{1}H NMR (400 MHz, $CDCl_3$) of **52** (*see procedure*)

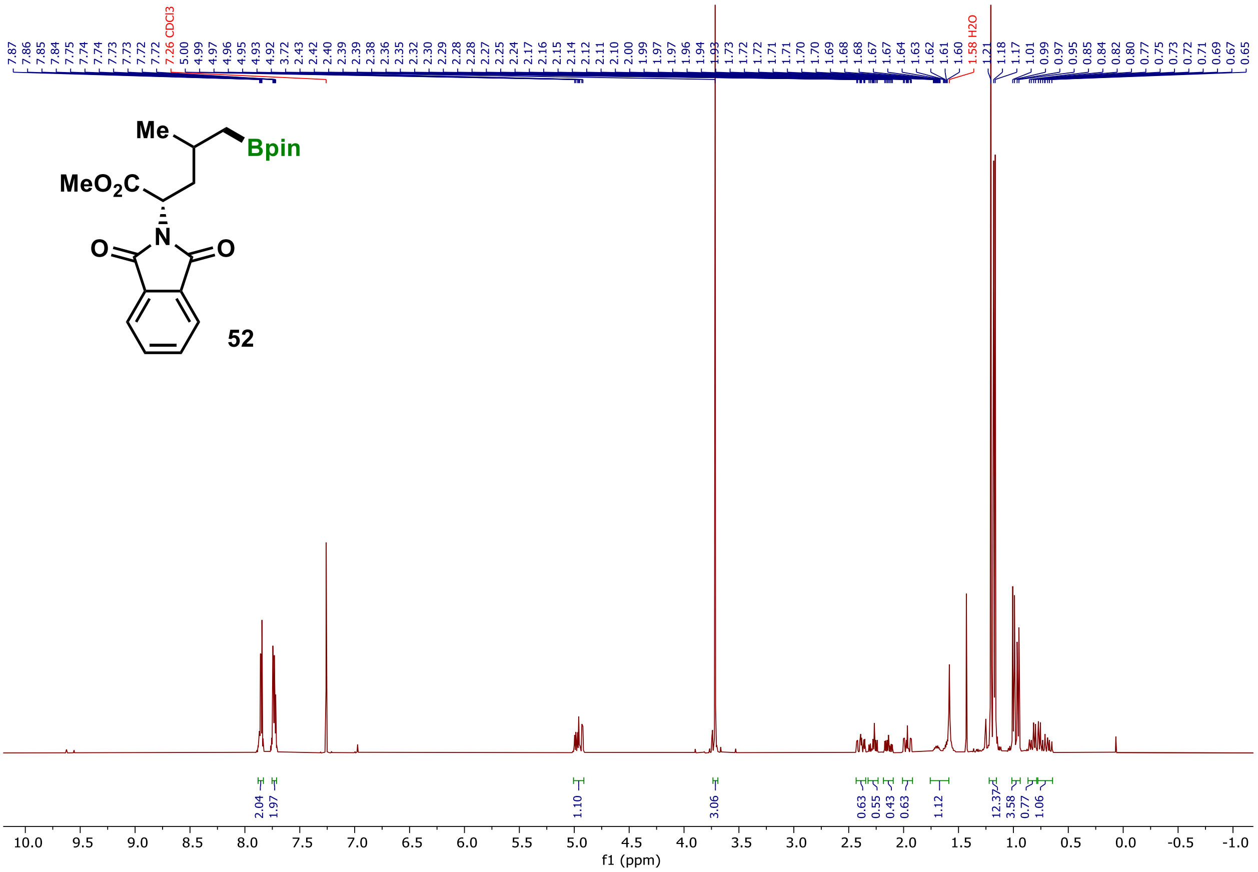

^{13}C NMR (101 MHz, $CDCl_3$) of **52**

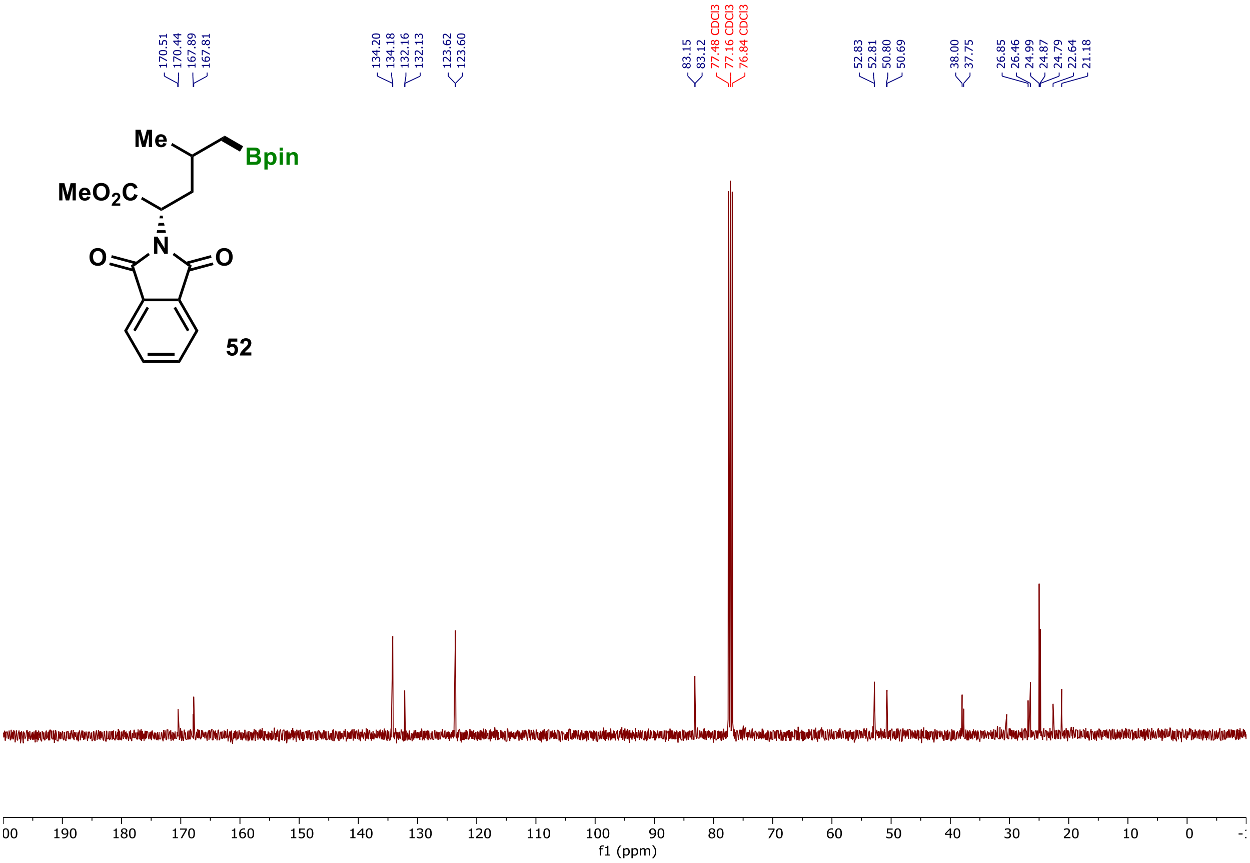

^{1}H NMR (400 MHz, $CDCl_3$) of **53-S** (*see procedure*)

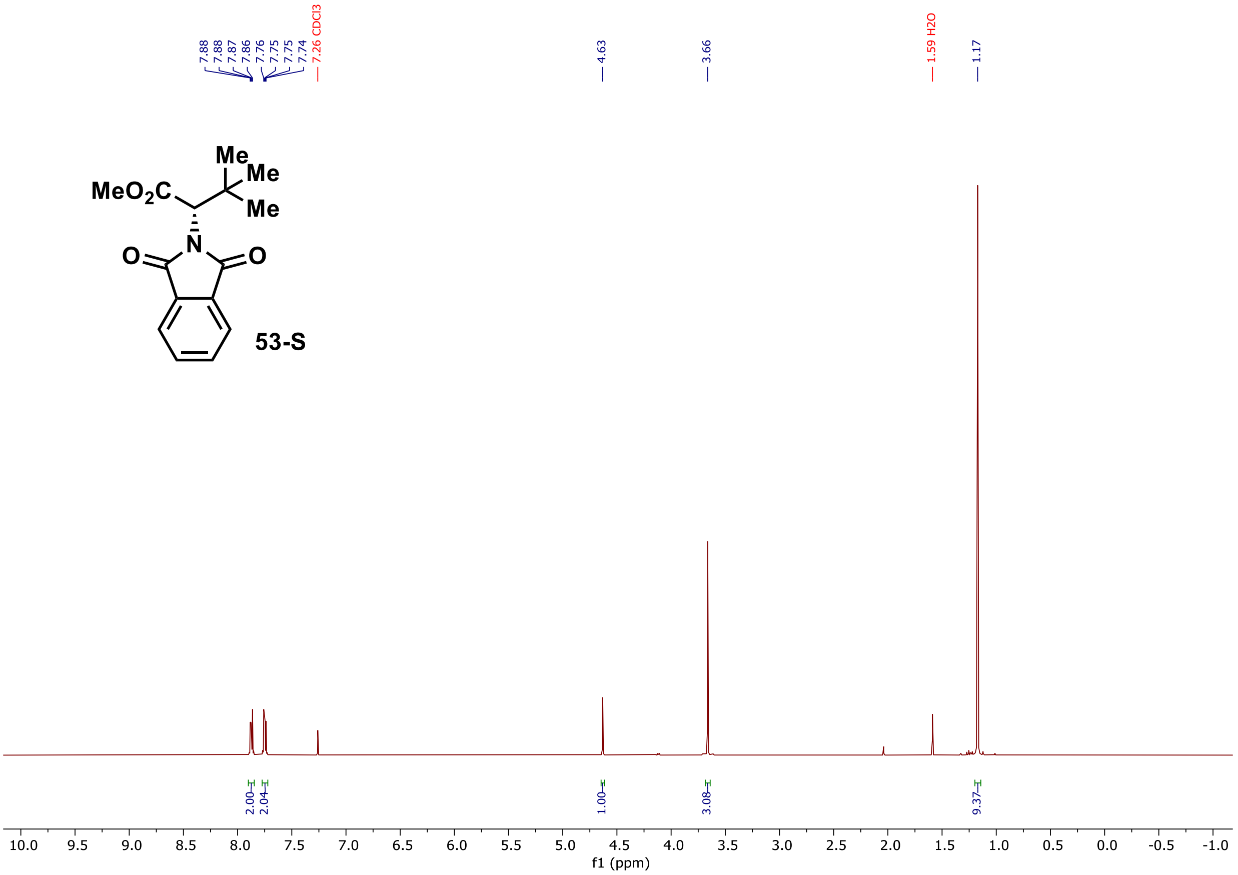

^{13}C NMR (101 MHz, $CDCl_3$) of **53-S**

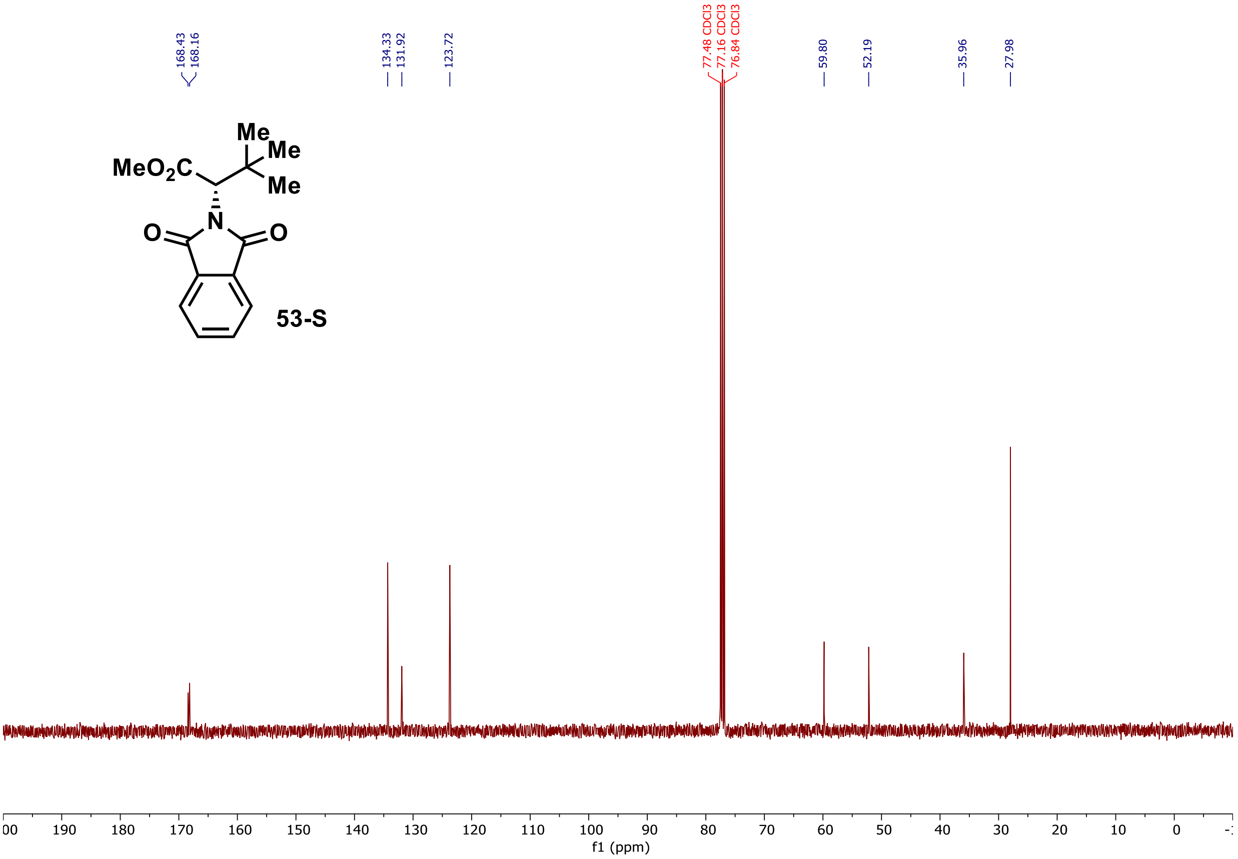

^{1}H NMR (400 MHz, $CDCl_3$) of **53** (*see procedure*)

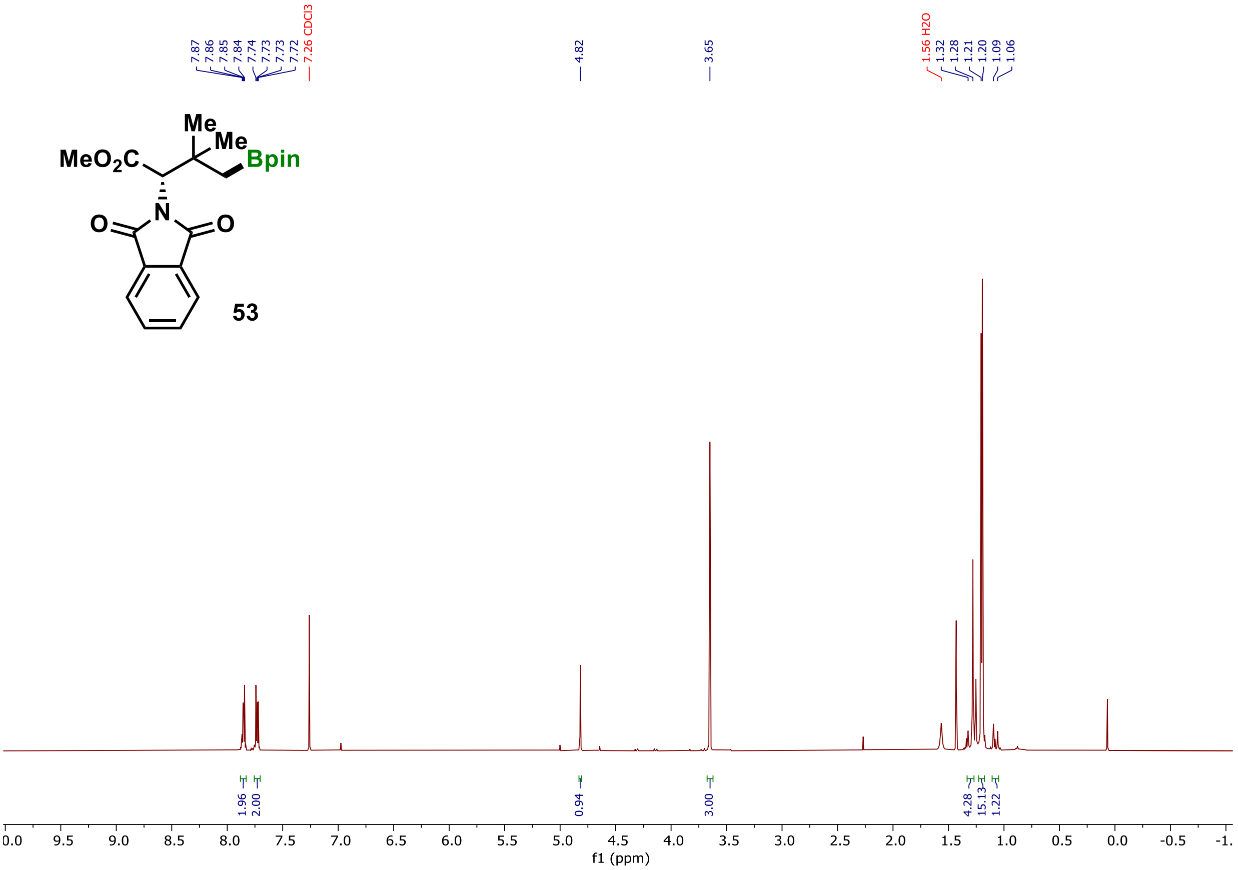

^{13}C NMR (101 MHz, $CDCl_3$) of **53**

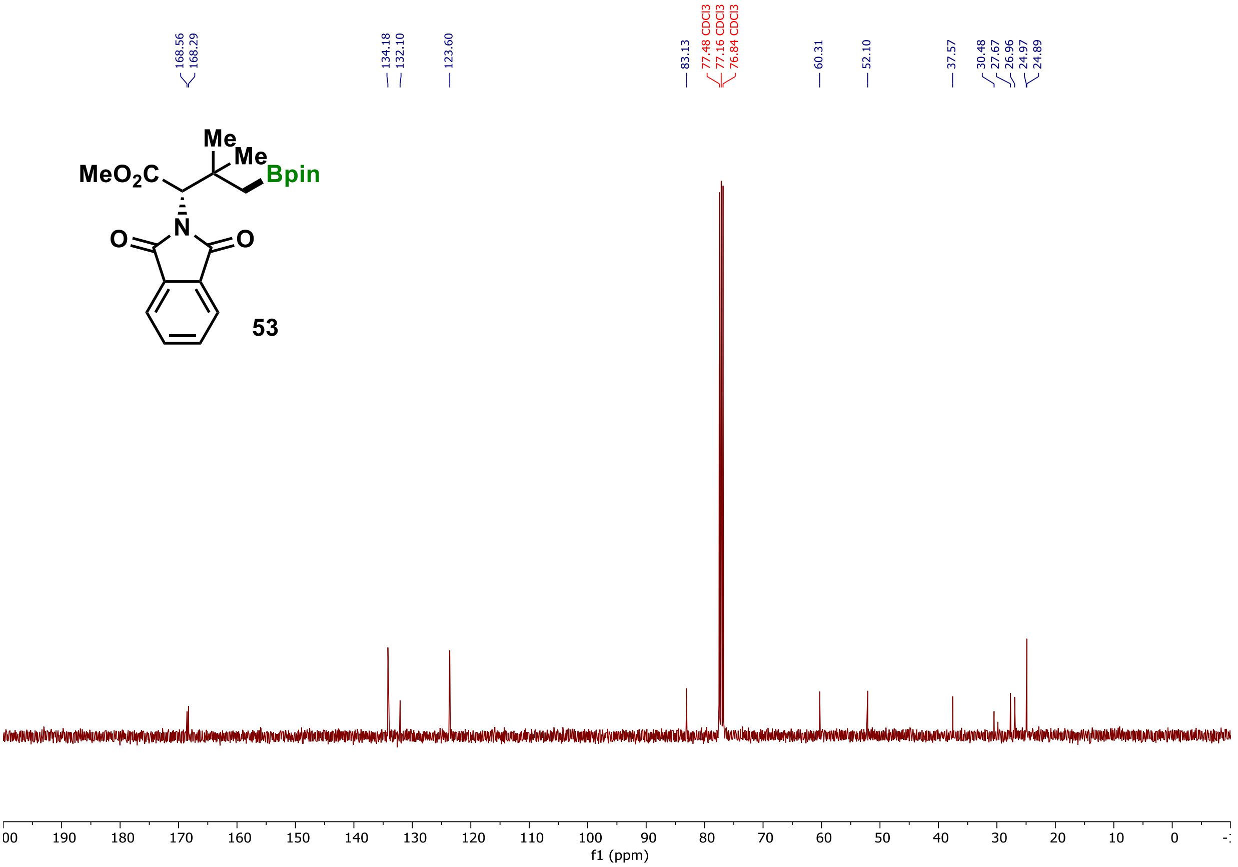

^{1}H NMR (400 MHz, $CDCl_3$) of **54-ox** (***see procedure***)

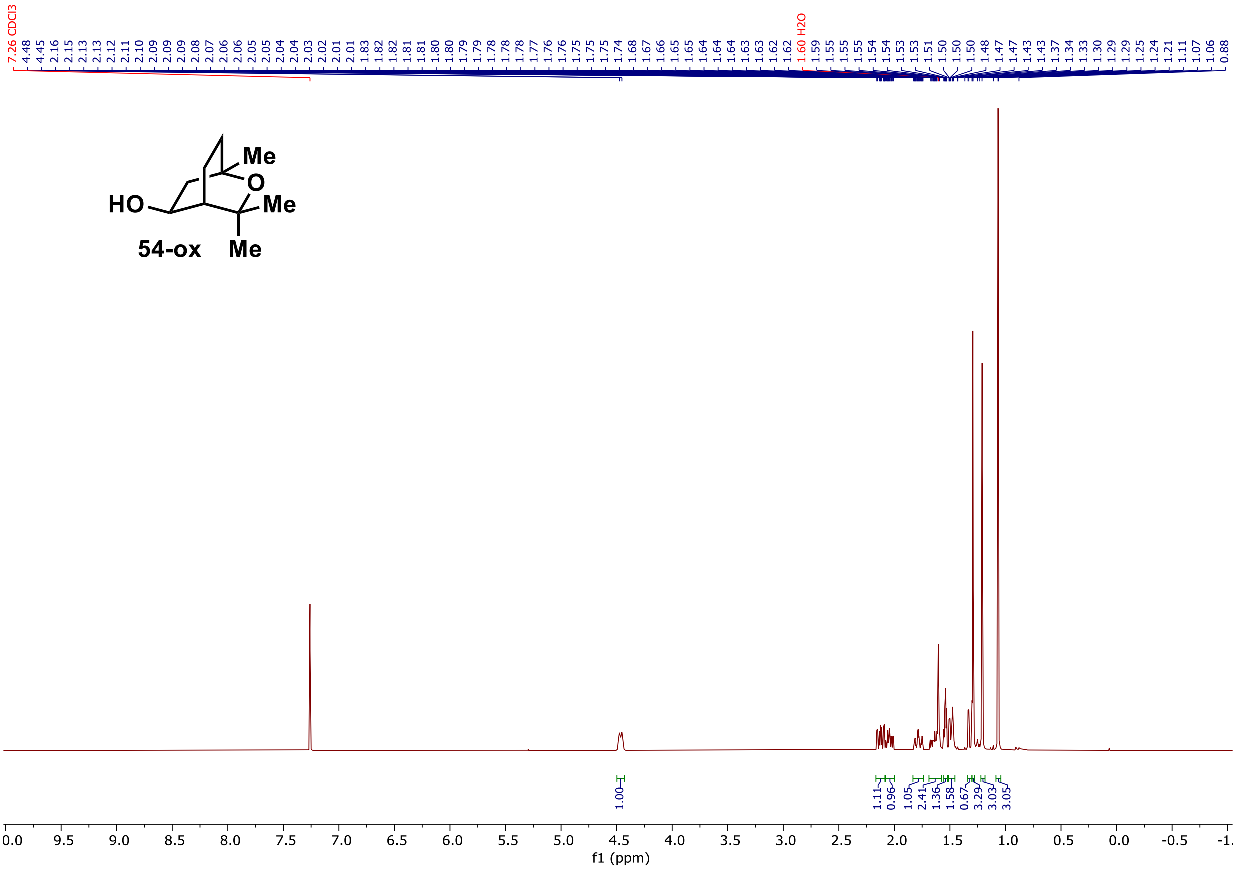

^{13}C NMR (101 MHz, $CDCl_3$) of **54-ox**

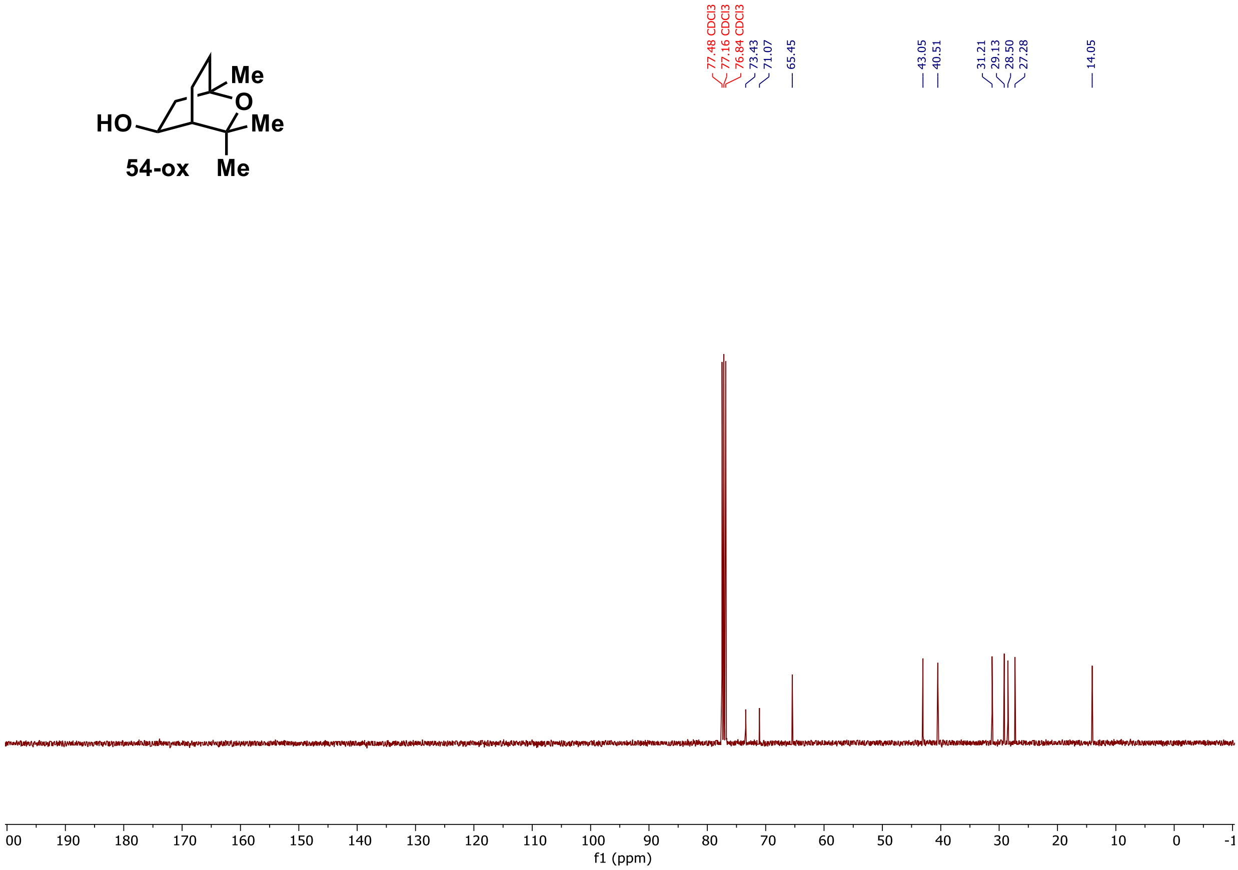

^{1}H NMR (400 MHz, $CDCl_3$) of **55-S** (*see procedure*)

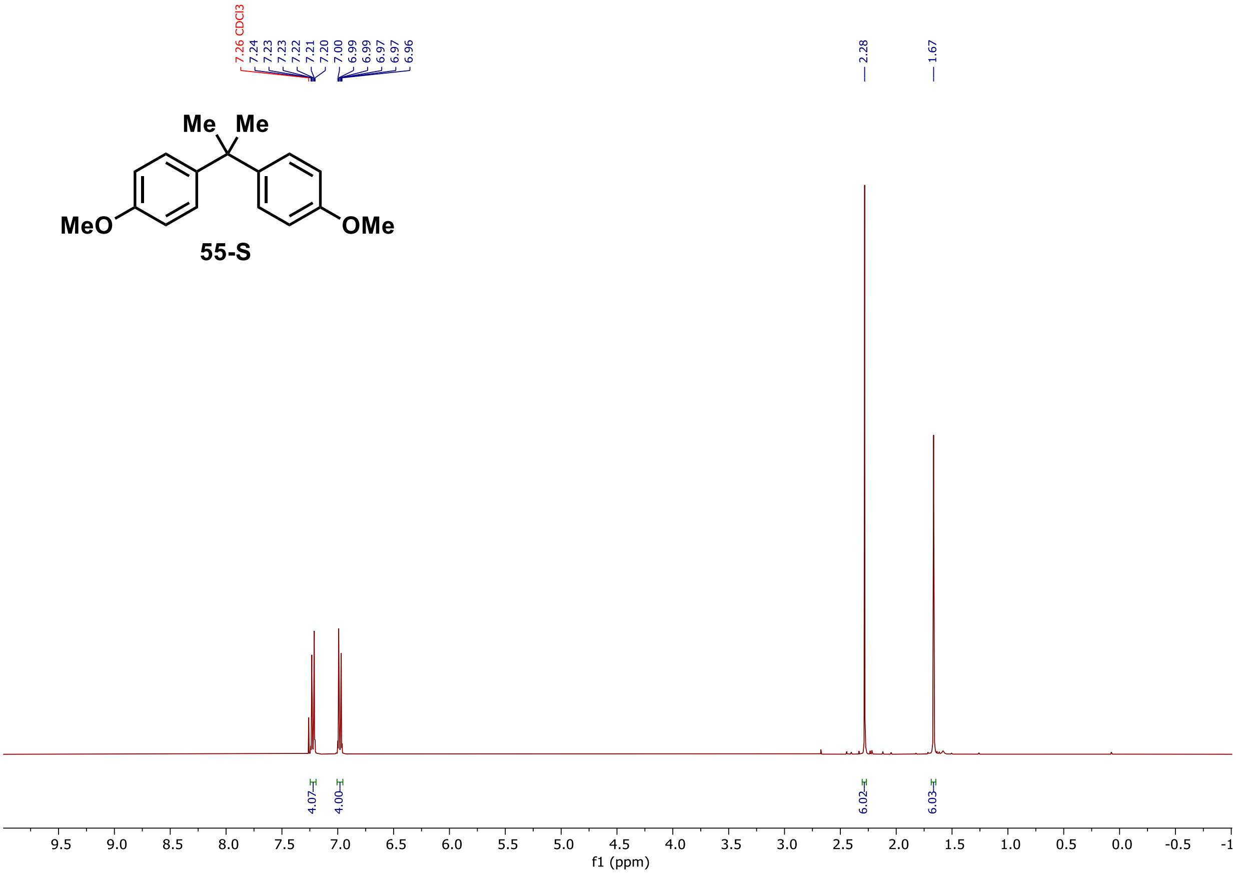

^{13}C NMR (101 MHz, $CDCl_3$) of **55-S**

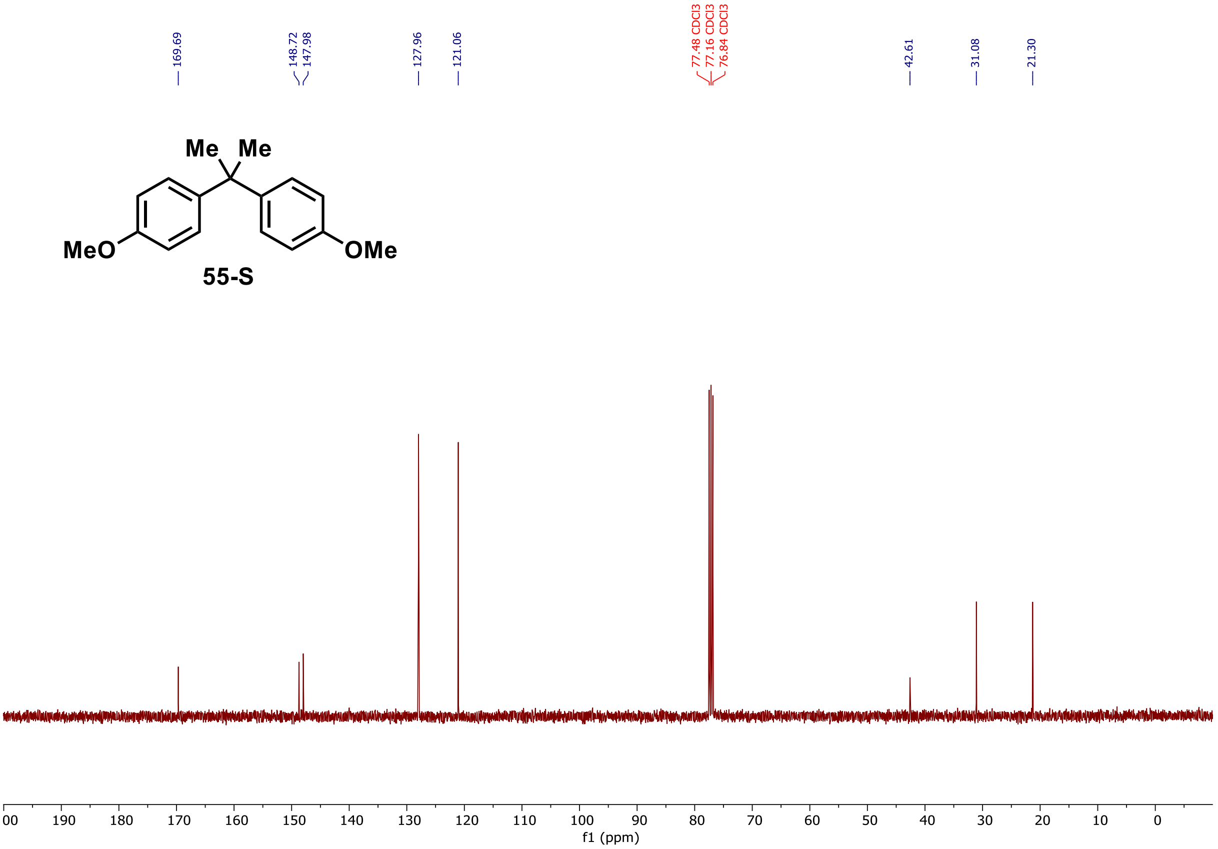

^{1}H NMR (400 MHz, $CDCl_3$) of **55** (*see procedure*)

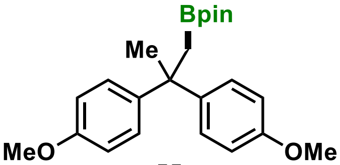

55

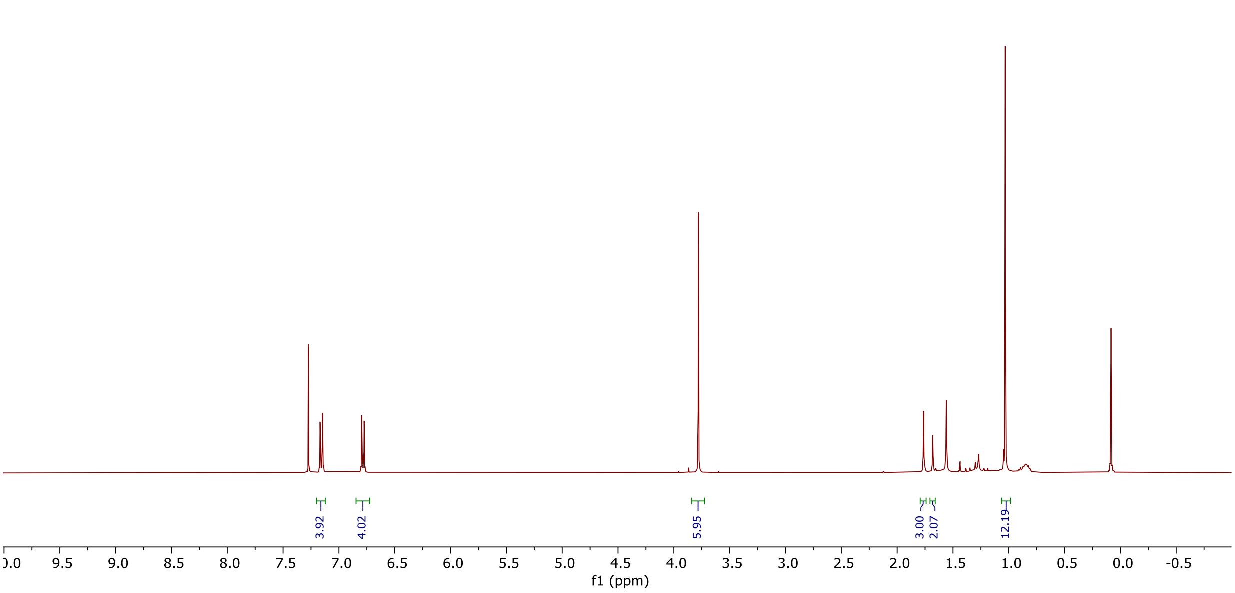

^{13}C NMR (101 MHz, $CDCl_3$) of **55**

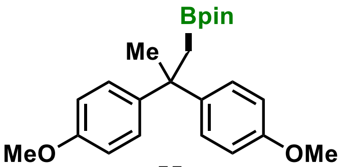

55

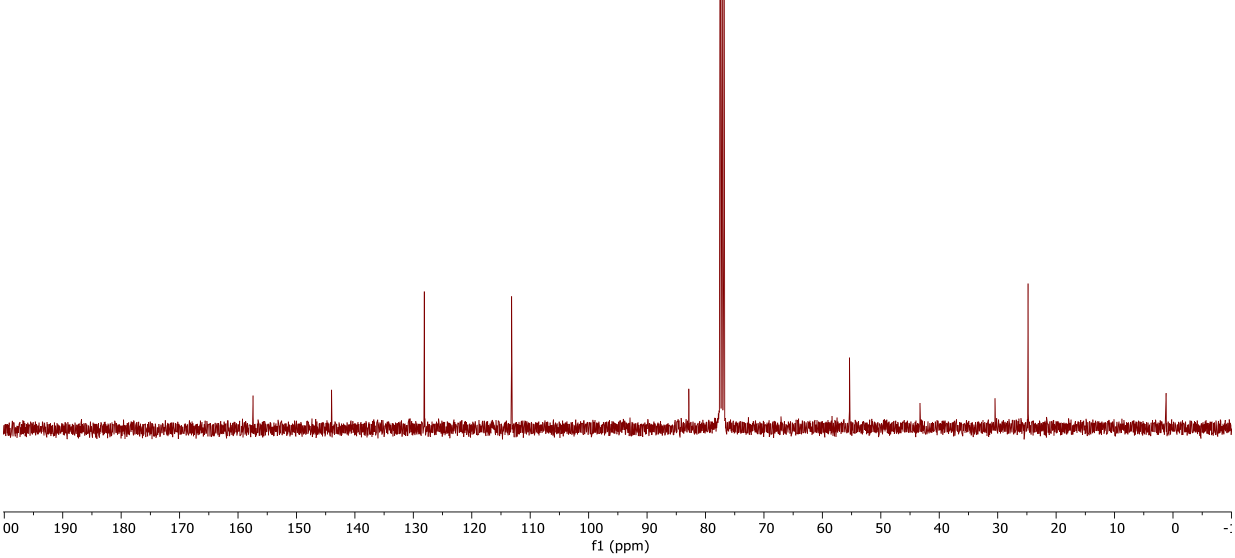

^{11}B NMR (128 MHz, $CDCl_3$) of **55**

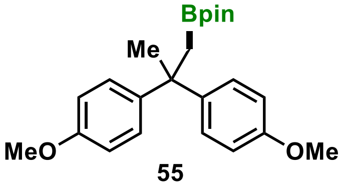

55

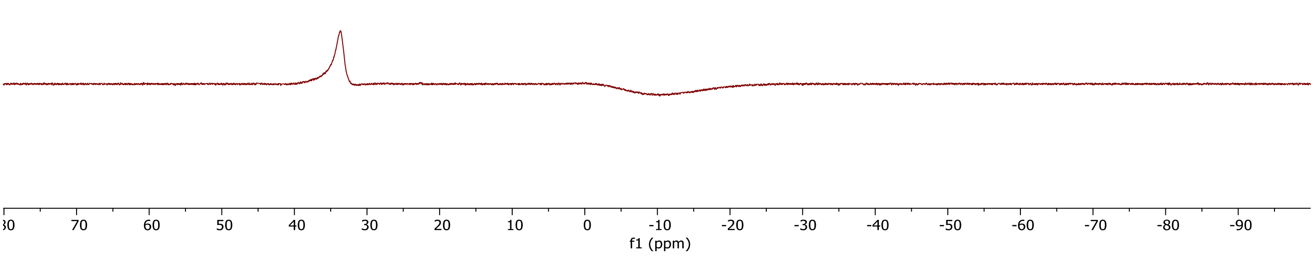

^{1}H NMR (400 MHz, $CDCl_3$) of **56-S** (*see procedure*)

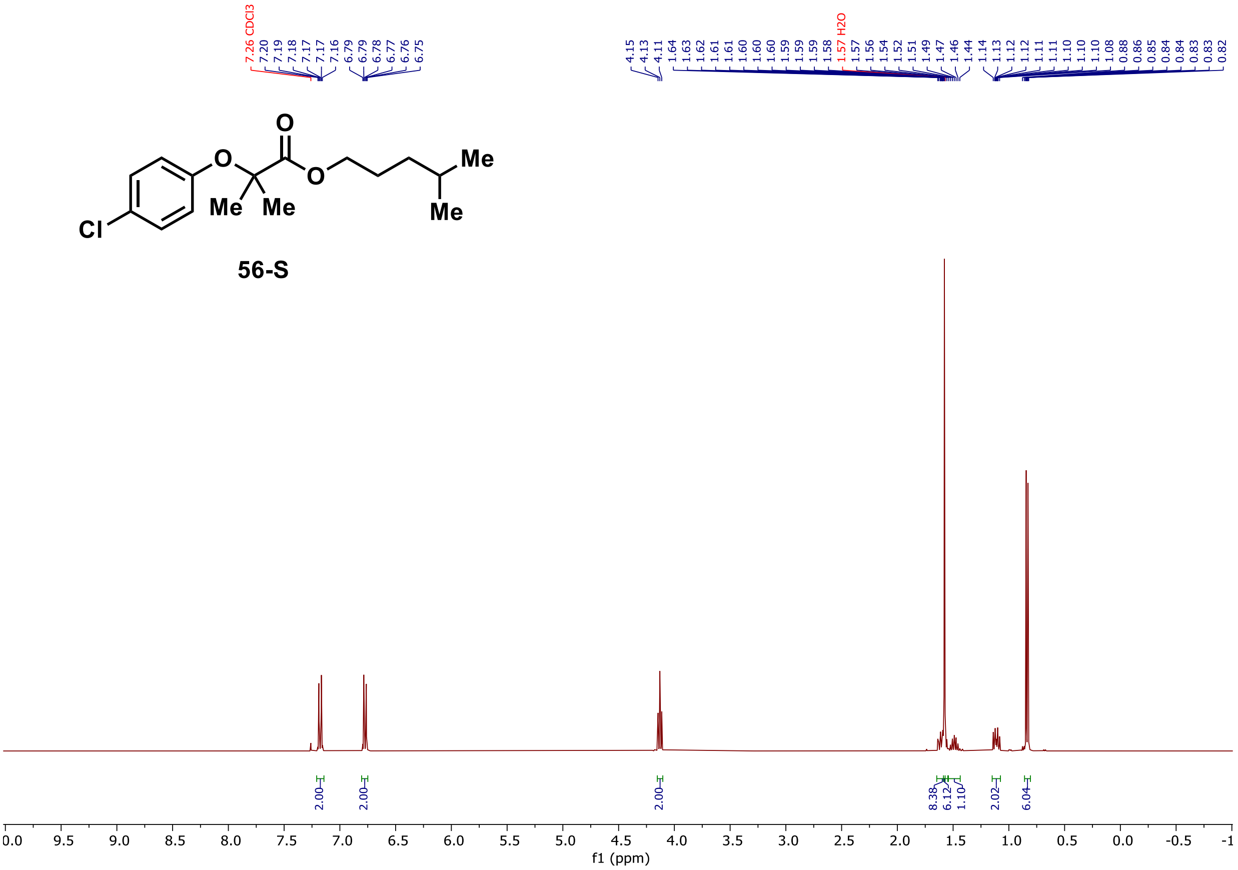

56-S

^{13}C NMR (101 MHz, $CDCl_3$) of **56-S**

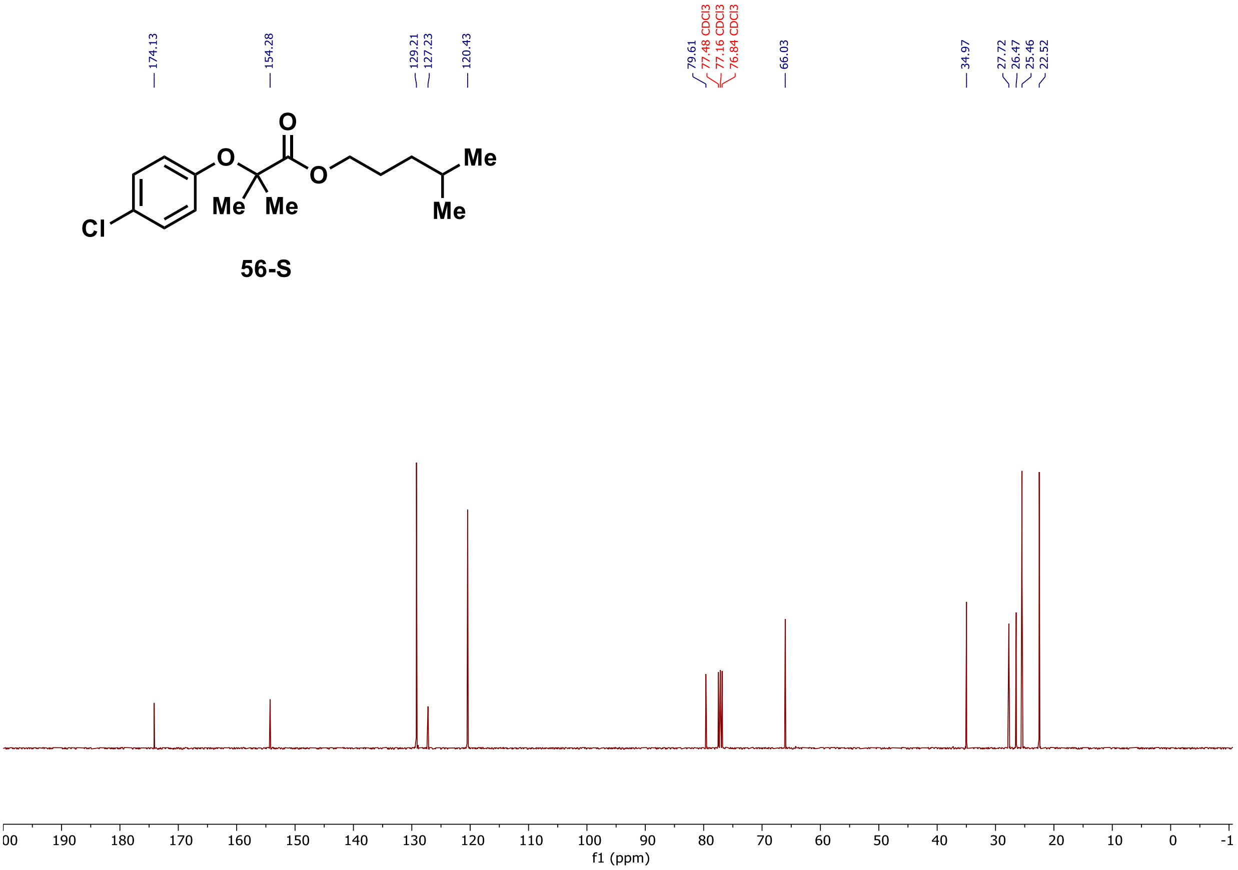

56-S

^{1}H NMR (400 MHz, $CDCl_3$) of **56** (***see procedure***)

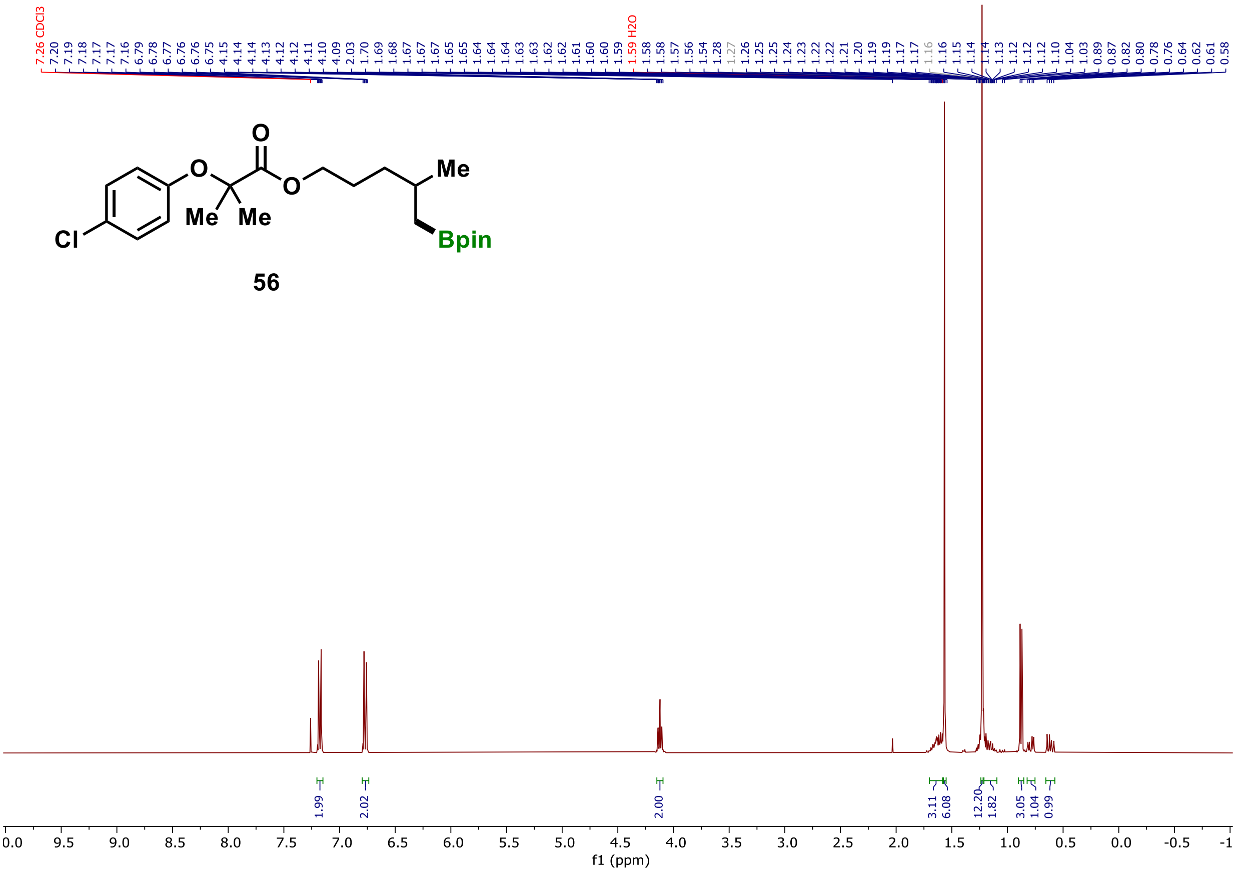

^{13}C NMR (101 MHz, $CDCl_3$) of **56**

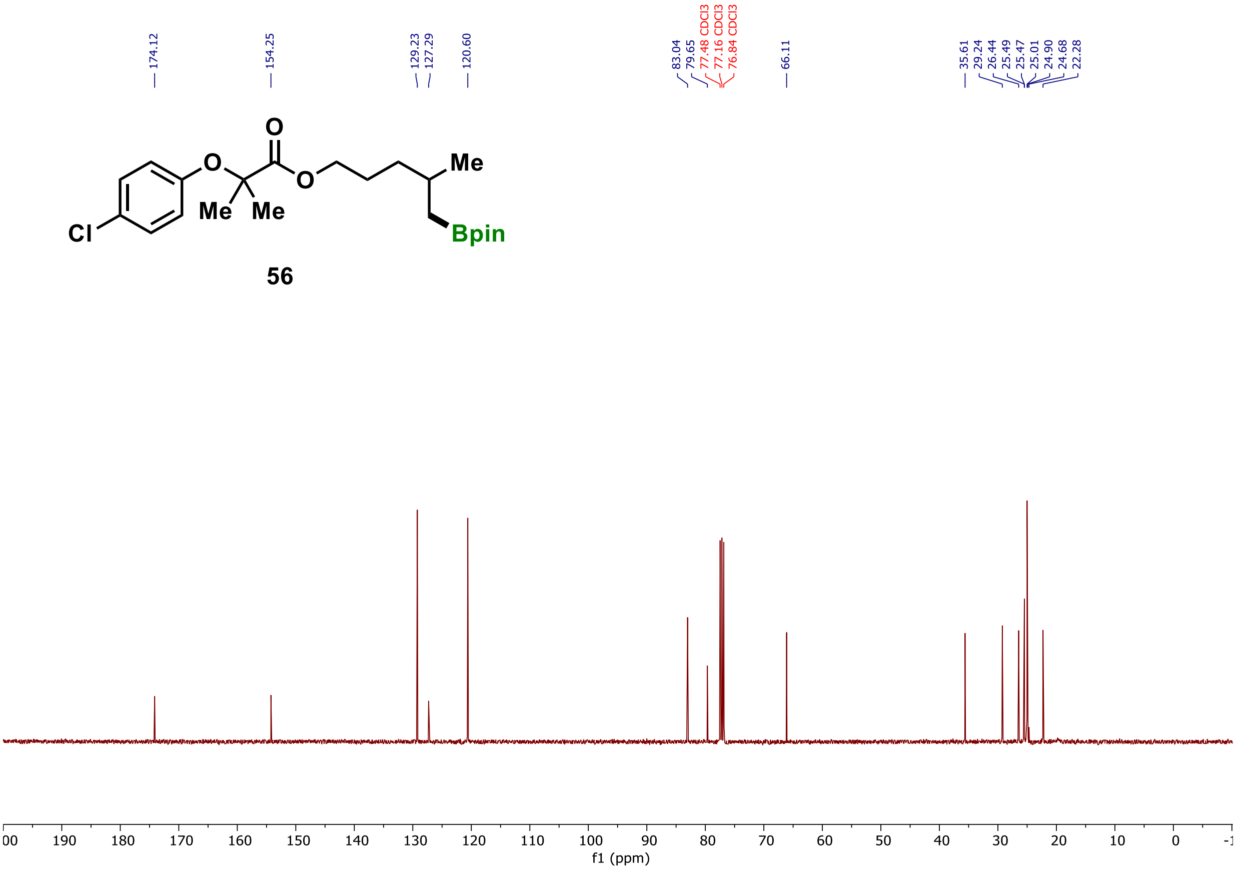

^{11}B NMR (128 MHz, $CDCl_3$) of **56**

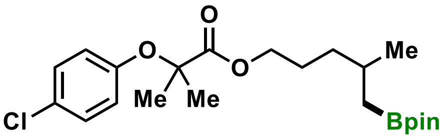

56

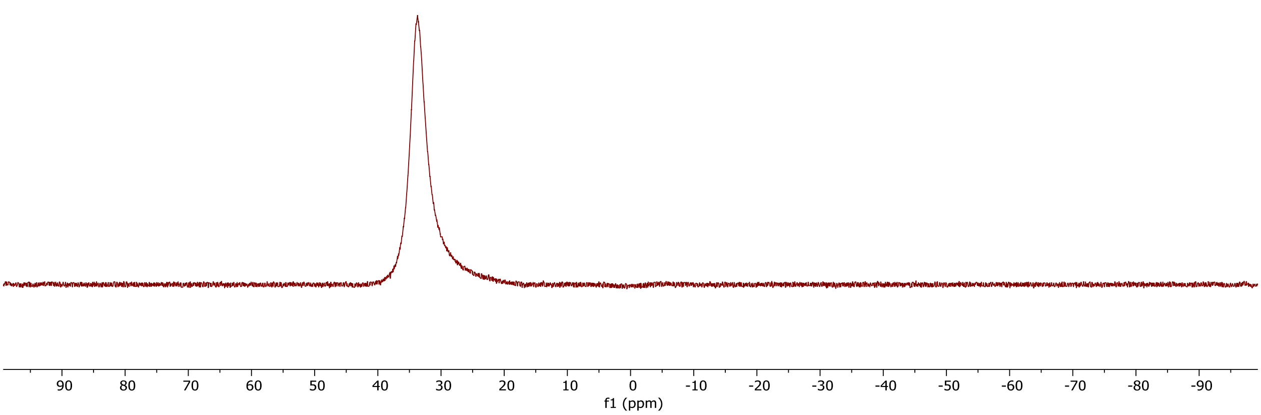

^{1}H NMR (400 MHz, $CDCl_3$) of **57-S** (***see procedure***)

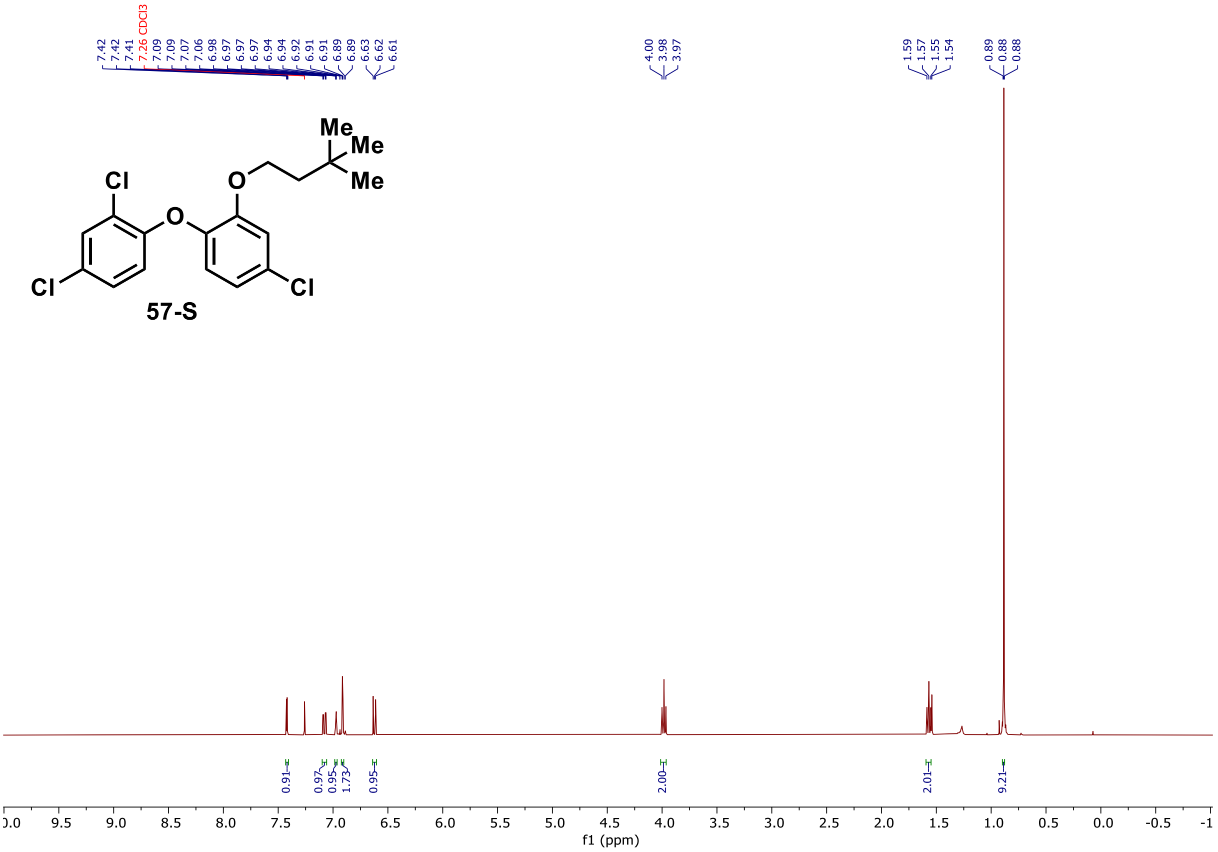

^{13}C NMR (101 MHz, $CDCl_3$) of **57-S**

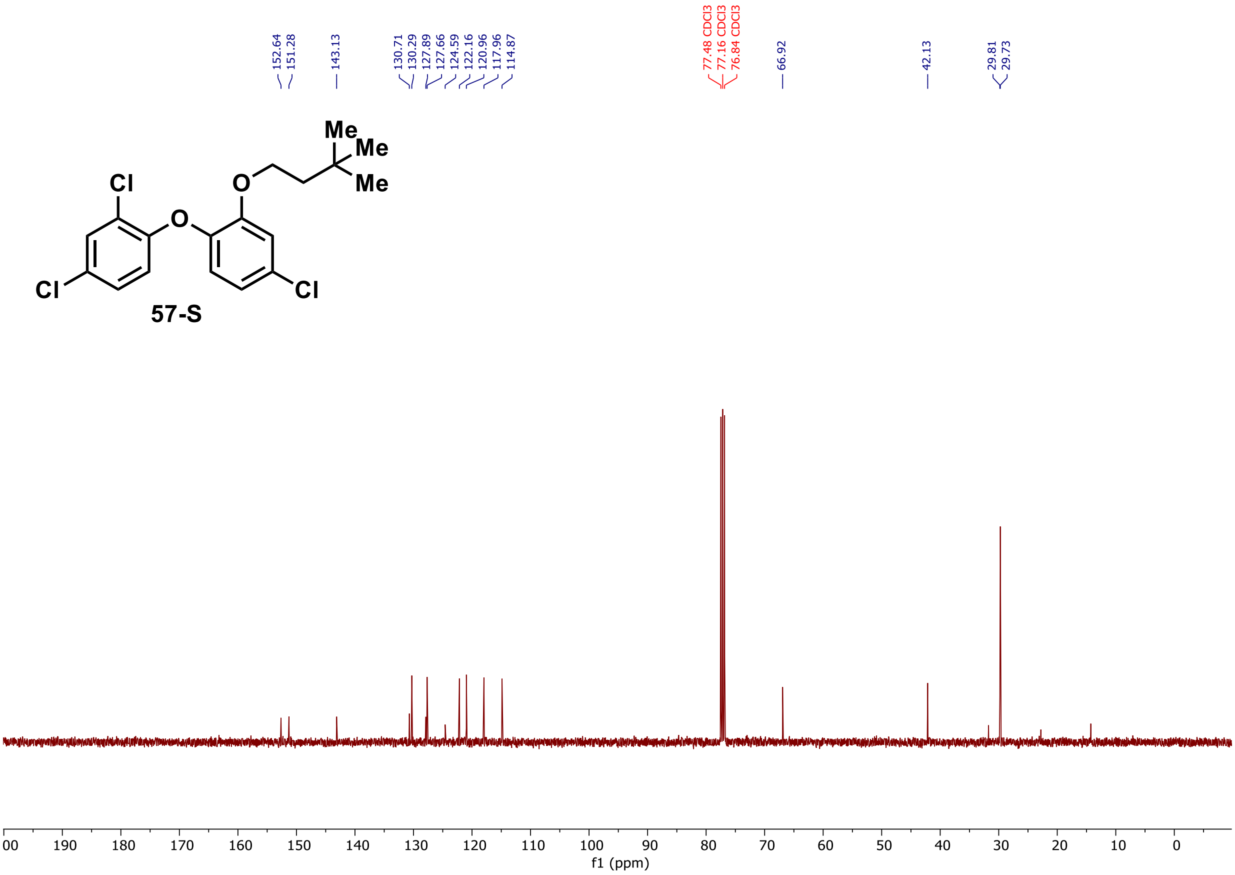

^{1}H NMR (400 MHz, $CDCl_3$) of **57** (*see procedure*)

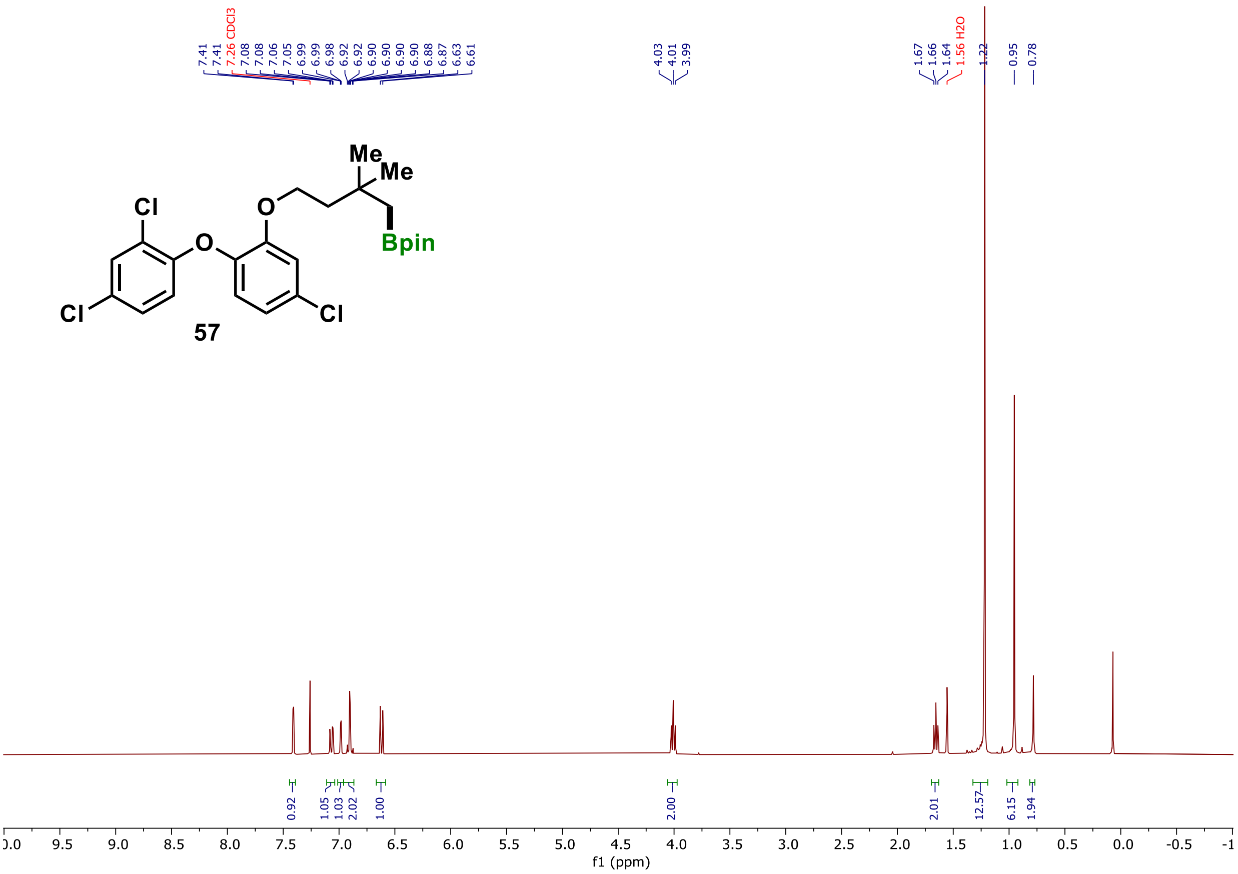

^{13}C NMR (101 MHz, $CDCl_3$) of **57**

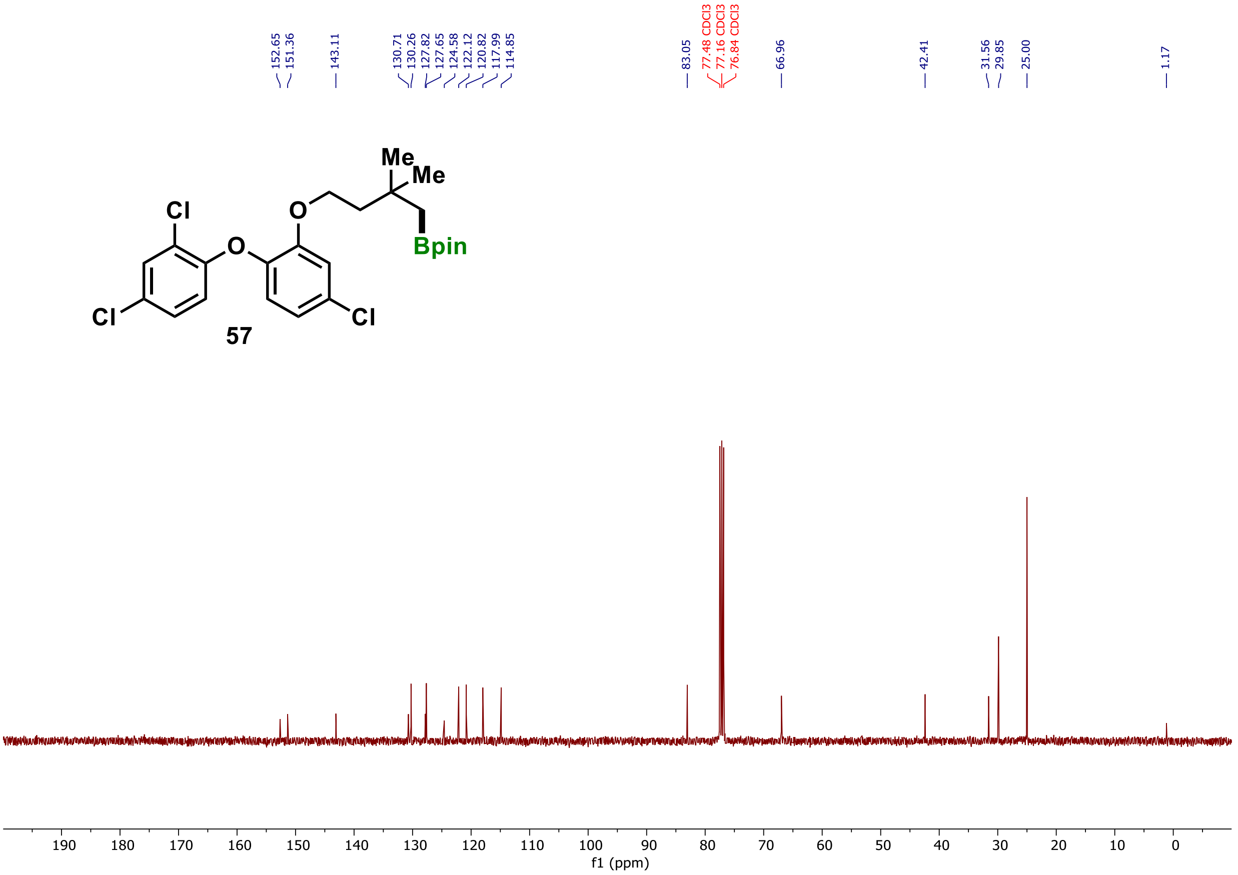

^{11}B NMR (128 MHz, $CDCl_3$) of **57**

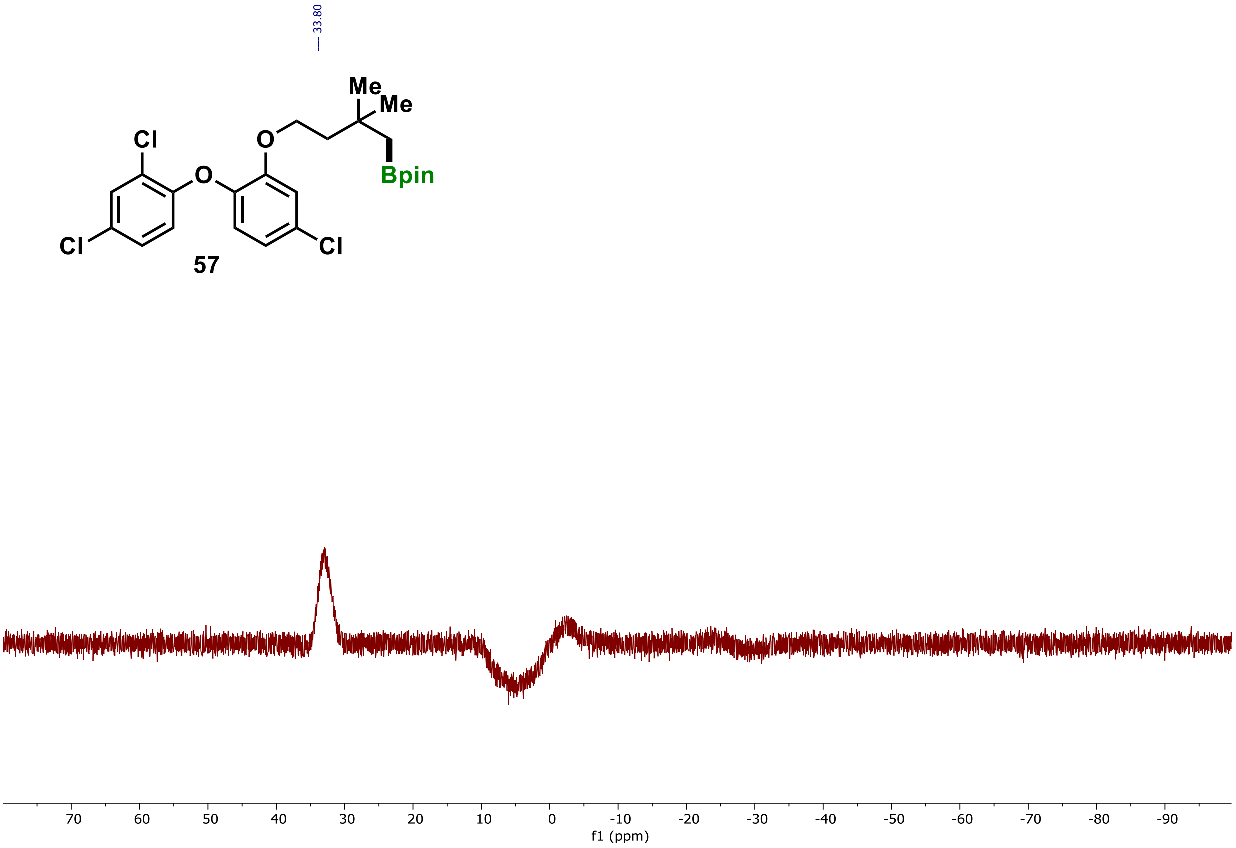

^{1}H NMR (400 MHz, $CDCl_3$) of **58-S** (*see procedure*)

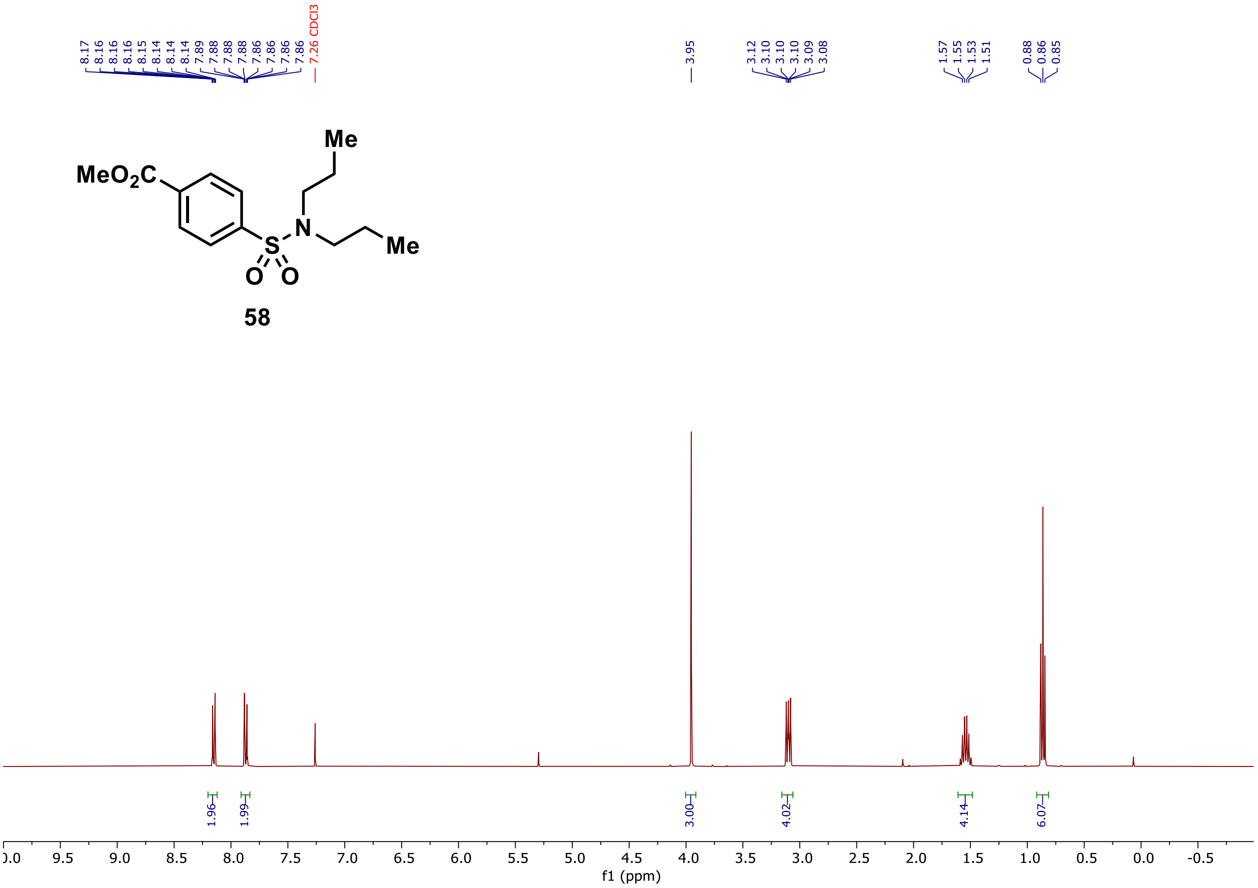

^{13}C NMR (101 MHz, $CDCl_3$) of **58-S**

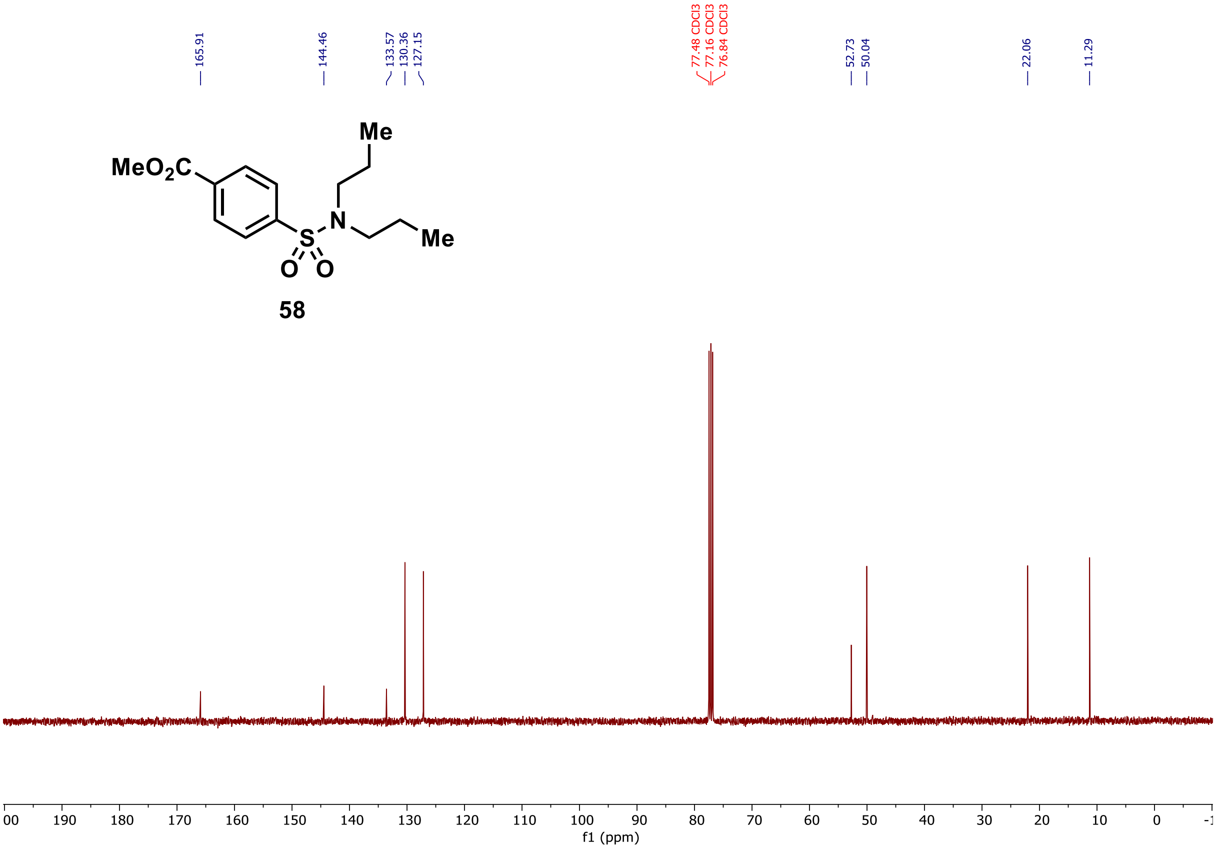

^{1}H NMR (400 MHz, $CDCl_3$) of **58** (***see procedure***)

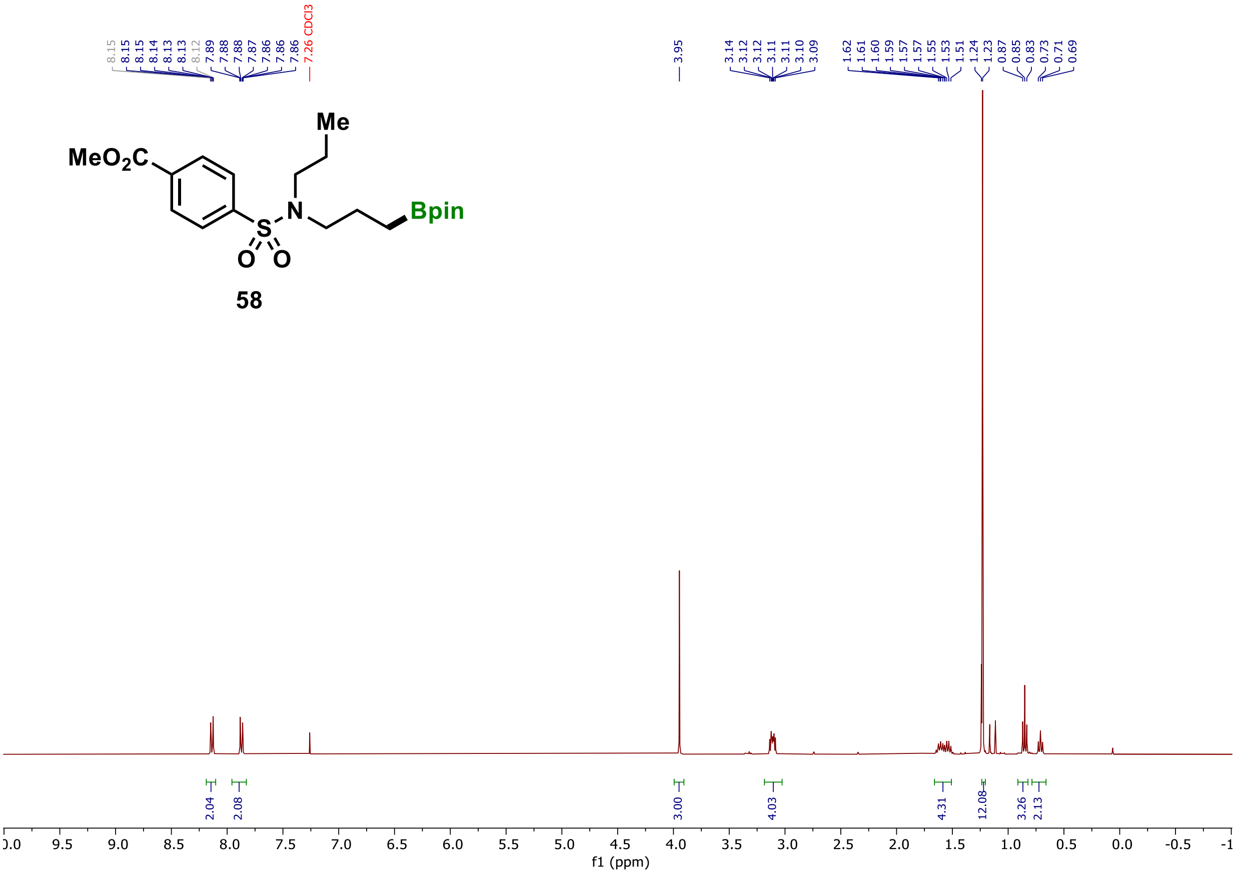

^{13}C NMR (101 MHz, $CDCl_3$) of **58**

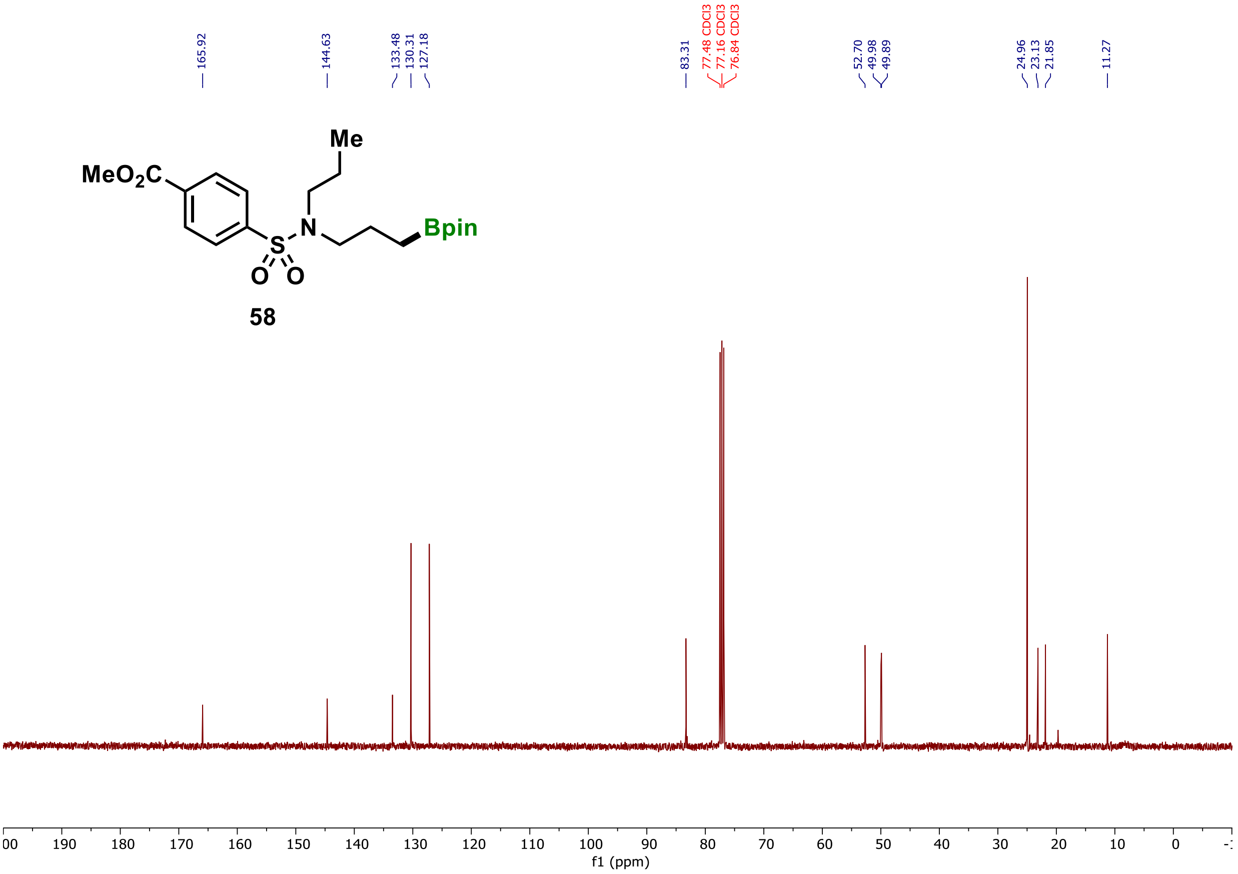

^{11}B NMR (128 MHz, $CDCl_3$) of **58**

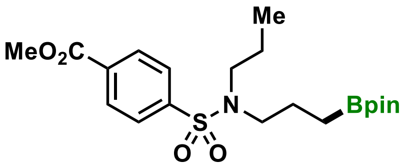

58

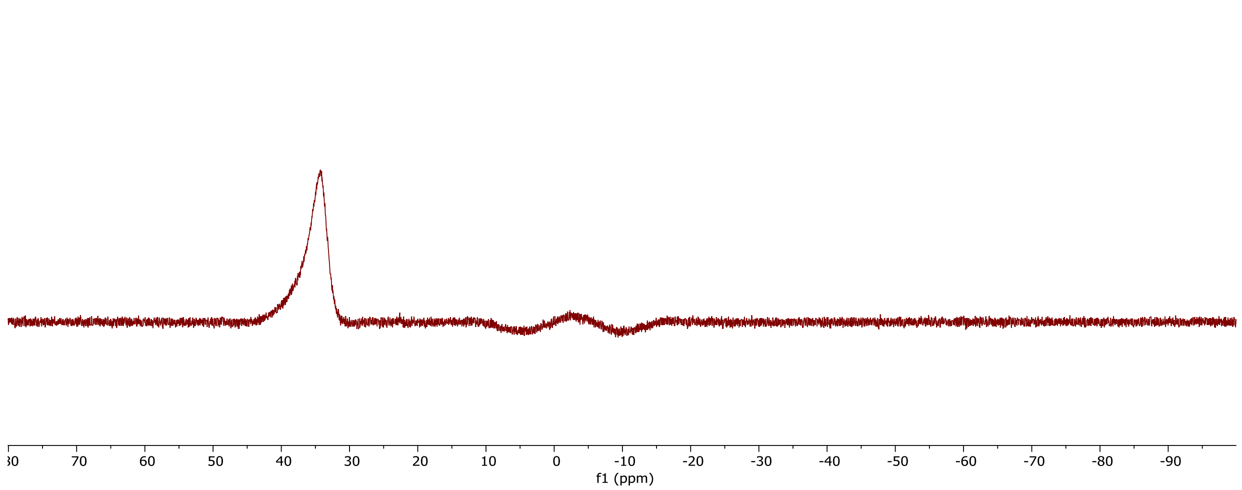

^{1}H NMR (400 MHz, $CDCl_3$) of **59-S** (*see procedure*)

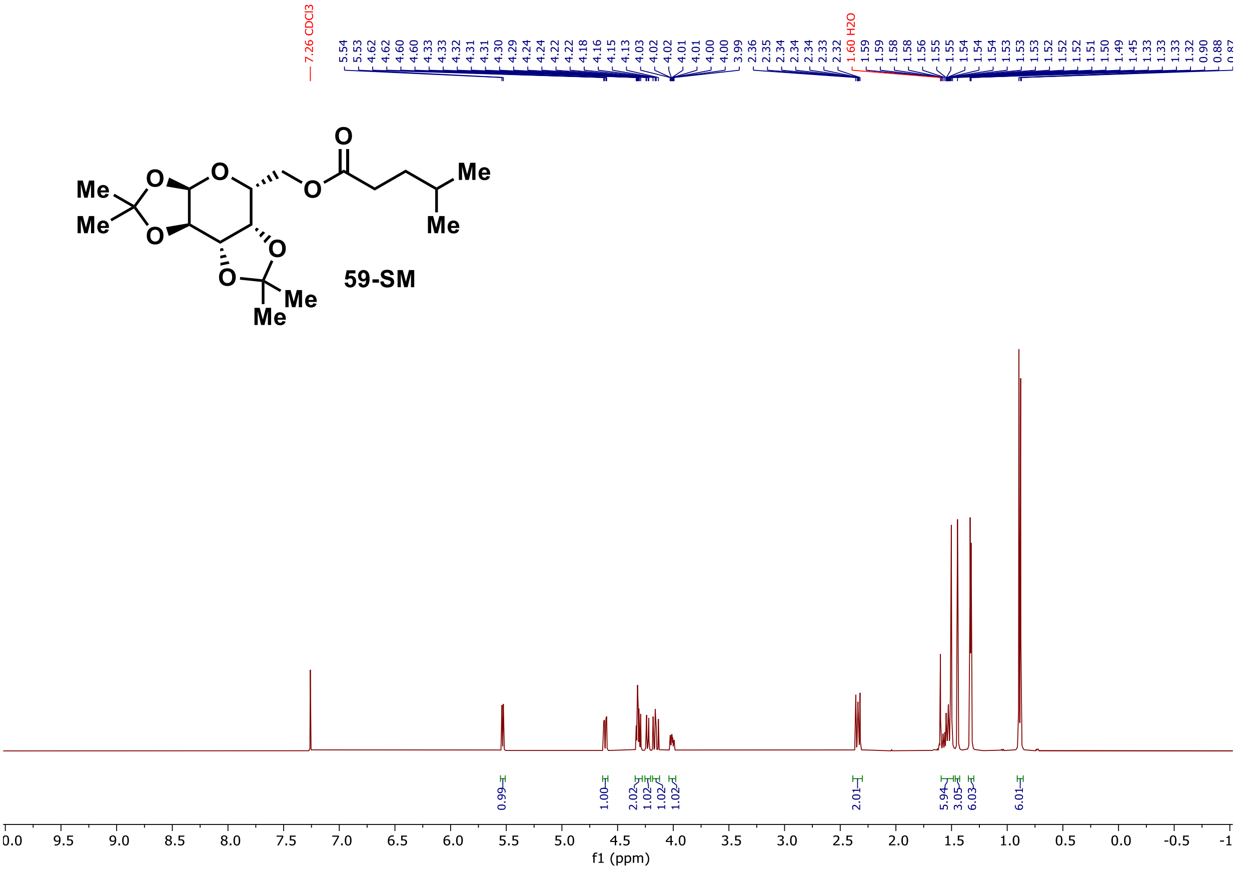

^{13}C NMR (101 MHz, $CDCl_3$) of **59-S**

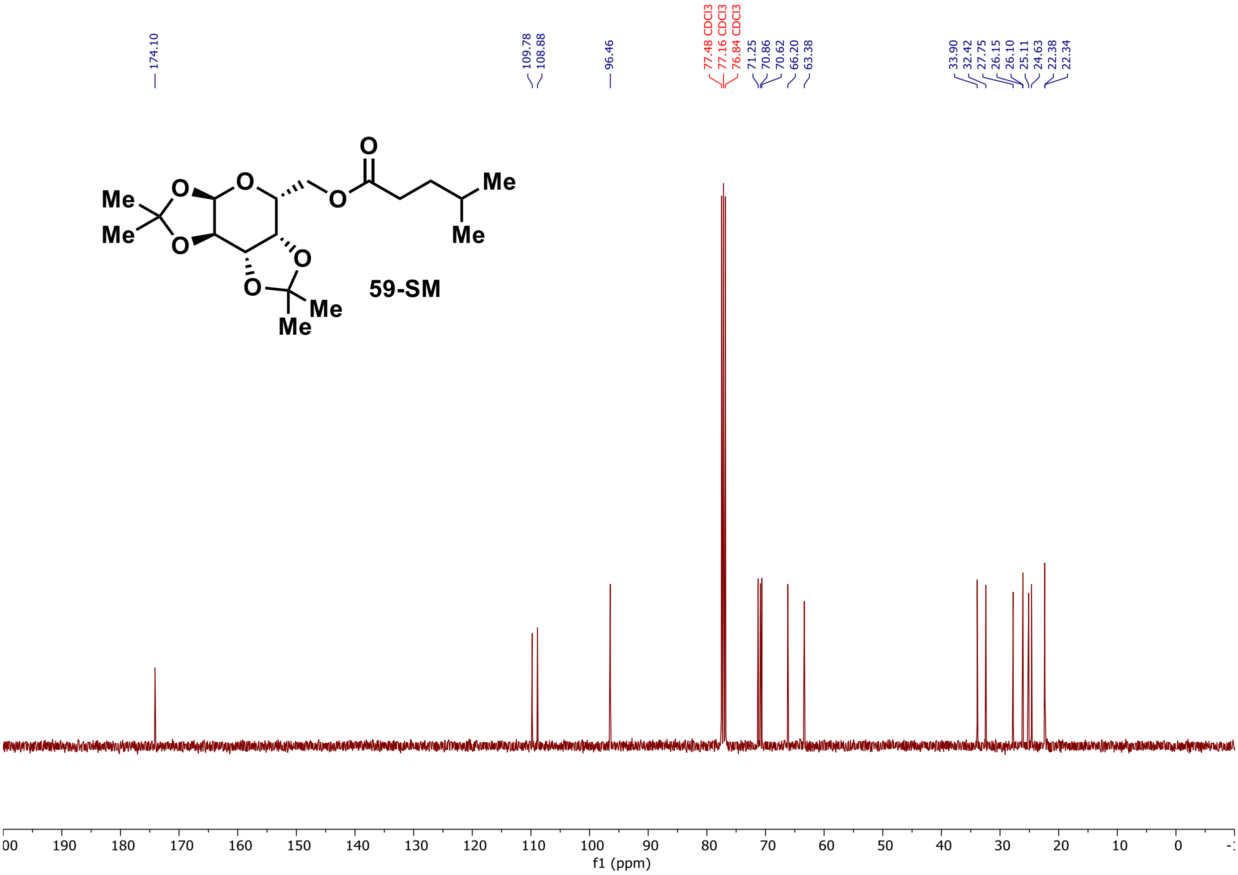

^{1}H NMR (400 MHz, $CDCl_3$) of **59** (***see procedure***)

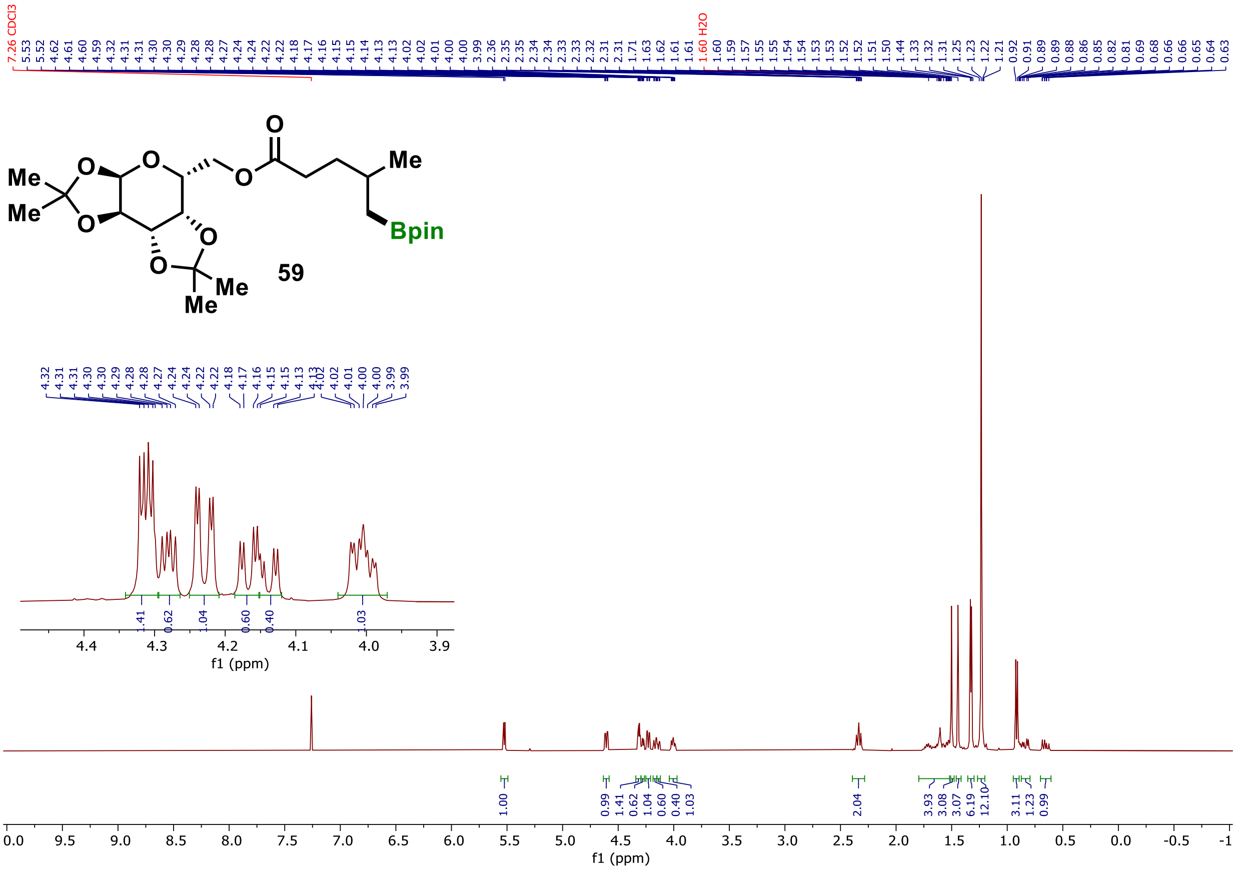

^{13}C NMR (101 MHz, $CDCl_3$) of **59**

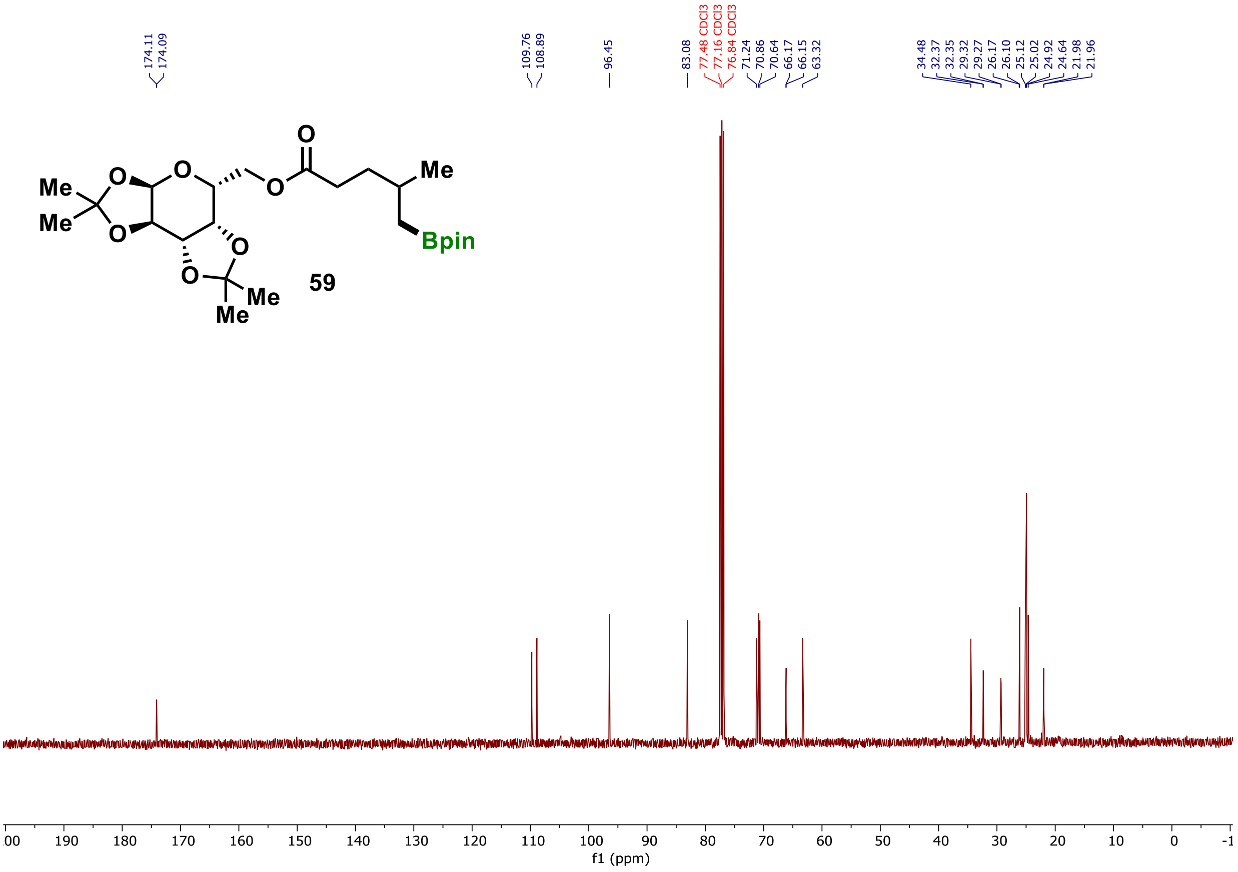

^{1}H NMR (400 MHz, $CDCl_3$) of **60-S** (***see procedure***)

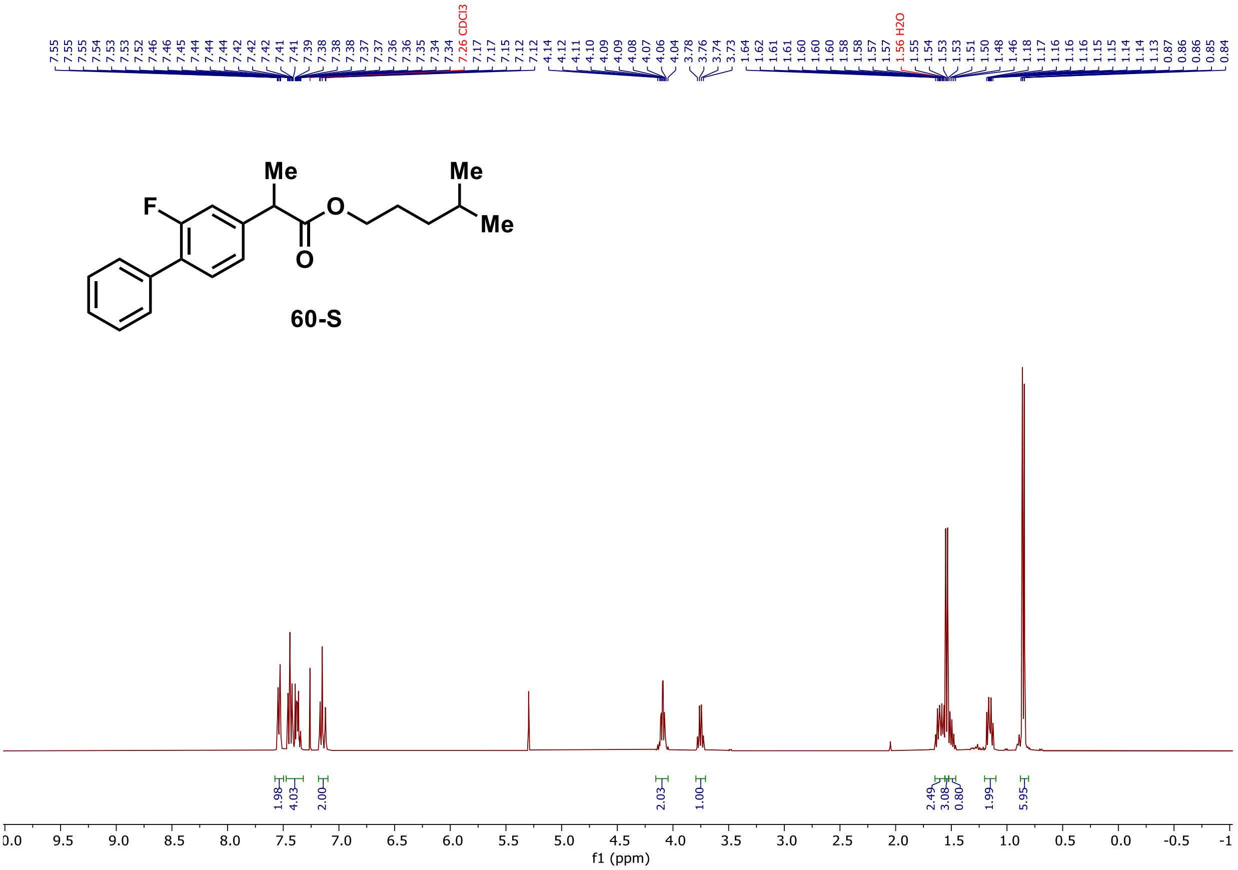

^{13}C NMR (101 MHz, $CDCl_3$) of **60-S**

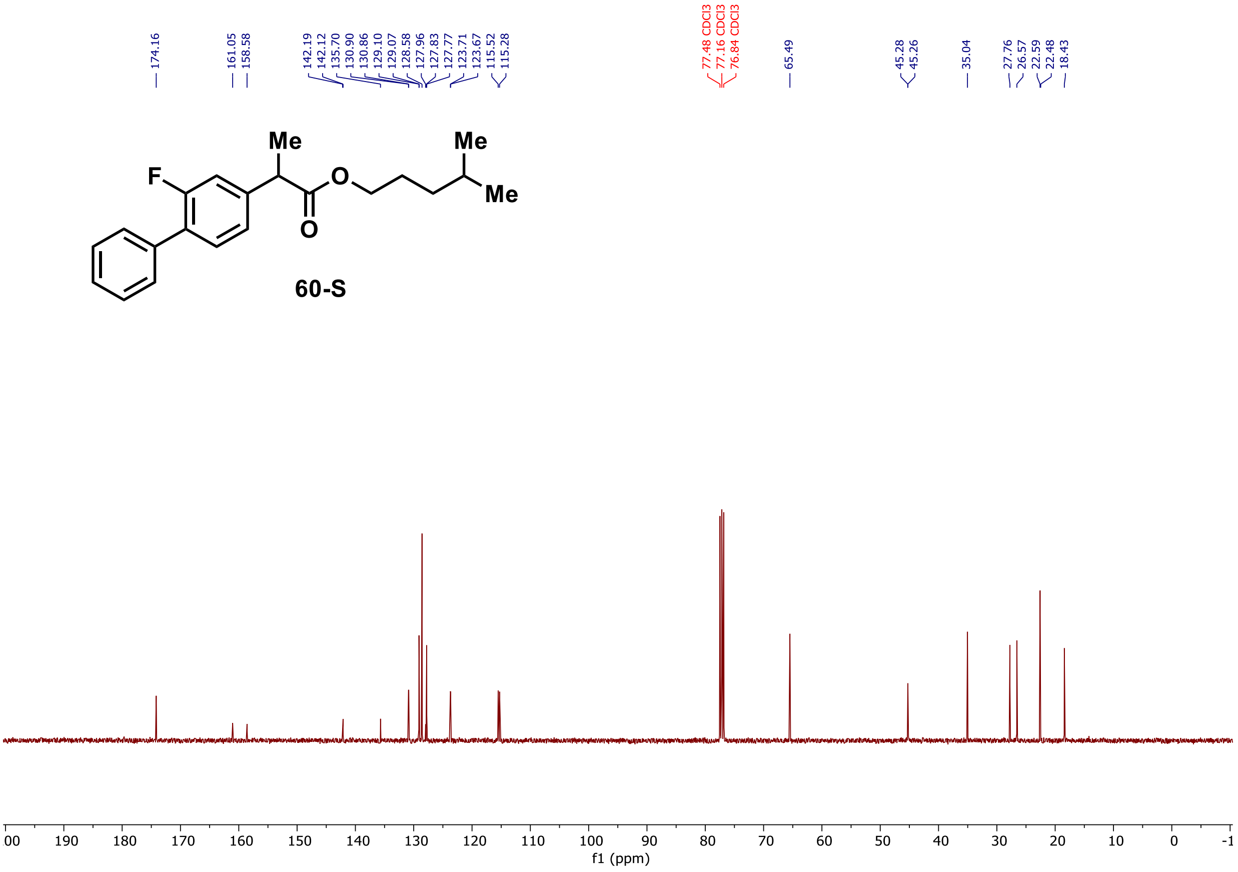

^{19}F NMR (377 MHz, $CDCl_3$) of **60-S**

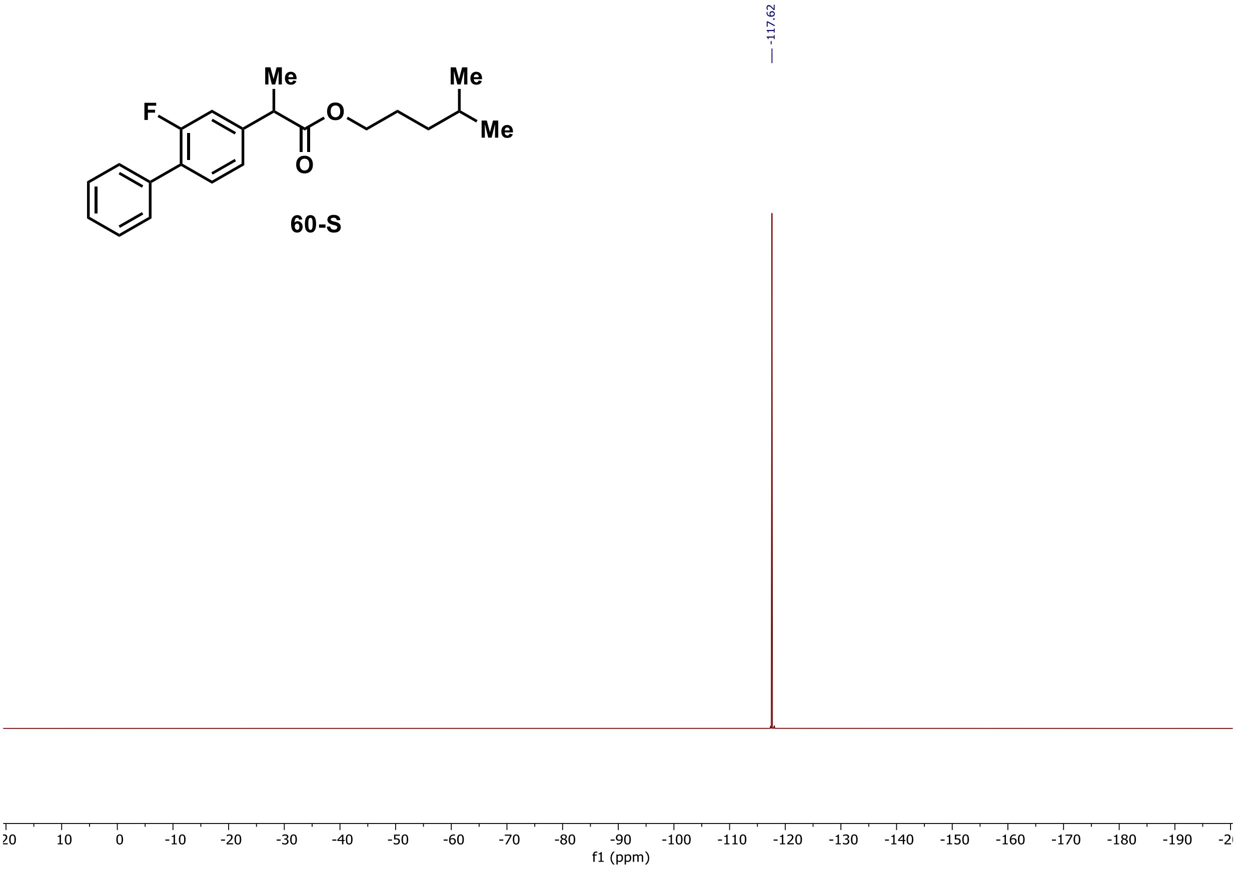

^{1}H NMR (400 MHz, $CDCl_3$) of **60** (*see procedure*)

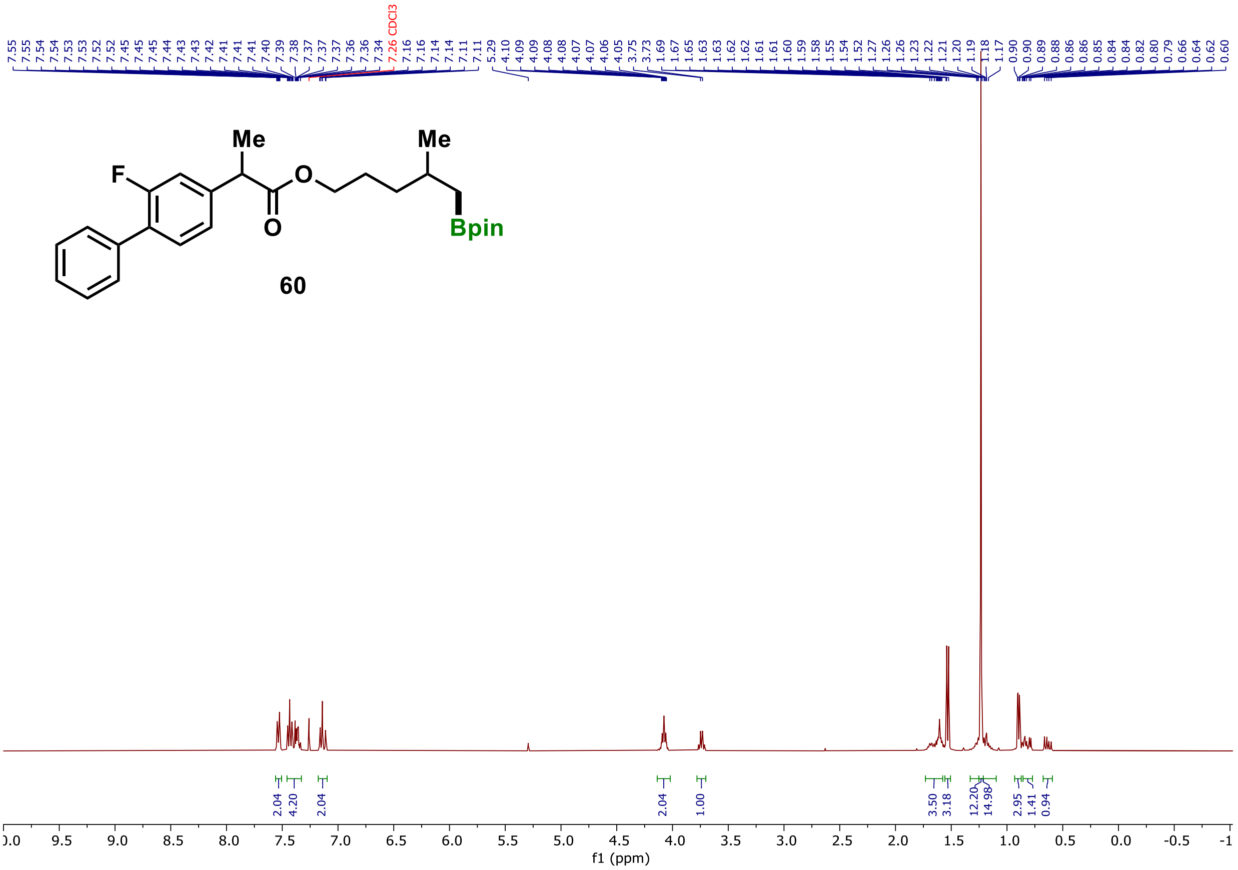

^{13}C NMR (101 MHz, $CDCl_3$) of **60**

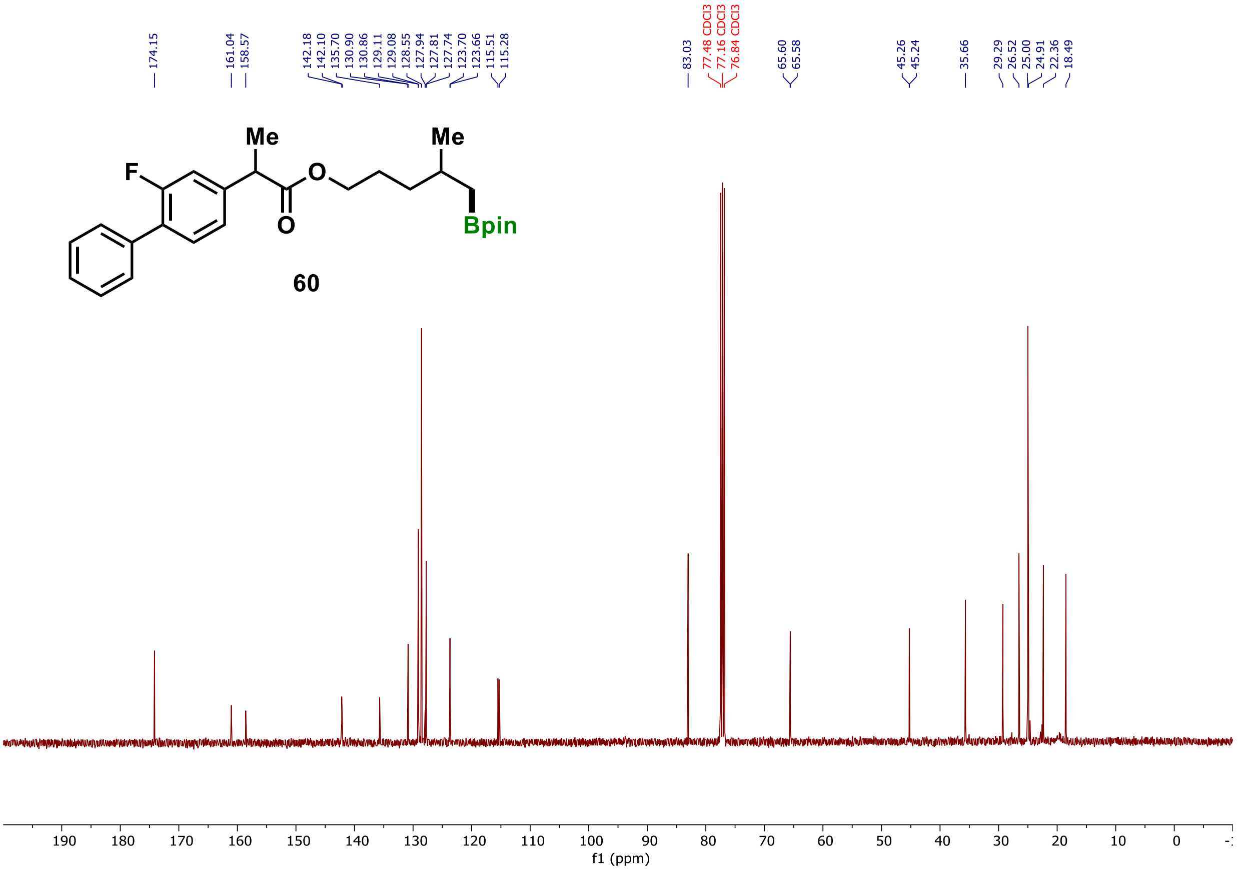

^{19}F NMR (377 MHz, $CDCl_3$) of **60**

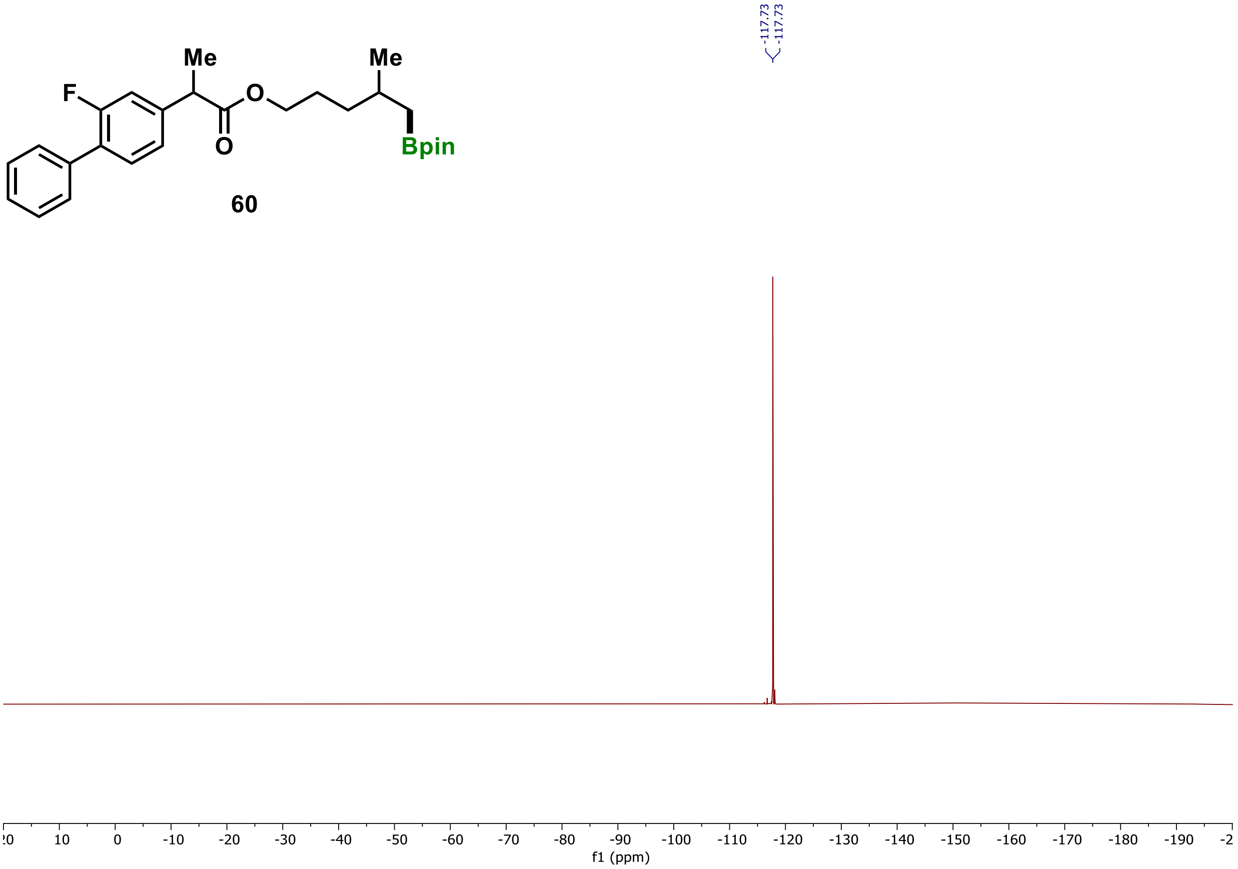

^{11}B NMR (128 MHz, $CDCl_3$) of **60**

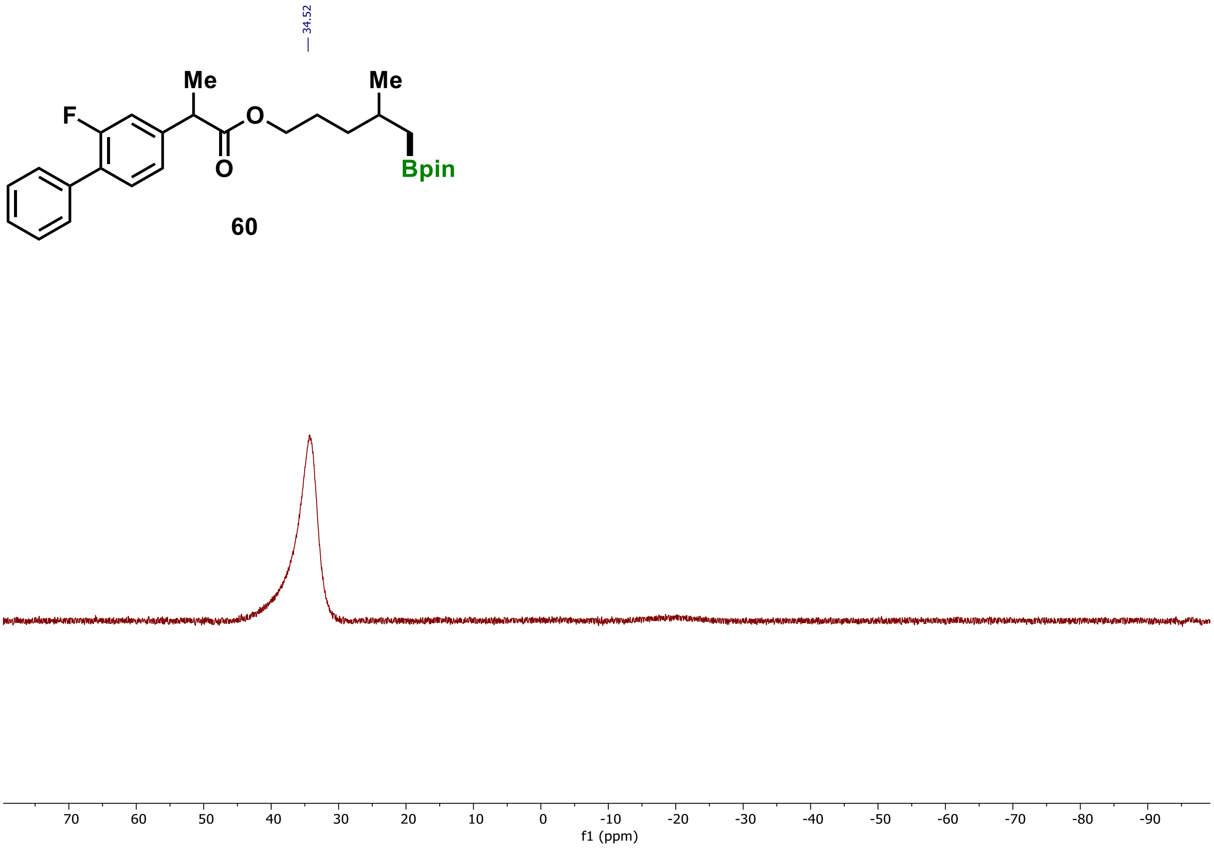

^{1}H NMR (400 MHz, $CDCl_3$) of **61-S** (*see procedure*)

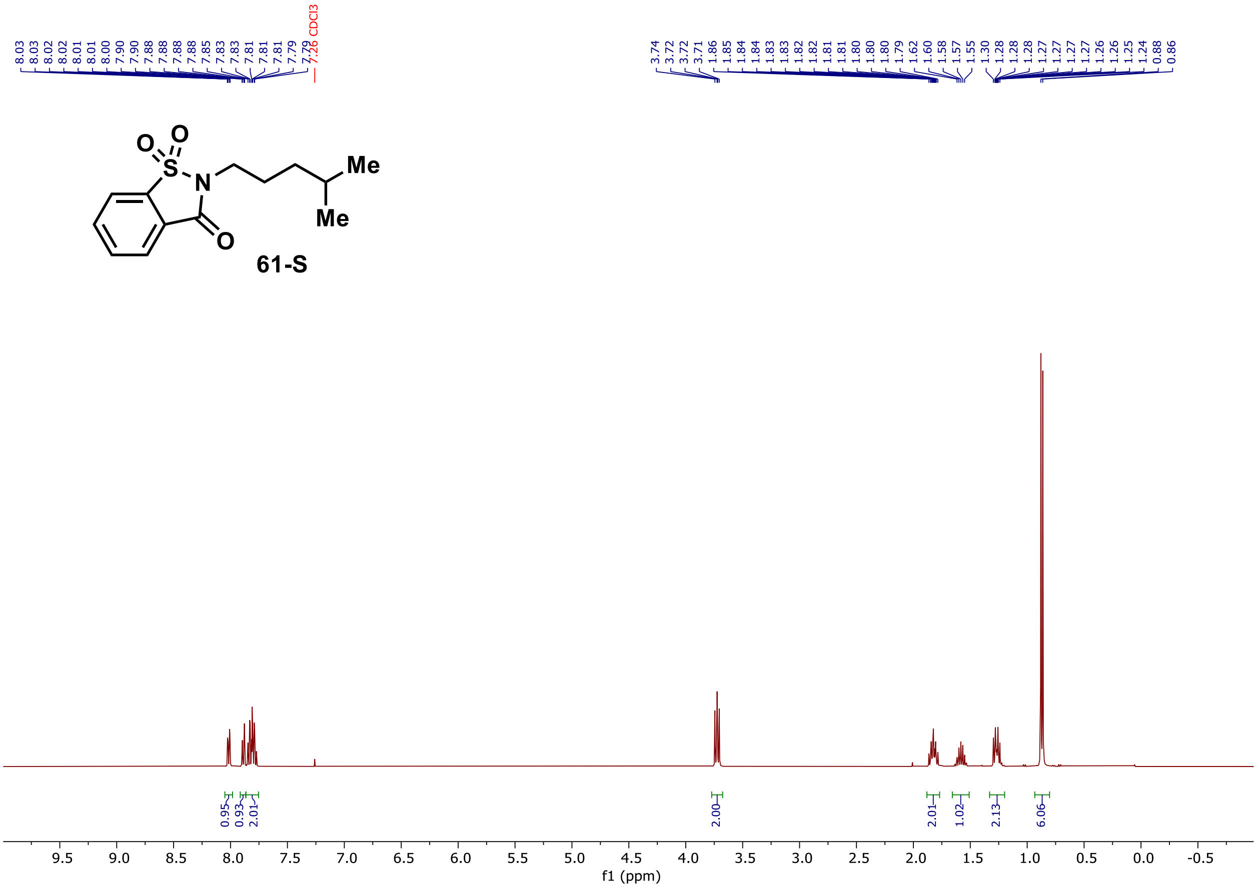

^{13}C NMR (101 MHz, $CDCl_3$) of **61-S**

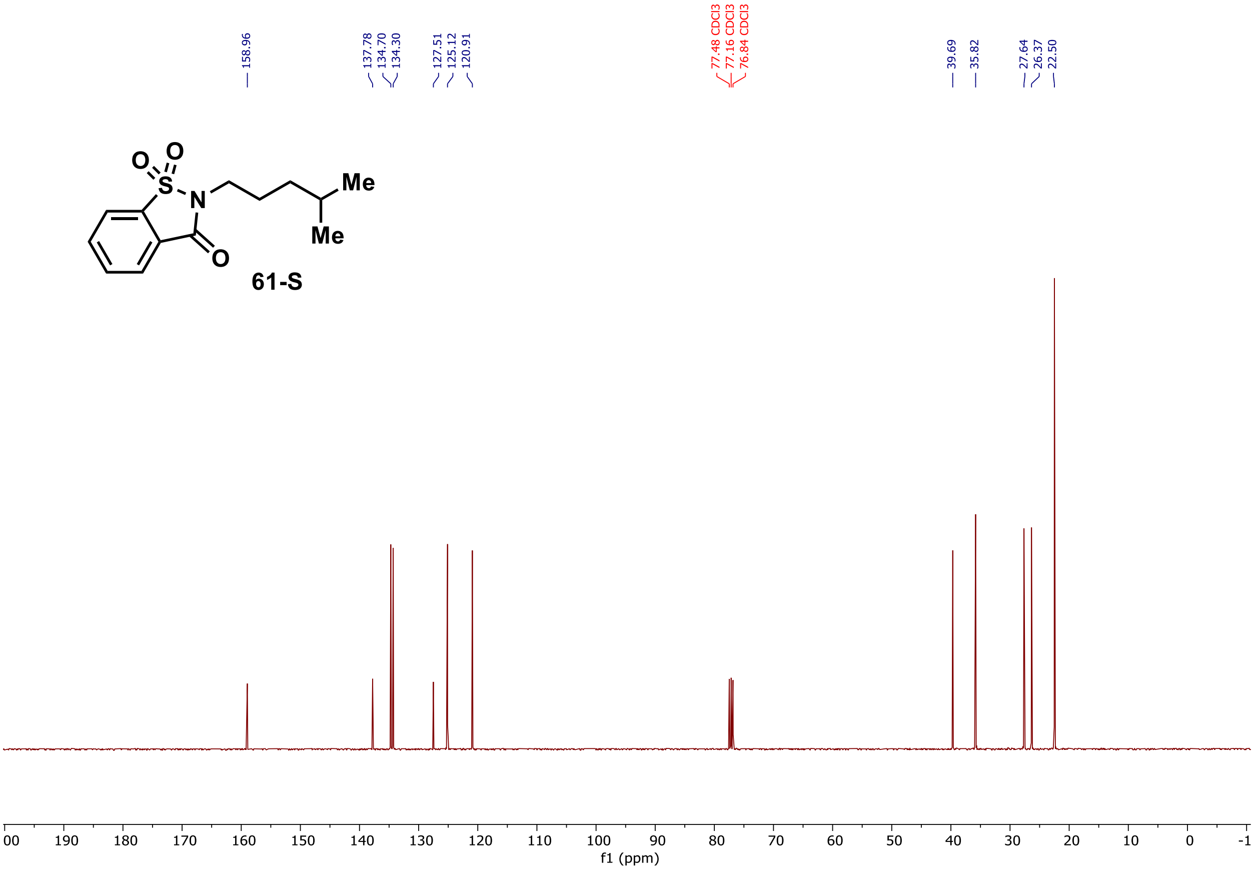

^{1}H NMR (400 MHz, $CDCl_3$) of **61** (*see procedure*)

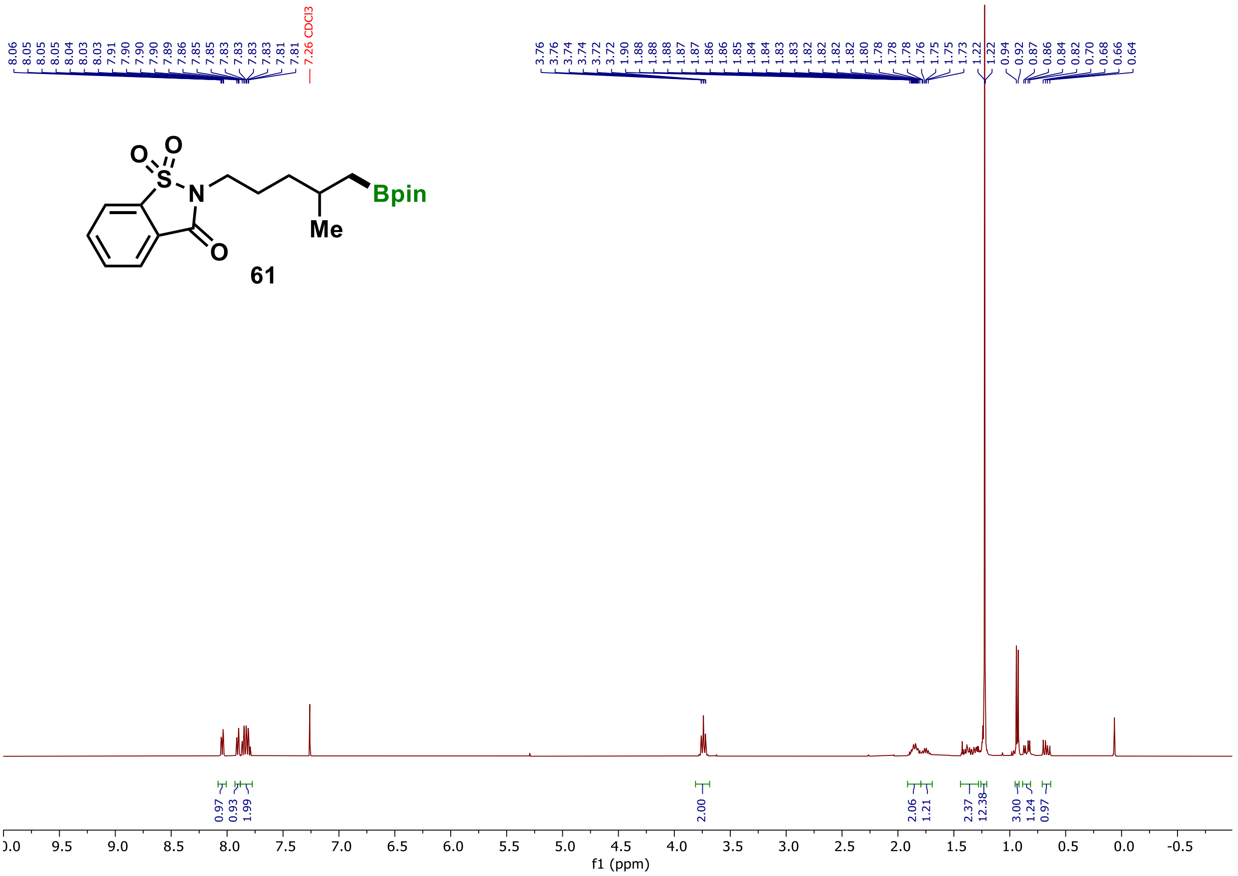

^{13}C NMR (101 MHz, $CDCl_3$) of **61**

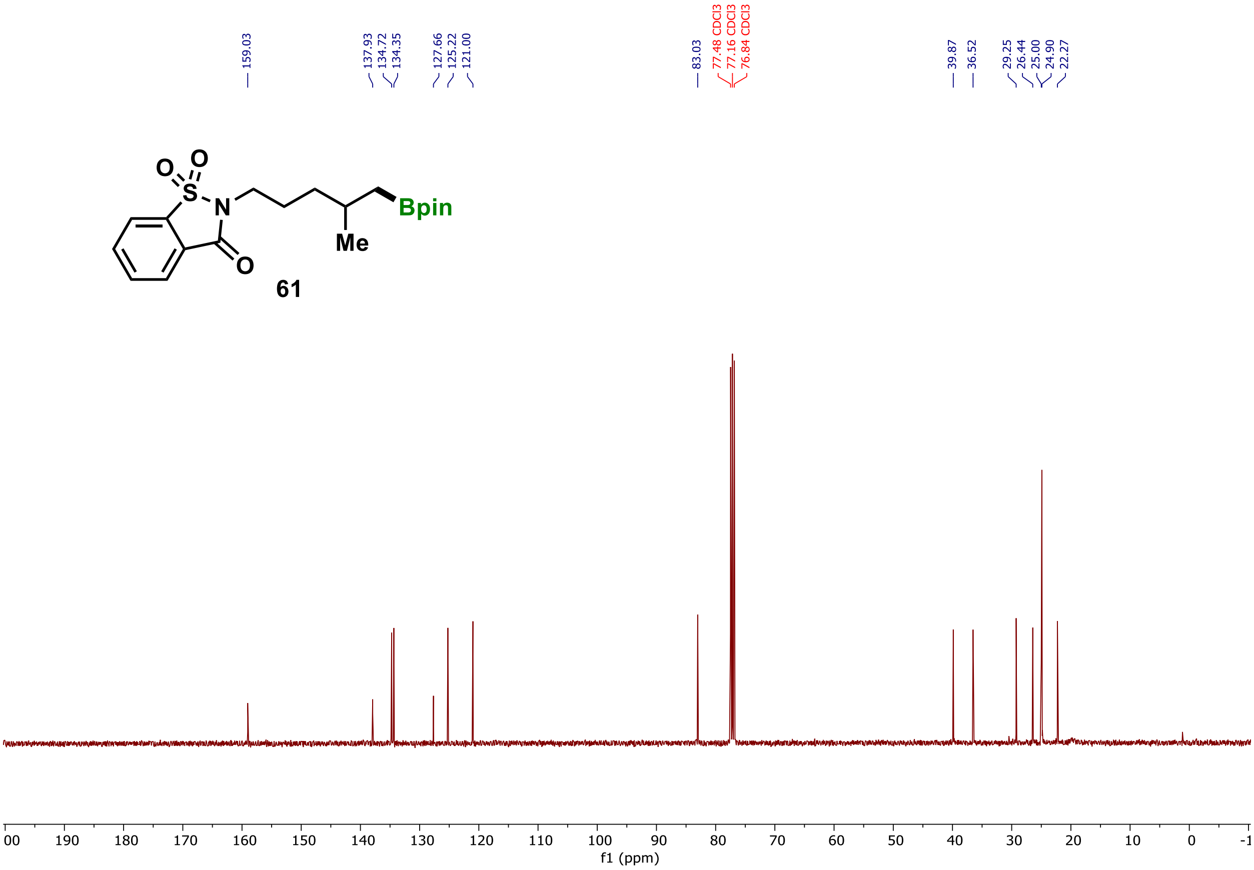

^{11}B NMR (128 MHz, $CDCl_3$) of **61**

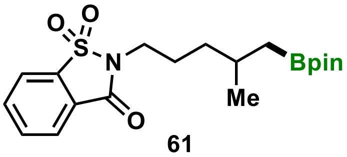

61

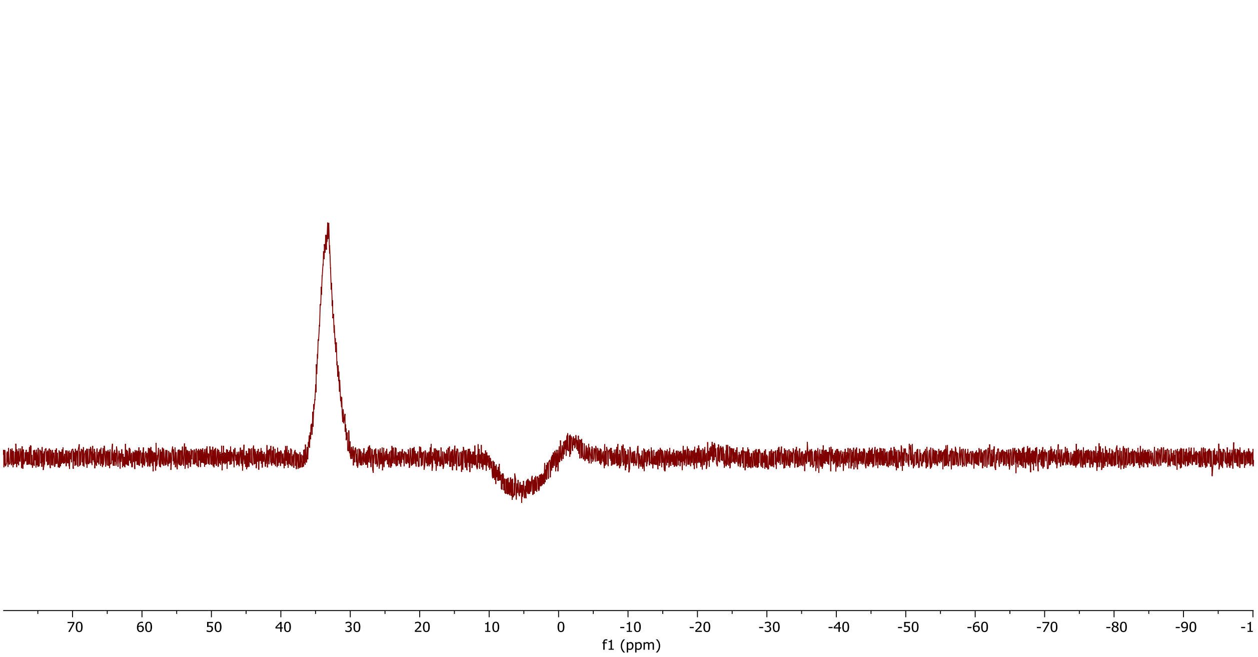

^{1}H NMR (400 MHz, $CDCl_3$) of **62-S** (*see procedure*)

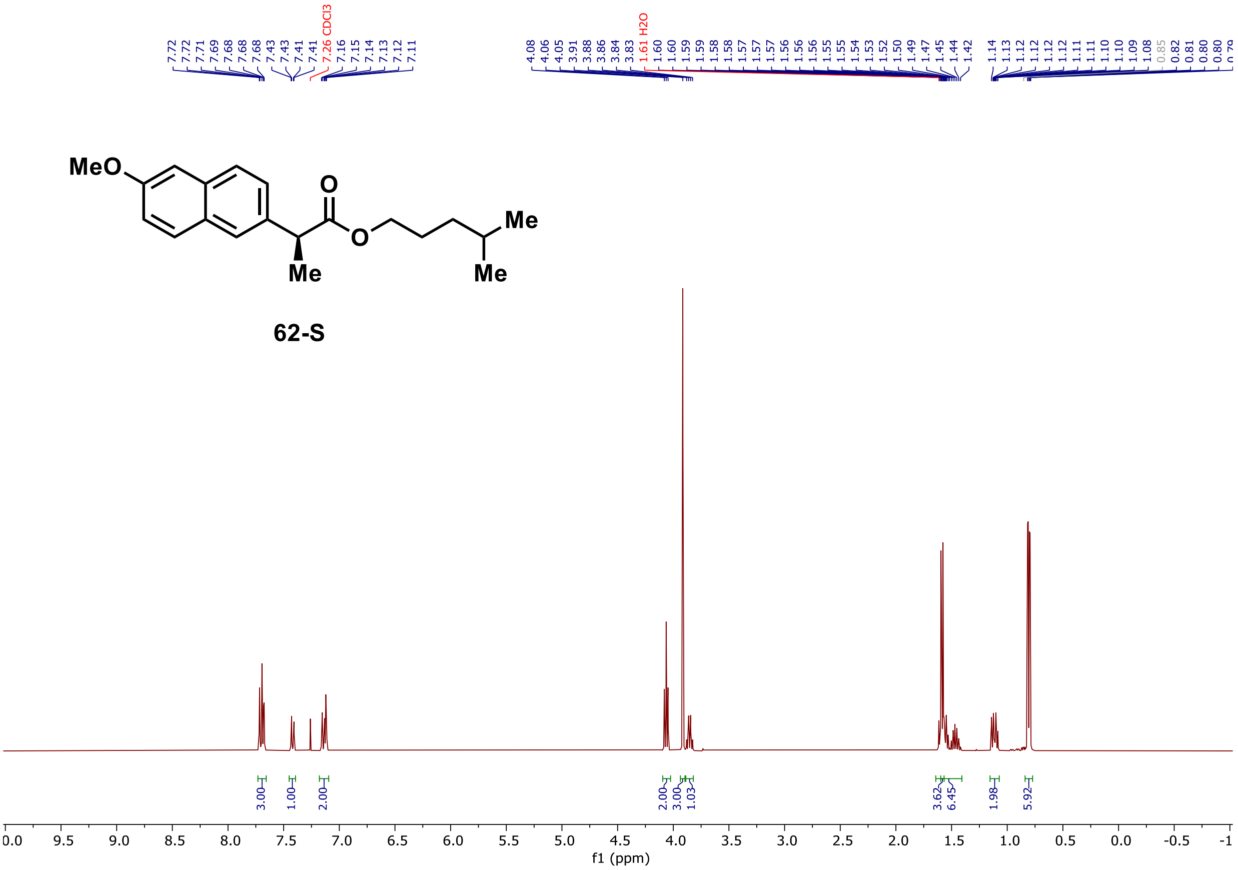

^{13}C NMR (101 MHz, $CDCl_3$) of **62-S**

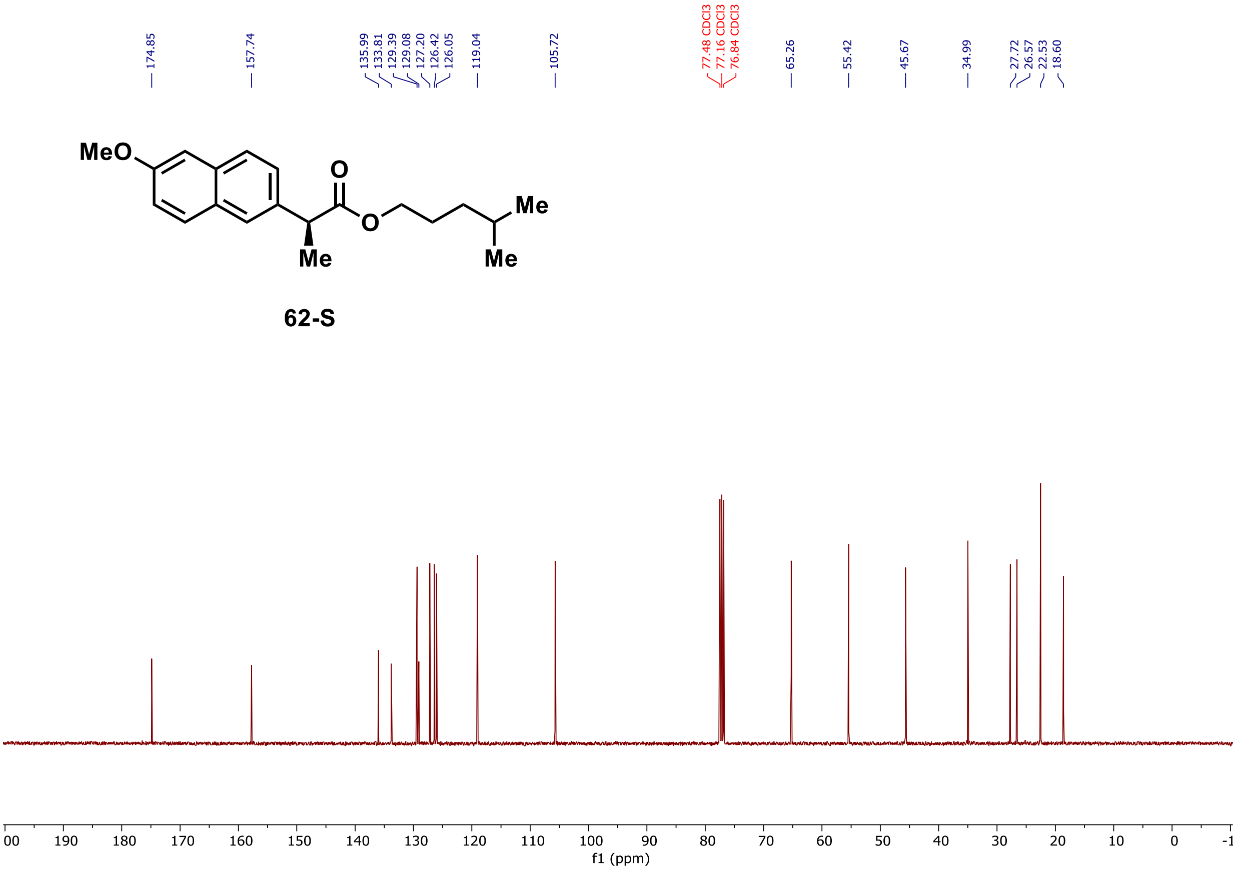

^{1}H NMR (400 MHz, $CDCl_3$) of **62** (*see procedure*)

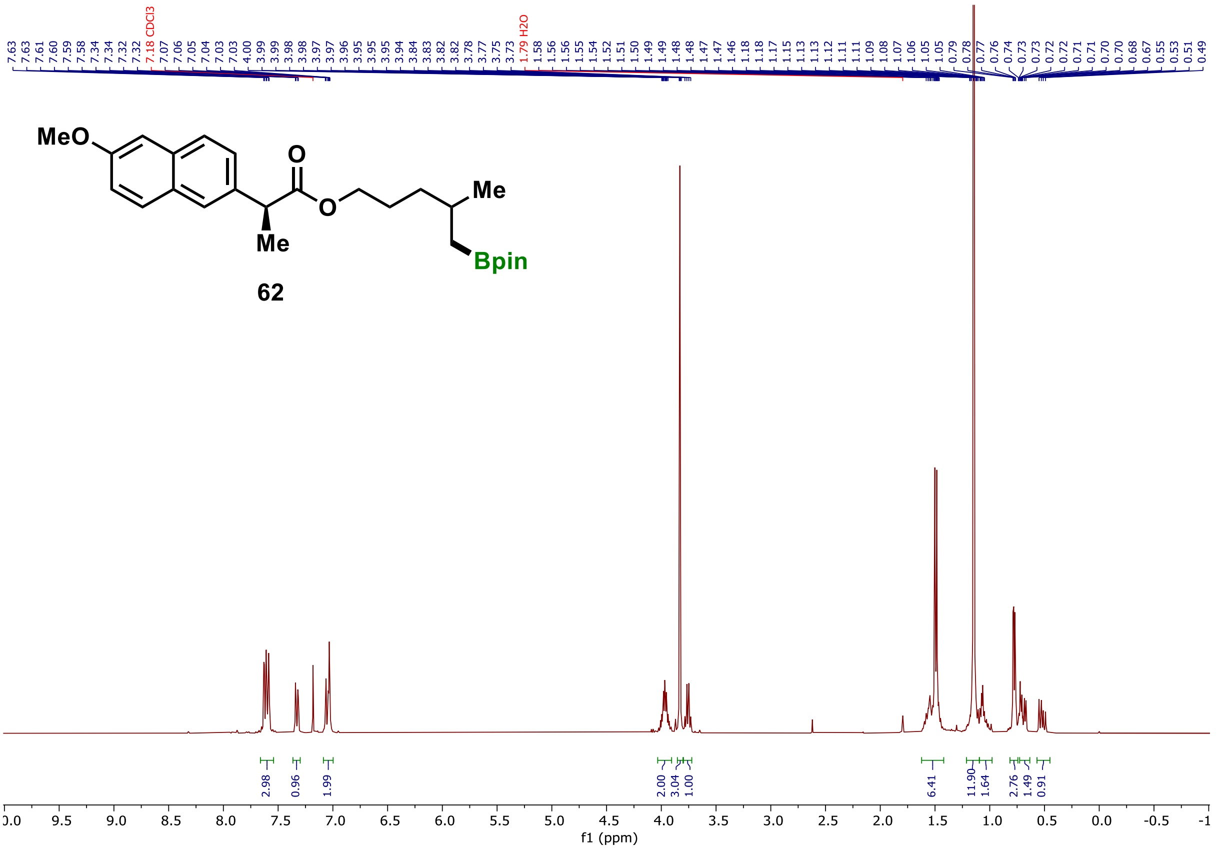

^{13}C NMR (101 MHz, $CDCl_3$) of **62**

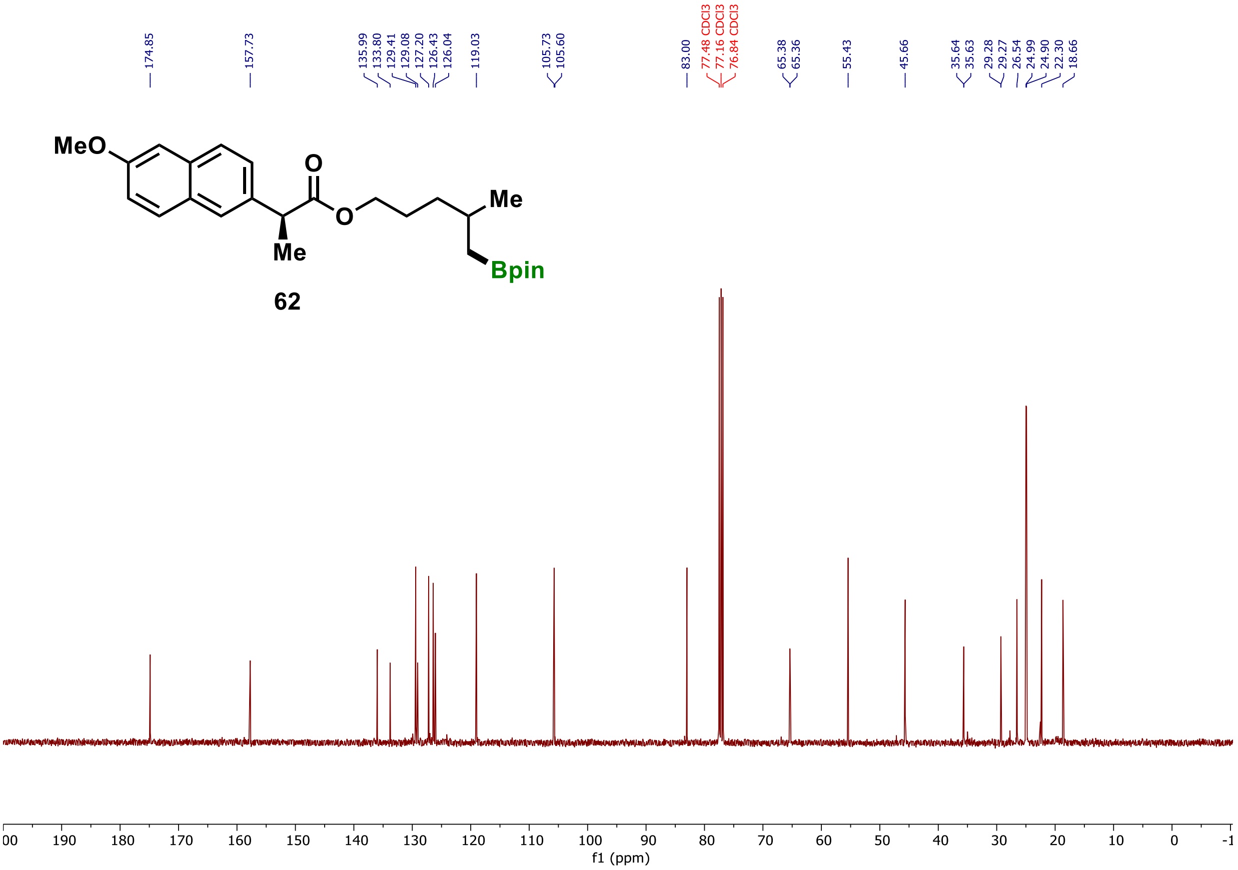

^{11}B NMR (128 MHz, $CDCl_3$) of **62**

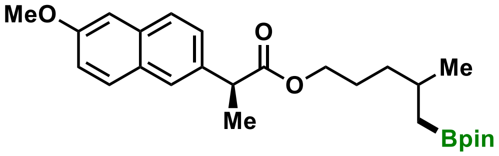

62

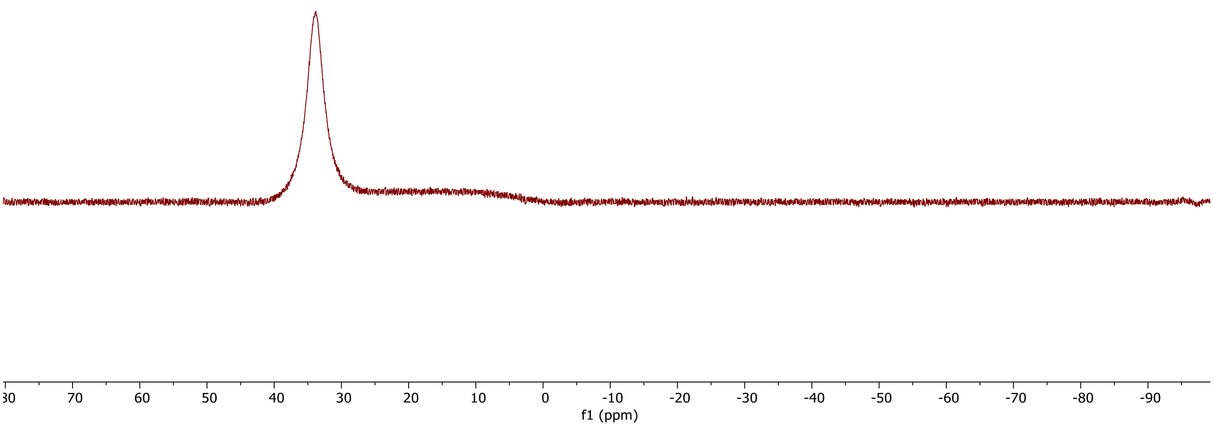

^{1}H NMR (400 MHz, CDCl$_3$) of **63-S** (*see procedure*)

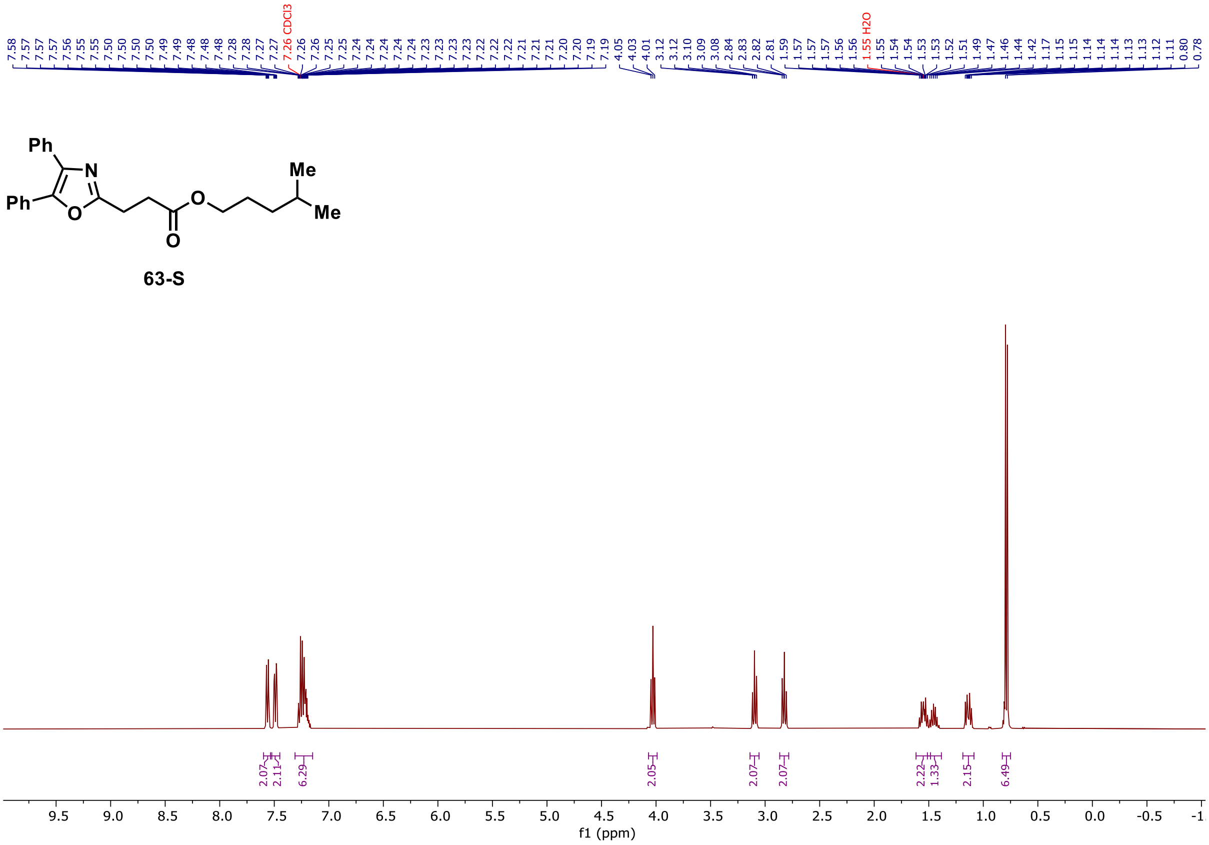

^{13}C NMR (101 MHz, CDCl$_3$) of **63-S**

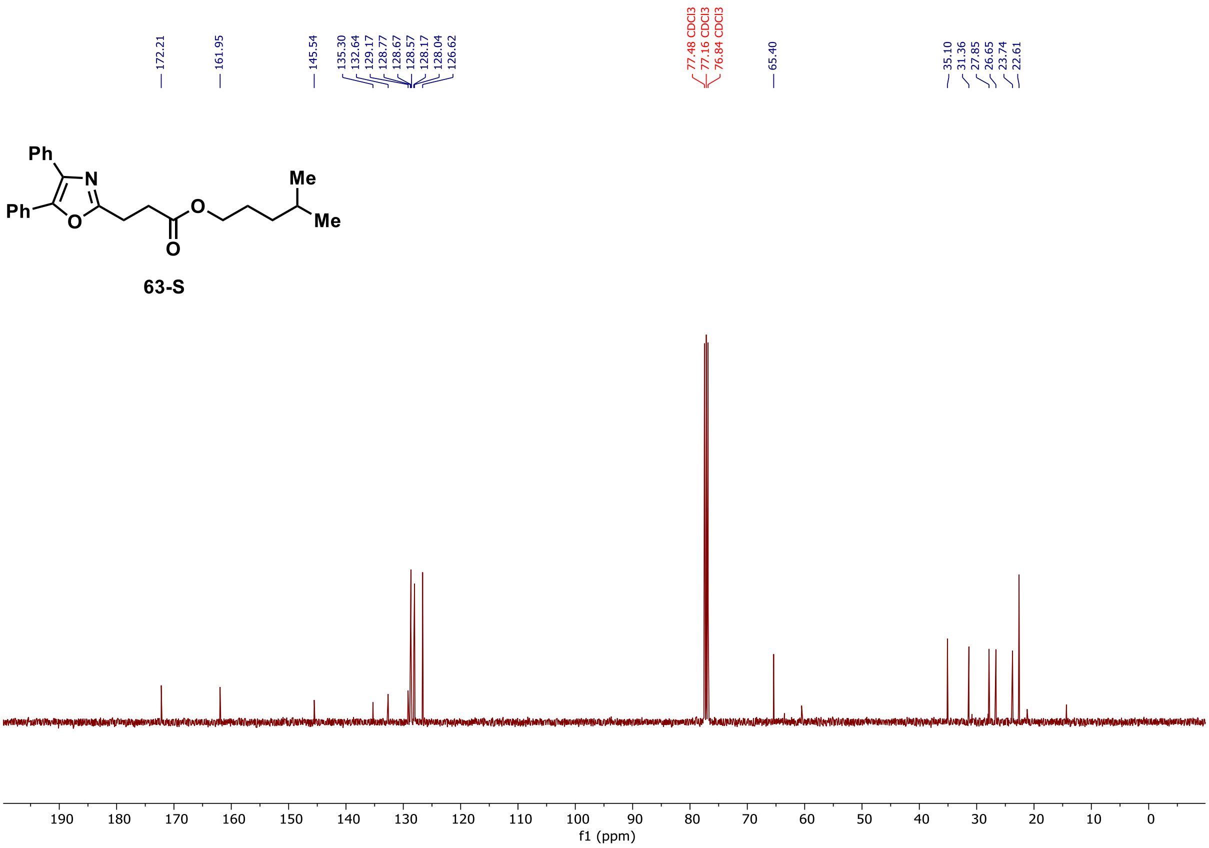

^{1}H NMR (400 MHz, $CDCl_3$) of **63** (*see procedure*)

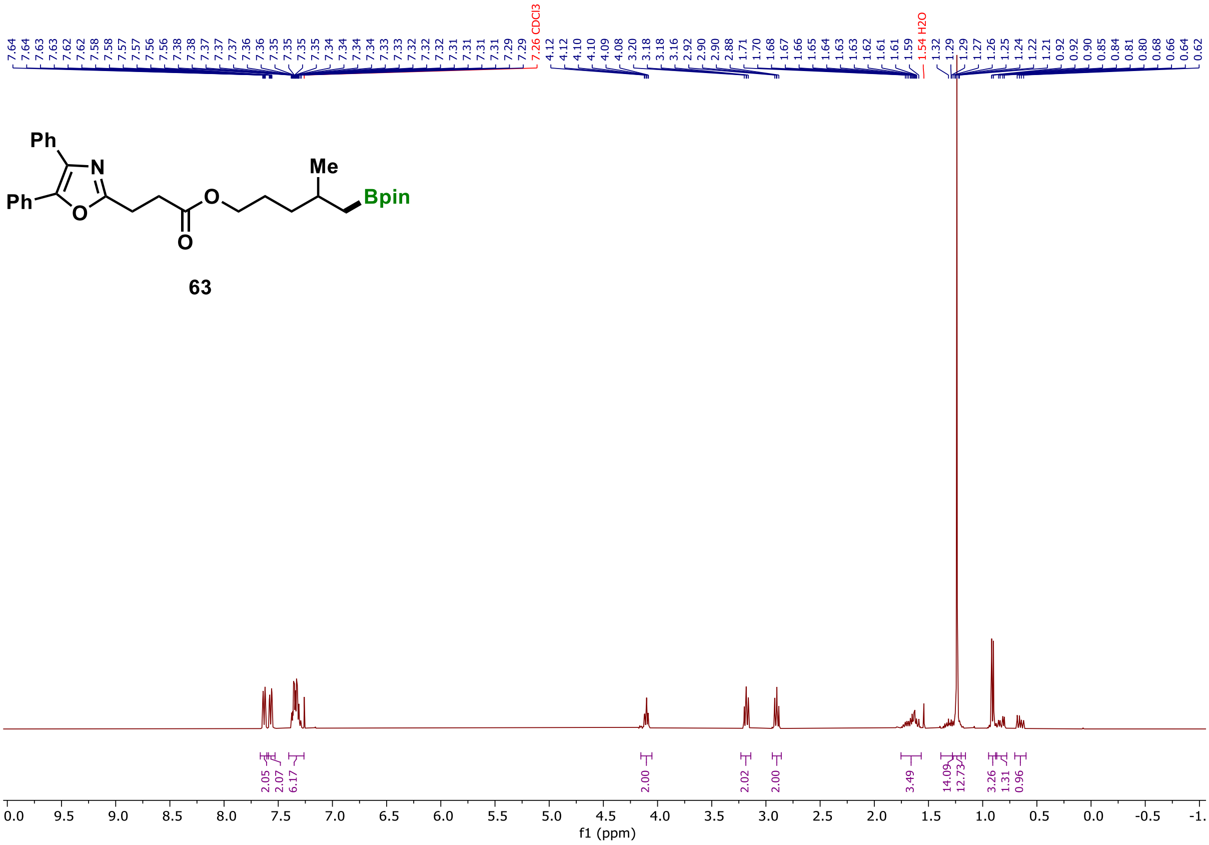

^{13}C NMR (101 MHz, $CDCl_3$) of **63**

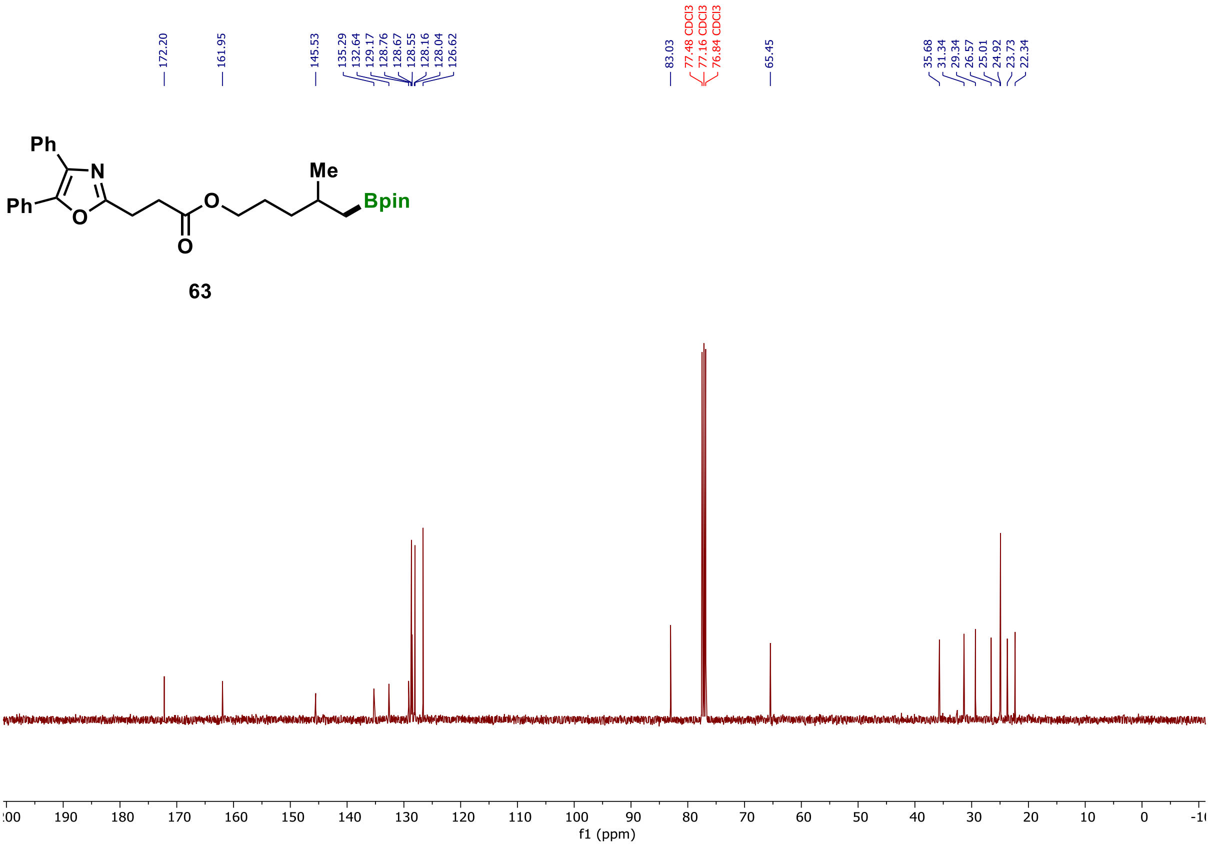

^{11}B NMR (128 MHz, $CDCl_3$) of **63**

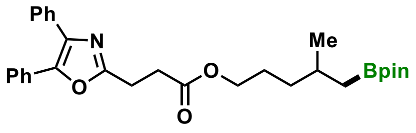

63

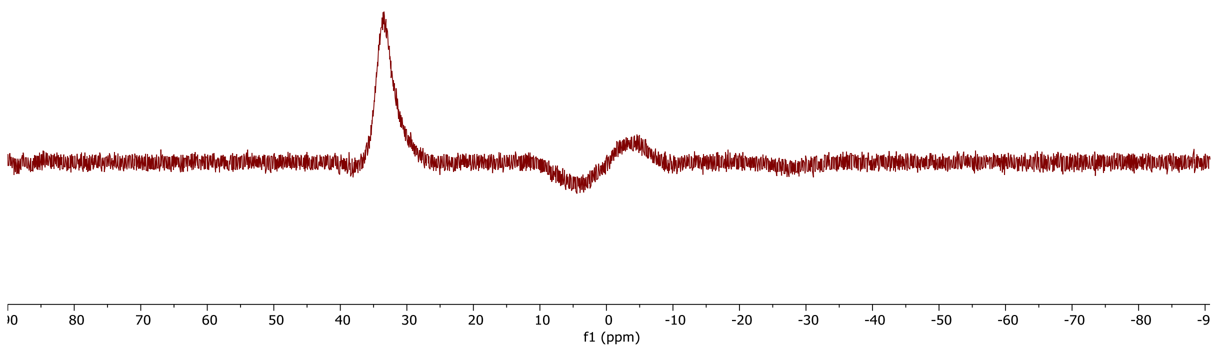

^{1}H NMR (400 MHz, $CDCl_3$) of **64-S** (*see procedure*)

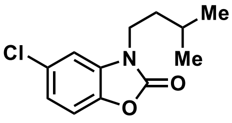

64-S

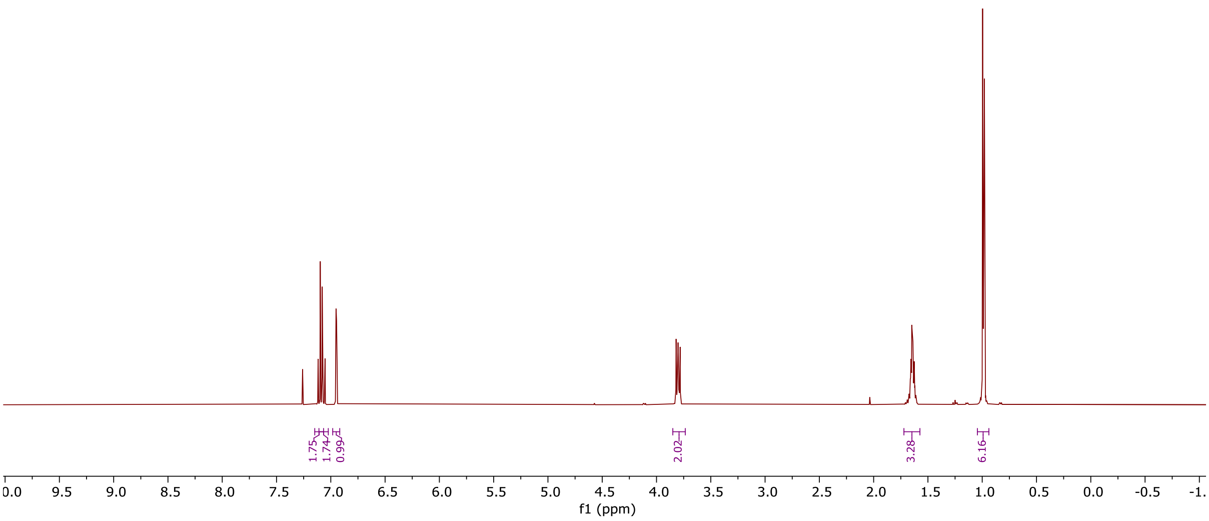

^{13}C NMR (101 MHz, $CDCl_3$) of **64-S**

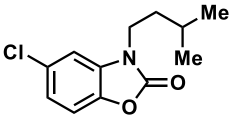

64-S

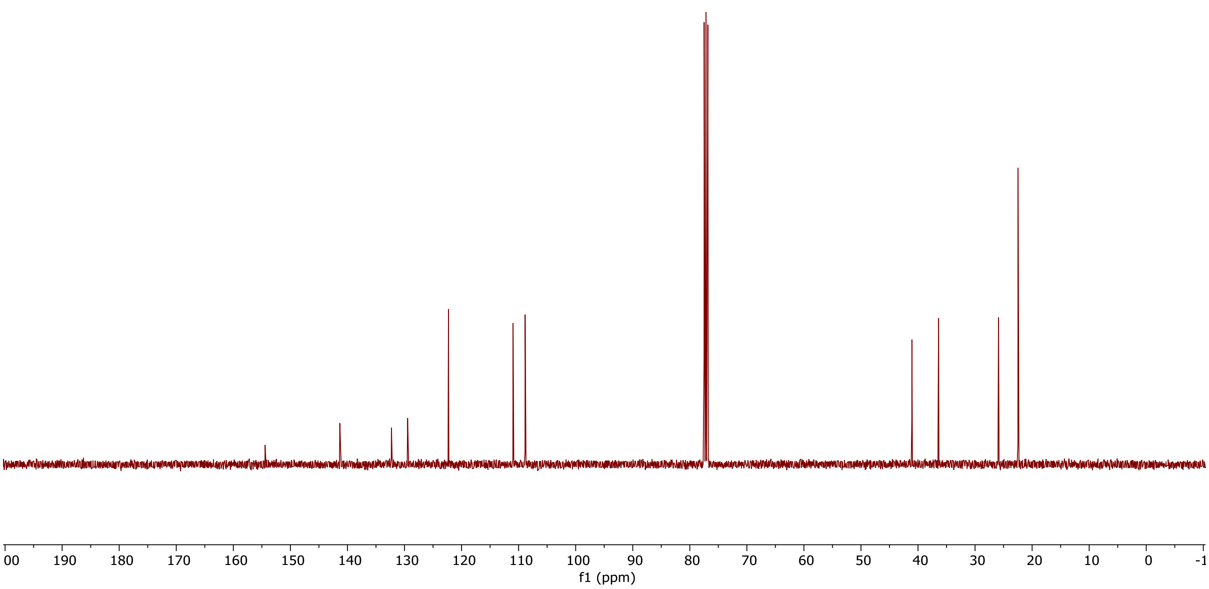

^{1}H NMR (400 MHz, $CDCl_3$) of **64** (*see procedure*)

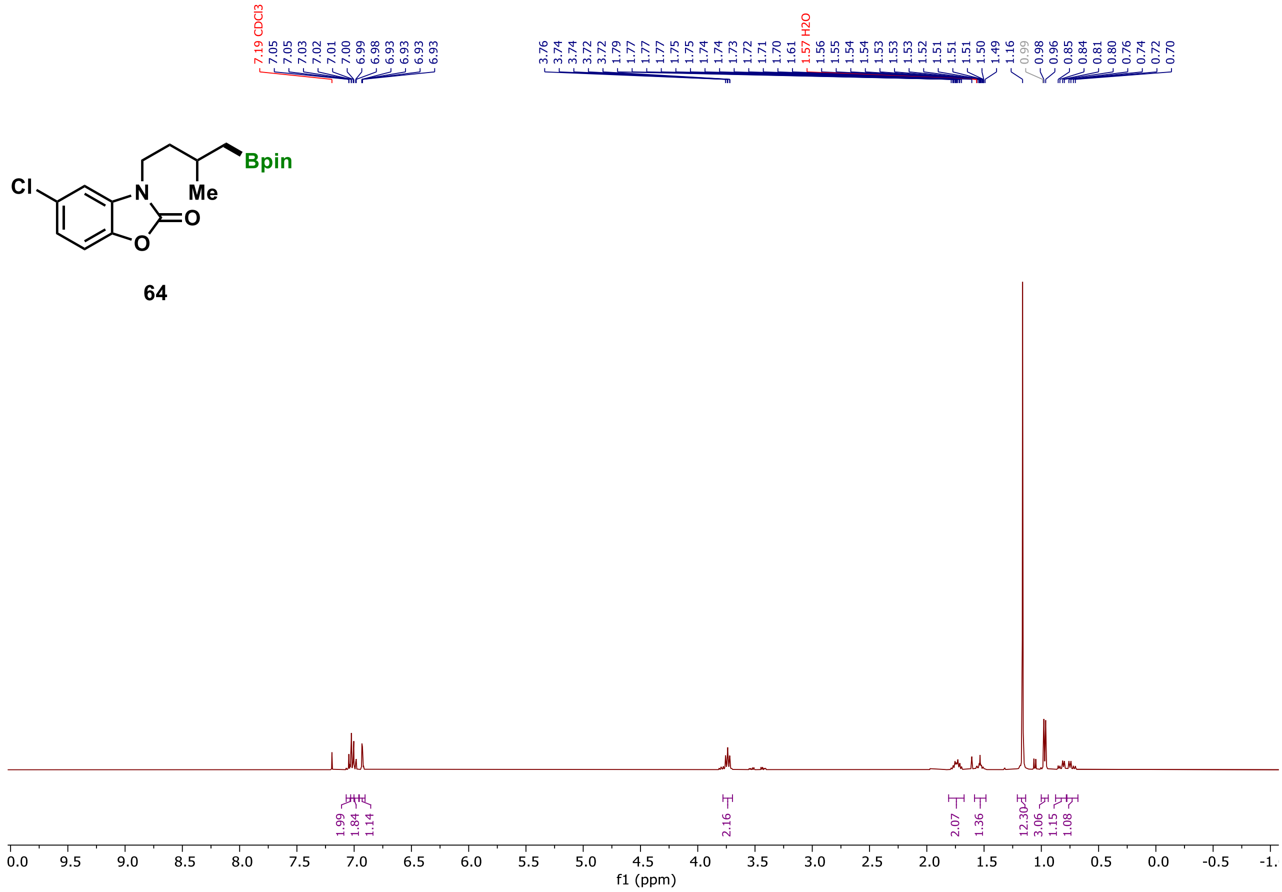

^{13}C NMR (101 MHz, $CDCl_3$) of **64**

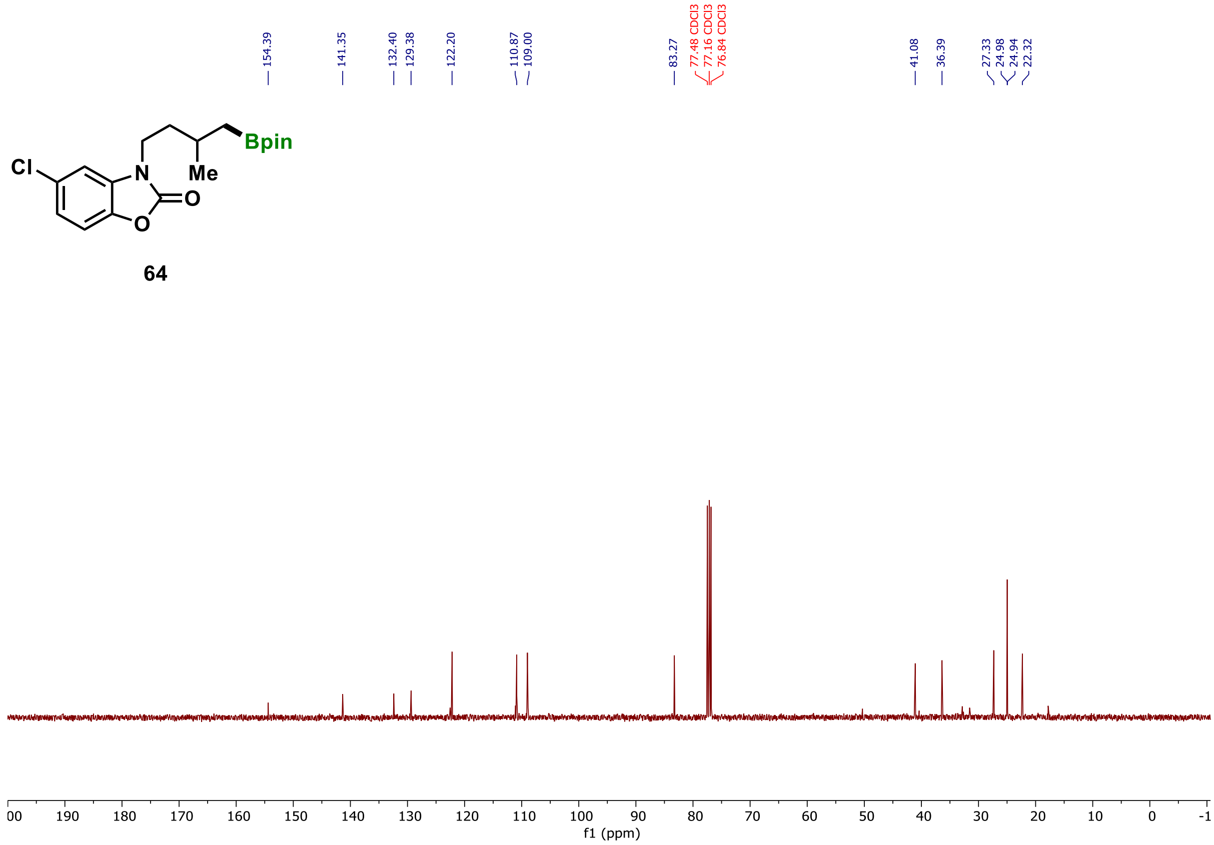

^{11}B NMR (128 MHz, $CDCl_3$) of **64**

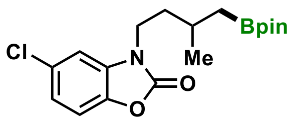

64

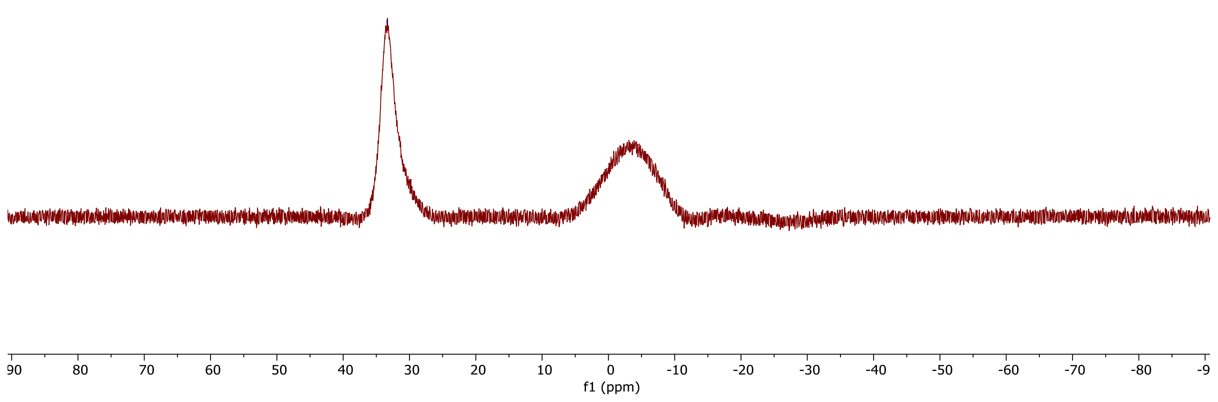

^{1}H NMR (400 MHz, $CDCl_3$) of **65-S** (*see procedure*)

A-RM1-SM

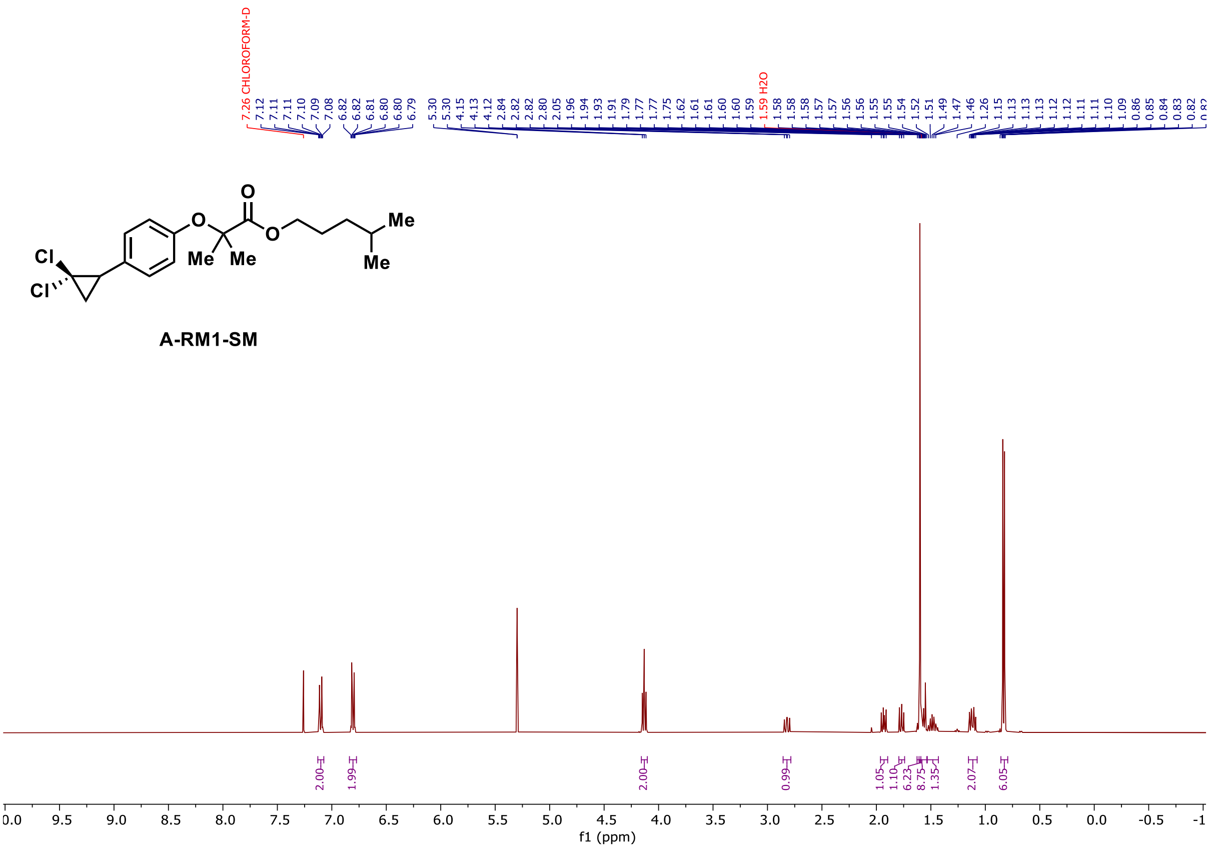

^{13}C NMR (101 MHz, $CDCl_3$) of **65-S**

A-RM1-SM

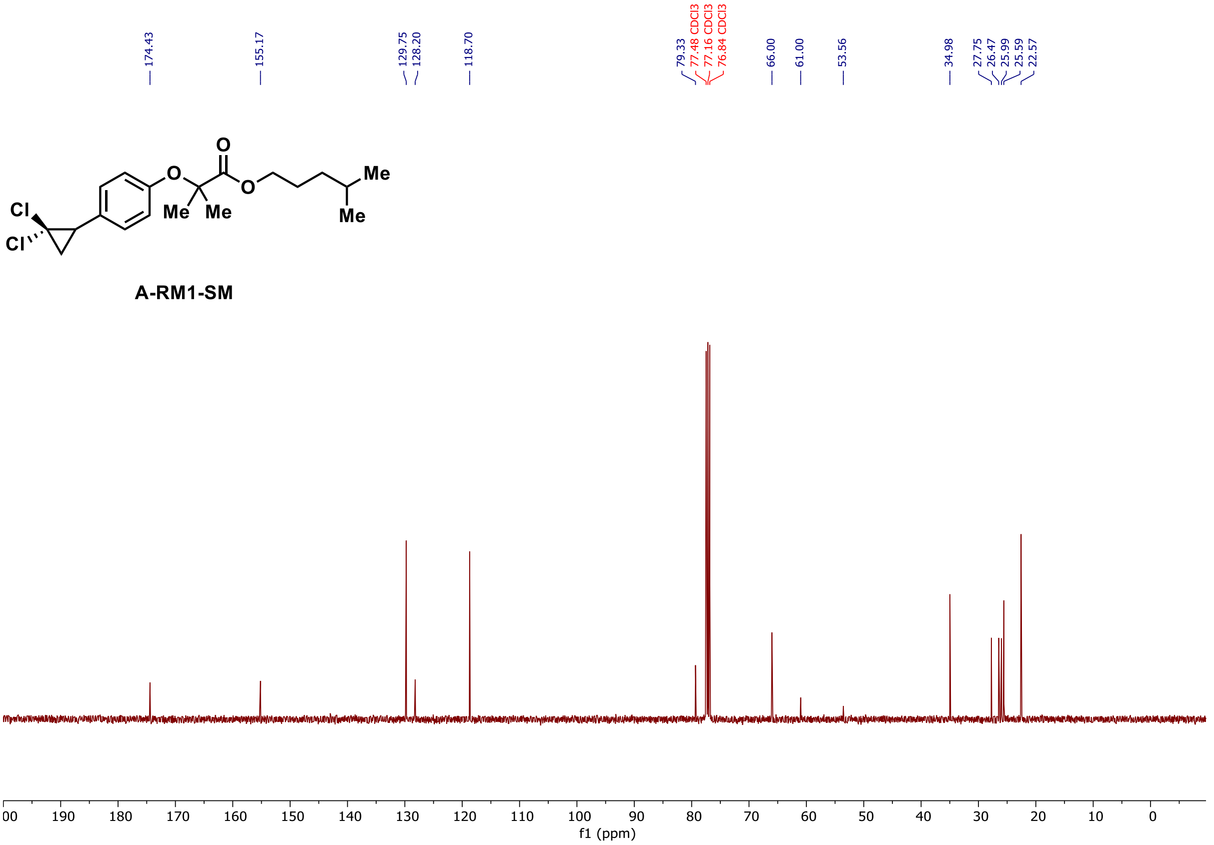

^{1}H NMR (400 MHz, $CDCl_3$) of **65** (*see procedure*)

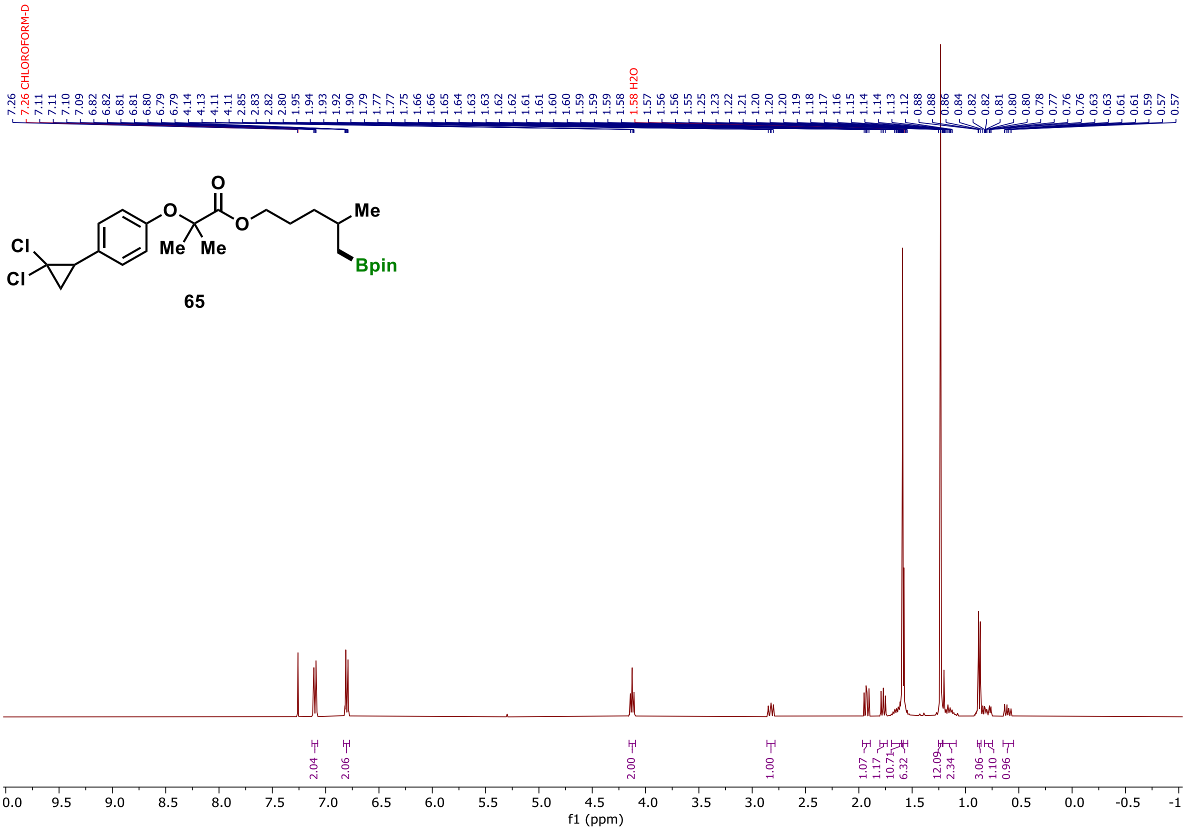

^{13}C NMR (101 MHz, $CDCl_3$) of **65**

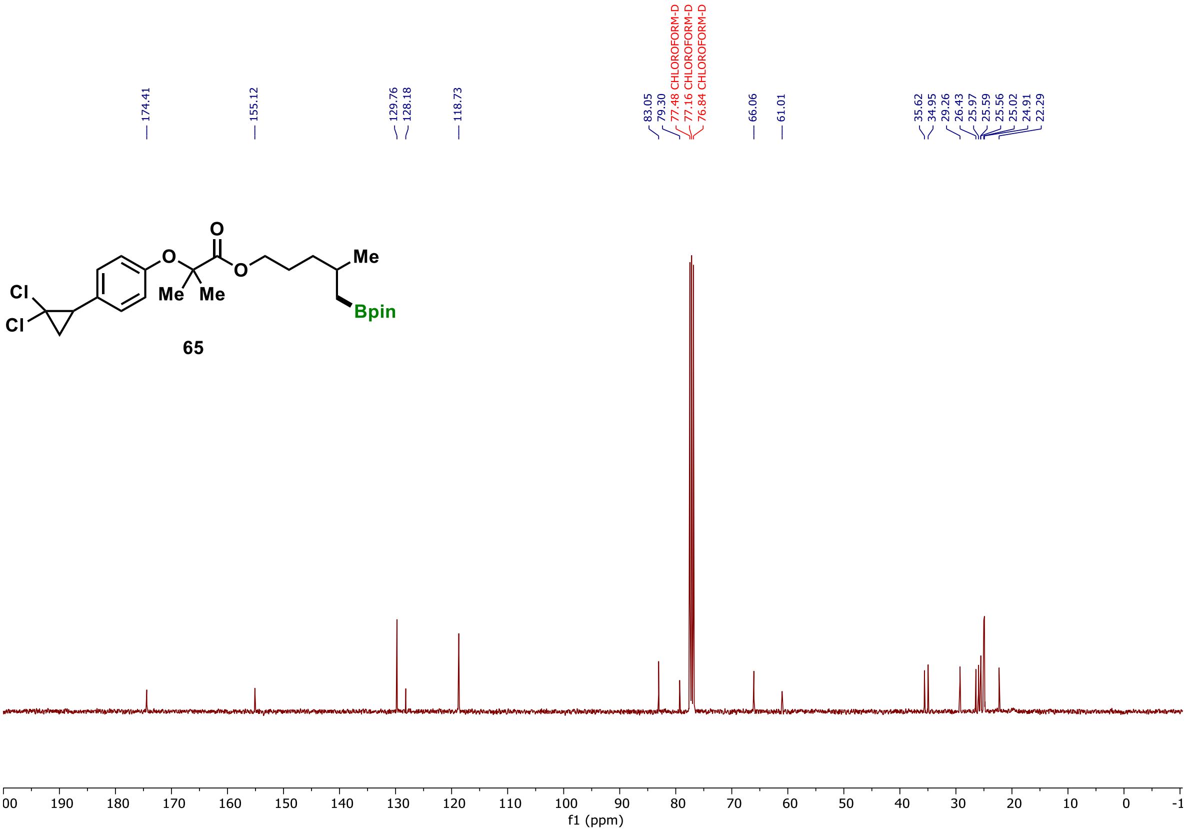

^{11}B NMR (128 MHz, $CDCl_3$) of **65**

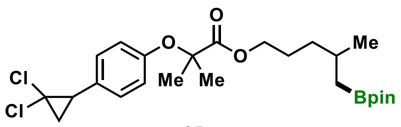

65

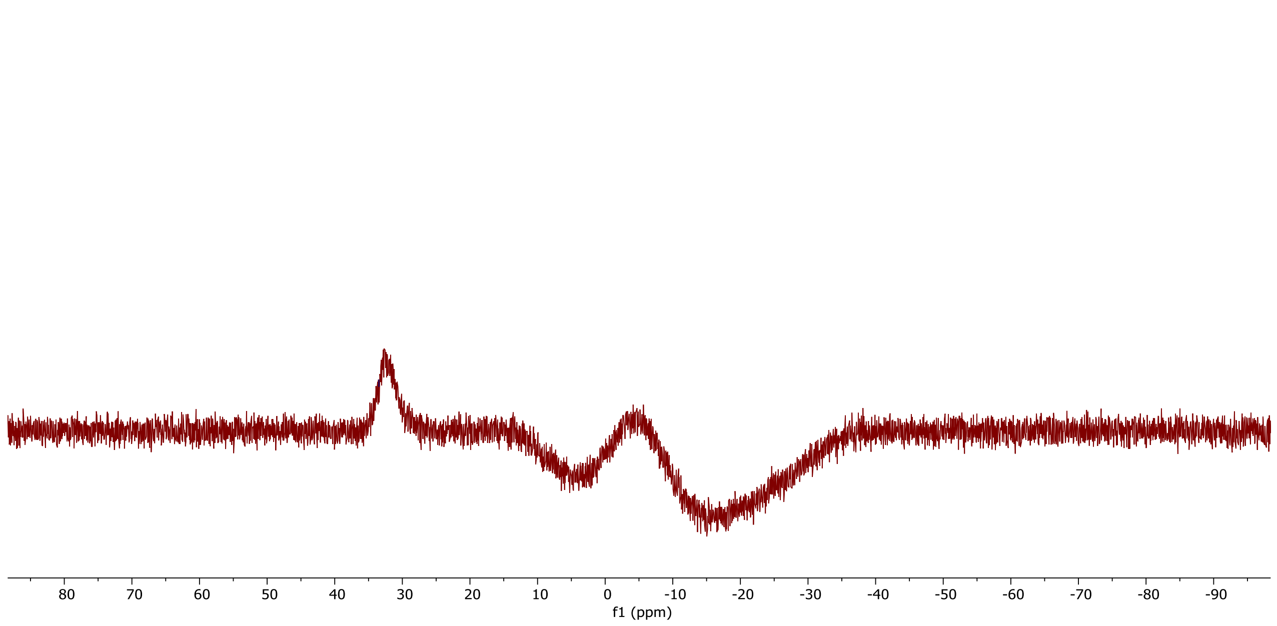

^{1}H NMR (400 MHz, CDCl$_3$) of **66** (***see procedure***)

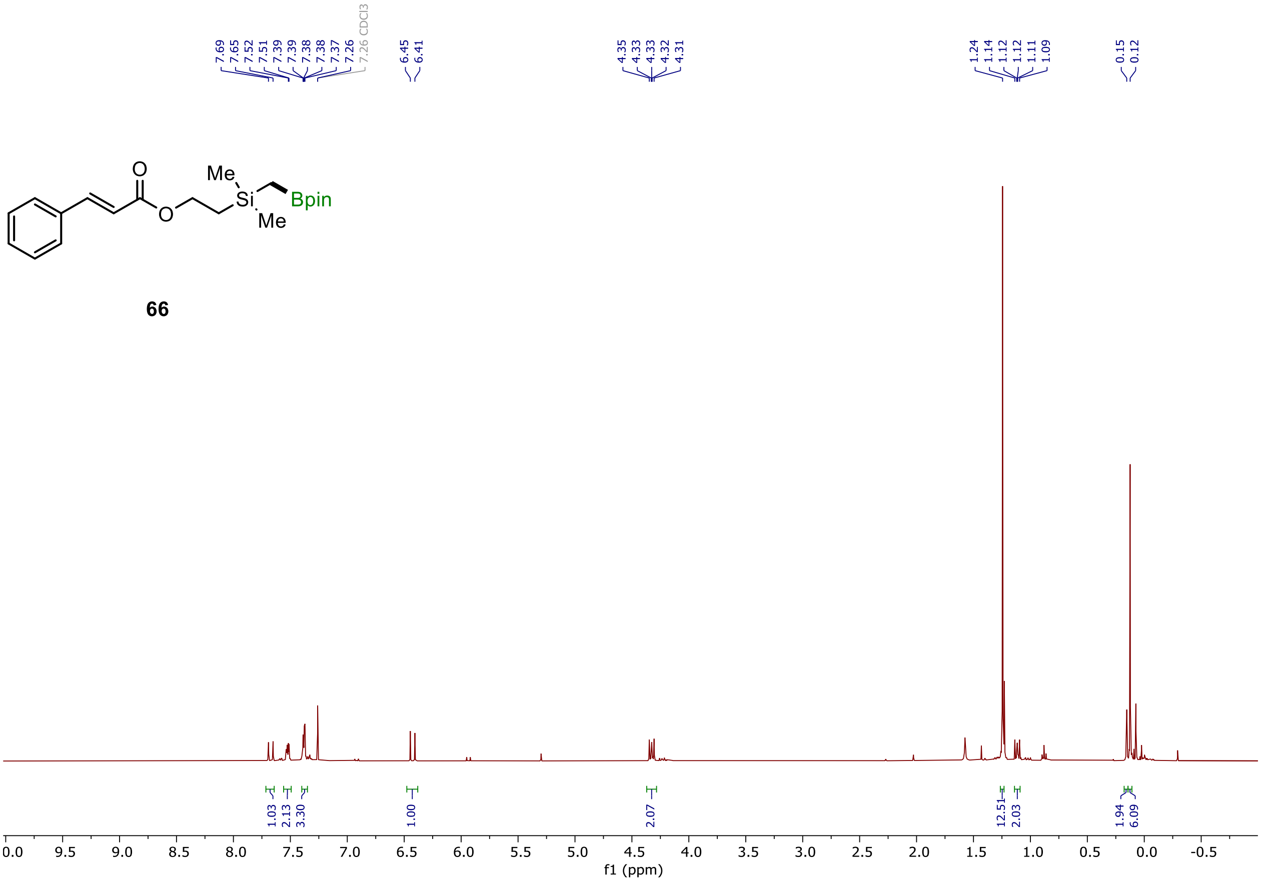

^{13}C NMR (101 MHz, CDCl$_3$) of **66**

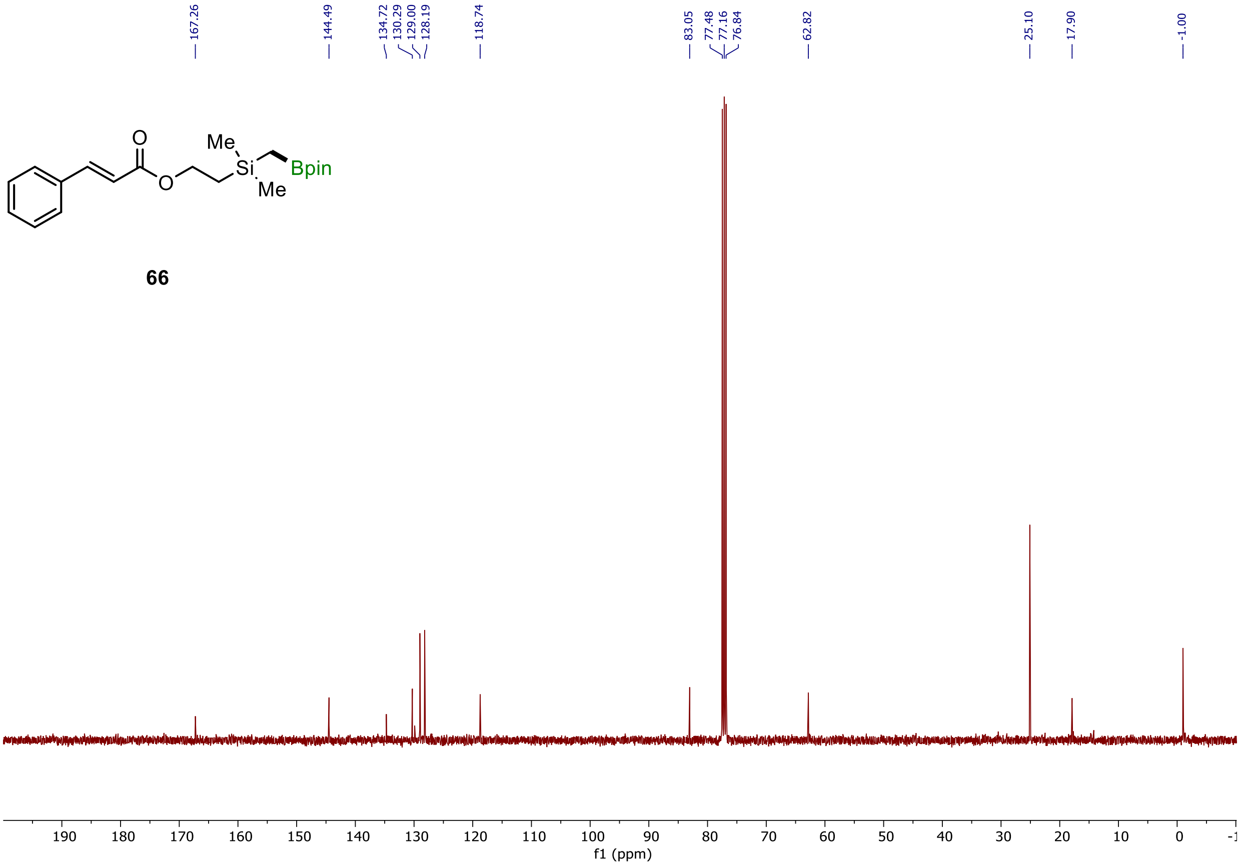

^{1}H NMR (400 MHz, $CDCl_3$) of **67-S** (*see procedure*)

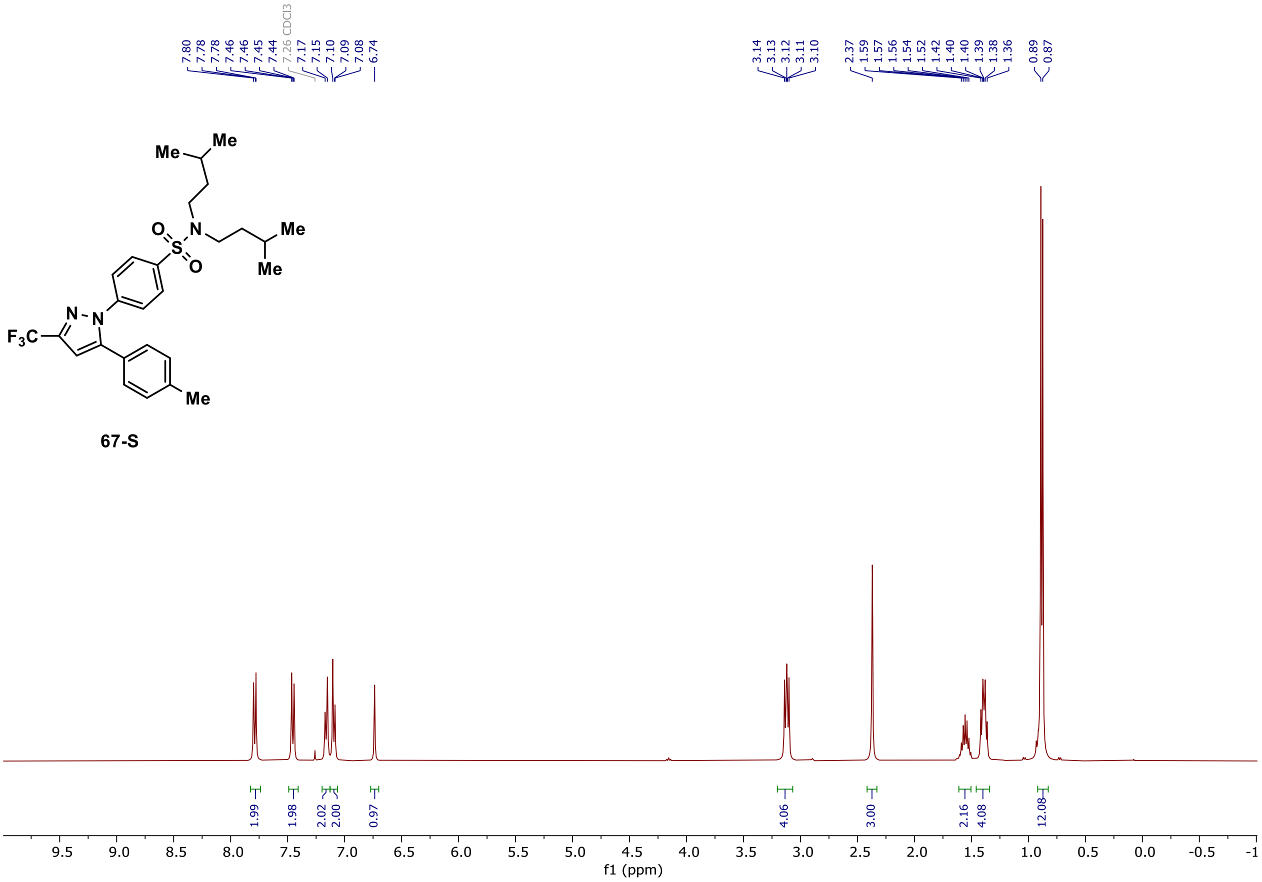

^{13}C NMR (101 MHz, $CDCl_3$) of **67-S**

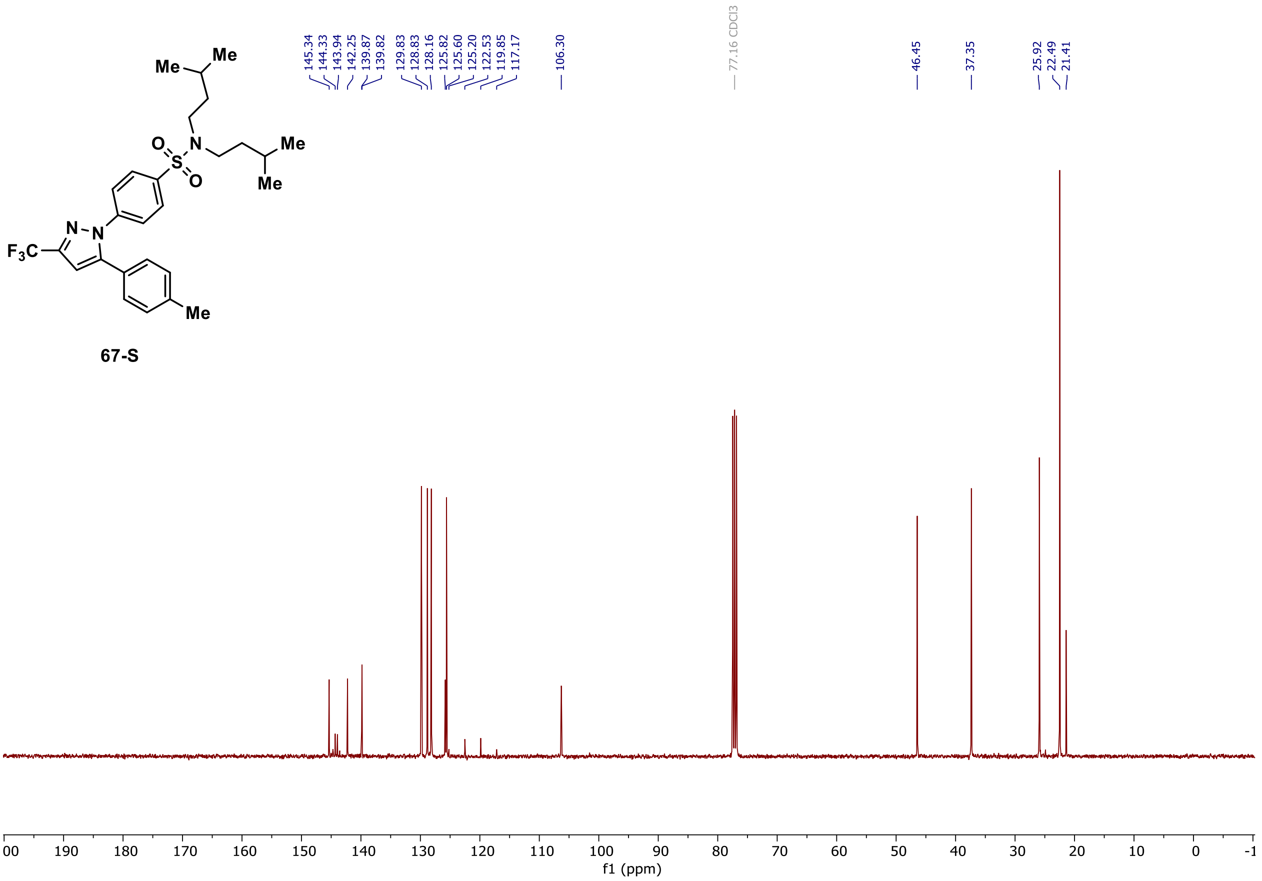

^{19}F NMR (377 MHz, $CDCl_3$) of **67-S**

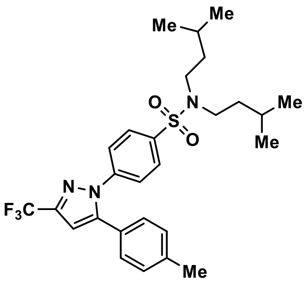

67-S

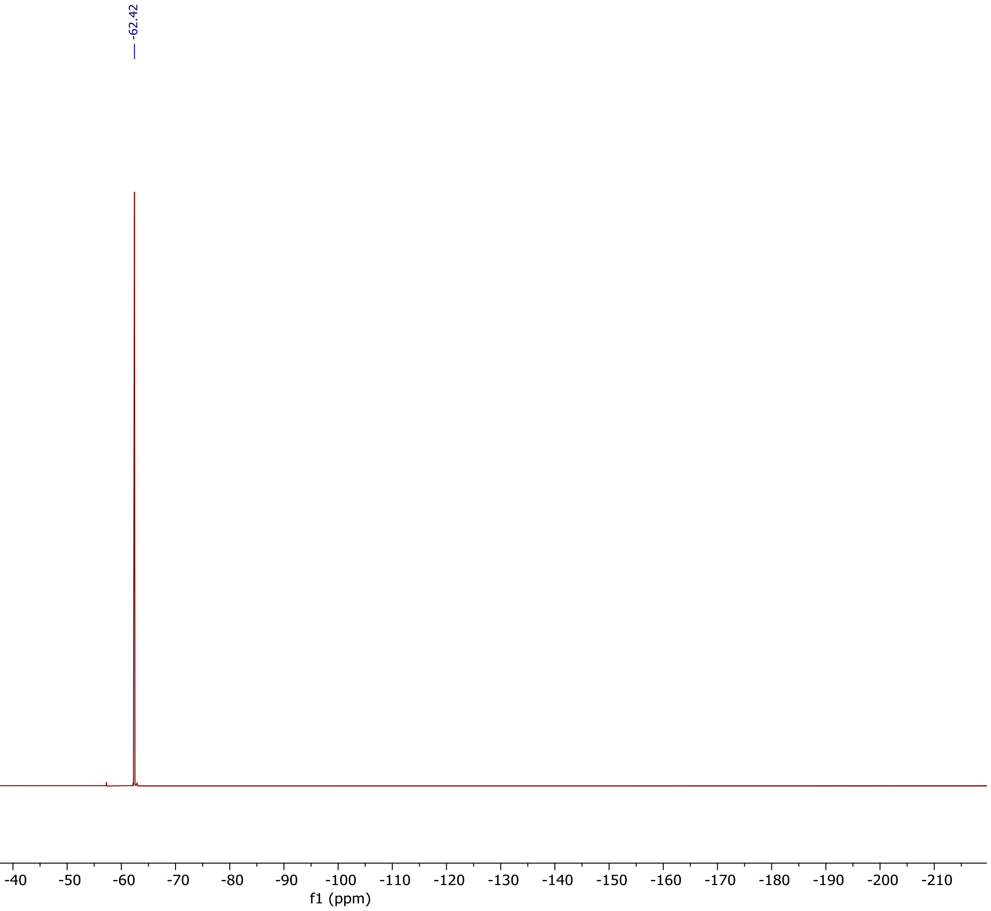

^{1}H NMR (400 MHz, $CDCl_3$) of **67** (*see procedure*)

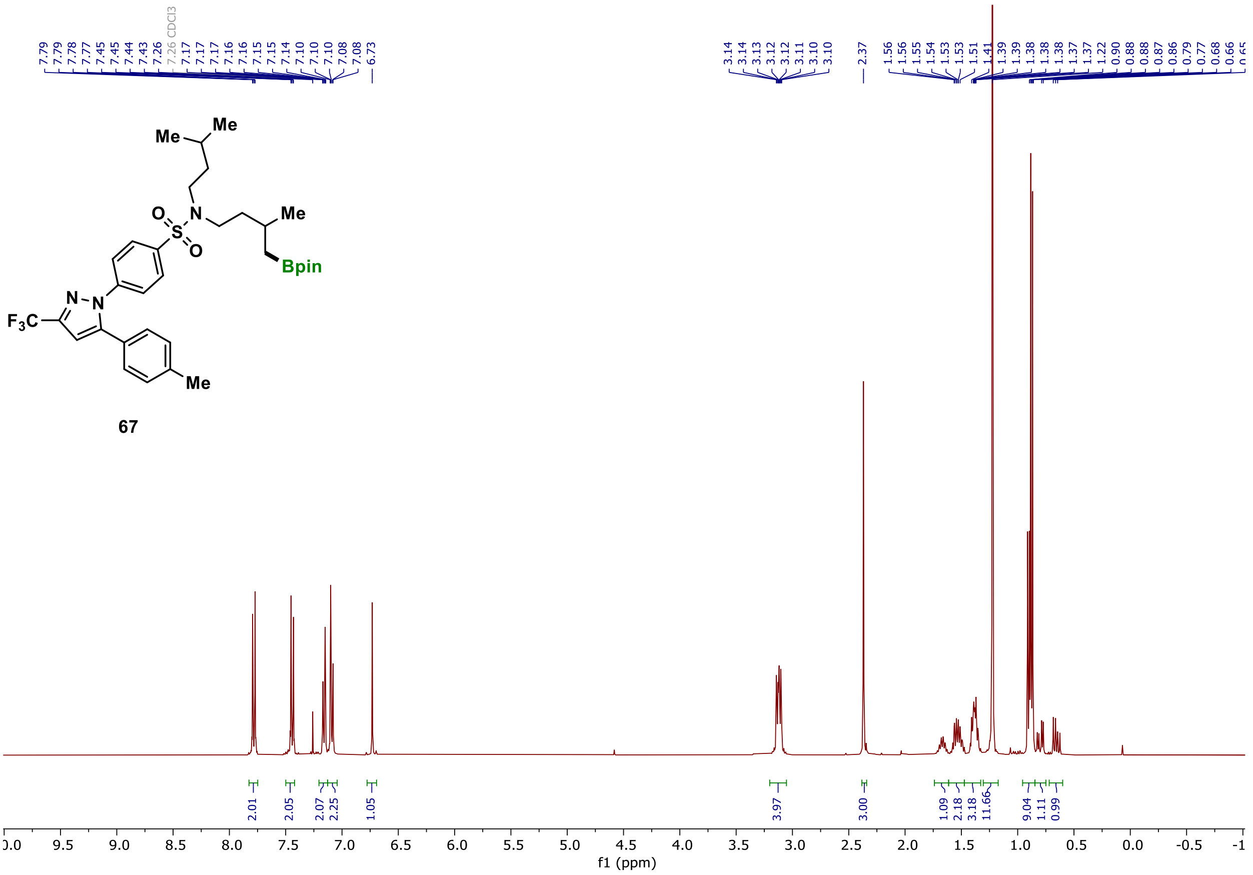

^{13}C NMR (101 MHz, $CDCl_3$) of **67**

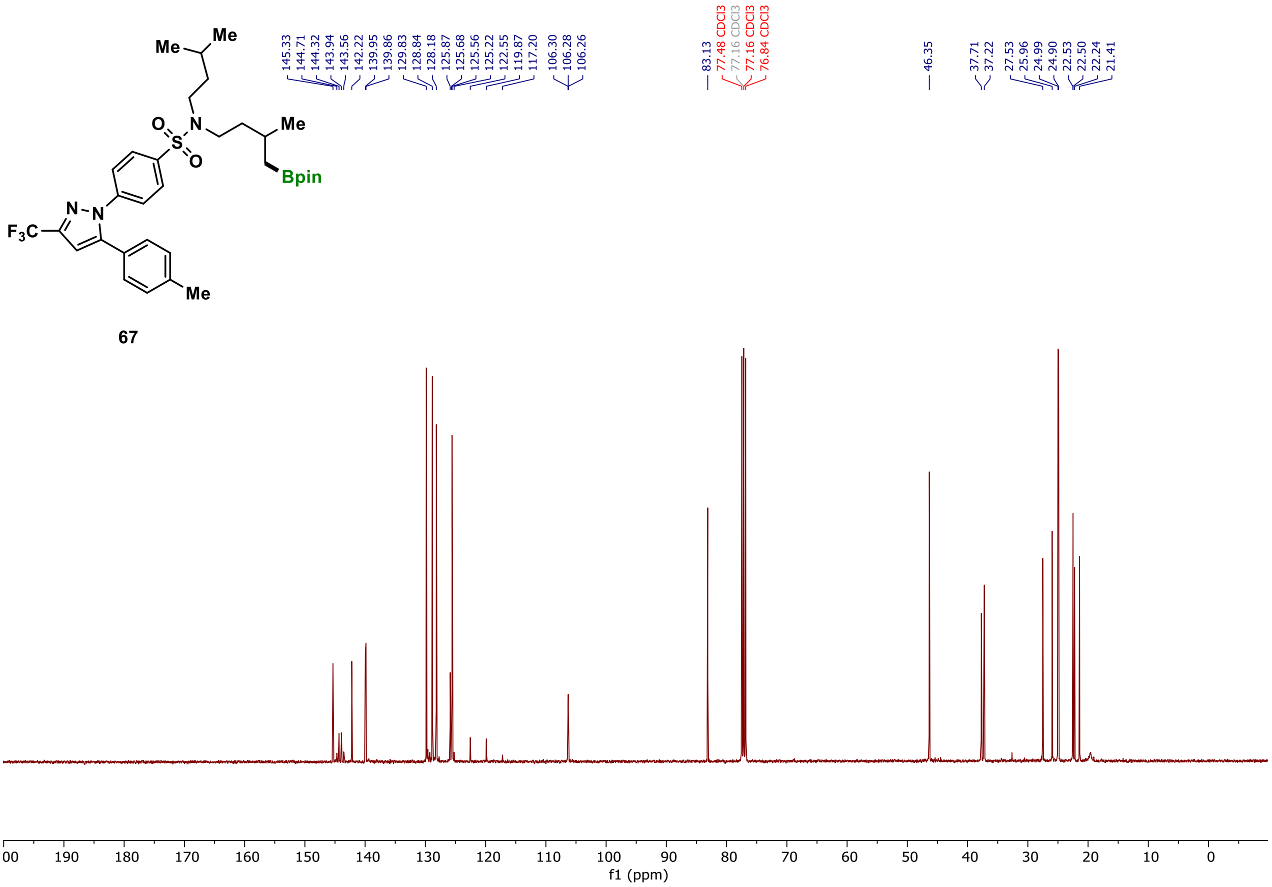

^{11}B NMR (128 MHz, $CDCl_3$) of **67**

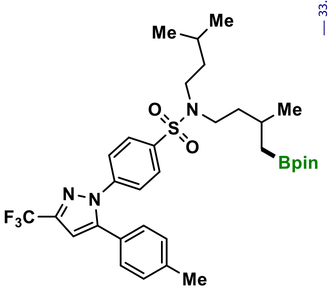

67

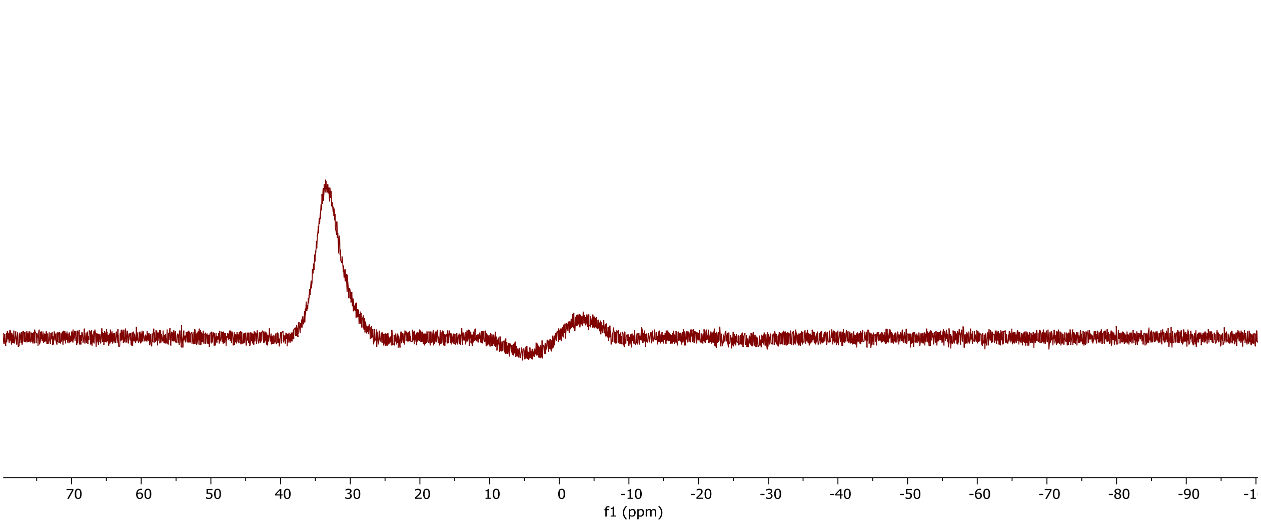

^{19}F NMR (377 MHz, $CDCl_3$) of **67**

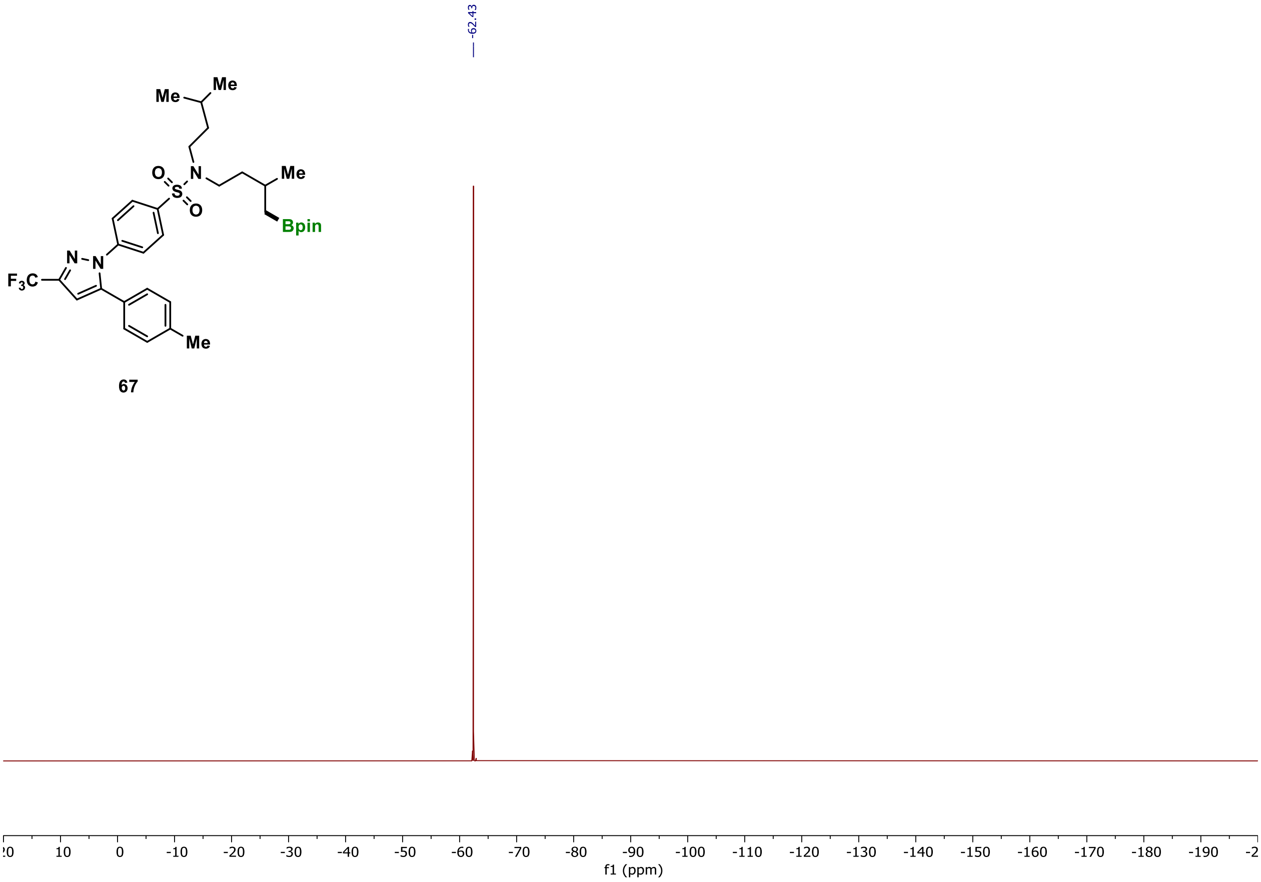

^{1}H NMR (400 MHz, CD_3CN) of **HOBcat** (*see procedure*)

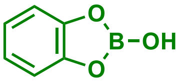

HOBcat

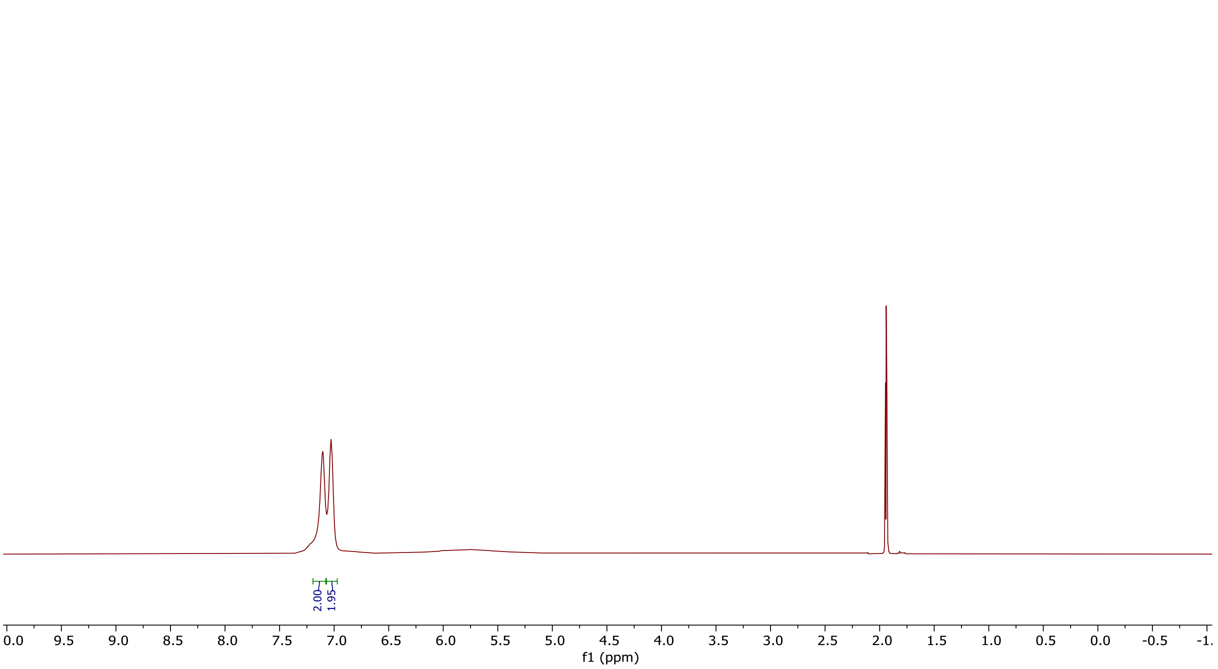

^{13}C NMR (101 MHz, CD_3CN) of **HOBcat**

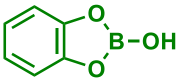

HOBcat

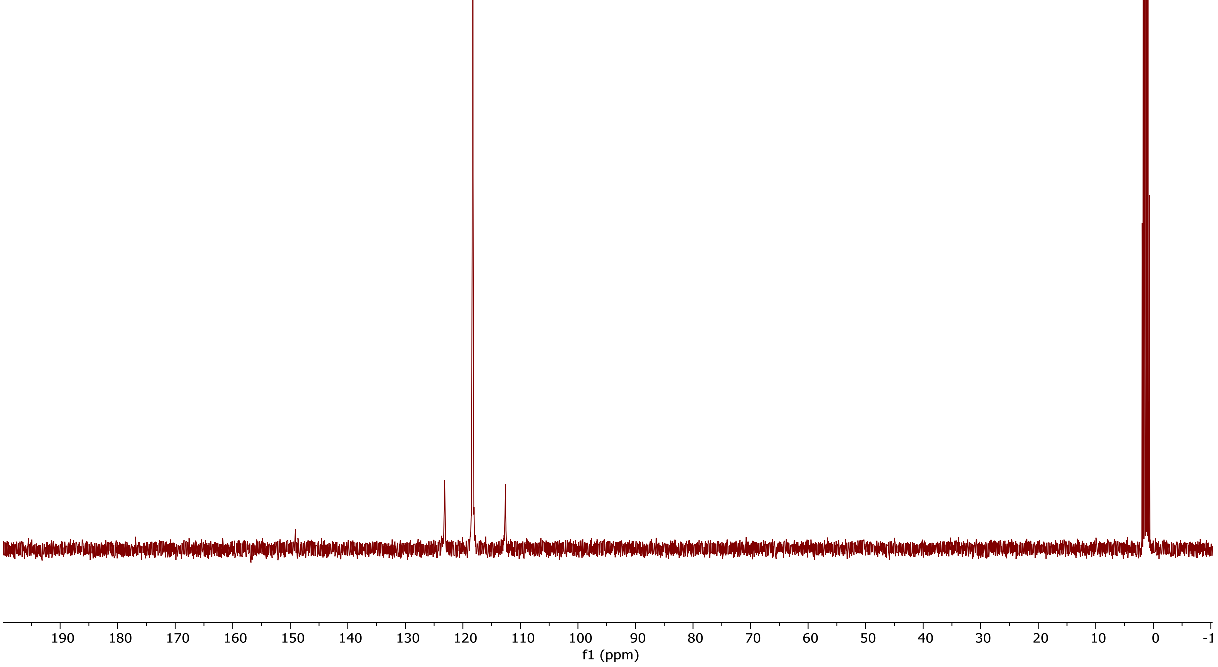

^{11}B NMR (128 MHz, CD_3CN) of **HOBcat**

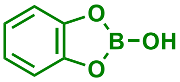

HOBcat

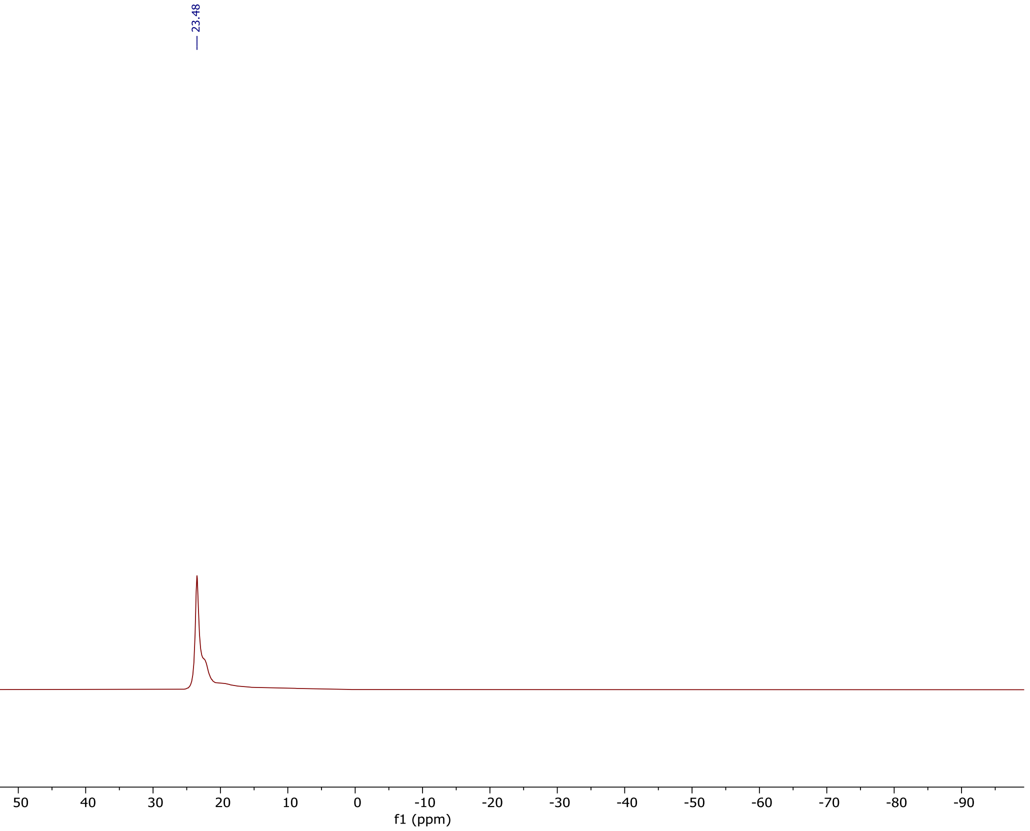

^{1}H NMR (400 MHz, CD_3CN) of **O(Bcat)$_2$** (*see procedure*)

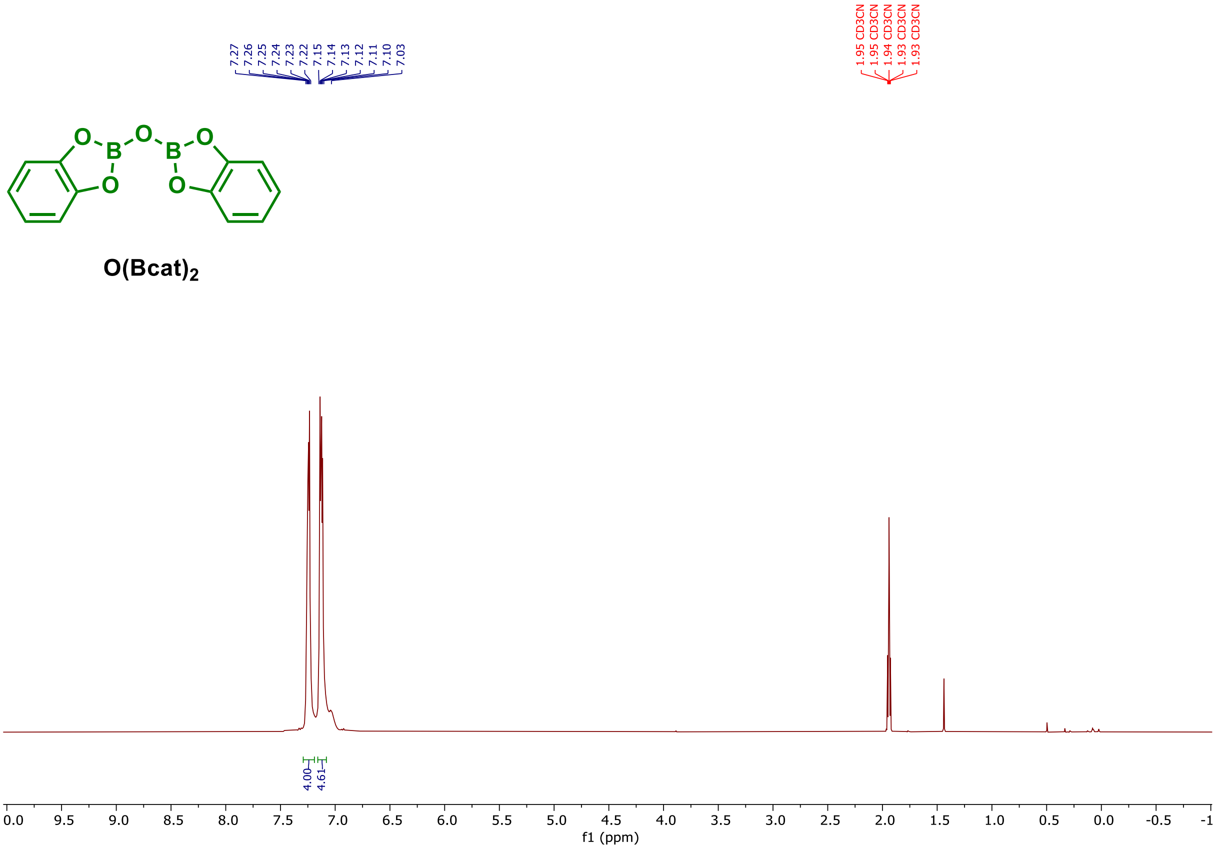

^{13}C NMR (101 MHz, CD_3CN) of **O(Bcat)$_2$**

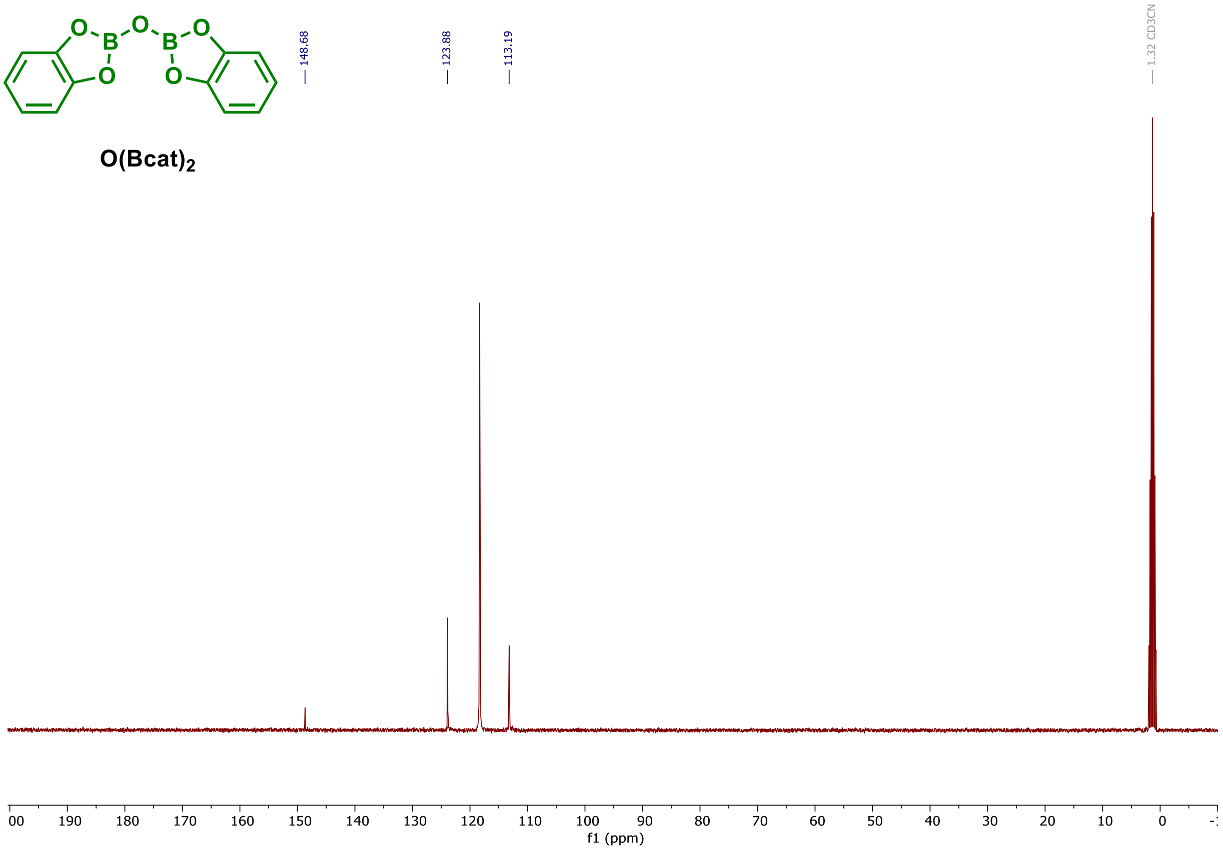

^{11}B NMR (128 MHz, CD_3CN) of **O(Bcat)$_2$**

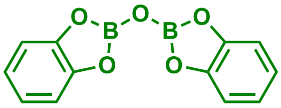

O(Bcat)$_2$

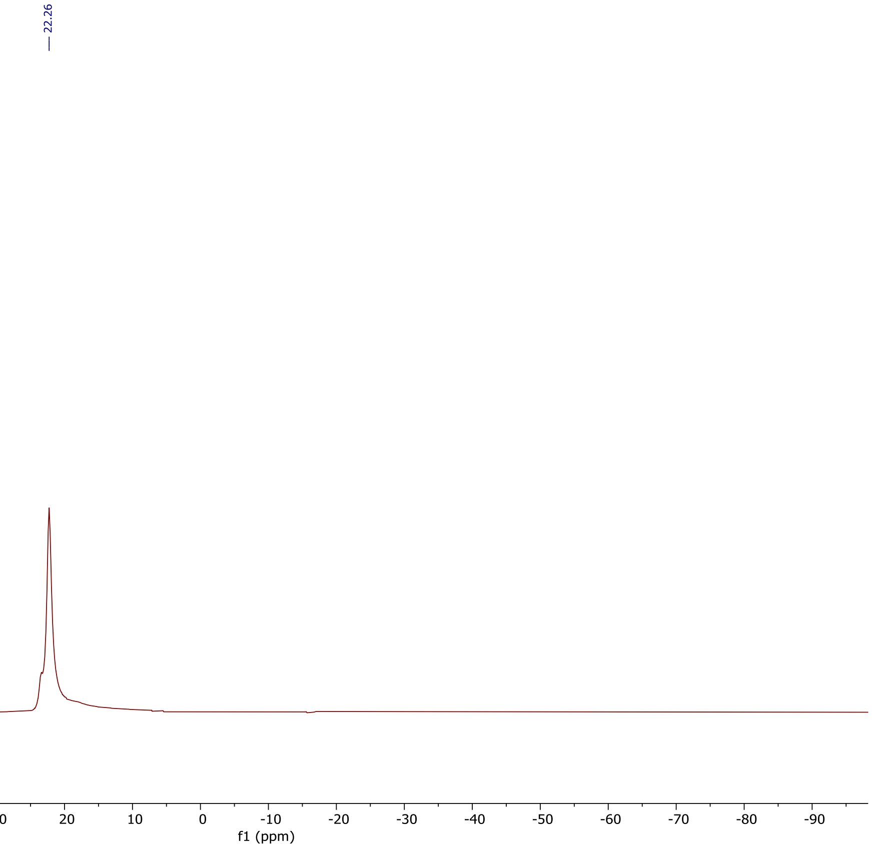

^{1}H NMR (400 MHz, $CDCl_3$) of **74** (*see procedure*)

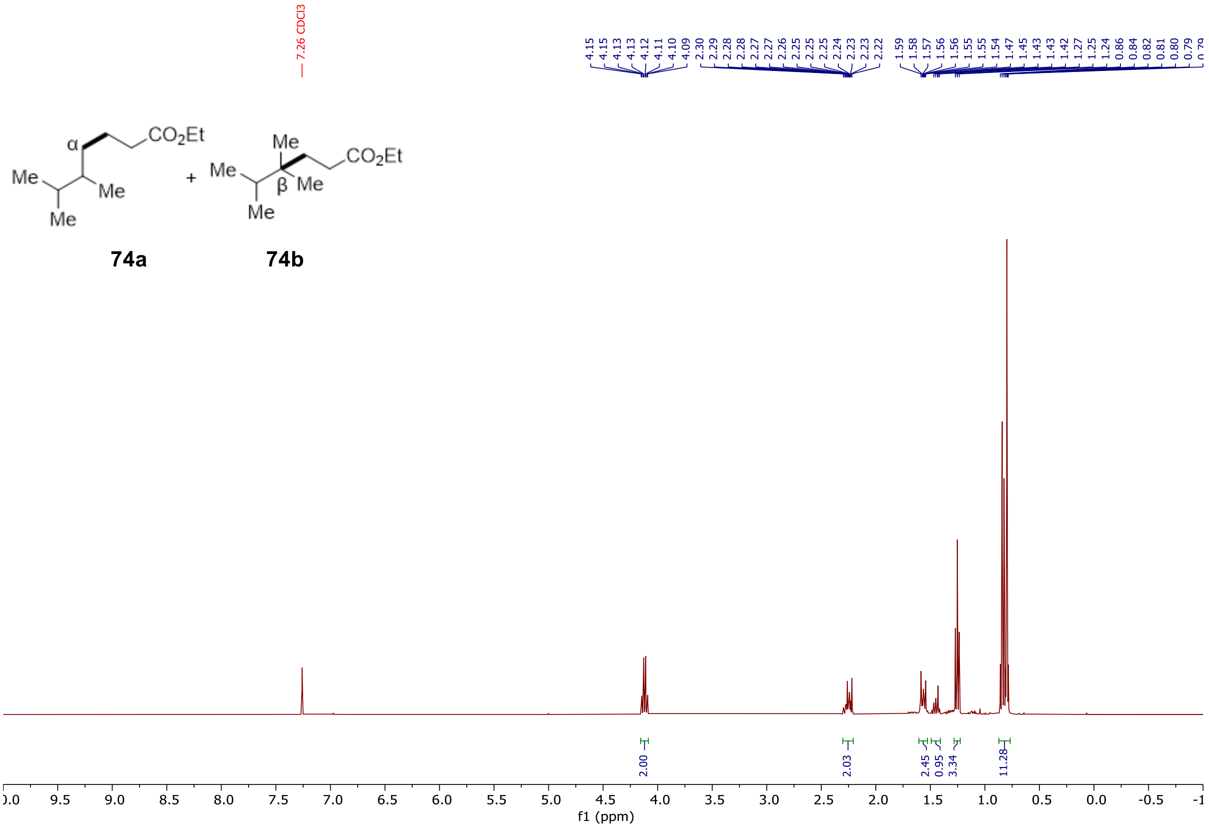

^{13}C NMR (101 MHz, $CDCl_3$) of **74**

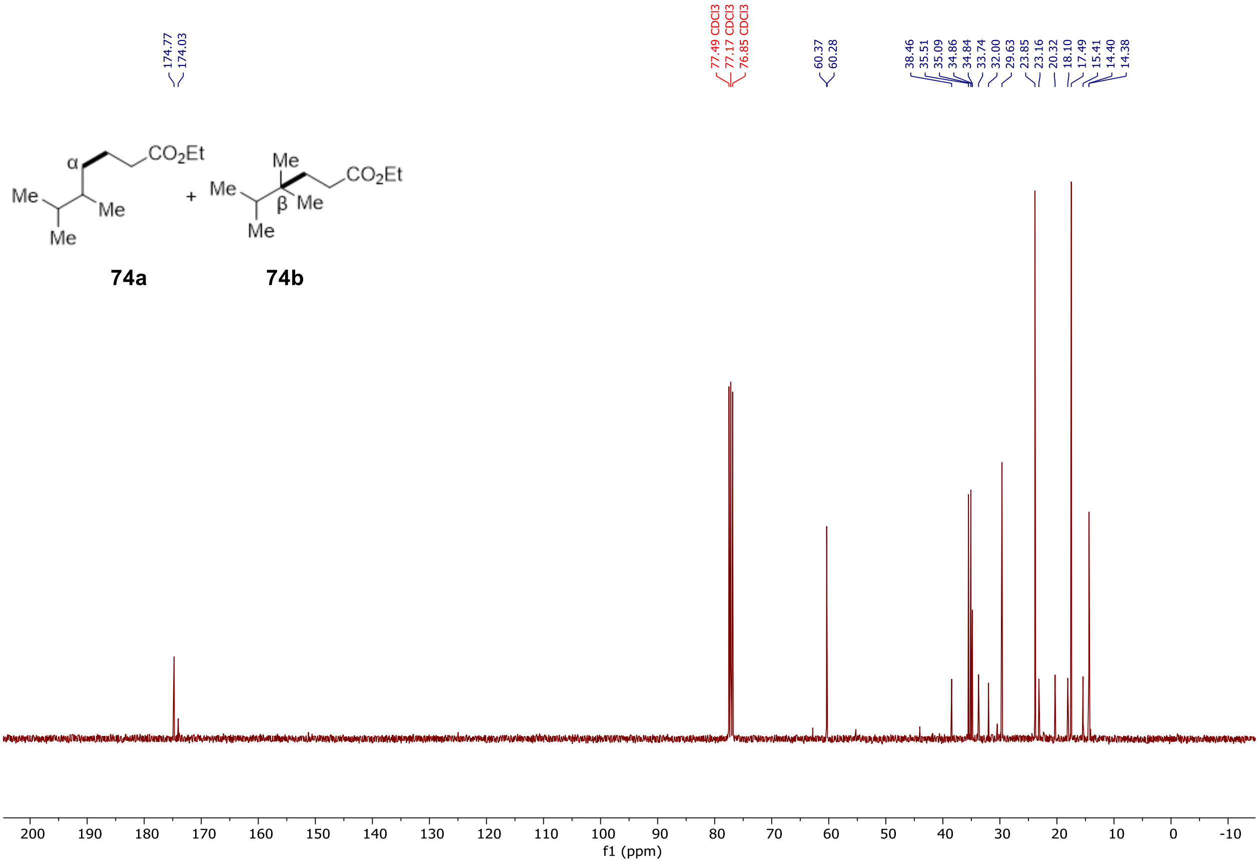

^{1}H NMR (400 MHz, $CDCl_3$) of **75a** (*see procedure*)

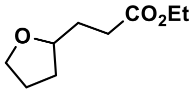

75a

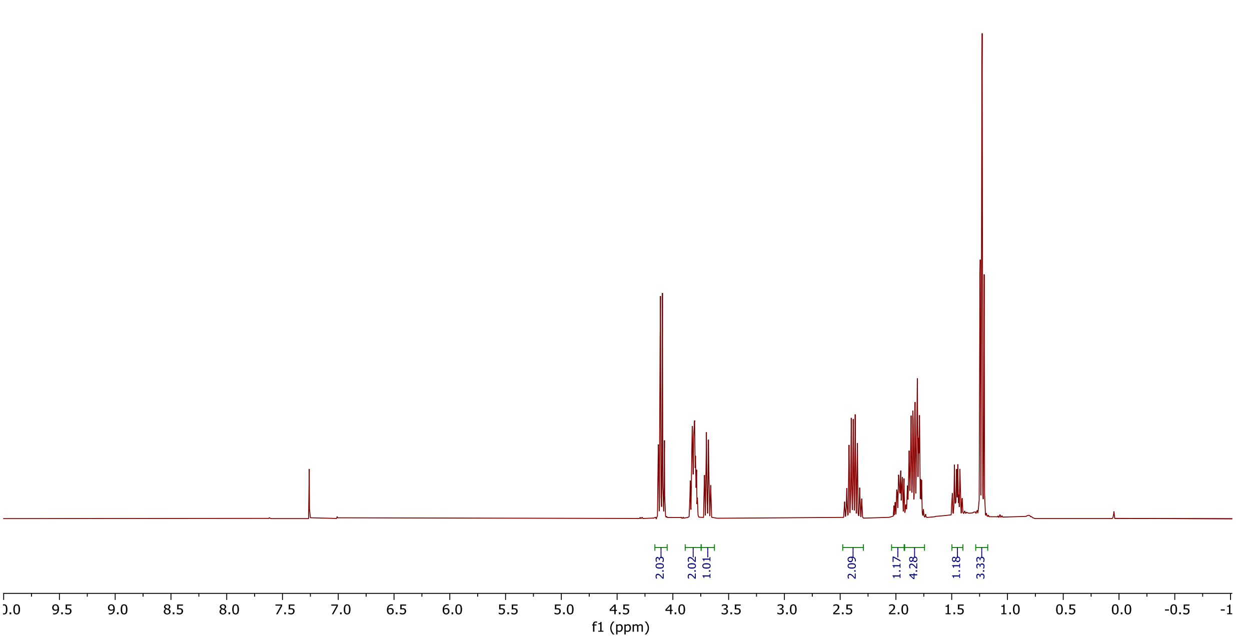

^{13}C NMR (101 MHz, $CDCl_3$) of **75a**

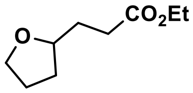

75a

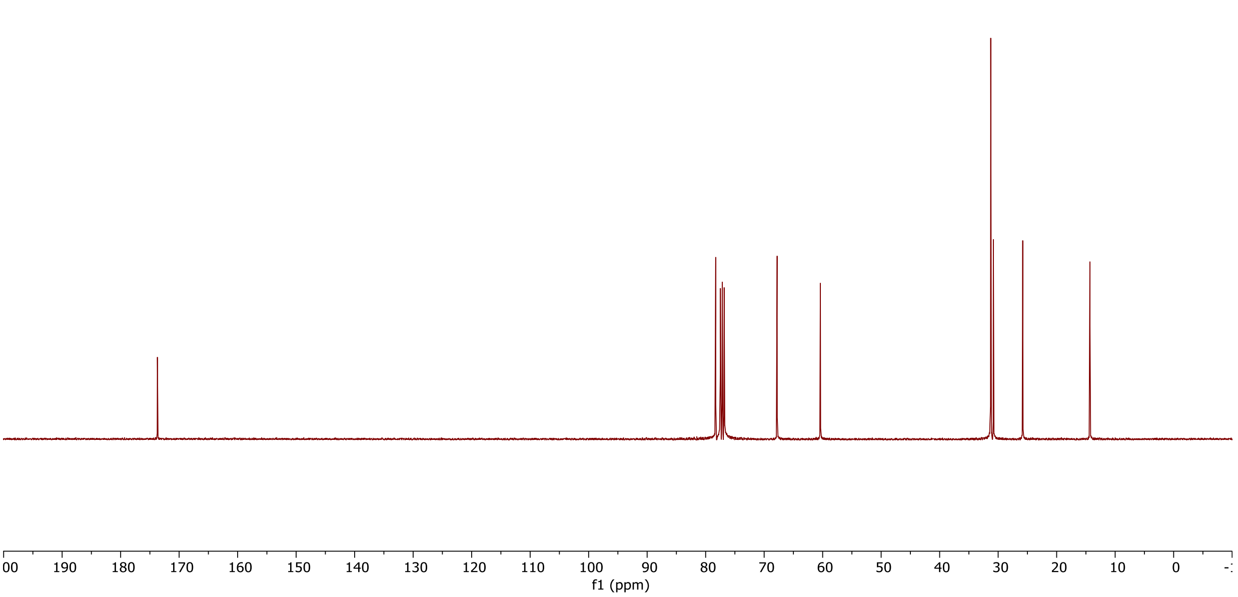

^{1}H NMR (400 MHz, CDCl$_3$) of **76** (*see procedure*)

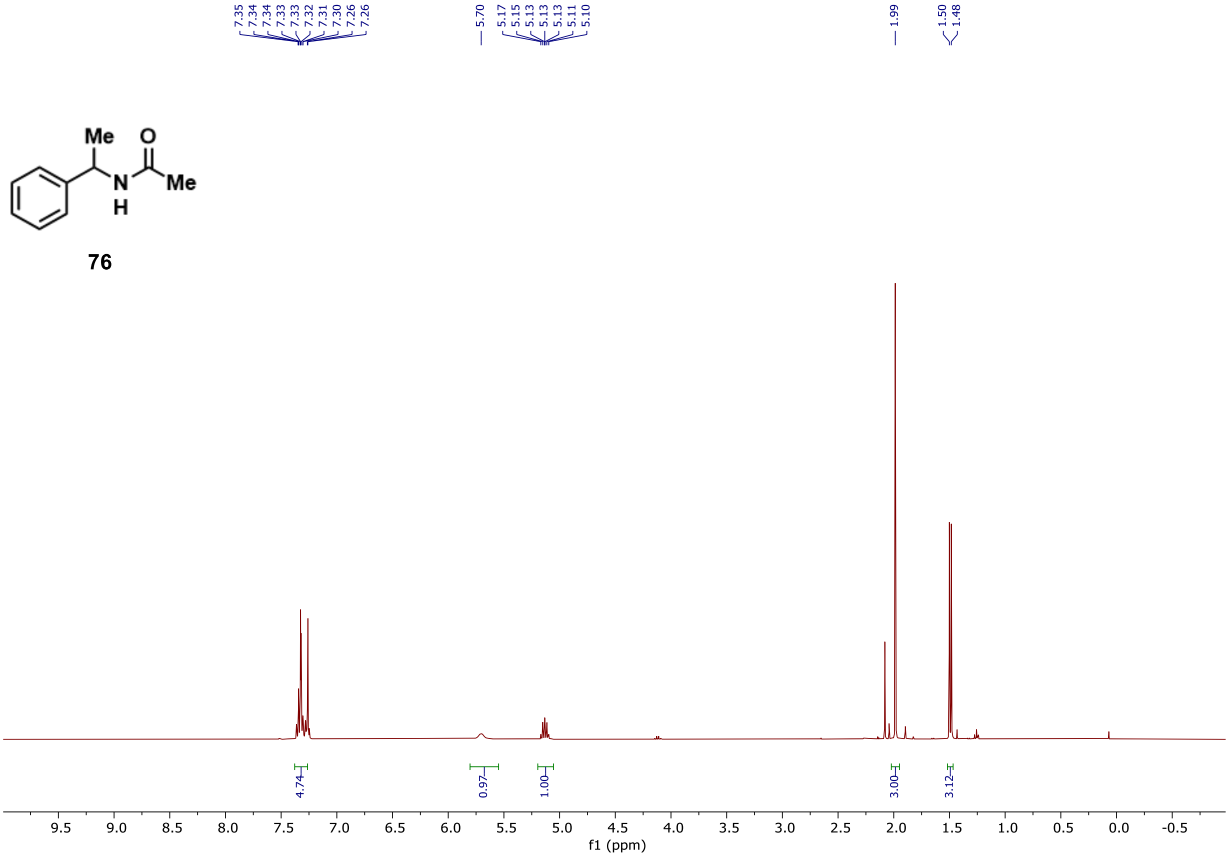

76

^{13}C NMR (101 MHz, CDCl$_3$) of **76**

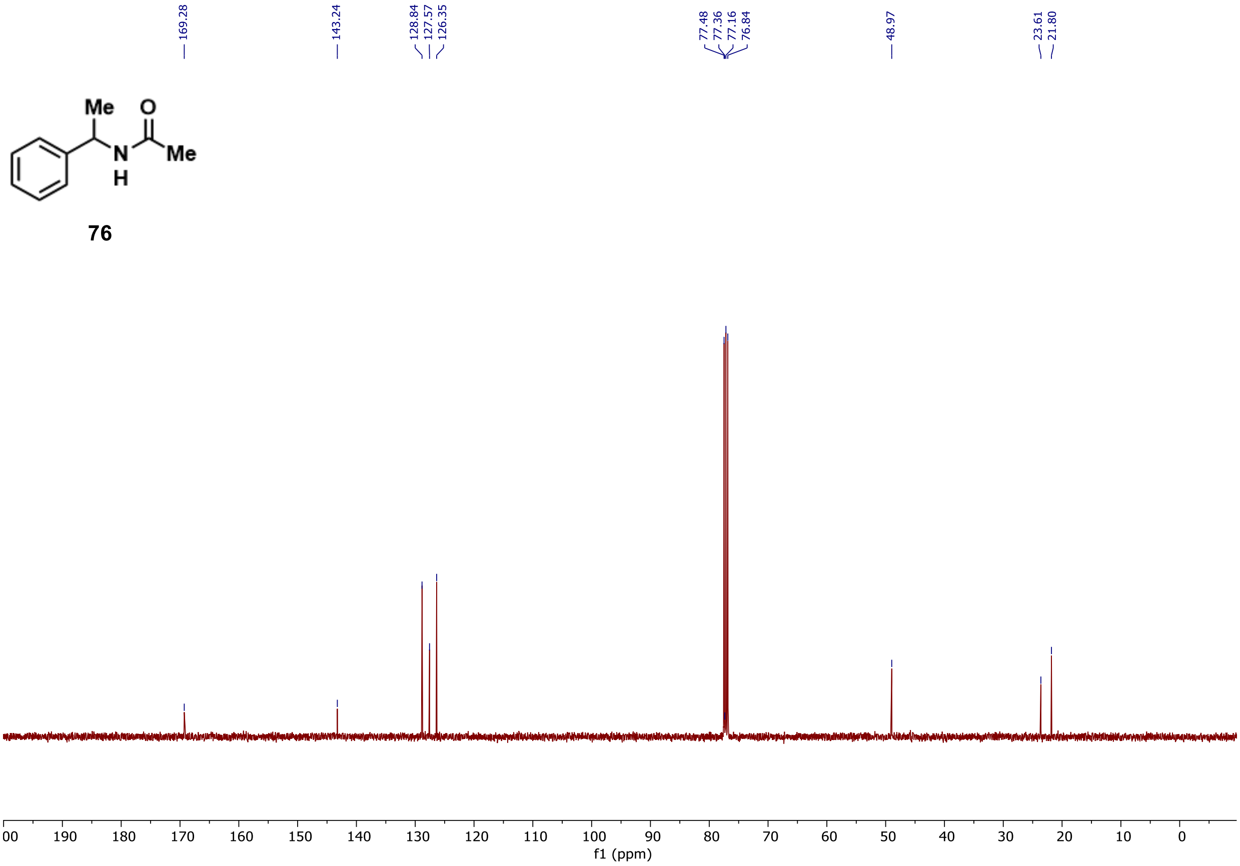

76

^{1}H NMR (400 MHz, $CDCl_3$) of **77** (***see procedure***)

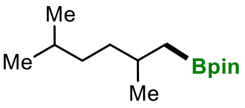

77

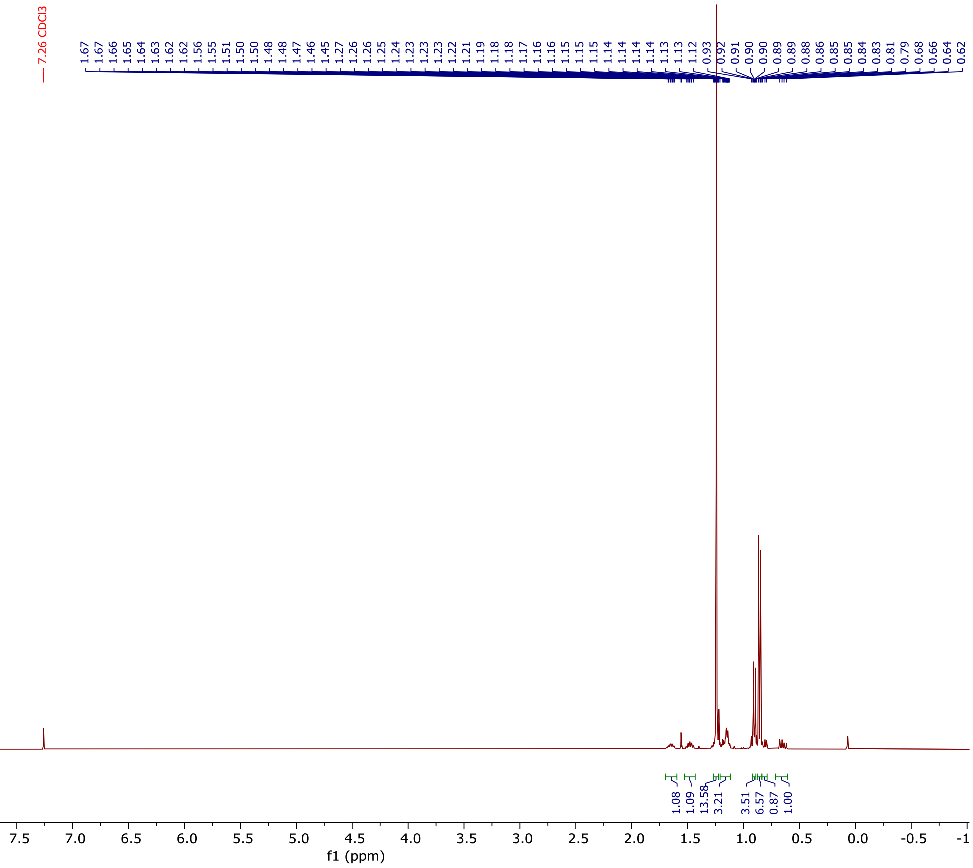

^{13}C NMR (101 MHz, $CDCl_3$) of **77**

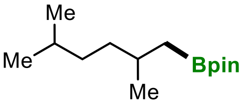

77

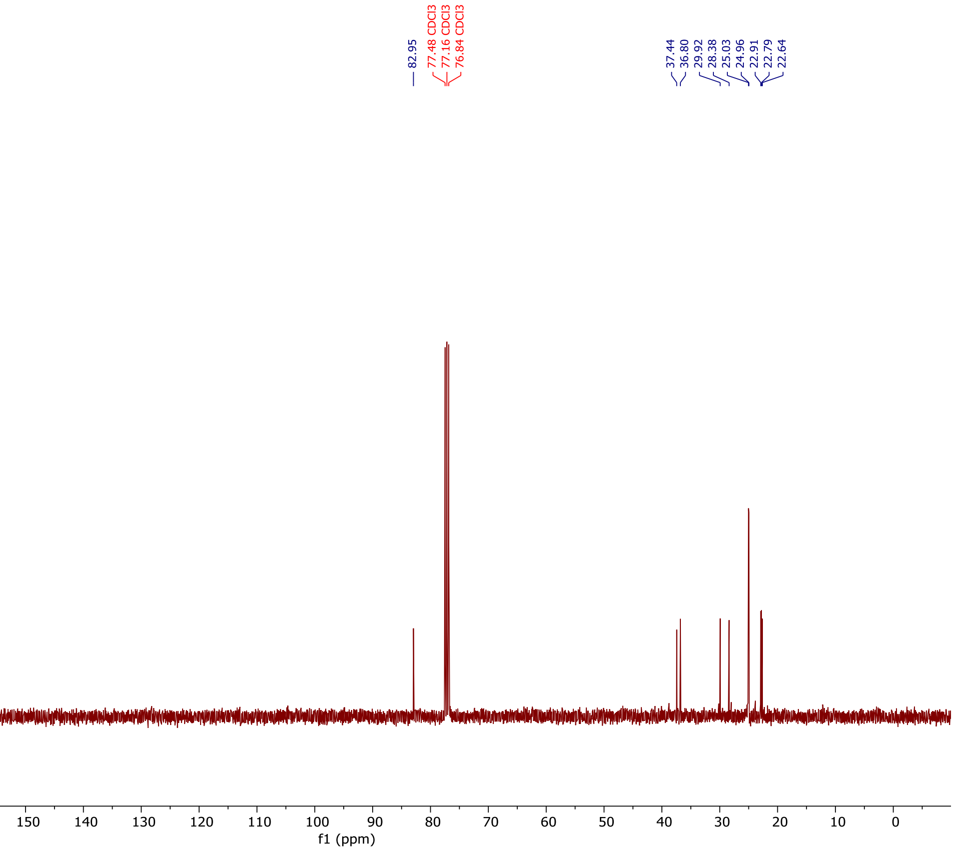

^{1}H NMR (400 MHz, $CDCl_3$) of **78** (***see procedure***)

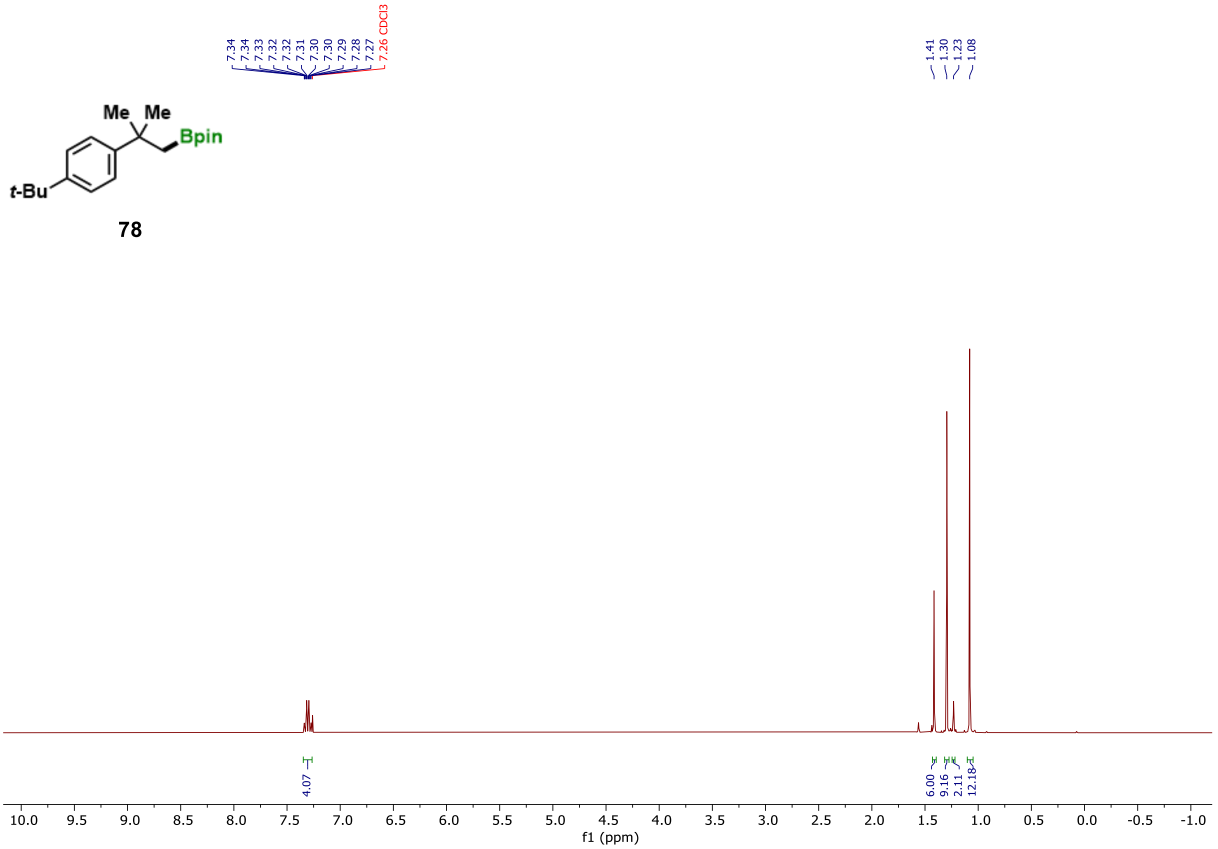

^{13}C NMR (101 MHz, $CDCl_3$) of **78**

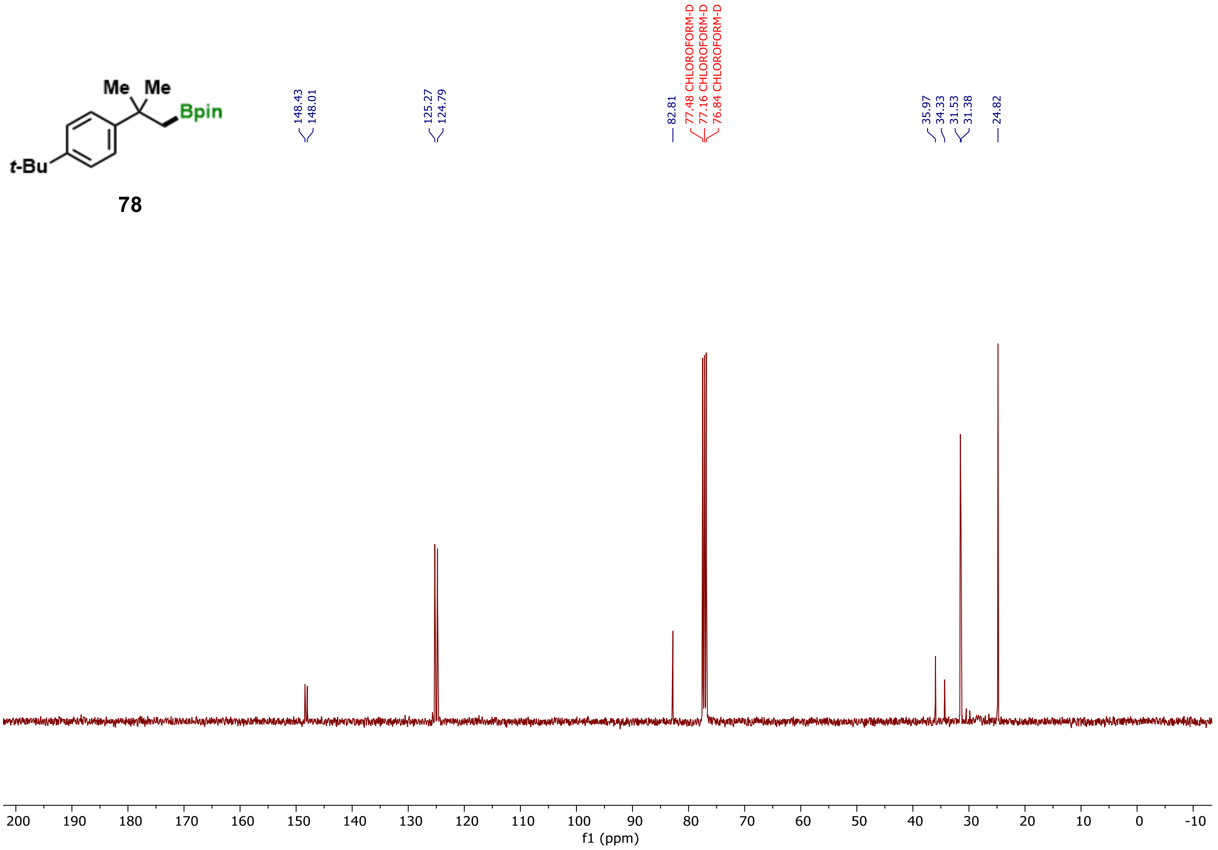

^{1}H NMR (400 MHz, $CDCl_3$) of **79** (*see procedure*)

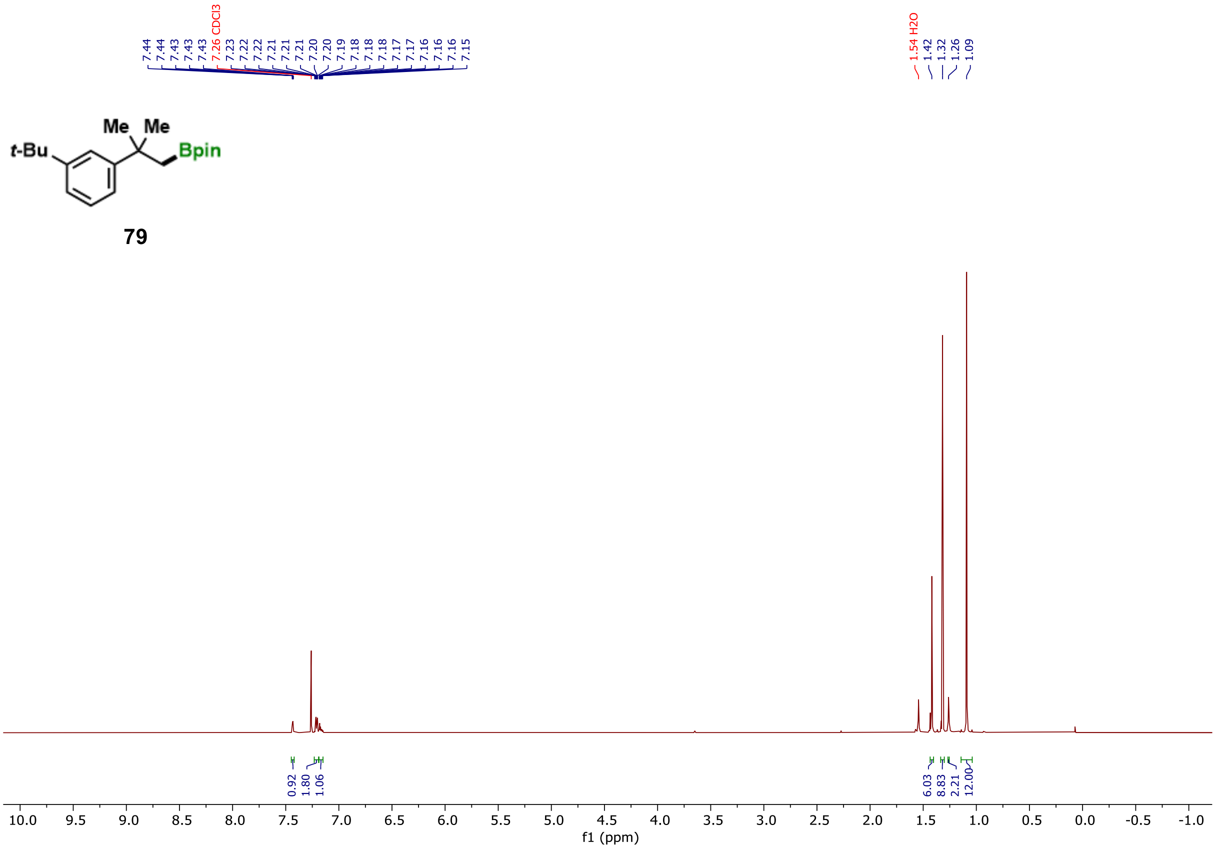

^{13}C NMR (101 MHz, $CDCl_3$) of **79**

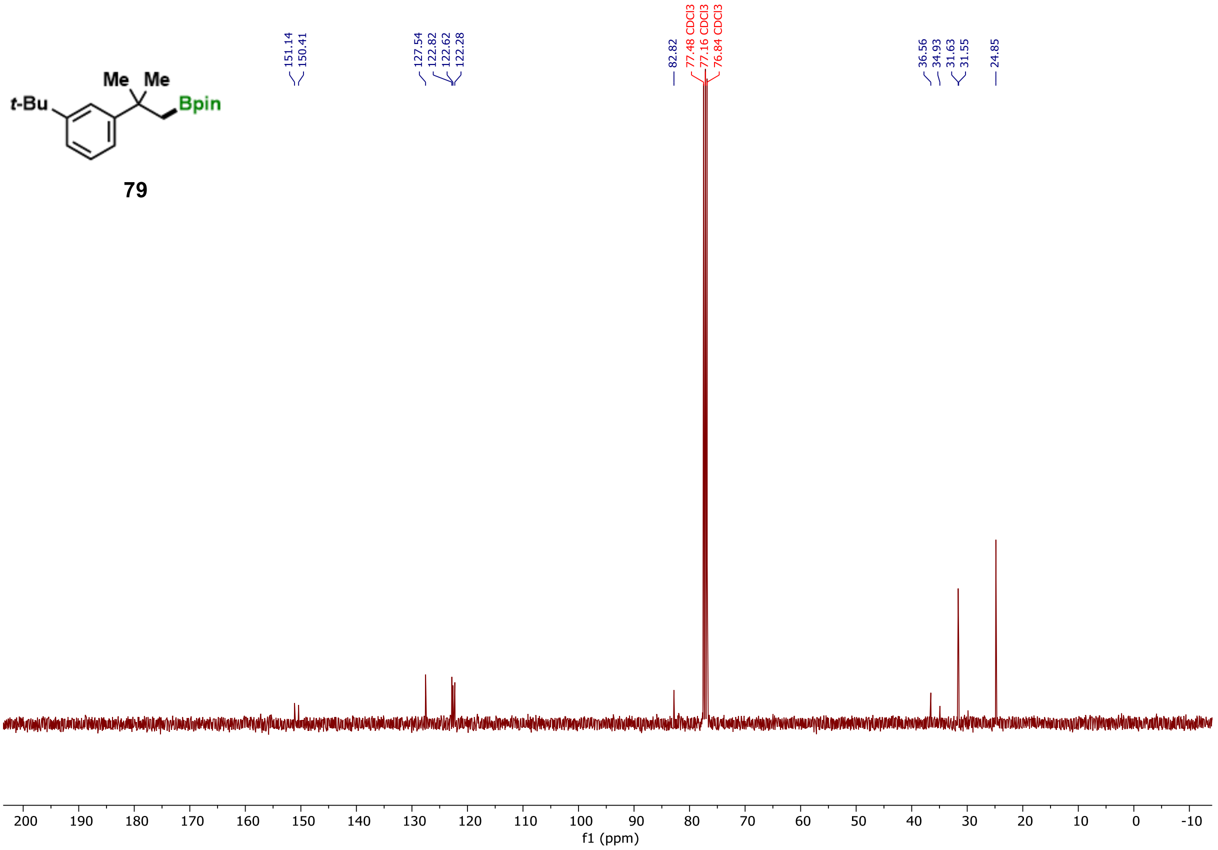

^{11}B NMR (128 MHz, $CDCl_3$) of **79**

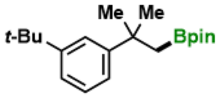

79

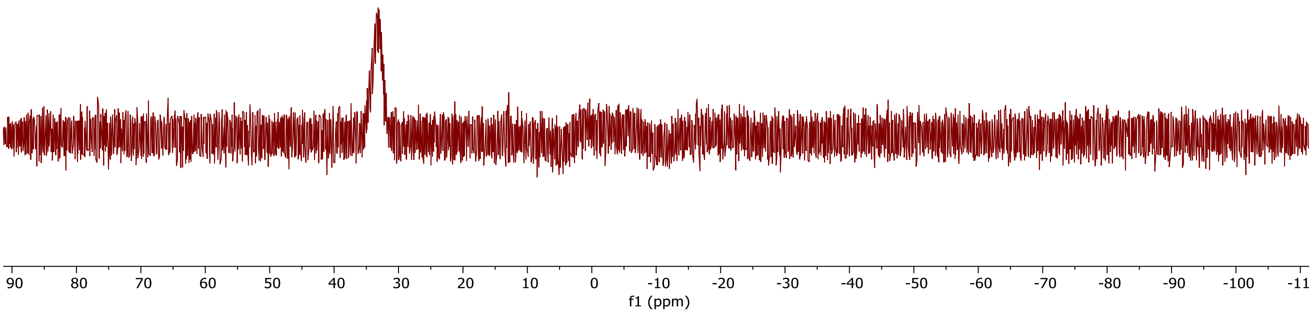

6. GC-FID DATA

All the GC-FID data recorded by analysis of the crude reaction mixtures.

GC-FID spectrum of **9** (***see procedure***)

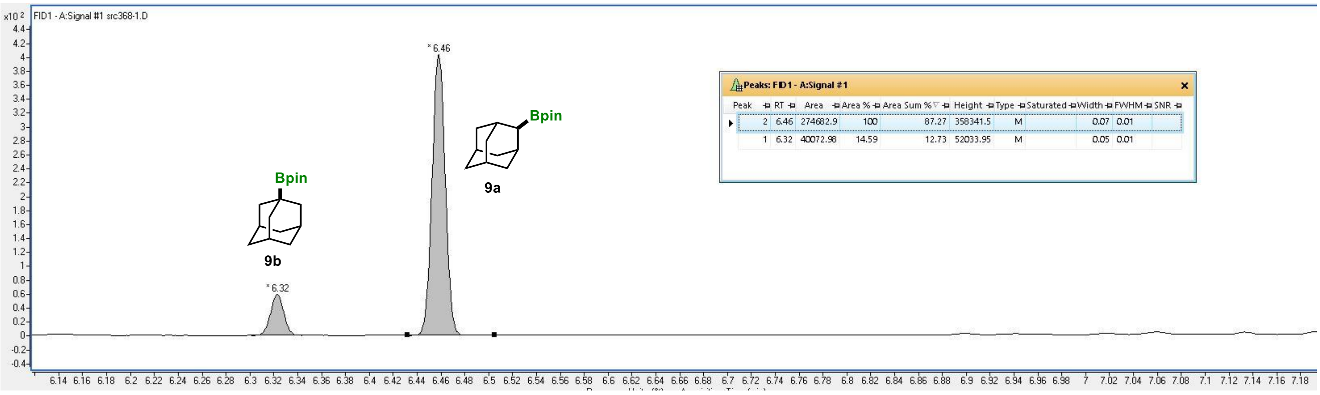

GC-FID spectrum of **10** (***see procedure***)

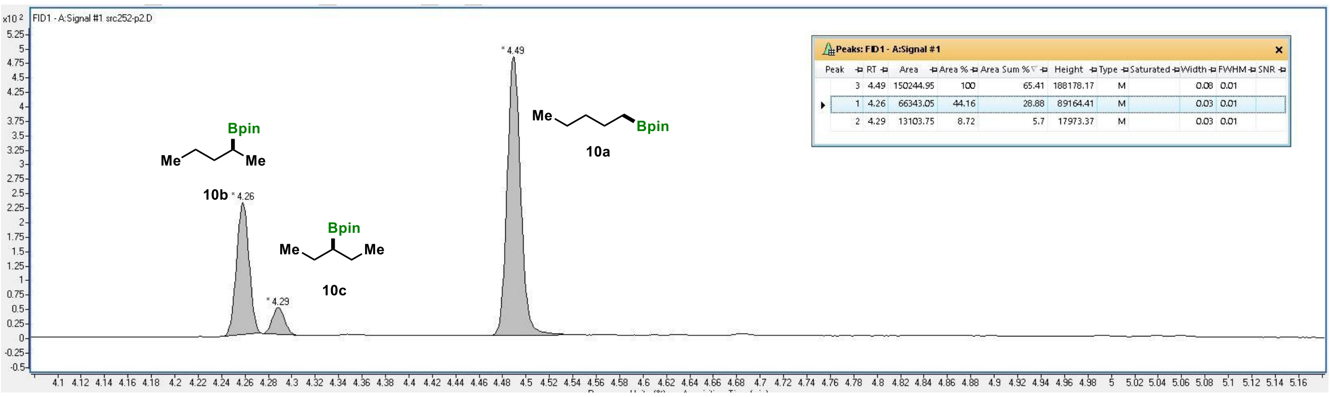

GC-FID spectrum of **11** (***see procedure***)

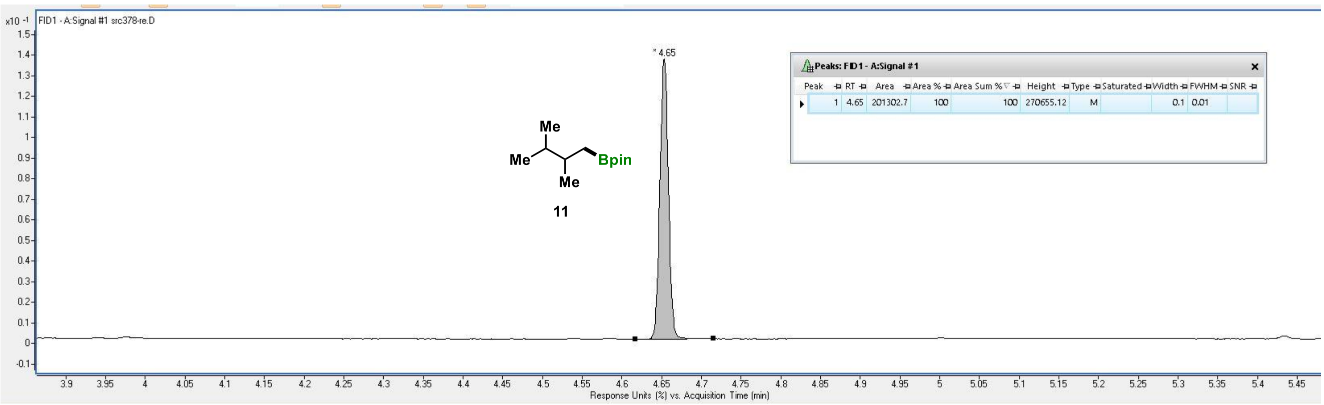

GC-FID spectrum of **12** (*see procedure*)

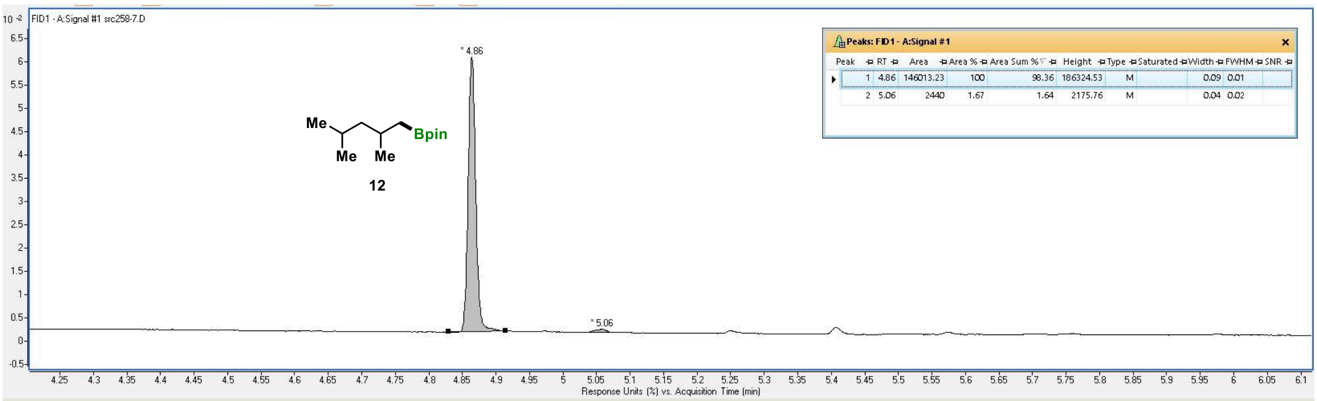

GC-FID spectrum of **14** (*see procedure*)

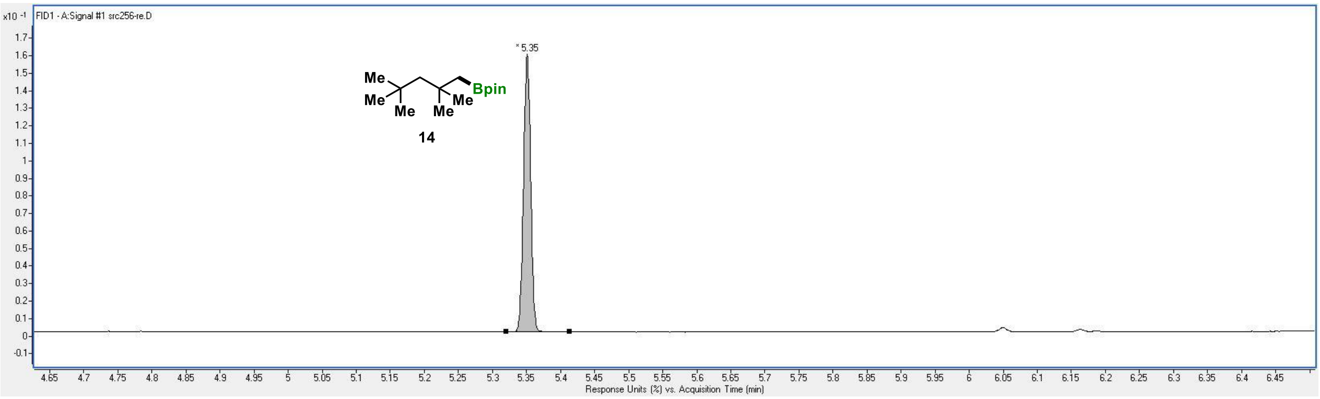

GC-FID spectrum of **15** (*see procedure*)

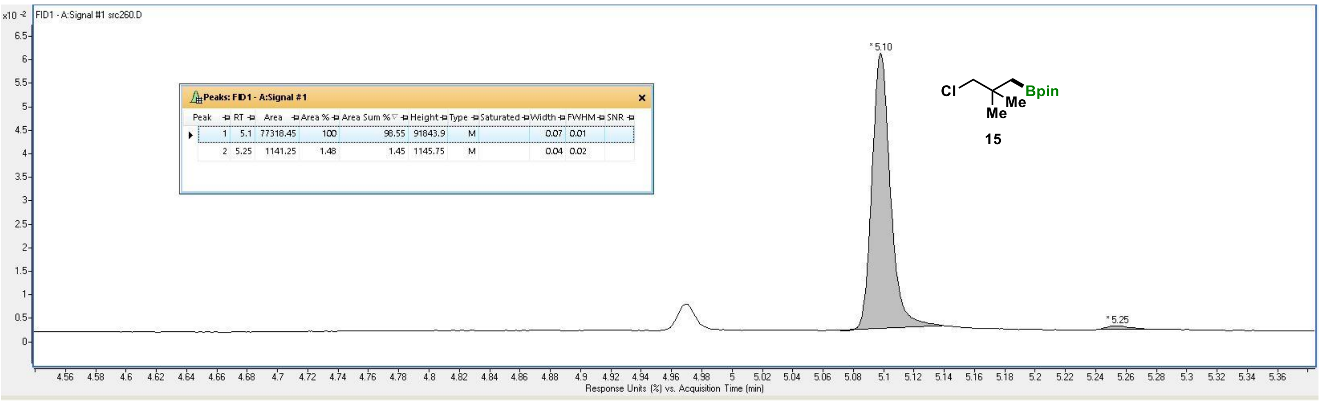

GC-FID spectrum of **16** (<u>*see procedure*</u>)

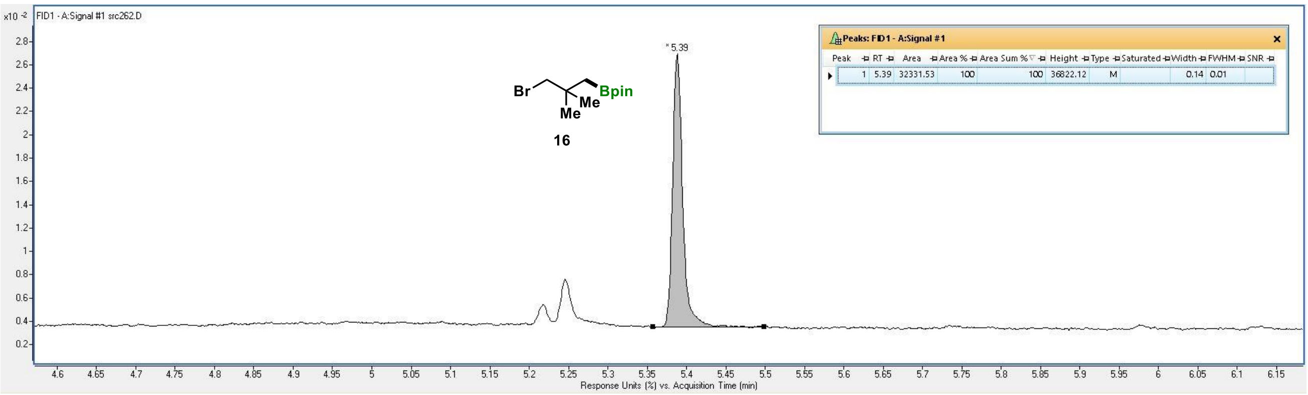

GC-FID spectrum of **17** (<u>*see procedure*</u>)

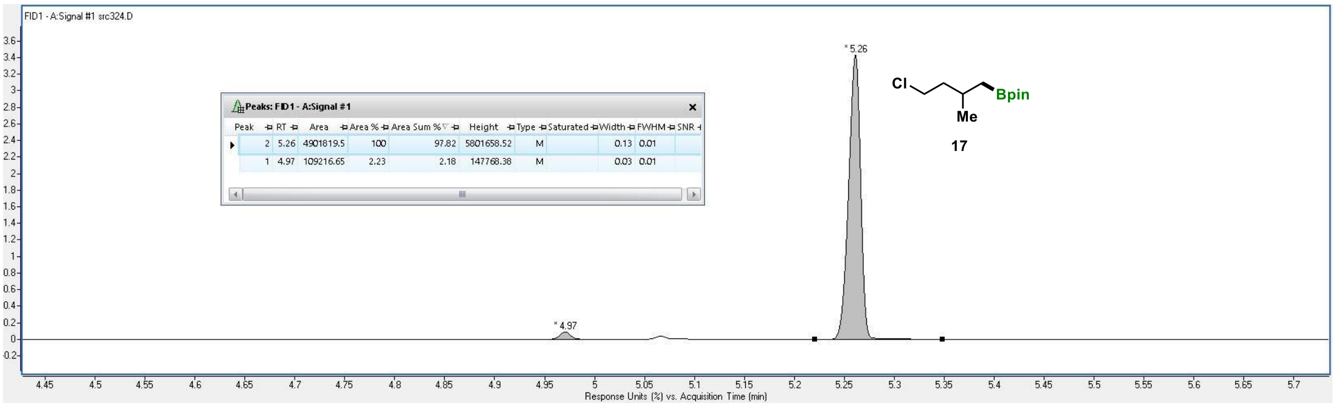

GC-FID spectrum of **18** (<u>*see procedure*</u>)

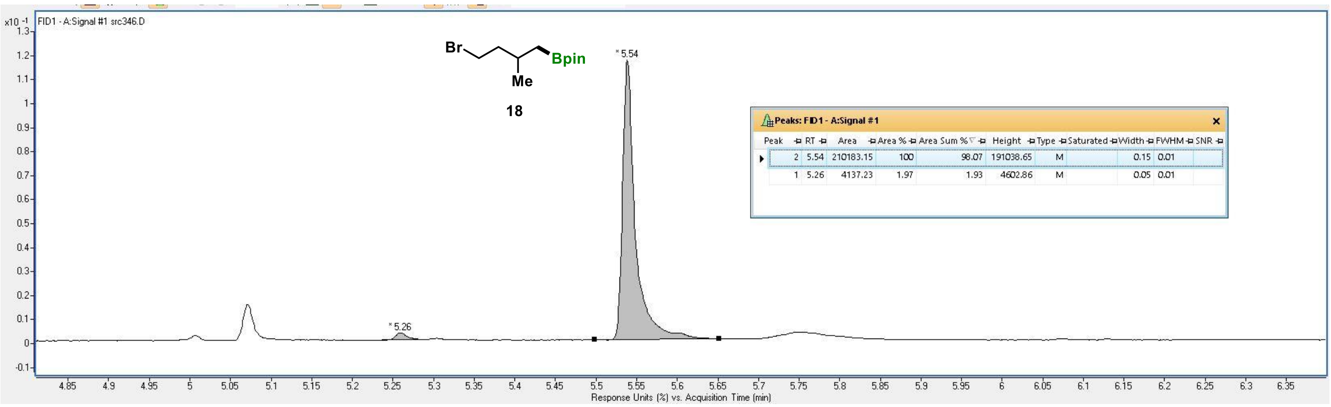

GC-FID spectrum of **19** (*see procedure*)

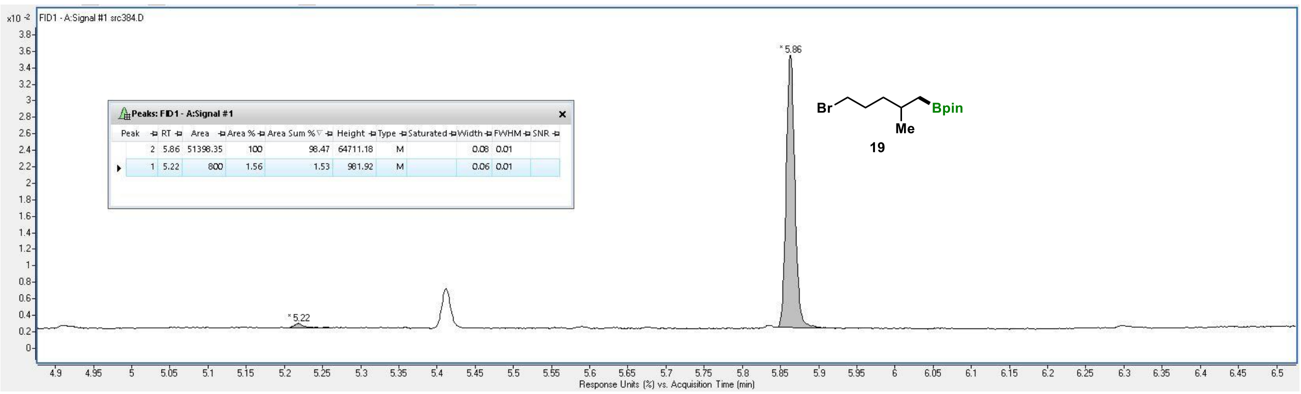

GC-FID spectrum of **20** (*see procedure*)

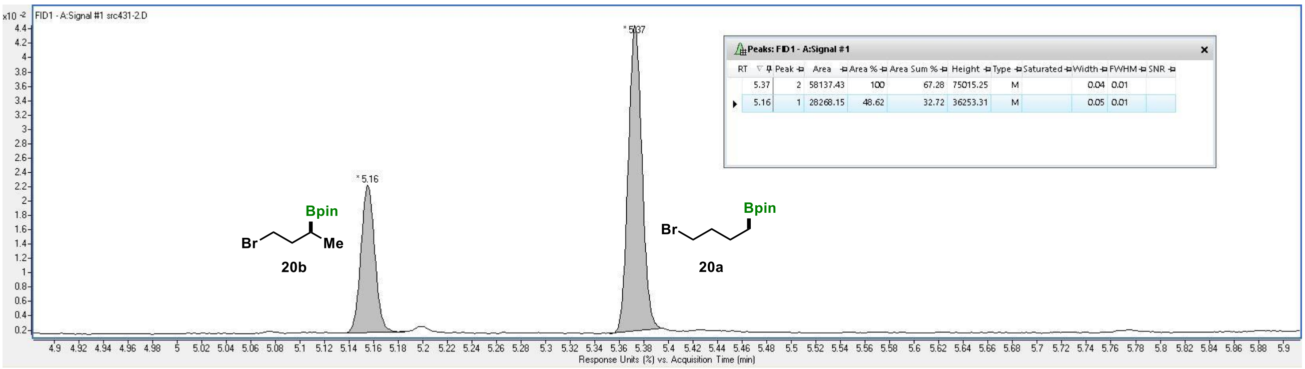

GC-FID spectrum of **21** (*see procedure*)

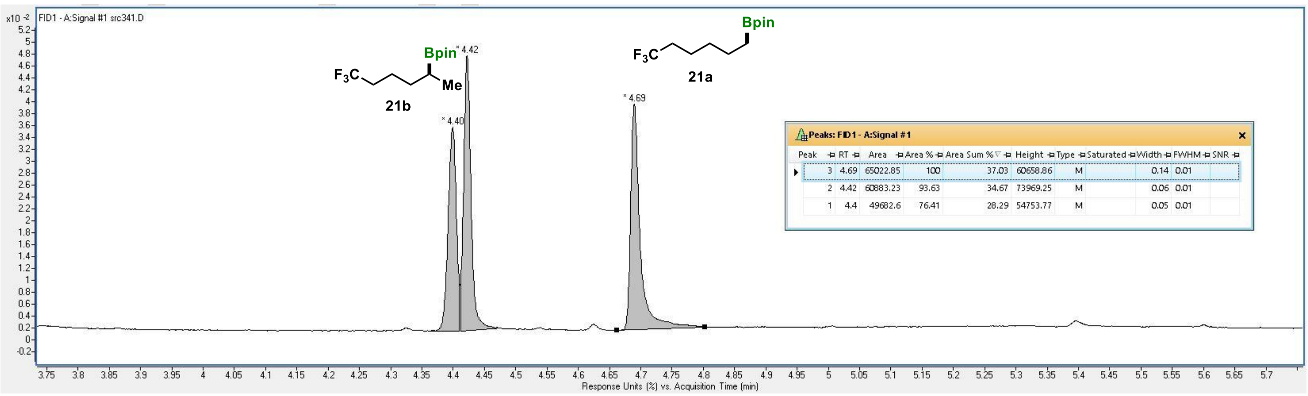

GC-FID spectrum of **22** (*see procedure*)

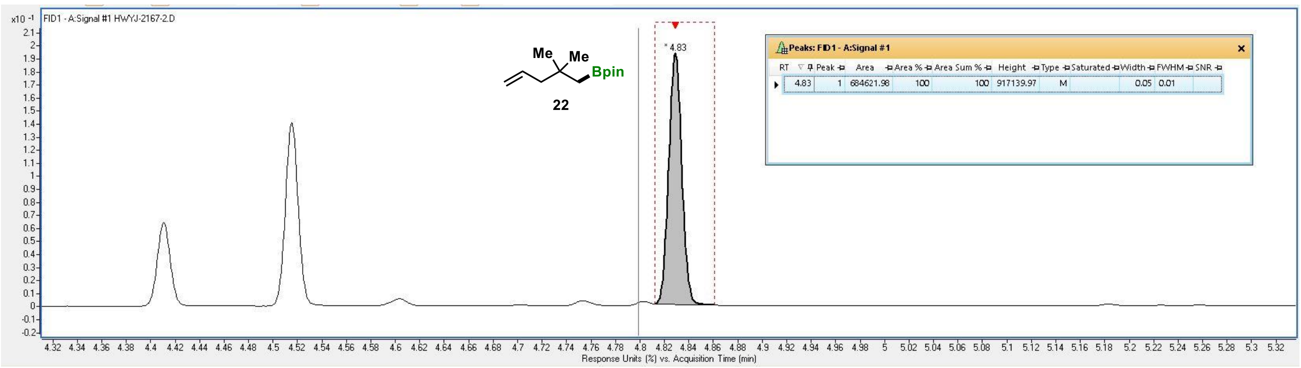

GC-FID spectrum of **25** (*see procedure*)

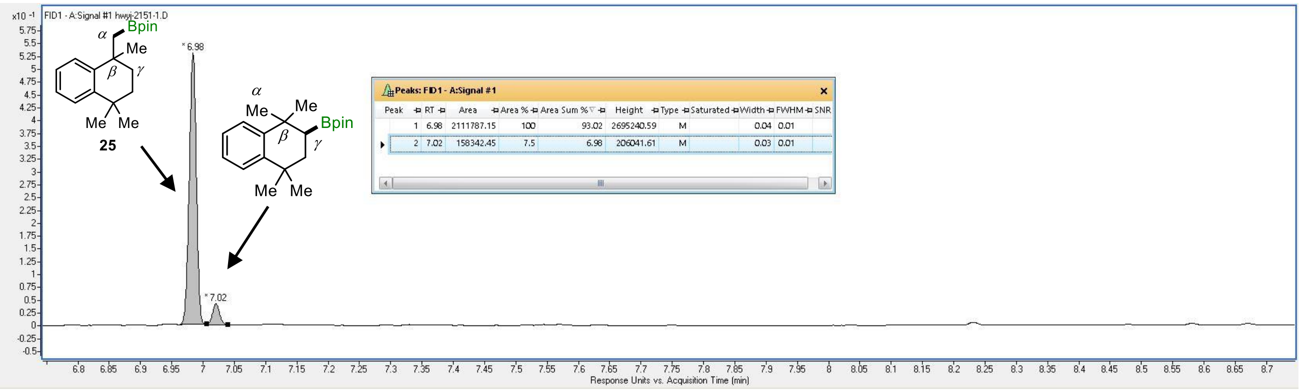

GC-FID spectrum of **26** (*see procedure*)

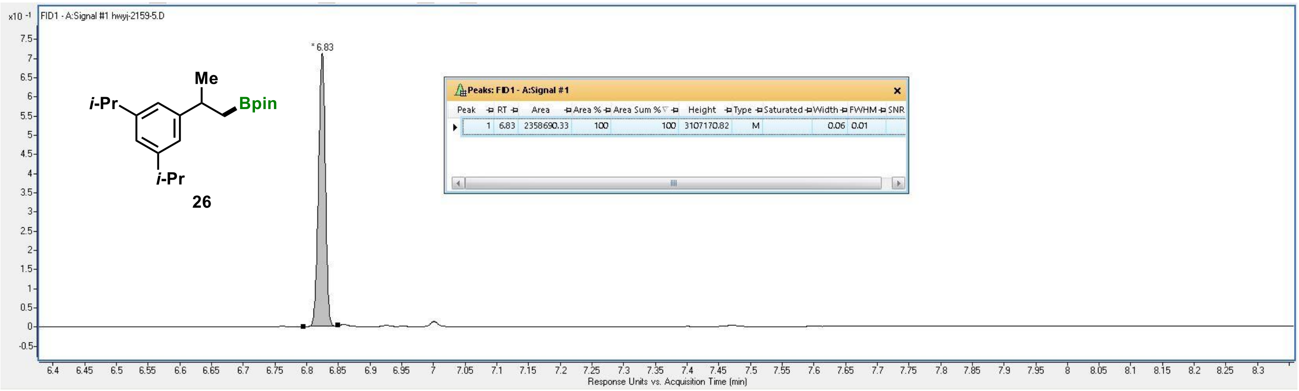

GC-FID spectrum of **27** (*see procedure*)

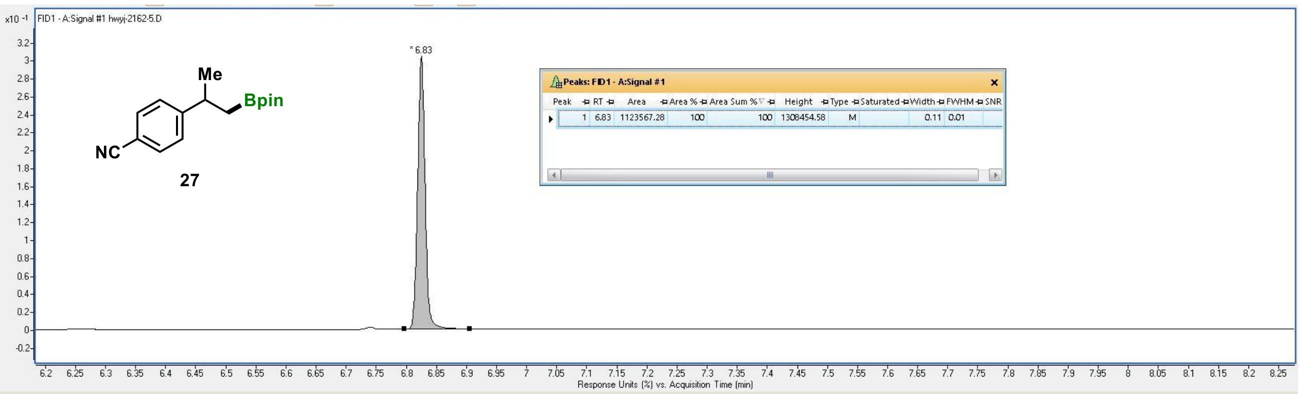

GC-FID spectrum of **29** (*see procedure*)

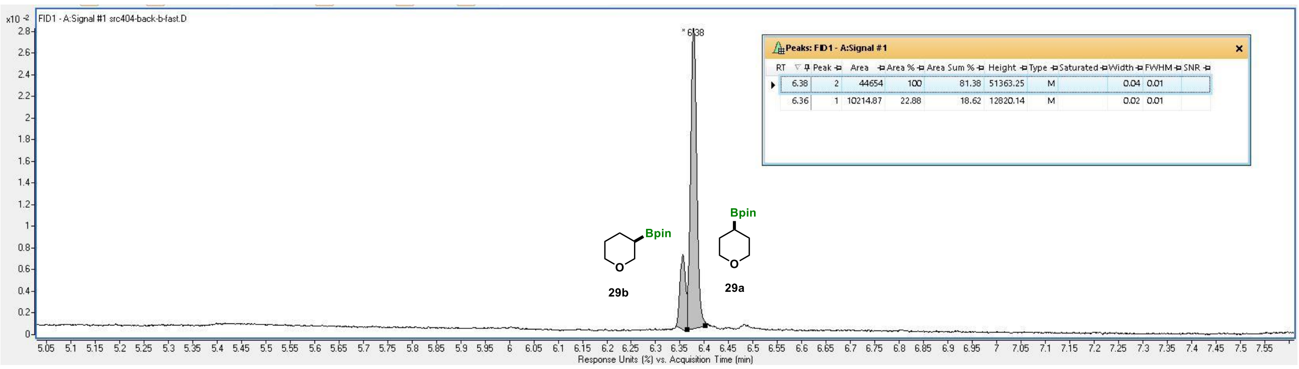

GC-FID spectrum of **30** (*see procedure*)

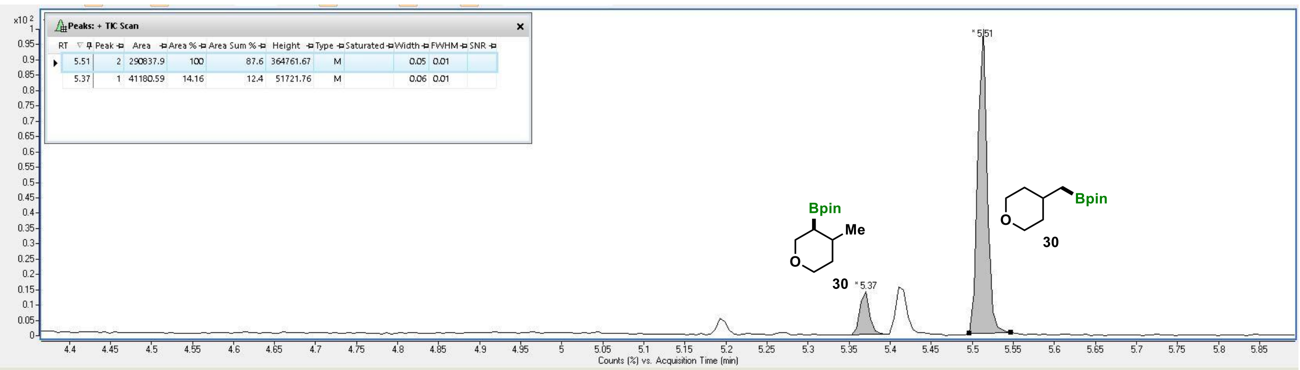

GC-FID spectrum of **31** (*see procedure*)

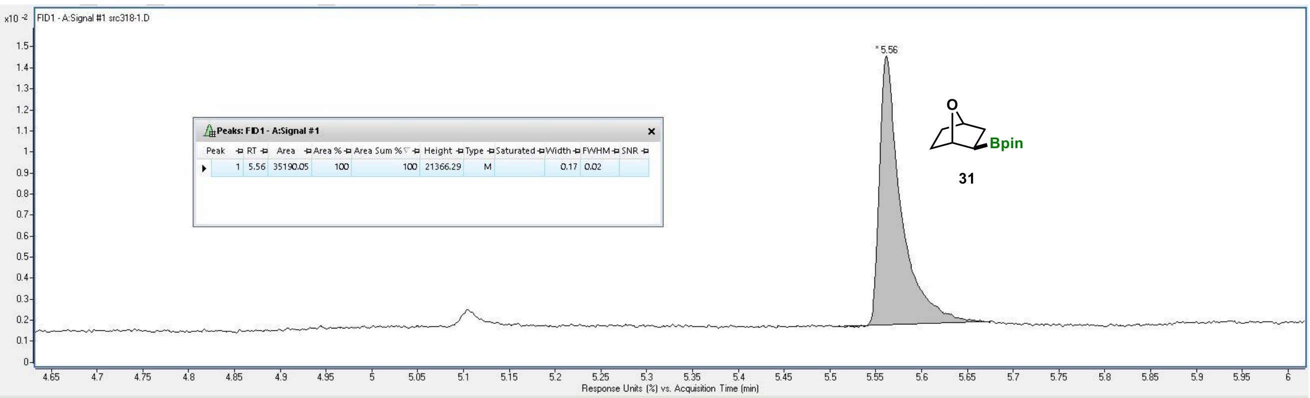

GC-FID spectrum of **32** (*see procedure*)

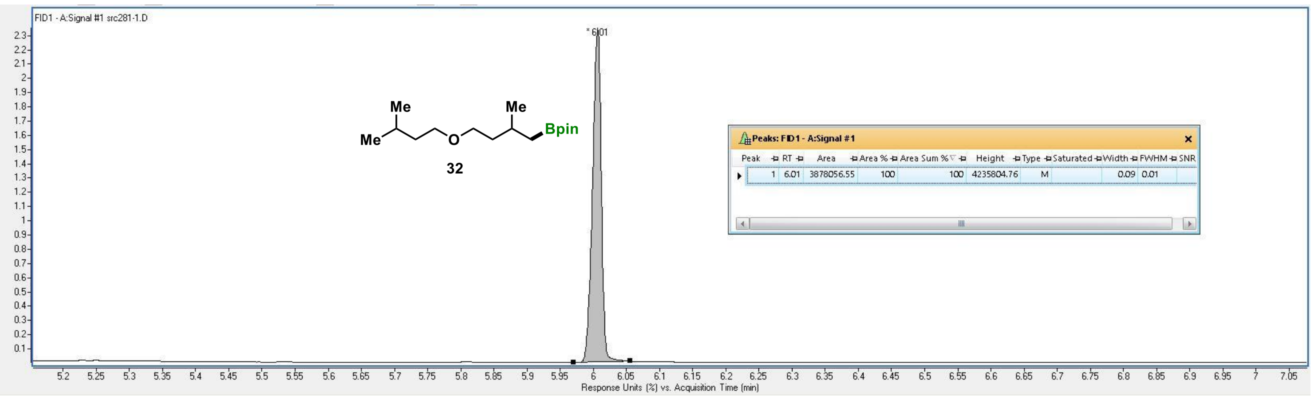

GC-FID spectrum of **33** (*see procedure*)

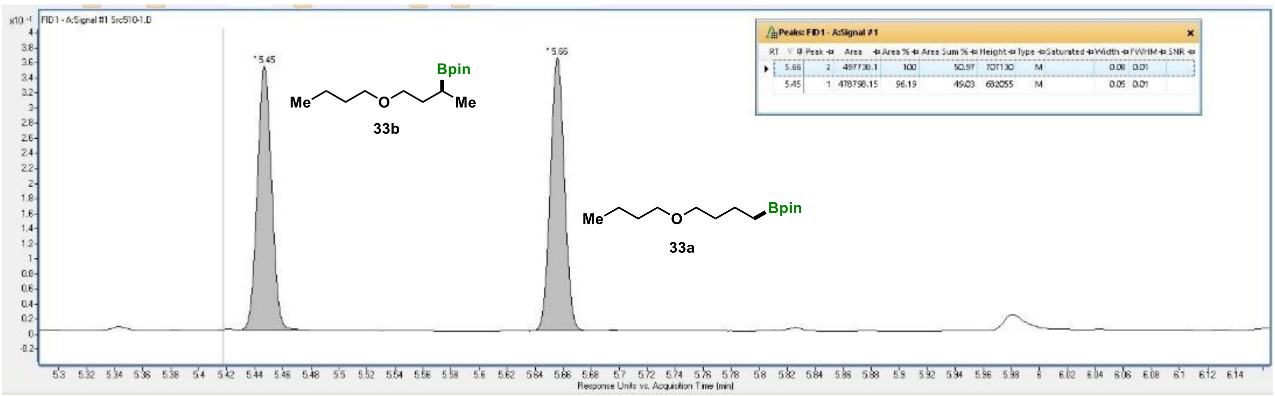

GC-FID spectrum of **34** (*see procedure*)

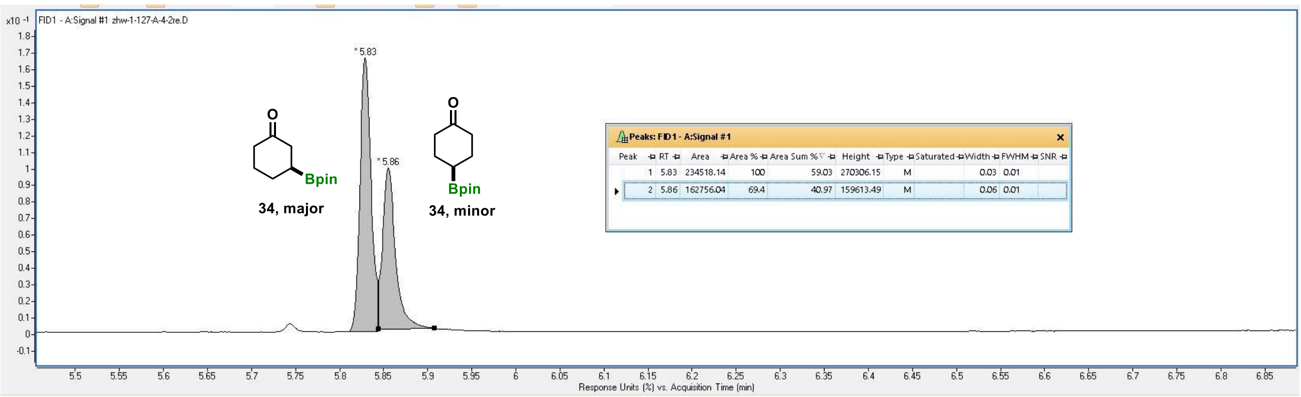

GC-FID spectrum of **35** (*see procedure*)

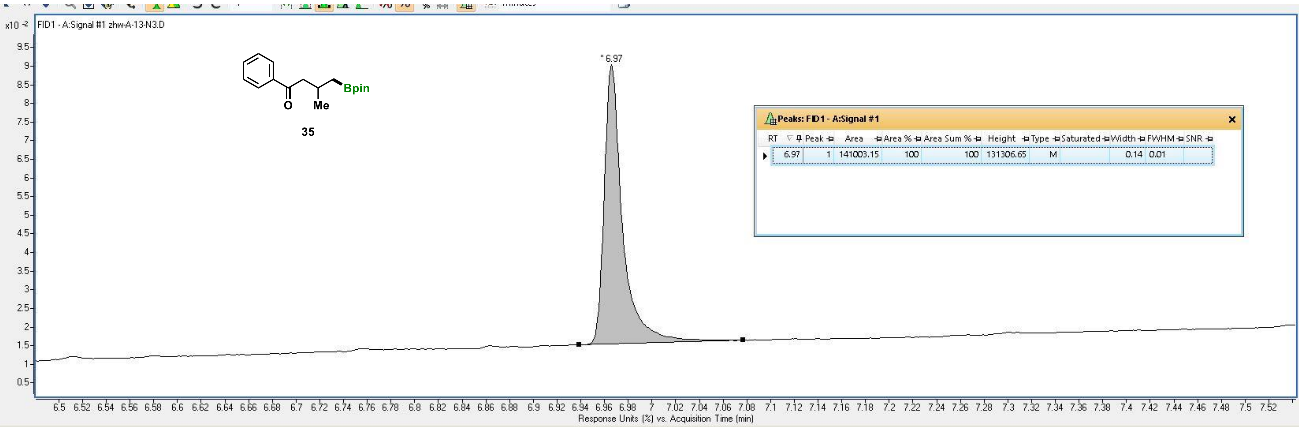

GC-FID spectrum of **36** (*see procedure*)

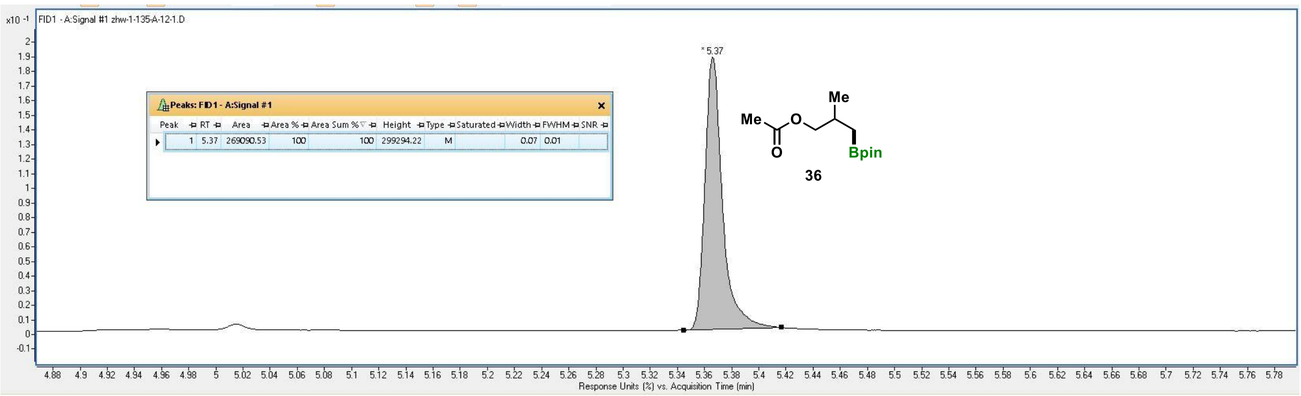

GC-FID spectrum of **37** (*see procedure*)

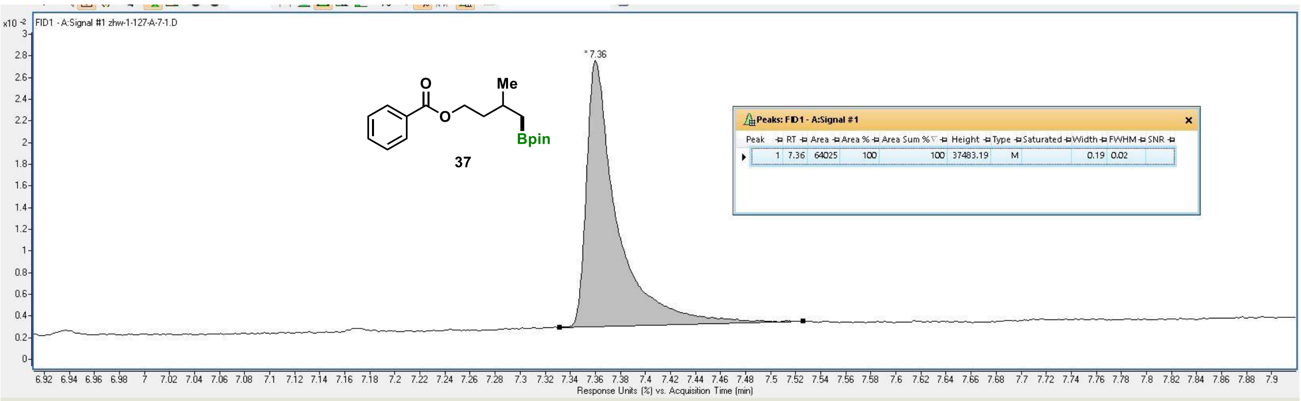

GC-FID spectrum of **39** (*see procedure*)

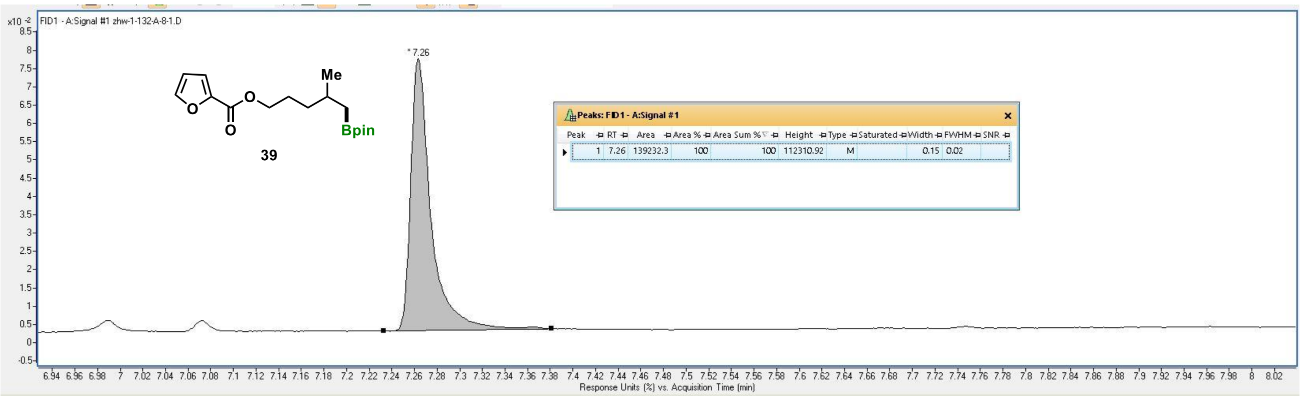

GC-FID spectrum of **40** (*see procedure*)

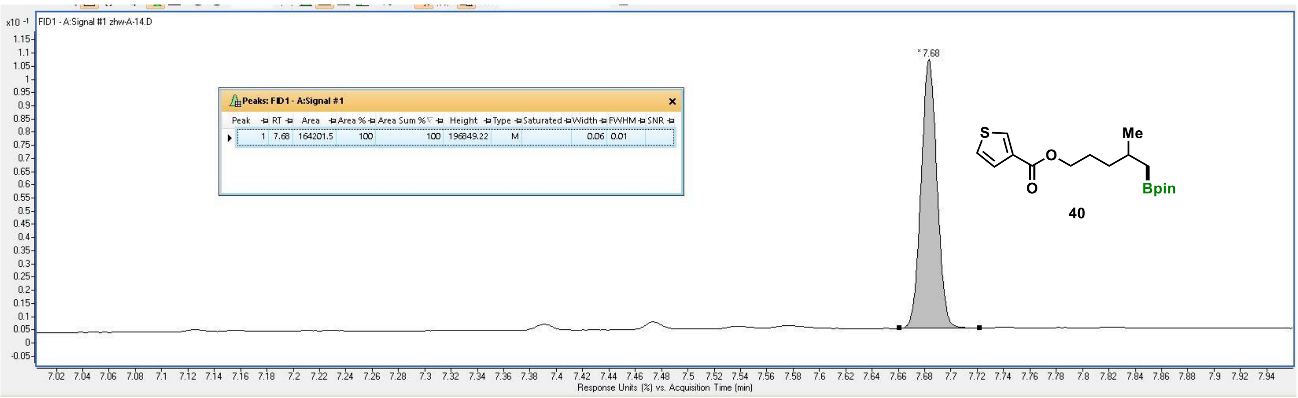

GC-FID spectrum of **41** (*see procedure*)

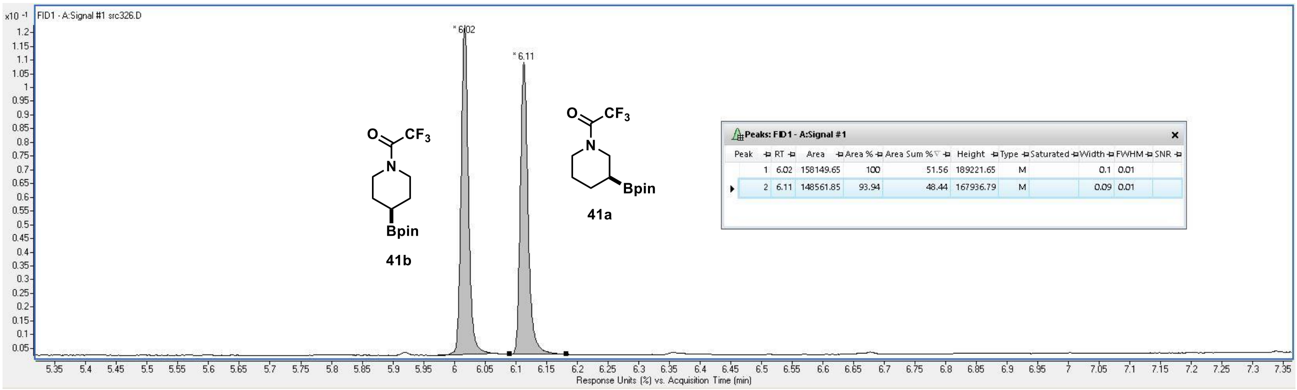

GC-FID spectrum of **42** (*see procedure*)

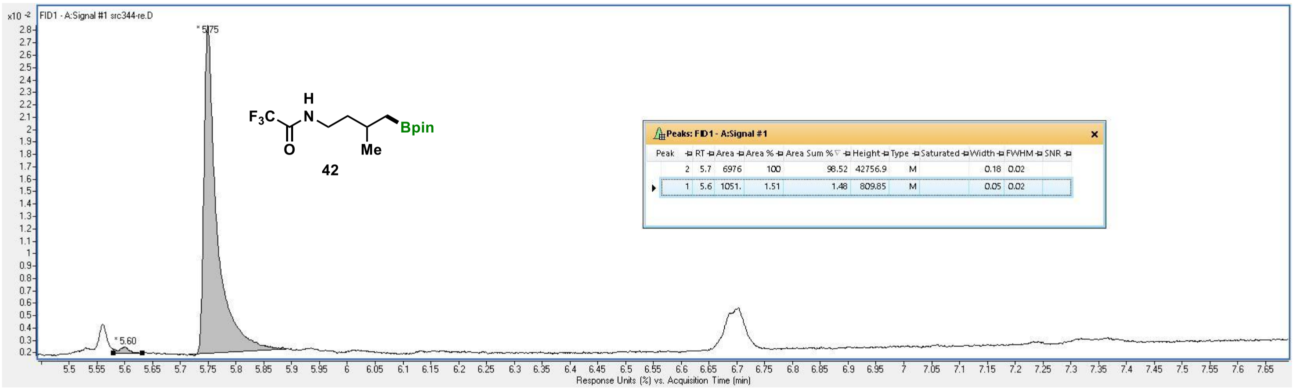

GC-FID spectrum of **43** (*see procedure*)

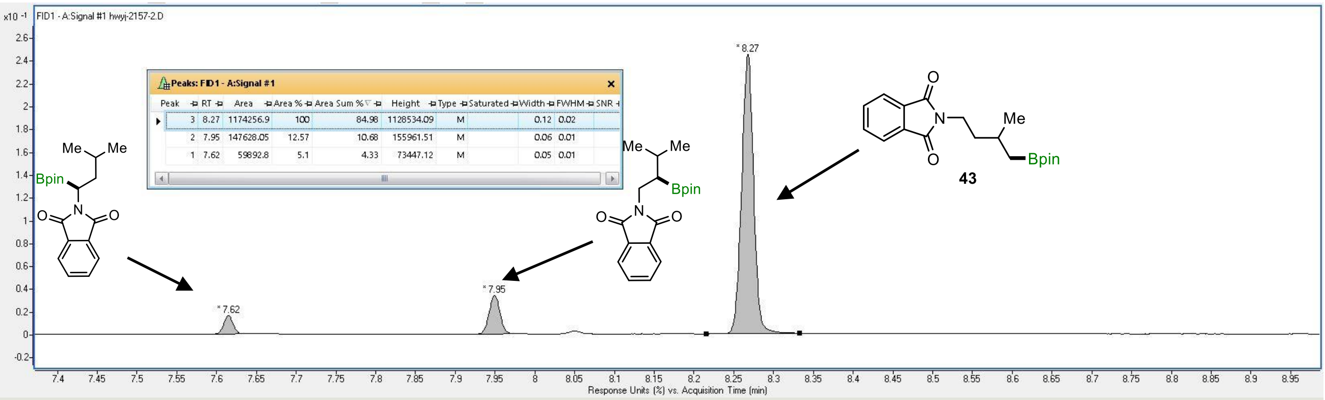

GC-FID spectrum of **44** (*see procedure*)

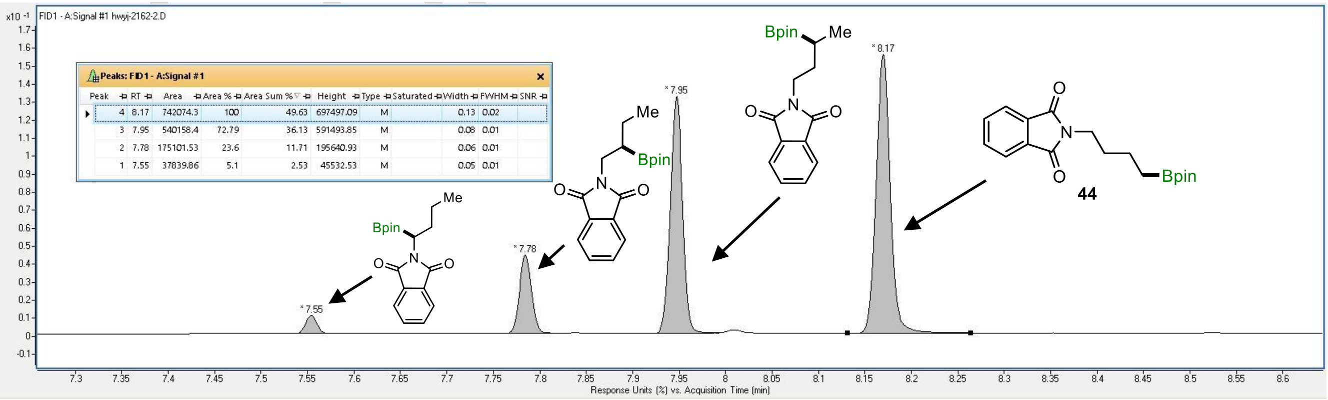

GC-FID spectrum of **45** (*see procedure*)

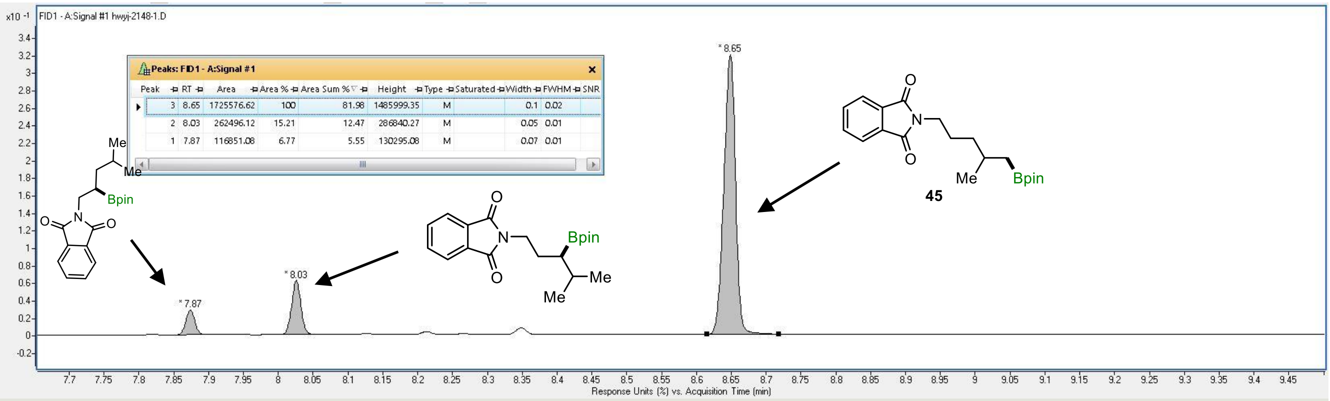

GC-FID spectrum of **46** (*see procedure*)

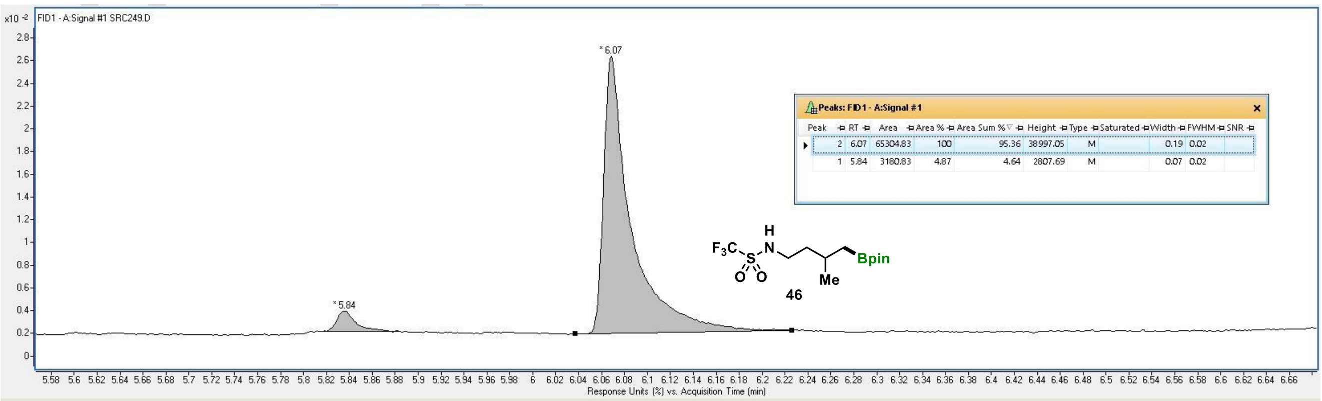

GC-FID spectrum of **47** (*see procedure*)

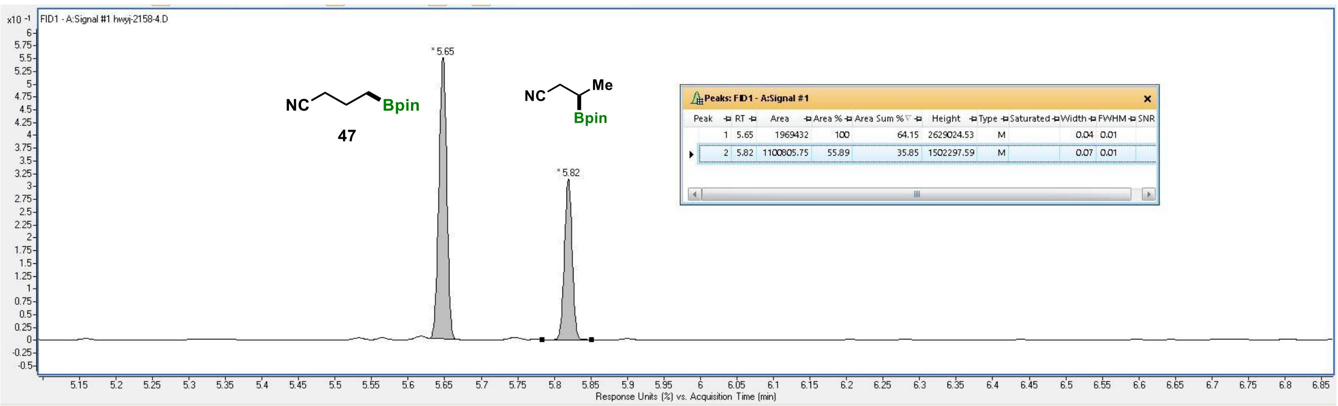

GC-FID spectrum of **48** (*see procedure*)

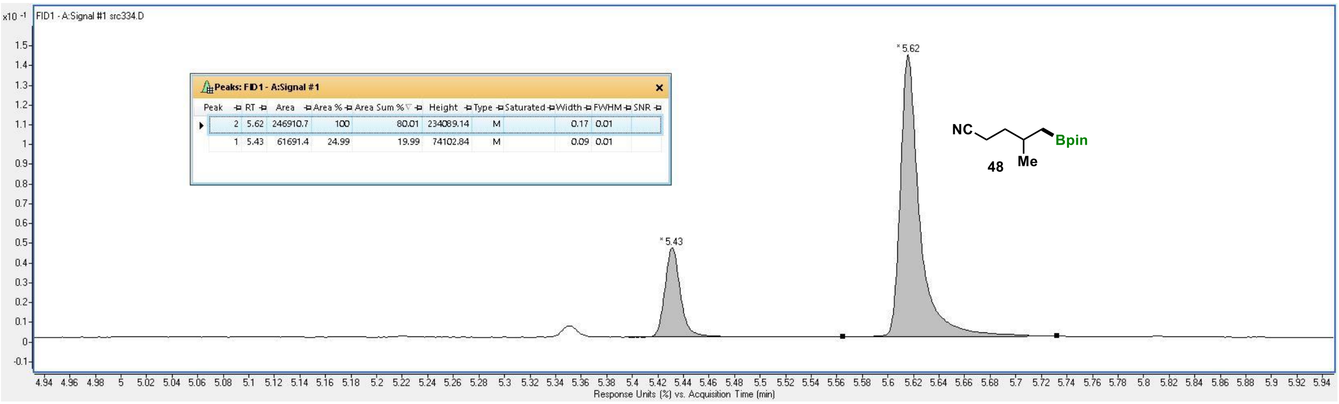

GC-FID spectrum of **49** (*see procedure*)

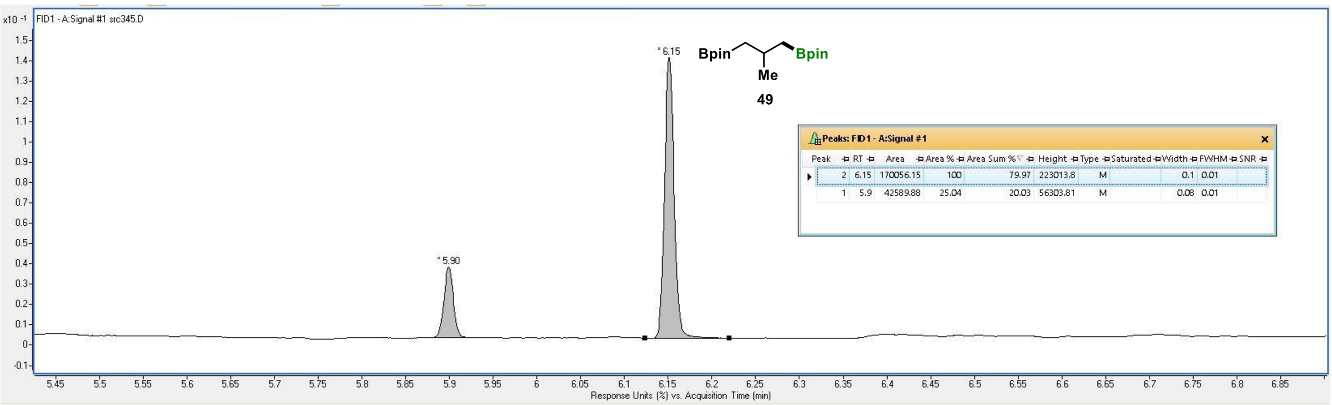

GC-FID spectrum of **51** (*see procedure*)

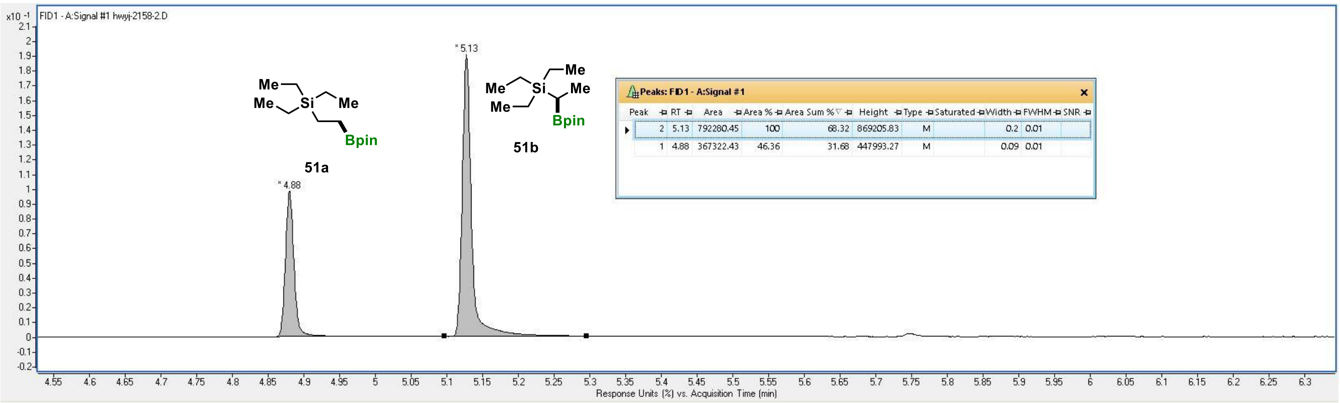

GC-FID spectrum of **52** (*see procedure*)

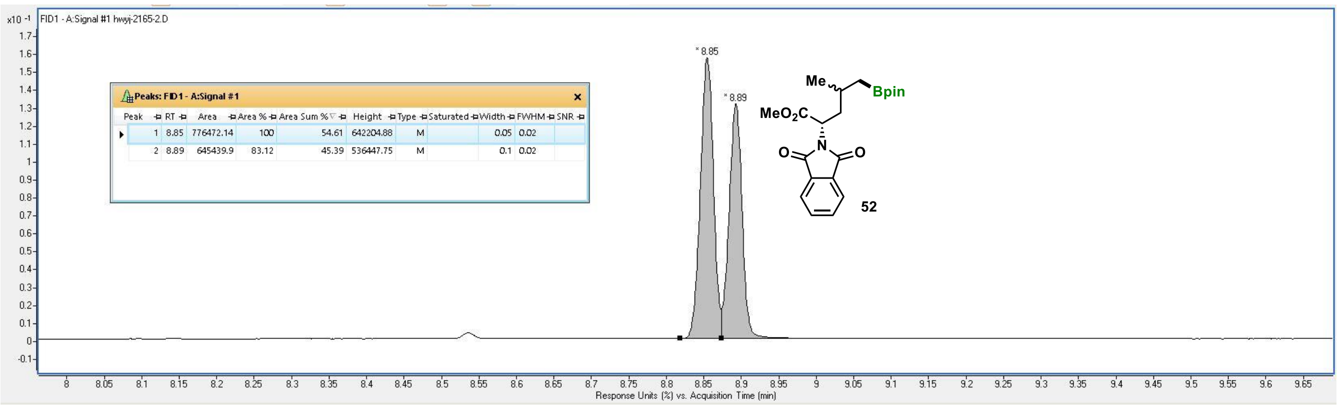

GC-FID spectrum of **53** (*see procedure*)

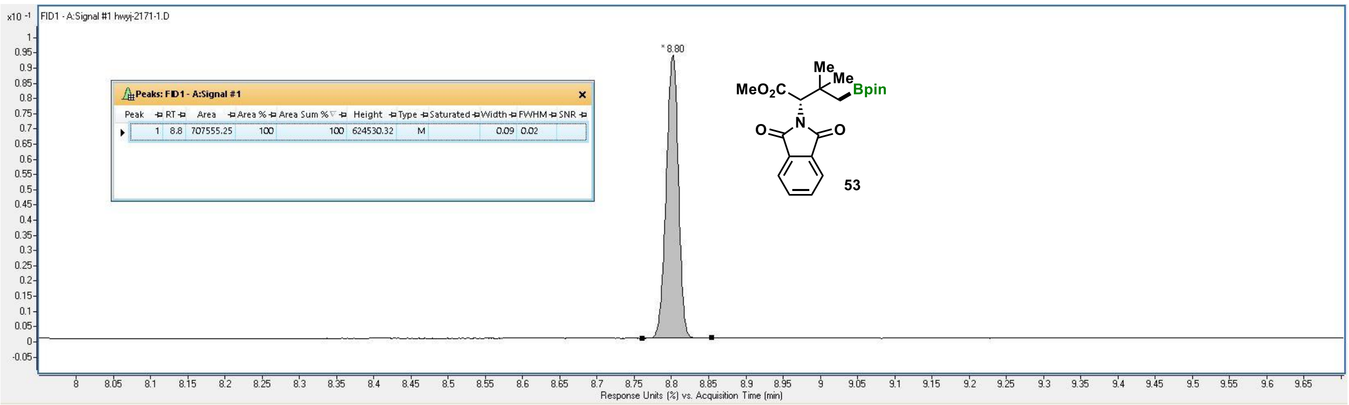

GC-FID spectrum of **54** (*see procedure*)

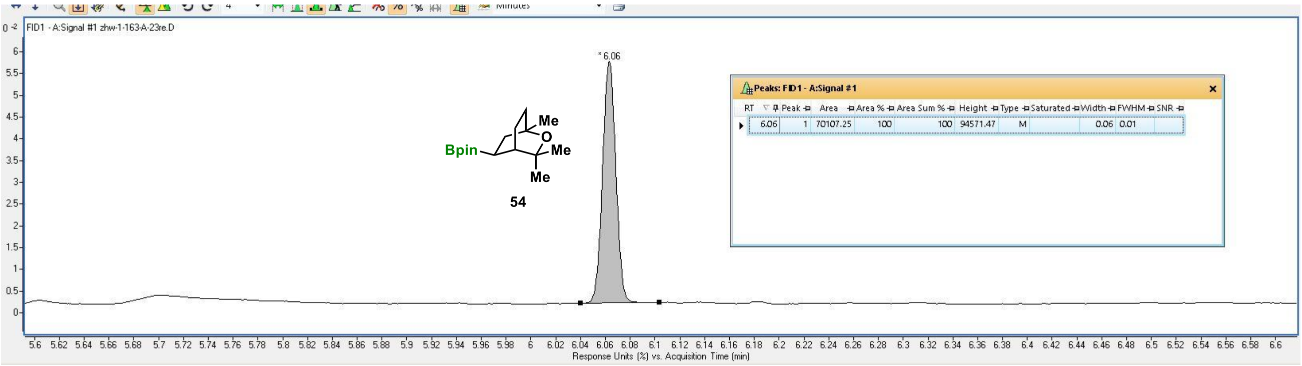

GC-FID spectrum of **59** (*see procedure*)

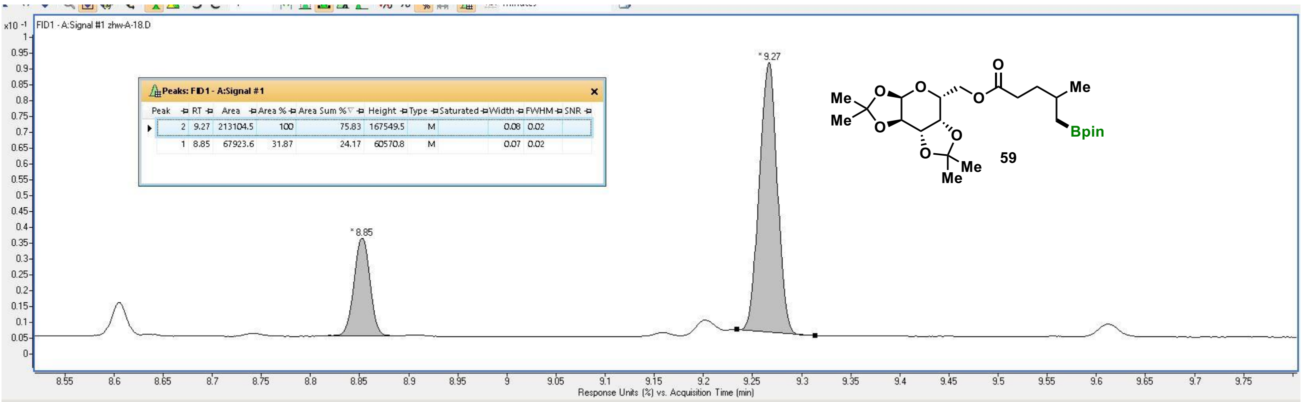

GC-FID spectrum of **60** (*see procedure*)

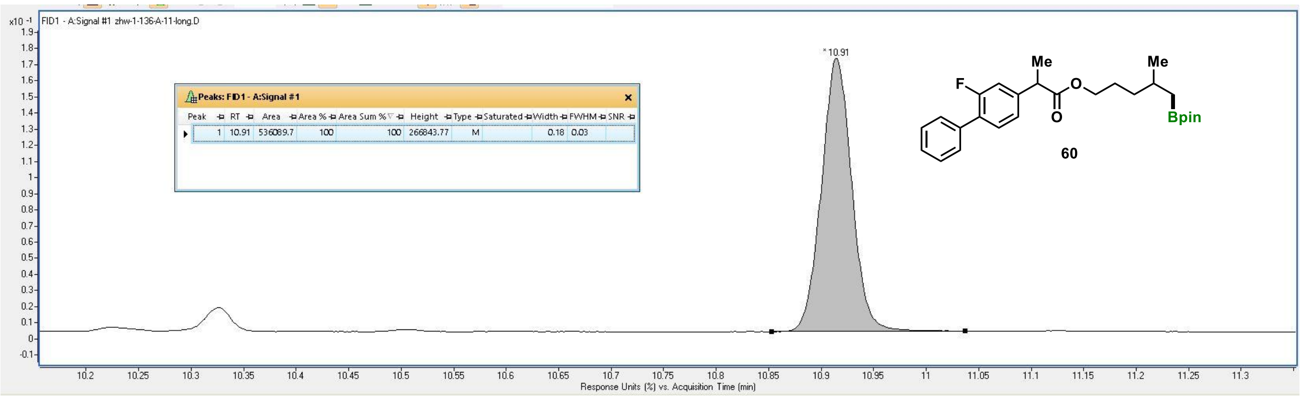

GC-FID spectrum of **61** (*see procedure*)

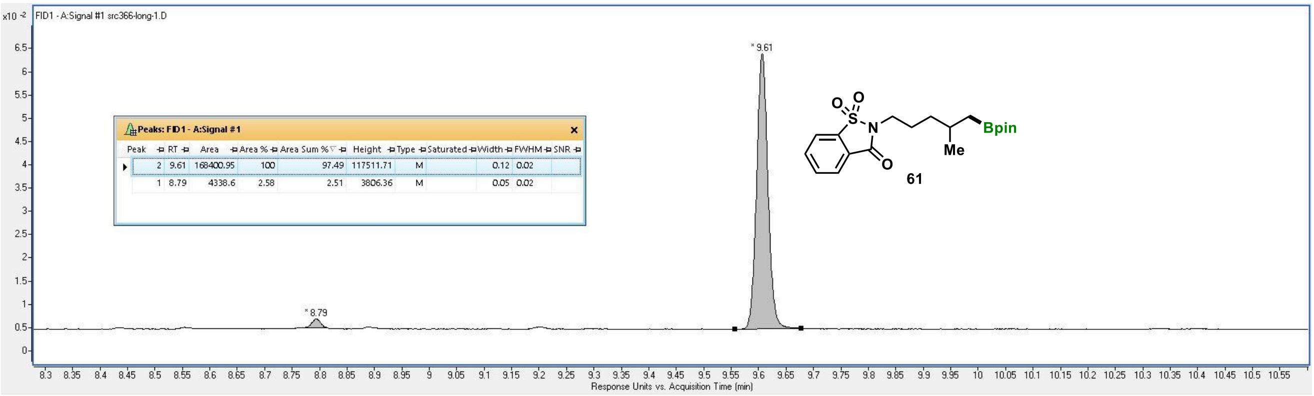

GC-FID spectrum of **62** (*see procedure*)

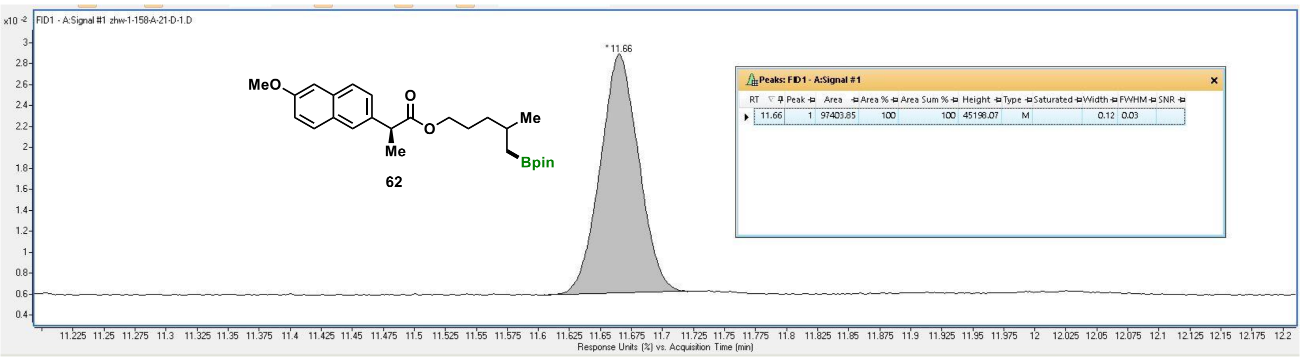

GC-FID spectrum of competition experiment of 2,3-dimethylbutane without B_2cat_2 (*see procedure*)

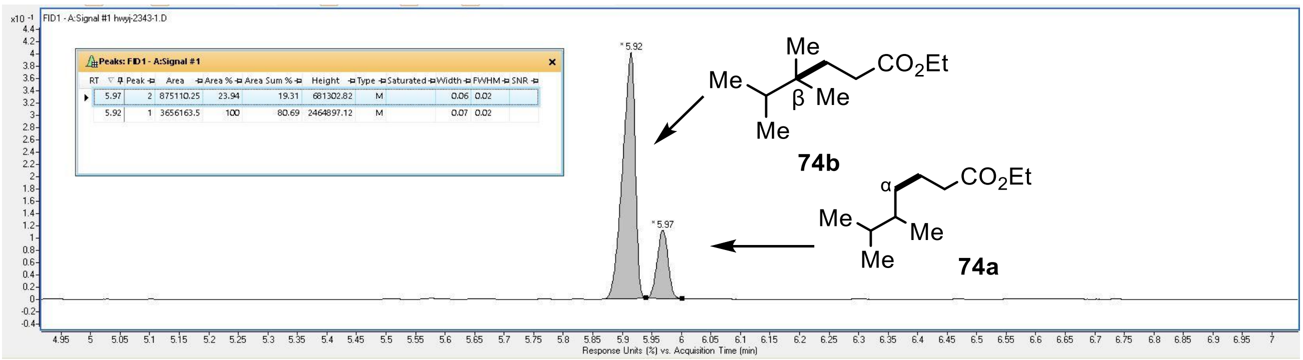

GC-FID spectrum of competition experiment of 2,3-dimethylbutane with B_2cat_2 (*see procedure*)

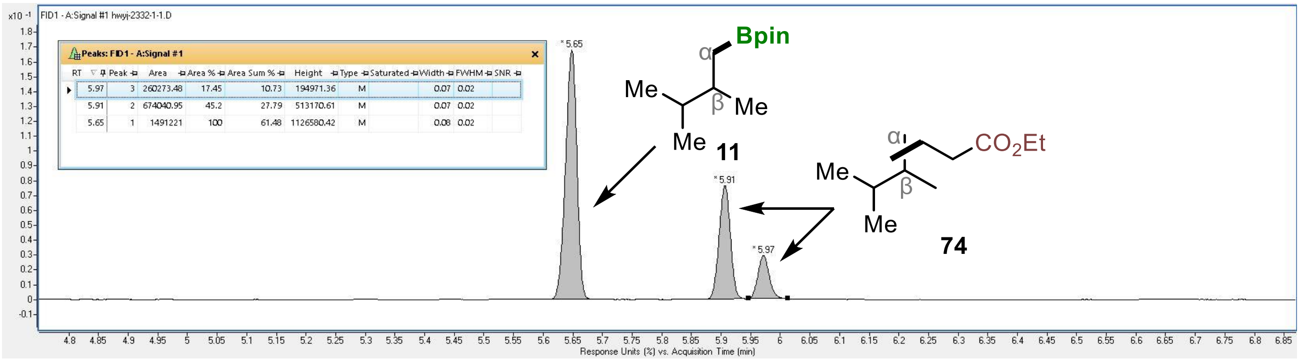

GC-FID spectrum of competition experiment of THF without B_2cat_2 (*see procedure*)

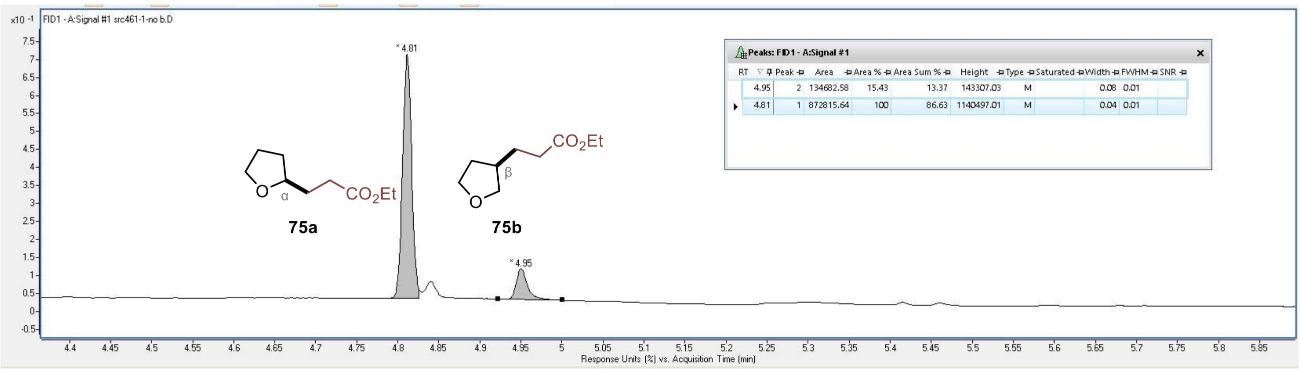

GC-FID spectrum of competition experiment of THF with B_2cat_2 (***see procedure***)

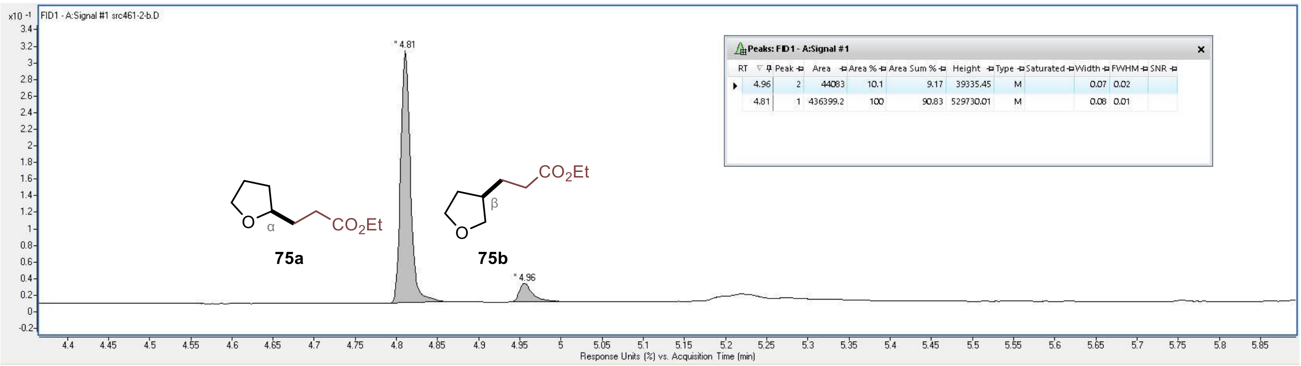

GC-FID spectrum of **77** (***see procedure***)

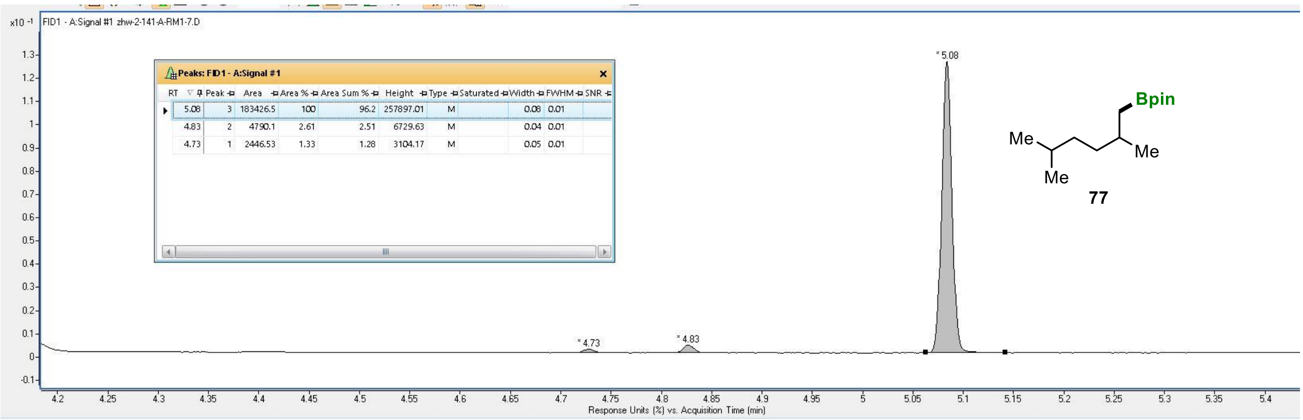

GC-FID spectrum of intermolecular competition experiment for KIE determination without pre-stirring (***see procedure***)

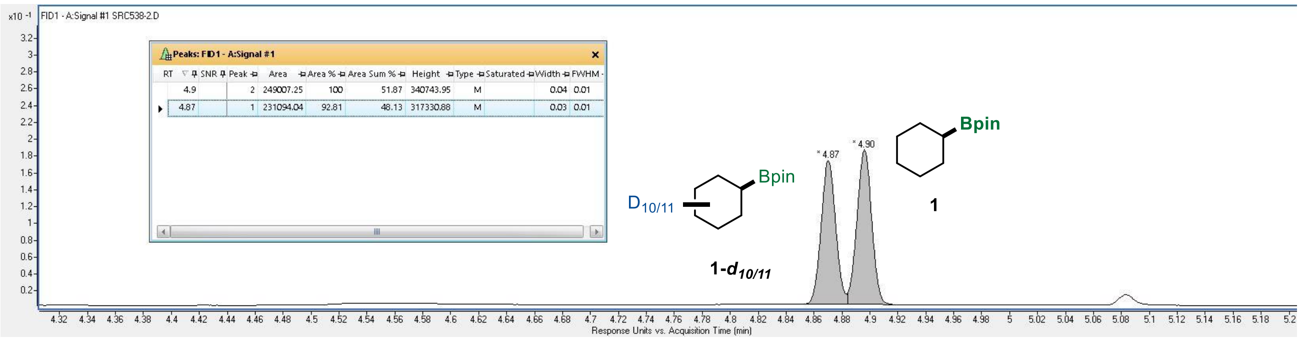

GC-FID spectrum of intermolecular competition experiment for KIE determination with pre-stirring (***see procedure***)

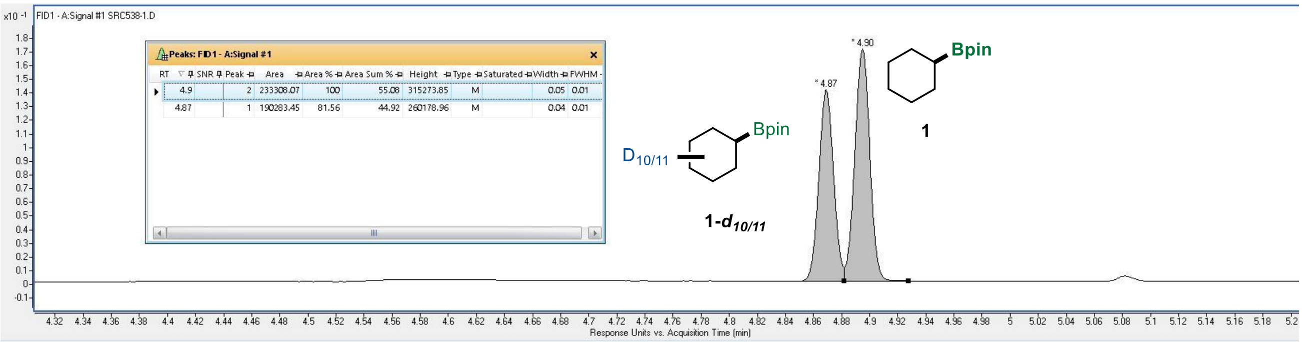

7. REFERENCES

1. Mukherjee, S.; Maji, B.; Tlahuext-Aca, A.; Glorius, F., Visible-Light-Promoted Activation of Unactivated C(sp^3)-H Bonds and Their Selective Trifluoromethylthiolation. *J. Am. Chem. Soc.* **2016**, *138*, 16200–16203.
2. Yang, M.; Su, B.; Wang, Y.; Chen, K.; Jiang, X.; Zhang, Y. F.; Zhang, X. S.; Chen, G.; Cheng, Y.; Cao, Z.; Guo, Q. Y.; Wang, L.; Shi, Z. J., Silver-Catalysed Direct Amination of Unactivated C-H Bonds of Functionalized Molecules. *Nat. Commun.* **2014**, *5*, 4707–4712.
3. Shu, C.; Noble, A.; Aggarwal, V. K., Metal-Free Photoinduced C(sp^3)-H Borylation of Alkanes. *Nature* **2020**, *586*, 714–719.
4. van der Vlugt, J. I.; Bonet, J. M.; Mills, A. M.; Spek, A. L.; Vogt, D., Modular Diphosphine Ligands Based on Bisphenol A Backbones. *Tetrahedron Lett.* **2003**, *44*, 4389–4392.
5. Svec, R. L.; Hergenrother, P. J., Imidazotetrazines as Weighable Diazomethane Surrogates for Esterifications and Cyclopropanations. *Angew. Chem. Int. Ed.* **2020**, *59*, 1857–1862.
6. Tu, J.; Hu, A.; Guo, L.; Xia, W.; Iron-Catalyzed C(Sp^3)–H Borylation, Thiolation, and Sulfinylation Enabled by Photoinduced Ligand-to-Metal Charge Transfer. *J. Am. Chem. Soc.* **2023**, *145*, 7600-7611.
7. Zhang, L.; Wu, Z. Q.; Jiao, L., Photoinduced Radical Borylation of Alkyl Bromides Catalyzed by 4-Phenylpyridine. *Angew. Chem. Int. Ed.* **2020**, *59*, 2095–2099.
8. Shu, C.; Noble, A.; Aggarwal, V. K., Photoredox-Catalyzed Cyclobutane Synthesis by a Deboronative Radical Addition-Polar Cyclization Cascade. *Angew. Chem. Int. Ed.* **2019**, *58*, 3870–3874.
9. Wu, J.; He, L.; Noble, A.; Aggarwal, V. K., Photoinduced Deaminative Borylation of Alkylamines. *J. Am. Chem. Soc.* **2018**, *140*, 10700–10704.
10. Bose, S. K.; Brand, S.; Omoregie, H. O.; Haehnel, M.; Maier, J.; Bringmann, G.; Marder, T. B., Highly Efficient Synthesis of Alkylboronate Esters via Cu(II)-Catalyzed Borylation of Unactivated Alkyl Bromides and Chlorides in Air. *ACS Catal.* **2016**, *6*, 8332–8335.
11. Reichle, M. A.; Breit, B., Preparation of Alkylmagnesium Reagents from Alkenes Through Hydroboration and Boron-magnesium Exchange. *Angew. Chem. Int. Ed.* **2012**, *51*, 5730–5734.
12. Fawcett, A.; Pradeilles, J.; Wang, Y.; Mutsuga, T.; Myers, E. L.; Aggarwal, V. K., Photoinduced Decarboxylative Borylation of Carboxylic Acids. *Science* **2017**, *357*, 283–286.
13. Ohmura, T.; Torigoe, T.; Suginome, M., Iridium-Catalysed Borylation of Sterically Hindered C(sp^3)-H bonds: Remarkable Rate Acceleration by a Catalytic Amount of Potassium *tert*-Butoxide. *Chem. Commun.* **2014**, *50*, 6333–6336.
14. Oeschger, R.; Su, B.; Yu, I.; Ehinger, C.; Romero, E.; He, S.; Hartwig, J., Diverse Functionalization of Strong Alkyl C-H Bonds by Undirected Borylation. *Science* **2020**, *368*, 736–741.
15. Hu, J.; Wang, G.; Li, S.; Shi, Z., Selective C-N Borylation of Alkyl Amines Promoted by Lewis Base. *Angew. Chem. Int. Ed.* **2018**, *57*, 15227–15231.
16. Cao, H.; Kuang, Y.; Shi, X.; Wong, K. L.; Tan, B. B.; Kwan, J. M. C.; Liu, X.; Wu, J., Photoinduced Site-selective Alkenylation of Alkanes and Aldehydes with Aryl Alkenes. *Nat. Commun.* **2020**, *11*, 1956.
17. Jones, M. R.; Fast, C. D.; Schley, N. D., Iridium-Catalyzed sp^3 C-H Borylation in Hydrocarbon Solvent Enabled by 2,2'-Dipyridylarylmethane Ligands. *J. Am. Chem. Soc.* **2020**, *142*, 6488–6492.
18. Oeschger, R.; Su, B.; Yu, I.; Ehinger, C.; Romero, E.; He, S.; Hartwig, J., Diverse Functionalization of Strong Alkyl C-H Bonds by Undirected Borylation. *Science* **2020**, *368*, 736–741.
19. Sole, C.; Tatla, A.; Mata, J. A.; Whiting, A.; Gulyas, H.; Fernandez, E., Catalytic 1,3-Difunctionalisation of

Organic Backbones through a Highly Stereoselective, One-pot, Boron Conjugate-addition/Reduction/Oxidation Process. *Chem. Eur. J.* **2011**, *17*, 14248-14257.

20. Wessig, P.; Mollnitz, K., Nanoscale Molecular Rods with a New Building Block for Solubility Enhancement. *J. Org. Chem.* **2008**, *73*, 4452–4457.
21. Lacheretz, R.; Pardo, D. G.; Cossy, J., Daucus Carota Mediated-Reduction of Cyclic 3-Oxo-amines. *Org. Lett.* **2009**, *11*, 1245–1248.
22. Wang, X.; Cui, P.; Xia, C.; Wu, L., Catalytic Boration of Alkyl Halides with Borane without Hydrodehalogenation Enabled by Titanium Catalyst. *Angew. Chem. Int. Ed.* **2021**, *60*, 12298–12303.
23. Stok, J. E.; Hall, E. A.; Stone, I. S. J.; Noble, M. C.; Wong, S. H.; Bell, S. G.; De Voss, J. J., In vivo and in vitro Hydroxylation of Cineole and Camphor by Cytochromes P450CYP101A1, CYP101B1 and N242A CYP176A1. *J. Mol. Catal. B: Enzym.* **2016**, *128*, 52–64.
24. Matsumoto, M.; Espenson, J. H., Kinetics of the interconversion of parahydrogen and orthohydrogen catalyzed by paramagnetic complex ions. *J. Am. Chem. Soc.* **2005**, *127*, 11447–11453.
25. Treacy, S. M.; Rovis, T., Copper Catalyzed C(sp^3)-H Bond Alkylation via Photoinduced Ligand-to-Metal Charge Transfer. *J. Am. Chem. Soc.* **2021**, *143*, 2729-2735.
26. Wappes, E. A.; Nakafuku, K. M.; Nagib, D. A., Directed β C–H Amination of Alcohols via Radical Relay Chaperones. *J. Am. Chem. Soc.* **2017**, *139*, 10204–10207.
27. Jin, Y.; Wang, L.; Zhang, Q.; Zhang, Y.; Liao, Q.; Duan, C., Photo-induced Direct Alkynylation of Methane and Other Light Alkanes by Iron Catalysis. *Green Chem.* **2021**, *23*, 9406–9411.
28. Yang, Q.; Wang, Y. H.; Qiao, Y.; Gau, M.; Carroll, P. J.; Walsh, P. J.; Schelter, E. J., Photocatalytic C-H Activation and the Subtle Role of Chlorine Radical Complexation in Reactivity. *Science* **2021**, *372*, 847–852.
29. Russell, G. A., Solvent Effects in the Reactions of Free Radicals and Atoms. III. Effects of Solvents in the Competitive Photochlorination of Hydrocarbons and Their Derivatives. *J. Am. Chem. Soc.* **1958**, *80*, 4997–5001.
30. Baban, J. A.; Goodchild, N. J.; Roberts, B. P., Electron Spin Resonance Studies of Radicals Derived from 1,3,2-Benzodioxaboroles. *J. Chem. Soc., Perkin Trans. 2,* **1986**, 157–161.
31. Perdew, J. P.; Ernzerhof, M.; Burke, K., Rationale for mixing exact exchange with density functional approximations. *J. Chem. Phys.* **1996**, *105*, 9982–9985.
32. Adamo, C.; Barone, V. Toward reliable density functional methods without adjustable parameters: The PBE0 model. *J. Chem. Phys.* **1999**, *110*, 6158–6170.
33. Weigend, F.; Ahlrichs, R., Balanced basis sets of split valence, triple zeta valence and quadruple zeta valence quality for H to Rn: Design an assessment of accuracy. *Phys. Chem. Chem. Phys.* **2005**, *7*, 3297–3305.
34. Grimme, S.; Antony, J.; Ehrlich, S.; Krieg, H. A consistent and accurate ab initio parametrization of density functional dispersion correction (DFT-D) for the 94 elements H-Pu. *J. Chem. Phys.* **2010**, *132*, 154104.
35. Gaussian 16, Revision A.03, Frisch, M. J.; Trucks, G. W.; Schlegel, H. B.; Scuseria, G. E.; Robb, M. A.; Cheeseman, J. R.; Scalmani, G.; Barone, V.; Petersson, G. A.; Nakatsuji, H.; Li, X.; Caricato, M.; Marenich, A. V.; Bloino, J.; Janesko, B. G.; Gomperts, R.; Mennucci, B.; Hratchian, H. P.; Ortiz, J. V.; Izmaylov, A. F.; Sonnenberg, J. L.; Williams-Young, D.; Ding, F.; Lipparini, F.; Egidi, F.; Goings, J.; Peng, B.; Petrone, A.; Henderson, T.; Ranasinghe, D.; Zakrzewski, V. G.; Gao, J.; Rega, N.; Zheng, G.; Liang, W.; Hada, M.; Ehara, M.; Toyota, K.; Fukuda, R.; Hasegawa, J.; Ishida, M.; Nakajima, T.; Honda, Y.; Kitao, O.; Nakai, H.;

Vreven, T.; Throssell, K.; Montgomery, J. A., Jr.; Peralta, J. E.; Ogliaro, F.; Bearpark, M. J.; Heyd, J. J.; Brothers, E. N.; Kudin, K. N.; Staroverov, V. N.; Keith, T. A.; Kobayashi, R.; Normand, J.; Raghavachari, K.; Rendell, A. P.; Burant, J. C.; Iyengar, S. S.; Tomasi, J.; Cossi, M.; Millam, J. M.; Klene, M.; Adamo, C.; Cammi, R.; Ochterski, J. W.; Martin, R. L.; Morokuma, K.; Farkas, O.; Foresman, J. B.; Fox, D. J. Gaussian, Inc., Wallingford CT, 2016.

36. Cheng, Y.; Mück-Lichtenfeld, C.; Studer, A., Transition Metal-Free 1,2-Carboboration of Unactivated Alkenes. *J. Am. Chem. Soc.* **2018**, *140*, 6221–6225.

37. Gonzalez, C.; Schlegel, H. B., Reaction Path Following in Mass-Weighted Internal Coordinates. *J. Phys. Chem.* **1990**, *94*, 5523–5527.

38. Fukui, K., The Path of Chemical Reactions - The IRC Approach. *Acc. Chem. Res.* **1981**, *14*, 363–368.

39. Maeda, S.; Harabuchi, Y.; Ono, Y.; Taketsugu, T.; Morokuma, K., Intrinsic Reaction Coordinate: Calculation, Bifurcation, and Automated Search. *Int. J. Quantum Chem.* **2015**, *115*, 258–269.
